# Palladium‐Catalyzed Dual Csp^2^─Csp^3^ Bond Formation: A Versatile Platform for the Synthesis of Benzo‐Fused Heterocycles

**DOI:** 10.1002/advs.202500897

**Published:** 2025-04-28

**Authors:** Jiahui Huang, Yuantao You, Yijian Ma, Xingying He, Yixiao Li, Arunachalam Kesavan, Chengzhi Jin, Chengshuo Shen, Min Zhang, Kedong Yuan

**Affiliations:** ^1^ Guangzhou Municipal and Guangdong Provincial Key Laboratory of Molecular Target & Clinical Pharmacology the NMPA and State Key Laboratory of Respiratory Disease School of Pharmaceutical Sciences Guangzhou Medical University Guangzhou 511436 China; ^2^ School of Chemistry and Chemical Engineering Zhejiang Sci‐Tech University Hangzhou Zhejiang 310018 China; ^3^ Key Lab of Functional Molecular Engineering of Guangdong Province School of Chemistry and Chemical Engineering South China University of Technology Guangzhou 510641 China

**Keywords:** arylsulfonyl chlorides, cyclization, heterocycles, palladium catalysis

## Abstract

Transition‐metal‐catalyzed transformations offer a powerful approach to rapidly synthesize complex benzo‐fused heterocycles, crucial for drug and material development. However, existing synthetic strategies face challenges such as limited functional group compatibility, reliance on complex ligands, and difficulties in controlling chemoselectivity with prefunctionalized substrates. Herein, a ligand‐free Pd(II)/Cu(I) catalytic system is presented that facilitates reactions between arylsulfonyl chlorides and unactivated olefins under mild conditions, enabling the efficient synthesis of saturated benzo‐fused six‐membered heterocycles. This streamlined strategy employs dual Csp^2^─Csp^3^ bond formation, producing diverse N/O‐polyheterocycles and allowing late‐stage functionalization of bioactive molecules with excellent yields and high chemoselectivity. The key to the success of this reaction is the formation of high‐valent Ar‐Pd(III) intermediate, which drives the reaction through 1,2‐Pd migration and electrophilic C─H arylation. This unique reactivity pathway facilitates the formation of benzo‐fused heterocycles while effectively avoiding the β‐H elimination typically associated with Heck‐type reactions.

## Graphical abstract

Pd(II)/Cu(I) co‐catalyzed dual Csp^2^─Csp^3^ bonds construction from unactivated alkenes and arylsulfonyl chlorides to access benzo‐fused heterocycles with high efficiency and chemoselectivity.

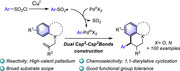

## 1 Introduction

**Figure 1:**
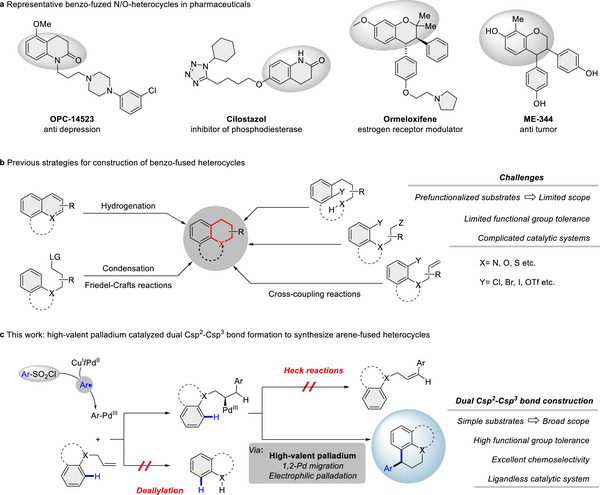
Importance of benzo‐fused heterocycles and synthetic strategies.

The synthesis of benzo‐fused heterocycles, which allows rapid access to highly functionalized molecules, is vital for developments in pharmaceuticals. Their inherent structural versatility and intricate ring systems facilitate the integration of diverse functional groups, offering enhanced stability and favorable electronic properties with broad potential applications (**Figure** **1**a).^[^ ^1^ ^]^ Therefore, various synthetic strategies have been developed to meet the increasing demand for assembling complex benzo‐fused heterocycles from different substrates (Preparation of benzo‐fused heterocycles, see ref. [2]). Benzo‐fused heterocycles can be synthesized through arene hydrogenation^[^ ^3^ ^]^ or intramolecular Friedel‐Crafts condensation reactions^[^ ^4^ ^]^ (Figure 1b, left). However, these methods often limit molecular diversity due to the need for pre‐functionalized substrates. Transition metal catalysis enables accurate and chemoselective intramolecular cyclization of aryl halide derivatives and alkenes, efficiently constructing benzo‐fused heterocycles via metal‐ligand bifunctional pathways (Figure 1b, right) (Transition metal‐catalyzed synthesis of benzo‐fused heterocycles, see ref. [5]). These reactions are valued for facilitating aryl or alkyl group migration to form well‐defined benzo‐fused rings (Selected reviews, see ref. [6]). However, challenges remain in terms of functional group tolerance, ligand requirements, scalability, and the need for strict endo/exo stereopreference^[^ ^7^ ^]^ to avoid undesired products. Developing a streamlined method that enhances functional group compatibility and reaction scope is crucial for expanding the diversity of benzo‐fused heterocycles.

Recent advancements in high‐valent palladium catalysis^[^ ^8^ ^]^ offer an alternative approach, increasing efficiency and expanding the range of accessible compounds through electrophilic C(sp^2^)‐H functionalization of both arenes and alkenes.^[^ ^9^ ^]^ Traditionally, reactive high‐valent metal intermediates are generated using oxidants, which facilitate the incorporation of various functional groups derived from these oxidants.^[^ ^7^, ^10^ ^]^ Another solution involves intercepting a radical species with metal catalysts, such as Pd(II) or Ni(II), to promote the formation of reactive intermediates and stabilize transition states.^[^ ^11^ ^]^ This process entails the migration of an aryl or alkyl group to an adjacent alkene linked to a benzene ring, enabling cyclization to form benzo‐fused structures^[^ ^12^ ^]^ while addressing challenges such as functional group tolerance and regioselectivity. Inspired by transition‐metal‐catalyzed inter‐molecular migrative difunctionalization of alkenes,^[^ ^13^ ^]^ we envisioned that combining high‐valent transition metal catalysis with migrative alkene functionalization using a radical precursor could provide efficient access to complex heterocyclic architectures. In practice, arylsulfonyl chlorides, as oxidative arylating reagents, exhibit excellent orthogonality in synthesis and compatibility with palladium catalysis,^[^ ^14^ ^]^ making them highly effective for desulfitative arylation reactions. Their high reactivity and versatility as internal oxidants facilitate the formation of high‐valent metal species^[^ ^15^ ^]^ and electrophilic radical precursors for reaction design. Moreover, their compatibility with various functional groups, including C‐halogen bonds, allows for the incorporation of diverse substituents, enhancing both the complexity and functionality of the resulting heterocycles.

Herein, we present a general and chemoselective method for the construction of benzo‐fused heterocycles via a Pd(II)/Cu(I) co‐catalyzed migratory 1,1‐diarylation process. This approach enables the simultaneous formation of two Csp^2^–Csp^3^ bonds in a single‐pot reaction. Notably, it circumvents intrinsic challenges associated with Heck reactions and deallylation side reactions,^[^ ^16^ ^]^ while also addressing the previous substrate limitations to N‐allyl sulfonamides^[^ ^17^ ^]^ (Figure 1c). This advancement significantly expands the diversity of benzo‐fused heterocycles and highlights the potential of high‐valent palladium in migratory difunctionalization of alkenes.

## 2 Results and Discussion

Our study commenced by exploring the reactivities between 4‐methylbenzenesulfonyl chloride (**1**) and 4‐allyl‐2H‐benzo[b][1,4]oxazin‐3(4H)‐one (**2**) under various reaction conditions (**Table** **1**, See also Tables S2—S5, Supporting Information). Different palladium sources were evaluated with the aim of improving chemoselectivity for the production of benzo‐fused N‐heterocycle **3**. with PdCl_2_ proving most effective, yielding the target product **3** with a 67% isolated yield (entries 1–3). Notably, Heck‐type products **4** and **5** were isolated in yields below 15%, and no product formed without Pd, confirming its essential role in the desulfitative transformation (entry 4). Lower‐valent metal species can activate arylsulfonyl chlorides to generate radical species,^[^ ^18^ ^]^ so we explored Cu(I) and Fe(II) additives to assess radical accumulation, which successfully facilitated the cyclization (entries 5–6). A control experiment showed that the absence of Cu(I) still led to the formation of product **3**, albeit with a lower yield (entry 7). Next, a series of inorganic bases were tested, identifying Li_2_CO_3_ as the most efficency, while Pd‐loading was lowered to 2.5 mol% without compromising yield or chemoselectivity, delivering cyclization product **3** with a 72% isolated yield and chemoselectivity up to 9:1 (entries 8–10). Solvent and temperature significantly influenced reactivity and chemoselectivity, with the yield of **3** further increasing to 78% when refluxed in DMC at 130 °C under N_2_ atmosphere for 14 h (entries 11−15).

**Table 1:** Condition optimization for chemoselective synthesis of benzo‐fused *N*‐heterocycles **3**.

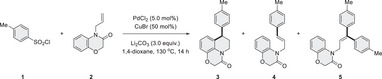			

Entry^a)^	Variation from the given conditions	Ratio of 3/4/5 [%]	Yield of 3 [%]^b)^
1	PdCl_2_	28:1:5	76% (67%)
2	[PdCl(Allyl)]_2_	39:1:10	71%
3	Pd(OPiv)_2_	24:1:8	65%
4	No [Pd]	‐	0
5	CuTc in lieu of CuBr	14:1:3	66%
6	FeCl_2_ in lieu of CuBr	9:7:1	46%
7	No [Cu]	1:6:0	12%
8	Na_2_CO_3_ in lieu of Li_2_CO_3_	24:25:1	37%
9	Decreasing CuBr loading to 20 mol%	26:1:7	71%
10	PdCl_2_ 2.5 mol%	7:0:1	80% (72%)
11	PdCl_2_ 2.5 mol%, reaction in MeNO_2_	4:0:1	74%
12	PdCl_2_ 2.5 mol%, reaction in DMC	9:0:1	91% (78%)
13	100 ^o^C	‐	n.r.
14	120 ^o^C	6:0:1	80%
15	140 oC	9:0:1	81%

^a)^ Reaction conditions: A mixture of **1** (0.75 mmol, 1.5 equiv.), **2** (0.5 mmol, 1.0 equiv.), [Pd] cat. (5.0 mol%), [Cu] cat. (50 mol%), base (1.5 mmol, 3.0 equiv.) in solvent (2.0 mL, 0.4 m) was heated at 130 °C under N_2_ for 14 h.

^b)^ The yield was determined by crude ^1^H NMR analysis using 0.1 mmol 1,3,5‐trimethoxybenzene as an internal standard, isolated yield in parenthesis.

With optimal reaction conditions established, we first investigated the reactivities of different arylsulfonyl chlorides to construct various benzo‐fused *N*‐heterocycles (**Table** **2**). A broad range of *para*‐substituted arylsulfonyl chlorides demonstrated good reactivity, affording aryl‐substituted triheterocycles **6**–**24** with excellent chemoselectivity and in good isolated yields (57–83%). Notably, arylsulfonyl chlorides bearing synthetically useful functional groups, such as trifluoromethyl (**17**), ketone (**18**), cyano (**20**), and halides (**22**‐**24**) on the aromatic ring, were well tolerated. Other substitution pattern was also examined. For instance, arylsulfonyl chlorides with alkyl or halide groups at the meta‐position (**25**‐**29**) of the aromatic ring reacted smoothly, yielding the corresponding products in 65–77%. Orth‐substituted and poly‐substituted arylsulfonyl chlorides also successfully afforded the desired cyclization products (**30‐37**). Notably, the absolute configuration of compound **34** was unambiguously established by X‐ray crystallography. Interestingly, (hetero)arylsulfonyl chlorides were successfully employed in the reaction, delivering annulation products (**38‐43**) with moderate yields (37‐67%). Additionally, an alkenylsulfonyl chloride proved reactive, forming the corresponding olefinated triheterocycle **44** in a satisfactory yield.

**Table 2:** Scope variation from arylsulfonyl chlorides (See table footnotes).

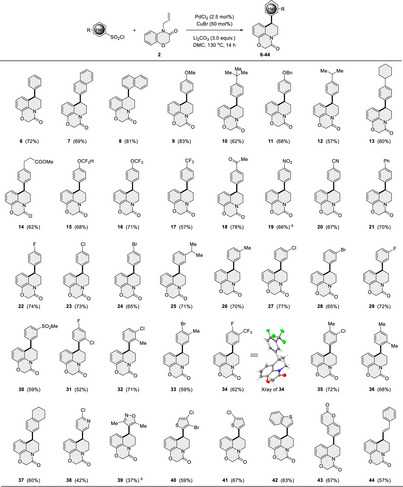

^a)^Reaction conditions: arylsulfonyl chlorides (0.75 mmol), **2** (0.50 mmol), PdCl_2_ (2.5 mol %), CuBr (50 mol %), and Li_2_CO_3_ (1.50 mmol) in DMC (2.0 mL) at 130 °C for 14 h. Yields are of isolated products.

^b)^Reaction performed in MeNO_2_.

Subsequently, several *N*‐allyl lactams and **1** were tested under the optimized conditions to further explore the scope of this transformation (**Table** **3**). Pleasingly, a wide range of substituted aromatic or heteroaromatic rings all occurred well (**45−56**). Intriguingly, this strategy was successfully applied to the construction of aryl‐substituted polyheterocycles containing three heteroatoms (N, O, and S) by simply combining compound **1** with *N*‐allyl substrates (**57−65**). This method offers a novel and general route for synthesizing polyheterocycle products with diverse substituents on both coupling partners. Besides, substrates derived from phenoxazine and phenothiazine were effectively tolerated, with the reaction occurring chemoselectively on the more electrophilic arenes, yielding the corresponding products in moderate yields (**66‐67**). To our delight, reactions between *O*‐allyl phenol derivatives and arylsulfonyl chlorides proceeded smoothly without the need for further optimization, and no Heck products were detected in these cases. For instance, simple *O*‐allyl phenol exhibited higher reactivities and chemoselectivity, favoring the formation of 4‐arylbenzohydropurans (**68‐78**), with cyclization preferentially occurring at the less hindered positions (**79**). Complex structures, such as pyrene, coumarin derivatives, and dibenzofuran, were successfully incorporated, enabling the construction of intricate polycyclic structures (**80**‐**83**).

**Table 3:** Scope variation from allylic substrates (See table footnotes).

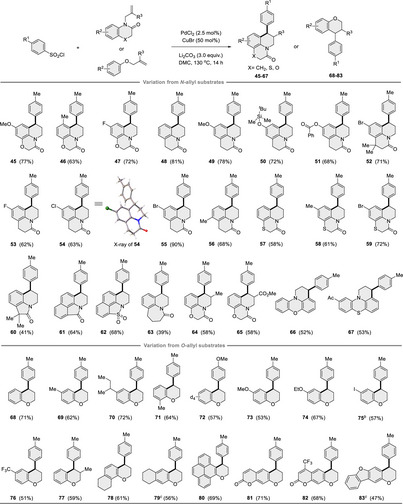

^a)^Reaction conditions: *N/O*‐allyl substrates (0.50 mmol) and arylsulfonyl chlorides (0.75 mmol), PdCl_2_ (2.5 mol %), CuBr (50 mol %), and Li_2_CO_3_ (1.50 mmol) in DMC (2.0 mL) at 130 °C for 14 h.

^b)^Halogen exchange was observed. *^c^* Compounds **79** and **83** were obtained as a mixture of regioisomers (**79**, C1:C3 = 1:2; **83**, C1:C3 = 1:2).

**Figure 2:**
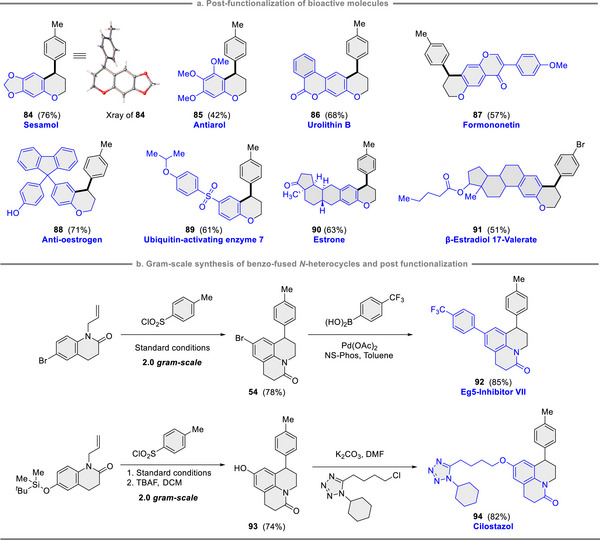
Post‐functionalization of drug molecules.

The applications of our ligandless catalytic system were further evaluated using quinolinone and phenol derivatives with notable biological functions. As shown in **Figure** **2**a, complex molecules such as urolithin B, chromone, and steroids were successfully incorporated, yielding the corresponding cyclization products (**84**‐**91**). Notably, scaling the reaction up to a 2.0‐gram scale maintained the efficiency of cyclization product formation (**54** and **93**), demonstrating the method's scalability for practical applications. The freshly obtained products also proved versatile as synthons for the late‐stage functionalization of drug‐like compounds (**92** and **94**, Figure 2b). This highlights the potential of this catalytic system to advance drug discovery processes.

**Figure 3:**
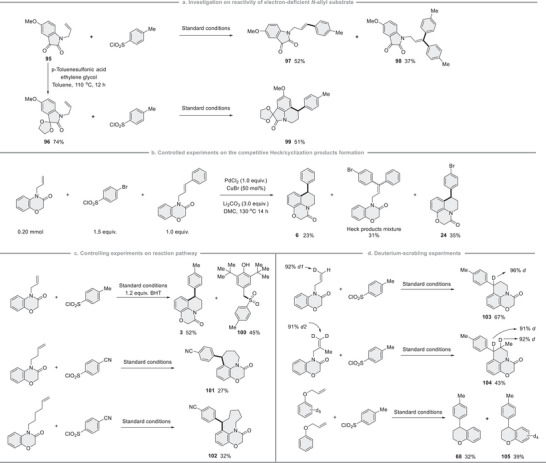
Controlling experiments for mechanistic studies.

Several experiments were conducted to gain a better understanding of the reaction mechanism, as outlined in Figure 5. When the indoline‐2,3‐dione substrate **95** was subjected to the standard conditions, only Heck‐type products **97** and **98** were obtained in high yields. However, the reaction between **1** and the freshly prepared acetal compound **96** afforded the expected cyclization product **99** in a 51% isolated yield. These results clearly indicate that palladium intermediates preferentially attack electron‐rich (hetero)arenes (**Figure** **3**a). Subsequently, real‐time monitoring of reaction mixture and a series combinational experiments were conducted to elucidate the reaction pathway leading to the formation of different products. For instance, it was confirmed that cyclization product **6** could be generated from the mono Heck product, which subsequently converts into product **24** through a Pd‐H intermediate (Figure 3b; see Schemes S11–S13, Supporting Information for more details). Notably, an aryl sulfone radical^[^ ^18a^ ^]^ was successfully trapped using BHT as a radical scavenger. Additionally, the reaction left the C‐halogen bonds intact, deviating from the typical Pd(0)/Pd(II) catalytic cycle. These findings provide compelling evidence for the involvement of a radical pathway and further support the formation of high‐valent palladium species^[^ ^14^, ^15^ ^]^ as key intermediates. Interestingly, extending the carbon chain of the terminal alkenes on the corresponding substrates consistently yielded the expected cyclization products **101** and **102**, further validating the role of the Pd‐1,2 migrative reaction pathway in the synthesis of benzo‐fused heterocycles. This process enables the efficient construction of dual Csp^2^–Csp^3^ bonds, showcasing the robustness and adaptability of the catalytic system for generating complex molecular frameworks (Figure 3c). It is noteworthy that steric hindrance and the surrounding substitution environment interfere with the chain walking process. When using internal alkenes instead of allyl moieties on the substrates, Heck‐type products emerged as the major products. Finally, deuterium scrambling experiments provided further evidence supporting the Pd‐1,2‐migrative process. The high‐valent palladium species likely undergoes an intramolecular electrophilic C─H arylation via a seven‐membered palladacycle^[^ ^19^ ^]^ to yield the cyclized products. Such a mechanism contrasts with the formation of Heck‐type products, which would typically arise through β‐H elimination (Figure 3d).

**Figure 4:**
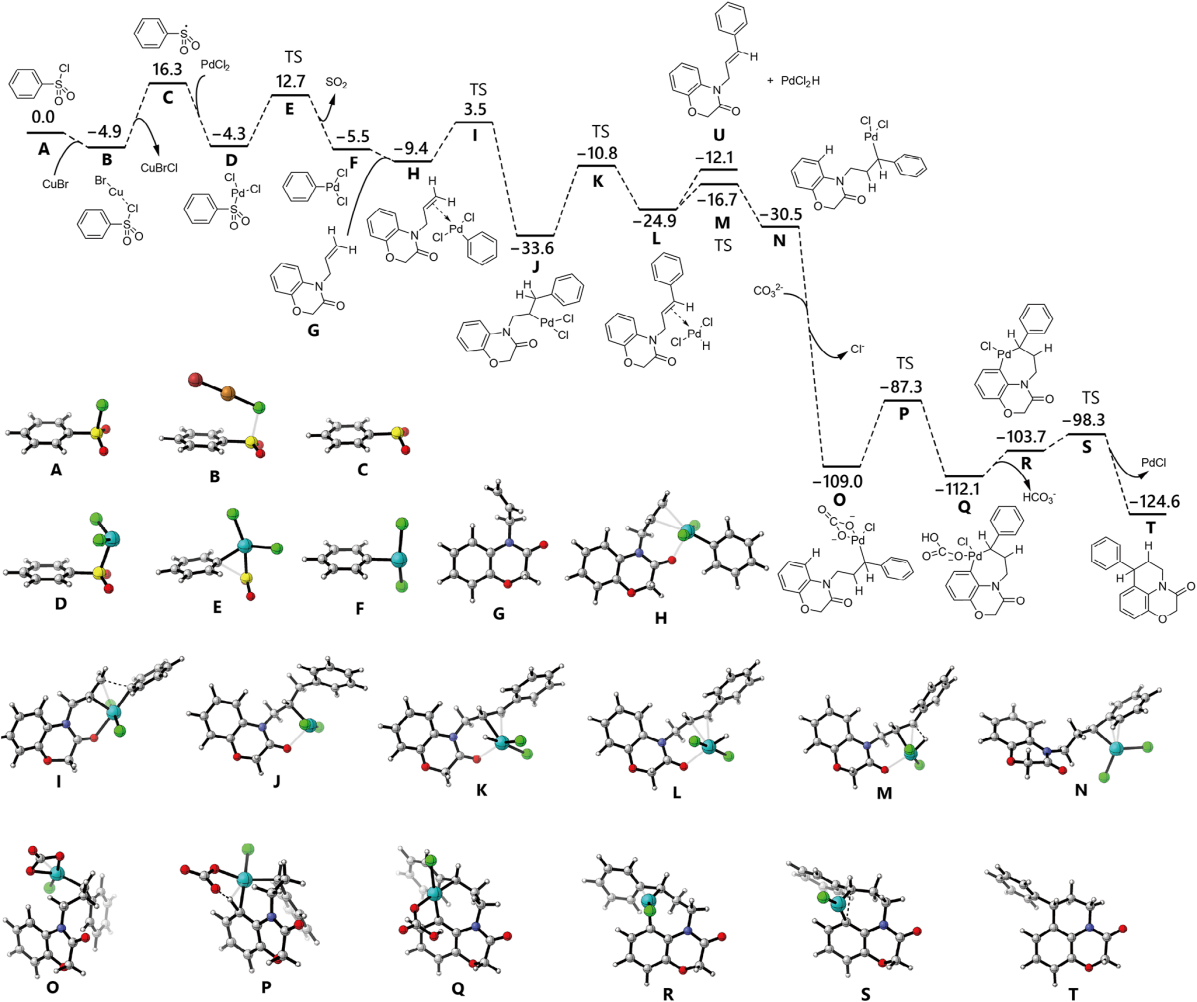
Theoretical calculations of reaction mechanism. DFT calculations were carried out at the PBE0‐D3(BJ)/ma‐ TZVPP//PBE0‐D3(BJ)/def‐TZVP level of theory. Free energies referring to the change of free energy in the corresponding reactions are given in kcal mol−.

**Figure 5:**
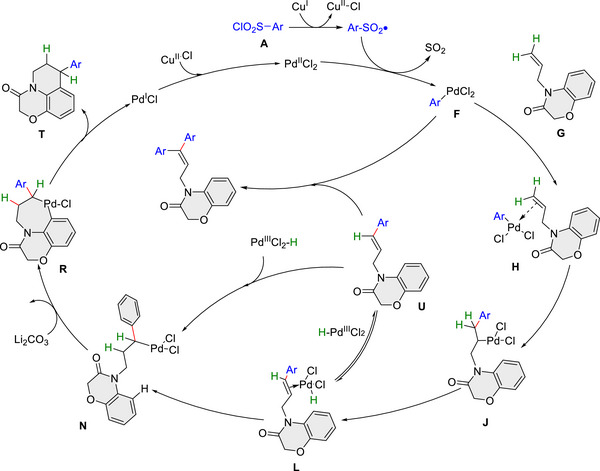
Proposed catalytic cycle.

Density functional theory (DFT) calculations (**Figure** **4**) reveal the reaction process starts from the formation of a phenyl sulfone radical (**C**) facilitated by CuBr. This radical **C** then interacted with PdCl_2_, producing a Pd(III) species (For Pd(III) complexes, see ref. [20]) (**D)** and releasing SO_2_, thus yielding a Ph‐Pd(III) derivative (**F**). Complex **F** subsequently coordinates with the C═C bond of the substrate (**G**), forming complex (**H**). Complex **H** is transformed into intermediate **J** with an activation free energy of 12.9 kcal mol^−1^, wherein the phenyl group migrates from the Pd center to the terminus of the C═C bond. Following this, intermediate **J** transformed into another C═C‐bond‐coordinated Pd(III) complex (**L)**, accompanied by a hydride shift to the Pd(III) center at an activation energy of 22.7 kcal mol^−1^. Complex **L** demonstrates greater stability compared to its precursor **H**, indicated by a lower free energy (−25.0 vs −9.5 kcal mol^−1^), suggesting a strong preference for phenyl transfer onto the C═C bond. Subsequently, complex **L** transforms into intermediate **N**, wherein the hydrogen atom is repositioned within the substrate backbone. A ligand exchange occurs between carbonate and chloride, yielding a more stable structure (**O**). The critical C─H activation on the aromatic ring occurs with an activation energy of 21.7 kcal mol^−1^, leading to the cyclized intermediate (**Q**) via transition state (**P**). The subsequent elimination of bicarbonate and PdCl results in final product (**T**), with a total free energy release of 125.6 kcal mol^−1^. Notably, during the conversion from **L** to **N**, an alternative Heck product (**U**) is generated via Pd elimination. However, this pathway is associated with an increase in free energy of 12.9 kcal mol^−1^, underscoring the preference for the chemoselective formation of the cyclization product over intrinsic Heck side products. Finally, a mechanism involving high‐valent palladium and migratory 1,1‐diarylation of unactivated alkenes is proposed for the Pd/Cu co‐catalyzed endo‐selective synthesis of benzo‐fused heterocycles, and the synergistic role of Pd and Cu in dual Csp^2^─Csp^3^ bonds construction was shown in **Figure** **5**.

## 3 Conclusion

In summary, we have developed a general strategy to construct complex heterocyclics featuring arene‐fused six‐membered rings from readily available substrates through Pd(II)/Cu(I) co‐catalyzed cascade process. This approach, notably avoiding the use of expensive ligands and oxidants, maximizes molecular complexity in a regio‐ and chemo‐ selective manner, offering diverse access to a wide range of sophisticated heterocycles and enabling late‐stage functionalization of drug‐like molecules such as chromones and steroids. The in situ generated aryl‐Pd(III) species subsequently undergo a thermodynamically favored 1,1‐chain walking process followed by electrophilic C─H functionalization, enabling the selective construction of dual Csp^2^─Csp^3^ bonds. This pathway avoids β‐H elimination, a common side reaction, and directs the reaction toward the formation of benzo‐fused heterocycles with exceptional chemoselectivity and efficiency. We anticipate that this precisely controlled skeletal construction will pave the way for broader applications in synthetic and medicinal chemistry.

## Conflict of Interest

The authors declare no conflict of interest.

## Supporting information



Supporting Information 1

Supporting Information 2

Supporting Information 3

## Data Availability

The data that support the findings of this study are available in the supplementary material of this article.
