# Supplementary material for: Palladium‐Catalyzed Dual Csp2─Csp3 Bond Formation: A Versatile Platform for the Synthesis of Benzo‐Fused Heterocycles
Source: Adv Sci (Weinh). 2025 Apr 28;12(25):2500897. doi: 10.1002/advs.202500897 (PMC12224930; doi:10.1002/advs.202500897)
Supplement: Supplementary file 1 — Supporting Information 1 [file ADVS-12-2500897-s003.pdf]

ADVANCED SCIENCE
Open Access

Supporting Information

for *Adv. Sci.*, DOI 10.1002/advs.202500897

Palladium-Catalyzed Dual Csp^2—Csp^3 Bond Formation: A Versatile Platform for the Synthesis of Benzo-Fused Heterocycles

Jiahui Huang, Yuantao You, Yijian Ma, Xingying He, Yixiao Li, Arunachalam Kesavan, Chengzhi Jin, Chengshuo Shen, Min Zhang* and Kedong Yuan**

Supporting Information

Palladium-Catalyzed Dual Csp^2-Csp^3 Bond Formation: A Versatile Platform for the Synthesis of Benzo-Fused Heterocycles

Jiahui Huang,[a] Yuantao You,[a] Yijian Ma,[b] Xingying He,[a] Yixiao Li,[a] Arunachalam Kesavan,[a] Chengzhi Jin,[a] Chengshuo Shen,*[b] Min Zhang,*[c] and Kedong Yuan*[a]

[a] Guangzhou Municipal and Guangdong Provincial Key Laboratory of Molecular Target & Clinical Pharmacology, the NMPA and State Key Laboratory of Respiratory Disease, School of Pharmaceutical Sciences, Guangzhou Medical University, Guangzhou 511436, China.

*email: kedongyuan@gzhmu.edu.cn

[b] School of Chemistry and Chemical Engineering, Zhejiang Sci-Tech University, Hangzhou, Zhejiang, 310018, China

*email: shenchengshuo@zstu.edu.cn

[c] Key Lab of Functional Molecular Engineering of Guangdong Province, School of Chemistry and Chemical Engineering, South China University of Technology, Guangzhou 510641, China.

*email: minzhang@scut.edu.cn

General methods S1
Optimization of reaction conditions S2
Experimental procedures S6
Preparation of starting materials S7
Conditions A: S7
Conditions B: S8
General protocol for synthesis of benzo-fused heterocycles S9
Conditions C: S9
Gram-scale preparation of benzo-fused heterocycles S9
Synthetic applications S11
Mechanistic experiments S12
Real time reaction monitoring S12
Controlling experiments S12
Radical scavenger experiments S14
Synthesis of deuterated substrates and deuterium scrambling experiments S14
Characterizationdata S18
NMR spectra S100
References S251

General methods

Unless otherwise noted, all reactions were conducted in a 25 mL oven-dried Schlenk tubes, the reagents, all arylsulfonyl chlorides and solvents (chemicals) were purchased from commercial sources (J&K Scientific, Bide Pharmatech Co.,Ltd, Adamas) and used without further purification. The new products were characterized by using ^{1}H NMR, ^{13}C NMR, ^{19}F NMR and HRMS analysis. ^{1}H NMR spectra were recorded on a Bruker GPX 400 MHz spectrometer. Chemical shifts (δ) reported in parts per million relative to residual chloroform (7.26 ppm for ^{1}H NMR; 77.0 ppm for ^{13}C NMR), Coupling constants were reported in Hertz. ^{1}H NMR assignment abbreviations were the following: singlet (s), doublet (d), triplet (t), doublet of doublets (dd) and multiplet (m). ^{13}C NMR spectra (101 MHz), ^{19}F NMR spectra (376 MHz) were recorded at on the same spectrometer and reported in ppm. Known compounds were analyzed by GC-MS, ^{1}H NMR and confirmed by comparison with literature data. Mass spectra were collected at Agilent Technologies 5973N (EI). High resolution mass spectrometry (HRMS) was recorded on a Q-Tof mass analyzer with electrospray ionization (ESI) by Waters Acquity UPLC Class I/Xevo G2 Q-Tof. Tof 100–1000 m/z, Quadrupole 4000 m/z. Column chromatography was performed using Silica gel 60 (300-400 mesh), and the eluent was a mixture of petroleum ether (PE) and ethyl acetate (EA).

Optimization of reaction conditions

Table S1. Optimization of [Pd] sources for the cyclization reaction [a]

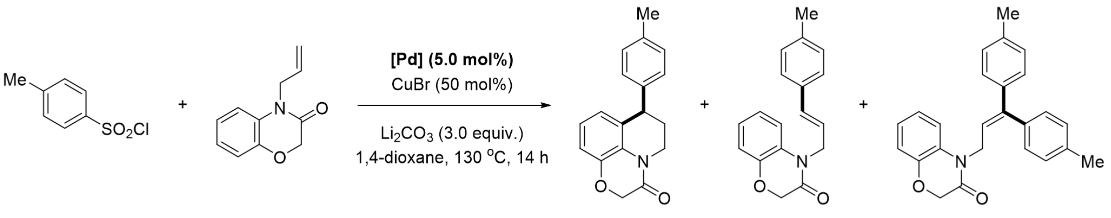

1 2 3 4 5

Entry	[Pd]-Cat.	Conversion [c]	**3:4:5**	Yield of **3** [c]
1[b]	$PdCl_2$	100%	28:1:5	76%(67%)
2	$Pd(acac)_2$	100%	3:0:1	66%
3	$Pd(ally)_2$	97%	39:1:10	71%
4	$Pd(OPiv)_2$	99%	24:1:8	65%
5	$Pd(OAc)_2$	100%	4:0:1	71%
6	No [Pd]	-	-	n.r.

[a]All the reactions were performed using **1** (0.75 mmol) and **2** (0.50 mmol) with **[Pd]** (5.0 mol %) and CuBr (50 mol %), Li_2CO_3 (3.0 equiv.) in 1,4-dioxane (2.0 mL) at 130 ˚C for 14 h under N_2 in a 25 mL sealed Schlenk tube. [b]Dealkyklation of **2** was observed. [C]Conversion and yield of **3** were measured by GC using dodecane as internal standard, isolated yields in parenthesis.

Table S2. Optimization of additives for the cyclization reaction [a]

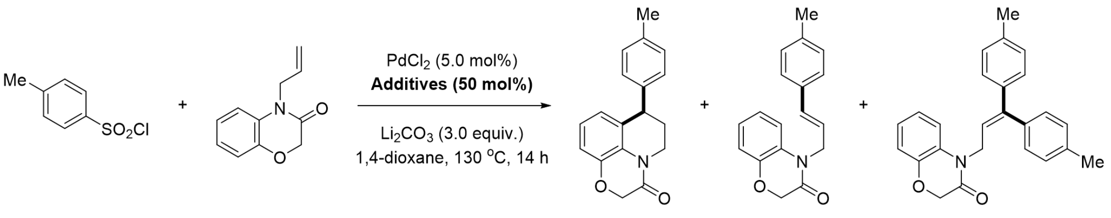

1 2 3 4 5

Entry	Additives	Conversion [c]	**3:4:5**	Yield of **3** [c]
1	CuBr	100%	6:0:1	77%(69%)
2	CuTc	100%	14:1:3	66%
3[b]	CuOAc	100%	6:7:1	38%
4	$FeCl_2$	72%	9:7:1	46%
5	No [Cu]	32%	1:6:0	12%

[a]All the reactions were performed using **1** (0.75 mmol) and **2** (0.50 mmol) with $PdCl_2$ (5.0 mol %) and **Additives** (50 mol %), Li_2CO_3 (3.0 equiv.) in 1,4-dioxane (2.0 mL) at 130 ˚C for 14 h under N_2 in a 25 mL sealed Schlenk tube. [b]Dealkyklation of **2** was observed. [c]Conversion and yield of **3** were measured by GC using dodecane as internal standard, isolated yields in parenthesis.

Table S3. Optimization of Base for the cyclization reaction [a]

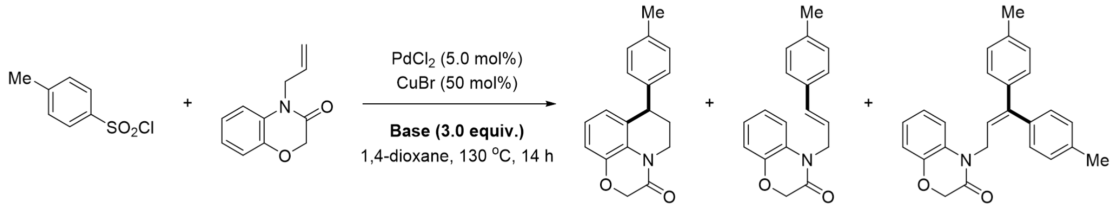

Entry	Base	Conversion [b]	**3:4:5**	Yield of **3** [b]
1	K_2CO_3	35%	12:1:0	21%
2	Na_2CO_3	98%	24:25:1	37%
3	Cs_2CO_3	-	-	n.r.
4	*t*BuOLi	-	-	n.r.
5	Li_2CO_3	100%	7:0:1	78%(71%)[c]
6	No [Li_2CO_3]	42%	1:1:1	21%

[a]All the reactions were performed using **1** (0.75 mmol) and **2** (0.50 mmol) with $PdCl_2$ (5.0 mol %) and CuBr (50 mol %), **Base** (3.0 equiv.) in 1,4-dioxane (2.0 mL) at 130 ˚C for 14 h under N_2 in a 25 mL sealed Schlenk tube. [b]Conversion and yield of **3** were measured by GC using dodecane as internal standard. [c]Isolated yields in parenthesis.

Table S4. Optimization of Solvent for the cyclization reaction [a]

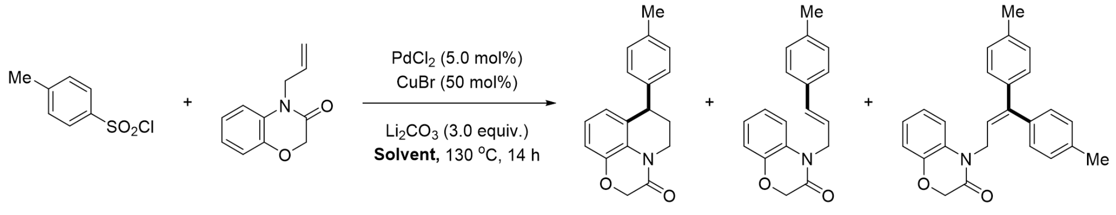

Entry	Solvent	Conversion [c]	**3:4:5**	Yield of **3** [c]
1	DMF	-	-	n.r.
2	DMSO	-	-	n.r.
3[b]	Xylenes	36%	41:1:8	25%
4	1,2-Diethoxyethane	100%	7:1:2	68%
5	1,4-dioxane	100%	7:0:1	79%(72%)

[a]All the reactions were performed using **1** (0.75 mmol) and **2** (0.50 mmol) with $PdCl_2$ (2.5 mol %) and CuBr (50 mol %), Li_2CO_3 (3.0 equiv.) in **Solvent** (2.0 mL) at 130 ˚C for 14 h under N_2 in a 25 mL sealed Schlenk tube. [b]Dealkyklation of **2** was observed. [c]Conversion and yield of **3** were measured by GC using dodecane as internal standard, isolated yields in parenthesis.

Table S5. Optimization of catalyst-loading for the cyclization reaction [a]

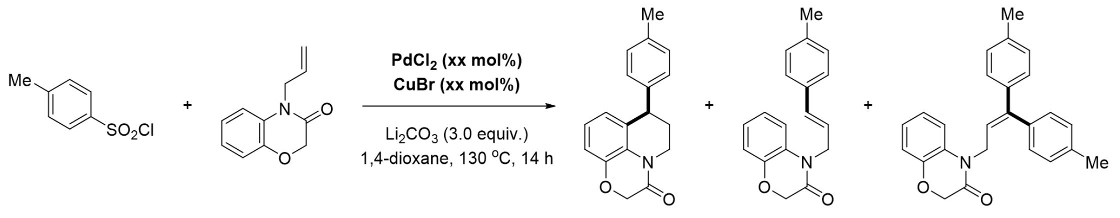

1 **2** **3** **4** **5**

Entry	catalyst-loading	Conversion [b]	**3**:**4**:**5**	Yield of **3** [b]
1	$PdCl_2$ (5.0 mol%) CuBr (50 mol%)	100%	7:0:1	79%
2	$PdCl_2$ (2.5 mol%) CuBr (50 mol%)	100%	7:0:1	80%(72%)
3	$PdCl_2$ (10 mol%) CuBr (50 mol%)	100%	5:0:1	74%
4	$PdCl_2$ (2.5 mol%) CuBr (20mol%)	100%	26:1:7	71%
5	$PdCl_2$ (2.5 mol%) CuBr (100 mol%)	100%	4:0:1	72%
6 [c]	$PdCl_2$ (2.5 mol%) CuBr (20 mol%)	100%	5:0:2	65%
7 [c]	$PdCl_2$ (2.5 mol%) CuBr (10 mol%)	100%	5:0:4	52%
8 [c]	$PdCl_2$ (2.5 mol%) CuBr (5.0 mol%)	100%	1:0:1	45%

[a]All the reactions were performed using **1** (0.75 mmol) and **2** (0.50 mmol) with **$PdCl_2$ (xx mol %)** and **CuBr (xx mol %)**, Li_2CO_3 (3.0 equiv.) in 1,4-dioxane (2.0 mL) at 130 ˚C for 14 h under N_2 in a 25 mL sealed Schlenk tube. [b]Conversion and yield of **3** were measured by GC using dodecane as internal standard, isolated yields in parenthesis.[c] Reaction in DMC.

Table S6. Optimization of Solvent for the cyclization reaction [a]

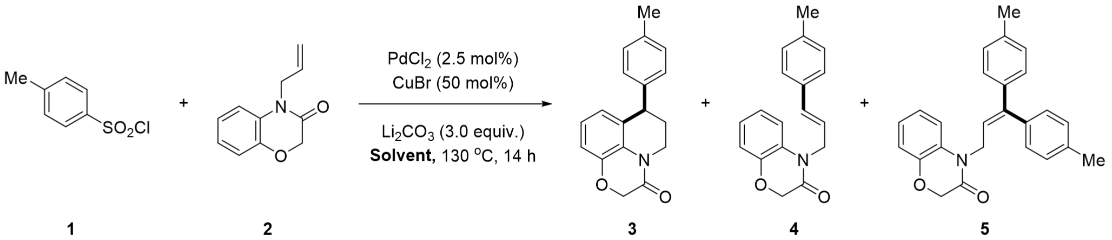

Entry	Solvent	Conversion [b]	**3:4:5**	Yield of **3** [b]
1	1,4-dioxane	100%	5:0:1	78%
2	$MeNO_2$	100%	4:0:1	74%
3	DMC	100%	9:0:1	91% (78%)

[a]All the reactions were performed using **1** (0.75 mmol) and **2** (0.50 mmol) with $PdCl_2$ (2.5 mol %) and CuBr (50 mol %), Li_2CO_3 (3.0 equiv.) in **Solvent** (2.0 mL) at 130 ˚C for 14 h under N_2 in a 25 mL sealed Schlenk tube. [b]Conversion and yield of **3** were measured by GC using dodecane as internal standard, isolated yields in parenthesis.

Table S7. Optimization of temperature for the cyclization reaction [a]

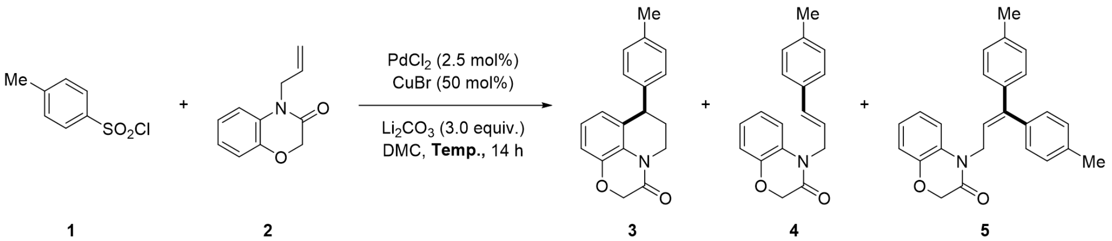

Entry	Temp. (°C)	Conversion [b]	**3:4:5**	Yield of **3** [b]
1	100	-	-	n.r.
2	110	100%	5:0:1	78%
3	120	100%	6:0:1	80%
4	130	100%	9:0:1	91% (78%)
5	140	100%	9:0:1	81%

[a]All the reactions were performed using **1** (0.75 mmol) and **2** (0.50 mmol) with $PdCl_2$ (2.5 mol %) and CuBr (50 mol %), Li_2CO_3 (3.0 equiv.) in DMC (2.0 mL) at **Temp (°C)** for 14 h under N_2 in a 25 mL sealed Schlenk tube. [b]Conversion and yield of **3** were measured by GC using dodecane as internal standard, isolated yields in parenthesis.

Unsuccessful Examples：

Our systematic substrate screening identified that specific substrates failed to form cyclization products under the tested conditions, as illustrated in the figure.

***Unreactive sulfonyl chlorides*：**

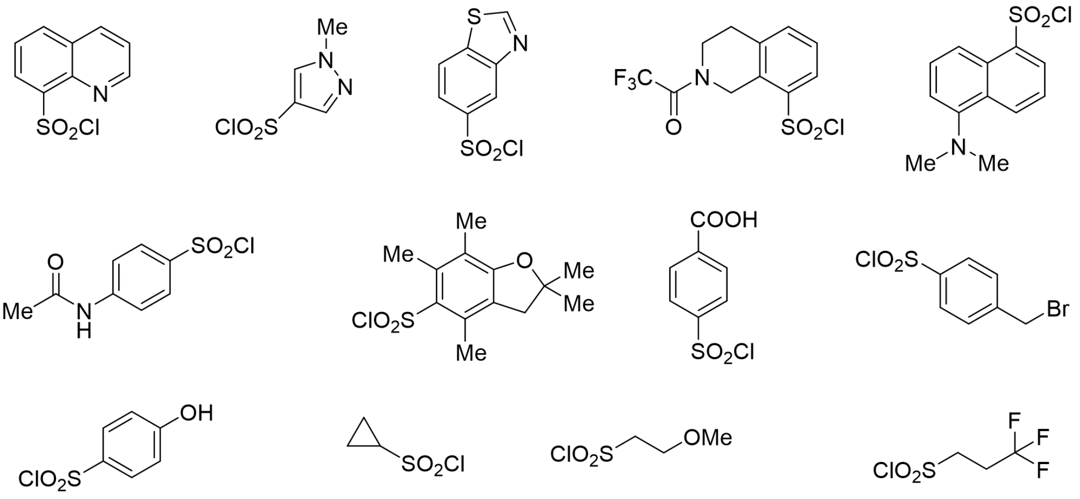

Unreactive allyl substrates:

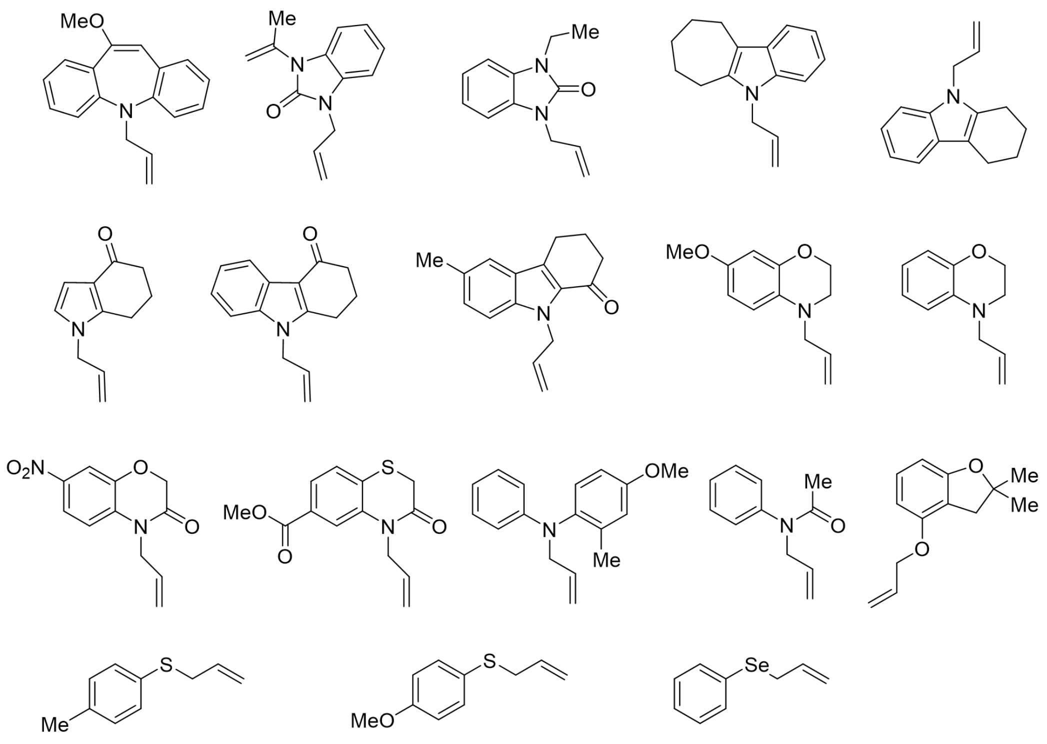

Experimental procedures

Preparation of starting materials

Conditions A:

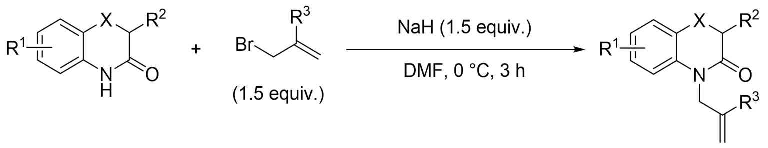

Scheme S1. Synthesis of *N*-allyl substrates

To a suspension of NaH (1.5 equiv.) in dry DMF (5 mL) at 0 °C under N_2 was added quinolinone (5.0 mmol). The mixture was stirred at this temperature for 30 minutes, and then allyl bromide (1.5 equiv.) was added (**Scheme S1**). The mixture was allowed to warm to room temperature. After completion indicated by TLC, the mixture was diluted with water (15 mL) and extracted with EtOAc (10 mL x 3). The combined organic layers were washed with water and brine. The organic layer was dried over anhydrous Na_2SO_4, filtered, and concentrated under reduced pressure. The residue was purified via column chromatography (petroleum ether and ethyl acetate mixture as eluting solvent) to afford the desired products (**2, S45-S67**).

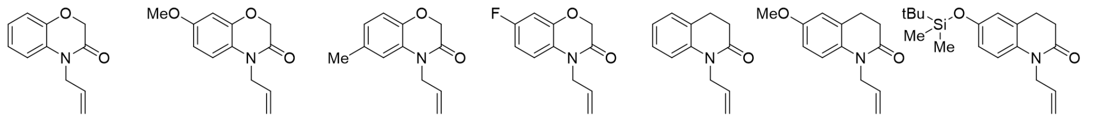

2 **S45** **S46** **S47** **S48** **S49** **S50**

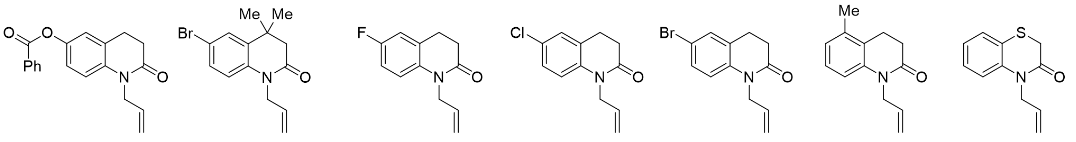

S51 **S52** **S53** **S54** **S55** **S56** **S57**

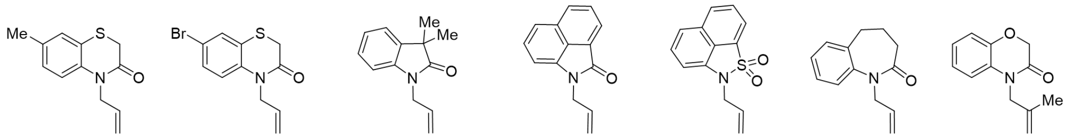

S58 **S59** **S60** **S61** **S62** **S63** **S64**

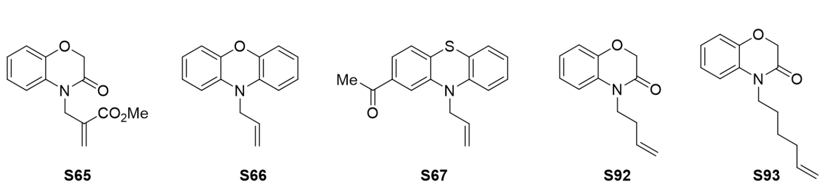

S65 **S66** **S67** **S92** **S93**

Conditions B:

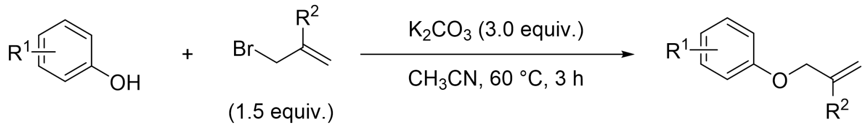

Scheme S2. Synthesis of *O*-allyl substrates

To a suspension of K_2CO_3 (3.0 equiv.) in dry CH_3CN (5 mL) at 60 °C under N_2 was added phenol (5.0 mmol). The mixture was stirred at this temperature for 30 minutes, and then allyl bromide (1.5 equiv.) was added (**Scheme S2**). After completion indicated by TLC, the mixture was diluted with water (15 mL) and extracted with EtOAc (10 mL x 3). The combined organic layers were washed with water and brine. The organic layer was dried over anhydrous Na_2SO_4, filtered, and concentrated under reduced pressure. The residue was purified via column chromatography (petroleum ether and ethyl acetate mixture as eluting solvent) to afford the desired products (**S68-S90**).

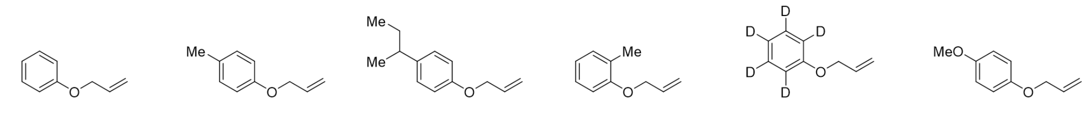

S68 **S69** **S70** **S71** **S72** **S73**

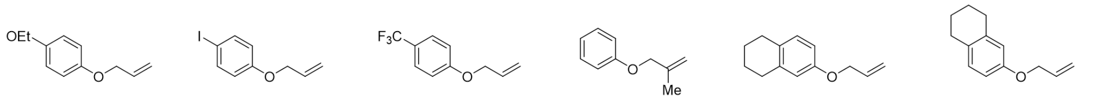

S74 **S75** **S76** **S77** **S78** **S79**

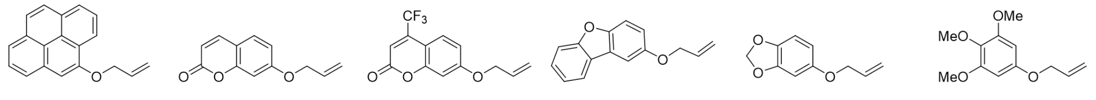

S80 **S81** **S82** **S83** **S84** **S85**

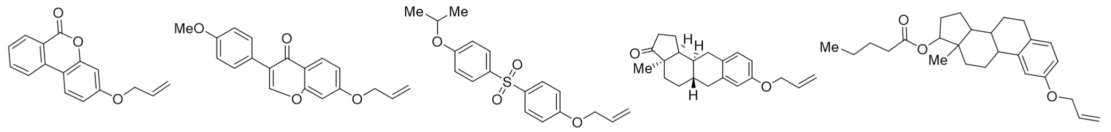

S86 **S87** **S89** **S90** **S91**

General protocol for synthesis of benzo-fused heterocycles

Conditions C:

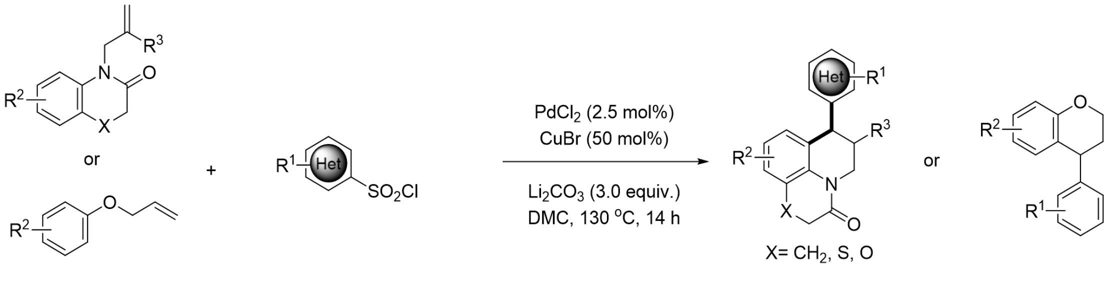

1a 2a 3a

Scheme S3. General protocol for synthesis of benzo-fused heterocycles

To an oven-dried 25 ml Schlenk tube, $PdCl_2$ (2.2 mg, 0.0125 mmol), Li_2CO_3 (111.0 mg, 1.50 mmol), CuBr (35.9 mg, 0.25 mmol), Ar-SO_2Cl (0.75 mmol.) and 1a (0.50 mmol, 1.0 equiv.) were added sequentially. The reaction mixture was then rinsed with anhydrous dimethylcarbonate (DMC, 2.0 mL) under a N_2 atmosphere, in which anhydrous dimethyl carbonate (2.0 mL) was added. The reaction tube was then sealed under a N_2 atmosphere and stirred in a preheated 130 °C oil bath for 14 h (**Scheme S3**). Upon cooling, the mixture was filtered through a short silica gel column for GC and GC-MS. Then the combined filtrate was concentrated under vacuo and purified by silica gel column chromatography using Ethyl acetate/Petroleum ether as eluent to afford the desired products.

Gram-scale preparation of benzo-fused heterocycles

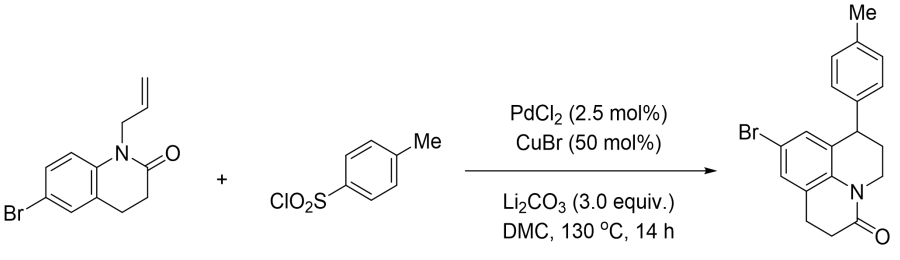

S12 **1** **54** (78%)

Scheme S4. Gram-scale preparation of **54**

To an oven-dried 200 ml PTFE reactor, $PdCl_2$ (33.5 mg, 0.189 mmol), Li_2CO_3 (1.7 g, 22.5 mmol), CuBr (537.9 mg, 3.8 mmol), **1** (2.2 g, 11.3 mmol.) and **S12** (2.0 g, 7.5 mmol.) were added sequentially. The reaction device was then added with anhydrous DMC (60.0 mL) in an N_2 atmosphere. Above-mention reaction mixture in the PTFE reactor was heated at

130 °C for 14 hours with vigorous stirring (**Scheme S4**). Upon cooling, the mixture was filtered through a short silica gel column for GC and GC-MS analysis. Then the combined filtrate was concentrated under vacuo and purified by silica gel column chromatography using Ethyl acetate/pentane as eluent to afford the desired product **54** (2.1 g) in 78% yield.

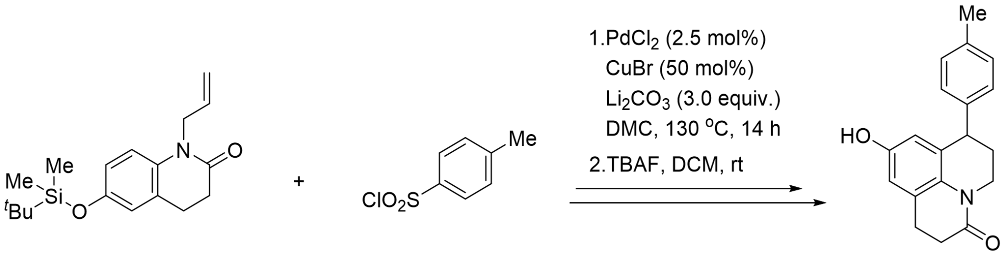

Scheme S5. Gram-scale preparation of **93**

To an oven-dried 200 ml PTFE reactor, $PdCl_2$ (27.9 mg, 0.158 mmol), Li_2CO_3 (1.4 g, 18.9 mmol), CuBr (451.9 mg, 3.2 mmol), **1** (1.8 g, 9.5 mmol.) and **S7** (2.0 g, 6.3 mmol.) were added sequentially. The reaction device was then added with anhydrous DMC (60.0 mL) in an N_2 atmosphere. Above-mention reaction mixture in the PTFE reactor was heated at 130 °C for 14 hours with vigorous stirring (**Scheme S5**). Upon cooling, the mixture was filtered through a short silica gel column for GC and GC-MS analysis. Then the combined filtrate was concentrated under vacuo and purified by silica gel column chromatography using Ethyl acetate/pentane as eluent to afford the desired product **50** (1.7 g) in 67% yield. **50** was then added to the DCM, followed by TBAF, and stirred at room temperature to get compound **93** in 74% yield

Synthetic applications

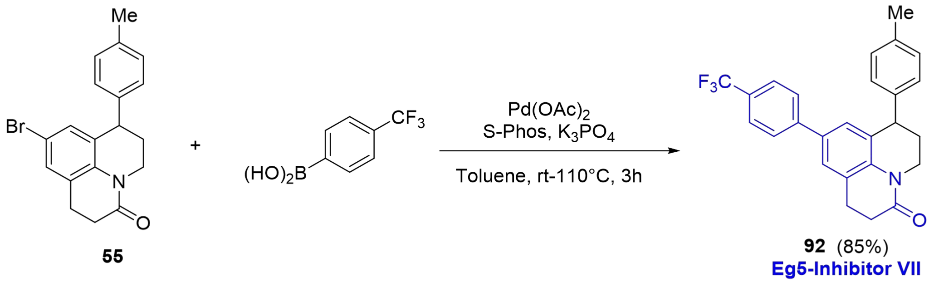

Scheme S6. Synthesis of Eg5-Inhibitor VII analog **92**

To an oven-dried 25 ml Schlenk tube with nitrogen, $Pd(OAc)_2$ (5.0 mg, 0.024 mmol), S-Phos (0.210 g, 0.05 mmol), (4-(trifluoromethyl)phenyl)boronic acid (0.6 mmol), powdered anhydrous K_3PO_4 (0.170 g, 0.8 mmol) and **55** (0.4 mmol). Add dry Toluene (3 mL) to the mixture degassed by nitrogen for 30 minutes. Stir the mixture for a few minutes at room temperature. Heat the mixture to 110 °C (**Scheme S6**). Monitor the reaction by TLC or GC-MS analysis until the disappearance of **55**. Cool the reaction mixture to room temperature. Then the combined filtrate was concentrated under vacuo and purified by silica gel column chromatography using Ethyl acetate/Petroleum ether as eluent to afford the desired product of **92** (143.2 mg, 85%).

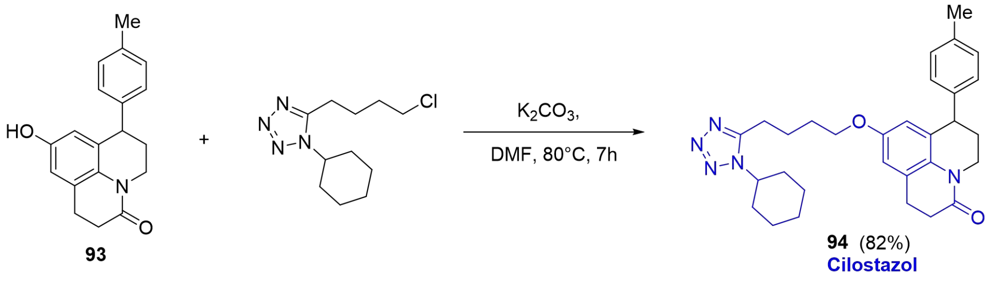

Scheme S7. Synthesis of Cilostazol analog **94**

To an oven-dried 25 ml Schlenk tube with nitrogen, K_2CO_3 (66.5 mg, 0.90 mmol), 5-(4-chlorobutyl)-1-cyclohexyl-1H-tetrazole (87.2 mg, 0.36 mmol) and **93** (0.30 mmol). Add dry DMF (3 mL) to the mixture for 110 °C (**Scheme S7**). Monitor the reaction by TLC or GC-MS analysis until the disappearance of **93**. Cool the reaction mixture to room temperature. Then the combined filtrate was concentrated under vacuo and purified by silica gel column chromatography using Ethyl acetate/Petroleum ether as eluent to afford the desired product of **94** (122.8 mg, 82%).

Mechanistic experiments

Real time reaction monitoring

Six sets of parallel experiments were conducted under standard conditions. The reactions were terminated at 30 minutes, 1 hour, 2 hours, 4 hours, 8 hours, and 14 hours, respectively. It was analyzed by GC-MS as shown below (**Figure S1**). When the reaction is 30 min, it can be observed that the reaction has not started. When the reaction takes one hour, the feedstock starts to be partially converted into compound **3** and compound **4**. As the reaction time increases, compound **5** starts to be produced, but compound **3** gradually becomes the main product.

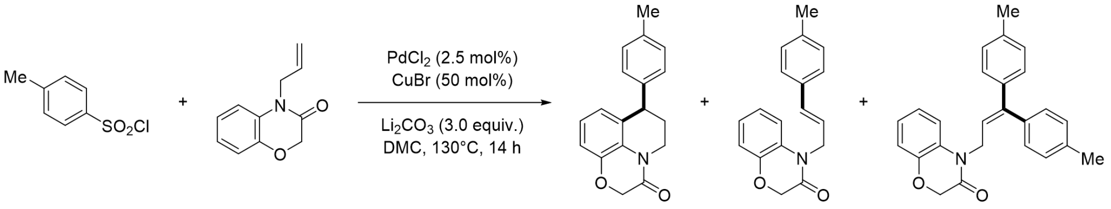

1 **2** **3** **4** **5**

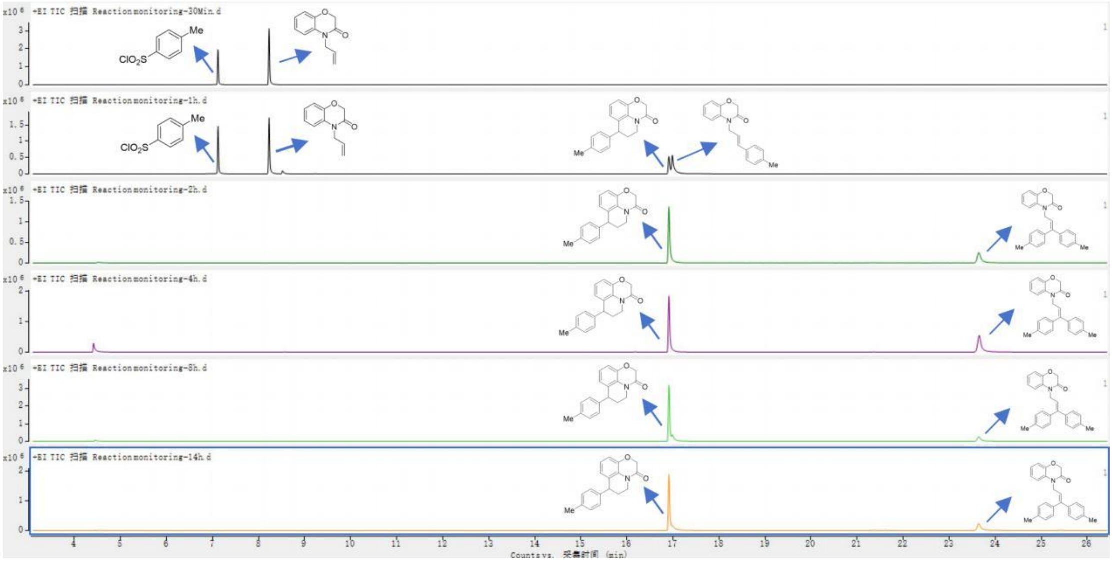

Figure S1. Real time reaction monitoring.

Controlling experiments

To gain more insight into the relationship of the three observed products (**3**, **4**, and **5**), several controlling experiments were designed (**Scheme S8**). First, compound **4** was submitted under our standard conditions in the absence of arylsulfonyl chloride **1**, while there was no transformation and **4** was recovered. Similarly, there was no reaction using compound

5 under standard conditions. When compound **4** and arylsulfonyl chloride **1** were submitted under standard conditions, compound **5** was isolated in 85%.

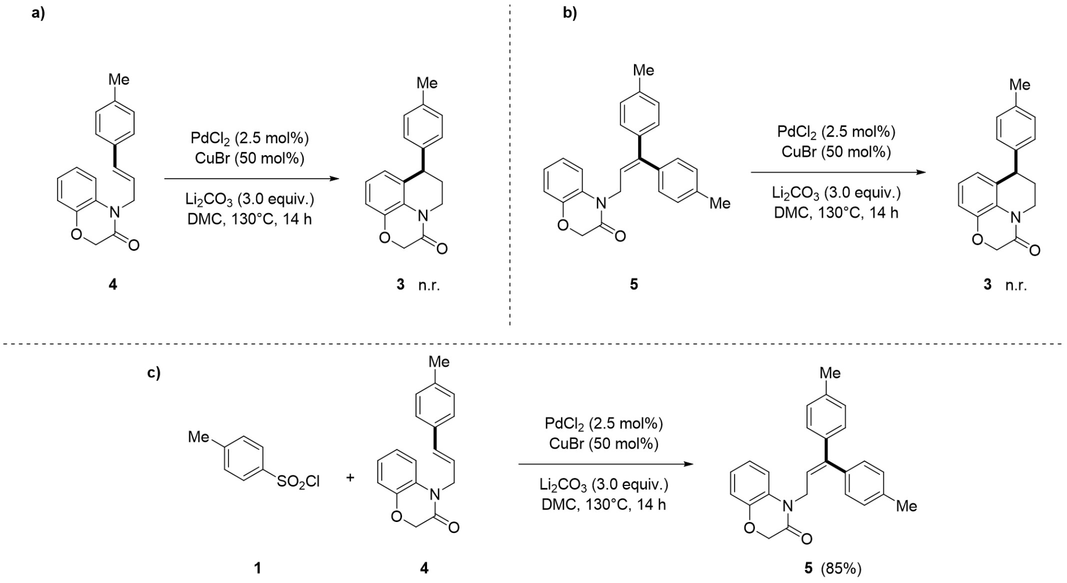

Scheme S8. Controlling experiments of transformations between compound 3, 4, 5

To an oven-dried 25 ml Schlenk tube, $PdCl_2$ (1.0 equiv.), Li_2CO_3 (3.0 equiv.), CuBr (50 mol%), **2** (0.2 mmol, 1.0 equiv.), 4-bromobenzenesulfonyl chloride (1.5 equiv.) and 4-(3-phenylallyl)-2H-benzo[b][1,4]oxazin-3(4H)-one (1.0 equiv.) were added subsequently, followed by addition of anhydrous DMC. The reaction tube was then sealed under a N_2 atmosphere and stirred in a preheated 130 °C oil bath for 14 h. Upon cooling, the mixture was filtered through a short silica gel column for GC-MS analysis (**Figure S2**). We observed the production of compound **6**. These results are indicative of the fact that the single heck product is converted into the cyclized product. So in the initial reaction, compound **4** would be converted into compound **3**. These results are consistent with the experiments of the real time reaction monitoring.

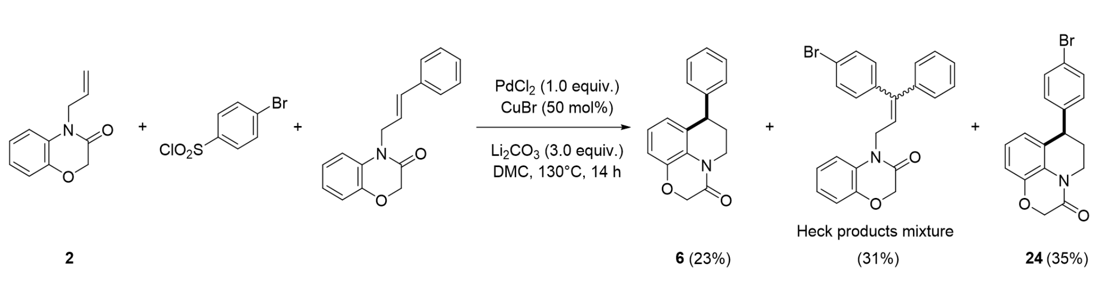

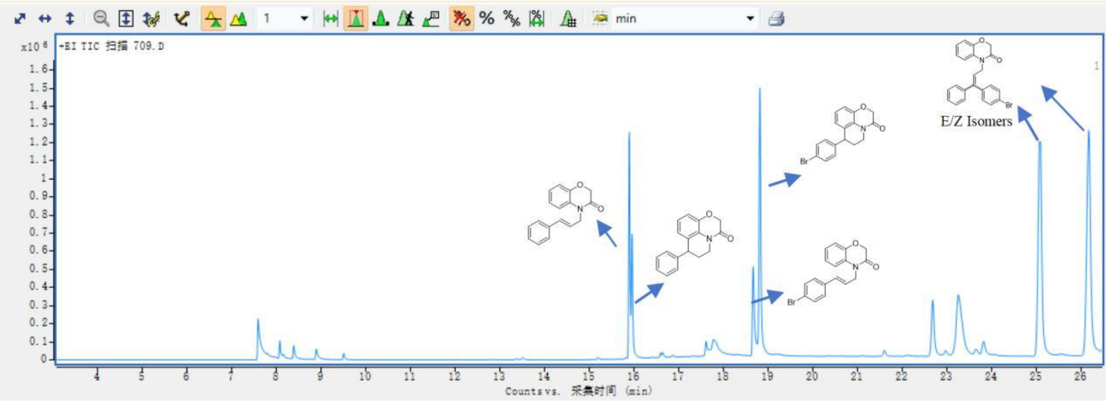

Figure S2. Controlling experiments

Radical scavenger experiments

To an oven-dried 25 ml Schlenk tube, $PdCl_2$ (2.5 mol%), Li_2CO_3 (3.0 equiv.), CuBr (0.5 equiv.), *p*-toluenesulfonyl chloride (**1**, 1.5 equiv.) and **2** were added subsequently. The reaction tube was then charged with radical scavengers (BHT), followed by addition of anhydrous DMC. The reaction tube was then sealed under a N_2 atmosphere and stirred in a preheated 130 °C oil bath for 14 h (**Scheme S9**). Upon cooling, the mixture was filtered through a short silica gel column for GC (0.1 mmol 1,3,5-Trimethoxybenzene was used as internal standard) and GC-MS analysis.

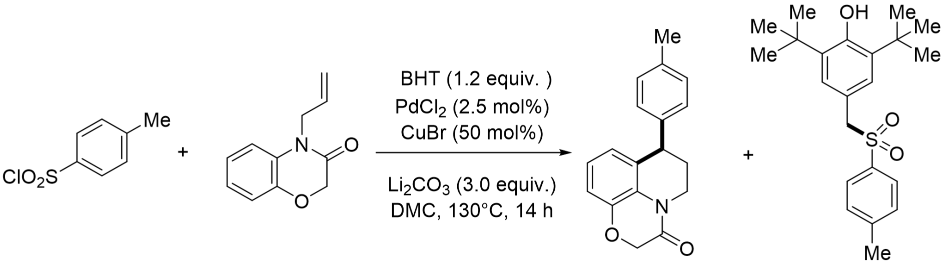

1 **2** **3** (52%) **100** (45%)

Scheme S9. Radical scavenger experiments

Synthesis of deuterated substrates and deuterium scrambling experiments

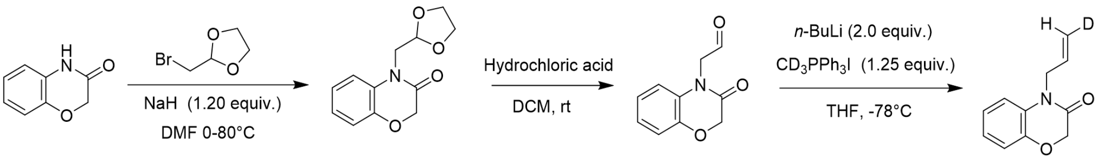

Y1 **Y2** **Y3** **Y4** (33%)

Scheme S10. Synthesis of deuterated compound **Y4**

To a flask charged with **Y1** (5.00 g, 33.56 mmol) and *N*,*N*-Dimethylformamide (60 ml) was added Sodium hydride (0.97 g, 40.27 mmol) and stirred in an ice-water bath for 30 min. followed by 2-(bromomethyl)-1,3-dioxolane (8.41 g, 50.34 mmol). The reaction mixture was stirred at 80 °C for 6 h. The resulting solution was filtered and the filtrate was concentrated. The crude product was purified by flash column chromatography (eluted with PE/EA) to afford **Y2** as a colorless oil (6.86 g, 87%). Then **Y2** was dissolved in Dichloromethane and added hydrochloric acid slowly. The reaction mixture was stirred at room temperature overnight. The crude product was purified by flash column chromatography (eluted with PE/EA) to afford **Y3** as a white solid (4.91 g, 88%).

To a suspension of CD_3PPh_3I (2.54 g, 6.25 mmol) in THF (15 mL) at -78 °C was added *n*-BuLi (1.6 M solution in hexane, 6.25 mL, 10.00 mmol), and the resulting solution was stirred for 30 min at -78 °C. A solution of **Y3** (0.96 g, 5.00 mmol) in THF (5 mL) was added dropwise at -78 °C, and the resulting mixture was stirred for 4-6 hours at -78 °C (**Scheme S10**). Saturated aqueous NH_4Cl was added to quench the reaction, and the mixture was extracted with ether. The combined extract was washed with water and brine, dried over $MgSO_4$, and concentrated. The crude product was purified by flash column chromatography (eluted with PE/EA) to afford **Y4** as a pale yellow oil (0.31 g, 33%).

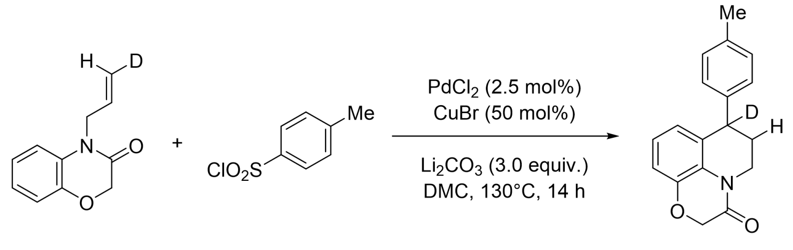

Y4 **1** **103** (67%)

Scheme S11. Synthesis of deuterated product **103**

To an oven-dried 25 ml Schlenk tube, $PdCl_2$ (2.2 mg, 0.0125 mmol), Li_2CO_3 (111.0 mg, 1.50 mmol), CuBr (354.6 mg, 2.50 mmol), **1** (0.75 mmol.) and **Y4** (0.50 mmol, 1.0 equiv.) were added sequentially. The reaction mixture was then rinsed with anhydrous dimethylcarbonate (DMC, 2.0 mL) under a N_2 atmosphere, in which anhydrous dimethyl carbonate (2.0 mL) was added. The reaction tube was then sealed under a N_2 atmosphere and stirred in a preheated 130 °C oil bath for 14 h (**Scheme S11**). Upon cooling, the mixture was

filtered through a short silica gel column for GC and GC-MS. Then the combined filtrate was concentrated under vacuo and purified by silica gel column chromatography using Ethyl acetate/Petroleum ether as eluent to afford the desired products of **103** (93.8mg, 67%).

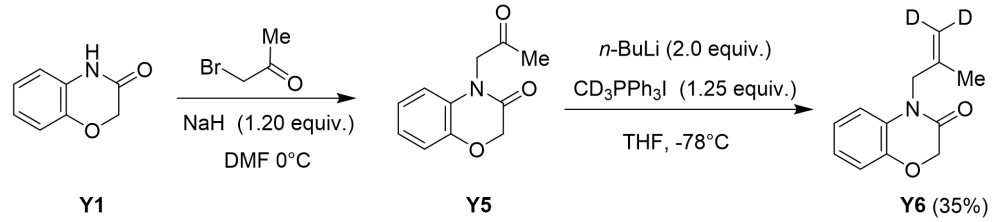

Scheme S12. Synthesis of deuterated compound **Y6**

To a flask charged with **Y1** (5.00 g, 33.56 mmol) and *N*,*N*-Dimethylformamide (60 ml) was added Sodium hydride (0.97 g, 40.27 mmol) and stirred in an ice-water bath for 30 min. followed by 1-bromopropan-2-one (6.84 g, 50.34 mmol). The reaction mixture was stirred for 6 h.The resulting solution was filtered and the filtrate was concentrated. The crude product was purified by flash column chromatography (eluted with PE/EA) to afford **Y5** as a colorless oil (5.30 g, 77%).

To a suspension of CD_3PPh_3I (2.54 g, 6.25 mmol) in THF (15 mL) at -78 °C was added *n*-BuLi (1.6 M solution in hexane, 6.25 mL, 10.00 mmol), and the resulting solution was stirred for 30 min at -78 °C. A solution of **Y5** (1.03 g, 5.00 mmol) in THF (5 mL) was added dropwise at -78 °C, and the resulting mixture was stirred for 4-6 hours at -78 °C (**Scheme S12**). Saturated aqueous NH_4Cl was added to quench the reaction, and the mixture was extracted with ether. The combined extract was washed with water and brine, dried over $MgSO_4$, and concentrated. The crude product was purified by flash column chromatography (eluted with PE/EA) to afford **Y6** as a pale yellow oil (0.36 g, 35%).

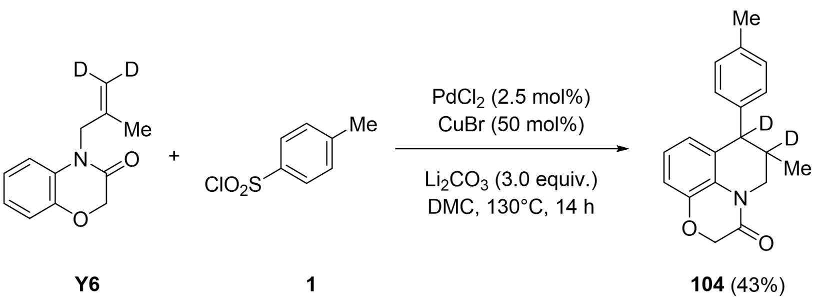

Scheme S13. Synthesis of deuterated product **104**

To an oven-dried 25 ml Schlenk tube, $PdCl_2$ (2.2 mg, 0.0125 mmol), Li_2CO_3 (111.0 mg,

1.50 mmol), CuBr (354.6 mg, 2.50 mmol), **1** (0.75 mmol) and **Y6** (0.50 mmol, 1.0 equiv.) were added sequentially. The reaction mixture was then rinsed with anhydrous dimethylcarbonate (DMC, 2.0 mL) under a N_2 atmosphere, in which anhydrous dimethyl carbonate (2.0 mL) was added. The reaction tube was then sealed under a N_2 atmosphere and stirred in a preheated 130 °C oil bath for 14 h (**Scheme S13**). Upon cooling, the mixture was filtered through a short silica gel column for GC and GC-MS. Then the combined filtrate was concentrated under vacuo and purified by silica gel column chromatography using Ethyl acetate/Petroleum ether as eluent to afford the desired products of **104** (64.9 mg, 43%).

Characterization data

4-allyl-2H-benzo[b][1,4]oxazin-3(4H)-one (2)

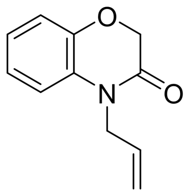

The *N*-allyl substrate **2** was prepared according to the general procedure **A** from 2H-benzo[b][1,4]oxazin-3(4H)-one and 3-bromoprop-1-ene, and purified with chromatography on silica gel (petroleum ether) to afford the title compound in 89% yield as a white solid, 0.84 g. M.P. 62.1-63.3 °C.

^{1}H NMR (400 MHz, $CDCl_3$) δ 6.99 (d, J = 2.1 Hz, 4H), 5.88 (ddt, J = 17.1, 10.2, 5.0 Hz, 1H), 5.28–5.17 (m, 2H), 4.64 (s, 2H), 4.56 (dt, J = 4.9, 1.8 Hz, 2H). ^{13}C NMR (101 MHz, $CDCl_3$) δ 164.4, 145.3, 131.7, 128.9, 124.0, 122.8, 117.4, 117.1, 115.6, 67.7, 43.8.

HRMS (ESI, m/z): calculated for $C_{11}H_{11}NO_2$ $[M+H]^+$ = 190.0863, found 190.0865.

4-allyl-7-methoxy-2H-benzo[b][1,4]oxazin-3(4H)-one (S45)

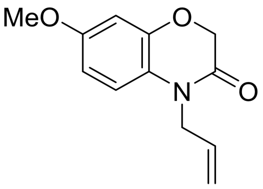

The *N*-allyl substrate **S45** was prepared according to the general procedure **A** from 7-methoxy-2H-benzo[b][1,4]oxazin-3(4H)-one and 3-bromoprop-1-ene, and purified with chromatography on silica gel (petroleum ether) to afford the title compound in 83% yield as a white solid, 0.91 g. M.P. 56.1-57.3 °C.

^{1}H NMR (400 MHz, $CDCl_3$) δ 6.87 (d, J = 8.8 Hz, 1H), 6.59–6.51 (m, 2H), 5.85 (ddt, J = 17.2, 10.5, 5.0 Hz, 1H), 5.25–5.16 (m, 2H), 4.61 (s, 2H), 4.51 (d, J = 4.9 Hz, 2H), 3.75 (s, 3H). ^{13}C NMR (101 MHz, $CDCl_3$) δ 163.7, 156.4, 146.2, 131.8, 122.3, 117.2, 116.1, 108.0, 103.2, 67.8, 55.7, 43.9.

HRMS (ESI, m/z): calculated for $C_{12}H_{13}NO_3$ $[M+H]^+$ = 220.0968, found 220.0970.

4-allyl-6-methyl-2H-benzo[b][1,4]oxazin-3(4H)-one (S46)

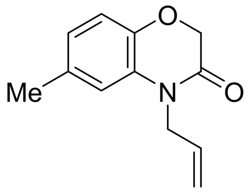

The *N*-allyl substrate **S46** was prepared according to the general procedure **A** from 6-methyl-2H-benzo[b][1,4]oxazin-3(4H)-one and 3-bromoprop-1-ene, and purified with chromatography on silica gel (petroleum ether) to afford the title compound in 81% yield as a white solid, 0.82 g. M.P. 70.2-71.4 °C.

^{1}H NMR (400 MHz, $CDCl_3$) δ 6.87 (d, J = 7.9 Hz, 1H), 6.78 (d, J = 8.2 Hz, 2H), 5.95–5.79 (m, 1H), 5.26–5.17 (m, 2H), 4.59 (s, 2H), 4.55–4.51 (m, 2H), 2.30 (s, 3H). ^{13}C NMR (101 MHz, $CDCl_3$) δ 164.5, 143.1, 132.4, 131.6, 128.5, 124.3, 117.1, 116.7, 116.1, 67.7, 43.7, 21.1.

HRMS (ESI, m/z): calculated for $C_{12}H_{13}NO_2$ $[M+H]^+$ = 204.1019, found 204.1014.

4-allyl-7-fluoro-2H-benzo[b][1,4]oxazin-3(4H)-one (S47)

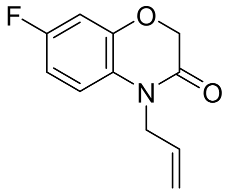

The *N*-allyl substrate **S47** was prepared according to the general procedure **A** from 7-fluoro-2H-benzo[b][1,4]oxazin-3(4H)-one and 3-bromoprop-1-ene, and purified with chromatography on silica gel (petroleum ether) to afford the title compound in 79% yield as a yellow oil, 0.82 g.

^{1}H NMR (400 MHz, $CDCl_3$) δ 6.90 (dd, J = 8.7, 5.2 Hz, 1H), 6.71 (ddt, J = 11.5, 8.6, 4.1 Hz, 2H), 5.93–5.77 (m, 1H), 5.28–5.14 (m, 2H), 4.63 (s, 2H), 4.53 (d, J = 5.0 Hz, 2H). ^{19}F NMR (376 MHz, $CDCl_3$) δ -117.77. ^{13}C NMR (101 MHz, $CDCl_3$) δ 163.6, 158.9, 146.1, 131.5, 125.2, 117.5, 116.1, 109.2, 105.1, 67.7, 44.0.

HRMS (ESI, m/z): calculated for $C_{11}H_{10}FNO_2$ $[M+H]^+$ = 208.0769, found 208.0772.

1-allyl-3,4-dihydroquinolin-2(1H)-one (S48)

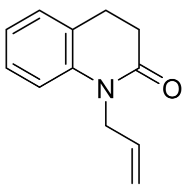

The *N*-allyl substrate **S48** was prepared according to the general procedure **A** from 3,4-dihydroquinolin-2(1H)-one and 3-bromoprop-1-ene, and purified with chromatography on silica gel (petroleum ether) to afford the title compound in 86% yield as a colorless oil, 0.80 g.

^{1}H NMR (400 MHz, $CDCl_3$) δ 7.22–7.11 (m, 2H), 6.98 (dd, J = 6.9, 5.6 Hz, 2H), 5.93–5.82 (m, 1H), 5.21–5.09 (m, 2H), 4.54 (d, J = 2.7 Hz, 2H), 2.90 (t, J = 7.3 Hz, 2H), 2.66 (t, J = 7.0 Hz, 2H). ^{13}C NMR (101 MHz, $CDCl_3$) δ 170.0, 139.8, 132.6, 127.7, 127.3, 126.2, 122.8, 116.2, 115.3, 45.0, 31.7, 25.5.

HRMS (ESI, m/z): calculated for $C_{12}H_{13}NO$ $[M+H]^+$ = 188.1070, found 188.1078.

1-allyl-6-methoxy-3,4-dihydroquinolin-2(1H)-one (S49)

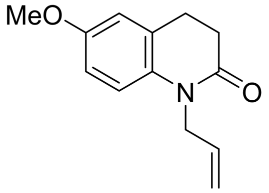

The *N*-allyl substrate **S49** was prepared according to the general procedure **A** from 6-methoxy-3,4-dihydroquinolin-2(1H)-one and 3-bromoprop-1-ene, and purified with chromatography on silica gel (petroleum ether) to afford the title compound in 81% yield as a white solid, 0.87 g. M.P. 53.5-54.1 °C.

^{1}H NMR (400 MHz, $CDCl_3$) δ 6.92–6.87 (m, 1H), 6.75–6.70 (m, 2H), 5.87 (ddt, J = 17.2, 10.5, 4.8 Hz, 1H), 5.21–5.09 (m, 2H), 4.55–4.49 (m, 2H), 3.77 (s, 3H), 2.91–2.84 (m, 2H), 2.65 (dd, J = 8.5, 6.2 Hz, 2H). ^{13}C NMR (101 MHz, $CDCl_3$) δ 169.7, 155.3, 133.5, 132.8, 127.9, 116.4, 116.3, 113.9, 111.9, 55.6, 45.2, 31.8, 25.9.

HRMS (ESI, m/z): calculated for $C_{13}H_{15}NO_2$ $[M+H]^+$ = 218.1176, found 218.1177.

1-allyl-6-((tert-butyldimethylsilyl)oxy)-3,4-dihydroquinolin-2(1H)-one (S50)

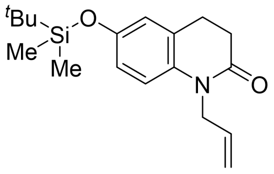

The *N*-allyl substrate **S50** was prepared according to the general procedure **A** from 6-((tert-butyldimethylsilyl)oxy)-3,4-dihydroquinolin-2(1H)-one and 3-bromoprop-1-ene, and purified with chromatography on silica gel (petroleum ether) to afford the title compound in 83% yield as a white solid, 1.32 g. M.P. 97.1-97.8 °C.

^{1}H NMR (400 MHz, $CDCl_3$) δ 6.87–6.80 (m, 1H), 6.66 (d, *J* = 9.5 Hz, 2H), 5.96–5.82 (m, 1H), 5.23–5.08 (m, 2H), 4.51 (dt, *J* = 4.0, 2.1 Hz, 2H), 2.85 (t, *J* = 7.5 Hz, 2H), 2.66 (dt, *J* = 8.8, 4.3 Hz, 2H), 0.98 (d, *J* = 2.6 Hz, 9H), 0.19 (d, *J* = 1.9 Hz, 6H). ^{13}C NMR (126 MHz, $CDCl_3$) δ 169.9, 151.3, 134.0, 133.0, 127.8, 119.6, 118.3, 116.4, 116.3, 45.4, 31.9, 31.9, 25.8, 25.8, 25.8, 18.3, -4.3, -4.3.

HRMS (ESI, m/z): calculated for $C_{18}H_{27}NO_2Si$ $[M+H]^+$ = 318.1884, found 318.1886.

1-allyl-2-oxo-1,2,3,4-tetrahydroquinolin-6-yl benzoate (S51)

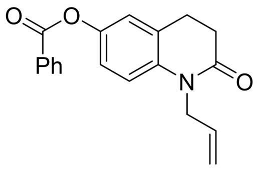

The *N*-allyl substrate **S51** was prepared according to the general procedure **A** from 2-oxo-1,2,3,4-tetrahydroquinolin-6-yl benzoate and 3-bromoprop-1-ene, and purified with chromatography on silica gel (petroleum ether) to afford the title compound in 78% yield as a white solid, 1.19 g. M.P. 81.7-82.9 °C.

^{1}H NMR (400 MHz, $CDCl_3$) δ 8.19 (d, *J* = 7.7 Hz, 2H), 7.65 (t, *J* = 7.4 Hz, 1H), 7.52 (t, *J* = 7.7 Hz, 2H), 7.10–6.98 (m, 3H), 5.96–5.82 (m, 1H), 5.19 (dd, *J* = 20.0, 14.2 Hz, 2H), 4.57 (d, *J* = 3.2 Hz, 2H), 2.95 (t, *J* = 7.4 Hz, 2H), 2.72 (t, *J* = 7.4 Hz, 2H). ^{13}C NMR (101 MHz, $CDCl_3$) δ 170.0, 165.6, 146.2, 137.9, 133.9, 132.5, 130.3, 130.3, 129.5, 128.7, 128.7, 127.8, 121.3, 120.4, 116.6, 116.3, 45.4, 31.6, 25.7.

HRMS (ESI, m/z): calculated for $C_{19}H_{17}NO_3$ $[M+H]^+$ = 308.1281, found 308.1280.

1-allyl-6-bromo-4,4-dimethyl-3,4-dihydroquinolin-2(1H)-one (S52)

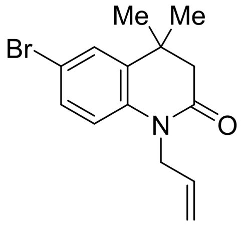

The *N*-allyl substrate **S52** was prepared according to the general procedure **A** from 6-bromo-4,4-dimethyl-3,4-dihydroquinolin-2(1H)-one and 3-bromoprop-1-ene, and purified with chromatography on silica gel (petroleum ether) to afford the title compound in 84% yield as a white solid, 1.23 g. M.P. 66.4-67.9 °C.

^{1}H NMR (400 MHz, $CDCl_3$) δ 7.38 (d, *J* = 2.3 Hz, 1H), 7.31 (dd, *J* = 8.7, 2.4 Hz, 1H), 6.86 (d, *J* = 8.7 Hz, 1H), 5.90–5.79 (m, 1H), 5.23–5.08 (m, 2H), 4.59–4.53 (m, 2H), 2.53 (s, 2H), 1.29 (s, 6H). ^{13}C NMR (101 MHz, $CDCl_3$) δ 169.0, 137.7, 137.1, 132.3, 130.2, 127.6, 117.6, 116.9, 116.4, 45.6, 45.0, 33.4, 27.3, 27.3.

HRMS (ESI, m/z): calculated for $C_{14}H_{16}BrNO$ $[M+H]^+$ = 294.0488, found 294.0489.

1-allyl-6-fluoro-3,4-dihydroquinolin-2(1H)-one (S53)

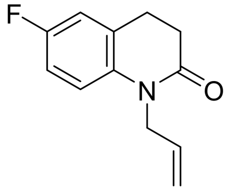

The *N*-allyl substrate **S53** was prepared according to the general procedure **A** from 6-fluoro-3,4-dihydroquinolin-2(1H)-one and 3-bromoprop-1-ene, and purified with chromatography on silica gel (petroleum ether) to afford the title compound in 82% yield as a colorless oil, 0.80 g.

^{1}H NMR (400 MHz, $CDCl_3$) δ 6.93–6.82 (m, 3H), 5.91–5.77 (m, 1H), 5.20–5.06 (m, 2H), 4.50 (d, *J* = 4.8 Hz, 2H), 2.92–2.83 (m, 2H), 2.68–2.60 (m, 2H). ^{19}F NMR (376 MHz, $CDCl_3$) δ -120.57. ^{13}C NMR (101 MHz, $CDCl_3$) δ 169.6, 159.6, 157.2, 136.1, 132.5, 128.3, 116.4, 114.7, 1136, 45.3, 31.4, 25.5.

HRMS (ESI, m/z): calculated for $C_{12}H_{12}FNO$ $[M+H]^+$ = 206.0976, found 206.0973.

1-allyl-6-chloro-3,4-dihydroquinolin-2(1H)-one (S54)

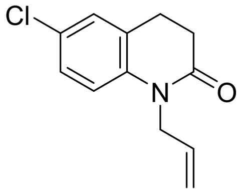

The *N*-allyl substrate **S54** was prepared according to the general procedure **A** from 6-chloro-3,4-dihydroquinolin-2(1H)-one and 3-bromoprop-1-ene, and purified with chromatography on silica gel (petroleum ether) to afford the title compound in 78% yield as a white solid, 0.86 g. M.P. 62.1-63.4 °C.

^{1}H NMR (400 MHz, $CDCl_3$) δ 7.16 (d, *J* = 8.8 Hz, 2H), 6.90 (d, *J* = 8.4 Hz, 1H), 5.92–5.80 (m, 1H), 5.24–5.07 (m, 2H), 4.53 (dd, *J* = 3.3, 1.6 Hz, 2H), 2.90 (t, *J* = 7.4 Hz, 2H), 2.68 (t, *J* = 7.3 Hz, 2H). ^{13}C NMR (101 MHz, $CDCl_3$) δ 169.8, 138.6, 132.4, 128.2, 128.1, 127.9, 127.3, 116.7, 116.7, 45.2, 31.5, 25.5.

HRMS (ESI, m/z): calculated for $C_{12}H_{12}ClNO$ $[M+H]^+$ = 222.0680, found 222.0682.

1-allyl-6-bromo-3,4-dihydroquinolin-2(1H)-one (S55)

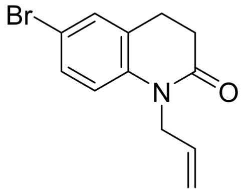

The *N*-allyl substrate **S55** was prepared according to the general procedure **A** from 6-bromo-3,4-dihydroquinolin-2(1H)-one and 3-bromoprop-1-ene, and purified with chromatography on silica gel (petroleum ether) to afford the title compound in 80% yield as a white solid, 1.06 g. M.P. 72.8-73.9 °C.

^{1}H NMR (400 MHz, $CDCl_3$) δ 7.31 (d, *J* = 8.6 Hz, 2H), 6.85 (d, *J* = 8.4 Hz, 1H), 5.86 (ddt, *J* = 17.2, 10.5, 4.8 Hz, 1H), 5.23–5.08 (m, 2H), 4.53 (d, *J* = 4.8 Hz, 2H), 2.93–2.88 (m, 2H), 2.71–2.65 (m, 2H). ^{13}C NMR (101 MHz, $CDCl_3$) δ 169.7, 139.2, 132.4, 130.7, 130.3, 128.6, 117.1, 116.7, 115.7, 45.2, 31.6, 25.4.

HRMS (ESI, m/z): calculated for $C_{12}H_{12}BrNO$ $[M+H]^+$ = 266.0175, found 266.0177.

1-allyl-5-methyl-3,4-dihydroquinolin-2(1H)-one (S56)

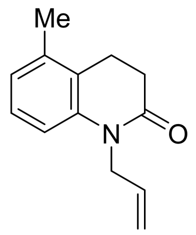

The *N*-allyl substrate **S56** was prepared according to the general procedure **A** from 5-methyl-3,4-dihydroquinolin-2(1H)-one and 3-bromoprop-1-ene, and purified with chromatography on silica gel (petroleum ether) to afford the title compound in 82% yield as a white solid, 0.82 g. M.P. 56.9-58.2 °C.

^{1}H NMR (400 MHz, $CDCl_3$) δ 7.06 (t, *J* = 7.9 Hz, 1H), 6.84 (t, *J* = 7.0 Hz, 2H), 5.98–5.72 (m, 1H), 5.21–4.93 (m, 2H), 4.65–4.28 (m, 2H), 2.91 (s, 1H), 2.84 (s, 1H), 2.62 (t, *J* = 7.3 Hz, 2H), 2.26 (s, 3H). ^{13}C NMR (101 MHz, $CDCl_3$) δ 169.8, 162.4, 139.9, 135.3, 132.7, 126.7, 124.8 (d, *J* = 40.0 Hz), 116.0, 113.4, 45.2, 31.3 (d, *J* = 36.0 Hz), 21.5, 19.5.

HRMS (ESI, m/z): calculated for $C_{13}H_{15}NO$ $[M+H]^+$ = 202.1126, found 202.1223.

4-allyl-2H-benzo[b][1,4]thiazin-3(4H)-one (S57)

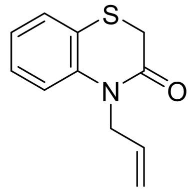

The *N*-allyl substrate **S57** was prepared according to the general procedure **A** from 2H-benzo[b][1,4]thiazin-3(4H)-one and 3-bromoprop-1-ene, and purified with chromatography on silica gel (petroleum ether) to afford the title compound in 75% yield as a yellow oil, 0.77 g.

^{1}H NMR (400 MHz, $CDCl_3$) δ 7.34 (dd, *J* = 7.6, 1.3 Hz, 1H), 7.23–7.17 (m, 1H), 7.12 (dd, *J* = 8.3, 1.3 Hz, 1H), 7.00 (td, *J* = 7.4, 1.0 Hz, 1H), 5.91 (ddt, *J* = 17.2, 10.5, 4.7 Hz, 1H), 5.24–5.12 (m, 2H), 4.61–4.55 (m, 2H), 3.41 (s, 2H). ^{13}C NMR (101 MHz, $CDCl_3$) δ 165.0, 139.8, 132.5, 128.3, 127.2, 123.6, 118.0, 116.5, 47.8, 31.5, 26.9.

HRMS (ESI, m/z): calculated for $C_{11}H_{11}NOS$ $[M+H]^+$ = 206.0634, found 206.0635.

4-allyl-7-methyl-2H-benzo[b][1,4]thiazin-3(4H)-one (S58)

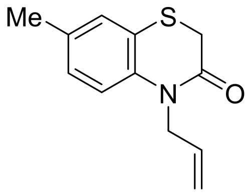

The *N*-allyl substrate **S58** was prepared according to the general procedure **A** from 7-methyl-2H-benzo[b][1,4]thiazin-3(4H)-one and 3-bromoprop-1-ene, and purified with chromatography on silica gel (petroleum ether) to afford the title compound in 79% yield as a white solid, 0.87 g. M.P. 62,3-63.9 °C.

^{1}H NMR (400 MHz, $CDCl_3$) δ 7.16 (s, 1H), 7.00 (s, 2H), 5.90 (ddt, *J* = 17.2, 10.4, 4.7 Hz, 1H), 5.24–5.11 (m, 2H), 4.56 (dt, *J* = 4.7, 1.9 Hz, 2H), 3.41 (s, 2H), 2.28 (s, 3H). ^{13}C NMR (101 MHz, $CDCl_3$) δ 165.0, 137.5, 133.5, 132.7, 128.7, 128.0, 123.4, 117.9, 116.5, 47.7, 31.7, 20.6.

HRMS (ESI, m/z): calculated for $C_{12}H_{13}NOS$ $[M+H]^+$ = 220.0791, found 220.0791.

4-allyl-7-bromo-2H-benzo[b][1,4]thiazin-3(4H)-one (S59)

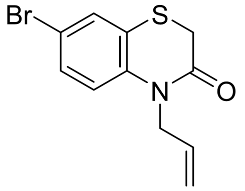

The *N*-allyl substrate **S59** was prepared according to the general procedure **A** from 7-bromo-2H-benzo[b][1,4]thiazin-3(4H)-one and 3-bromoprop-1-ene, and purified with chromatography on silica gel (petroleum ether) to afford the title compound in 82% yield as a white solid, 1.16 g. M.P. 57.8-59.2 °C.

^{1}H NMR (400 MHz, $CDCl_3$) δ 7.48 (d, *J* = 2.2 Hz, 1H), 7.30 (dt, *J* = 9.0, 2.1 Hz, 1H), 6.98 (dd, *J* = 8.8, 1.7 Hz, 1H), 5.94–5.82 (m, 1H), 5.25–5.10 (m, 2H), 4.56 (t, *J* = 2.4 Hz, 2H), 3.42 (t, *J* = 2.3 Hz, 2H). ^{13}C NMR (101 MHz, $CDCl_3$) δ 164.6, 138.9, 132.2, 130.8, 130.2, 125.8, 119.4, 116.8, 116.3, 47.8, 31.3.

HRMS (ESI, m/z): calculated for $C_{11}H_{10}BrNOS$ $[M+H]^+$ = 283.9739, found 283.9742.

1-allyl-3,3-dimethylindolin-2-one (S60)

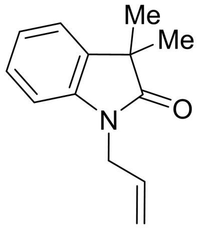

The *N*-allyl substrate **S60** was prepared according to the general procedure **A** from 3,3-dimethylindolin-2-one and 3-bromoprop-1-ene, and purified with chromatography on silica gel (petroleum ether) to afford the title compound in 75% yield as a white solid, 0.75 g. M.P. 71.6-73.3 °C.

^{1}H NMR (400 MHz, $CDCl_3$) δ 7.23–7.15 (m, 2H), 7.02 (t, *J* = 7.5 Hz, 1H), 6.82 (d, *J* = 7.9 Hz, 1H), 5.82 (ddt, *J* = 15.8, 10.3, 5.1 Hz, 1H), 5.21–5.11 (m, 2H), 4.32 (dt, *J* = 5.4, 1.7 Hz, 2H), 1.37 (s, 6H). ^{13}C NMR (126 MHz, $CDCl_3$) δ 180.9, 141.7, 135.7, 131.5, 127.5, 122.4, 122.2, 117.1, 108.8, 44.0, 42.0, 24.4, 24.4.

HRMS (ESI, m/z): calculated for $C_{13}H_{15}NO$ $[M+H]^+$ = 202.1226, found 202.1224.

1-allylbenzo[cd]indol-2(1H)-one (S61)

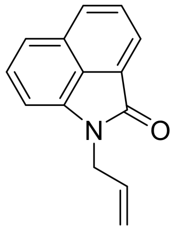

The *N*-allyl substrate **S61** was prepared according to the general procedure **A** from benzo[cd]indol-2(1H)-one and 3-bromoprop-1-ene, and purified with chromatography on silica gel (petroleum ether) to afford the title compound in 76% yield as a yellow solid, 0.79 g. M.P. 72.7-73.5 °C.

^{1}H NMR (400 MHz, $CDCl_3$) δ 8.04 (dd, *J* = 25.7, 7.5 Hz, 2H), 7.70 (dd, *J* = 8.2, 7.0 Hz, 1H), 7.52 (d, *J* = 8.3 Hz, 1H), 7.43 (dd, *J* = 8.4, 7.1 Hz, 1H), 6.89 (d, *J* = 7.0 Hz, 1H), 5.96 (ddt, *J* = 17.2, 10.5, 5.4 Hz, 1H), 5.30–5.20 (m, 2H), 4.55 (dt, *J* = 5.4, 1.5 Hz, 2H). ^{13}C NMR (101 MHz, $CDCl_3$) δ 167.8, 139.3, 132.7, 131.0, 129.2, 128.7, 128.6, 126.5, 125.3, 124.4, 120.4, 117.3, 105.8, 42.7.

HRMS (ESI, m/z): calculated for $C_{14}H_{11}NO$ $[M+H]^+$ = 210.0914, found 210.0910.

2-allyl-2H-naphtho[1,8-cd]isothiazole 1,1-dioxide (S62)

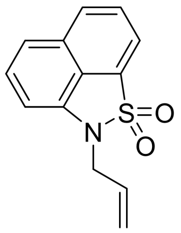

The *N*-allyl substrate **S62** was prepared according to the general procedure **A** from 2H-naphtho[1,8-cd]isothiazole 1,1-dioxide and 3-bromoprop-1-ene, and purified with chromatography on silica gel (petroleum ether) to afford the title compound in 77% yield as a yellow solid, 0.94 g. M.P. 82.1-82.9 °C.

^{1}H NMR (400 MHz, $CDCl_3$) δ 8.07 (dd, *J* = 8.3, 3.1 Hz, 1H), 7.97 (dd, *J* = 7.3, 3.0 Hz, 1H), 7.75 (td, *J* = 8.3, 3.5 Hz, 1H), 7.56–7.43 (m, 2H), 6.73 (dd, *J* = 7.3, 2.9 Hz, 1H), 6.09–5.97 (m, 1H), 5.54–5.45 (m, 1H), 5.35 (ddt, *J* = 10.3, 2.7, 1.3 Hz, 1H), 4.48–4.43 (m, 2H). ^{13}C NMR (101 MHz, $CDCl_3$) δ 136.3, 131.7, 131.3, 130.7, 130.3, 129.4, 128.1, 120.0, 119.4, 119.1, 118.4, 103.9, 44.2.

HRMS (ESI, m/z): calculated for $C_{13}H_{11}NO_2S$ $[M+H]^+$ = 246.0583, found 246.0585.

1-allyl-1,3,4,5-tetrahydro-2H-benzo[b]azepin-2-one (S63)

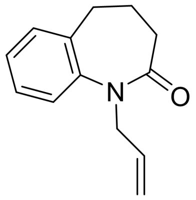

The *N*-allyl substrate **S63** was prepared according to the general procedure **A** from 1,3,4,5-tetrahydro-2H-benzo[b]azepin-2-one and 3-bromoprop-1-ene, and purified with chromatography on silica gel (petroleum ether) to afford the title compound in 79% yield as a yellow oil, 0.79 g.

^{1}H NMR (400 MHz, $CDCl_3$) δ 7.27– 7.14 (m, 4H), 5.89 (ddt, *J* = 17.1, 10.2, 5.9 Hz, 1H), 5.18–5.09 (m, 2H), 4.42 (d, *J* = 6.0 Hz, 2H), 2.70 (d, *J* = 7.1 Hz, 2H), 2.33–2.09 (m, 4H). ^{13}C NMR (101 MHz, $CDCl_3$) δ 172.9, 142.7, 135.7, 133.7, 129.3, 127.5, 126.3, 122.6, 117.3, 50.8, 33.3, 30.2, 28.9.

HRMS (ESI, m/z): calculated for $C_{13}H_{15}NO$ $[M+H]^+$ = 202.1227, found 202.1225.

4-(2-methylallyl)-2H-benzo[b][1,4]oxazin-3(4H)-one (S64)

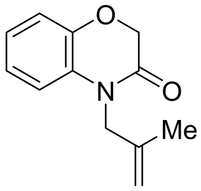

The *N*-allyl substrate **S64** was prepared according to the general procedure **A** from 2H-benzo[b][1,4]oxazin-3(4H)-one and 3-bromo-2-methylprop-1-ene, and purified with chromatography on silica gel (petroleum ether) to afford the title compound in 82% yield as a colorless oil, 0.83 g.

^{1}H NMR (400 MHz, $CDCl_3$) δ 7.02–6.94 (m, 3H), 6.96–6.90 (m, 1H), 4.95–4.90 (m, 1H), 4.80–4.75 (m, 1H), 4.65 (d, *J* = 2.1 Hz, 2H), 4.46 (s, 2H), 1.80–1.75 (m, 3H). ^{13}C NMR (101 MHz, $CDCl_3$) δ 164.4, 145.3, 139.0, 128.9, 124.0, 122.8, 116.9, 115.8, 111.5, 67.7, 46.9, 20.0.

HRMS (ESI, m/z): calculated for $C_{12}H_{13}NO_2$ $[M+H]^+$ = 204.1019, found 204.1022.

Methyl 2-((3-oxo-2,3-dihydro-4H-benzo[b][1,4]oxazin-4-yl)methyl)acrylate (S65)

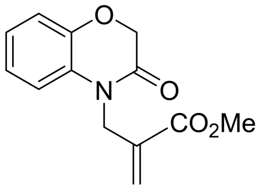

The *N*-allyl substrate **S65** was prepared according to the general procedure **A** from 2*H*-benzo[b][1,4]oxazin-3(4H)-one and methyl 2-(bromomethyl)acrylate, and purified with chromatography on silica gel (petroleum ether) to afford the title compound in 82% yield as a white solid, 1.01 g. M.P. 104.7-105.3 °C.

^{1}H NMR (400 MHz, $CDCl_3$) δ 7.03–6.92 (m, 3H), 6.83–6.80 (m, 1H), 6.29 (t, *J* = 1.7 Hz, 1H), 5.45 (t, *J* = 1.8 Hz, 1H), 4.79 (d, *J* = 1.8 Hz, 2H), 4.66 (d, *J* = 1.6 Hz, 2H), 3.82 (d, *J* = 1.6 Hz, 3H). ^{13}C NMR (101 MHz, $CDCl_3$) δ 166.0, 164.5, 145.3, 133.6, 128.3, 125.3, 124.3, 123.0, 117.2, 115.5, 67.7, 52.3, 41.8.

HRMS (ESI, m/z): calculated for $C_{13}H_{13}NO_4$ $[M+H]^+$ = 248.0917, found 248.0914.

10-allyl-10H-phenoxazine--methane (1/1) (S66)

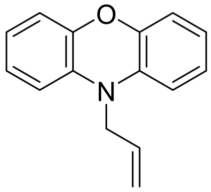

The *N*-allyl substrate **S66** was prepared according to the general procedure **A** from 10*H*-phenoxazine and 3-bromoprop-1-ene, and purified with chromatography on silica gel (petroleum ether) to afford the title compound in 78% yield as a white solid, 0.87 g. M.P. 68.2-68.8 °C.

^{1}H NMR (400 MHz, $CDCl_3$) δ 6.85–6.73 (m, 2H), 6.72–6.63 (m, 4H), 6.45 (d, *J* = 7.9 Hz, 2H), 5.88 (ddt, *J* = 17.2, 10.5, 3.9 Hz, 1H), 5.36–5.20 (m, 2H), 4.16 (dt, *J* = 4.1, 2.1 Hz, 2H). ^{13}C NMR (101 MHz, $CDCl_3$) δ 145.3, 145.3, 133.8, 131.1, 131.1, 123.8, 123.8, 121.2, 121.2, 117.0, 115.3, 115.3, 112.2, 112.2, 47.8.

HRMS (ESI, m/z): calculated for $C_{15}H_{13}NO$ $[M+H]^+$ = 224.1070, found 224.1071.

1-(10-allyl-10H-phenothiazin-2-yl)ethan-1-one (S67)

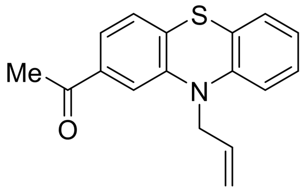

The *N*-allyl substrate **S67** was prepared according to the general procedure **A** from 1-(10H-phenothiazin-2-yl)ethan-1-one and 3-bromoprop-1-ene, and purified with chromatography on silica gel (petroleum ether) to afford the title compound in 77% yield as a white solid, 1.08 g. M.P. 89.6-90.7 °C.

^{1}H NMR (400 MHz, $CDCl_3$) δ 7.42 (dd, *J* = 7.9, 1.7 Hz, 1H), 7.37 (d, *J* = 1.8 Hz, 1H), 7.13–7.06 (m, 2H), 7.03 (dd, *J* = 7.7, 1.6 Hz, 1H), 6.89 (td, *J* = 7.5, 1.2 Hz, 1H), 6.83 (dd, *J* = 8.2, 1.3 Hz, 1H), 6.05–5.95 (m, 1H), 5.38–5.25 (m, 2H), 4.50 (dt, *J* = 4.2, 2.0 Hz, 2H), 2.52 (d, *J* = 2.3 Hz, 3H). ^{13}C NMR (101 MHz, $CDCl_3$) δ 197.5, 144.6, 143.9, 136.3, 132.5, 130.3, 127.8, 126.9, 126.5, 123.1, 122.9, 121.8, 118.0, 115.7, 114.1, 51.4, 26.6.

HRMS (ESI, m/z): calculated for $C_{17}H_{15}NOS$ $[M+H]^+$ = 282.3803, found 282.3807

(allyloxy)benzene[1] (S68)

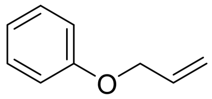

The *O*-allyl substrate **S68** was prepared according to the general procedure **B** from phenol and 3-bromoprop-1-ene, and purified with chromatography on silica gel (petroleum ether) to afford the title compound in 93% yield as a colorless oil.

^{1}H NMR (400 MHz, $CDCl_3$): δ 4.47 (d, J = 5.5 Hz, 2H), 5.23 (d, J = 12.4 Hz, 1H), 5.36 (d, J = 17.4 Hz, 1H), 5.95-6.05 (m, 1H), 6.85-6.92 (m, 3H), 7.20-7.26 (m, 2H).

1-(allyloxy)-4-methylbenzene[2] (S69)

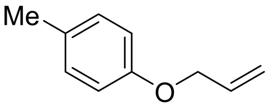

The *O*-allyl substrate **S69** was prepared according to the general procedure **B** from *p*-cresol and 3-bromoprop-1-ene, and purified with chromatography on silica gel (petroleum ether) to afford the title compound in 92% yield as a colorless oil.

^{1}H NMR (400 MHz, $CDCl_3$) δ 7.07 (d, J = 8.5 Hz, 2H), 6.81 (d, J = 8.4 Hz, 2H), 6.05 (ddd, J = 16.5,10.5, 5.3 Hz,1H), 5.39 (dd, J = 17.3,1.4 Hz,1H), 5.26 (dd, J = 10.5,1.2 Hz,1H), 4.50 (d, J = 5.3 Hz, 2H), 2.28 (s, 3H).

1-(allyloxy)-4-(sec-butyl)benzene[3] (S70)

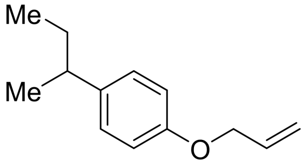

The *O*-allyl substrate **S70** was prepared according to the general procedure **B** from 4-(sec-butyl)phenol and 3-bromoprop-1-ene, and purified with chromatography on silica gel (petroleum ether) to afford the title compound in 93% yield as a colorless oil.

^{1}H NMR (400 MHz, $CDCl_3$) δ 7.18–7.10 (m, 2H), 6.95–6.86 (m, 2H), 6.11 (ddt, J = 15.9, 10.5, 5.2 Hz, 1H), 5.46 (dt, J = 17.2, 2.0 Hz, 1H), 5.36–5.28 (m, 1H), 4.56 (dd, J = 5.4, 2.4 Hz,

2H), 2.60 (h, J = 7.1 Hz, 1H), 1.60 (q, J = 7.3 Hz, 2H), 1.26 (d, J = 6.9 Hz, 3H), 0.87 (t, J = 7.3 Hz, 3H).

1-(allyloxy)-2-methylbenzene[4] (S71)

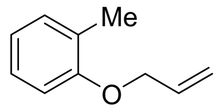

The *O*-allyl substrate **S71** was prepared according to the general procedure **B** from *o*-cresol and 3-bromoprop-1-ene, and purified with chromatography on silica gel (petroleum ether) to afford the title compound in 92% yield as a colorless oil.

^{1}H NMR (400 MHz, $CDCl_3$) δ 7.19–7.08 (m, 2H), 6.86 (t, J = 7.4 Hz, 1H), 6.81 (d, J = 8.4 Hz, 1H), 6.07 (ddt, J = 17.3, 10.2, 5.0 Hz, 1H), 5.43 (dq, J = 17.2, 1.7 Hz, 1H), 5.27 (dq, J = 10.6, 1.6 Hz, 1H), 4.54 (dt, J = 5.1, 1.7 Hz, 2H), 2.25 (s, 3H).

1-(allyloxy)benzene-2,3,4,5,6-*d*5[5] (S72)

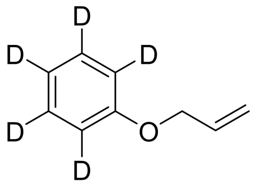

The *O*-allyl substrate **S72** was prepared according to the general procedure **B** from phen-d5-ol and 3-bromoprop-1-ene, and purified with chromatography on silica gel (petroleum ether) to afford the title compound in 87% yield as a colorless oil.

^{1}H NMR (400 MHz, $CDCl_3$) δ 6.11 (ddt, J = 15.8, 10.4, 5.2 Hz, 1H), 5.46 (d, J = 17.3 Hz, 1H), 5.33 (d, J = 10.6 Hz, 1H), 4.58 (d, J = 5.3 Hz, 2H).

1-(allyloxy)-4-methoxybenzene[6] (S73)

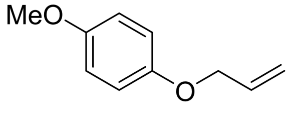

The *O*-allyl substrate **S73** was prepared according to the general procedure **B** from 4-methoxyphenol and 3-bromoprop-1-ene, and purified with chromatography on silica gel (petroleum ether) to afford the title compound in 91% yield as a colorless oil.

^{1}H NMR (300 MHz, $CDCl_3$): δ 3.75 (s, 3H), 4.47 (dt, 2H, J = 5.3, 1.5 Hz), 5.26 (dq, 1H, J =

10.5, 1.4 Hz), 5.40 (dq, 1H, J = 17.3, 1.4 Hz), 6.04 (ddt, 1H, J = 17.3, 10.5, 1.4 Hz), 6.78-6.89 (m, 4H).

1-(allyloxy)-4-ethoxybenzene[7] (S74)

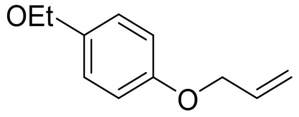

The *O*-allyl substrate **S74** was prepared according to the general procedure **B** from 4-ethoxyphenol and 3-bromoprop-1-ene, and purified with chromatography on silica gel (petroleum ether) to afford the title compound in 89% yield as a colorless oil.

^{1}H NMR (600 MHz, $CDCl_3$) δ: 1.39 (t, J = 7.0 Hz, 3H), 3.98 (q, J = 7.0 Hz, 2H), 4.48 (dt, J = 5.3, 1.5 Hz, 2H), 5.27 (dq, J = 10.5, 1.4 Hz, 1H), 5.40 (dq, J = 17.3, 1.6 Hz, 1H), 6.05 (ddt J = 17.2, 10.6, 5.3 Hz, 1H,), 6.78–6.89 (m, 4H)

1-(allyloxy)-4-iodobenzene[8] (S75)

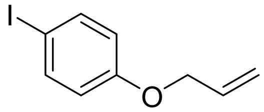

The *O*-allyl substrate **S75** was prepared according to the general procedure **B** from 4-iodophenol and 3-bromoprop-1-ene, and purified with chromatography on silica gel (petroleum ether) to afford the title compound in 87% yield as a colorless oil.

^{1}H NMR (600 MHz, CDCl3) δ 7.55 (d, J = 6.8 Hz, 2H), 6.69 (d, J = 6.8 Hz, 2H), 6.02 (dt, J = 11.4, 5.8 Hz, 1H), 5.34 (dd, J = 64.0, 14.0 Hz, 2H), 4.50 (s, 2H).

1-(allyloxy)-4-(trifluoromethyl)benzene[9] (S76)

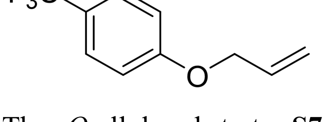

The *O*-allyl substrate **S76** was prepared according to the general procedure **B** from 4-(trifluoromethyl)phenol and 3-bromoprop-1-ene, and purified with chromatography on silica gel (petroleum ether) to afford the title compound in 84% yield as a colorless oil.

^{1}H NMR (300 MHz, $CDCl_3$): δ 7.54 (m, 2H), 6.98 (m, 2H), 6.05 (ddt, J = 17.3, 10.5, 5.3 Hz, 1H), 5.43 (dq, J = 17.3 Hz, J = 1.6 Hz, 1H), 5.32 (dq, J = 10.5 Hz, 1H), 4.59 (dt, J = 5.2 Hz,

2H).

(but-3-en-2-yloxy)benzene[10] **(S77)**

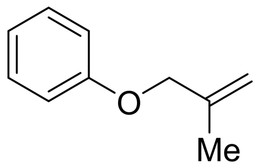

The *O*-allyl substrate **S77** was prepared according to the general procedure **B** from phenol and 3-bromobut-1-ene, and purified with chromatography on silica gel (petroleum ether) to afford the title compound in 87% yield as a colorless oil.

^{1}H NMR (500 MHz, $CDCl_3$) δ 7.51–6.8 (m, 5H), 5.28 (d, *J* = 17.3, 1H), 5.17 (d, *J* = 10.6, 1H), 4.91–4.73 (m, 1H), 4.63–4.40 (d, 1H), 1.44 (d, *J* = 6.4, 3H).

6-(allyloxy)-1,2,3,4-tetrahydronaphthalene[11] **(S78)**

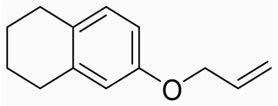

The *O*-allyl substrate **S78** was prepared according to the general procedure **B** from 5,6,7,8-tetrahydronaphthalen-2-ol and 3-bromoprop-1-ene, and purified with chromatography on silica gel (petroleum ether) to afford the title compound in 85% yield as a colorless oil.

^{1}H NMR (400 MHz, $CDCl_3$) δ 7.00 (d, *J* = 8.3 Hz, 1H), 6.73 (d, *J* = 8.4 Hz, 1H), 6.67 (s, 1H), 6.09 (m, 1H), 5.44 (d, *J* = 17.2 Hz, 1H), 5.31 (d, *J* = 10.5 Hz, 1H), 4.54 (d, *J* = 4.6 Hz, 2H), 2.76 (d, *J* = 15.6 Hz, 4H), 1.82 (s, 4H).

5-(allyloxy)-1,2,3,4-tetrahydronaphthalene[12] **(S79)**

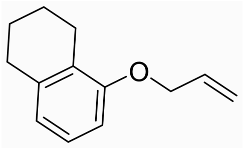

The *O*-allyl substrate **S79** was prepared according to the general procedure **B** from 5,6,7,8-tetrahydronaphthalen-1-ol and 3-bromoprop-1-ene, and purified with chromatography on silica gel (petroleum ether) to afford the title compound in 87% yield as a colorless oil.

^{1}H NMR (400 MHz, $CDCl_3$): δ = 7.08 (t, *J* = 7.9 Hz, 1 H), 6.73 (d, *J* = 7.6 Hz, 1 H), 6.66

(d, J = 8.1 Hz, 1 H), 6.10 (ddt, J = 17.2, 10.1, 5.0 Hz, 1 H), 5.47 (ddd, J = 17.2, 3.2, 1.5 Hz, 1 H), 5.34–5.25 (m, 1 H), 4.56 (dt, J = 4.9, 1.5 Hz, 2 H), 2.76 (dt, J = 20.9, 6.1 Hz, 4 H), 1.84–1.78 (m, 4 H).

2-(allyloxy)pyrene (S80)

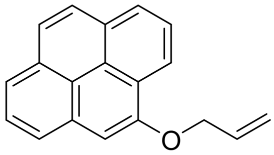

The *O*-allyl substrate **S80** was prepared according to the general procedure **B** from pyren-2-ol and 3-bromoprop-1-ene, and purified with chromatography on silica gel (petroleum ether) to afford the title compound in 83% yield as a white solid. M.P. 42.7-43.6°C.

^{1}H NMR (400 MHz, $CDCl_3$) δ 8.54 (d, J = 9.1 Hz, 1H), 8.13 (t, J = 7.9 Hz, 2H), 8.09 (d, J = 2.3 Hz, 1H), 8.07 (s, 1H), 8.00 (dd, J = 7.6, 1.2 Hz, 1H), 7.97 (d, J = 3.0 Hz, 1H), 7.92 (d, J = 9.0 Hz, 1H), 7.50 (d, J = 8.5 Hz, 1H), 6.27 (ddd, J = 10.6, 5.2, 1.1 Hz, 1H), 5.62 (dt, J = 17.3, 1.5 Hz, 1H), 5.42 (dt, J = 10.5, 1.4 Hz, 1H), 4.88 (dt, J = 5.0, 1.5 Hz, 2H). ^{13}C NMR (101 MHz, $CDCl_3$) δ 152.7, 133.5, 131.8, 131.7, 127.3, 126.5, 126.2, 125.9, 125.5, 125.4, 125.2, 125.0, 124.4, 124.3, 121.4, 120.6, 117.7, 109.5, 69.7.

HRMS (ESI, m/z): calculated for $C_{19}H_{14}O$ $[M+H]^+$ = 259.1117, found 259.1117.

7-(allyloxy)-2H-chromen-2-one[13] (S81)

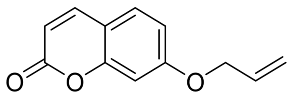

The *O*-allyl substrate **S81** was prepared according to the general procedure **B** from 7-hydroxy-2H-chromen-2-one and 3-bromoprop-1-ene, and purified with chromatography on silica gel (petroleum ether) to afford the title compound in 91% yield as a white oil.

^{1}H NMR (500 MHz, $CDCl_3$) δ 7.63 (d, J = 9.4 Hz, 1H), 7.36 (d, J = 8.5 Hz, 1H), 6.86 (dd, J = 8.5, 2.5 Hz, 1H), 6.83 (d, J = 2.5 Hz, 1H), 6.25 (d, J = 9.5 Hz, 1H), 6.04 (ddt, J = 17.0, 10.4, 5.5 Hz, 1H), 5.44 (dd, J = 17.0, 0.5 Hz, 1H), 5.34 (dd, J = 10.4, 0.5 Hz, 1H), 4.60 (d, J = 5.5 Hz, 2H).

7-(allyloxy)-4-(trifluoromethyl)-2H-chromen-2-one[14] (S82)

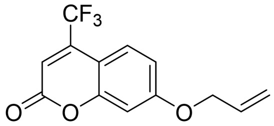

The *O*-allyl substrate **S82** was prepared according to the general procedure **B** from 7-hydroxy-4-(trifluoromethyl)-2H-chromen-2-one and 3-bromoprop-1-ene, and purified with chromatography on silica gel (petroleum ether) to afford the title compound in 92% yield as a colorless oil.

^{1}H NMR (400 MHz, $CDCl_3$) δ7.66 (dd, *J* = 9.3, 1.7Hz, 1H), 7.06–6.77 (m, 2H), 6.66 (s, 1H), 6.14 (m, 1H), 5.54 (dd, *J* = 15.9, 1.5 Hz, 1H), 5.54 (dd, *J* = 10.7, 1.3 Hz, 1H), 4.79 (d, *J* = 2.4 Hz, 2H).

2-(allyloxy)dibenzo[b,d]furan (S83)

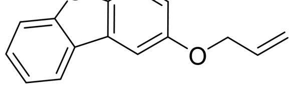

The *O*-allyl substrate **S83** was prepared according to the general procedure **B** from dibenzo[b,d]furan-2-ol and 3-bromoprop-1-ene, and purified with chromatography on silica gel (petroleum ether) to afford the title compound in 86% yield as a colorless oil.

^{1}H NMR (400 MHz, $CDCl_3$) δ 7.93 (d, *J* = 7.6 Hz, 1H), 7.62 (d, *J* = 8.3 Hz, 1H), 7.55 – 7.44 (m, 3H), 7.37 (t, *J* = 7.5 Hz, 1H), 7.12 (dd, *J* = 9.1, 2.6 Hz, 1H), 6.26 – 6.12 (m, 1H), 5.55 (d, *J* = 17.3 Hz, 1H), 5.40 (d, *J* = 10.5 Hz, 1H), 4.63 (d, *J* = 5.2 Hz, 2H). ^{13}C NMR (101 MHz, $CDCl_3$) δ 156.9, 154.8, 150.9, 133.4, 127.1, 124.6, 124.4, 122.4, 120.5, 117.6, 115.7, 112.0, 111.7, 104.9, 69.6.

HRMS (ESI, m/z): calculated for $C_{15}H_{12}O_2$ $[M+H]^+$ = 259.1117, found 259.1117.

5-(allyloxy)benzo[d][1,3]dioxole[15](S84)

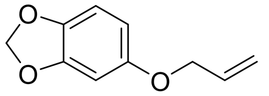

The *O*-allyl substrate **S84** was prepared according to the general procedure **B** from benzo[d][1,3]dioxol-5-ol and 3-bromoprop-1-ene, and purified with chromatography on silica

gel (petroleum ether) to afford the title compound in 88% yield as a colorless oil.

^{1}H NMR (300 MHz, $CDCl_3$) δ 6.70 (d, J = 8.5 Hz, 1H), 6.52 (d, J = 2.5 Hz, 1H), 6.34 (dd, J = 8.5, 2.5 Hz, 1H), 6.03 (ddt, J = 17.2, 10.6, 5.3 Hz, 1H), 5.91 (s, 2H), 5.39 (dq, J = 17.3, 1.6 Hz, 1H), 5.27 (dq, J = 10.5, 1.5 Hz, 1H), 4.46 (dt, J = 5.3, 1.5 Hz, 2H).

5-(allyloxy)-1,2,3-trimethoxybenzene[16](S85)

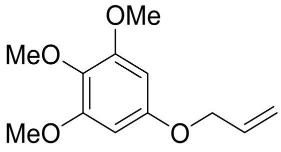

The *O*-allyl substrate **S85** was prepared according to the general procedure **B** from 3,4,5-trimethoxyphenol and 3-bromoprop-1-ene, and purified with chromatography on silica gel (petroleum ether) to afford the title compound in 83% yield as a colorless oil.

^{1}H NMR (400 MHz, $CDCl_3$) δ 6.18 (s, 2H), 6.06 (ddd, J = 22.6, 10.6, 5.4 Hz, 1H), 5.43 (dd, J = 17.3, 1.5 Hz, 1H), 5.33–5.27 (m, 1H), 4.51 (d, J = 5.4 Hz, 2H), 3.84 (s, 6H), 3.79 (s, 3H).

3-(allyloxy)-6H-benzo[c]chromen-6-one[17] (S86)

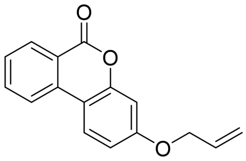

The *O*-allyl substrate **S86** was prepared according to the general procedure **B** from 3-hydroxy-6H-benzo[c]chromen-6-one and 3-bromoprop-1-ene, and purified with chromatography on silica gel (petroleum ether) to afford the title compound in 82% yield as a white solid, 1.03 g. M.P. 92.3-93.2 °C.

^{1}H NMR (400 MHz, $CDCl_3$) δ 8.31 (dd, J = 7.9, 1.3 Hz, 1H), 7.98–7.92 (m, 1H), 7.88 (d, 1H, J = 8.8 Hz, 1H), 7.75 (ddd, J = 8.4, 7.3, 1.5 Hz, 1H), 7.47 (ddd, J = 8.2, 7.3, 1.1 Hz, 1H), 6.90 (dd, J = 8.8, 2.6 Hz, 1H), 6.82 (d, J = 2.6 Hz, 1H), 6.06 (ddt, J = 17.3, 10.5, 5.3 Hz, 1H), 5.45 (dq, J = 17.2, 1.6 Hz, 1H), 5.34 (dq, J = 10.5, 1.4 Hz, 1H), 4.58 (dt J = 5.3, 1.6 Hz, 2H).

7-(allyloxy)-3-(4-methoxyphenyl)-4H-chromen-4-one (S87)

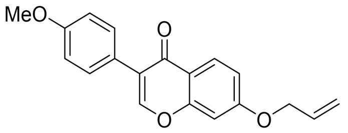

The *O*-allyl substrate **S87** was prepared according to the general procedure **B** from 7-hydroxy-3-(4-methoxyphenyl)-4*H*-chromen-4-one and 3-bromoprop-1-ene, and purified with chromatography on silica gel (petroleum ether) to afford the title compound in 82% yield as a white solid, 1.26 g. M.P. 137.3-138.2 °C.

^{1}H NMR (400 MHz, $CDCl_3$) δ 8.21 (d, *J* = 8.9 Hz, 1H), 7.91 (s, 1H), 7.49 (d, *J* = 8.9 Hz, 2H), 6.98 (ddd, *J* = 11.0, 8.9, 4.5 Hz, 3H), 6.86 (s, 1H), 6.07 (ddt, *J* = 15.9, 10.6, 5.3 Hz, 1H), 5.46 (d, *J* = 17.2 Hz, 1H), 5.36 (d, *J* = 10.5 Hz, 1H), 4.64 (d, *J* = 5.2 Hz, 2H), 3.84 (s, 3H). ^{13}C NMR (101 MHz, $CDCl_3$) δ 176.0, 163.0, 159.7, 158.0, 152.2, 132.2, 130.3, 130.3, 128.0, 125.0, 124.4, 118.7, 118.6, 115.1, 114.1, 114.1, 101.2, 69.4, 55.5.

HRMS (ESI, m/z): calculated for $C_{19}H_{16}O_4$ $[M+H]^+$ = 309.1121, found 309.1127.

1-(allyloxy)-4-((4-isopropoxyphenyl)sulfonyl)benzene[18] (S89)

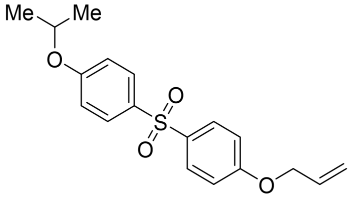

The *O*-allyl substrate **S89** was prepared according to the general procedure **B** from 4-((4-isopropoxyphenyl)sulfonyl)phenol and 3-bromoprop-1-ene, and purified with chromatography on silica gel (petroleum ether) to afford the title compound in 76% yield as a white solid, 1.26 g. M.P. 115.6-116.2 °C.

^{1}H NMR (400 MHz, $CDCl_3$) δ 7.86–7.75 (m, 4H), 7.03–6.85 (m, 4H), 6.09–5.94 (m, 1H), 5.45–5.34 (m, 1H), 5.34–5.24 (m, 1H), 4.61–4.50 (m, 3H), 1.33 (d, *J* = 6.3 Hz, 6H). ^{13}C NMR (101 MHz, $CDCl_3$) δ 162.1, 161.6, 134.2, 134.2, 133.2, 132.1, 132.1, 129.5, 129.5, 118.4, 118.4, 115.6, 115.3, 115.3, 70.6, 69.0, 21.7, 21.7.

(3aR,5aS,11aS,11bS)-8-(allyloxy)-3a-methyl-1,2,3a,4,5,5a,6,11,11a,11b-decahydro-3H-cyclopenta[a]anthracen-3-one (S90)

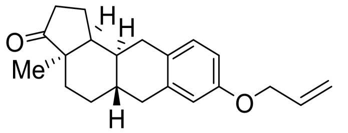

The *O*-allyl substrate **S90** was prepared according to the general procedure **B** from (3aR,5aS,11aS,11bS)-8-hydroxy-3a-methyl-1,2,3a,4,5,5a,6,11,11a,11b-decahydro-3H-cyclopenta[a]anthracen-3-one and 3-bromoprop-1-ene, and purified with chromatography on silica gel (petroleum ether) to afford the title compound in 83% yield as a white solid, 1.29 g. M.P. 104.7-105.8 °C.

^{1}H NMR (400 MHz, $CDCl_3$) δ 7.20 (d, *J* = 8.6 Hz, 1H), 6.73 (d, *J* = 8.6 Hz, 1H), 6.66 (s, 1H), 6.10–6.00 (m, 1H), 5.41 (dd, *J* = 17.2, 1.5 Hz, 1H), 5.27 (dd, *J* = 10.5, 1.4 Hz, 1H), 4.51 (dd, *J* = 3.8, 1.6 Hz, 2H), 2.89 (dd, *J* = 7.7, 3.3 Hz, 2H), 2.51 (dd, *J* = 18.9, 8.6 Hz, 1H), 2.39 (t, *J* = 6.6 Hz, 1H), 2.28–2.22 (m, 1H), 2.16 (q, *J* = 9.1 Hz, 1H), 2.07–1.89 (m, 4H), 1.71–1.26 (m, 5H), 0.91 (s, 3H). ^{13}C NMR (101 MHz, $CDCl_3$) δ 221.2, 156.7, 137.9, 133.6, 132.3, 126.4, 126.4, 117.6, 114.9, 112.4, 68.9, 50.5, 48.2, 44.1, 38.5, 36.0, 31.7, 29.8, 26.0, 21.7, 14.0. HRMS (ESI, m/z): calculated for $C_{21}H_{26}O_2$ $[M+H]^+$ = 311.2006, found 311.2007.

2-(allyloxy)-13-methyl-7,8,9,11,12,13,14,15,16,17-decahydro-6H-cyclopenta[a]phenanthren-17-yl pentanoate (S91)

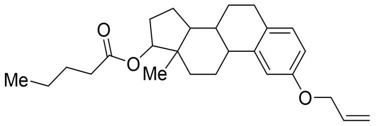

The *O*-allyl substrate **S91** was prepared according to the general procedure **B** from 2-hydroxy-13-methyl-7,8,9,11,12,13,14,15,16,17-decahydro-6H-cyclopenta[a]phenanthren-17-yl pentanoate and 3-bromoprop-1-ene, and purified with chromatography on silica gel (petroleum ether) to afford the title compound in 84% yield as a colorless oil, 1.66 g.

^{1}H NMR (400 MHz, $CDCl_3$) δ 7.20 (d, *J* = 8.6 Hz, 1H), 6.81–6.70 (m, 1H), 6.66 (d, *J* = 2.8 Hz, 1H), 6.06 (ddd, *J* = 20.8, 10.6, 5.2 Hz, 1H), 5.42 (dd, *J* = 17.3, 2.1 Hz, 1H), 5.28 (d, *J* = 10.5 Hz, 1H), 4.72 (t, *J* = 8.6 Hz, 1H), 4.56–4.48 (m, 2H), 2.94–2.79 (m, 2H), 2.42–2.11 (m, 5H), 1.89 (dt, *J* = 12.1, 2.9 Hz, 2H), 1.83–1.71 (m, 1H), 1.66 (t, *J* = 7.6 Hz, 2H), 1.60–1.28 (m, 8H), 1.07–0.77 (m, 7H). ^{13}C NMR (101 MHz, $CDCl_3$) δ 174.0, 156.6, 137.9, 133.7, 132.7,

126.4, 117.5, 114.8, 112.3, 82.5, 68.8, 49.8, 43.9, 43.0, 38.6, 37.0, 34.7, 31.6, 29.8, 27.7, 27.0, 25.2, 23.3, 22.6, 14.1, 12.2.

HRMS (ESI, m/z): calculated for $C_{26}H_{36}O_3$ $[M+H]^+$ = 397.2737, found 397.2739.

4-(but-3-en-1-yl)-2H-benzo[b][1,4]oxazin-3(4H)-one (S92)

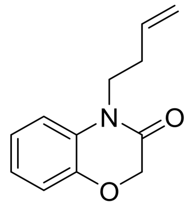

The *N*-allyl substrate **S92** was prepared according to the general procedure **A** from 2H-benzo[b][1,4]oxazin-3(4H)-one and 4-bromoprop-1-ene, and purified with chromatography on silica gel (petroleum ether) to afford the title compound in 89% yield as a colorless oil, 0.90 g.

^{1}H NMR (400 MHz, $CDCl_3$) δ 7.00–6.90 (m, 4H), 5.87–5.73 (m, 1H), 5.11–5.00 (m, 2H), 4.54 (s, 2H), 3.99–3.93 (m, 2H), 2.39 (q, *J* = 7.4 Hz, 2H). ^{13}C NMR (101 MHz, $CDCl_3$) δ 164.1, 145.3, 134.2, 128.3, 123.8, 122.7, 117.4, 117.1, 114.8, 67.5, 40.3, 31.3.

HRMS (ESI, m/z): calculated for $C_{12}H_{13}NO_2$ $[M+H]^+$ = 204.1019, found 204.1022.

4-(hex-5-en-1-yl)-2H-benzo[b][1,4]oxazin-3(4H)-one (S93)

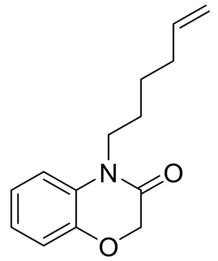

The *N*-allyl substrate **S93** was prepared according to the general procedure **A** from 2H-benzo[b][1,4]oxazin-3(4H)-one and 6-bromohex-1-ene, and purified with chromatography on silica gel (petroleum ether) to afford the title compound in 88% yield as a colorless oil, 1.01 g.

^{1}H NMR (400 MHz, $CDCl_3$) δ 7.02–6.94 (m, 4H), 5.76 (ddd, *J* = 17.0, 8.7, 3.2 Hz, 1H), 5.06–4.88 (m, 2H), 4.54 (d, *J* = 2.6 Hz, 2H), 3.95–3.79 (m, 2H), 2.08 (d, *J* = 7.2 Hz, 2H),

1.70–1.61 (m, 2H), 1.50–1.43 (m, 2H). ^{13}C NMR (101 MHz, $CDCl_3$) δ 164.1, 145.3, 138.2, 128.4, 123.7, 122.7, 117.1, 114.9, 114.8, 67.5, 40.8, 33.2, 26.4, 25.9.

HRMS (ESI, m/z): calculated for $C_{14}H_{17}NO_2$ $[M+H]^+$ = 232.1332, found 232.1333.

7-(p-tolyl)-6,7-dihydro-5H-[1,4]oxazino[2,3,4-ij]quinolin-3(2H)-one (3)

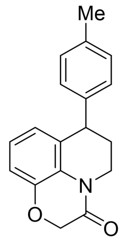

The product was prepared according to the general procedure **C** from 4-allyl-2H-benzo[b][1,4]oxazin-3(4H)-one and 4-methylbenzenesulfonyl chloride, and purified with chromatography on silica gel (petroleum ether) to afford the title compound **3** in 78 % yield as a white solid, 108.8 mg. M.P. 87.3-87.9 °C.

^{1}H NMR (400 MHz, $CDCl_3$) δ 7.13 (dd, *J* = 8.2, 2.8 Hz, 2H), 6.99 (dd, *J* = 8.2, 2.8 Hz, 2H), 6.90–6.82 (m, 2H), 6.59 (q, *J* = 4.2 Hz, 1H), 4.67 (d, *J* = 2.4 Hz, 2H), 4.15 (t, *J* = 5.8 Hz, 1H), 3.95–3.77 (m, 2H), 2.34 (d, *J* = 2.7 Hz, 3H), 2.32–2.04 (m, 2H). ^{13}C NMR (101 MHz, $CDCl_3$) δ 163.7, 144.4, 141.1, 136.6, 129.4, 129.4, 128.4, 128.4, 128.4, 125.5, 123.8, 123.4, 115.0, 67.4, 42.0, 37.3, 29.7, 21.1.

HRMS (ESI, m/z): calculated for $C_{18}H_{17}NO_2$ $[M+H]^+$ = 280.1332, found 280.1333.

(E)-4-(3-(p-tolyl)allyl)-2H-benzo[b][1,4]oxazin-3(4H)-one (4)

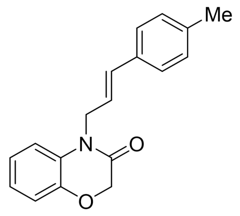

The product was prepared according to the general procedure **C** from 4-allyl-2H-benzo[b][1,4]oxazin-3(4H)-one and 4-methylbenzenesulfonyl chloride, and purified with chromatography on silica gel (petroleum ether) to afford the title compound **4** in 10 % yield as a white solid, 14.0 mg. M.P. 92.3-93.9 °C.

^{1}H NMR (400 MHz, $CDCl_3$) δ 7.31–7.26 (m, 3H), 7.13 (d, *J* = 8.0 Hz, 2H), 7.03 (d, *J* = 3.1 Hz, 3H), 6.60 (d, *J* = 16.0 Hz, 1H), 6.20 (dt, *J* = 16.0, 5.7 Hz, 1H), 4.74 (d, *J* = 5.7 Hz, 2H), 4.69 (d, *J* = 2.0 Hz, 2H), 2.35 (s, 3H). ^{13}C NMR (126 MHz, $CDCl_3$) δ 164.5, 145.4, 137.9, 133.6, 132.8, 129.4, 129.4, 129.0, 126.5, 126.5, 124.1, 123.0, 122.2, 117.1, 115.6, 67.8, 43.6, 21.3.

HRMS (ESI, m/z): calculated for $C_{18}H_{17}NO_2$ $[M+H]^+$ = 280.1332, found 280.1334.

4-(3,3-di-p-tolylallyl)-2H-benzo[b][1,4]oxazin-3(4H)-one (5)

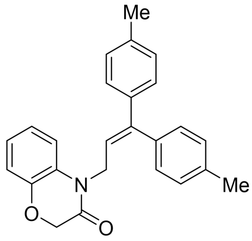

The product was prepared according to the general procedure **C** from 4-allyl-2H-benzo[b][1,4]oxazin-3(4H)-one and 4-methylbenzenesulfonyl chloride, and purified with chromatography on silica gel (petroleum ether) to afford the title compound **5** in 12% yield as a white solid, 22.1 mg. M.P. 96.8-97.7℃.

^{1}H NMR (400 MHz, $CDCl_3$) δ 7.27 (dd, *J* = 4.6, 2.4 Hz, 2H), 7.17 (dd, *J* = 8.1, 2.2 Hz, 2H), 7.12–7.00 (m, 4H), 6.98–6.95 (m, 2H), 6.88 (ddt, *J* = 8.8, 5.8, 2.8 Hz, 1H), 6.71–6.63 (m, 1H), 5.92 (td, *J* = 6.3, 2.2 Hz, 1H), 4.66 (dd, *J* = 6.4, 2.1 Hz, 2H), 4.63 (d, *J* = 2.2 Hz, 2H), 2.43 (d, *J* = 2.2 Hz, 3H), 2.31 (d, *J* = 2.2 Hz, 3H). ^{13}C NMR (101 MHz, $CDCl_3$) δ 164.5, 145.4, 145.1, 138.7, 137.8, 137.6, 136.1, 129.8, 129.8, 129.3, 129.3, 129.0, 129.0, 128.6, 127.5, 127.5, 123.9, 122.7, 122.3, 117.0, 115.7, 67.8, 40.9, 21.4, 21.2.

HRMS (ESI, m/z): calculated for $C_{25}H_{23}NO_2$ $[M+H]^+$ = 370.1802, found 370.1806.

(R)-7-phenyl-6,7-dihydro-5H-[1,4]oxazino[2,3,4-i*J*]quinolin-3(2H)-one (6)

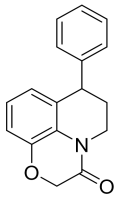

The product was prepared according to the general procedure **C** from 4-allyl-2H-benzo[b][1,4]oxazin-3(4H)-one and benzenesulfonyl chloride, and purified wih chromatography on silica gel (petroleum ether) to afford the title compound **6** in 72% yield as a white solid, 95.4 mg. M.P. 75.2-76.7 ℃.

^{1}H NMR (400 MHz, $CDCl_3$) δ 7.32 (tt, *J* = 8.2, 1.8 Hz, 2H), 7.28–7.22 (m, 1H), 7.14–7.05 (m, 2H), 6.86 (d, *J* = 5.0 Hz, 2H), 6.64–6.53 (m, 1H), 4.67 (s, 2H), 4.19 (dd, *J* = 7.1, 5.0 Hz, 1H),

3.94–3.75 (m, 2H), 2.33–2.08 (m, 2H). ^{13}C NMR (101 MHz, $CDCl_3$) δ 163.7, 144.4, 144.1, 128.8, 128.8, 128.5, 128.5, 128.1, 127.0, 125.5, 123.8, 123.4, 115.1, 67.4, 42.4, 37.3, 29.6.

HRMS (ESI, m/z): calculated for $C_{17}H_{15}NO_2$ $[M+H]^+$ = 266.1176, found 266.1174.

(R)-7-(naphthalen-2-yl)-6,7-dihydro-5H-[1,4]oxazino[2,3,4-i*J*]quinolin-3(2H)-one (7)

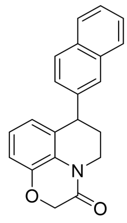

The product was prepared according to the general procedure **C** from 4-allyl-2H-benzo[b][1,4]oxazin-3(4H)-one and naphthalene-2-sulfonyl chloride, and purified with chromatography on silica gel (petroleum ether) to afford the title compound **7** in 69% yield as a white solid, 108.7 mg. M.P. 179.2-181.2 °C.

^{1}H NMR (400 MHz, $CDCl_3$) δ 7.83 (dd, *J* = 8.9, 4.8 Hz, 2H), 7.78–7.74 (m, 1H), 7.53–7.46 (m, 3H), 7.29–7.24 (m, 1H), 6.91 (dd, *J* = 8.1, 1.9 Hz, 1H), 6.87 (t, *J* = 7.7 Hz, 1H), 6.61 (ddd, *J* = 7.3, 1.9, 0.9 Hz, 1H), 4.71 (d, *J* = 1.6 Hz, 2H), 4.35 (dd, *J* = 7.4, 5.1 Hz, 1H), 3.92 (ddd, *J* = 7.4, 4.5, 3.0 Hz, 2H), 2.43–2.16 (m, 2H). ^{13}C NMR (101 MHz, $CDCl_3$) δ 163.6, 144.5, 141.4, 133.4, 132.5, 128.6, 128.0, 127.8, 127.8, 127.5, 126.4, 126.4, 126.0, 125.5, 123.9, 123.5, 115.2, 67.4, 42.6, 37.4, 29.5.

HRMS (ESI, m/z): calculated for $C_{21}H_{17}NO_2$ $[M+H]^+$ = 316.1332, found 316.1335.

(R)-7-(naphthalen-1-yl)-6,7-dihydro-5H-[1,4]oxazino[2,3,4-i*J*]quinolin-3(2H)-one (8)

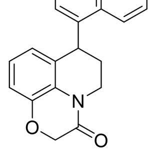

The product was prepared according to the general procedure **C** from 4-allyl-2H-benzo[b][1,4]oxazin-3(4H)-one and naphthalene-1-sulfonyl chloride, and purified

wih chromatography on silica gel (petroleum ether) to afford the title compound **8** in 81% yield as a white solid, 127.6 mg. M.P. 181.4-182.6 °C.

^{1}H NMR (400 MHz, $CDCl_3$) δ 8.12 (d, *J* = 8.3 Hz, 1H), 7.92 (dd, *J* = 8.2, 1.5 Hz, 1H), 7.76 (d, *J* = 8.2 Hz, 1H), 7.56 (ddd, *J* = 8.1, 6.8, 1.4 Hz, 2H), 7.35 (dd, *J* = 8.3, 7.1 Hz, 1H), 6.95–6.83 (m, 3H), 6.64 (ddd, *J* = 7.0, 2.0, 0.8 Hz, 1H), 5.03 (t, *J* = 5.2 Hz, 1H), 4.77–4.65 (m, 2H), 4.14 (dt, *J* = 13.4, 4.9 Hz, 1H), 3.52 (dd, *J* = 13.8, 7.5 Hz, 1H), 2.40–2.29 (m, 2H). ^{13}C NMR (101 MHz, $CDCl_3$) δ 163.7, 144.5, 139.8, 134.2, 131.1, 129.4, 128.0, 127.7, 127.1, 126.6, 126.0, 125.9, 125.4, 124.1, 123.7, 123.1, 115.2, 67.5, 38.3, 36.7, 27.8.

HRMS (ESI, m/z): calculated for $C_{21}H_{17}NO_2$ $[M+H]^+$ = 316.1332, found 316.1333.

(R)-7-(4-methoxyphenyl)-6,7-dihydro-5H-[1,4]oxazino[2,3,4-i*J*]quinolin-3(2H)-one (9)

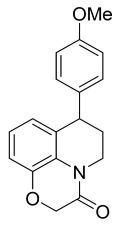

The product was prepared according to the general procedure **C** from 4-allyl-2H-benzo[b][1,4]oxazin-3(4H)-one and 4-methoxybenzenesulfonyl chloride, and purified wih chromatography on silica gel (petroleum ether) to afford the title compound **9** in 83% yield as a white solid, 122.4 mg. M.P. 116.6-117.9 °C.

^{1}H NMR (400 MHz, $CDCl_3$) δ 7.06–6.96 (m, 2H), 6.90–6.80 (m, 4H), 6.60–6.56 (m, 1H), 4.66 (s, 2H), 4.13 (dd, *J* = 7.2, 4.9 Hz, 1H), 3.87 (tdd, *J* = 11.5, 7.7, 4.1 Hz, 2H), 3.80 (s, 3H), 2.29–2.03 (m, 2H). ^{13}C NMR (101 MHz, $CDCl_3$) δ 163.7, 158.5, 144.4, 136.1, 129.5, 129.5, 128.5, 125.4, 123.8, 123.4, 115.1, 114.1, 114.1, 67.5, 55.4, 41.6, 37.4, 29.7.

HRMS (ESI, m/z): calculated for $C_{18}H_{17}NO_3$ $[M+H]^+$ = 296.1281, found 296.1284.

(R)-7-(4-(tert-butyl)phenyl)-6,7-dihydro-5H-[1,4]oxazino[2,3,4-i*J*]quinolin-3(2H)-one (10)

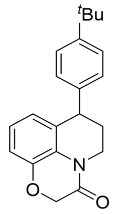

The product was prepared according to the general procedure **C** from 4-allyl-2H-benzo[b][1,4]oxazin-3(4H)-one and 4-(tert-butyl)benzenesulfonyl chloride, and purified with chromatography on silica gel (petroleum ether) to afford the title compound **10** in 62% yield as a white solid, 99.8 mg. M.P. 123.5-124.7 °C.

^{1}H NMR (400 MHz, $CDCl_3$) δ 7.33 (dd, J = 8.7, 2.3 Hz, 2H), 7.03–6.99 (m, 2H), 6.92–6.83 (m, 2H), 6.66–6.57 (m, 1H), 4.67 (s, 2H), 4.17 (dd, J = 6.7, 4.9 Hz, 1H), 3.96–3.71 (m, 2H), 2.31–2.07 (m, 2H), 1.31 (s, 9H). ^{13}C NMR (101 MHz, $CDCl_3$) δ 163.7, 149.8, 144.4, 140.9, 128.3, 128.1, 128.1, 125.6, 125.6, 125.5, 123.9, 123.4, 115.0, 67.4, 41.8, 37.3, 34.6, 31.5, 31.5, 31.5, 29.5.

HRMS (ESI, m/z): calculated for $C_{21}H_{23}NO_2$ $[M+H]^+$ = 322.1802, found 322.1802.

(R)-7-(4-(benzyloxy)phenyl)-6,7-dihydro-5H-[1,4]oxazino[2,3,4-i*J*]quinolin-3(2H)-one (11)

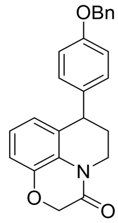

The product was prepared according to the general procedure **C** from 4-allyl-2H-benzo[b][1,4]oxazin-3(4H)-one and 4-(benzyloxy)benzenesulfonyl chloride, and purified wih chromatography on silica gel (petroleum ether) to afford the title compound **11** in 68% yield as a white solid, 126.1 mg. M.P. 128.1-129.6 °C.

^{1}H NMR (400 MHz, $CDCl_3$) δ 7.46–7.31 (m, 5H), 7.05–6.89 (m, 4H), 6.88–6.84 (m, 2H), 6.60 (qd, J = 4.7, 2.4 Hz, 1H), 5.05 (s, 2H), 4.66 (s, 2H), 4.13 (t, J = 6.0 Hz, 1H), 3.96–3.77 (m, 2H), 2.30–2.04 (m, 2H). ^{13}C NMR (101 MHz, $CDCl_3$) δ 163.7, 157.8, 144.4, 137.1, 136.4, 129.5, 129.5, 128.7, 128.7, 128.5, 128.1, 127.6, 127.6, 125.4, 123.8, 123.4, 115.1, 115.1, 115.1, 115.1, 70.2, 67.4, 41.6, 37.3, 29.7.

HRMS (ESI, m/z): calculated for $C_{24}H_{21}NO_3$ $[M+H]^+$ = 372.1594, found 372.1595.

(R)-7-(4-isopropylphenyl)-6,7-dihydro-5H-[1,4]oxazino[2,3,4-i*J*]quinolin-3(2H)-one (12)

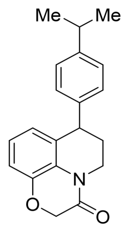

The product was prepared according to the general procedure **C** from 4-allyl-2H-benzo[b][1,4]oxazin-3(4H)-one and 4-isopropylbenzenesulfonyl chloride, and purified wih chromatography on silica gel (petroleum ether) to afford the title compound **12** in 57% yield as a white solid, 87.5 mg. M.P. 107.9-109.2 °C.

^{1}H NMR (400 MHz, $CDCl_3$) δ 7.17 (d, *J* = 8.1 Hz, 2H), 7.00 (d, *J* = 8.3 Hz, 2H), 6.86 (d, *J* = 5.2 Hz, 2H), 6.65–6.55 (m, 1H), 4.66 (s, 2H), 4.16 (t, *J* = 5.9 Hz, 1H), 3.97–3.73 (m, 2H), 2.89 (p, *J* = 6.9 Hz, 1H), 2.19 (ddd, *J* = 13.6, 7.7, 4.1 Hz, 2H), 1.24 (d, *J* = 6.9 Hz, 6H). ^{13}C NMR (101 MHz, $CDCl_3$) δ 163.7, 147.5, 144.4, 141.3, 128.4, 128.4, 128.4, 126.8, 126.8, 125.5, 123.9, 123.4, 115.0, 67.4, 41.9, 37.3, 33.8, 29.5, 24.1, 24.1.

HRMS (ESI, m/z): calculated for $C_{20}H_{21}NO_2$ $[M+H]^+$ = 308.1645, found 308.1647.

(R)-7-(4-cyclohexylphenyl)-6,7-dihydro-5H-[1,4]oxazino[2,3,4-i*J*]quinolin-3(2H)-one (13)

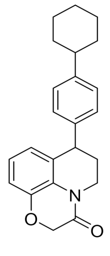

The product was prepared according to the general procedure **C** from 4-allyl-2H-benzo[b][1,4]oxazin-3(4H)-one and 4-cyclohexylbenzenesulfonyl chloride, and purified wih chromatography on silica gel (petroleum ether) to afford the title compound **13** in 60% yield as a white solid, 104.1 mg. M.P. 145.3-146.7 °C.

^1H NMR (400 MHz, $CDCl_3$) δ 7.14 (d, *J* = 8.2 Hz, 2H), 6.99 (d, *J* = 8.2 Hz, 2H), 6.92–6.81 (m, 2H), 6.61 (p, *J* = 4.6 Hz, 1H), 4.66 (s, 2H), 4.15 (dd, *J* = 6.8, 4.9 Hz, 1H), 3.97–3.72 (m, 2H), 2.47 (ddt, *J* = 11.3, 6.4, 3.7 Hz, 1H), 2.31–2.04 (m, 2H), 1.94–1.69 (m, 5H), 1.50–1.19 (m, 5H). ^{13}C NMR (101 MHz, $CDCl_3$) δ 163.7, 146.7, 144.4, 141.3, 128.4, 128.3, 128.3, 127.1, 127.1, 125.5, 123.9, 123.4, 115.0, 67.4, 44.2, 41.9, 37.3, 34.6, 34.5, 29.5, 27.0, 27.0, 26.3.

HRMS (ESI, m/z): calculated for $C_{23}H_{25}NO_2$ $[M+H]^+$ = 348.1958, found 348.1952.

Methyl(R)-3-(4-(3-oxo-2,3,6,7-tetrahydro-5H-[1,4]oxazino[2,3,4-i*J*]quinolin-7-yl) phenyl)propanoate (14)

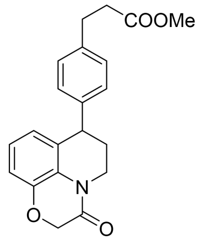

The product was prepared according to the general procedure **C** from 4-allyl-2H-benzo[b][1,4]oxazin-3(4H)-one and methyl 3-(4-(chlorosulfonyl)phenyl)propanoate, and purified wih chromatography on silica gel (petroleum ether) to afford the title compound **14** in 62% yield as a white solid, 108.8 mg. M.P. 136.7-137.9 °C.

^1H NMR (400 MHz, $CDCl_3$) δ 7.14 (d, *J* = 8.1 Hz, 2H), 7.00 (d, *J* = 8.1 Hz, 2H), 6.85 (d, *J* = 4.8 Hz, 2H), 6.57 (td, *J* = 4.5, 0.9 Hz, 1H), 4.66 (s, 2H), 4.15 (dd, *J* = 7.0, 5.0 Hz, 1H), 3.94–3.74 (m, 2H), 3.67 (s, 3H), 2.93 (t, *J* = 7.8 Hz, 2H), 2.70–2.57 (m, 2H), 2.17 (ddd, *J* = 14.0, 7.2, 3.7 Hz, 2H). ^{13}C NMR (101 MHz, $CDCl_3$) δ 173.4, 163.6, 144.4, 142.0, 139.2, 128.6, 128.6, 128.6, 128.6, 128.1, 125.4, 123.8, 123.4, 115.1, 67.4, 51.8, 41.9, 37.2, 35.7, 30.6, 29.5.

HRMS (ESI, m/z): calculated for $C_{21}H_{21}NO_4$ $[M+H]^+$ = 352.1543, found 352.1544.

(R)-7-(4-(difluoromethoxy)phenyl)-6,7-dihydro-5H-[1,4]oxazino[2,3,4-i*J*]quinolin -3(2H)-one (15)

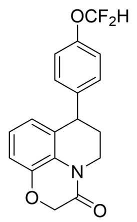

The product was prepared according to the general procedure **C** from 4-allyl-2H-benzo[b][1,4]oxazin-3(4H)-one and 4-(difluoromethoxy)benzenesulfonyl chloride, and purified with chromatography on silica gel (petroleum ether) to afford the title compound **15** in 68% yield as a white solid, 112.5 mg. M.P. 155.4-156.8 °C.

^{1}H NMR (400 MHz, $CDCl_3$) δ 7.08 (d, *J* = 1.5 Hz, 4H), 6.87 (d, *J* = 4.6 Hz, 2H), 6.70–6.29 (m, 2H), 4.66 (s, 2H), 4.18 (dd, *J* = 7.1, 5.0 Hz, 1H), 3.95–3.76 (m, 2H), 2.35–1.98 (m, 2H). ^{19}F NMR (376 MHz, $CDCl_3$) δ -80.48, -80.67. ^{13}C NMR (101 MHz, $CDCl_3$) δ 163.6, 150.0 (t, *J* = 2.9 Hz), 144.5, 141.3, 129.8, 129.8, 127.7, 125.5, 123.6 (d, *J* = 16.5 Hz), 119.9, 118.6, 116.0, 115.3, 113.4, 67.4, 41.7, 37.2, 29.7.

HRMS (ESI, m/z): calculated for $C_{18}H_{15}F_2NO_3$ $[M+H]^+$ = 332.1093, found 332.1090.

(R) -7-(4-(trifluoromethoxy)phenyl)-6,7-dihydro-5H-[1,4]oxazino[2,3,4-i*J*]quinolin-3(2H)-one (16)

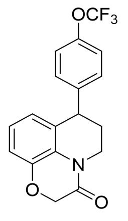

The product was prepared according to the general procedure **C** from 4-allyl-2H-benzo[b][1,4]oxazin-3(4H)-one and 4-(trifluoromethoxy)benzenesulfonyl chloride, and purified wih chromatography on silica gel (petroleum ether) to afford the title compound **16** in 71% yield as a white solid, 123.9 mg. M.P. 147.9-149.4 °C.

^{1}H NMR (400 MHz, $CDCl_3$) δ 7.18–7.09 (m, 4H), 6.88 (d, *J* = 4.2 Hz, 2H), 6.56 (t, *J* = 4.6 Hz, 1H), 4.67 (s, 2H), 4.25–4.16 (m, 1H), 3.96–3.75 (m, 2H), 2.37–2.00 (m, 2H). ^{19}F NMR (376 MHz, $CDCl_3$) δ -57.70, -57.70, -57.70. ^{13}C NMR (101 MHz, $CDCl_3$) δ 163.6, 148.1, 144.5, 142.8, 129.8, 129.8, 127.4, 125.5, 123.7, 123.6, 121.3, 121.3, 119.3, 115.4, 67.4, 41.8, 37.2, 29.6.

HRMS (ESI, m/z): calculated for $C_{18}H_{14}F_3NO_3$ $[M+H]^+$ = 350.0999, found 350.0993.

(R)-7-(4-(trifluoromethyl)phenyl)-6,7-dihydro-5H-[1,4]oxazino[2,3,4-i*J*]quinolin-3(2H)-one (17)

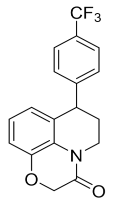

The product was prepared according to the general procedure **C** from 4-allyl-2H-benzo[b][1,4]oxazin-3(4H)-one and 4-(trifluoromethyl)benzenesulfonyl chloride, and purified wih chromatography on silica gel (petroleum ether) to afford the title compound **17** in 57% yield as a white solid, 94.9 mg. M.P. 57.8-59.2 °C.

^{1}H NMR (400 MHz, $CDCl_3$) δ 7.66–7.48 (m, 4H), 7.03–6.97 (m, 3H), 5.01 (dd, *J* = 8.1, 6.0 Hz, 1H), 4.63–4.48 (m, 2H), 4.28–4.00 (m, 2H), 2.48–2.41 (m, 2H). ^{19}F NMR (376 MHz, $CDCl_3$) δ -62.54, -62.54, -62.54. ^{13}C NMR (101 MHz, $CDCl_3$) δ 164.6, 145.5, 144.6, 128.3, 127.4, 127.4, 126.0, 126.0, 125.9, 125.9, 124.3, 123.1, 117.5, 114.7, 67.7, 60.1, 39.1, 36.9. HRMS (ESI, m/z): calculated for $C_{18}H_{14}F_3NO_2$ $[M+H]^+$ = 334.1049, found 334.1052.

(R)-7-(4-acetylphenyl)-6,7-dihydro-5H-[1,4]oxazino[2,3,4-i*J*]quinolin-3(2H)-one (18)

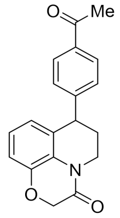

The product was prepared according to the general procedure **C** from 4-allyl-2H-benzo[b][1,4]oxazin-3(4H)-one and 4-acetylbenzenesulfonyl chloride, and purified wih chromatography on silica gel (petroleum ether) to afford the title compound **18** in 78% yield as a white solid, 119.7 mg. M.P. 73.7-74.3 °C.

^{1}H NMR (400 MHz, $CDCl_3$) δ 7.92 (dd, *J* = 8.3, 1.6 Hz, 2H), 7.20 (dd, *J* = 8.3, 1.5 Hz, 2H), 6.92–6.84 (m, 2H), 6.56–6.50 (m, 1H), 4.67 (s, 2H), 4.25 (t, *J* = 6.1 Hz, 1H), 3.97–3.78 (m,

2H), 2.59 (d, J = 1.3 Hz, 3H), 2.35–2.08 (m, 2H). ^{13}C NMR (101 MHz, $CDCl_3$) δ 197.8, 163.6, 149.6, 144.6, 136.0, 128.9, 128.9, 128.8, 128.8, 127.2, 125.5, 123.7, 123.6, 115.5, 67.4, 42.4, 37.2, 29.5, 26.7.

HRMS (ESI, m/z): calculated for $C_{19}H_{17}NO_3$ $[M+H]^+$ = 308.1281, found 308.1280.

(R)-7-(4-nitrophenyl)-6,7-dihydro-5H-[1,4]oxazino[2,3,4-i*J*]quinolin-3(2H)-one (19)

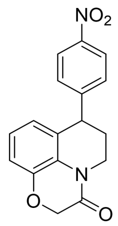

The product was prepared according to the general procedure **C** from 4-allyl-2H-benzo[b][1,4]oxazin-3(4H)-one and 4-nitrobenzenesulfonyl chloride, and purified wih chromatography on silica gel (petroleum ether) to afford the title compound **19** in 66% yield as a white solid, 102.3 mg. M.P. 92.3-92.9 °C.

^{1}H NMR (400 MHz, $CDCl_3$) δ 8.26–8.16 (m, 2H), 7.57 (d, J = 8.8 Hz, 2H), 7.10–6.98 (m, 3H), 5.04 (t, J = 7.0 Hz, 1H), 4.55 (d, J = 6.3 Hz, 2H), 4.29–3.99 (m, 2H), 2.44 (q, J = 7.4 Hz, 2H). ^{13}C NMR (101 MHz, $CDCl_3$) δ 164.7, 148.0, 147.6, 145.5, 128.2, 128.0, 128.0, 124.4, 124.2, 124.2, 123.2, 117.6, 114.6, 67.6, 59.5, 39.0, 36.8.

HRMS (ESI, m/z): calculated for $C_{17}H_{14}N_2O_4$ $[M+H]^+$ = 311.1026, found 311.1026.

(R)-4-(3-oxo-2,3,6,7-tetrahydro-5H-[1,4]oxazino[2,3,4-i*J*]quinolin-7-yl)benzonitrile (20)

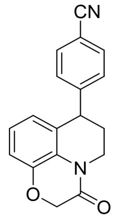

The product was prepared according to the general procedure **C** ($MeNO_2$ instead of DMC) from 4-allyl-2H-benzo[b][1,4]oxazin-3(4H)-one and 4-cyanobenzenesulfonyl chloride, and purified wih chromatography on silica gel (petroleum ether) to afford the title compound **20**

in 67% yield as a white solid, 97.2 mg. M.P. 155.3-156.4 °C.

^{1}H NMR (400 MHz, $CDCl_3$) δ 7.66–7.60 (m, 2H), 7.25–7.19 (m, 2H), 6.95–6.83 (m, 2H), 6.57–6.48 (m, 1H), 4.67 (s, 2H), 4.26 (dd, *J* = 7.1, 5.1 Hz, 1H), 3.96–3.75 (m, 2H), 2.37–2.05 (m, 2H). ^{13}C NMR (101 MHz, $CDCl_3$) δ 163.5, 149.6, 144.6, 132.6, 132.6, 129.4, 129.4, 126.4, 125.5, 123.7, 123.6, 118.8, 115.7, 111.0, 67.4, 42.4, 36.9, 29.4.

HRMS (ESI, m/z): calculated for $C_{18}H_{14}N_2O_2$ $[M+H]^+$ = 291.1128, found 291.1127.

(R)-7-([1,1'-biphenyl]-4-yl)-6,7-dihydro-5H-[1,4]oxazino[2,3,4-i*J*]quinolin-3(2H)-one (21)

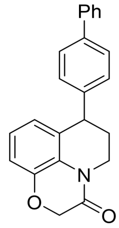

The product was prepared according to the general procedure **C** ($MeNO_2$ instead of DMC) from 4-allyl-2H-benzo[b][1,4]oxazin-3(4H)-one and [1,1'-biphenyl]-4-sulfonyl chloride, and purified wih chromatography on silica gel (petroleum ether) to afford the title compound **21** in 70% yield as a white solid, 119.4 mg. M.P. 133.9-134.8 °C.

^{1}H NMR (400 MHz, $CDCl_3$) δ 7.61–7.53 (m, 4H), 7.44 (t, *J* = 7.7 Hz, 2H), 7.35 (t, *J* = 7.3 Hz, 1H), 7.17 (d, *J* = 8.2 Hz, 2H), 6.93–6.86 (m, 2H), 6.65 (dd, *J* = 5.7, 3.4 Hz, 1H), 4.69 (s, 2H), 4.29–4.17 (m, 1H), 4.02–3.79 (m, 2H), 2.40–2.05 (m, 2H). ^{13}C NMR (101 MHz, $CDCl_3$) δ 163.7, 144.5, 143.1, 140.8, 139.9, 128.9, 128.9, 128.9, 128.9, 128.0, 127.5, 127.5, 127.4, 127.2, 127.2, 125.5, 123.9, 123.5, 115.2, 67.4, 42.0, 37.2, 29.6.

HRMS (ESI, m/z): calculated for $C_{23}H_{19}NO_2$ $[M+H]^+$ = 342.1489, found 342.1490.

(R)-7-(4-fluorophenyl)-6,7-dihydro-5H-[1,4]oxazino[2,3,4-i*J*]quinolin-3(2H)-one (22)

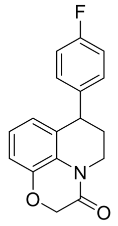

The product was prepared according to the general procedure **C** from 4-allyl-2H-benzo[b][1,4]oxazin-3(4H)-one and 4-fluorobenzenesulfonyl chloride, and purified with chromatography on silica gel (petroleum ether) to afford the title compound **22** in 74% yield as a white solid, 104.7 mg. M.P. 110.2-111.7 °C.

^{1}H NMR (400 MHz, $CDCl_3$) δ 7.11–6.95 (m, 4H), 6.87 (d, *J* = 4.6 Hz, 2H), 6.55 (pd, *J* = 4.1, 0.8 Hz, 1H), 4.66 (s, 2H), 4.17 (dd, *J* = 7.2, 4.9 Hz, 1H), 3.85 (qdd, *J* = 13.2, 9.8, 4.1 Hz, 2H), 2.39–1.96 (m, 2H). ^{19}F NMR (376 MHz, $CDCl_3$) δ -115.75 (t, *J* = 8.9 Hz). ^{13}C NMR (101 MHz, $CDCl_3$) δ 163.6, 163.0, 160.5, 144.4, 139.7 (d, *J* = 3.3 Hz), 129.9 (d, *J* = 7.9 Hz), 127.9, 125.4, 123.6, 123.4, 115.7, 115.5, 115.2, 67.4, 41.6, 37.2, 29.7.

HRMS (ESI, m/z): calculated for $C_{17}H_{14}FNO_2$ $[M+H]^+$ = 284.1081, found 284.1082.

(R)-7-(4-chlorophenyl)-6,7-dihydro-5H-[1,4]oxazino[2,3,4-i*J*]quinolin-3(2H)-one (23)

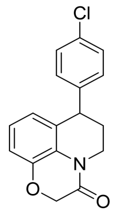

The product was prepared according to the general procedure **C** from 4-allyl-2H-benzo[b][1,4]oxazin-3(4H)-one and 4-chlorobenzenesulfonyl chloride, and purified with chromatography on silica gel (petroleum ether) to afford the title compound **23** in 73% yield as a white solid, 109.1 mg. M.P. 123.8-124.6 °C.

^{1}H NMR (400 MHz, $CDCl_3$) δ 7.32–7.26 (m, 2H), 7.08–6.98 (m, 2H), 6.92–6.82 (m, 2H), 6.54 (pd, *J* = 4.1, 0.9 Hz, 1H), 4.66 (s, 2H), 4.16 (dd, *J* = 7.2, 5.0 Hz, 1H), 3.94–3.77 (m, 2H), 2.43–1.91 (m, 2H). ^{13}C NMR (101 MHz, $CDCl_3$) δ 163.6, 144.5, 142.6, 132.8, 129.9, 129.9, 128.9, 128.9, 127.5, 125.5, 123.7, 123.5, 115.4, 67.4, 41.8, 37.2, 29.6.

HRMS (ESI, m/z): calculated for $C_{17}H_{14}ClNO_2$ $[M+H]^+$ = 300.0786, found 300.0782.

(R)-7-(4-bromophenyl)-6,7-dihydro-5H-[1,4]oxazino[2,3,4-i*J*]quinolin-3(2H)-one (24)

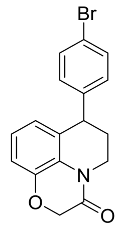

The product was prepared according to the general procedure **C** from 4-allyl-2H-benzo[b][1,4]oxazin-3(4H)-one and 4-bromobenzenesulfonyl chloride, and purified with chromatography on silica gel (petroleum ether) to afford the title compound **24** in 65% yield as a white solid, 111.5 mg. M.P. 89.7-90.6 °C.

^{1}H NMR (400 MHz, $CDCl_3$) δ 7.44 (dd, J = 8.8, 2.3 Hz, 2H), 7.06–6.82 (m, 4H), 6.59–6.51 (m, 1H), 4.66 (d, J = 2.1 Hz, 2H), 4.14 (dd, J = 7.3, 5.0 Hz, 1H), 3.85 (tdd, J = 17.3, 9.8, 3.6 Hz, 2H), 2.41–1.98 (m, 2H). ^{13}C NMR (101 MHz, $CDCl_3$) δ 163.6, 144.5, 143.1, 131.9, 131.9, 130.3, 130.3, 127.5, 125.5, 123.7, 123.5, 120.9, 115.4, 67.4, 41.9, 37.2, 29.6.

HRMS (ESI, m/z): calculated for $C_{17}H_{14}BrNO_2$ $[M+H]^+$ = 344.0281, found 344.0282.

(R)-7-(3-isopropylphenyl)-6,7-dihydro-5H-[1,4]oxazino[2,3,4-i*J*]quinolin-3(2H)-one (25)

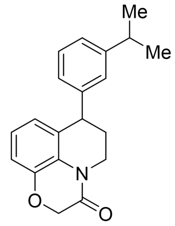

The product was prepared according to the general procedure **C** from 4-allyl-2H-benzo[b][1,4]oxazin-3(4H)-one and 3-isopropylbenzenesulfonyl chloride, and purified wih chromatography on silica gel (petroleum ether) to afford the title compound **25** in 71% yield as a white solid, 109.0 mg. M.P. 104.5-105.9 °C.

^{1}H NMR (400 MHz, $CDCl_3$) δ 7.23 (t, J = 7.6 Hz, 1H), 7.15–7.10 (m, 1H), 6.98 (t, J = 2.0 Hz, 1H), 6.89–6.84 (m, 3H), 6.59 (pd, J = 4.2, 0.9 Hz, 1H), 4.67 (s, 2H), 4.16 (dd, J = 7.3, 5.0 Hz, 1H), 3.88 (t, J = 5.9 Hz, 2H), 2.87 (hept, J = 6.9 Hz, 1H), 2.33–2.06 (m, 2H), 1.23 (dd, J = 7.0, 1.0 Hz, 6H). ^{13}C NMR (101 MHz, $CDCl_3$) δ 163.6, 149.4, 144.4, 143.9, 128.6, 128.3, 126.8, 125.9, 125.4, 124.9, 123.8, 123.3, 115.0, 67.4, 42.5, 37.4, 34.1, 29.6, 24.1, 24.1.

HRMS (ESI, m/z): calculated for $C_{20}H_{21}NO_2$ $[M+H]^+$ = 308.1645, found 308.1645.

(R)-7-(m-tolyl)-6,7-dihydro-5H-[1,4]oxazino[2,3,4-i*J*]quinolin-3(2H)-one(26)

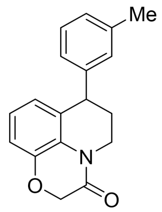

The product was prepared according to the general procedure **C** from 4-allyl-2H-benzo[b][1,4]oxazin-3(4H)-one and 3-methylbenzenesulfonyl chloride, and purified wih chromatography on silica gel (petroleum ether) to afford the title compound **26** in 70% yield as a white solid, 97.7 mg. M.P. 99.8-101.3 ℃.

^{1}H NMR (400 MHz, $CDCl_3$) δ 7.21 (t, *J* = 7.6 Hz, 1H), 7.08 (d, *J* = 7.5 Hz, 1H), 6.97–6.82 (m, 4H), 6.60 (dd, *J* = 5.4, 3.7 Hz, 1H), 4.67 (s, 2H), 4.15 (dd, *J* = 7.2, 5.0 Hz, 1H), 3.95–3.80 (m, 2H), 2.34 (s, 3H), 2.30–2.08 (m, 2H). ^{13}C NMR (101 MHz, $CDCl_3$) δ 163.5, 144.3, 144.0, 138.3, 129.1, 128.5, 128.2, 127.6, 125.5, 125.4, 123.8, 123.3, 115.0, 67.3, 42.2, 37.3, 29.5, 21.5.

HRMS (ESI, m/z): calculated for $C_{18}H_{17}NO_2$ $[M+H]^+$ = 280.1332, found 280.1333.

(R)-7-(3-chlorophenyl)-6,7-dihydro-5H-[1,4]oxazino[2,3,4-i*J*]quinolin-3(2H)-one (27)

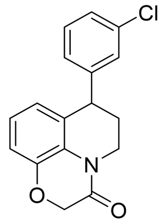

The product was prepared according to the general procedure **C** from 4-allyl-2H-benzo[b][1,4]oxazin-3(4H)-one and 3-chlorobenzenesulfonyl chloride, and purified wih chromatography on silica gel (petroleum ether) to afford the title compound **27** in 77% yield as a white solid, 115.1 mg. M.P. 117.4-118.4 ℃.

^{1}H NMR (400 MHz, $CDCl_3$) δ 7.25–7.23 (m, 2H), 7.09 (s, 1H), 6.98 (ddd, *J* = 6.5, 3.4, 1.7 Hz, 1H), 6.91–6.85 (m, 2H), 6.56 (pd, *J* = 4.1, 0.9 Hz, 1H), 4.67 (s, 2H), 4.21–4.09 (m, 1H), 3.94–3.77 (m, 2H), 2.32–2.04 (m, 2H). ^{13}C NMR (101 MHz, $CDCl_3$) δ 163.6, 146.1, 144.5, 134.6, 130.0, 128.6, 127.2, 127.2, 126.7, 125.5, 123.7, 123.6, 115.4, 67.4, 42.1, 37.1, 29.5.

HRMS (ESI, m/z): calculated for $C_{17}H_{14}ClNO_2$ $[M+H]^+$ = 300.0786, found 300.0791.

(R)-7-(3-bromophenyl)-6,7-dihydro-5H-[1,4]oxazino[2,3,4-i*J*]quinolin-3(2H)-one (28)

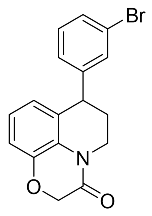

The product was prepared according to the general procedure **C** from 4-allyl-2H-benzo[b][1,4]oxazin-3(4H)-one and 3-bromobenzenesulfonyl chloride, and purified wih chromatography on silica gel (petroleum ether) to afford the title compound **28** in 65% yield as a white solid, 111.5 mg. M.P. 62.4-64.5 °C.

^{1}H NMR (400 MHz, $CDCl_3$) δ 7.39 (ddd, *J* = 8.0, 2.0, 1.0 Hz, 1H), 7.25 (t, *J* = 1.8 Hz, 1H), 7.19 (t, *J* = 7.8 Hz, 1H), 7.04–6.98 (m, 1H), 6.89–6.86 (m, 2H), 6.56 (td, *J* = 4.4, 0.9 Hz, 1H), 4.67 (s, 2H), 4.16 (dd, *J* = 7.1, 5.0 Hz, 1H), 3.94–3.77 (m, 2H), 2.32–2.03 (m, 2H). ^{13}C NMR (101 MHz,$CDCl_3$) δ 163.6, 146.4, 144.5, 131.5, 130.3, 130.2, 127.2, 127.1, 125.5, 123.7, 123.6, 122.9, 115.4, 67.4, 42.1, 37.1, 29.5.

HRMS (ESI, m/z): calculated for $C_{17}H_{14}BrNO_2$ $[M+H]^+$ = 344.0281, found 344.0284.

(R)-7-(3-fluorophenyl)-6,7-dihydro-5H-[1,4]oxazino[2,3,4-i*J*]quinolin-3(2H)-one (29)

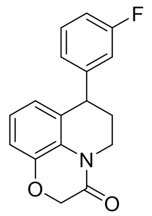

The product was prepared according to the general procedure **C** from 4-allyl-2H-benzo[b][1,4]oxazin-3(4H)-one and 3-fluorobenzenesulfonyl chloride, and purified wih chromatography on silica gel (petroleum ether) to afford the title compound **29** in 72% yield as a white solid, 101.9 mg. M.P. 117.4-119.6 °C.

^{1}H NMR (400 MHz, $CDCl_3$) δ 7.33–7.27 (m, 1H), 6.95 (tdd, *J* = 8.4, 2.6, 1.0 Hz, 1H), 6.89 (dd, *J* = 5.5, 4.5 Hz, 3H), 6.78 (dt, *J* = 10.0, 2.2 Hz, 1H), 6.62–6.55 (m, 1H), 4.67 (s, 2H), 4.19 (dd, *J* = 6.9, 5.0 Hz, 1H), 3.98–3.73 (m, 2H), 2.36–2.04 (m, 2H). ^{19}F NMR (376 MHz, $CDCl_3$) δ -112.50 (q, *J* = 8.9 Hz). ^{13}C NMR (101 MHz, $CDCl_3$) δ 164.3, 163.6, 161.9, 146.6 (d, *J* = 6.9 Hz), 144.5, 130.2 (d, *J* = 8.3 Hz), 127.3, 125.5, 124.2 (d, *J* = 2.7 Hz), 123.6 (d, *J* = 17.2 Hz), 115.6, 115.4 (d, *J* = 6.1 Hz), 113.9 (d, *J* = 21.2 Hz), 67.4, 42.1, 37.1, 29.5.

HRMS (ESI, m/z): calculated for $C_{17}H_{14}FNO_2$ $[M+H]^+$ = 284.1081, found 284.1081.

(R)-7-(3-(methylsulfonyl)phenyl)-6,7-dihydro-5H-[1,4]oxazino[2,3,4-ij]quinolin-3(2H)-one (30)

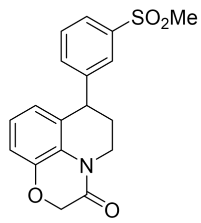

The product was prepared according to the general procedure **C** from 4-allyl-2H-benzo[b][1,4]oxazin-3(4H)-one and 3-(methylsulfonyl)benzenesulfonyl chloride, and purified wih chromatography on silica gel (petroleum ether) to afford the title compound **30** in 59% yield as a white solid, 101.2 mg. M.P. 114.4-115.6 °C.

^{1}H NMR (400 MHz, $CDCl_3$) δ 7.84 (d, *J* = 7.8 Hz, 1H), 7.77 (t, *J* = 1.8 Hz, 1H), 7.53 (td, *J* = 7.8, 2.0 Hz, 1H), 7.36 (d, *J* = 7.8 Hz, 1H), 6.94–6.82 (m, 2H), 6.49 (dd, *J* = 6.3, 2.8 Hz, 1H), 4.68 (d, *J* = 2.1 Hz, 2H), 4.29 (t, *J* = 6.2 Hz, 1H), 3.88 (dt, *J* = 7.4, 4.2 Hz, 2H), 3.06 (d, *J* = 2.0 Hz, 3H), 2.42–2.25 (m, 1H), 2.13 (ddd, *J* = 13.4, 7.0, 4.9 Hz, 1H). ^{13}C NMR (126 MHz, $CDCl_3$) δ 163.5, 146.1, 144.7, 141.3, 133.9, 129.9, 127.2, 126.6, 126.1, 125.6, 123.7, 123.5, 115.7, 67.4, 44.6, 42.4, 37.2, 29.8.

HRMS (ESI, m/z): calculated for $C_{18}H_{17}NO_4S$ $[M+H]^+$ = 344.0951, found 344.0952.

(R)-7-(2-chloro-4-fluorophenyl)-6,7-dihydro-5H-[1,4]oxazino[2,3,4-i*J*]quinolin-3(2H)-one (31)

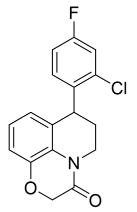

The product was prepared according to the general procedure **C** from 4-allyl-2H-benzo[b][1,4]oxazin-3(4H)-one and 2-chloro-4-fluorobenzenesulfonyl chloride, and purified wih chromatography on silica gel (petroleum ether) to afford the title compound **31** in 52% yield as a white solid, 82.4 mg. M.P. 132.6-134.5 °C.

^{1}H NMR (400 MHz, $CDCl_3$) δ 7.13 (dd, *J* = 7.0, 2.3 Hz, 1H), 7.09 (t, *J* = 8.6 Hz, 1H), 6.96 (ddd, *J* = 8.5, 4.6, 2.3 Hz, 1H), 6.94–6.83 (m, 2H), 6.54 (pd, *J* = 4.2, 0.9 Hz, 1H), 4.67 (s, 2H), 4.15 (dd, *J* = 7.2, 5.0 Hz, 1H), 3.85 (qdd, *J* = 13.3, 7.7, 3.9 Hz, 2H), 2.35– 2.00 (m, 2H). ^{19}F NMR (376 MHz, $CDCl_3$) δ -117.81. ^{13}C NMR (101 MHz, $CDCl_3$) δ 163.6, 158.3, 155.9, 144.5, 141.1 (d, *J* = 3.8 Hz), 130.5, 128.2 (d, *J* = 7.1 Hz), 127.0, 125.4, 123.6 (d, *J* = 3.8 Hz), 121.2 (d, *J* = 17.8 Hz), 116.8 (d, *J* = 21.0 Hz), 115.5, 67.4, 41.5, 37.1, 29.6.

HRMS (ESI, m/z): calculated for $C_{17}H_{13}ClFNO_2$ $[M+H]^+$ = 318.0692, found 318.0692.

(R)-7-(3-chloro-2-methylphenyl)-6,7-dihydro-5H-[1,4]oxazino[2,3,4-i*J*]quinolin-3(2H)-one (32)

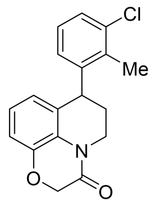

The product was prepared according to the general procedure **C** from 4-allyl-2H-benzo[b][1,4]oxazin-3(4H)-one and 3-chloro-2-methylbenzenesulfonyl chloride, and purified wih chromatography on silica gel (petroleum ether) to afford the title compound **32** in 71% yield as a white solid, 111.1 mg. M.P. 105.3-106.7 °C.

^{1}H NMR (400 MHz, $CDCl_3$) δ 7.27 (d, *J* = 9.4 Hz, 1H), 7.02 (t, *J* = 7.9 Hz, 1H), 6.87 (dt, *J* = 5.3, 1.3 Hz, 2H), 6.68 (d, *J* = 7.8 Hz, 1H), 6.52 (t, *J* = 4.6 Hz, 1H), 4.67 (d, *J* = 1.7 Hz, 2H), 4.45 (t, *J* = 5.9 Hz, 1H), 4.01–3.71 (m, 2H), 2.46 (s, 3H), 2.29–1.97 (m, 2H). ^{13}C NMR (101 MHz, $CDCl_3$) δ 163.6, 144.5, 144.0, 135.6, 133.9, 127.9, 127.9, 127.5, 126.8, 125.7, 123.6, 123.5, 115.2, 67.4, 39.5, 37.0, 27.7, 16.1.

HRMS (ESI, m/z): calculated for $C_{18}H_{16}ClNO_2$ $[M+H]^+$ = 314.0942, found 314.0940.

(R)-7-(4-bromo-3-methylphenyl)-6,7-dihydro-5H-[1,4]oxazino[2,3,4-i*J*]quinolin-3(2H)-one (33)

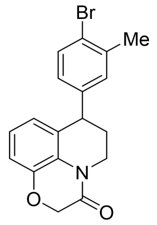

The product was prepared according to the general procedure **C** from 4-allyl-2H-benzo[b][1,4]oxazin-3(4H)-one and 4-bromo-3-methylbenzenesulfonyl chloride, and purified wih chromatography on silica gel (petroleum ether) to afford the title compound **33** in 59% yield as a white solid, 105.3 mg. M.P. 97.8-99.6 °C.

^{1}H NMR (400 MHz, $CDCl_3$) δ 7.45 (d, *J* = 8.2 Hz, 1H), 6.97 (d, *J* = 2.3 Hz, 1H), 6.91–6.82 (m, 2H), 6.77 (dd, *J* = 8.2, 2.3 Hz, 1H), 6.59–6.50 (m, 1H), 4.66 (s, 2H), 4.10 (dd, *J* = 7.3, 5.0 Hz, 1H), 3.86 (dd, *J* = 6.8, 5.1 Hz, 2H), 2.36 (s, 3H), 2.31–2.01 (m, 2H). ^{13}C NMR (101 MHz, $CDCl_3$) δ 163.6, 144.5, 143.4, 138.3, 132.6, 130.9, 127.6, 127.5, 125.4, 123.7, 123.5, 123.3, 115.3, 67.4, 41.9, 37.3, 29.6, 23.1.

HRMS (ESI, m/z): calculated for $C_{18}H_{16}BrNO_2$ $[M+H]^+$ = 358.0437, found 358.0435.

(R)-7-(4-fluoro-3-(trifluoromethyl)phenyl)-6,7-dihydro-5H-[1,4]oxazino[2,3,4-i*J*]quinolin-3(2H)-one (34)

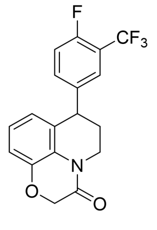

The product was prepared according to the general procedure **C** from 4-allyl-2H-benzo[b][1,4]oxazin-3(4H)-one and 4-fluoro-3-(trifluoromethyl)benzenesulfonyl chloride, and purified wih chromatography on silica gel (petroleum ether) to afford the title compound **34** in 62% yield as a white solid, 108.8 mg. M.P. 150.9-152.4 °C.

^{1}H NMR (400 MHz, $CDCl_3$) δ 7.38 (dd, *J* = 6.6, 2.3 Hz, 1H), 7.26–7.22 (m, 1H), 7.15 (t, *J* = 9.2 Hz, 1H), 6.94–6.83 (m, 2H), 6.56–6.45 (m, 1H), 4.67 (s, 2H), 4.23 (dd, *J* = 7.6, 5.0 Hz, 1H), 3.88 (dd, *J* = 6.9, 5.2 Hz, 2H), 2.36–2.01 (m, 2H). ^{19}F NMR (376 MHz, $CDCl_3$) δ -61.18 (d, *J* = 11.9 Hz), -61.18 (d, *J* = 11.9 Hz), -61.18 (d, *J* = 11.9 Hz), -116.82 (h, *J* = 14.9 Hz). ^{13}C NMR (101 MHz, $CDCl_3$) δ 163.6, 144.6, 140.3, 140.3, 133.9, 133.8, 127.0 (d, *J* = 4.8 Hz), 126.8, 125.5, 123.7, 123.5, 117.4, 117.2, 115.7, 67.4, 41.7, 37.2, 29.8.

HRMS (ESI, m/z): calculated for $C_{18}H_{13}F_4NO_2$ $[M+H]^+$ = 352.0955, found 352.0956.

(R)-7-(3-chloro-4-methylphenyl)-6,7-dihydro-5H-[1,4]oxazino[2,3,4-i*J*]quinolin-3(2H)-one (35)

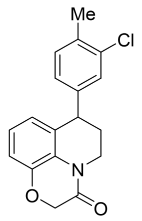

The product was prepared according to the general procedure **C** from 4-allyl-2H-benzo[b][1,4]oxazin-3(4H)-one and 3-chloro-4-methylbenzenesulfonyl chloride, and purified with chromatography on silica gel (petroleum ether) to afford the title compound **35** in 72% yield as a white solid, 112.7 mg. M.P. 122.7-123.9 °C.

^{1}H NMR (400 MHz, $CDCl_3$) δ 7.17 (d, *J* = 7.8 Hz, 1H), 7.08 (d, *J* = 1.9 Hz, 1H), 6.94–6.80 (m, 3H), 6.57 (tt, *J* = 4.4, 0.9 Hz, 1H), 4.67 (s, 2H), 4.24–4.01 (m, 1H), 3.95–3.75 (m, 2H), 2.35 (s, 3H), 2.31–1.99 (m, 2H). ^{13}C NMR (101 MHz, $CDCl_3$) δ 163.6, 144.5, 143.4, 134.7, 134.7, 131.2, 129.0, 127.5, 126.8, 125.5, 123.7, 123.5, 115.3, 67.4, 41.8, 37.2, 29.6, 19.8.

HRMS (ESI, m/z): calculated for $C_{18}H_{16}ClNO_2$ $[M+H]^+$ = 314.0942, found 314.0943.

(R)-7-(2,4-dimethylphenyl)-6,7-dihydro-5H-[1,4]oxazino[2,3,4-i*J*]quinolin-3(2H)-one (36)

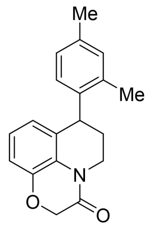

The product was prepared according to the general procedure **C** from 4-allyl-2H-benzo[b][1,4]oxazin-3(4H)-one and 2,4-dimethylbenzenesulfonyl chloride, and purified wih chromatography on silica gel (petroleum ether) to afford the title compound **36** in 68% yield as a white solid, 99.6 mg. M.P. 112.9-114.3 °C.

^{1}H NMR (400 MHz, $CDCl_3$) δ 7.05 (s, 1H), 6.92 (d, *J* = 7.9 Hz, 1H), 6.89–6.81 (m, 2H), 6.68 (d, *J* = 6.8 Hz, 1H), 6.52 (ddt, *J* = 5.2, 4.1, 1.1 Hz, 1H), 4.68 (d, *J* = 1.7 Hz, 2H), 4.35 (dd, *J* = 6.9, 5.2 Hz, 1H), 3.89 (tt, *J* = 13.1, 6.8 Hz, 2H), 2.39 (s, 3H), 2.30 (s, 3H), 2.25–1.98 (m, 2H).

^{13}C NMR (101 MHz, $CDCl_3$) δ 163.6, 144.4, 139.1, 136.4, 135.6, 131.6, 128.7, 128.7, 127.0, 125.7, 123.6, 123.5, 114.9, 67.4, 38.3, 37.3, 27.8, 21.0, 19.6.

HRMS (ESI, m/z): calculated for $C_{19}H_{19}NO_2$ $[M+H]^+$ = 294.1489, found 294.1491.

(R)-7-(5,6,7,8-tetrahydronaphthalen-2-yl)-6,7-dihydro-5H-[1,4]oxazino[2,3,4-i*J*]quinolin-3(2H)-one (37)

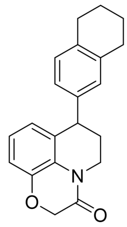

The product was prepared according to the general procedure **C** from 4-allyl-2H-benzo[b][1,4]oxazin-3(4H)-one and 5,6,7,8-tetrahydronaphthalene-2-sulfonyl chloride, and purified wih chromatography on silica gel (petroleum ether) to afford the title compound **37** in 60% yield as a white solid, 95.7 mg. M.P. 102.4-103.8 °C.

^{1}H NMR (400 MHz, $CDCl_3$) δ 7.00 (d, *J* = 8.3 Hz, 1H), 6.89–6.83 (m, 2H), 6.79 (d, *J* = 5.7 Hz, 2H), 6.65–6.56 (m, 1H), 4.66 (s, 2H), 4.10 (dd, *J* = 7.0, 5.1 Hz, 1H), 3.86 (pt, *J* = 7.6, 4.2 Hz, 2H), 2.74 (dd, *J* = 11.9, 5.7 Hz, 4H), 2.31–2.04 (m, 2H), 1.79 (dq, *J* = 6.4, 3.2 Hz, 4H).

^{13}C NMR (101 MHz, $CDCl_3$) δ 163.7, 144.4, 141.2, 137.5, 135.8, 129.5, 129.2, 128.5, 125.6, 125.4, 123.9, 123.4, 115.0, 67.4, 42.0, 37.3, 29.7, 29.6, 29.1, 23.3, 23.3.

HRMS (ESI, m/z): calculated for $C_{21}H_{21}NO_2$ $[M+H]^+$ = 320.1645, found 320.1650.

(S)-7-(6-chloropyridin-3-yl)-6,7-dihydro-5H-[1,4]oxazino[2,3,4-i*J*]quinolin-3(2H)-one (38)

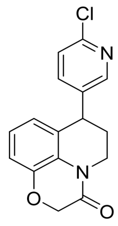

The product was prepared according to the general procedure **C** from 4-allyl-2H-benzo[b][1,4]oxazin-3(4H)-one and 6-chloropyridine-3-sulfonyl chloride, and purified wih chromatography on silica gel (petroleum ether) to afford the title compound **38** in 42% yield as a white solid, 63.0 mg. M.P. 127.6-129.5 °C.

^{1}H NMR (400 MHz, $CDCl_3$) δ 8.23 (d, J = 2.5 Hz, 1H), 7.35 (dd, J = 8.3, 2.5 Hz, 1H), 7.31–7.23 (m, 1H), 6.92–6.85 (m, 2H), 6.53–6.47 (m, 1H), 4.67 (s, 2H), 4.21 (dd, J = 7.7, 5.1 Hz, 1H), 3.88 (td, J = 6.6, 4.5 Hz, 2H), 2.38–2.24 (m, 1H), 2.13–2.02 (m, 1H). ^{13}C NMR (101 MHz, $CDCl_3$) δ 163.6, 150.3, 149.8, 144.7, 138.7, 138.5, 126.2, 125.6, 124.5, 123.8, 123.3, 115.8, 67.4, 39.4, 37.2, 29.6.

HRMS (ESI, m/z): calculated for $C_{16}H_{13}ClN_2O_2$ $[M+H]^+$ = 301.0738, found 301.0742.

(S)-7-(3,5-dimethylisoxazol-4-yl)-6,7-dihydro-5H-[1,4]oxazino[2,3,4-i*J*]quinolin-3 (2H)-one (39)

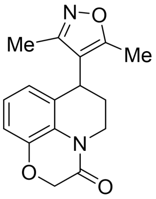

The product was prepared according to the general procedure **C** from 4-allyl-2H-benzo[b][1,4]oxazin-3(4H)-one and 3,5-dimethylisoxazole-4-sulfonyl chloride, and purified wih chromatography on silica gel (petroleum ether) to afford the title compound **39** in 37% yield as a white solid, 52.6 mg. M.P. 118.6-119.9 °C.

^{1}H NMR (400 MHz, $CDCl_3$) δ 6.92–6.85 (m, 2H), 6.59–6.54 (m, 1H), 4.66 (s, 2H), 4.22 (ddd, J = 13.4, 5.7, 3.8 Hz, 1H), 3.99 (dd, J = 9.6, 5.1 Hz, 1H), 3.67 (ddd, J = 13.6, 10.3, 3.4 Hz, 1H), 2.20–1.99 (m, 8H). ^{13}C NMR (101 MHz, $CDCl_3$) δ 166.1, 163.6, 159.2, 144.6, 125.9, 125.4, 123.8, 122.4, 115.6, 115.1, 67.4, 38.4, 31.8, 29.8, 27.8, 11.2 (d, J = 53.4 Hz).

HRMS (ESI, m/z): calculated for $C_{16}H_{16}N_2O_3$ $[M+H]^+$ = 285.1234, found 285.1238.

(S)-7-(3-bromo-4-chlorothiophen-2-yl)-6,7-dihydro-5H-[1,4]oxazino[2,3,4-i*J*]quinolin-3(2H)-one (40)

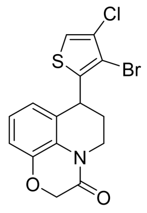

The product was prepared according to the general procedure **C** from 4-allyl-2H-benzo[b][1,4]oxazin-3(4H)-one and 3-bromo-4-chlorothiophene-2-sulfonyl chloride, and purified wih chromatography on silica gel (petroleum ether) to afford the title compound **40** in 67% yield as a white solid, 128.0 mg. M.P. 154.4-156.2 °C.

^{1}H NMR (400 MHz, $CDCl_3$) δ 7.00–6.86 (m, 2H), 6.78 (dd, *J* = 7.1, 2.0 Hz, 1H), 6.52 (d, *J* = 1.1 Hz, 1H), 4.64 (s, 2H), 4.33 (t, *J* = 5.1 Hz, 1H), 4.12 (ddd, *J* = 13.5, 5.7, 4.1 Hz, 1H), 3.63 (ddd, *J* = 13.7, 10.2, 3.8 Hz, 1H), 2.33–2.11 (m, 2H). ^{13}C NMR (101 MHz, $CDCl_3$) δ 163.5, 145.7, 144.6, 127.9, 125.5, 125.3, 125.0, 123.6, 123.6, 116.2, 110.0, 67.3, 37.7, 36.5, 29.1. HRMS (ESI, m/z): calculated for $C_{15}H_{11}BrClNO_2S$ $[M+H]^+$ = 383.9455, found 383.9459.

(S)-7-(5-chlorothiophen-2-yl)-6,7-dihydro-5H-[1,4]oxazino[2,3,4-i*J*]quinolin-3(2H)-one (41)

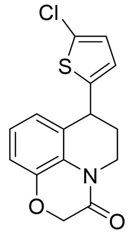

The product was prepared according to the general procedure **C** from 4-allyl-2H-benzo[b][1,4]oxazin-3(4H)-one and 5-chlorothiophene-2-sulfonyl chloride, and purified wih chromatography on silica gel (petroleum ether) to afford the title compound **41** in 67% yield as a white solid, 102.2 mg. M.P. 124.8-126.1 °C.

^{1}H NMR (400 MHz, $CDCl_3$) δ 6.95–6.87 (m, 2H), 6.79 (ddd, *J* = 7.3, 1.9, 0.8 Hz, 1H), 6.73 (d, *J* = 3.7 Hz, 1H), 6.47 (dd, *J* = 3.8, 1.0 Hz, 1H), 4.64 (s, 2H), 4.34 (t, *J* = 5.2 Hz, 1H), 4.07 (ddd, *J* = 13.5, 6.1, 4.1 Hz, 1H), 3.69 (ddd, *J* = 13.6, 9.8, 3.8 Hz, 1H), 2.33–2.13 (m, 2H). ^{13}C

NMR (101 MHz, $CDCl_3$) δ 163.6, 146.1, 144.5, 128.8, 126.4, 125.9, 125.2, 124.9, 123.6, 123.5, 115.9, 67.3, 37.8, 36.8, 29.4.

HRMS (ESI, m/z): calculated for $C_{15}H_{12}ClNO_2S$ $[M+H]^+$ = 306.0350, found 306.0353.

(S)-7-(benzo[b]thiophen-3-yl)-6,7-dihydro-5H-[1,4]oxazino[2,3,4-i*J*]quinolin-3(2H)-one (42)

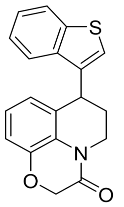

The product was prepared according to the general procedure **C** from 4-allyl-2H-benzo[b][1,4]oxazin-3(4H)-one and benzo[b]thiophene-3-sulfonyl chloride, and purified wih chromatography on silica gel (petroleum ether) to afford the title compound **42** in 63% yield as a white solid, 101.1 mg. M.P. 141.3-143.2 °C.

^{1}H NMR (400 MHz, $CDCl_3$) δ 7.94–7.86 (m, 1H), 7.81–7.74 (m, 1H), 7.41 (tt, J = 7.3, 5.6 Hz, 2H), 6.96–6.85 (m, 2H), 6.77 (s, 1H), 6.73 (dd, J = 6.7, 2.4 Hz, 1H), 4.76–4.65 (m, 2H), 4.63 (t, J = 5.2 Hz, 1H), 4.14 (ddd, J = 13.3, 6.0, 3.9 Hz, 1H), 3.57 (ddd, J = 13.4, 9.9, 3.6 Hz, 1H), 2.48–2.12 (m, 2H). ^{13}C NMR (101 MHz, $CDCl_3$) δ 163.5, 144.4, 141.0, 138.5, 137.5, 127.0, 125.3, 124.8, 124.6, 124.2, 123.7, 123.6, 123.3, 121.7, 115.3, 67.3, 36.6, 36.4, 26.6.

HRMS (ESI, m/z): calculated for $C_{19}H_{15}NO_2S$ $[M+H]^+$ = 322.0896, found 322.0895.

(R)-7-(2-oxo-2H-chromen-6-yl)-6,7-dihydro-5H-[1,4]oxazino[2,3,4-i*J*]quinolin-3(2H)-one (43)

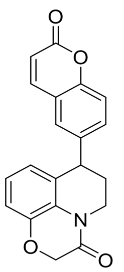

The product was prepared according to the general procedure **C** from 4-allyl-2H-benzo[b][1,4]oxazin-3(4H)-one and 2-oxo-2H-chromene-6-sulfonyl chloride, and purified wih chromatography on silica gel (petroleum ether) to afford the title compound **43**

in 67% yield as a white solid, 111.6 mg. M.P. 202.3-204.1 °C.

^{1}H NMR (400 MHz, $CDCl_3$) δ 7.64 (d, *J* = 9.5 Hz, 1H), 7.31 (d, *J* = 1.8 Hz, 2H), 7.16 (s, 1H), 6.90–6.87 (m, 2H), 6.59–6.51 (m, 1H), 6.42 (d, *J* = 9.5 Hz, 1H), 4.71–4.63 (m, 2H), 4.25 (dd, *J* = 7.2, 5.0 Hz, 1H), 3.98–3.76 (m, 2H), 2.37–2.05 (m, 2H). ^{13}C NMR (101 MHz, $CDCl_3$) δ 163.5, 160.8, 153.0, 144.5, 143.4, 140.5, 132.0, 127.5, 127.1, 125.5, 123.6, 123.6, 118.9, 117.3, 117.1, 115.5, 67.3, 41.6, 37.0, 29.7.

HRMS (ESI, m/z): calculated for $C_{20}H_{15}NO_4$ $[M+H]^+$ = 334.1074, found 334.1073.

(E)-7-styryl-6,7-dihydro-5H-[1,4]oxazino[2,3,4-i*J*]quinolin-3(2H)-one (44)

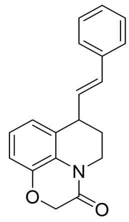

The product was prepared according to the general procedure **C** from 4-allyl-2H-benzo[b][1,4]oxazin-3(4H)-one and (E)-2-phenylethene-1-sulfonyl chloride, and purified wih chromatography on silica gel (petroleum ether) to afford the title compound **44** in 57% yield as a white solid, 82.9 mg. M.P. 101.3-102.5 °C.

^{1}H NMR (400 MHz, $CDCl_3$) δ 7.41–7.27 (m, 4H), 7.26–7.21 (m, 1H), 7.00–6.82 (m, 3H), 6.41 (d, *J* = 15.9 Hz, 1H), 6.23 (dd, *J* = 15.8, 7.8 Hz, 1H), 4.64 (s, 2H), 3.94 (ddd, *J* = 13.2, 8.0, 3.8 Hz, 2H), 3.70 (td, *J* = 7.4, 5.0 Hz, 1H), 2.19–1.93 (m, 2H). ^{13}C NMR (101 MHz, $CDCl_3$) δ 163.6, 144.5, 136.8, 132.4, 131.7, 128.7, 128.7, 127.7, 127.3, 126.4, 126.4, 124.8, 123.4, 123.3, 115.2, 67.4, 39.9, 37.4, 27.4.

HRMS (ESI, m/z): calculated for $C_{19}H_{17}NO_2$ $[M+H]^+$ = 292.1332, found 292.1334.

9-methoxy-7-(p-tolyl)-6,7-dihydro-5H-[1,4]oxazino[2,3,4-i*J*]quinolin-3(2H)-one (45)

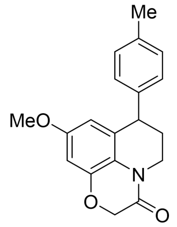

The product was prepared according to the general procedure **C** from 4-allyl-7-methoxy-2H-benzo[b][1,4]oxazin-3(4H)-one and 4-methylbenzenesulfonyl chloride, and purified with chromatography on silica gel (petroleum ether) to afford the title compound **45** in 77% yield as a white solid, 119.0 mg. M.P. 132.6-134.2 °C.

^{1}H NMR (400 MHz, $CDCl_3$) δ 7.12 (d, J = 7.8 Hz, 2H), 6.99 (d, J = 8.0 Hz, 2H), 6.46 (d, J = 2.7 Hz, 1H), 6.14 (d, J = 2.7 Hz, 1H), 4.64 (s, 2H), 4.10 (dd, J = 7.0, 5.1 Hz, 1H), 3.90–3.76 (m, 2H), 3.65 (s, 3H), 2.33 (s, 3H), 2.28–2.04 (m, 2H). ^{13}C NMR (101 MHz, $CDCl_3$) δ 162.8, 155.8, 145.3, 140.8, 136.6, 129.4, 129.4, 129.1, 128.3, 128.3, 119.0, 108.8, 101.6, 67.6, 55.6, 42.3, 37.1, 29.9, 21.1.

HRMS (ESI, m/z): calculated for $C_{19}H_{19}NO_3$ $[M+H]^+$ = 310.1438, found 310.1442.

8-methyl-7-(p-tolyl)-6,7-dihydro-5H-[1,4]oxazino[2,3,4-i*J*]quinolin-3(2H)-one (46)

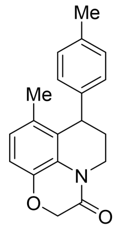

The product was prepared according to the general procedure **C** from 4-allyl-6-methyl-2H-benzo[b][1,4]oxazin-3(4H)-one and 4-methylbenzenesulfonyl chloride, and purified with chromatography on silica gel (petroleum ether) to afford the title compound **46** in 63% yield as a white solid, 95.3 mg. M.P. 72.6-77.9 °C.

^{1}H NMR (400 MHz, $CDCl_3$) δ 7.08 (d, J = 7.8 Hz, 2H), 6.90–6.77 (m, 4H), 4.71–4.57 (m, 2H), 4.45–4.36 (m, 1H), 4.25 (s, 1H), 3.01 (td, J = 12.8, 4.4 Hz, 1H), 2.31 (s, 3H), 2.15 (dq, J = 11.0, 3.8 Hz, 2H), 1.99 (s, 3H). ^{13}C NMR (101 MHz, $CDCl_3$) δ 164.1, 142.7, 140.1, 136.3, 131.2, 129.4, 129.4, 128.0, 128.0, 125.7, 125.4, 125.1, 114.9, 67.4, 38.7, 34.6, 29.0, 21.1, 18.9.

HRMS (ESI, m/z): calculated for $C_{19}H_{19}NO_2$ $[M+H]^+$ = 294.1489, found 294.1490.

9-fluoro-7-(p-tolyl)-6,7-dihydro-5H-[1,4]oxazino[2,3,4-i*J*]quinolin-3(2H)-one (47)

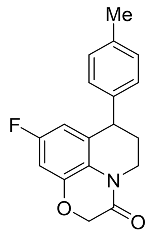

The product was prepared according to the general procedure **C** from 4-allyl-7-fluoro-2H-benzo[b][1,4]oxazin-3(4H)-one and 4-methylbenzenesulfonyl chloride, and purified with chromatography on silica gel (petroleum ether) to afford the title compound **47** in 72% yield as a white solid, 107.0 mg. M.P. 97.5-99.2 °C.

^{1}H NMR (400 MHz, $CDCl_3$) δ 7.14 (d, *J* = 7.8 Hz, 2H), 6.98 (d, *J* = 8.1 Hz, 2H), 6.60 (dd, *J* = 8.9, 2.8 Hz, 1H), 6.30 (dd, *J* = 9.1, 2.8 Hz, 1H), 4.67 (s, 2H), 4.08 (dd, *J* = 7.5, 5.0 Hz, 1H), 3.96–3.77 (m, 2H), 2.34 (s, 3H), 2.30–1.98 (m, 2H). ^{19}F NMR (376 MHz, $CDCl_3$) δ -117.85 (t, *J* = 9.0 Hz). ^{13}C NMR (101 MHz, $CDCl_3$) δ 162.9, 158.6, 145.3, 140.3, 136.9, 129.7, 129.6, 129.6, 128.3, 128.3, 121.7, 110.0, 103.2, 67.5, 42.3, 37.4, 29.7, 21.1.

HRMS (ESI, m/z): calculated for $C_{18}H_{16}FNO_2$ $[M+H]^+$ = 298.1238, found 298.1242.

1-(p-tolyl)-2,3,6,7-tetrahydro-1H,5H-pyrido[3,2,1-i*J*]quinolin-5-one (48)

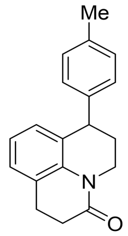

The product was prepared according to the general procedure **C** from 1-allyl-3,4-dihydroquinolin-2(1H)-one and 4-methylbenzenesulfonyl chloride, and purified with chromatography on silica gel (petroleum ether) to afford the title compound **48** in 81% yield as a white solid, 112.2 mg. M.P. 101.2-103.2 °C.

^{1}H NMR (400 MHz, $CDCl_3$) δ 7.12 (d, *J* = 7.8 Hz, 2H), 7.04 (d, *J* = 7.3 Hz, 1H), 6.97 (d, *J* = 7.9 Hz, 2H), 6.87 (t, *J* = 7.5 Hz, 1H), 6.79 (d, *J* = 7.7 Hz, 1H), 4.11 (dd, *J* = 7.2, 5.1 Hz, 1H), 3.87 (t, *J* = 5.9 Hz, 2H), 2.94 (t, *J* = 7.5 Hz, 2H), 2.70 (dd, *J* = 9.2, 6.6 Hz, 2H), 2.33 (s, 3H), 2.24–2.01 (m, 2H). ^{13}C NMR (101 MHz, $CDCl_3$) δ 169.8, 141.6, 136.4, 136.4, 129.4, 129.4, 128.8, 128.5, 128.5, 127.9, 126.3, 125.4, 122.6, 42.7, 38.4, 31.6, 30.2, 25.5, 21.2.

HRMS (ESI, m/z): calculated for $C_{19}H_{19}NO$ $[M+H]^+$ = 278.1540, found 278.1542.

9-methoxy-1-(p-tolyl)-2,3,6,7-tetrahydro-1H,5H-pyrido[3,2,1-i*J*]quinolin-5-one (49)

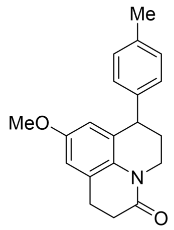

The product was prepared according to the general procedure **C** from 1-allyl-6-methoxy-3,4-dihydroquinolin-2(1H)-one and 4-methylbenzenesulfonyl chloride, and purified with chromatography on silica gel (petroleum ether) to afford the title compound **49** in 78% yield as a white solid, 119.8 mg. M.P. 88.2-89.6 °C.

^{1}H NMR (400 MHz, $CDCl_3$) δ 7.11 (d, *J* = 7.8 Hz, 2H), 6.98 (d, *J* = 7.8 Hz, 2H), 6.62 (d, *J* = 2.8 Hz, 1H), 6.34 (d, *J* = 2.8 Hz, 1H), 4.08 (dd, *J* = 7.0, 5.1 Hz, 1H), 3.84 (td, *J* = 7.0, 4.4 Hz, 2H), 3.66 (d, *J* = 0.9 Hz, 3H), 2.91 (t, *J* = 7.5 Hz, 2H), 2.68 (dd, *J* = 8.9, 6.5 Hz, 2H), 2.33 (s, 3H), 2.24–1.99 (m, 2H). ^{13}C NMR (101 MHz, $CDCl_3$) δ 169.1, 155.0, 141.4, 136.3, 130.0, 129.4, 129.4, 129.0, 128.4, 128.4, 126.9, 113.2, 112.6, 55.5, 42.9, 38.2, 31.6, 30.4, 25.8, 21.1. HRMS (ESI, m/z): calculated for $C_{20}H_{21}NO_2$ $[M+H]^+$ = 308.1645, found 308.1640.

9-((tert-butyldimethylsilyl)oxy)-1-(p-tolyl)-2,3,6,7-tetrahydro-1H,5H-pyrido[3,2,1-ij]quinolin-5-one (50)

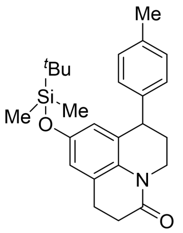

The product was prepared according to the general procedure **C** from 1-allyl-6-((tert-butyldimethylsilyl)oxy)-3,4-dihydroquinolin-2(1H)-one and 4-methylbenzenesulfonyl chloride, and purified with chromatography on silica gel (petroleum ether) to afford the title compound **50** in 72% yield as a white solid, 146.6 mg. M.P. 157.2-159.4 °C.

^{1}H NMR (400 MHz, $CDCl_3$) δ 7.11 (d, J = 7.7 Hz, 2H), 6.97 (d, J = 8.1 Hz, 2H), 6.54 (d, J = 2.9 Hz, 1H), 6.25 (d, J = 2.9 Hz, 1H), 4.04 (dd, J = 7.3, 5.0 Hz, 1H), 3.83 (t, J = 5.9 Hz, 2H), 2.87 (t, J = 7.4 Hz, 2H), 2.70–2.62 (m, 2H), 2.33 (s, 3H), 2.26–2.13 (m, 1H), 2.10–1.97 (m, 1H), 0.90 (s, 9H), 0.07 (s, 6H). ^{13}C NMR (101 MHz, $CDCl_3$) δ 169.2, 150.8, 141.5, 136.3, 130.4, 129.3, 129.3, 129.0, 128.4, 128.4, 126.7, 119.5, 119.5, 118.2, 118.2, 42.7, 38.3, 31.6, 30.4, 25.8, 25.8, 25.6, 21.1, 18.2, -4.4 (d, J = 7.5 Hz).

HRMS (ESI, m/z): calculated for $C_{25}H_{33}NO_2Si$ $[M+H]^+$ = 408.2353, found 408.2355.

5-oxo-1-(p-tolyl)-2,3,6,7-tetrahydro-1H,5H-pyrido[3,2,1-ij]quinolin-9-yl benzoate (51)

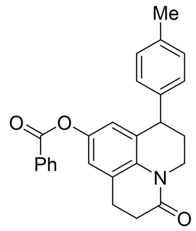

The product was prepared according to the general procedure **C** from 1-allyl-2-oxo-1,2,3,4-tetrahydroquinolin-6-yl benzoate and 4-methylbenzenesulfonyl chloride, and purified with chromatography on silica gel (petroleum ether) to afford the title compound **51** in 68% yield as a white solid, 135.0 mg. M.P. 147.3-148.3 °C.

^{1}H NMR (400 MHz, $CDCl_3$) δ 8.12 (d, J = 7.1 Hz, 2H), 7.60 (t, J = 7.4 Hz, 1H), 7.47 (t, J = 7.8 Hz, 2H), 7.13 (d, J = 8.0 Hz, 2H), 7.01 (d, J = 8.0 Hz, 2H), 6.94 (d, J = 2.6 Hz, 1H), 6.63 (d, J = 2.7 Hz, 1H), 4.12 (t, J = 6.2 Hz, 1H), 3.90 (ddd, J = 12.9, 7.7, 3.8 Hz, 2H), 2.97 (t, J = 7.5 Hz, 2H), 2.73 (dd, J = 8.9, 6.1 Hz, 2H), 2.32 (s, 3H), 2.28–2.01 (m, 2H). ^{13}C NMR (101 MHz, $CDCl_3$) δ 169.5, 165.5, 145.8, 141.0, 136.5, 134.3, 133.7, 130.2, 130.2, 129.5, 129.5, 129.5, 129.3, 128.7, 128.7, 128.4, 128.4, 126.8, 121.4, 119.8, 42.9, 38.5, 31.3, 30.3, 25.6, 21.1.

HRMS (ESI, m/z): calculated for $C_{26}H_{23}NO_3$ $[M+H]^+$ = 398.1751, found 398.1755.

9-bromo-7,7-dimethyl-1-(p-tolyl)-2,3,6,7-tetrahydro-1H,5H-pyrido[3,2,1-iJ]quinolin-5-one (52)

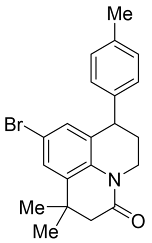

The product was prepared according to the general procedure **C** from 1-allyl-6-bromo-4,4-dimethyl-3,4-dihydroquinolin-2(1H)-one and 4-methylbenzenesulfonyl chloride, and purified with chromatography on silica gel (petroleum ether) to afford the title compound **52** in 71% yield as a white solid, 136.0 mg. M.P. 138.6-140.1 °C.

^{1}H NMR (400 MHz, $CDCl_3$) δ 7.28 (d, *J* = 2.3 Hz, 1H), 7.13 (d, *J* = 7.8 Hz, 2H), 7.01–6.89 (m, 3H), 4.13–4.05 (m, 1H), 3.96–3.79 (m, 2H), 2.61–2.47 (m, 2H), 2.34 (s, 3H), 2.24–1.99 (m, 2H), 1.35 (s, 3H), 1.29 (s, 3H). ^{13}C NMR (101 MHz, $CDCl_3$) δ 168.5, 140.8, 136.6, 136.4, 134.3, 131.3, 130.2, 129.5, 129.5, 128.3, 128.3, 126.0, 116.0, 45.5, 43.0, 38.2, 33.4, 30.0, 27.6, 27.5, 21.1.

HRMS (ESI, m/z): calculated for $C_{21}H_{22}BrNO$ $[M+H]^+$ = 384.0958, found 384.0955.

9-fluoro-1-(p-tolyl)-2,3,6,7-tetrahydro-1H,5H-pyrido[3,2,1-iJ]quinolin-5-one (53)

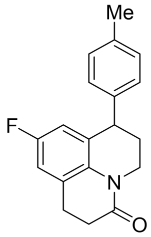

The product was prepared according to the general procedure **C** from 1-allyl-6-fluoro-3,4-dihydroquinolin-2(1H)-one and 4-methylbenzenesulfonyl chloride, and purified with chromatography on silica gel (petroleum ether) to afford the title compound **53** in 62% yield as a white solid, 91.5 mg. M.P. 117.5-119.2 °C.

^{1}H NMR (400 MHz, $CDCl_3$) δ 7.13 (d, *J* = 7.8 Hz, 2H), 6.97 (d, *J* = 7.8 Hz, 2H), 6.76 (dd, *J* = 8.2, 2.9 Hz, 1H), 6.48 (dd, *J* = 9.3, 2.9 Hz, 1H), 4.06 (dd, *J* = 7.8, 5.2 Hz, 1H), 3.87 (ddd, *J* = 12.9, 8.0, 3.8 Hz, 2H), 2.98–2.84 (m, 2H), 2.69 (dd, *J* = 9.0, 6.4 Hz, 2H), 2.34 (s, 3H), 2.26–2.00 (m, 2H). ^{19}F NMR (376 MHz, $CDCl_3$) δ -120.59. ^{13}C NMR (101 MHz, $CDCl_3$) δ 169.3, 158.7, 140.9, 136.7, 132.5, 129.9, 129.5, 129.5, 128.4, 128.4, 127.4, 114.7, 113.4, 43.0, 38.5, 31.3, 30.2, 25.6, 21.1.

HRMS (ESI, m/z): calculated for $C_{19}H_{18}FNO$ $[M+H]^+$ = 296.1445, found 296.1450.

9-chloro-1-(p-tolyl)-2,3,6,7-tetrahydro-1H,5H-pyrido[3,2,1-iJ]quinolin-5-one (54)

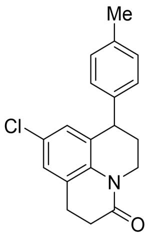

The product was prepared according to the general procedure **C** from 1-allyl-6-chloro-3,4-dihydroquinolin-2(1H)-one and 4-methylbenzenesulfonyl chloride, and purified with chromatography on silica gel (petroleum ether) to afford the title compound **54** in 63% yield as a white solid, 98.0 mg. M.P. 92.2-93.5 °C.

^{1}H NMR (400 MHz, $CDCl_3$) δ 7.13 (d, *J* = 7.8 Hz, 2H), 7.02 (d, *J* = 2.4 Hz, 1H), 6.96 (d, *J* = 8.0 Hz, 2H), 6.77 (d, *J* = 2.4 Hz, 1H), 4.06 (dd, *J* = 7.3, 5.1 Hz, 1H), 3.85 (t, *J* = 5.9 Hz, 2H), 2.96–2.85 (m, 2H), 2.76–2.62 (m, 2H), 2.34 (s, 3H), 2.23–1.99 (m, 2H). ^{13}C NMR (101 MHz, $CDCl_3$) δ 169.4, 140.8, 136.7, 135.1, 129.6, 129.5, 129.5, 128.4, 128.4, 128.3, 127.6, 127.2, 126.3, 42.7, 38.4, 31.3, 30.0, 25.4, 21.2.

HRMS (ESI, m/z): calculated for $C_{19}H_{18}ClNO$ $[M+H]^+$ = 312.1150, found 312.1152.

9-bromo-1-(p-tolyl)-2,3,6,7-tetrahydro-1H,5H-pyrido[3,2,1-iJ]quinolin-5-one (55)

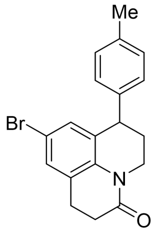

The product was prepared according to the general procedure **C** from 1-allyl-6-bromo-3,4-dihydroquinolin-2(1H)-one and 4-methylbenzenesulfonyl chloride, and purified with chromatography on silica gel (petroleum ether) to afford the title compound **55** in 90% yield as a white solid, 159.8 mg. M.P. 87.3-88.4 °C.

^{1}H NMR (400 MHz, $CDCl_3$) δ 7.17 (d, *J* = 2.2 Hz, 1H), 7.13 (d, *J* = 7.8 Hz, 2H), 6.98–6.90 (m, 3H), 4.12–4.04 (m, 1H), 3.84 (pt, *J* = 7.6, 4.3 Hz, 2H), 2.92 (t, *J* = 7.5 Hz, 2H), 2.7–2.65 (m, 2H), 2.34 (s, 3H), 2.24–1.96 (m, 2H). ^{13}C NMR (101 MHz, $CDCl_3$) δ 169.3, 140.7, 136.7,

135.6, 131.2, 129.9, 129.5, 129.5, 129.1, 128.3, 128.3, 127.5, 115.3, 42.6, 38.3, 31.3, 30.0, 25.3, 21.1.

HRMS (ESI, m/z): calculated for $C_{19}H_{18}BrNO$ $[M+H]^+$ = 356.0645, found 356.0643.

8-methyl-1-(p-tolyl)-2,3,6,7-tetrahydro-1H,5H-pyrido[3,2,1-ij]quinolin-5-one (56)

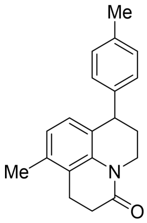

The product was prepared according to the general procedure **C** from 1-allyl-5-methyl-3,4-dihydroquinolin-2(1H)-one and 4-methylbenzenesulfonyl chloride, and purified with chromatography on silica gel (petroleum ether) to afford the title compound **56** in 68% yield as a white solid, 99.0 mg. M.P. 93.5-94.7 °C.

^{1}H NMR (400 MHz, $CDCl_3$) δ 7.11 (d, J = 7.6 Hz, 2H), 7.00–6.96 (m, 2H), 6.77 (d, J = 7.9 Hz, 1H), 6.69 (d, J = 7.9 Hz, 1H), 4.09 (t, J = 6.4 Hz, 1H), 3.94–3.79 (m, 2H), 2.87 (p, J = 8.0 Hz, 2H), 2.68 (t, J = 7.6 Hz, 2H), 2.33 (s, 3H), 2.28 (s, 3H), 2.22–2.00 (m, 2H). ^{13}C NMR (101 MHz, $CDCl_3$) δ 169.7, 141.9, 136.5, 136.3, 133.9, 129.3, 129.3, 128.5, 128.5, 128.3, 125.8, 124.8, 123.9, 42.7, 38.5, 31.1, 30.5, 21.8, 21.1, 19.6.

HRMS (ESI, m/z): calculated for $C_{20}H_{21}NO$ $[M+H]^+$ = 292.1696, found 292.1690.

7-(p-tolyl)-6,7-dihydro-5H-[1,4]thiazino[2,3,4-iJ]quinolin-3(2H)-one (57)

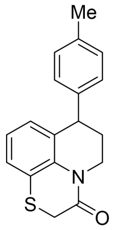

The product was prepared according to the general procedure **C** from 4-allyl-2H-benzo[b][1,4]thiazin-3(4H)-one and 4-methylbenzenesulfonyl chloride, and purified with chromatography on silica gel (petroleum ether) to afford the title compound **57** in 58% yield as a yellow solid, 85.6 mg. M.P. 90.2-91.4 °C.

^{1}H NMR (400 MHz, $CDCl_3$) δ 7.23 (dd, *J* = 7.8, 1.4 Hz, 1H), 7.12 (d, *J* = 7.8 Hz, 2H), 6.98 (d, *J* = 8.0 Hz, 2H), 6.89 (t, *J* = 7.7 Hz, 1H), 6.80 (d, *J* = 7.5 Hz, 1H), 4.17–4.10 (m, 1H), 4.05–3.81 (m, 2H), 3.51–3.42 (m, 2H), 2.33 (s, 3H), 2.30–2.03 (m, 2H). ^{13}C NMR (101 MHz, $CDCl_3$) δ 164.7, 141.3, 136.5, 136.1, 130.4, 129.5, 129.5, 128.7, 128.3, 128.3, 126.8, 123.2, 121.9, 43.0, 39.7, 31.0, 30.9, 21.1.

HRMS (ESI, m/z): calculated for $C_{18}H_{17}NOS$ $[M+H]^+$ = 296.1104, found 296.1101.

9-methyl-7-(p-tolyl)-6,7-dihydro-5H-[1,4]thiazino[2,3,4-iJ]quinolin-3(2H)-one (58)

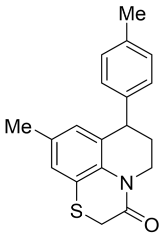

The product was prepared according to the general procedure **C** from 4-allyl-7-methyl-2H-benzo[b][1,4]thiazin-3(4H)-one and 4-methylbenzenesulfonyl chloride, and purified with chromatography on silica gel (petroleum ether) to afford the title compound **58** in 61% yield as a yellow solid, 94.3 mg. M.P. 93.4-94.1 °C.

^{1}H NMR (400 MHz, $CDCl_3$) δ 7.12 (d, *J* = 7.0 Hz, 2H), 7.05 (s, 1H), 6.97 (d, *J* = 6.5 Hz, 2H), 6.65–6.56 (m, 1H), 4.10 (t, *J* = 6.4 Hz, 1H), 3.98–3.81 (m, 2H), 3.52–3.38 (m, 2H), 2.34 (s, 3H), 2.28–2.21 (m, 1H), 2.17 (s, 3H), 2.12–2.00 (m, 1H). ^{13}C NMR (101 MHz, $CDCl_3$) δ 164.5, 141.4, 136.5, 133.8, 133.0, 130.0, 129.5, 129.4, 128.3, 128.3, 127.3, 119.3, 42.9, 39.4, 31.2, 31.0, 29.8, 21.2, 20.6.

HRMS (ESI, m/z): calculated for $C_{19}H_{19}NOS$ $[M+H]^+$ = 310.1260, found 310.1262.

9-bromo-7-(p-tolyl)-6,7-dihydro-5H-[1,4]thiazino[2,3,4-iJ]quinolin-3(2H)-one (59)

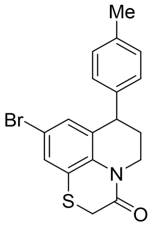

The product was prepared according to the general procedure **C** from 4-allyl-7-bromo-2H-benzo[b][1,4]thiazin-3(4H)-one and 4-methylbenzenesulfonyl chloride, and purified with chromatography on silica gel (petroleum ether) to afford the title compound **59** in 72% yield as a yellow solid, 134.3 mg. M.P. 119.2-121.1 °C.

^{1}H NMR (400 MHz, $CDCl_3$) δ 7.37 (s, 1H), 7.14 (d, *J* = 7.6 Hz, 2H), 7.00–6.90 (m, 3H), 4.09 (t, *J* = 6.6 Hz, 1H), 3.91 (ddd, *J* = 13.1, 7.9, 4.0 Hz, 2H), 3.51–3.40 (m, 2H), 2.34 (s, 3H), 2.27–2.01 (m, 2H). ^{13}C NMR (101 MHz, $CDCl_3$) δ 164.2, 140.3, 136.9, 135.2, 132.2, 131.2, 129.7, 129.7, 129.2, 128.2, 128.2, 124.0, 115.8, 43.0, 39.7, 30.7, 27.0, 21.2.

HRMS (ESI, m/z): calculated for $C_{18}H_{16}BrNOS$ $[M+H]^+$ = 374.0209, found 374.0205.

1,1-dimethyl-6-(p-tolyl)-5,6-dihydro-4H-pyrrolo[3,2,1-iJ]quinolin-2(1H)-one (60)

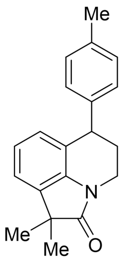

The product was prepared according to the general procedure **C** from 1-allyl-3,3-dimethylindolin-2-one and 4-methylbenzenesulfonyl chloride, and purified with chromatography on silica gel (petroleum ether) to afford the title compound **60** in 41% yield as a white solid, 59.7 mg. M.P. 102.4-103.7 °C.

^{1}H NMR (400 MHz, $CDCl_3$) δ 7.14 (d, *J* = 8.0 Hz, 2H), 7.08 (dt, *J* = 7.4, 1.0 Hz, 1H), 7.03 (dt, *J* = 8.2, 2.0 Hz, 2H), 6.91 (td, *J* = 7.6, 1.9 Hz, 1H), 6.76 (dt, *J* = 7.8, 1.0 Hz, 1H), 4.13 (dd, *J* = 8.0, 4.7 Hz, 1H), 3.82–3.62 (m, 2H), 2.35 (s, 3H), 2.30–2.06 (m, 2H), 1.42 (dd, *J* = 11.4, 1.9 Hz, 6H). ^{13}C NMR (126 MHz, $CDCl_3$) δ 180.4, 140.4, 138.7, 136.6, 134.4, 129.5, 129.5, 128.4, 128.4, 126.9, 123.0, 122.2, 120.6, 45.8, 41.3, 37.2, 30.5, 24.5, 24.4, 21.2.

HRMS (ESI, m/z): calculated for $C_{20}H_{21}NO$ $[M+H]^+$ = 292.1696, found 292.1692.

1-(p-tolyl)-2,3-dihydroisoindolo[7,1,2-hiJ]quinolin-5(1H)-one (61)

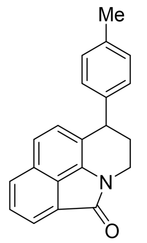

The product was prepared according to the general procedure **C** from 1-allylbenzo[cd]indol-2(1H)-one and 4-methylbenzenesulfonyl chloride, and purified with chromatography on silica gel (petroleum ether) to afford the title compound **61** in 64% yield as a yellow solid, 95.7 mg. M.P. 132.7-133.8 °C.

^{1}H NMR (400 MHz, $CDCl_3$) δ 8.07 (d, *J* = 7.0 Hz, 1H), 7.99 (d, *J* = 8.1 Hz, 1H), 7.70 (dd, *J* = 8.1, 7.0 Hz, 1H), 7.43 (d, *J* = 8.6 Hz, 1H), 7.15 (d, *J* = 7.6 Hz, 2H), 7.07 (dd, *J* = 8.3, 5.9 Hz, 3H), 4.25 (dd, *J* = 7.3, 4.8 Hz, 1H), 3.93 (dt, *J* = 7.3, 4.7 Hz, 2H), 2.47 (ddt, *J* = 13.7, 6.4, 4.7 Hz, 1H), 2.35 (s, 3H), 2.30–2.21 (m, 1H). ^{13}C NMR (101 MHz, $CDCl_3$) δ 166.9, 139.9, 136.8, 135.7, 130.6, 129.6, 128.8, 128.5, 128.4, 128.4, 128.4, 128.1, 128.0, 124.9, 123.5, 120.1, 119.2, 40.6, 37.1, 33.1, 21.2.

HRMS (ESI, m/z): calculated for $C_{21}H_{17}NO$ $[M+H]^+$ = 300.1383, found 300.1385.

1-(p-tolyl)-2,3-dihydro-1H-4-thia-3a-azacyclopenta[def]phenanthrene 4,4-dioxide (62)

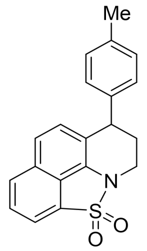

The product was prepared according to the general procedure **C** from 2-allyl-2H-naphtho[1,8-cd]isothiazole 1,1-dioxide and 4-methylbenzenesulfonyl chloride, and purified with chromatography on silica gel (petroleum ether) to afford the title compound **62** in 68% yield as a yellow solid, 114.0 mg. M.P. 121.7-123.4 °C.

^{1}H NMR (400 MHz, $CDCl_3$) δ 8.04 (d, *J* = 8.2 Hz, 1H), 7.96 (d, *J* = 7.2 Hz, 1H), 7.73 (dd, *J* = 8.2, 7.3 Hz, 1H), 7.34 (d, *J* = 8.7 Hz, 1H), 7.13 (t, *J* = 8.1 Hz, 3H), 7.05–7.00 (m, 2H), 4.27–4.19 (m, 1H), 3.82 (dd, *J* = 6.5, 4.9 Hz, 2H), 2.53 (dq, *J* = 13.6, 5.4 Hz, 1H), 2.34 (s,

4H). ^{13}C NMR (101 MHz, $CDCl_3$) δ 140.5, 136.9, 132.7, 131.5, 131.1, 130.1, 129.6, 129.6, 129.6, 128.4, 128.4, 128.0, 120.2, 118.6, 118.0, 117.6, 40.2, 37.4, 31.8, 21.2.

HRMS (ESI, m/z): calculated for $C_{20}H_{17}NO_2S$ $[M+H]^+$ = 336.1053, found 336.1055.

1-(p-tolyl)-2,3,7,8-tetrahydro-1H-azepino[3,2,1-iJ]quinolin-5(6H)-one (63)

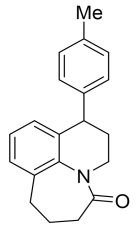

The product was prepared according to the general procedure **C** from 1-allyl-1,3,4,5-tetrahydro-2H-benzo[b]azepin-2-one and 4-methylbenzenesulfonyl chloride, and purified with chromatography on silica gel (petroleum ether) to afford the title compound **63** in 39% yield as a white solid, 56.8 mg. M.P. 122.3-124.1 °C.

^{1}H NMR (400 MHz, $CDCl_3$) δ 7.13 (d, J = 7.8 Hz, 2H), 7.08 (d, J = 7.5 Hz, 1H), 7.04–6.96 (m, 3H), 6.77 (d, J = 7.7 Hz, 1H), 3.95 (d, J = 32.9 Hz, 3H), 2.75 (t, J = 7.2 Hz, 2H), 2.42 (td, J = 7.1, 4.8 Hz, 2H), 2.34 (s, 3H), 2.24 (d, J = 5.9 Hz, 3H), 2.08 (dq, J = 14.6, 7.8 Hz, 1H). ^{13}C NMR (101 MHz, $CDCl_3$) δ 173.2, 140.3, 138.8, 136.4, 134.0, 132.6, 129.5, 129.5, 129.5, 129.5, 128.2, 127.8, 124.9, 43.1, 41.4, 34.4, 32.2, 30.6, 29.7, 21.2.

HRMS (ESI, m/z): calculated for $C_{20}H_{21}NO$ $[M+H]^+$ = 292.1696, found 292.1697.

6-methyl-7-(p-tolyl)-6,7-dihydro-5H-[1,4]oxazino[2,3,4-iJ]quinolin-3(2H)-one (64)

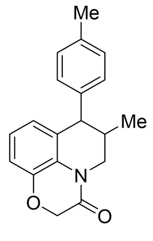

The product was prepared according to the general procedure **C** from 4-(2-methylallyl)-2H-benzo[b][1,4]oxazin-3(4H)-one and 4-methylbenzenesulfonyl chloride, and purified with chromatography on silica gel (petroleum ether) to afford the title compound **64** in 58% yield as a white solid, 85.0 mg. M.P. 96.5-99.1 °C.

^{1}H NMR (400 MHz, $CDCl_3$) δ 7.13 (d, *J* = 7.8 Hz, 2H), 6.97 (d, *J* = 8.1 Hz, 2H), 6.82–6.80 (m, 2H), 6.44 (ddt, *J* = 6.1, 3.1, 1.0 Hz, 1H), 4.67 (d, *J* = 3.9 Hz, 2H), 4.25 (dd, *J* = 13.2, 3.7 Hz, 1H), 3.64 (d, *J* = 8.6 Hz, 1H), 3.30 (dd, *J* = 13.2, 9.4 Hz, 1H), 2.35 (s, 3H), 2.17 (tdd, *J* = 9.9, 5.1, 3.1 Hz, 1H), 0.96 (dd, *J* = 6.7, 1.0 Hz, 3H). ^{13}C NMR (101 MHz, $CDCl_3$) δ 163.7, 144.3, 140.4, 136.6, 129.4, 129.4, 129.0, 129.0, 128.8, 125.1, 124.2, 123.5, 114.7, 67.4, 50.3, 44.1, 33.9, 21.2, 17.7.

HRMS (ESI, m/z): calculated for $C_{19}H_{19}NO_2$ $[M+H]^+$ = 294.1489, found 294.1489.

Methyl(7R)-3-oxo-7-(p-tolyl)-2,3,6,7-tetrahydro-5H-[1,4]oxazino[2,3,4-ij]quinoline-6-carboxylate (65)

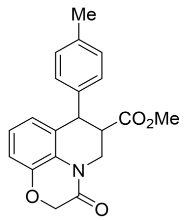

The product was prepared according to the general procedure **C** from methyl 2-((3-oxo-2,3-dihydro-4H-benzo[b][1,4]oxazin-4-yl)methyl)acrylate and 4-methylbenzenesulfonyl chloride, and purified with chromatography on silica gel (petroleum ether) to afford the title compound **65** in 58% yield as a white solid, 97.8 mg. M.P. 107.3-108.6 °C.

^{1}H NMR (400 MHz, $CDCl_3$) δ 7.11 (d, *J* = 7.7 Hz, 2H), 6.98 (d, *J* = 7.7 Hz, 2H), 6.86 (d, *J* = 4.3 Hz, 2H), 6.58 (t, *J* = 4.6 Hz, 1H), 4.66 (s, 2H), 4.50 (d, *J* = 7.0 Hz, 1H), 4.07 (d, *J* = 5.7 Hz, 2H), 3.60 (s, 3H), 3.05 (q, *J* = 6.1 Hz, 1H), 2.33 (s, 3H). ^{13}C NMR (101 MHz, $CDCl_3$) δ 172.1, 163.6, 144.3, 139.3, 137.1, 129.6, 129.6, 128.6, 128.6, 126.6, 124.9, 124.0, 123.7, 115.2, 67.4, 52.4, 45.6, 44.5, 38.4, 21.2.

HRMS (ESI, m/z): calculated for $C_{20}H_{19}NO_4$ $[M+H]^+$ = 338.1387, found 338.1392.

3-(p-tolyl)-2,3-dihydro-1H-pyrido[3,2,1-kl]phenoxazine (66)

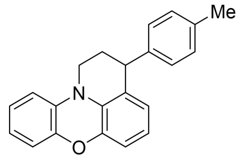

The product was prepared according to the general procedure **C** from 10-allyl-10H-phenoxazine--methane (1/1) and 4-methylbenzenesulfonyl chloride, and purified with chromatography on silica gel (petroleum ether) to afford the title compound **66** in 52% yield as a yellow solid, 81.4 mg. M.P. 69.7-71.3 °C.

^{1}H NMR (400 MHz, $CDCl_3$) δ 7.11 (d, J = 7.8 Hz, 2H), 7.01 (d, J = 7.8 Hz, 2H), 6.87–6.76 (m, 1H), 6.70 (dt, J = 4.7, 1.2 Hz, 2H), 6.60–6.51 (m, 2H), 6.47–6.38 (m, 2H), 4.02 (t, J = 5.3 Hz, 1H), 3.36–3.08 (m, 2H), 2.32 (s, 4H), 2.19 (ddd, J = 13.3, 6.8, 4.1 Hz, 1H). ^{13}C NMR (101 MHz, $CDCl_3$) δ 145.0, 144.2, 141.7, 136.2, 134.3, 130.7, 129.3, 129.3, 128.4, 128.4, 124.8, 124.7, 123.8, 121.0, 120.3, 115.2, 113.9, 110.4, 41.3, 39.7, 29.4, 21.1.

HRMS (ESI, m/z): calculated for $C_{22}H_{19}NO$ $[M+H]^+$ = 314.1540, found 314.1540.

1-(3-(p-tolyl)-2,3-dihydro-1H-pyrido[3,2,1-kl]phenothiazin-10-yl)ethan-1-one (67)

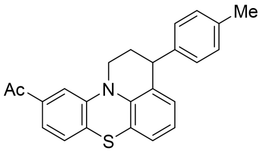

The product was prepared according to the general procedure **C** from 1-(10-allyl-10H-phenothiazin-2-yl)ethan-1-one and 4-methylbenzenesulfonyl chloride, and purified with chromatography on silica gel (petroleum ether) to afford the title compound **67** in 53% yield as a white solid, 98.3 mg. M.P. 63.6-65.2 °C.

^{1}H NMR (400 MHz, $CDCl_3$) δ 7.09 (dd, J = 12.4, 7.0 Hz, 4H), 7.04 (d, J = 7.7 Hz, 2H), 6.95 (t, J = 7.5 Hz, 1H), 6.86 (d, J = 7.8 Hz, 2H), 6.77 (d, J = 8.3 Hz, 1H), 4.90 (t, J = 4.3 Hz, 1H), 3.53–3.42 (m, 2H), 2.41–2.33 (m, 1H), 2.29 (s, 3H), 2.18 (s, 4H). ^{13}C NMR (101 MHz, $CDCl_3$) δ 202.5, 144.1, 143.0, 141.9, 138.2, 135.8, 129.2, 129.2, 128.3, 128.3, 127.9, 127.0, 125.4, 125.1, 125.0, 123.1, 122.5, 122.0, 113.8, 41.8, 38.1, 30.1, 29.4, 21.1.

HRMS (ESI, m/z): calculated for $C_{24}H_{21}NOS$ $[M+H]^+$ = 372.1417, found 372.1419.

4-(p-tolyl)chromane (68)

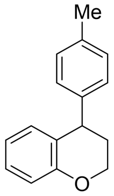

The product was prepared according to the general procedure **C** from (allyloxy)benzene and 4-methylbenzenesulfonyl chloride, and purified with chromatography on silica gel (petroleum ether) to afford the title compound **68** in 71% yield as a colorless oil, 79.6 mg.

^{1}H NMR (400 MHz, $CDCl_3$) δ 7.19 (dd, *J* = 8.2, 2.4 Hz, 3H), 7.11 (dd, *J* = 8.2, 2.4 Hz, 2H), 6.99–6.90 (m, 2H), 6.90–6.83 (m, 1H), 4.37–4.06 (m, 3H), 2.41 (d, *J* = 2.4 Hz, 3H), 2.38–2.33 (m, 1H), 2.19–2.10 (m, 1H). ^{13}C NMR (101 MHz, $CDCl_3$) δ 155.2, 142.8, 136.1, 130.7, 129.2, 129.2, 128.6, 128.6, 127.9, 124.9, 120.4, 116.8, 64.0, 40.7, 31.8, 21.1.

HRMS (ESI, m/z): calculated for $C_{16}H_{16}O$ $[M+H]^+$ = 225.1274, found 225.1275.

6-methyl-4-(p-tolyl)chromane (69)

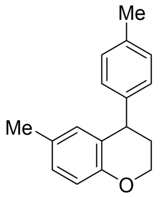

The product was prepared according to the general procedure **C** from 1-(allyloxy)-4-methylbenzene and 4-methylbenzenesulfonyl chloride, and purified with chromatography on silica gel (petroleum ether) to afford the title compound **69** in 62% yield as a colorless oil, 73.9 mg.

^{1}H NMR (400 MHz, $CDCl_3$) δ 7.16 (d, *J* = 7.7 Hz, 2H), 7.08 (d, *J* = 8.0 Hz, 2H), 6.97 (dd, *J* = 8.5, 2.2 Hz, 1H), 6.81 (d, *J* = 8.3 Hz, 1H), 6.70 (d, *J* = 2.2 Hz, 1H), 4.19 (ddd, *J* = 5.8, 3.9, 1.3 Hz, 2H), 4.15 (t, *J* = 6.3 Hz, 1H), 2.38 (s, 3H), 2.31 (ddd, *J* = 12.6, 6.6, 4.3 Hz, 1H), 2.20 (s, 3H), 2.12–2.04 (m, 1H). ^{13}C NMR (101 MHz, $CDCl_3$) δ 153.1, 143.0, 136.1, 130.9, 129.5, 129.2, 129.2, 128.7, 128.7, 128.6, 124.4, 116.6, 63.8, 40.7, 32.0, 21.1, 20.6.

HRMS (ESI, m/z): calculated for $C_{17}H_{18}O$ $[M+H]^+$ = 239.1431, found 239.1432.

6-(sec-butyl)-4-(p-tolyl)chromane (70)

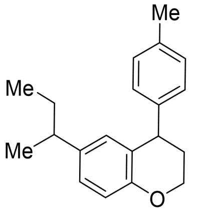

The product was prepared according to the general procedure **C** from 1-(allyloxy)-4-(sec-butyl)benzene and 4-methylbenzenesulfonyl chloride, and purified with chromatography on silica gel (petroleum ether) to afford the title compound **70** in 72% yield as a colorless oil, 90.1 mg.

^{1}H NMR (400 MHz, $CDCl_3$) δ 7.13 (d, J = 7.7 Hz, 2H), 7.04 (d, J = 7.9 Hz, 2H), 6.97 (dt, J = 8.4, 2.7 Hz, 1H), 6.82 (dd, J = 8.4, 2.5 Hz, 1H), 6.67 (d, J = 2.2 Hz, 1H), 4.20–4.11 (m, 3H), 2.42 (p, J = 7.2 Hz, 1H), 2.35 (s, 3H), 2.33–2.26 (m, 1H), 2.10–1.98 (m, 1H), 1.47 (td, J = 7.4, 5.6 Hz, 2H), 1.13 (dd, J = 7.0, 2.1 Hz, 3H), 0.76 (t, J = 7.4 Hz, 3H). ^{13}C NMR (101 MHz, $CDCl_3$) δ 153.3, 143.1, 139.5, 136.0, 129.5, 129.2, 129.2, 128.7, 128.7, 126.3, 123.9, 116.5, 63.7, 40.8, 40.7, 32.1, 31.4, 22.0, 21.1, 12.3.

HRMS (ESI, m/z): calculated for $C_{20}H_{24}O$ $[M+H]^+$ = 281.1900, found 281.1903.

8-methyl-4-(p-tolyl)chromane (71)

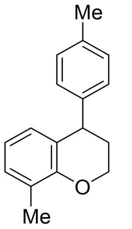

The product was prepared according to the general procedure **C** from 1-(allyloxy)-2-methylbenzene and 4-methylbenzenesulfonyl chloride, and purified with chromatography on silica gel (petroleum ether) to afford the title compound **71** in 64% yield as a colorless oil, 76.2 mg.

^{1}H NMR (400 MHz, $CDCl_3$) δ 7.16 (d, J = 8.0 Hz, 2H), 7.10–7.02 (m, 3H), 6.79–6.70 (m, 2H), 4.25 (ddd, J = 5.8, 4.1, 1.2 Hz, 2H), 4.19 (t, J = 6.4 Hz, 1H), 2.37 (s, 3H), 2.33 (ddt, J = 8.9, 4.5, 1.4 Hz, 1H), 2.30–2.27 (m, 3H), 2.16–2.02 (m, 1H). ^{13}C NMR (101 MHz, $CDCl_3$) δ 153.4, 143.1, 136.0, 129.2, 129.2, 129.0, 128.7, 128.7, 128.4, 125.9, 124.2, 119.7, 64.0, 40.9, 31.9, 21.1, 16.3.

HRMS (ESI, m/z): calculated for $C_{17}H_{18}O$ $[M+H]^+$ = 239.1431, found 239.1432.

4-(4-methoxyphenyl)chromane-5,6,7,8-d4 (72)

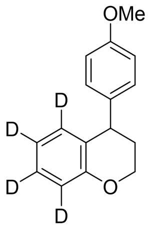

The product was prepared according to the general procedure **C** from 1-(allyloxy)benzene-2,3,4,5,6-d_5 and 4-methoxybenzenesulfonyl chloride, and purified with chromatography on silica gel (petroleum ether) to afford the title compound **72** in 57% yield as a colorless oil, 69.6 mg.

^{1}H NMR (400 MHz, $CDCl_3$) δ 7.06 (d, *J* = 8.5 Hz, 2H), 6.87–6.82 (m, 2H), 4.22–4.16 (m, 2H), 4.13 (t, *J* = 6.4 Hz, 1H), 3.79 (d, *J* = 1.0 Hz, 3H), 2.32–2.23 (m, 1H), 2.08–2.00 (m, 1H). ^{13}C NMR (101 MHz, $CDCl_3$) δ 158.3, 151.1, 143.5, 137.8, 130.0, 129.7, 129.7, 125.0, 122.3, 113.9, 113.9, 109.3, 64.1, 55.4, 40.3, 31.9.

HRMS (ESI, m/z): calculated for $C_{16}H_{12}D_4O_2$ $[M+H]^+$ = 245.1474, found 245.1471.

6-methoxy-4-(p-tolyl)chromane (73)

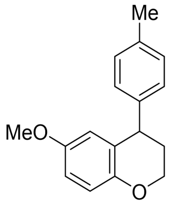

The product was prepared according to the general procedure **C** from 1-(allyloxy)-4-methoxybenzene and 4-methylbenzenesulfonyl chloride, and purified with chromatography on silica gel (petroleum ether) to afford the title compound **73** in 53% yield as a colorless oil, 67.3 mg.

^{1}H NMR (400 MHz, $CDCl_3$) δ 7.13–7.11 (m, 2H), 7.04 (d, *J* = 8.1 Hz, 2H), 6.80 (d, *J* = 8.8 Hz, 1H), 6.72 (dd, *J* = 8.9, 3.1 Hz, 1H), 6.38 (d, *J* = 3.0 Hz, 1H), 4.16–4.10 (m, 3H), 3.64 (d, *J* = 0.7 Hz, 3H), 2.34 (s, 3H), 2.32–2.28 (m, 1H), 2.08–2.01 (m, 1H). ^{13}C NMR (101 MHz,

$CDCl_3$) δ 153.3, 149.4, 142.6, 136.2, 129.3, 129.3, 128.7, 128.7, 125.3, 117.4, 115.0, 114.2, 63.9, 55.8, 41.0, 32.0, 21.2.

HRMS (ESI, m/z): calculated for $C_{17}H_{18}O_2$ $[M+H]^+$ = 255.1380, found 255.1380.

6-ethoxy-4-(*p*-tolyl)chromane (74)

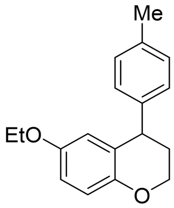

The product was prepared according to the general procedure **C** from 1-(allyloxy)-4-ethoxybenzene and 4-methylbenzenesulfonyl chloride, and purified with chromatography on silica gel (petroleum ether) to afford the title compound **74** in 67% yield as a colorless oil, 89.8 mg.

^{1}H NMR (500 MHz, $CDCl_3$) δ 7.11 (d, *J* = 7.7 Hz, 2H), 7.03 (d, *J* = 7.8 Hz, 2H), 6.78 (d, *J* = 8.9 Hz, 1H), 6.71 (dd, *J* = 8.9, 3.0 Hz, 1H), 6.38 (d, *J* = 2.9 Hz, 1H), 4.19–4.07 (m, 3H), 3.84 (qd, *J* = 7.0, 3.3 Hz, 2H), 2.33 (s, 3H), 2.27 (dtd, *J* = 13.0, 6.5, 3.5 Hz, 1H), 2.05 (dtd, *J* = 13.8, 6.9, 3.4 Hz, 1H), 1.62 (m, 3H). ^{13}C NMR (126 MHz, $CDCl_3$) δ 152.7, 149.4, 142.7, 136.2, 129.3, 129.3, 128.7, 128.7, 125.4, 117.4, 116.0, 114.9, 64.0, 64.0, 41.1, 32.1, 21.1, 15.0.

HRMS (ESI, m/z): calculated for $C_{18}H_{20}O_2$ $[M+H]^+$ = 269.1536, found 269.1536.

6-iodo-4-(p-tolyl)chromane (75)

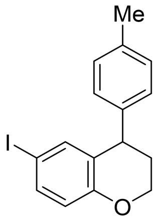

The product was prepared according to the general procedure **C** from 1-(allyloxy)-4-iodobenzene and 4-methylbenzenesulfonyl chloride, and purified with chromatography on silica gel (petroleum ether) to afford the title compound **75** in 57 % yield as a colorless oil, 99.8 mg.

^{1}H NMR (400 MHz, $CDCl_3$) δ 7.44 (dd, *J* = 8.6, 2.2 Hz, 1H), 7.22–7.15 (m, 3H), 7.05 (d, *J* = 8.1 Hz, 2H), 6.70 (d, *J* = 8.6 Hz, 1H), 4.25–4.11 (m, 3H), 2.40 (s, 3H), 2.37–2.24 (m, 1H), 2.16–2.02 (m, 1H). ^{13}C NMR (101 MHz, $CDCl_3$) δ 155.1, 141.9, 139.0, 136.6, 136.4, 129.4, 129.4, 128.5, 128.5, 127.5, 119.2, 82.5, 63.9, 40.3, 31.3, 21.1.
HRMS (ESI, m/z): calculated for $C_{16}H_{15}IO$ $[M+H]^+$ = 351.0241, found 351.0242.

4-(p-tolyl)-6-(trifluoromethyl)chromane (76)

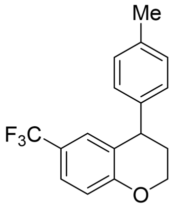

The product was prepared according to the general procedure **C** from 1-(allyloxy)-4-(trifluoromethyl)benzene and 4-methylbenzenesulfonyl chloride, and purified with chromatography on silica gel (petroleum ether) to afford the title compound **76** in 51% yield as a colorless oil, 74.5 mg.
^{1}H NMR (400 MHz, $CDCl_3$) δ 7.41 (d, *J* = 8.7 Hz, 1H), 7.17 (d, *J* = 8.0 Hz, 3H), 7.03 (d, *J* = 8.1 Hz, 2H), 6.97 (d, *J* = 8.6 Hz, 1H), 4.25 (t, *J* = 5.5 Hz, 2H), 4.19 (t, *J* = 6.3 Hz, 1H), 2.37 (s, 3H), 2.36–2.32 (m, 1H), 2.17–2.07 (m, 1H). ^{19}F NMR (376 MHz, $CDCl_3$) δ -61.15. ^{13}C NMR (101 MHz, $CDCl_3$) δ 157.9, 141.7, 136.6, 129.5, 129.5, 128.5, 129.5, 128.5, 128.5, 128.1, 128.0, 125.1, 117.3, 64.2, 40.5, 31.3, 21.1.
HRMS (ESI, m/z): calculated for $C_{17}H_{15}F_3O$ $[M+H]^+$ = 293.1148, found 293.1143.

3-methyl-4-(*p*-tolyl)chromane (77)

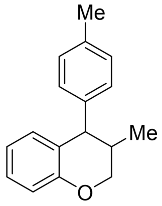

The product was prepared according to the general procedure **C** from 3-methyl-4-(*p*-tolyl)chromane and 4-methylbenzenesulfonyl chloride, and purified with chromatography on silica gel (petroleum ether) to afford the title compound **77** in 59% yield as a colorless oil, 70.3 mg.

^{1}H NMR (400 MHz, $CDCl_3$) δ 7.19–7.14 (m, 3H), 7.08–7.05 (m, 2H), 6.91 (dd, J = 8.1, 1.2 Hz, 1H), 6.84–6.76 (m, 2H), 4.27 (dd, J = 10.8, 3.4 Hz, 1H), 3.88 (dd, J = 10.8, 9.3 Hz, 1H), 3.68 (d, J = 8.9 Hz, 1H), 2.40 (s, 3H), 2.29–2.20 (m, 1H), 0.98 (d, J = 6.7 Hz, 3H). ^{13}C NMR (101 MHz, $CDCl_3$) δ 154.9, 141.5, 136.2, 130.8, 129.2, 129.2, 129.1, 129.1, 127.6, 125.5, 120.5, 116.5, 70.6, 49.4, 35.3, 21.2, 16.0.

HRMS (ESI, m/z): calculated for $C_{17}H_{18}O$ $[M+H]^+$ = 239.1430, found 239.1431.

4-(p-tolyl)-3,4,6,7,8,9-hexahydro-2H-benzo[g]chromene (78)

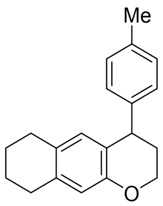

The product was prepared according to the general procedure **C** from 6-(allyloxy)-1,2,3,4-tetrahydronaphthalene and 4-methylbenzenesulfonyl chloride, and purified with chromatography on silica gel (petroleum ether) to afford the title compound **78** in 56% yield as a colorless oil, 77.9 mg.

^{1}H NMR (400 MHz, $CDCl_3$) δ 7.16–7.09 (m, 4H), 6.75 (d, J = 8.3 Hz, 1H), 6.60 (dd, J = 17.9, 5.3 Hz, 1H), 4.17–4.13 (m, 2H), 3.93 (ddd, J = 12.8, 10.8, 2.0 Hz, 1H), 2.73 (d, J = 5.6 Hz, 2H), 2.59–2.49 (m, 2H), 2.35 (s, 3H), 2.32–2.24 (m, 1H), 2.11–2.05 (m, 1H), 1.68–1.59 (m, 4H). ^{13}C NMR (101 MHz, $CDCl_3$) δ 153.0, 142.5, 136.6, 135.8, 130.8, 129.2, 129.2, 128.7, 128.3, 128.3, 121.2, 114.4, 63.9, 40.5, 37.0, 29.3, 28.6, 23.2, 23.0, 21.1.

HRMS (ESI, m/z): calculated for $C_{20}H_{22}O$ $[M+H]^+$ = 279.1744, found 279.1741.

4-(p-tolyl)-3,4,7,8,9,10-hexahydro-2H-benzo[h]chromene (79)

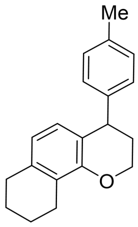

The product was prepared according to the general procedure **C** from 5-(allyloxy)-1,2,3,4-tetrahydronaphthalene and 4-methylbenzenesulfonyl chloride, and

purified with chromatography on silica gel (petroleum ether) to afford the title compound **79** in 61% yield as a colorless oil, 80.3 mg.

^{1}H NMR (400 MHz, $CDCl_3$) δ 7.16 (d, *J* = 7.6 Hz, 2H), 7.10 (d, *J* = 7.9 Hz, 2H), 6.66–6.57 (m, 2H), 4.24 (ddd, *J* = 6.5, 3.8, 2.3 Hz, 2H), 4.16 (t, *J* = 6.5 Hz, 1H), 2.74 (dd, *J* = 22.7, 6.1 Hz, 4H), 2.38 (s, 3H), 2.35–2.29 (m, 1H), 2.14–2.05 (m, 1H), 1.83 (ddd, *J* = 13.1, 6.7, 3.5 Hz, 4H). ^{13}C NMR (101 MHz, $CDCl_3$) δ 152.8, 143.2, 136.7, 136.0, 129.2, 129.2, 128.7, 128.7, 127.3, 125.2, 121.1, 120.8, 64.0, 40.8, 32.0, 29.6, 23.3, 23.0, 23.0, 21.1.

HRMS (ESI, m/z): calculated for $C_{20}H_{22}O$ $[M+H]^+$ = 279.1744, found 279.1745.

12-(p-tolyl)-11,12-dihydro-10H-phenanthro[4,5-fgh]chromene (80)

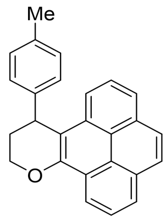

The product was prepared according to the general procedure **C** from 4-(allyloxy)pyrene and 4-methylbenzenesulfonyl chloride, and purified with chromatography on silica gel (petroleum ether) to afford the title compound **80** in 69% yield as a white solid, 120.1 mg. M.P. 138.6-140.1 °C.

^{1}H NMR (400 MHz, CDCl3) δ 8.46 (td, *J* = 6.5, 3.3 Hz, 1H), 8.16–8.03 (m, 3H), 7.98–7.91 (m, 1H), 7.87–7.74 (m, 2H), 7.68 (s, 1H), 7.16 (d, *J* = 8.1 Hz, 2H), 7.09 (d, *J* = 8.2 Hz, 2H), 4.59 (t, *J* = 6.2 Hz, 1H), 4.52 (t, *J* = 5.2 Hz, 2H), 2.59–2.48 (m, 1H), 2.38 (d, *J* = 3.0 Hz, 3H), 2.35–2.24 (m, 1H). ^{13}C NMR (101 MHz, $CDCl_3$) δ 149.6, 143.2, 136.3, 131.7, 131.6, 129.3, 129.3, 128.9, 128.9, 127.2, 127.0, 126.5, 126.0, 125.0, 125.0, 124.9, 124.8, 124.2, 124.2, 122.4, 121.2, 119.7, 64.5, 41.3, 31.8, 21.2.

HRMS (ESI, m/z): calculated for $C_{26}H_{20}O$ $[M+H]^+$ = 349.1587, found 349.1587.

6-(p-tolyl)-7,8-dihydro-2H,6H-pyrano[3,2-g]chromen-2-one (81)

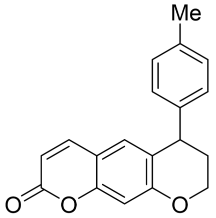

The product was prepared according to the general procedure **C** from 7-(allyloxy)-2H-chromen-2-one and 4-methylbenzenesulfonyl chloride, and purified with chromatography on silica gel (petroleum ether) to afford the title compound **81** in 71% yield as a white solid, 103.7 mg. M.P. 115.1-116.5 °C.

^{1}H NMR (400 MHz, $CDCl_3$) δ 7.45 (d, *J* = 9.5 Hz, 1H), 7.15 (d, *J* = 7.8 Hz, 2H), 7.02 (d, *J* = 8.0 Hz, 2H), 6.93 (s, 1H), 6.81 (s, 1H), 6.17 (d, *J* = 9.5 Hz, 1H), 4.27 (ddd, *J* = 6.8, 3.9, 2.3 Hz, 2H), 4.16 (t, *J* = 6.6 Hz, 1H), 2.35 (s, 3H), 2.36–2.05 (m, 2H). ^{13}C NMR (101 MHz, $CDCl_3$) δ 161.5, 158.5, 154.2, 143.5, 141.6, 136.8, 129.7, 129.6, 129.6, 128.5, 128.5, 122.7, 113.2, 112.9, 104.3, 64.9, 40.4, 31.2, 21.2.

HRMS (ESI, m/z): calculated for $C_{19}H_{16}O_3$ $[M+H]^+$ = 293.1172, found 293.1176.

6-(p-tolyl)-4-(trifluoromethyl)-7,8-dihydro-2H,6H-pyrano[3,2-g]chromen-2-one (82)

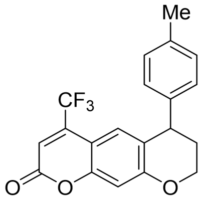

The product was prepared according to the general procedure **C** from 7-(allyloxy)-4-(trifluoromethyl)-2H-chromen-2-one and 4-methylbenzenesulfonyl chloride, and purified with chromatography on silica gel (petroleum ether) to afford the title compound **82** in 68% yield as a white solid, 122.5 mg. M.P. 89.6-90.7 °C.

^{1}H NMR (400 MHz, $CDCl_3$) δ 7.6 (d, *J* = 9.0 Hz, 1H), 7.3 (d, *J* = 7.8 Hz, 2H), 7.2 (d, *J* = 7.8 Hz, 2H), 6.9 (dd, *J* = 9.0, 1.5 Hz, 1H), 6.9 (d, *J* = 1.7 Hz, 1H), 5.2 (dd, *J* = 9.0, 5.6 Hz, 1H), 4.3 (td, *J* = 8.6, 4.9 Hz, 1H), 4.1 (dt, *J* = 10.7, 5.4 Hz, 1H), 2.7–2.4 (m, 2H), 2.4 (s, 3H). ^{19}F NMR (376 MHz, $CDCl_3$) δ -64.50. ^{13}C NMR (101 MHz, $CDCl_3$) δ 162.7, 159.5, 156.4, 138.8,

13 7.9, 129.7, 129.7, 129.7, 127.0, 127.0, 127.0, 126.5, 113.7, 112.5 (d, *J* = 5.7 Hz), 107.3, 102.2, 65.7, 59.7, 39.1, 21.3.

HRMS (ESI, m/z): calculated for $C_{20}H_{15}F_3O_3$ $[M+H]^+$ = 361.1046, found 361.1044.

4-(*p*-tolyl)-3,4-dihydro-2*H*-benzofuro[2,3-*g*]chromene (83)

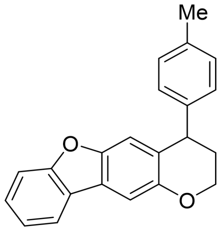

The product was prepared according to the general procedure **C** from 2-(allyloxy)dibenzo[*b,d*]furan and 4-methylbenzenesulfonyl chloride, and purified with chromatography on silica gel (petroleum ether) to afford the title compound **83** in 47% yield as a white solid, 73.8 mg. M.P. 112.6-113.6 °C.

^{1}H NMR (400 MHz, $CDCl_3$) δ 7.62 (ddd, *J* = 18.4, 14.9, 7.7 Hz, 5H), 7.48 (t, *J* = 7.7 Hz, 2H), 7.31 (t, *J* = 8.7 Hz, 2H), 7.21 (s, 1H), 4.90 (d, *J* = 5.6 Hz, 1H), 4.45–4.40 (m, 1H), 4.24 (td, *J* = 11.8, 2.0 Hz, 1H), 2.76–2.62 (m, 1H), 2.46 (s, 3H), 2.25 (d, *J* = 13.6 Hz, 1H). ^{13}C NMR (101 MHz, $CDCl_3$) δ 156.8, 151.0, 150.7, 141.7, 136.2, 129.5, 129.5, 128.5, 128.5, 126.4, 124.2, 123.1, 122.9, 122.3, 117.1, 116.8, 111.5, 111.2, 61.6, 38.0, 31.1, 21.1.

HRMS (ESI, m/z): calculated for $C_{22}H_{18}O_2$ $[M+H]^+$ = 315.1380, found 315.1385.

8-(p-tolyl)-7,8-dihydro-6H-[1,3]dioxolo[4,5-g]chromene (84)

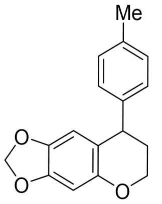

The product was prepared according to the general procedure **C** from 5-(allyloxy)benzo[d][1,3]dioxole and 4-methylbenzenesulfonyl chloride, and purified with chromatography on silica gel (petroleum ether) to afford the title compound **84** in 76% yield as a brown solid, 101.9 mg. M.P. 93.8-94.6 °C.

^{1}H NMR (400 MHz, $CDCl_3$) δ 7.15–7.11 (m, 2H), 7.07–7.03 (m, 2H), 6.42 (d, J = 1.9 Hz, 1H), 6.28 (t, J = 1.0 Hz, 1H), 5.88–5.81 (m, 2H), 4.16–4.08 (m, 2H), 4.04 (t, J = 6.4 Hz, 1H), 2.35 (d, J = 1.8 Hz, 3H), 2.33–2.20 (m, 1H), 2.02 (dtd, J = 13.7, 6.6, 3.9 Hz, 1H). ^{13}C NMR (101 MHz, $CDCl_3$) δ 150.0, 146.9, 142.9, 141.5, 136.2, 129.3, 129.3, 128.6, 128.6, 116.3, 109.2, 100.9, 98.4, 64.0, 40.7, 32.0, 21.1.

HRMS (ESI, m/z): calculated for $C_{17}H_{16}O_3$ $[M+H]^+$ = 269.1172, found 269.1173.

5,6,7-trimethoxy-4-(p-tolyl)chromane (85)

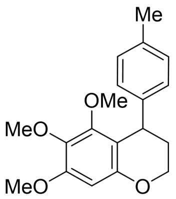

The product was prepared according to the general procedure **C** from 5-(allyloxy)-1,2,3-trimethoxybenzene and 4-methylbenzenesulfonyl chloride, and purified with chromatography on silica gel (petroleum ether) to afford the title compound **85** in 42% yield as a colorless oil, 66.0 mg.

^{1}H NMR (400 MHz, $CDCl_3$) δ 7.08 (d, J = 8.2 Hz, 2H), 7.00 (d, J = 8.1 Hz, 2H), 6.26 (s, 1H), 4.28–4.22 (m, 1H), 4.09 (d, J = 11.0 Hz, 1H), 4.00–3.92 (m, 1H), 3.84 (s, 3H), 3.76 (s, 3H), 3.43 (s, 3H), 2.31 (s, 3H), 2.32–2.19 (m, 2H). ^{13}C NMR (101 MHz, $CDCl_3$) δ 153.1, 151.9, 151.3, 144.0, 136.3, 135.6, 129.0, 129.0, 128.1, 128.1, 109.9, 95.8, 62.0, 61.0, 60.4, 55.9, 34.8, 30.9, 21.1.

HRMS (ESI, m/z): calculated for $C_{19}H_{22}O_4$ $[M+H]^+$ = 315.1591, found 315.1593.

11-(p-tolyl)-10,11-dihydro-5H,9H-benzo[c]pyrano[3,2-g]chromen-5-one (86)

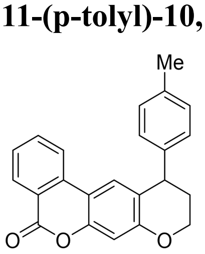

The product was prepared according to the general procedure **C** from 3-(allyloxy)-6H-benzo[c]chromen-6-one and 4-methylbenzenesulfonyl chloride, and purified with chromatography on silica gel (petroleum ether) to afford the title compound **86** in 68 %

yield as a colorless oil, 116.3 mg.

^{1}H NMR (400 MHz, CDCl$_3$) δ 8.32 (dd, *J* = 8.0, 1.4 Hz, 1H), 7.75 (d, *J* = 7.6 Hz, 1H), 7.66 (td, *J* = 7.7, 1.4 Hz, 1H), 7.53 (s, 1H), 7.44 (ddd, *J* = 8.1, 7.2, 1.2 Hz, 1H), 7.16 (d, *J* = 7.9 Hz, 2H), 7.06 (d, *J* = 8.1 Hz, 2H), 6.87 (s, 1H), 4.28–4.22 (m, 3H), 2.36 (s, 4H), 2.17–2.08 (m, 1H). ^{13}C NMR (101 MHz, CDCl$_3$) δ 161.7, 157.2, 151.2, 142.1, 136.6, 135.2, 134.8, 130.6, 129.5, 129.5, 128.6, 128.6, 127.7, 124.8, 122.2, 121.3, 120.1, 111.4, 104.9, 64.3, 40.4, 31.4, 21.2.

HRMS (ESI, m/z): calculated for $C_{23}H_{18}O_3$ [M+H]$^+$ = 343.1329, found 343.1326.

3-(4-methoxyphenyl)-10-(p-tolyl)-9,10-dihydro-4H,8H-pyrano[2,3-f]chromen-4-one (87)

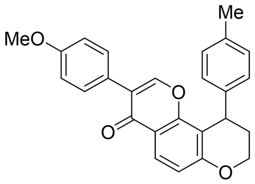

The product was prepared according to the general procedure **C** from 7-(allyloxy)-3-(4-methoxyphenyl)-4*H*-chromen-4-one and 4-methylbenzenesulfonyl chloride, and purified with chromatography on silica gel (petroleum ether) to afford the title compound **87** in 57% yield as a colorless oil, 113.5 mg.

^{1}H NMR (400 MHz, CDCl$_3$) δ 8.16 (d, *J* = 8.9 Hz, 1H), 7.73 (s, 1H), 7.43 (d, *J* = 8.2 Hz, 2H), 7.12 (d, *J* = 7.7 Hz, 2H), 7.00 (d, *J* = 8.3 Hz, 3H), 6.92 (d, *J* = 8.2 Hz, 2H), 4.56 (d, *J* = 5.3 Hz, 1H), 4.29 (d, *J* = 11.1 Hz, 1H), 4.06 (t, *J* = 11.9 Hz, 1H), 3.81 (s, 3H), 2.46–2.32 (m, 2H), 2.33 (s, 3H). ^{13}C NMR (101 MHz, CDCl$_3$) δ 176.2, 159.6, 159.5, 155.5, 152.0, 141.5, 136.3, 130.2, 130.2, 129.3, 129.3, 127.9, 127.9, 125.9, 124.6, 124.4, 118.3, 115.9, 114.0, 114.0, 111.2, 62.4, 55.4, 34.1, 30.0, 21.1.

HRMS (ESI, m/z): calculated for $C_{26}H_{22}O_4$ [M+H]$^+$ = 399.1591, found 399.1593.

4-(9-(4-(p-tolyl)chroman-6-yl)-9H-fluoren-9-yl)phenol (88)

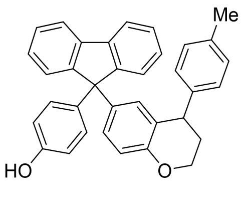

The product was prepared according to the general procedure **C** from 9,9-bis(4-(allyloxy)phenyl)-9H-fluorene and 4-methylbenzenesulfonyl chloride, and purified with chromatography on silica gel (petroleum ether) to afford the title compound **88** in 71 % yield as a colorless oil, 170.5 mg. (The reaction material is 9,9-Bis(4-(allyloxy)phenyl)-9H-fluorene, purchased from Bide Pharmatech Co.,Ltd, and used without further purification.)

^{1}H NMR (400 MHz, $CDCl_3$) δ 7.71 (dd, *J* = 7.7, 3.5 Hz, 2H), 7.36–7.28 (m, 2H), 7.26–7.14 (m, 4H), 7.09 (d, *J* = 7.7 Hz, 2H), 7.00–6.91 (m, 4H), 6.84 (d, *J* = 8.5 Hz, 2H), 6.68 (d, *J* = 8.2 Hz, 1H), 6.58 (d, *J* = 8.8 Hz, 2H), 4.09 (ddd, *J* = 18.5, 8.8, 4.7 Hz, 3H), 2.35 (s, 3H), 2.30–2.24 (m, 1H), 2.08–2.00 (m, 1H). ^{13}C NMR (101 MHz, $CDCl_3$) δ 154.1, 151.9, 142.7, 142.7, 140.0, 138.5, 138.5, 137.5, 135.9, 131.2, 129.3, 129.3, 129.1, 129.1, 128.6, 128.6, 127.7, 127.7, 127.3, 127.2, 126.1, 126.1, 126.1, 126.1, 124.3, 120.1, 120.1, 116.3, 115.0, 115.0, 64.2, 63.9, 40.5, 31.8, 21.2.

HRMS (ESI, m/z): calculated for $C_{35}H_{28}O_2$ $[M+H]^+$ = 481.2162, found 481.2166.

6-((4-isopropoxyphenyl)sulfonyl)-4-(p-tolyl)chromane (89)

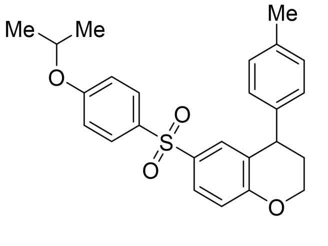

The product was prepared according to the general procedure **C** from 1-(allyloxy)-4-((4-isopropoxyphenyl)sulfonyl)benzene and 4-methylbenzenesulfonyl chloride, and purified with chromatography on silica gel (petroleum ether) to afford the title compound **89** in 61% yield as a yellow oil, 128.8 mg.

^{1}H NMR (400 MHz, $CDCl_3$) δ 7.73–7.68 (m, 2H), 7.63 (dd, *J* = 8.7, 2.4 Hz, 1H), 7.50–7.46 (m, 1H), 7.11 (d, *J* = 7.8 Hz, 2H), 6.99–6.83 (m, 5H), 4.59 (hept, *J* = 6.0 Hz, 1H), 4.27–4.07 (m, 3H), 2.34 (s, 3H), 2.31–2.21 (m, 1H), 2.12–1.98 (m, 1H), 1.33 (dd, *J* = 6.1, 0.7 Hz, 6H). ^{13}C NMR (101 MHz, $CDCl_3$) δ 161.6, 159.1, 141.2, 136.7, 133.6, 133.6, 130.7, 130.7, 129.5, 129.5, 129.5, 128.4, 127.5, 127.5, 125.7, 117.9, 117.9, 115.7, 70.5, 64.6, 40.6, 31.1, 21.9, 21.9, 21.1.

HRMS (ESI, m/z): calculated for $C_{25}H_{26}O_4S$ $[M+H]^+$ = 423.1625, found 423.1626.

(3aR,5aS,13aS,13bS)-3a-methyl-11-(p-tolyl)-1,3a,4,5,5a,6,9,10,11,13,13a,13b-dodecahydrocyclopenta[7,8]naphtho[2,3-g]chromen-3(2H)-one (90)

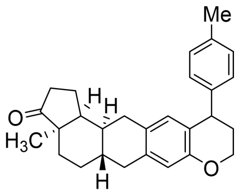

The product was prepared according to the general procedure **C** from (3aR,5aS,11aS,11bS)-8-(allyloxy)-3a-methyl-1,2,3a,4,5,5a,6,11,11a,11b-decahydro-3H-cyclopenta[a]anthracen-3-one and 4-methylbenzenesulfonyl chloride, and purified with chromatography on silica gel (petroleum ether) to afford the title compound **90** in 63% yield as a white solid, 126.1 mg. M.P. 132.2-133.7 **°C**.

^{1}H NMR (400 MHz, $CDCl_3$) δ 7.16–7.06 (m, 3H), 7.06–7.02 (m, 1H), 7.00–6.93 (m, 1H), 6.79 (d, J = 7.0 Hz, 1H), 4.13 (q, J = 7.4 Hz, 3H), 2.89 (d, J = 5.3 Hz, 1H), 2.53–2.44 (m, 1H), 2.34 (s, 3H), 2.14 (t, J = 6.9 Hz, 2H), 2.11–1.92 (m, 5H), 1.69 (s, 1H), 1.59 (dt, J = 16.8, 9.4 Hz, 2H), 1.46 (t, J = 14.0 Hz, 3H), 1.38 (d, J = 8.8 Hz, 1H), 1.28 (d, J = 7.1 Hz, 1H), 0.88 (s, 3H). ^{13}C NMR (101 MHz, $CDCl_3$) δ 221.3, 153.3, 143.0, 136.4, 136.0, 129.3, 129.3, 129.3, 128.6, 128.6, 128.6, 125.2, 114.5, 63.8, 50.5, 48.1, 44.0, 40.6, 38.5, 37.8, 36.0, 31.7, 29.4, 26.7, 26.0, 21.7, 21.2, 14.0.

HRMS (ESI, m/z): calculated for $C_{28}H_{32}O_2$ $[M+H]^+$ = 401.2475, found 401.2477.

7-(4-bromophenyl)-13a-methyl-1,2,3,3a,3b,4,5,7,8,9,11b,12,13,13a-tetradecahydrocyclopenta[5,6]naphtho[2,1-*g*]chromen-1-yl pentanoate (91)

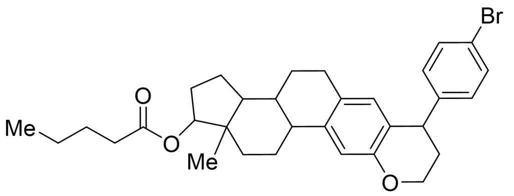

The product was prepared according to the general procedure **C** from 2-(allyloxy)-13-methyl-7,8,9,11,12,13,14,15,16,17-decahydro-6*H*-cyclopenta[*a*]phenanthren-17-yl pentanoate and 4-bromobenzenesulfonyl chloride, and purified with chromatography on

silica gel (petroleum ether) to afford the title compound **91** in 51% yield as a white solid, 140.3 mg. M.P. 143.2-144.5 °C.

^{1}H NMR (400 MHz, CDCl$_3$) δ 7.40 (d, *J* = 7.7 Hz, 2H), 7.03 (d, *J* = 7.8 Hz, 2H), 6.71 (s, 1H), 6.59 (s, 1H), 4.66 (td, *J* = 8.4, 4.0 Hz, 1H), 4.13–4.04 (m, 3H), 2.88–2.78 (m, 2H), 2.30 (dt, *J* = 7.4, 3.6 Hz, 3H), 2.22–2.19 (m, 1H), 2.01–1.97 (m, 1H), 1.91–1.84 (m, 2H), 1.77–1.71 (m, 2H), 1.63–1.59 (m, 4H), 1.37–1.32 (m, 5H), 1.32–1.27 (m, 3H), 0.94–0.92 (m, 3H), 0.79 (s, 3H). ^{13}C NMR (101 MHz, CDCl$_3$) δ 174.1, 153.0, 144.6, 137.0, 131.6, 131.6, 130.5, 130.5, 130.2, 127.4, 125.5, 121.1, 114.4, 82.5, 63.7, 49.9, 44.3, 44.0, 40.6, 37.3, 37.1, 36.9, 34.5, 27.3, 27.2, 26.4, 26.2, 23.4, 23.3, 22.4, 13.9, 12.2.

HRMS (ESI, m/z): calculated for $C_{32}H_{39}BrO_3$ [M+H]$^+$ = 551.2155, found 551.2159.

1-(p-tolyl)-9-(4-(trifluoromethyl)phenyl)-2,3,6,7-tetrahydro-1H,5H-pyrido[3,2,1-ij]quinolin-5-one (92)

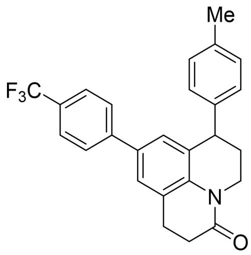

^{1}H NMR (400 MHz, CDCl$_3$) δ 7.61 (d, *J* = 8.1 Hz, 2H), 7.54 (d, *J* = 8.1 Hz, 2H), 7.30 (s, 1H), 7.13 (d, *J* = 7.7 Hz, 2H), 7.06 (s, 1H), 7.01 (d, *J* = 7.0 Hz, 2H), 4.20 (t, *J* = 6.0 Hz, 1H), 3.89 (ddd, *J* = 12.4, 7.5, 3.6 Hz, 2H), 3.03 (t, *J* = 7.5 Hz, 2H), 2.81–2.72 (m, 2H), 2.34 (s, 3H), 2.27–2.09 (m, 2H). ^{19}F NMR (376 MHz, CDCl$_3$) δ -62.21. ^{13}C NMR (101 MHz, CDCl$_3$) δ 169.6, 143.9, 141.2, 136.6, 136.5, 133.9, 129.5, 129.5, 129.2, 128.9, 128.4, 128.4, 127.5, 127.5, 127.0, 127.0, 126.1, 125.8, 125.7, 125.1, 42.8, 38.4, 31.5, 30.2, 25.7, 21.1.

HRMS (ESI, m/z): calculated for $C_{26}H_{22}F_3NO$ [M+H]$^+$ = 422.1726, found 422.1727.

9-hydroxy-1-(p-tolyl)-2,3,6,7-tetrahydro-1H,5H-pyrido[3,2,1-ij]quinolin-5-one (93)

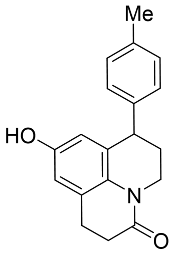

^{1}H NMR (400 MHz, DMSO-D_6) δ 8.96 (s, 1H), 7.08 (d, J = 7.8 Hz, 2H), 6.96 (d, J = 7.8 Hz, 2H), 6.45 (d, J = 2.7 Hz, 1H), 6.03 (d, J = 2.7 Hz, 1H), 4.04 – 3.94 (m, 1H), 3.70 (ddd, J = 13.1, 7.4, 3.8 Hz, 1H), 3.56 (ddd, J = 12.7, 8.3, 3.7 Hz, 1H), 2.74 (t, J = 7.4 Hz, 2H), 2.50 – 2.46 (m, 2H), 2.23 (s, 3H), 2.03 (ddt, J = 12.5, 8.2, 4.3 Hz, 1H), 1.87 (dtd, J = 12.3, 8.0, 3.8 Hz, 1H). ^{13}C NMR (101 MHz, DMSO-D_6) δ 167.9, 152.2, 141.7, 135.5, 129.1, 128.8, 128.8, 128.3, 128.2, 128.2, 126.7, 113.8, 113.4, 41.9, 37.9, 31.1, 29.9, 24.9, 20.6.

HRMS (ESI, m/z): calculated for $C_{19}H_{19}NO_2$ $[M+H]^+$ = 294.1489, found 294.1485.

9-(4-(1-cyclohexyl-1H-tetrazol-5-yl)butoxy)-1-(p-tolyl)-2,3,6,7-tetrahydro-1H,5H-pyrido[3,2,1-ij]quinolin-5-one (94)

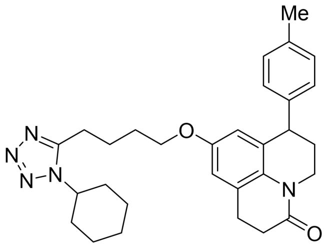

^{1}H NMR (400 MHz, $CDCl_3$) δ 7.08 (d, J = 7.6 Hz, 2H), 6.94 (d, J = 7.7 Hz, 2H), 6.58 (d, J = 2.9 Hz, 1H), 6.29 (d, J = 2.8 Hz, 1H), 4.07 (dq, J = 23.5, 5.9 Hz, 2H), 3.82 (tq, J = 8.0, 4.1 Hz, 4H), 2.95–2.81 (m, 5H), 2.64 (dd, J = 9.0, 6.3 Hz, 2H), 2.29 (s, 3H), 2.20–2.12 (m, 1H), 1.93 (qd, J = 11.1, 5.4 Hz, 8H), 1.81–1.65 (m, 3H), 1.43–1.18 (m, 3H). ^{13}C NMR (101 MHz, $CDCl_3$) δ 169.0, 154.1, 153.6, 141.3, 136.2, 130.0, 129.2, 128.9, 128.9, 128.3, 128.3, 126.8, 113.7, 112.9, 67.2, 57.5, 42.8, 38.0, 32.8, 31.4, 30.3, 28.4, 25.7, 25.2, 25.2, 24.8, 24.8, 24.0, 22.9, 21.0.

HRMS (ESI, m/z): calculated for $C_{30}H_{37}N_5O_2$ $[M+H]^+$ = 500.3020, found 500.3021.

1-allyl-5-methoxyindoline-2,3-dione (95)

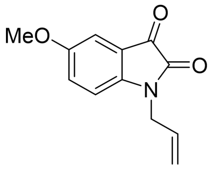

The product was prepared according to the general procedure **A** from 5-methoxyindoline-2,3-dione and 3-bromoprop-1-ene, and purified with chromatography on silica gel (petroleum ether) to afford the title compound **95** in 81% yield as a red solid, 0.88 g. ^{1}H NMR (400 MHz, $CDCl_3$) δ 7.16–7.08 (m, 2H), 6.80 (d, *J* = 8.3 Hz, 1H), 5.82 (ddd, *J* = 10.5, 5.4, 1.2 Hz, 1H), 5.33–5.25 (m, 2H), 4.32 (dt, *J* = 5.3, 1.6 Hz, 2H), 3.79 (s, 3H). ^{13}C NMR (101 MHz, $CDCl_3$) δ 183.7, 158.1, 156.6, 144.8, 130.6, 124.8, 118.7, 118.1, 112.1, 109.6, 56.1, 42.6.

HRMS (ESI, m/z): calculated for $C_{12}H_{11}NO_3$ $[M+H]^+$ = 218.0812, found 218.0816.

1-allyl-5-methoxyspiro[indoline-3,2'-[1,3]dioxolan]-2-one (96)

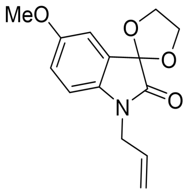

^{1}H NMR (400 MHz, $CDCl_3$) δ 6.95 (dd, *J* = 2.6, 1.0 Hz, 1H), 6.81 (ddd, *J* = 8.5, 2.7, 1.0 Hz, 1H), 6.66 (dd, *J* = 8.5, 1.0 Hz, 1H), 5.75 (ddd, *J* = 10.4, 5.2, 1.0 Hz, 1H), 5.27–5.12 (m, 2H), 4.61–4.48 (m, 2H), 4.34–4.21 (m, 2H), 4.17 (dq, *J* = 4.3, 1.5 Hz, 2H), 3.73 (s, 3H). ^{13}C NMR (101 MHz, $CDCl_3$) δ 173.0, 156.5, 137.2, 131.2, 125.0, 117.8, 116.7, 111.3, 110.4, 102.4, 66.0, 66.0, 55.9, 42.1.

HRMS (ESI, m/z): calculated for $C_{14}H_{15}NO_4$ $[M+H]^+$ = 262.1074, found 262.1077.

(*E*)-5-methoxy-1-(3-(p-tolyl)allyl)indoline-2,3-dione (97)

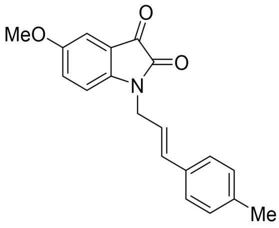

The product was prepared according to the general procedure **C** from 1-allyl-5-methoxyindoline-2,3-dione and 4-methylbenzenesulfonyl chloride, and purified with chromatography on silica gel (petroleum ether) to afford the title compound **97** in 52% yield as a red solid, 79.8 mg. M.P. 149.5-149.8 °C.

^{1}H NMR (400 MHz, $CDCl_3$) δ 7.25 (s, 1H), 7.23 (s, 1H), 7.16 (d, *J* = 2.7 Hz, 1H), 7.13 (s, 1H), 7.14–7.05 (m, 2H), 6.88 (d, *J* = 8.6 Hz, 1H), 6.63 (d, *J* = 16.0 Hz, 1H), 6.11 (dt, *J* = 15.9, 6.1 Hz, 1H), 4.49 (dd, *J* = 6.1, 1.6 Hz, 2H), 3.79 (s, 3H), 2.32 (s, 3H). ^{13}C NMR (101 MHz, $CDCl_3$) δ 183.9, 158.2, 156.6, 144.9, 138.3, 134.1, 133.1, 129.5, 129.5, 126.5, 126.5, 124.9, 120.7, 118.2, 112.1, 109.7, 56.1, 42.4, 21.3.

HRMS (ESI, m/z): calculated for $C_{19}H_{17}NO_3$ $[M+H]^+$ = 308.1281, found 308.1286.

1-(3,3-di-p-tolylallyl)-5-methoxyindoline-2,3-dione (98)

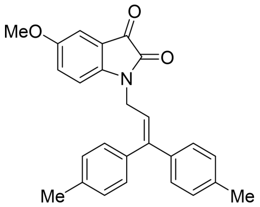

The product was prepared according to the general procedure C from 1-allyl-5-methoxyindoline-2,3-dione and 4-methylbenzenesulfonyl chloride, and purified with chromatography on silica gel (petroleum ether) to afford the title compound **98** in 37% yield as a red solid, 73.4 mg. M.P. 157.2-157-5 °C.

^{1}H NMR (400 MHz, $CDCl_3$) δ 7.25 (d, *J* = 7.4 Hz, 2H), 7.14–7.00 (m, 8H), 6.50 (d, *J* = 8.7 Hz, 1H), 5.92 (t, *J* = 6.5 Hz, 1H), 4.44 (d, *J* = 6.5 Hz, 2H), 3.78 (s, 3H), 2.42 (s, 3H), 2.31 (s, 3H). ^{13}C NMR (101 MHz, $CDCl_3$) δ 158.0, 156.6, 146.6, 146.5, 144.8, 138.5, 138.1, 138.0, 135.8, 129.7, 129.7, 129.4, 129.4, 129.1, 129.1, 127.5, 127.5, 124.7, 120.2, 118.2, 112.0, 109.6, 56.1, 39.6, 21.4, 21.2.

HRMS (ESI, m/z): calculated for $C_{26}H_{23}NO_3$ $[M+H]^+$ = 398.1751, found 398.1753.

8-methoxy-6-(p-tolyl)-5,6-dihydro-2H,4H-spiro[pyrrolo[3,2,1-ij]quinoline-1,2'-[1,3]dioxolan]-2-one (99)

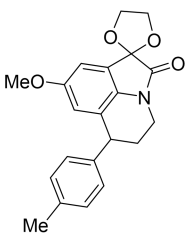

The product was prepared according to the general procedure **C** from 1-allyl-5-methoxyspiro[indoline-3,2'-[1,3]dioxolan]-2-one and 4-methylbenzenesulfonyl chloride, and purified with chromatography on silica gel (petroleum ether) to afford the title compound **99** in 51% yield as a white solid, 89.5 mg. M.P. 132.5-132.7 °C.

^{1}H NMR (400 MHz, $CDCl_3$) δ 7.13 (d, *J* = 7.8 Hz, 2H), 7.00 (d, *J* = 8.1 Hz, 2H), 6.86 (d, *J* = 2.3 Hz, 1H), 6.44 (dd, *J* = 2.4, 0.9 Hz, 1H), 4.67–4.55 (m, 2H), 4.41–4.27 (m, 2H), 4.04 (dd, *J* = 7.7, 4.6 Hz, 1H), 3.68 (s, 3H), 3.61 (td, *J* = 7.0, 4.6 Hz, 2H), 2.34 (s, 3H), 2.28–1.99 (m, 2H). ^{13}C NMR (101 MHz, $CDCl_3$) δ 171.9, 156.6, 139.8, 136.7, 134.1, 129.5, 129.5, 129.5, 128.3, 128.3, 124.4, 123.2, 116.5, 109.6, 103.8, 65.9 (d, *J* = 3.1 Hz), 56.0, 40.8, 36.4, 30.1, 21.1.

HRMS (ESI, m/z): calculated for $C_{21}H_{21}NO_4$ $[M+H]^+$ = 352.1543, found 352.1545.

2,6-di-tert-butyl-4-(tosylmethyl)phenol[19] (100)

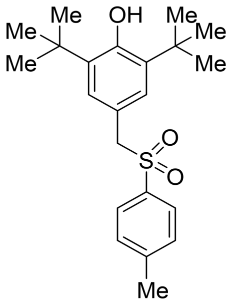

The compound was prepared according to the procedure mentioned above using $PdCl_2$ (2.2 mg, 0.0125 mmol), Li_2CO_3 (111.0 mg, 1.50 mmol), CuBr (35.9 mg, 0.25 mmol), Tosyl chloride (0.75 mmol.) , BHT(132.2 mg, 1.2 equiv.) and DMC (2ml) reflux for 14h.Upon cooling, the mixture was filtered through a short silica gel column for GC-MS. Then the combined filtrate was concentrated under vacuo and purified by silica gel column chromatography using EA/PE as eluent to afford the sulfonylation product [2,6-di-tert-butyl-4-(tosylmethyl)phenol] as colorless oil in 45% yield, 84.2 mg.

^{1}H NMR (500 MHz, $CDCl_3$) δ 7.44 (d, *J* = 8.3 Hz, 2H), 7.21 (d, *J* = 8.3 Hz, 2H), 6.73 (s, 2H), 5.23 (s, 1H), 4.19 (s, 2H), 2.40 (s, 3H), 1.32 (s, 18H).

4-(1-oxo-1,2,7,8,9,10-hexahydro-3-oxa-10a-azacyclohepta[*de*]naphthalen-7-yl)benzonitrile (101)

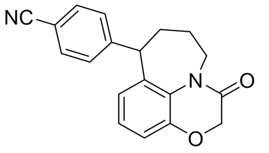

The product was prepared according to the general procedure **C** from 4-(but-3-en-1-yl)-2*H*-benzo[*b*][1,4]oxazin-3(4*H*)-one and 4-cyanobenzenesulfonyl chloride, and purified with chromatography on silica gel (petroleum ether) to afford the title compound **101** in 27% yield as acolorless oil, 41.1 mg.

^{1}H NMR (400 MHz, $CDCl_3$) δ 7.62 (d, *J* = 8.3 Hz, 2H), 7.47 (d, *J* = 8.3 Hz, 2H), 7.00 (dd, *J* = 3.8, 1.9 Hz, 2H), 6.93 (dd, *J* = 6.9, 2.6 Hz, 1H), 4.95 (dd, *J* = 8.8, 5.4 Hz, 1H), 4.57 (s, 2H), 4.15–3.88 (m, 2H), 2.20–1.95 (m, 2H), 1.98–1.68 (m, 2H). ^{13}C NMR (101 MHz, $CDCl_3$) δ 164.6, 146.5, 145.5, 132.7, 132.7, 128.1, 127.8, 127.8, 124.2, 123.0, 118.6, 117.5, 114.8, 112.3, 67.7, 61.6, 39.7, 36.8, 24.5.

HRMS (ESI, m/z): calculated for $C_{19}H_{16}N_2O_2$ $[M+H]^+$ = 305.1285, found 305.1288.

7-(*p*-tolyl)-7,8,9,10,11,12-hexahydro-3-oxa-12a-azacyclonona[*de*]naphthalen-1(2*H*)-one (102)

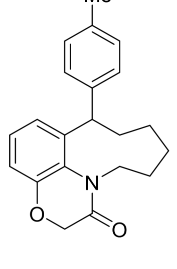

The product was prepared according to the general procedure **C** from 4-(hex-5-en-1-yl)-2H-benzo[b][1,4]oxazin-3(4H)-one and 4-methylbenzenesulfonyl chloride, and purified with chromatography on silica gel (petroleum ether) to afford the title compound **102** in 32% yield as a colorless oil, 51.4 mg.

^{1}H NMR (400 MHz, $CDCl_3$) δ 7.90–7.83 (m, 2H), 7.58–7.51 (m, 2H), 7.25–7.21 (m, 2H), 7.01 (dd, J = 43.0, 7.4 Hz, 1H), 4.86–4.83 (m, 2H), 4.25–4.09 (m, 2H), 3.71 (dq, J = 11.0, 6.0 Hz, 1H), 3.48–3.16 (m, 2H), 2.48–2.20 (m, 3H), 2.10–1.51 (m, 6H). ^{13}C NMR (126 MHz, $CDCl_3$) δ 164.4, 149.0, 145.5, 132.6, 132.6, 128.6, 128.6, 128.2, 124.0, 122.8, 118.8, 117.3, 114.8, 110.8, 67.7, 42.7, 42.6, 40.5, 39.0, 32.9, 24.8.

HRMS (ESI, m/z): calculated for $C_{21}H_{23}NO_2$ $[M+H]^+$ = 322.1802, found 322.1804.

4-allyl-2H-benzo[b][1,4]oxazin-3(4H)-one-*d*1 (92% *d*) (Y4)

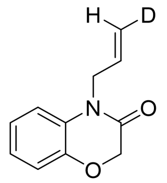

^{1}H NMR (400 MHz, $CDCl_3$) δ 6.99 (d, J = 2.1 Hz, 4H), 5.94 – 5.81 (m, 1H), 5.28–5.15 (m, 1H), 4.63 (s, 2H), 4.57–4.51 (m, 2H). ^{13}C NMR (126 MHz, $CDCl_3$) δ 164.3, 145.5, 131.5, 128.7, 124.1, 122.2, 117.2, 117.1, 115.6, 67.7, 43.8.

HRMS (ESI, m/z): calculated for $C_{11}H_{10}DNO_2$ $[M+H]^+$ = 191.0925, found 191.0927.

7-(p-tolyl)-6,7-dihydro-5H-[1,4]oxazino[2,3,4-ij]quinolin-3(2H)-one-7-*d* (96%*d*) (103)

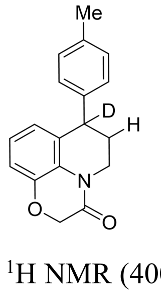

^{1}H NMR (400 MHz, $CDCl_3$) δ 7.14 (d, J = 7.8 Hz, 2H), 6.99 (d, J = 8.0 Hz, 2H), 6.87 (d, J = 4.6 Hz, 2H), 6.60 (q, J = 4.4 Hz, 1H), 4.67 (s, 2H), 3.94–3.79 (m, 2H), 2.35 (s, 3H), 2.30–2.19 (m, 1H), 2.17–2.00 (m, 1H). ^{13}C NMR (101 MHz, $CDCl_3$) δ 163.6, 144.4, 141.0, 136.5, 129.4, 129.4, 128.4, 128.4, 128.4, 125.4, 123.8, 123.4, 115.0, 67.4, 42.0, 37.2, 29.8, 21.1.

HRMS (ESI, m/z): calculated for $C_{18}H_{16}DNO_2$ $[M+H]^+$ = 281.1395, found 281.1399.

4-(2-methylallyl-3,3-d2)-2H-benzo[b][1,4]oxazin-3(4H)-one (Y6)

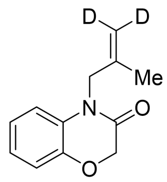

^{1}H NMR (400 MHz, $CDCl_3$) δ 7.03–6.89 (m, 4H), 4.65 (s, 2H), 4.46 (s, 2H), 1.78 (s, 3H). ^{13}C NMR (101 MHz, $CDCl_3$) δ 164.3, 145.1, 139.1, 128.9, 124.1, 122.9, 116.8, 115.8, 111.5, 67.6, 46.8, 20.1.

HRMS (ESI, m/z): calculated for $C_{12}H_{11}D_2NO_2$ $[M+H]^+$ = 206.1145, found 206.1147.

6-methyl-7-(p-tolyl)-6,7-dihydro-5H-[1,4]oxazino[2,3,4-ij]quinolin-3(2H)-one-6,7-*d*2 (104)

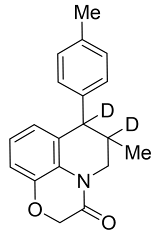

^{1}H NMR (400 MHz, $CDCl_3$) δ 7.14 (d, J = 7.8 Hz, 2H), 6.98 (d, J = 7.9 Hz, 2H), 6.85–6.79 (m, 2H), 6.45 (dd, J = 6.5, 2.7 Hz, 1H), 4.69–4.59 (m, 2H), 4.22–4.26 (m, 1H), 3.36–3.26 (m, 1H), 2.35 (s, 3H), 0.96 (s, 3H). ^{13}C NMR (101 MHz, $CDCl_3$) δ 163.7, 144.2, 140.4, 136.6, 129.4, 129.4, 128.9, 128.9, 128.7, 125.1, 124.2, 123.4, 114.7, 67.4, 50.1, 43.9, 33.8, 21.2, 17.5.

HRMS (ESI, m/z): calculated for $C_{19}H_{17}D_2NO_2$ $[M+H]^+$ = 296.1614, found 296.1616.

4-(p-tolyl)chromane-5,6,7,8-*d*4 (105)

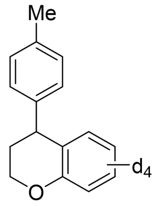

^{1}H NMR (400 MHz, $CDCl_3$) δ 7.20–7.11 (m, 2H), 7.09–7.00 (m, 2H), 4.27–4.13 (m, 3H), 2.36 (s, 3H), 2.33–2.24 (m, 1H), 2.18–2.04 (m, 1H). ^{13}C NMR (101 MHz, $CDCl_3$) δ 155.2, 142.8, 136.1, 130.3, 129.2, 129.2, 128.6, 128.6, 127.7, 124.8, 119.8, 116.5, 64.0, 40.7, 31.8, 21.1.

HRMS (ESI, m/z): calculated for $C_{16}H_{12}D_4O$ $[M+H]^+$ = 229.1525, found 229.1526.

NMR spectra

4-allyl-2H-benzo[b][1,4]oxazin-3(4H)-one (2)

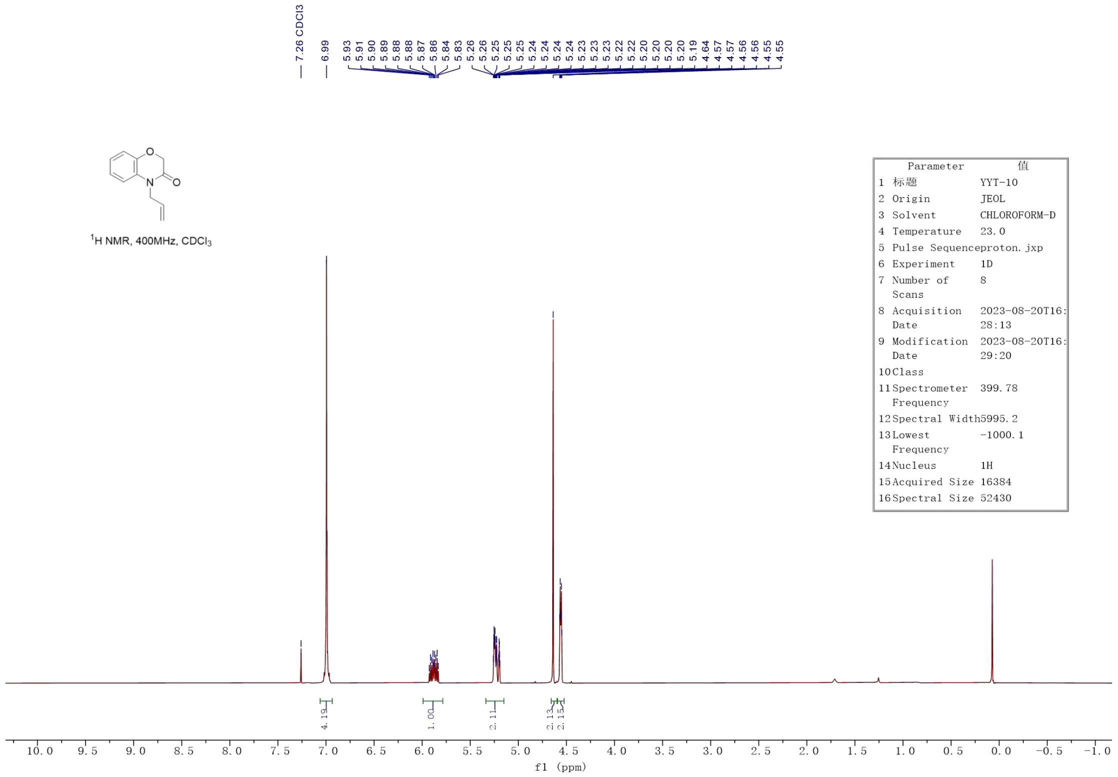

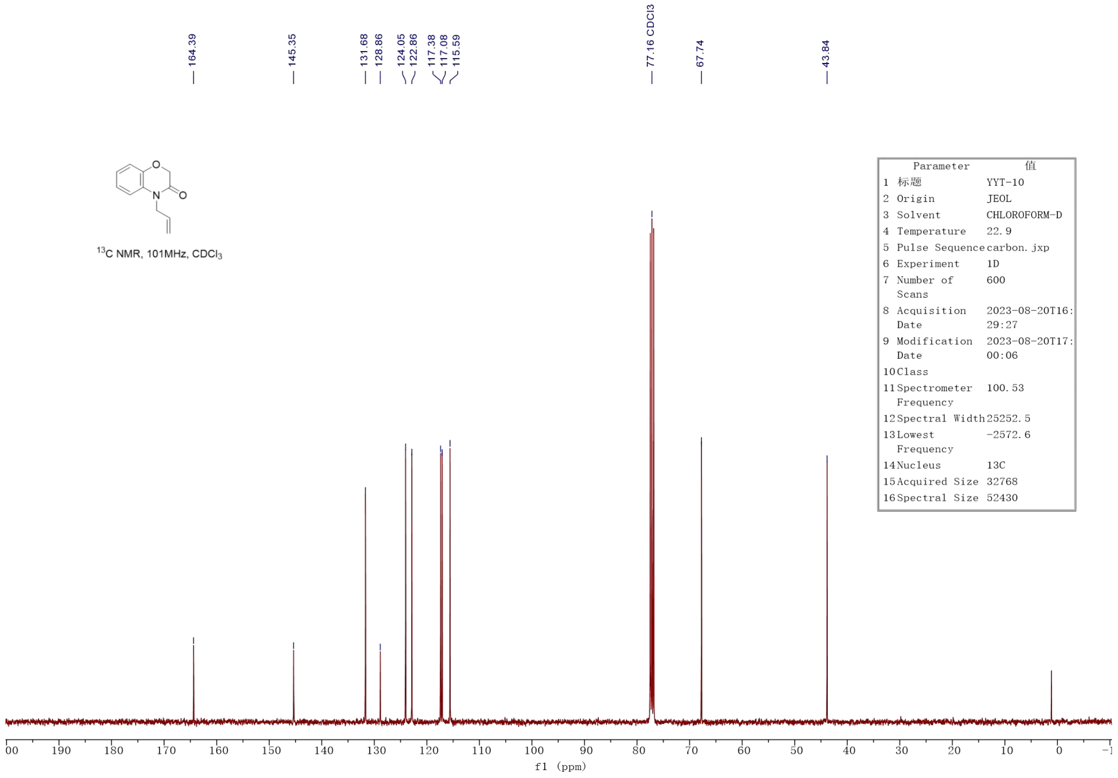

4-allyl-7-methoxy-2H-benzo[b][1,4]oxazin-3(4H)-one (S45)

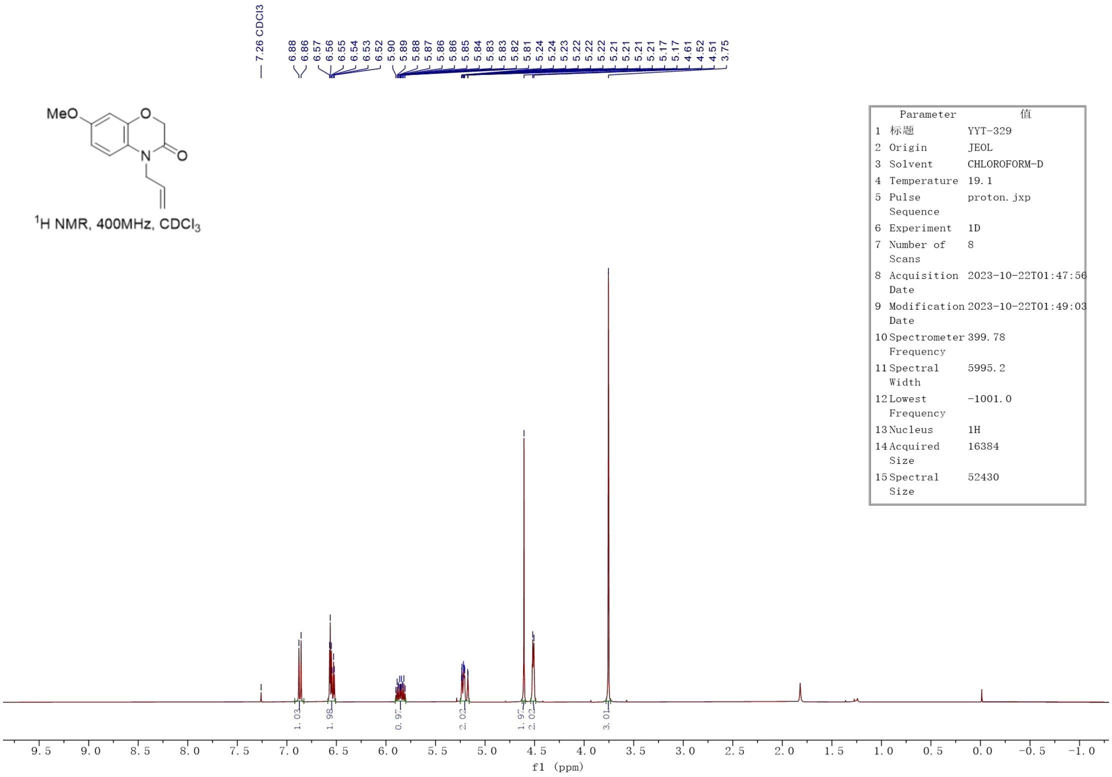

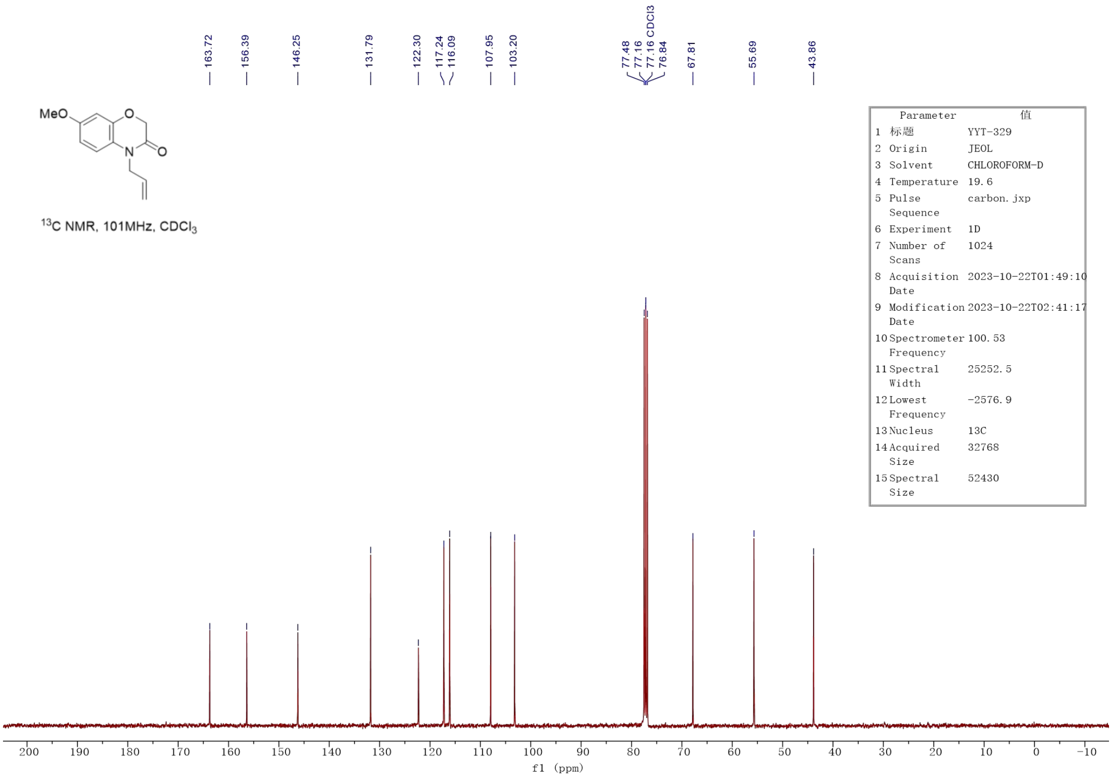

4-allyl-6-methyl-2H-benzo[b][1,4]oxazin-3(4H)-one (S46)

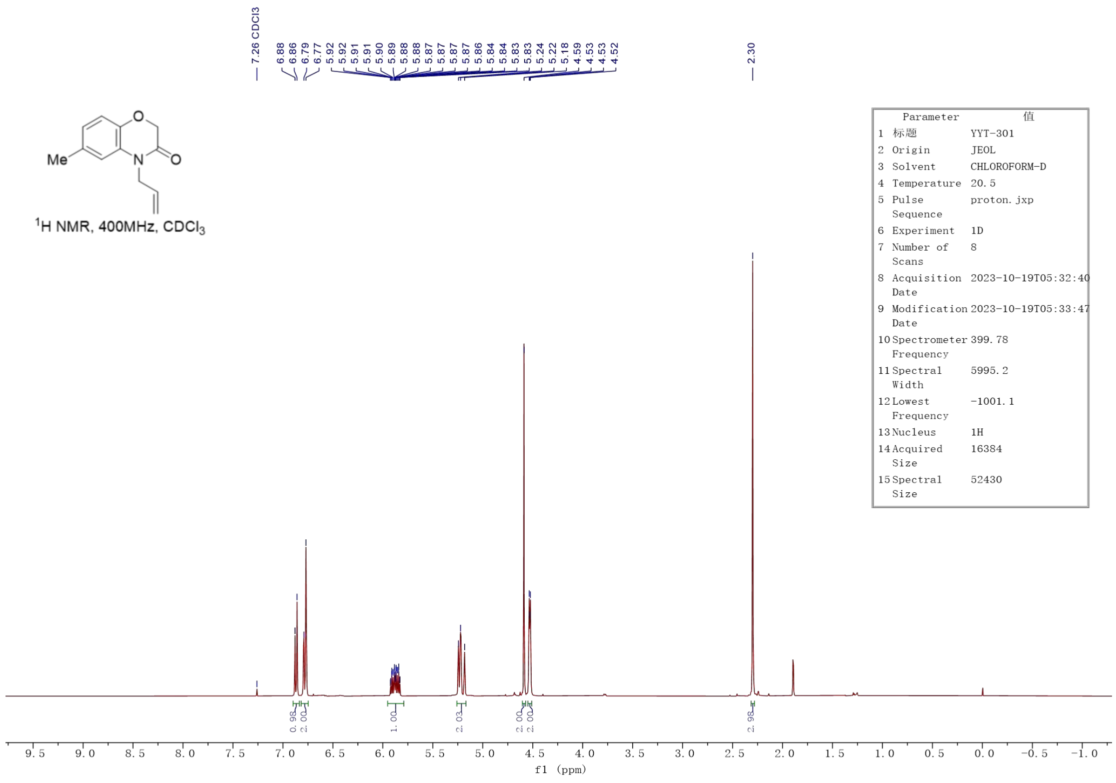

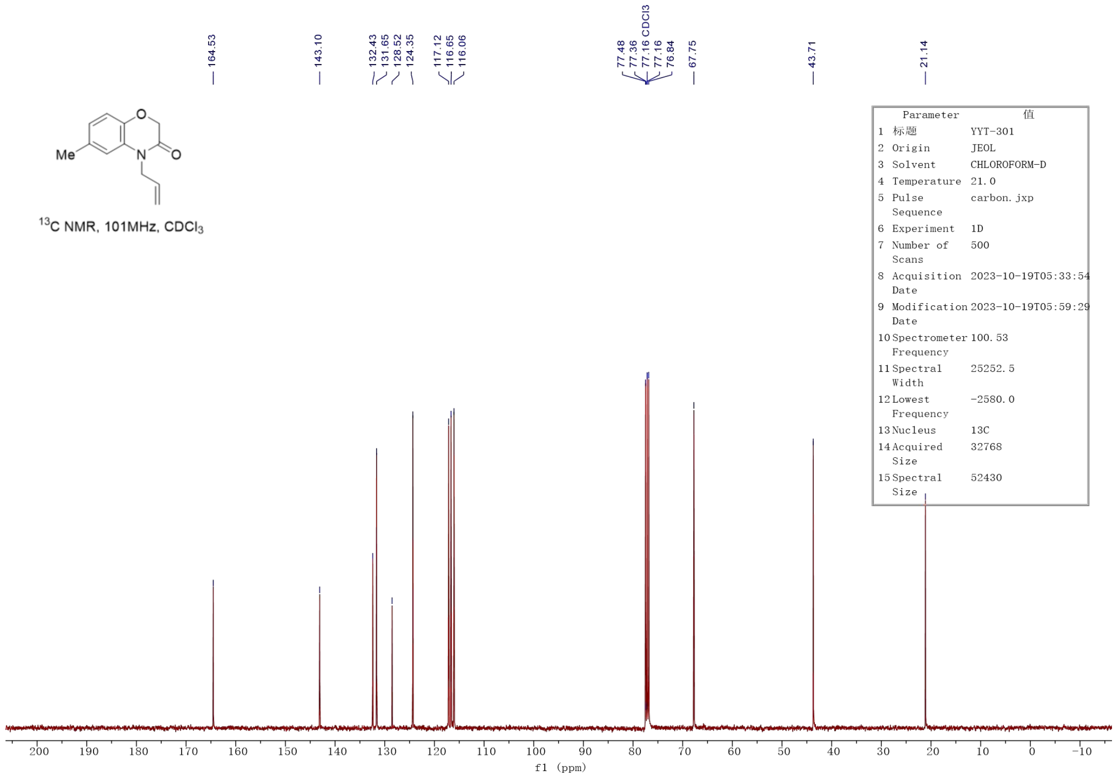

4-allyl-7-fluoro-2H-benzo[b][1,4]oxazin-3(4H)-one (S47)

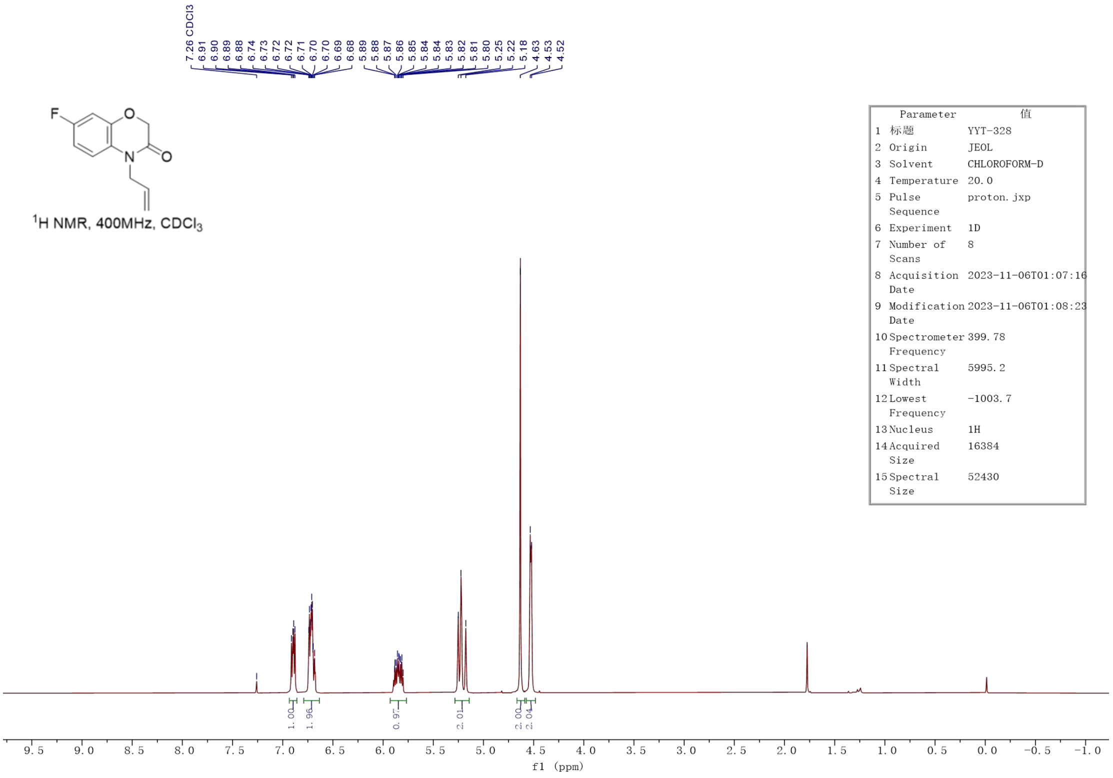

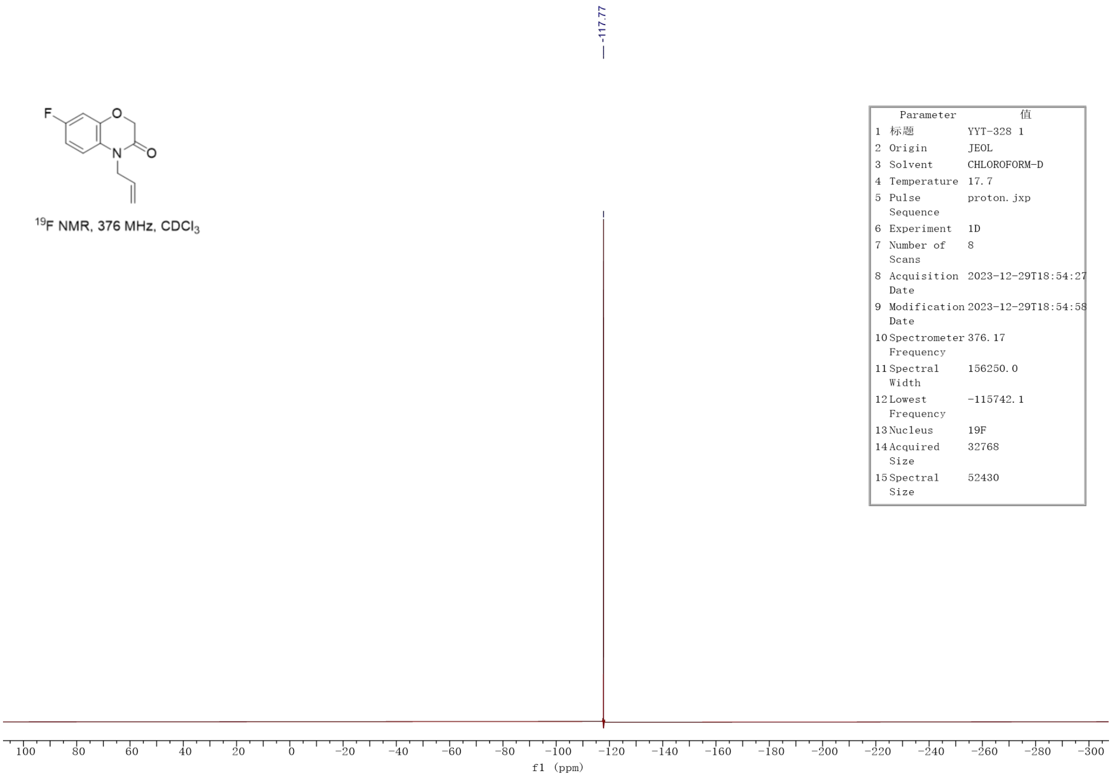

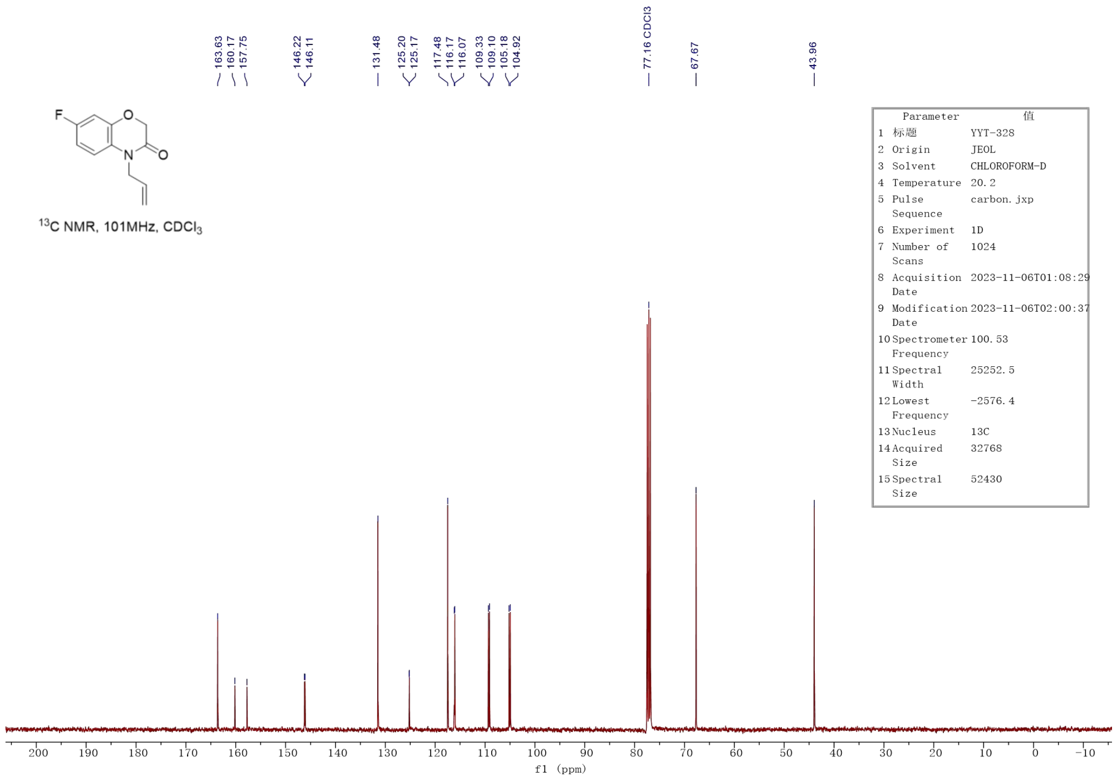
163.63
160.17
157.75
146.22
146.11
131.48
125.20
125.17
117.48
116.17
116.07
109.33
109.10
105.18
104.92
77.16 CDCl3
67.67
43.96
F
O
N
O
^{13}C NMR, 101MHz, $CDCl_3$
Parameter 值
1 标题 YYT-328
2 Origin JEOL
3 Solvent CHLOROFORM-D
4 Temperature 20.2
5 Pulse Sequence carbon.jxp
6 Experiment 1D
7 Number of Scans 1024
8 Acquisition Date 2023-11-06T01:08:29
9 Modification Date 2023-11-06T02:00:37
10 Spectrometer Frequency 100.53
11 Spectral Width 25252.5
12 Lowest Frequency -2576.4
13 Nucleus 13C
14 Acquired Size 32768
15 Spectral Size 52430
f1 (ppm)

1-allyl-3,4-dihydroquinolin-2(1H)-one (S48)

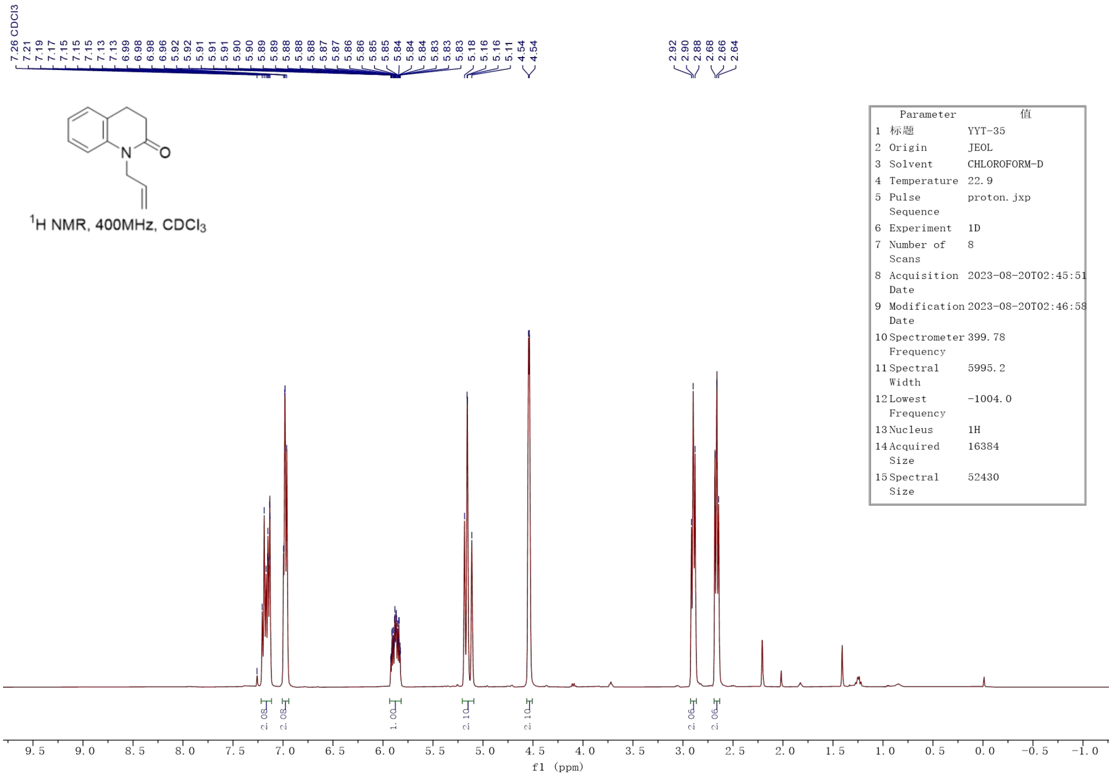

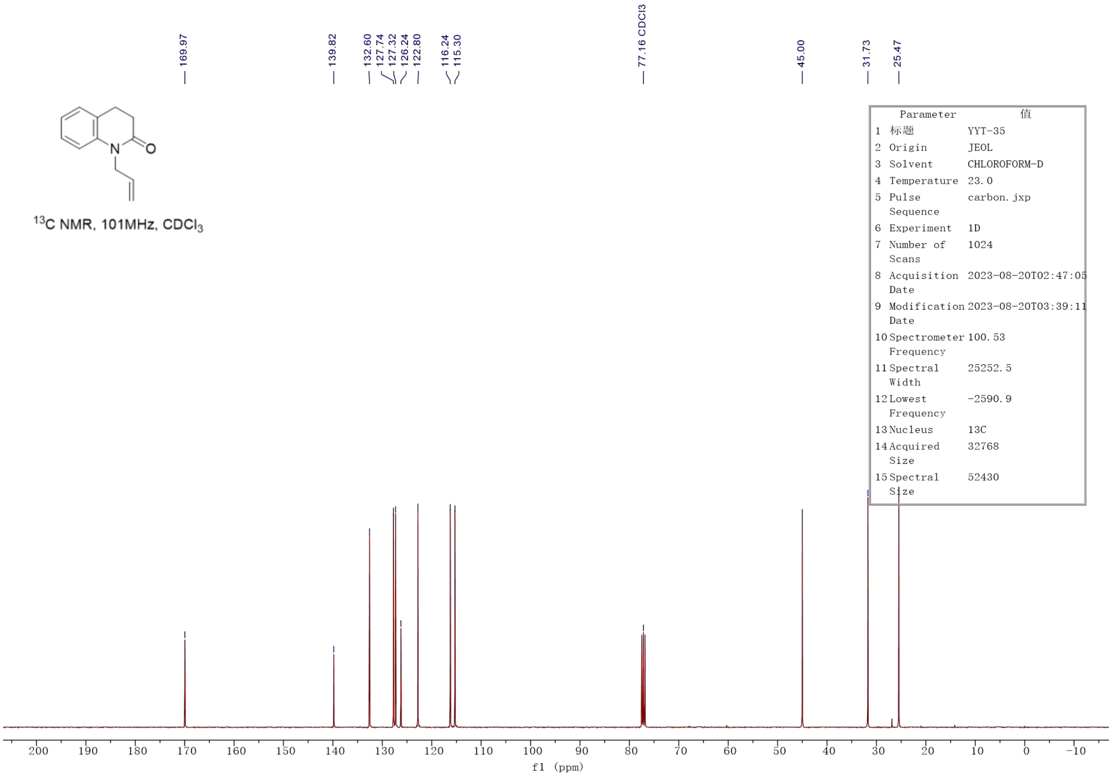

1-allyl-6-methoxy-3,4-dihydroquinolin-2(1H)-one (S49)

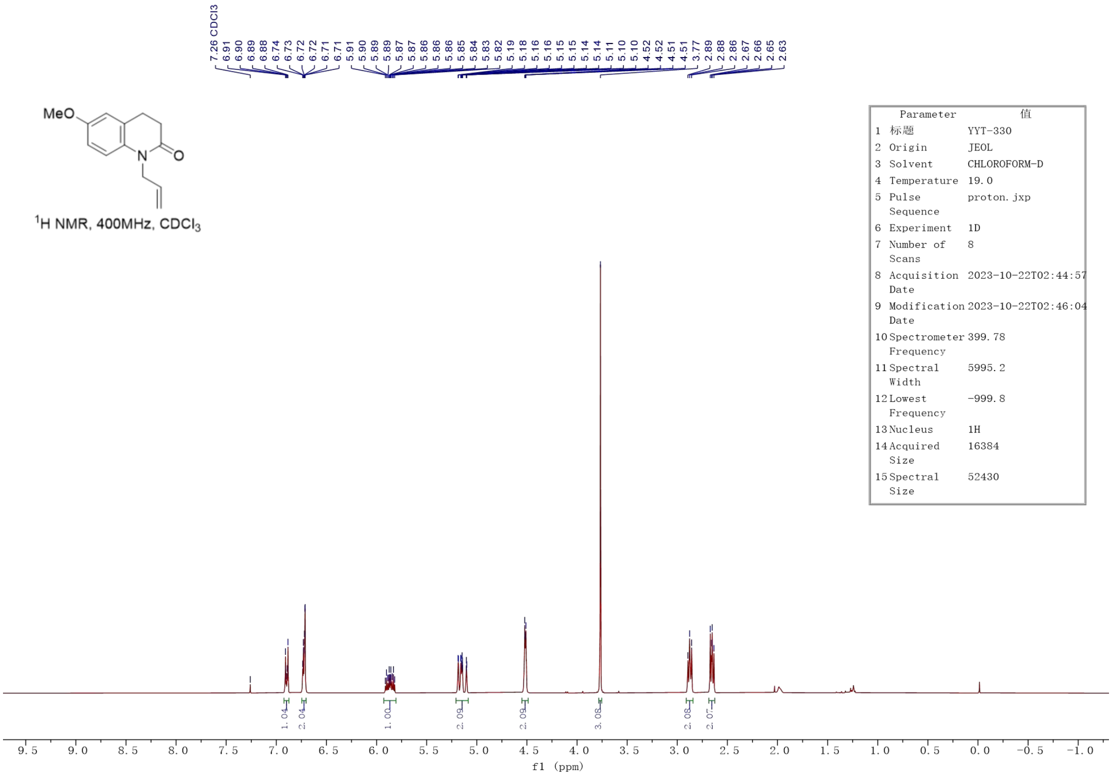

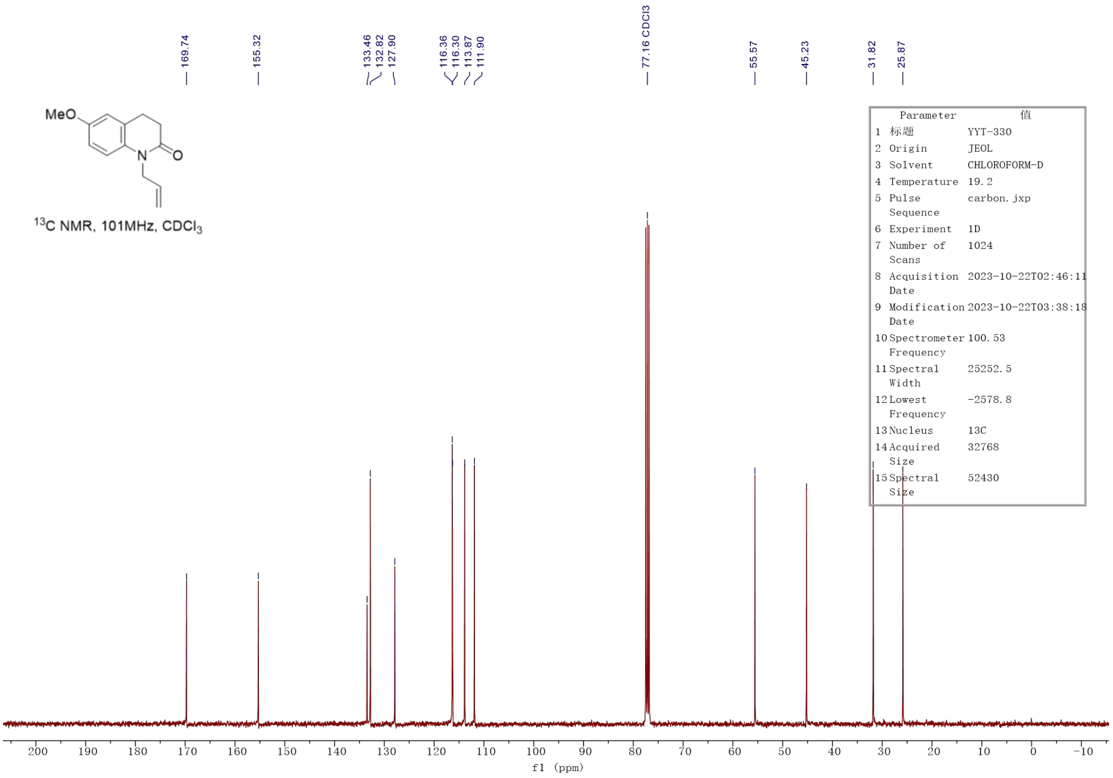

1-allyl-6-((tert-butyldimethylsilyl)oxy)-3,4-dihydroquinolin-2(1H)-one (S50)

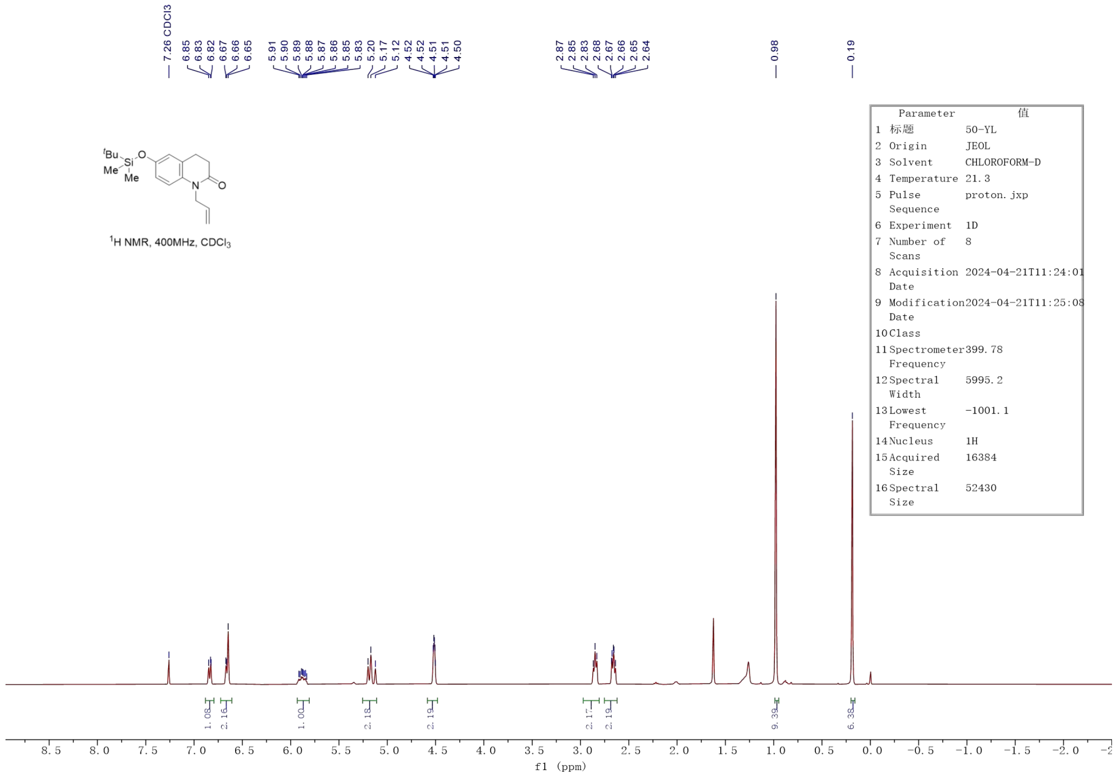

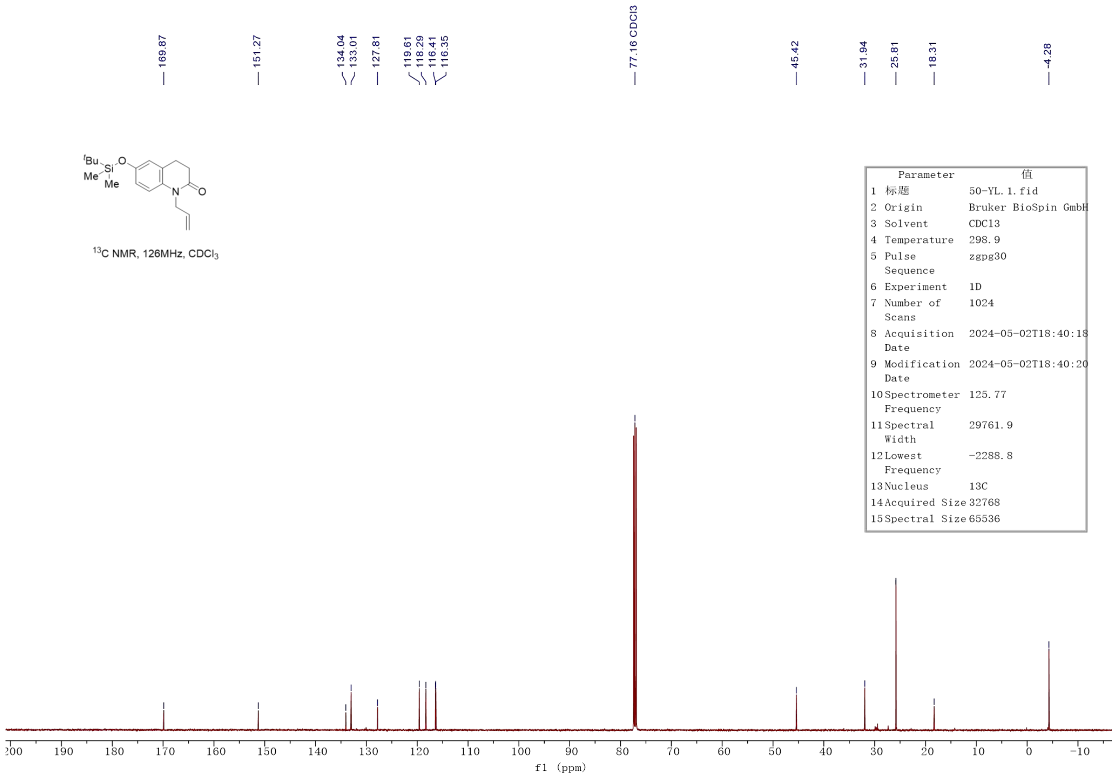

1-allyl-2-oxo-1,2,3,4-tetrahydroquinolin-6-yl benzoate (S51)

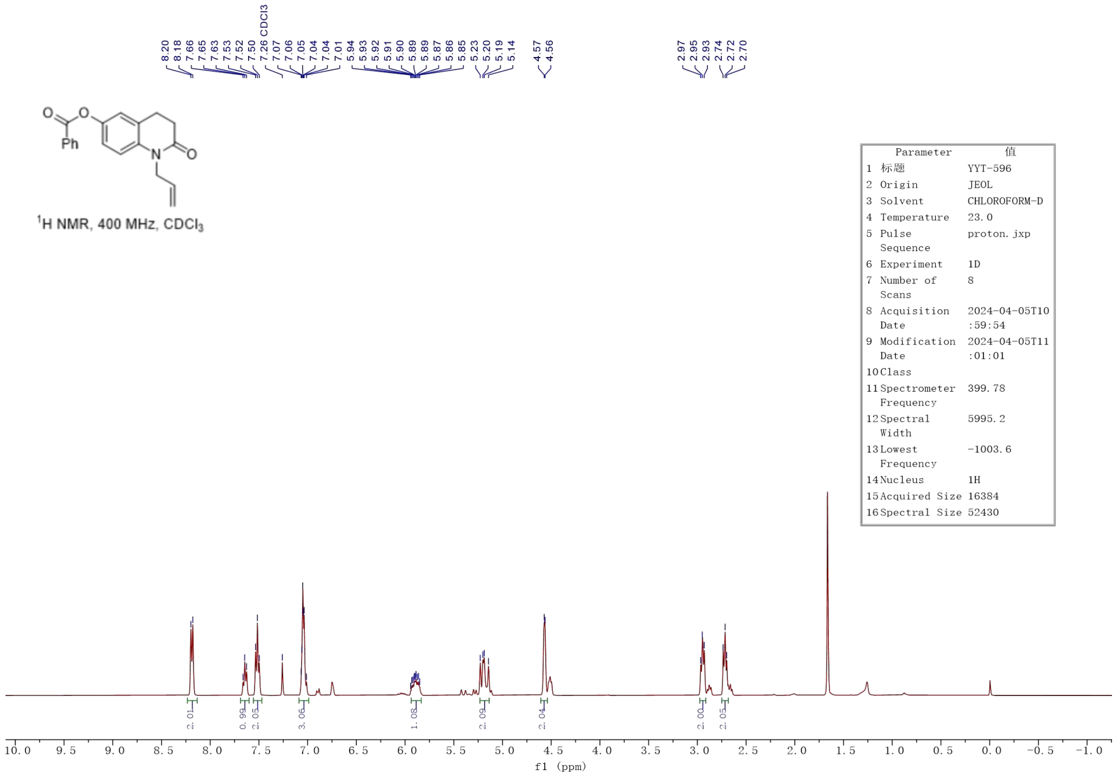

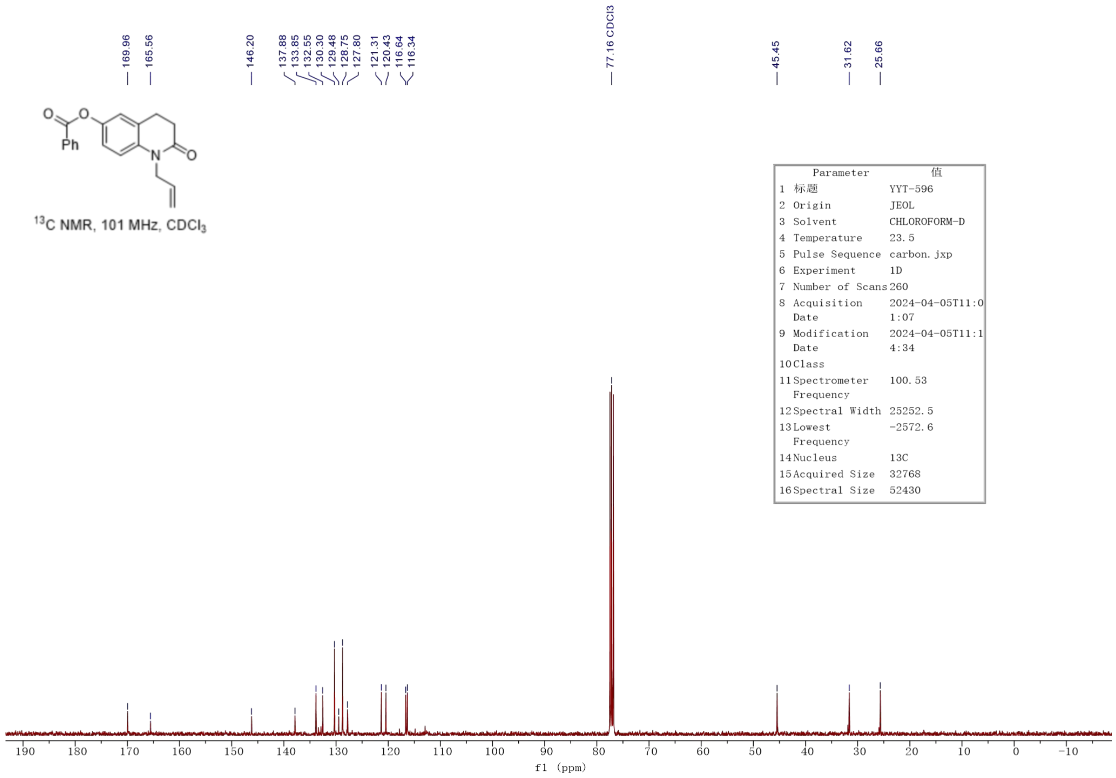

1-allyl-6-bromo-4,4-dimethyl-3,4-dihydroquinolin-2(1H)-one (S52)

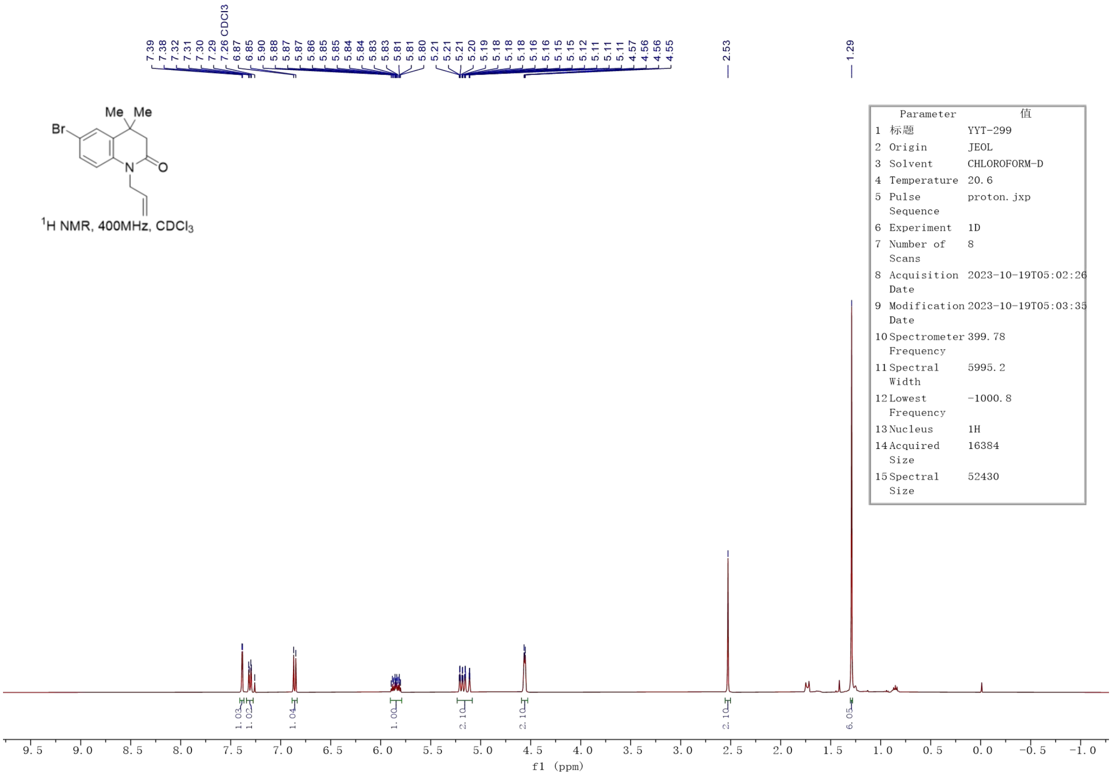

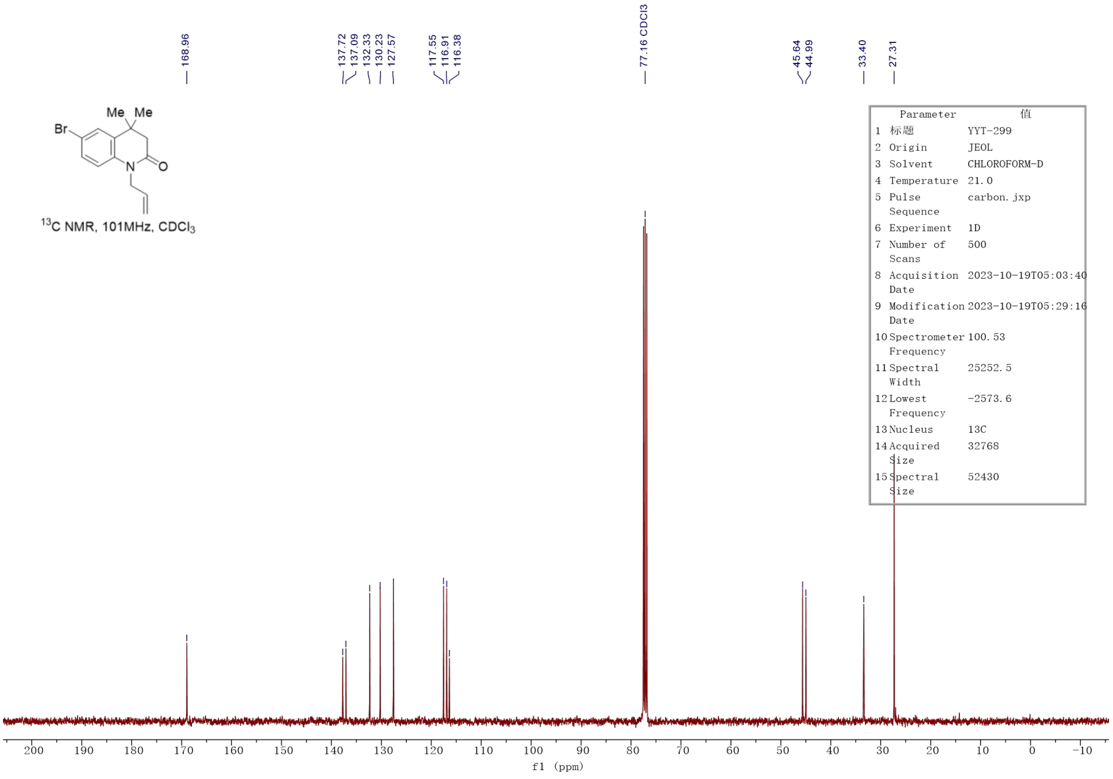

1-allyl-6-fluoro-3,4-dihydroquinolin-2(1H)-one (S53)

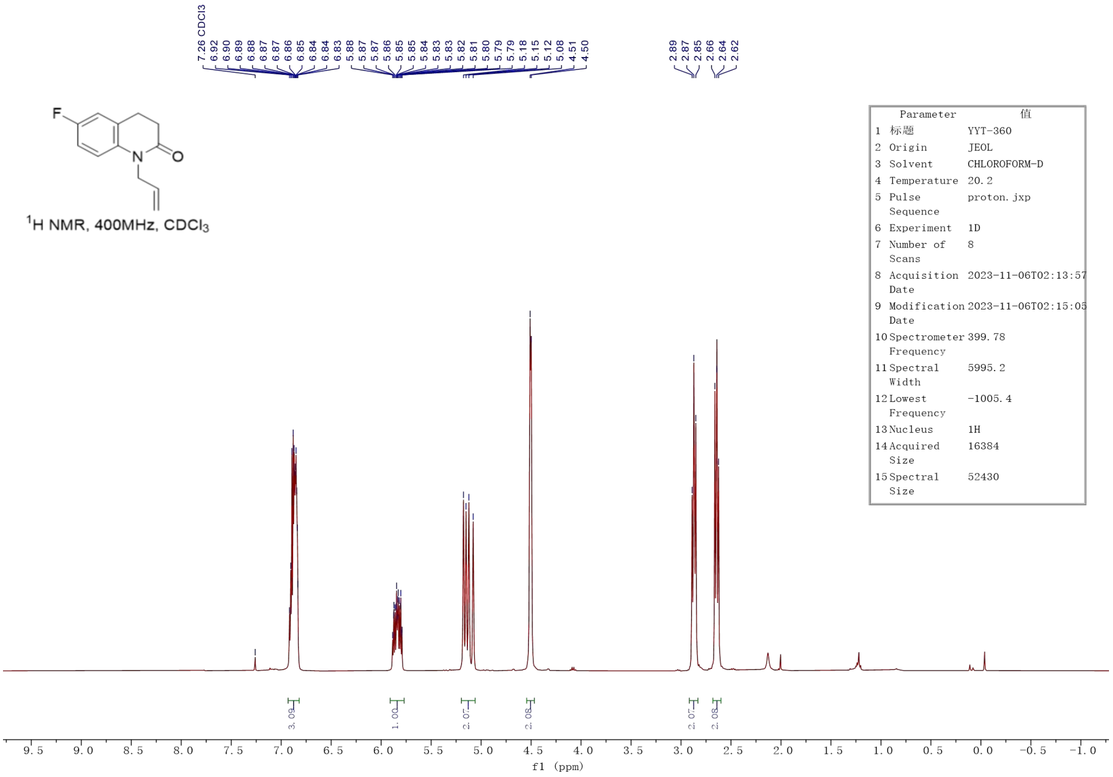

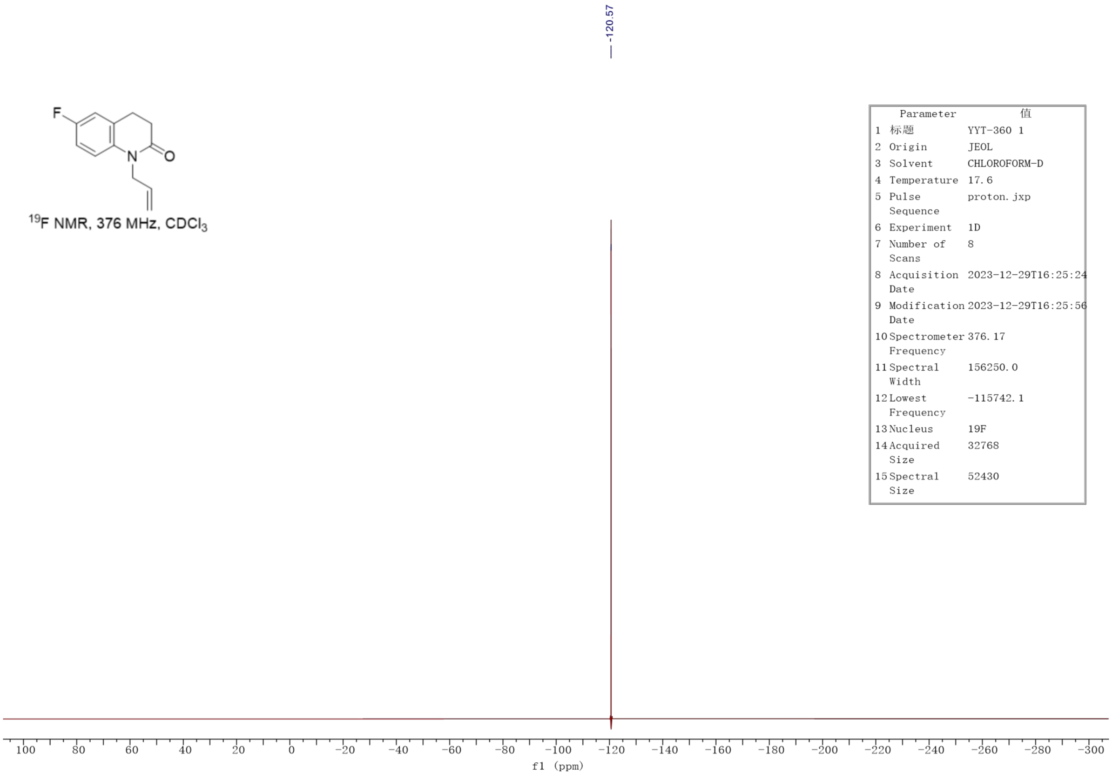

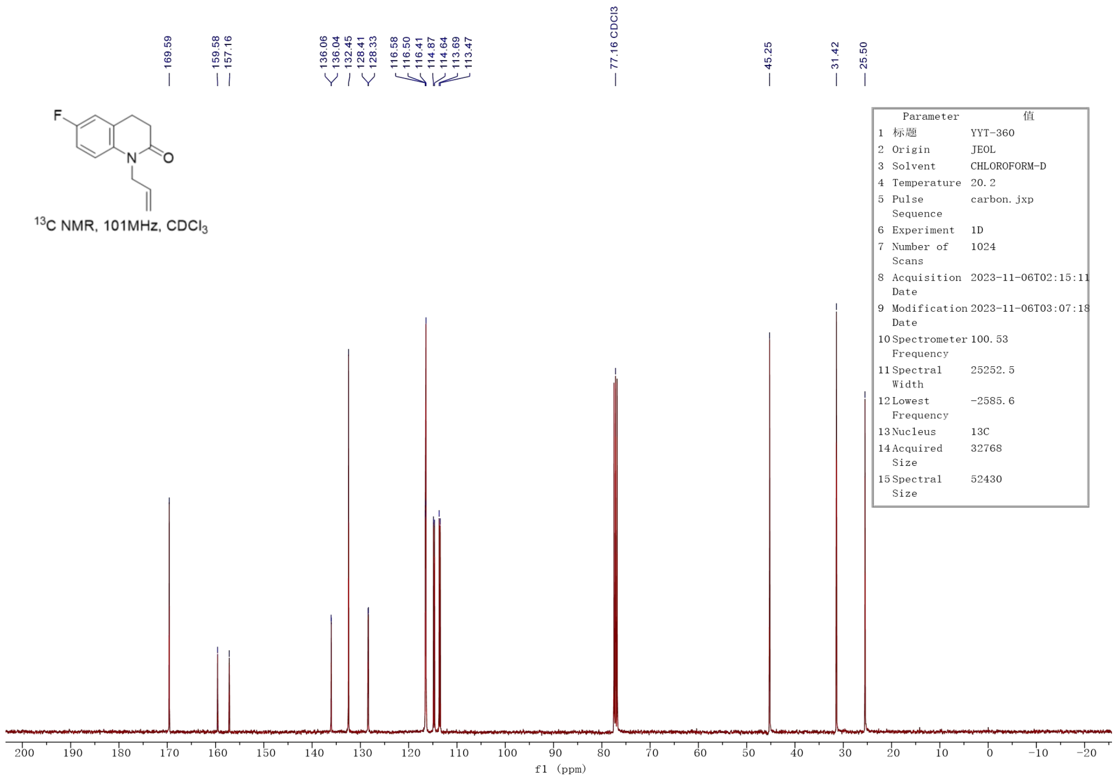
169.59
159.58
157.16
136.06
136.04
132.45
128.41
128.33
116.58
116.50
116.41
114.87
114.64
113.69
113.47
77.16 CDCl3
45.25
31.42
25.50
F
N
O
13C NMR, 101MHz, CDCl3
Parameter 值
1 标题 YYT-360
2 Origin JEOL
3 Solvent CHLOROFORM-D
4 Temperature 20.2
5 Pulse Sequence carbon.jxp
6 Experiment 1D
7 Number of Scans 1024
8 Acquisition Date 2023-11-06T02:15:11
9 Modification Date 2023-11-06T03:07:18
10 Spectrometer Frequency 100.53
11 Spectral Width 25252.5
12 Lowest Frequency -2585.6
13 Nucleus 13C
14 Acquired Size 32768
15 Spectral Size 52430
f1 (ppm)

1-allyl-6-chloro-3,4-dihydroquinolin-2(1H)-one (S54)

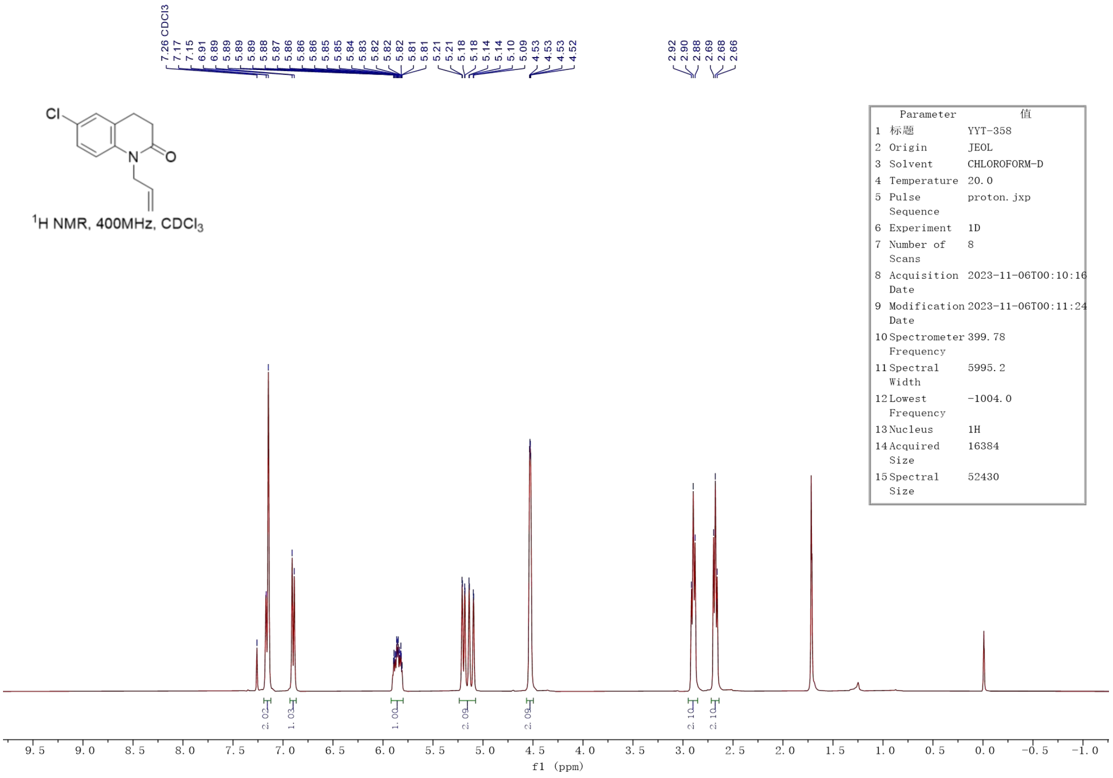

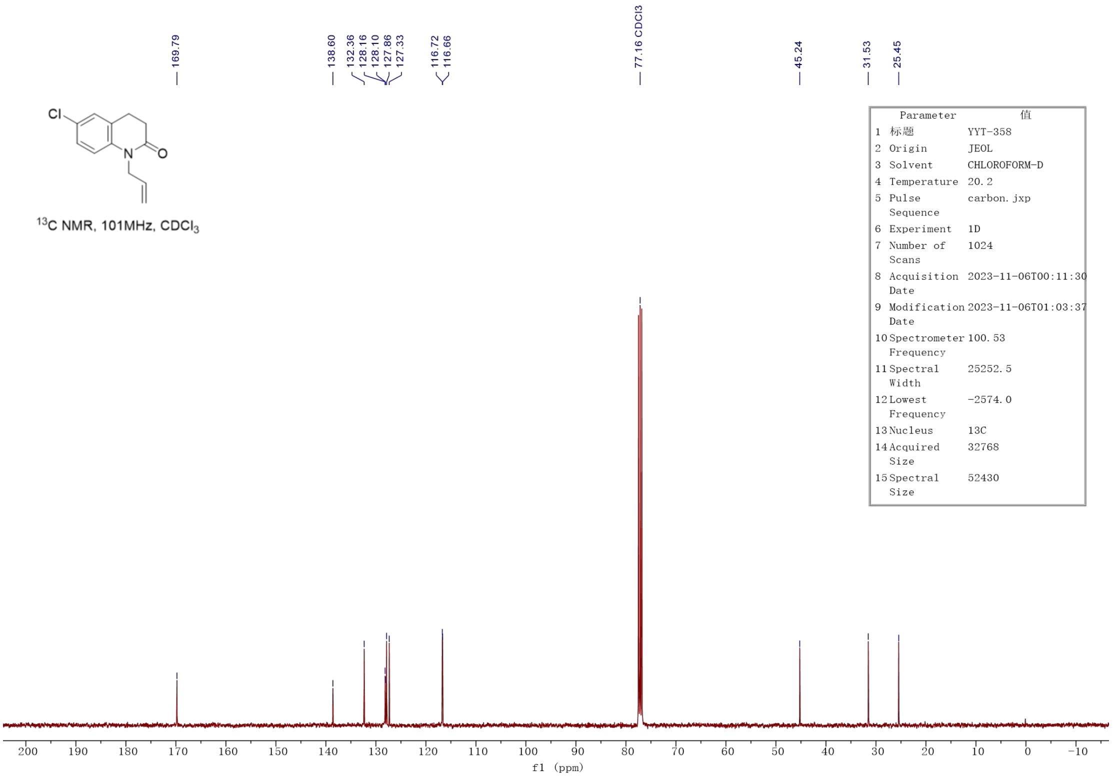

1-allyl-6-bromo-3,4-dihydroquinolin-2(1H)-one (S55)

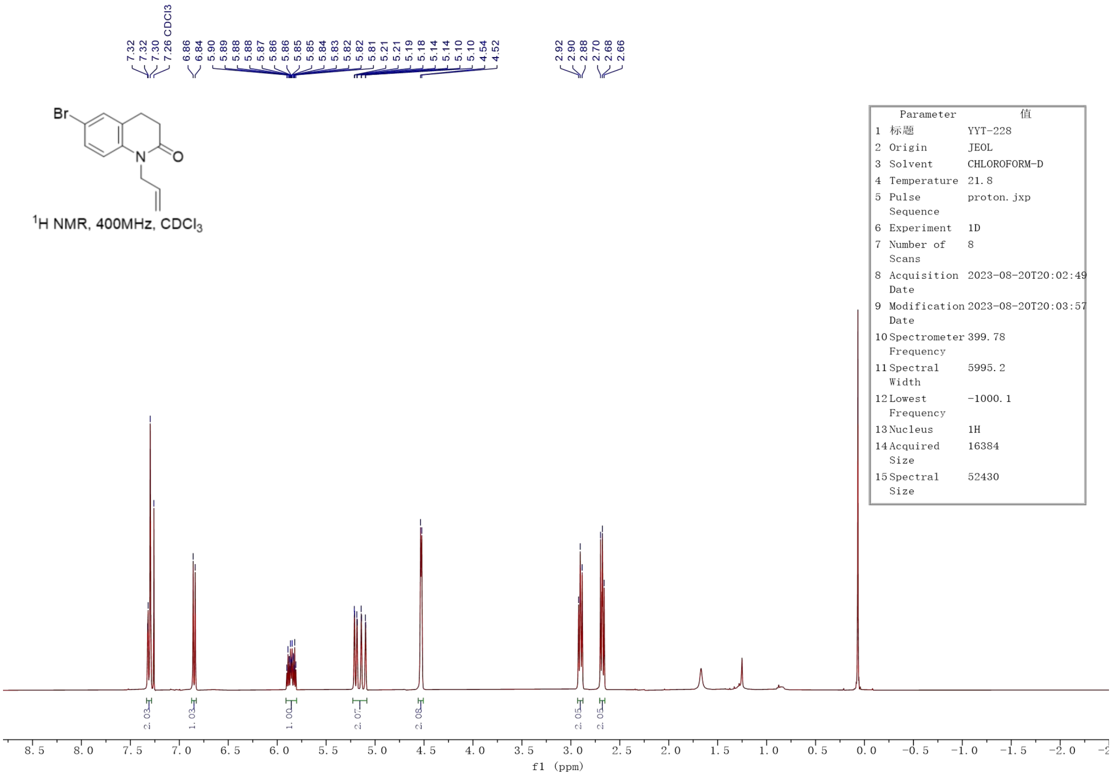

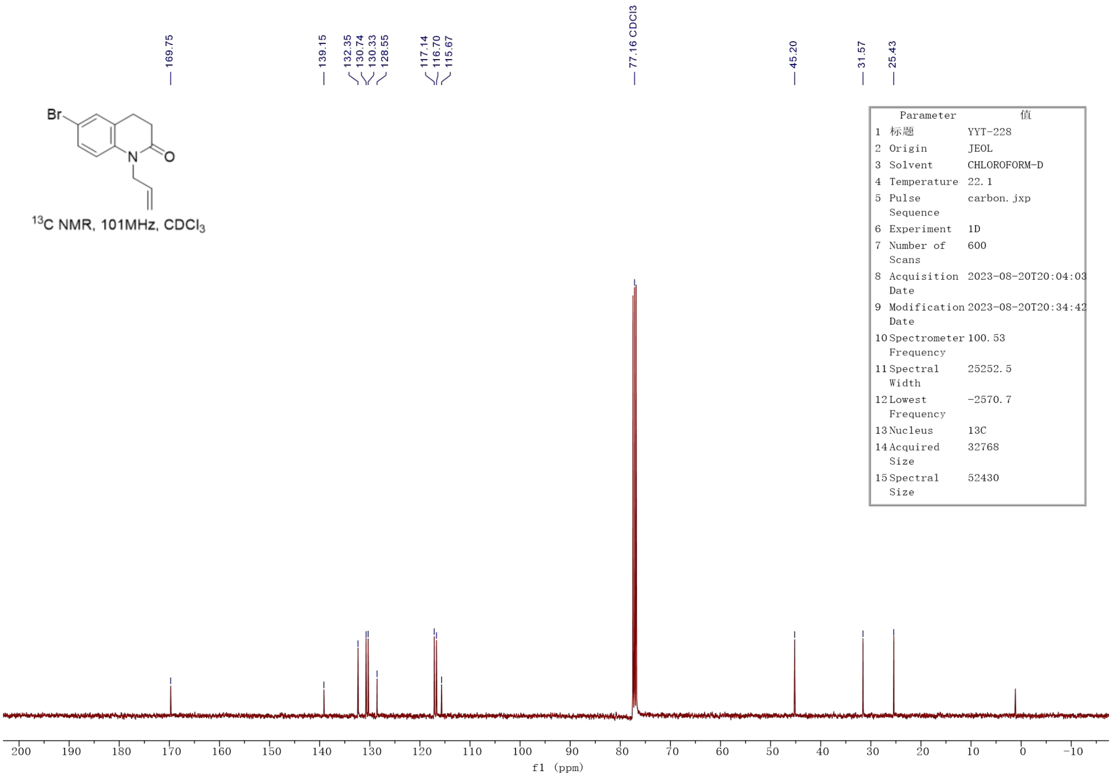

1-allyl-5-methyl-3,4-dihydroquinolin-2(1H)-one (S56)

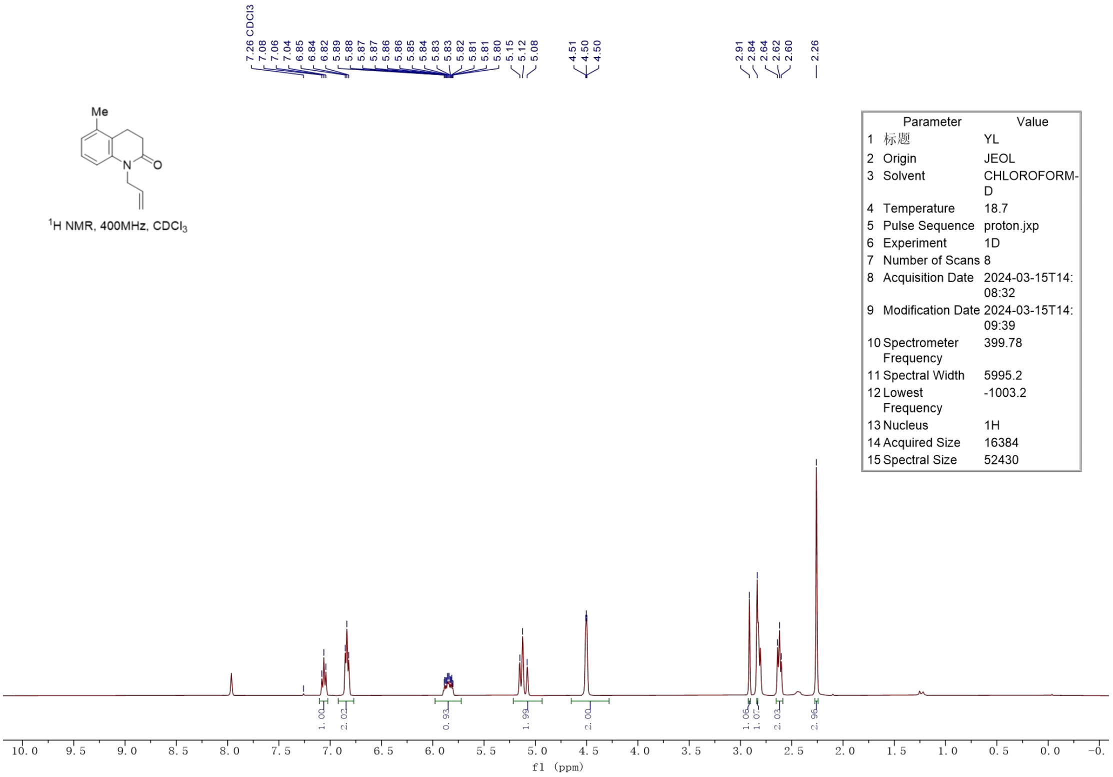

^{1}H NMR, 400MHz, $CDCl_3$

Parameter	Value
1 标题	YL
2 Origin	JEOL
3 Solvent	CHLOROFORM-D
4 Temperature	18.7
5 Pulse Sequence	proton.jxp
6 Experiment	1D
7 Number of Scans	8
8 Acquisition Date	2024-03-15T14:08:32
9 Modification Date	2024-03-15T14:09:39
10 Spectrometer Frequency	399.78
11 Spectral Width	5995.2
12 Lowest Frequency	-1003.2
13 Nucleus	1H
14 Acquired Size	16384
15 Spectral Size	52430

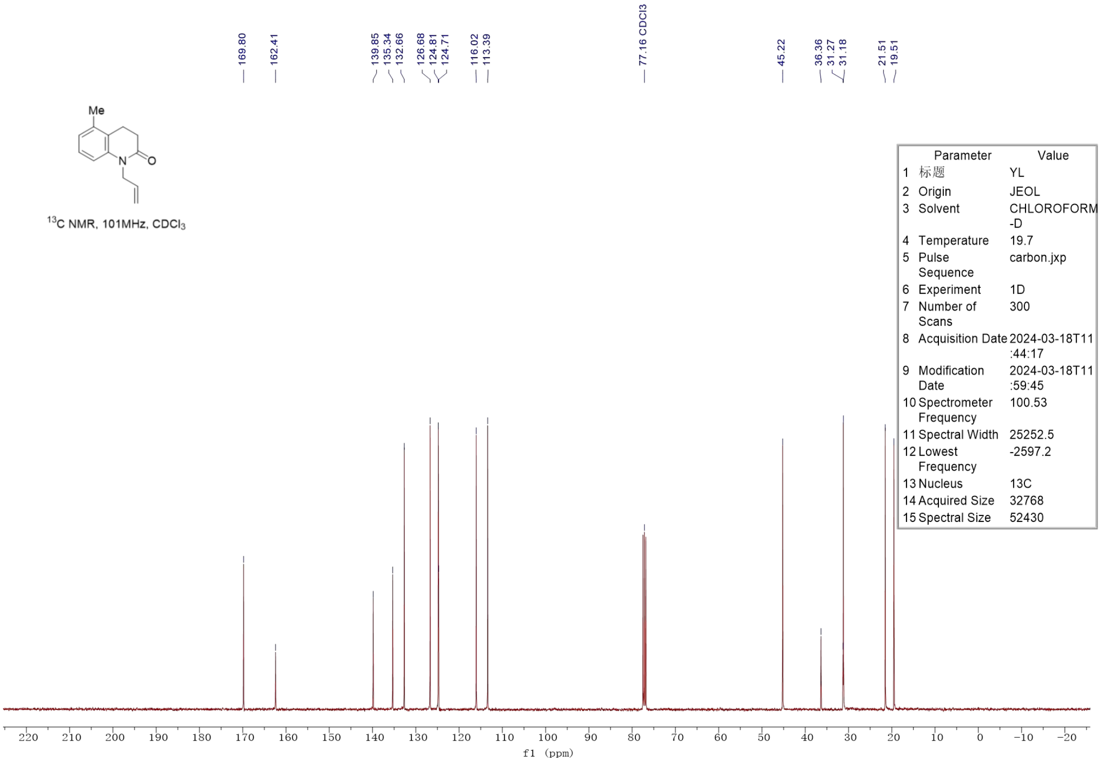

^{13}C NMR, 101MHz, $CDCl_3$

Parameter	Value
1 标题	YL
2 Origin	JEOL
3 Solvent	CHLOROFORM-D
4 Temperature	19.7
5 Pulse Sequence	carbon.jxp
6 Experiment	1D
7 Number of Scans	300
8 Acquisition Date	2024-03-18T11:44:17
9 Modification Date	2024-03-18T11:59:45
10 Spectrometer Frequency	100.53
11 Spectral Width	25252.5
12 Lowest Frequency	-2597.2
13 Nucleus	13C
14 Acquired Size	32768
15 Spectral Size	52430

4-allyl-2H-benzo[b][1,4]thiazin-3(4H)-one (S57)

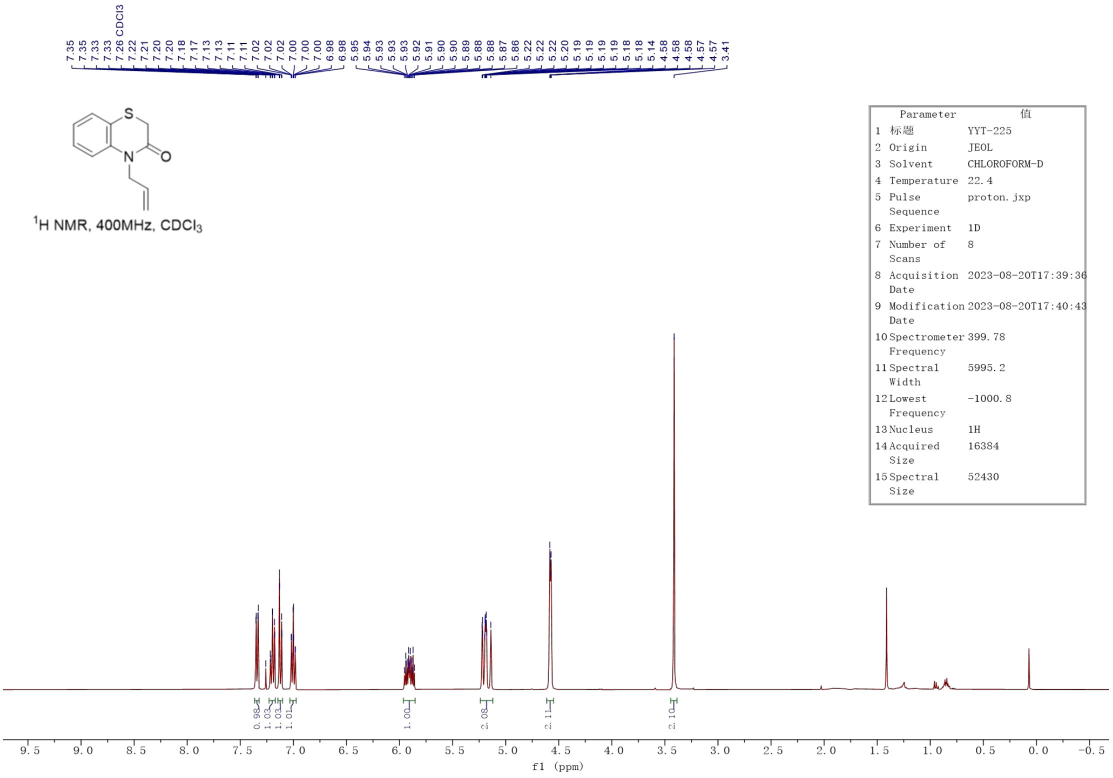

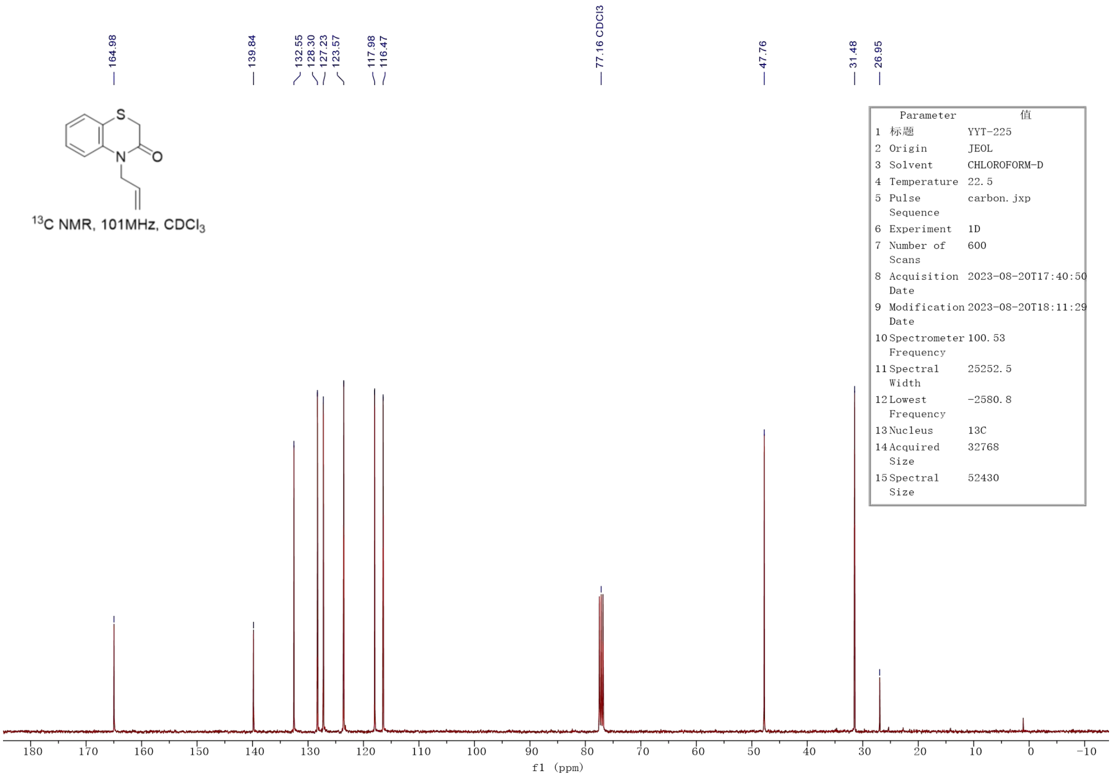

4-allyl-7-methyl-2H-benzo[b][1,4]thiazin-3(4H)-one (S58)

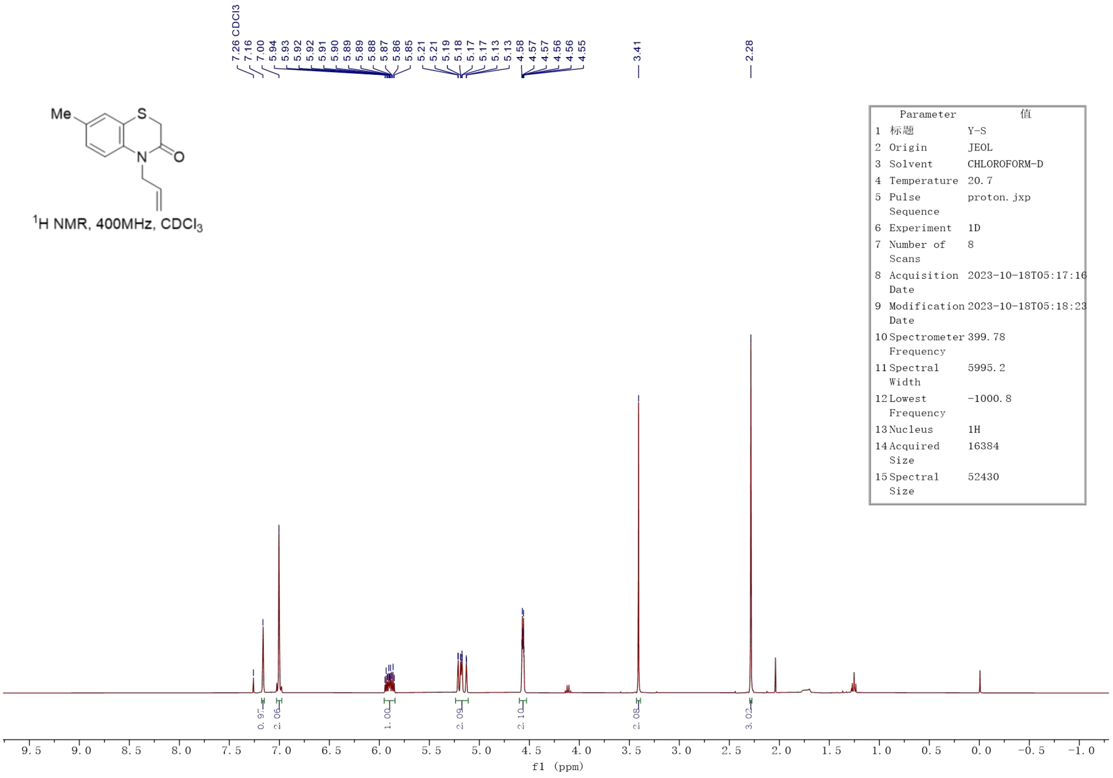

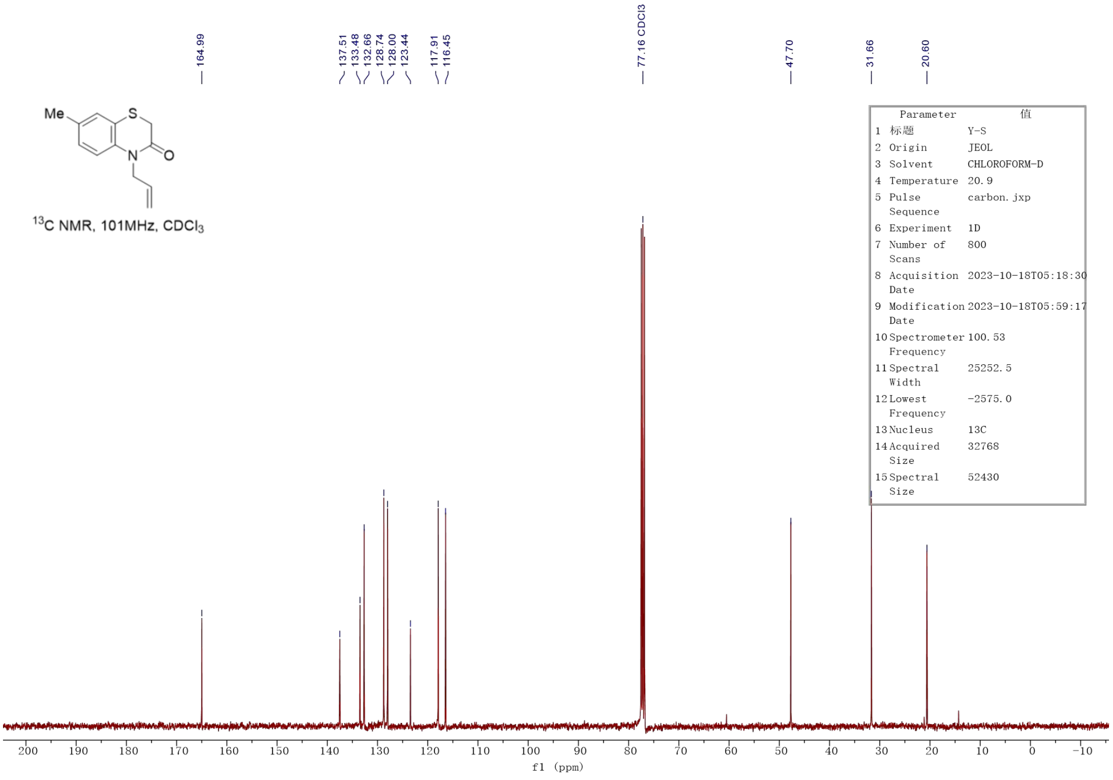

4-allyl-7-bromo-2H-benzo[b][1,4]thiazin-3(4H)-one (S59)

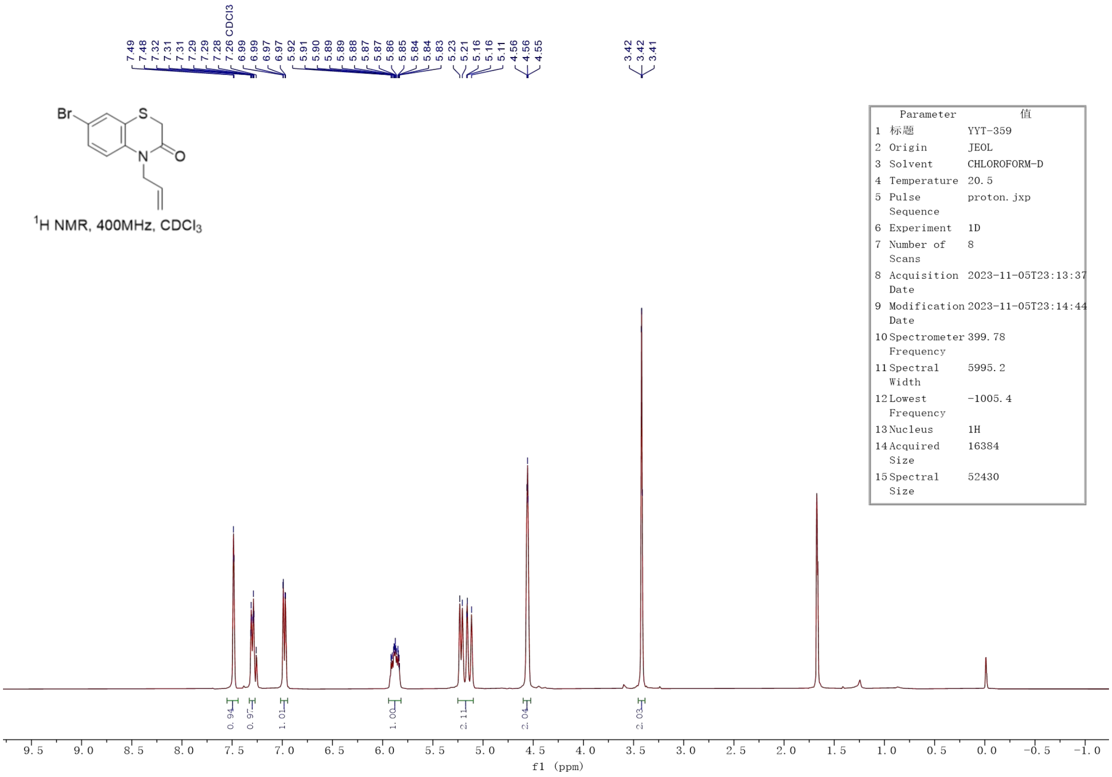

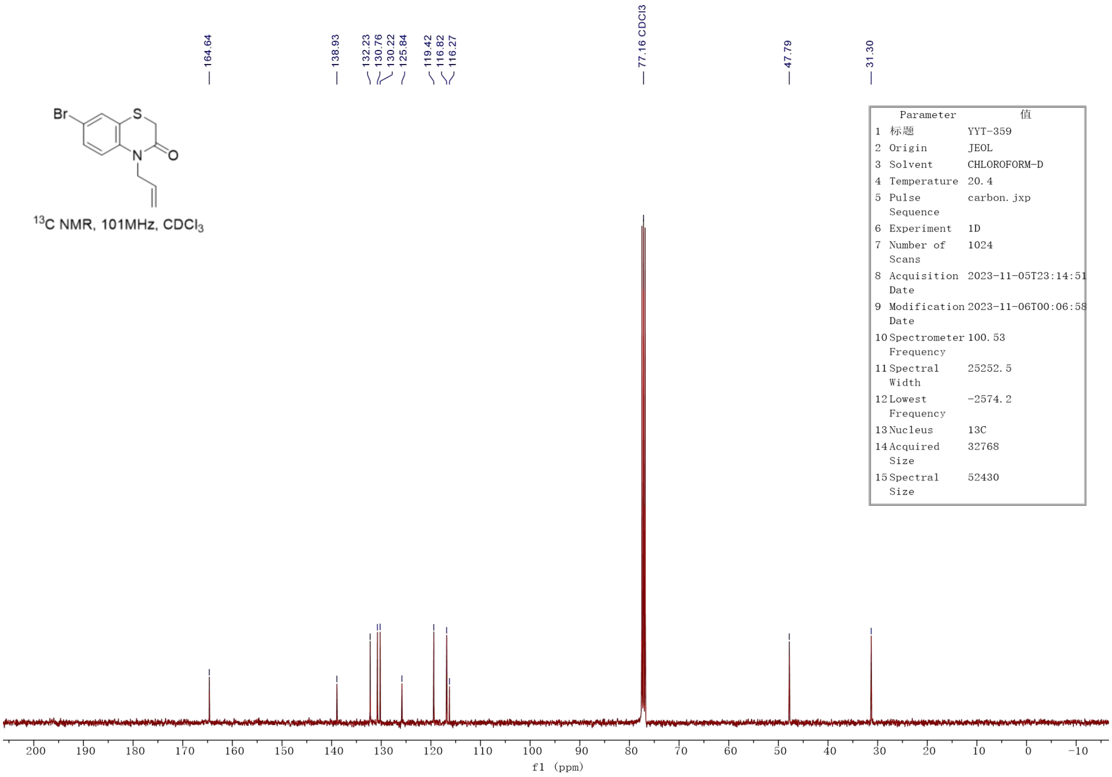

1-allyl-3,3-dimethylindolin-2-one (S60)

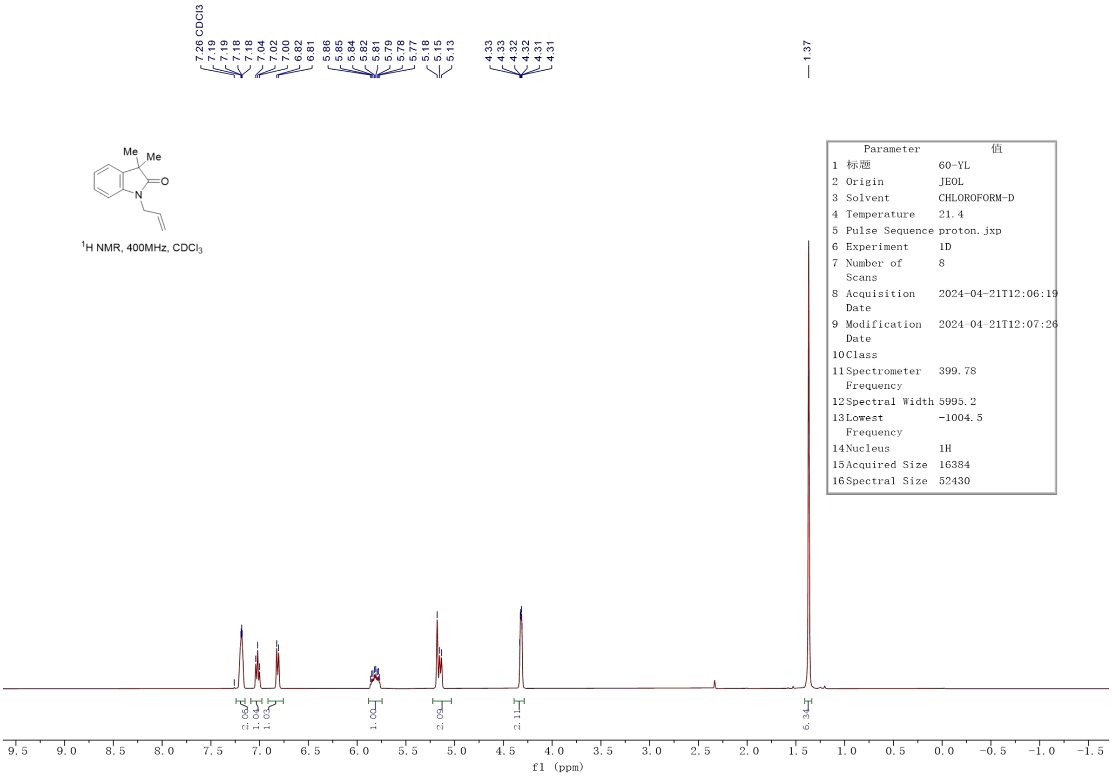

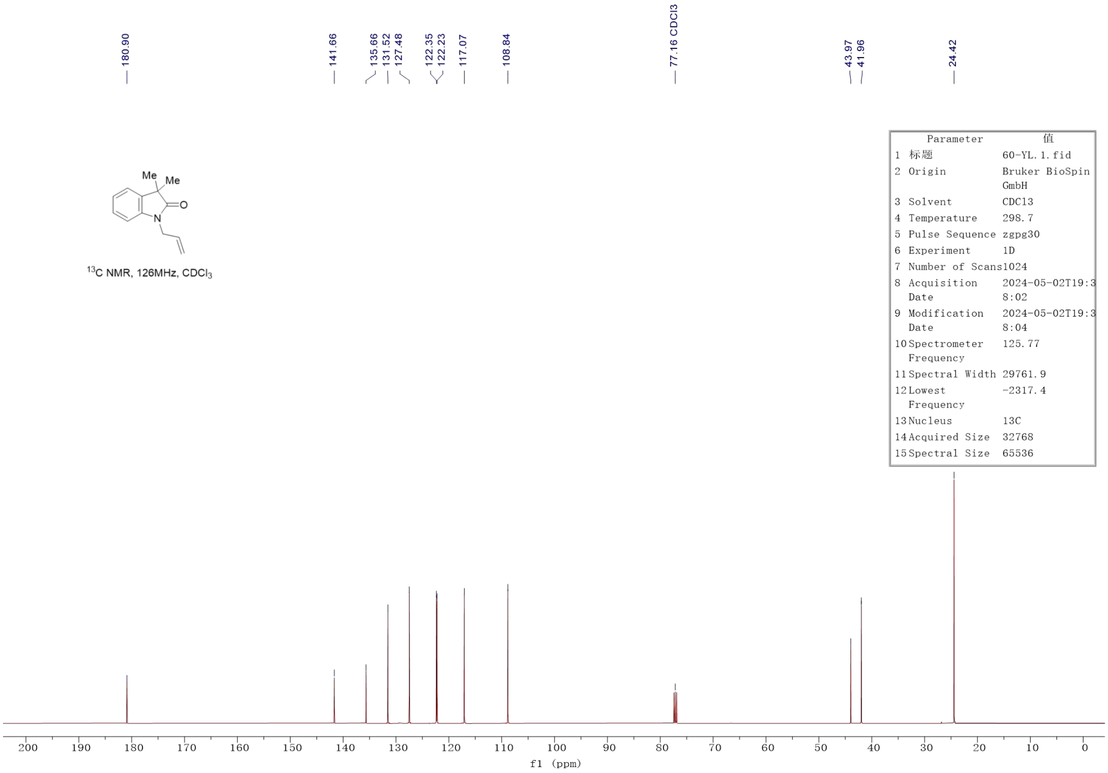

1-allylbenzo[cd]indol-2(1H)-one (S61)

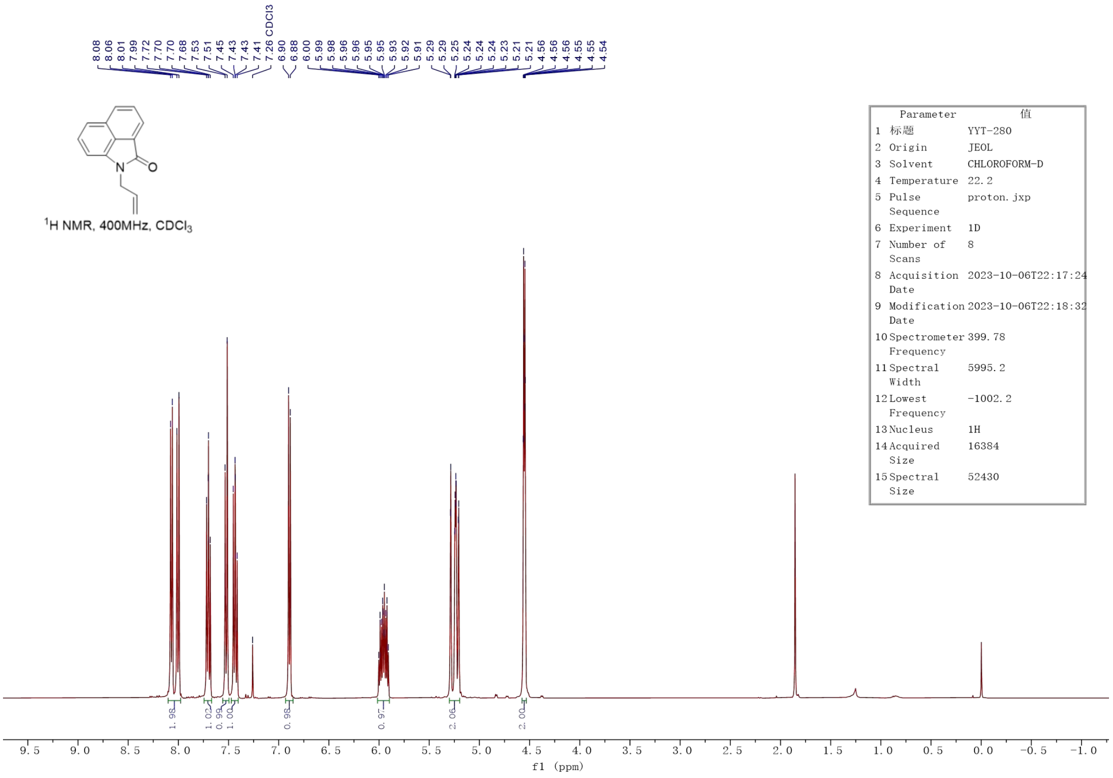

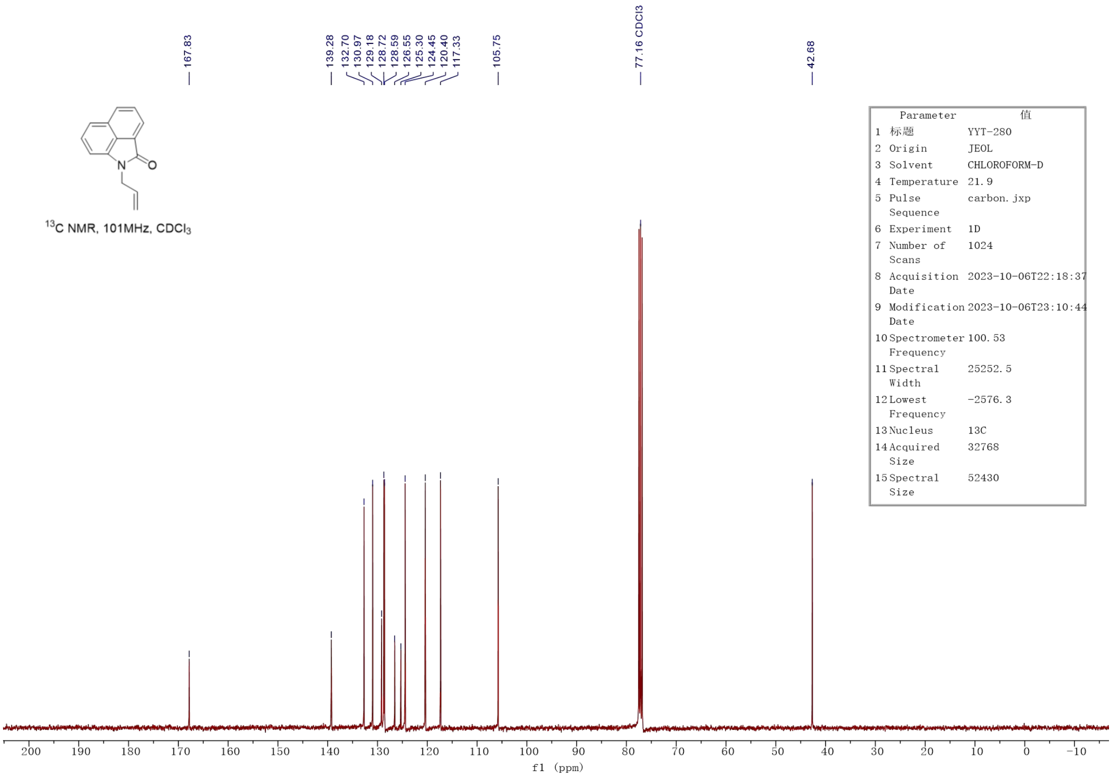

2-allyl-2H-naphtho[1,8-cd]isothiazole 1,1-dioxide (S62)

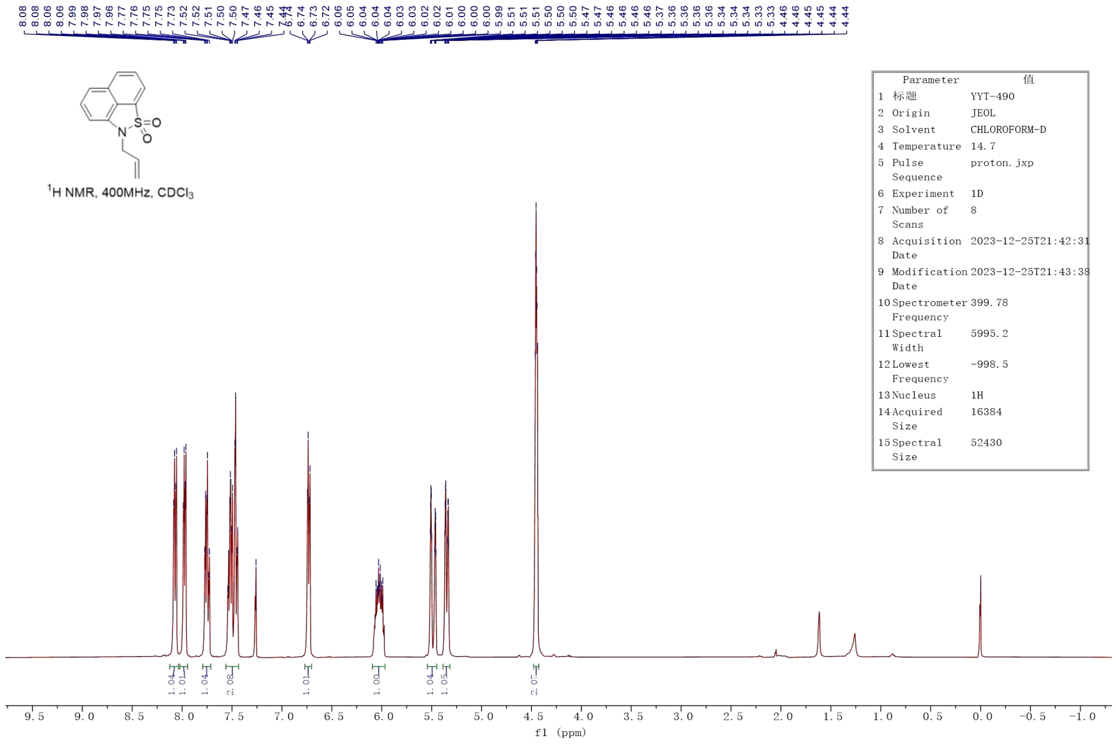

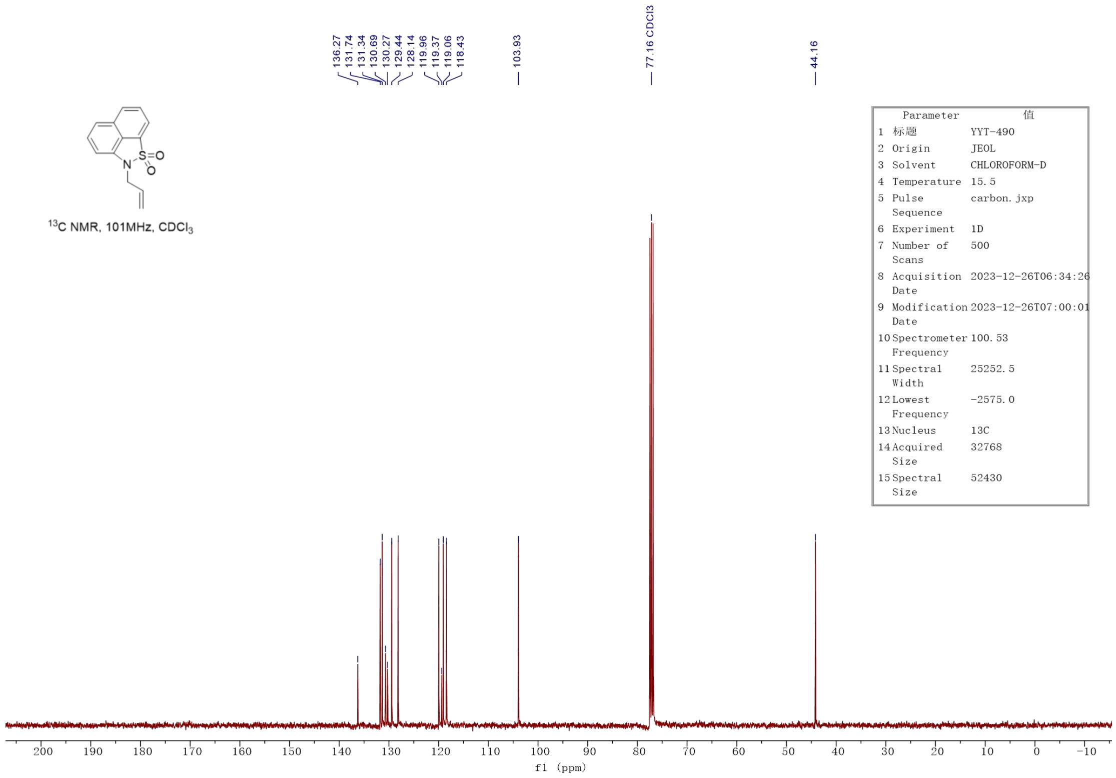

1-allyl-1,3,4,5-tetrahydro-2H-benzo[b]azepin-2-one (S63)

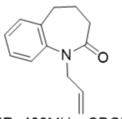

^{1}H NMR, 400MHz, $CDCl_3$

Parameter	值
1 标题	YYT-224
2 Origin	JEOL
3 Solvent	CHLOROFORM-D
4 Temperature	22.0
5 Pulse Sequence	proton.jxp
6 Experiment	1D
7 Number of Scans	8
8 Acquisition Date	2023-08-20T18:14:40
9 Modification Date	2023-08-20T18:15:48
10 Spectrometer Frequency	399.78
11 Spectral Width	5995.2
12 Lowest Frequency	-995.9
13 Nucleus	1H
14 Acquired Size	16384
15 Spectral Size	52430

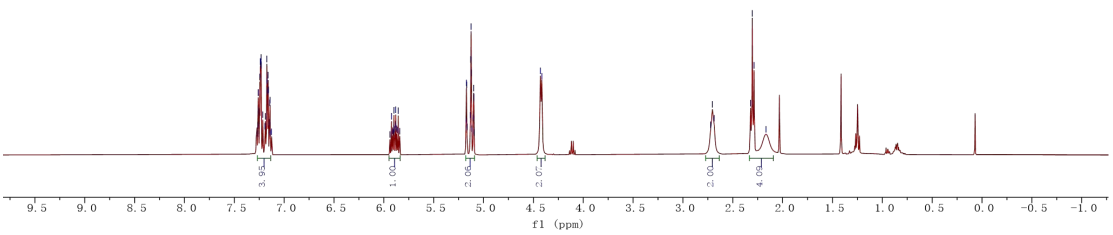

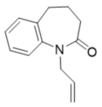

^{13}C NMR, 101MHz, $CDCl_3$

Parameter	值
1 标题	YYT-224
2 Origin	JEOL
3 Solvent	CHLOROFORM-D
4 Temperature	22.1
5 Pulse Sequence	carbon.jxp
6 Experiment	1D
7 Number of Scans	600
8 Acquisition Date	2023-08-20T18:15:53
9 Modification Date	2023-08-20T18:46:32
10 Spectrometer Frequency	100.53
11 Spectral Width	25252.5
12 Lowest Frequency	-2577.9
13 Nucleus	13C
14 Acquired Size	32768
15 Spectral Size	52430

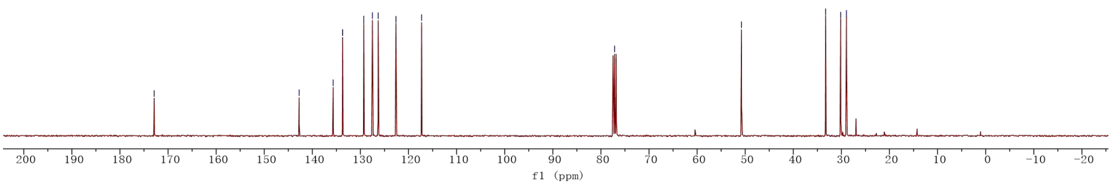

4-(2-methylallyl)-2H-benzo[b][1,4]oxazin-3(4H)-one (S64)

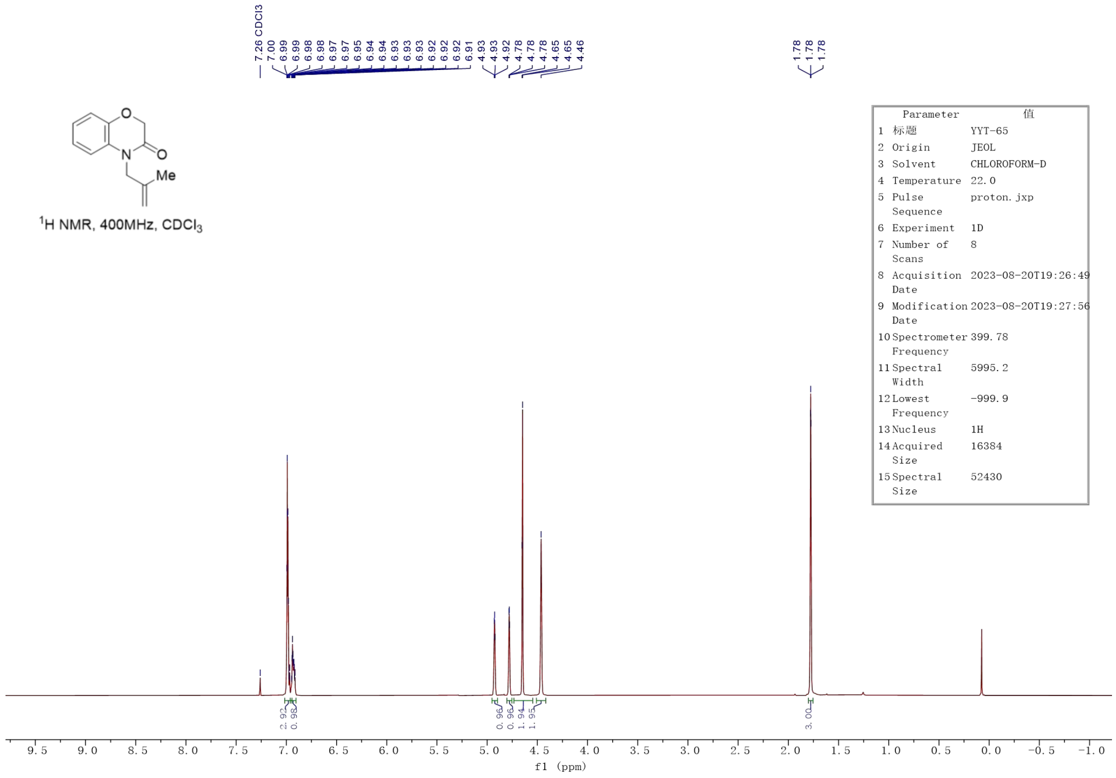

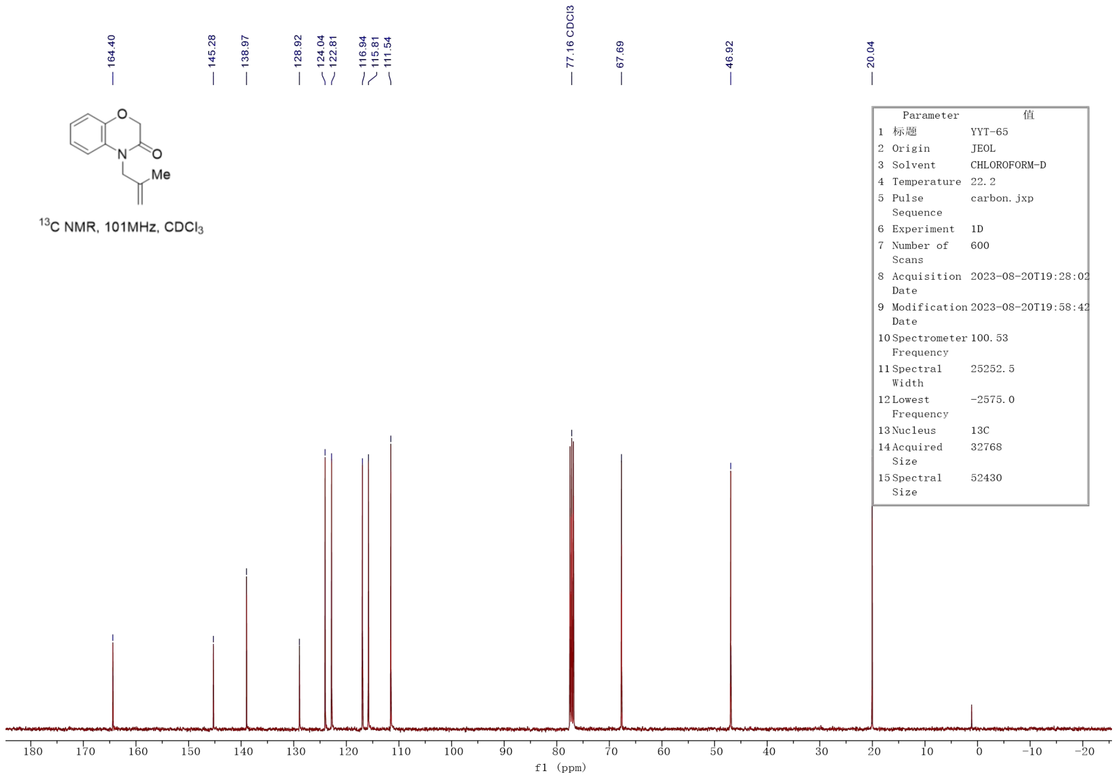

Methyl 2-((3-oxo-2,3-dihydro-4H-benzo[b][1,4]oxazin-4-yl)methyl)acrylate (S65)

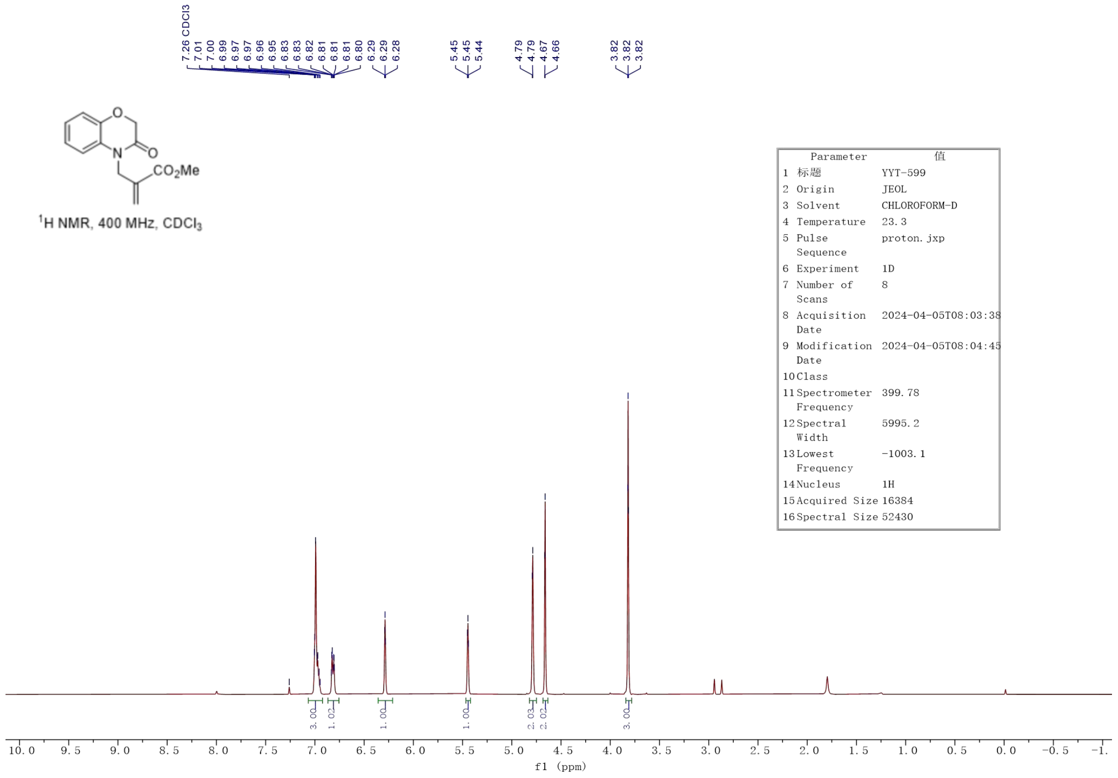

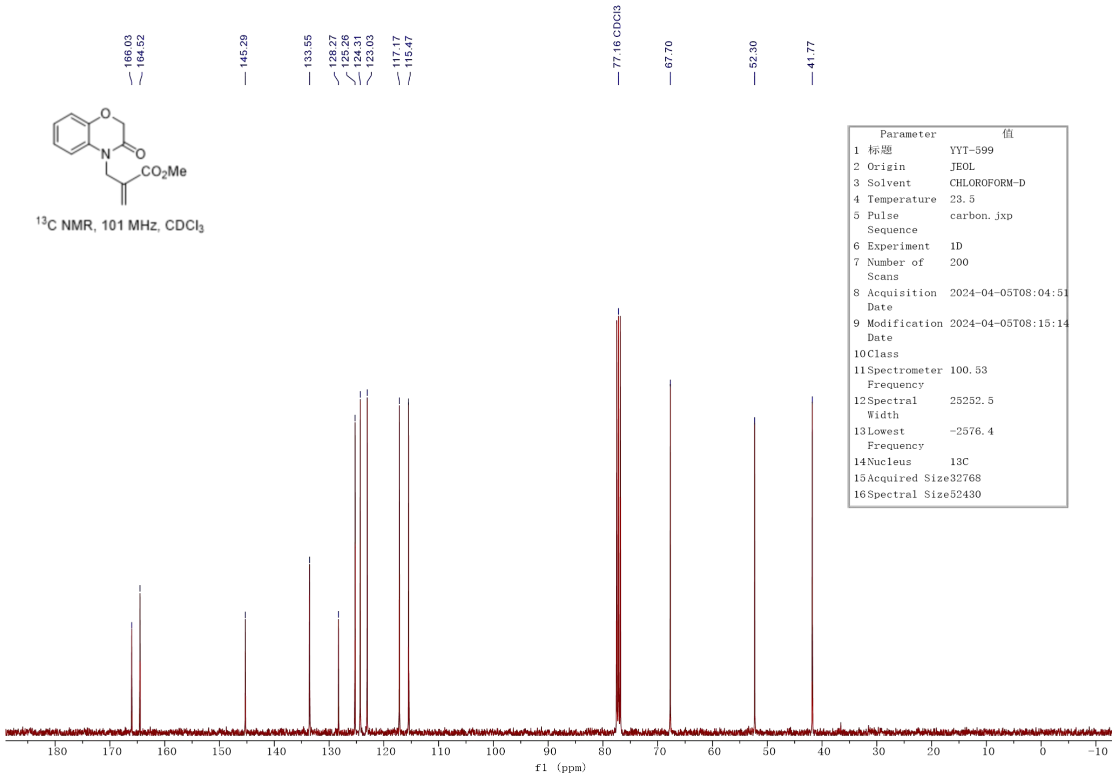

10-allyl-10H-phenoxazine--methane (1/1) (S66)

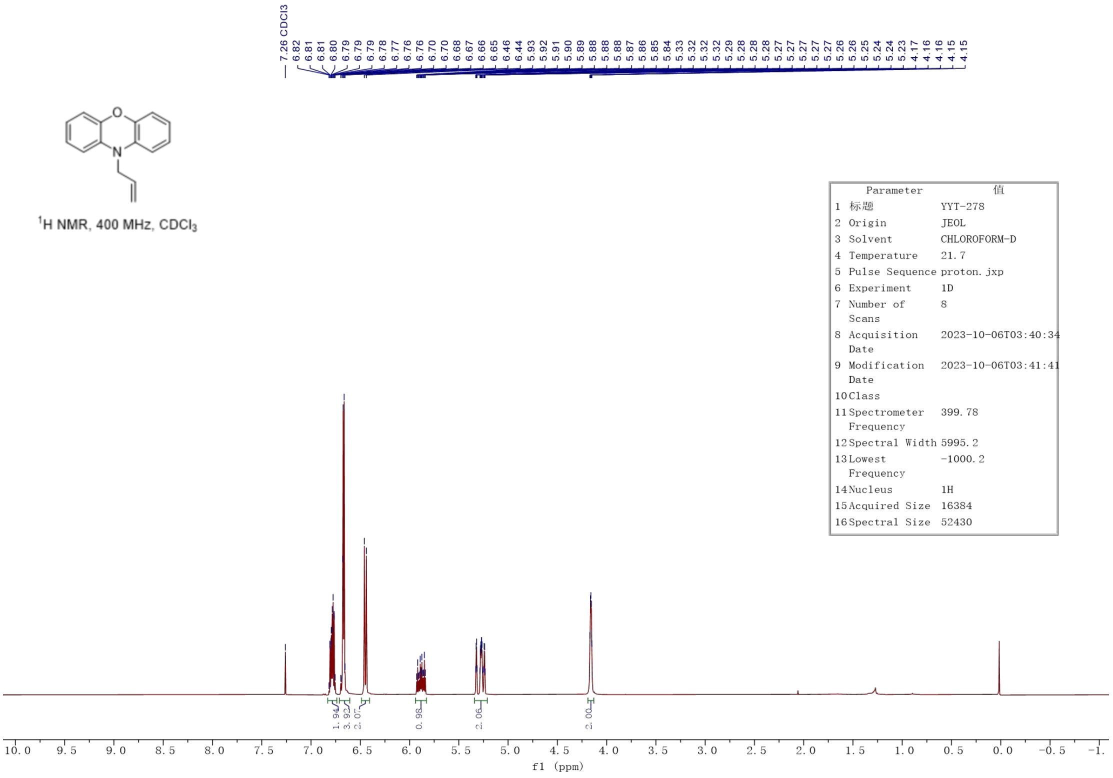

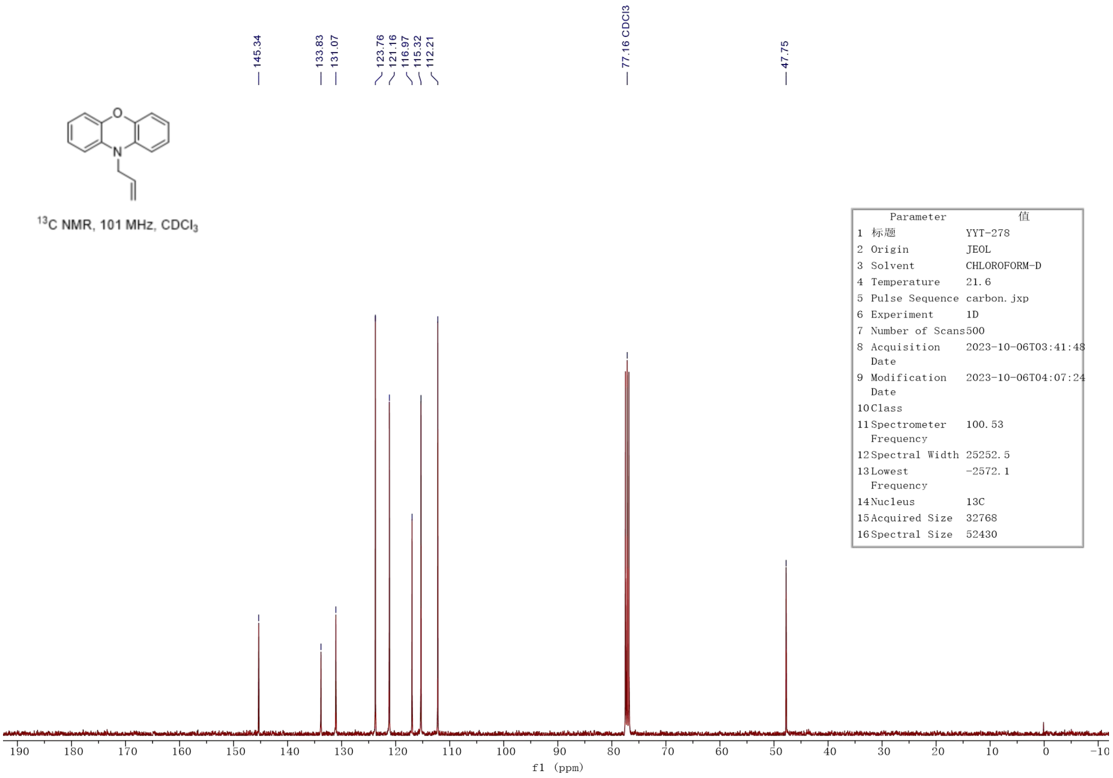

1-(10-allyl-10H-phenothiazin-2-yl)ethan-1-one (S67)

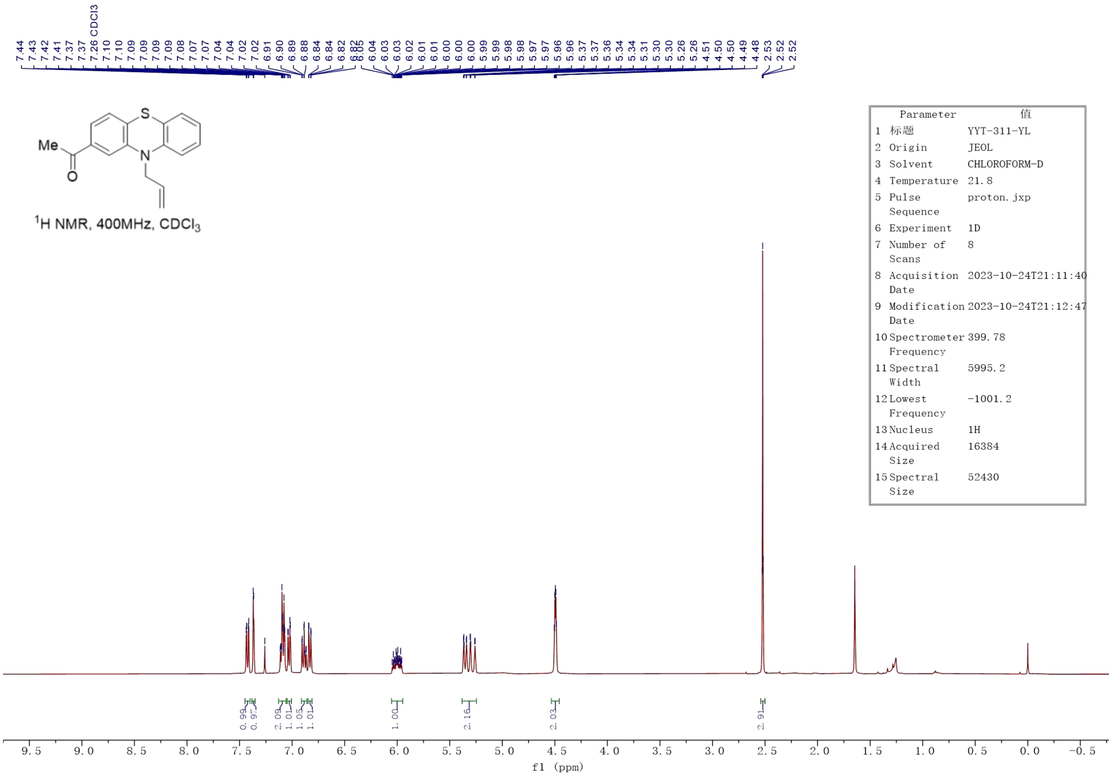

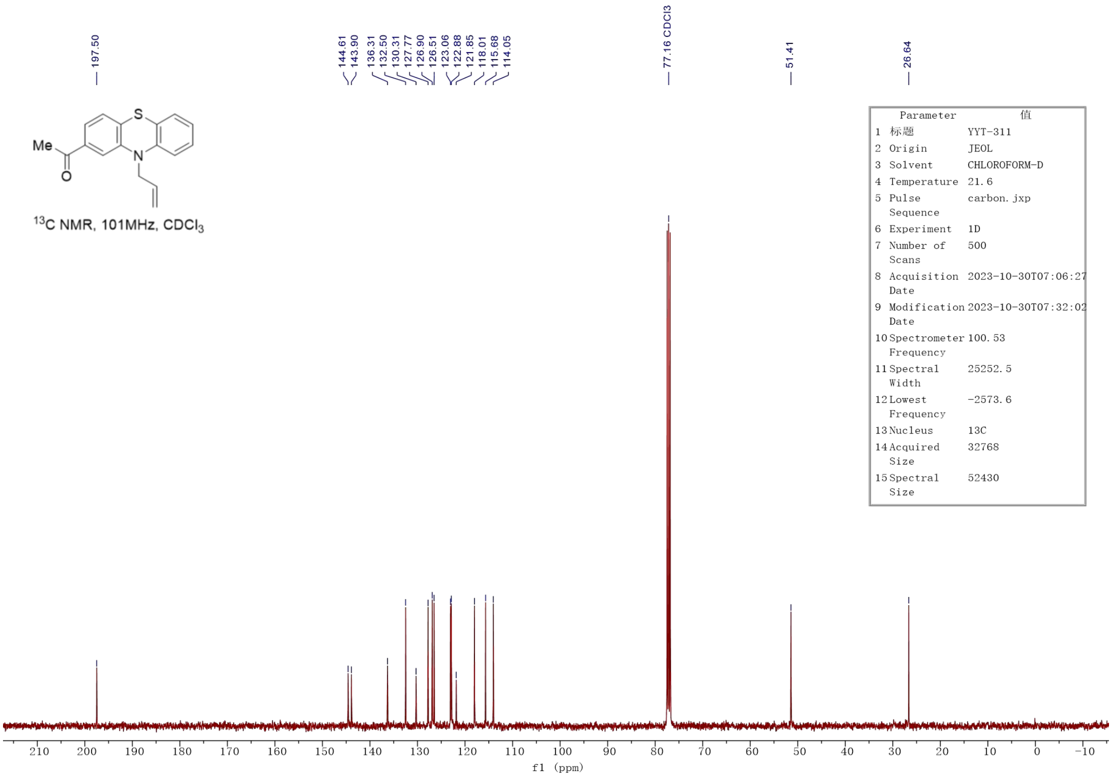

2-(allyloxy)pyrene (S80)

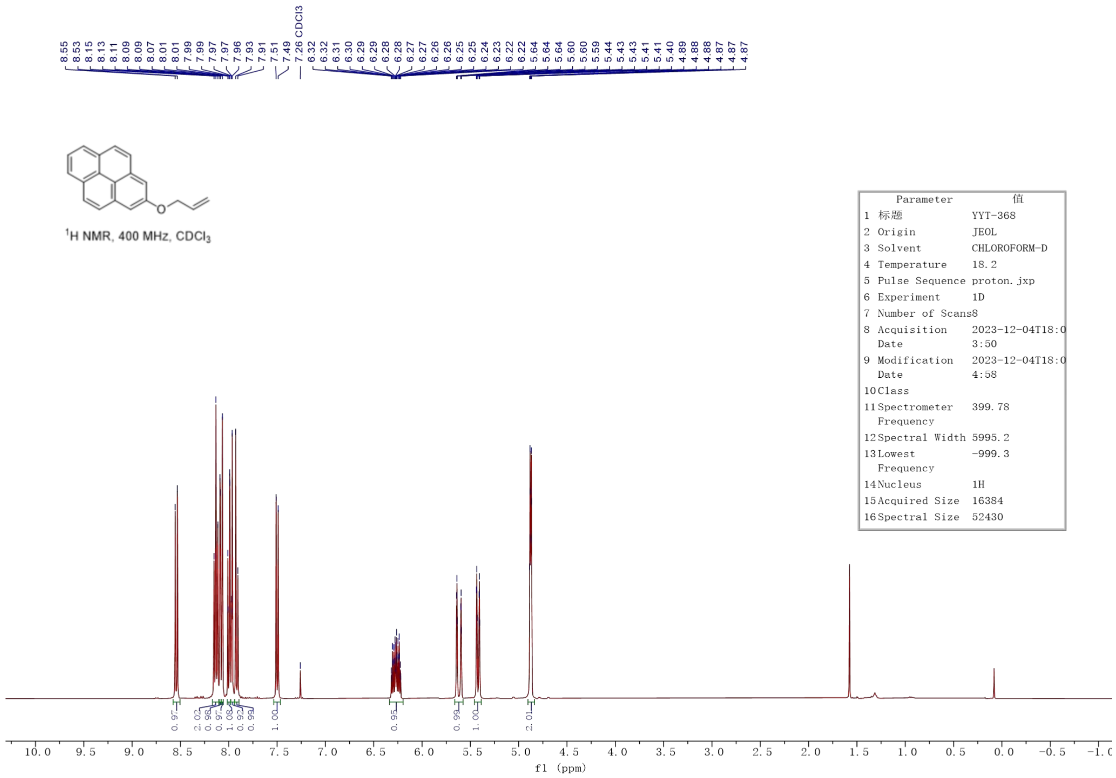

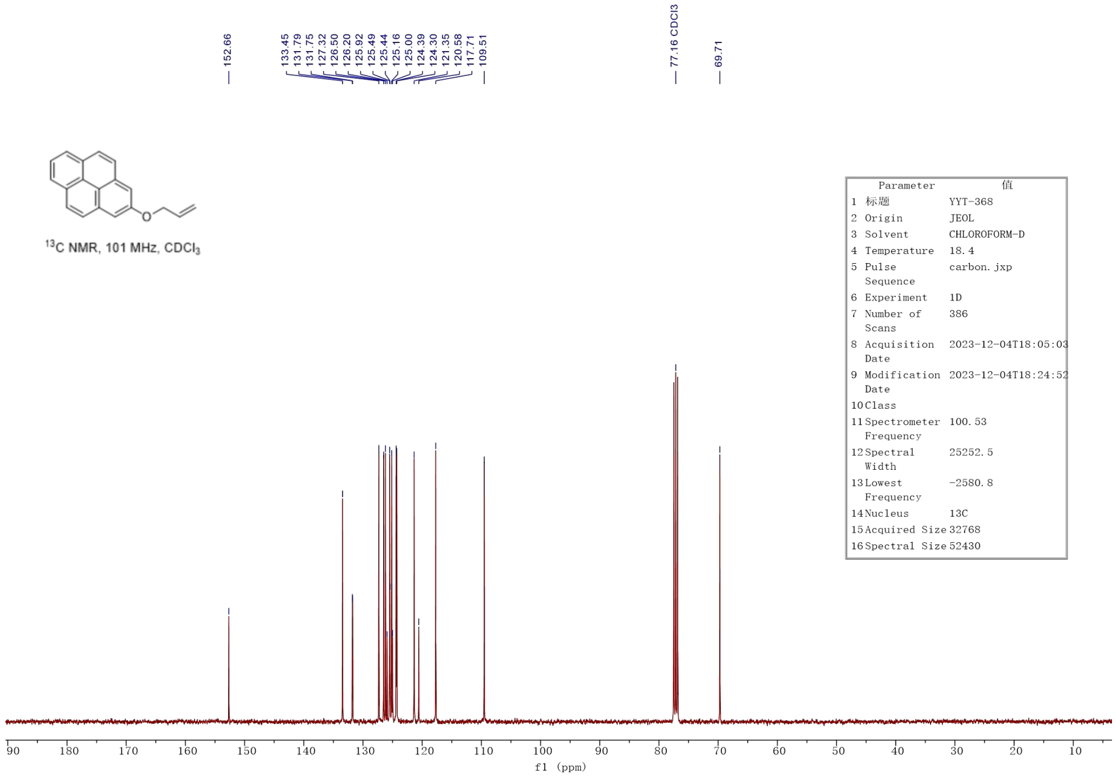

2-(allyloxy)dibenzo[b,d]furan (S83)

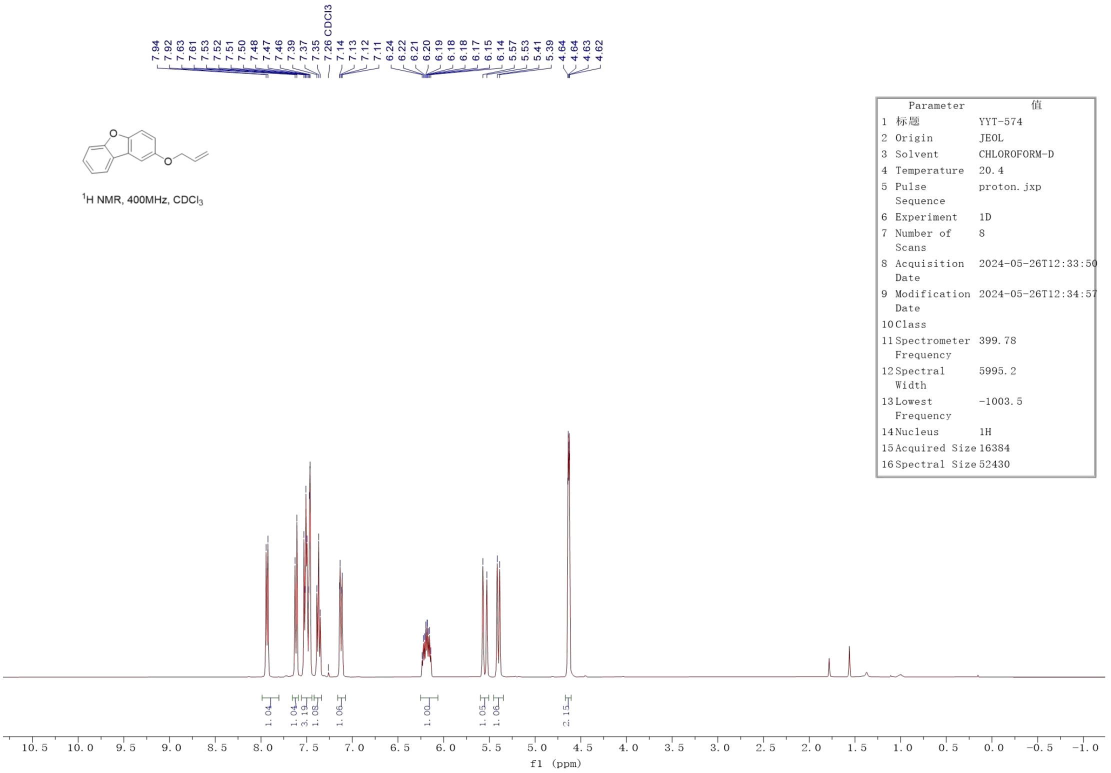

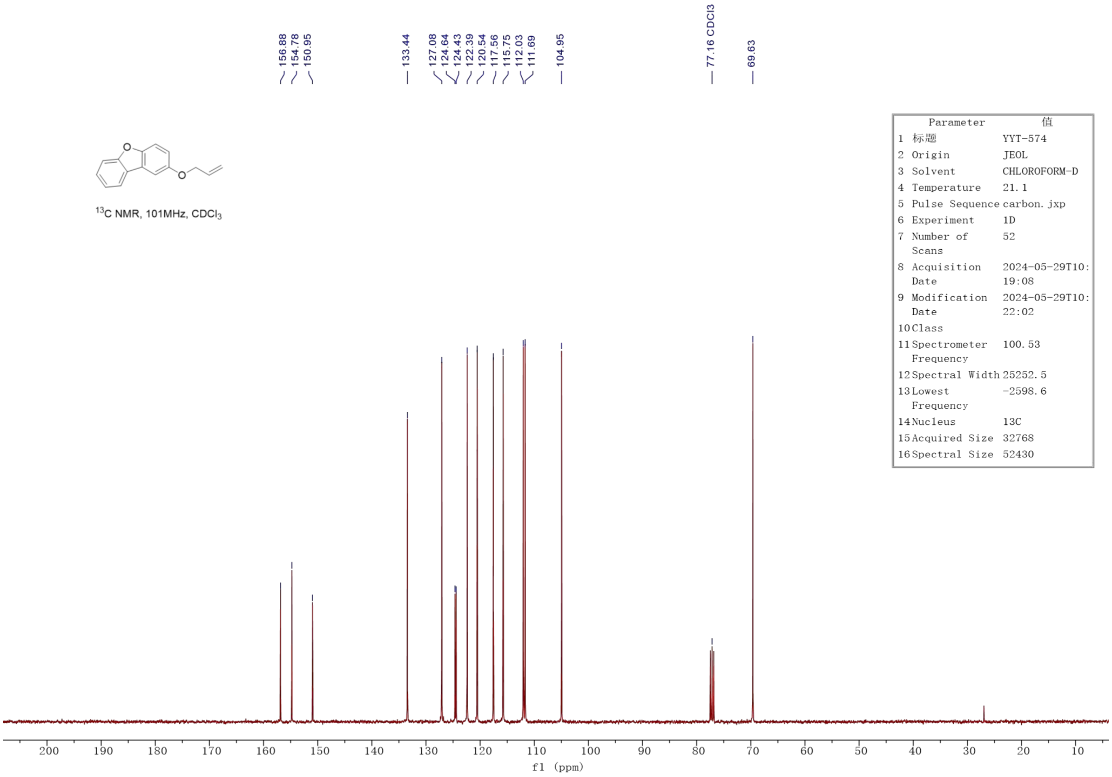

7-(allyloxy)-3-(4-methoxyphenyl)-4H-chromen-4-one (S87)

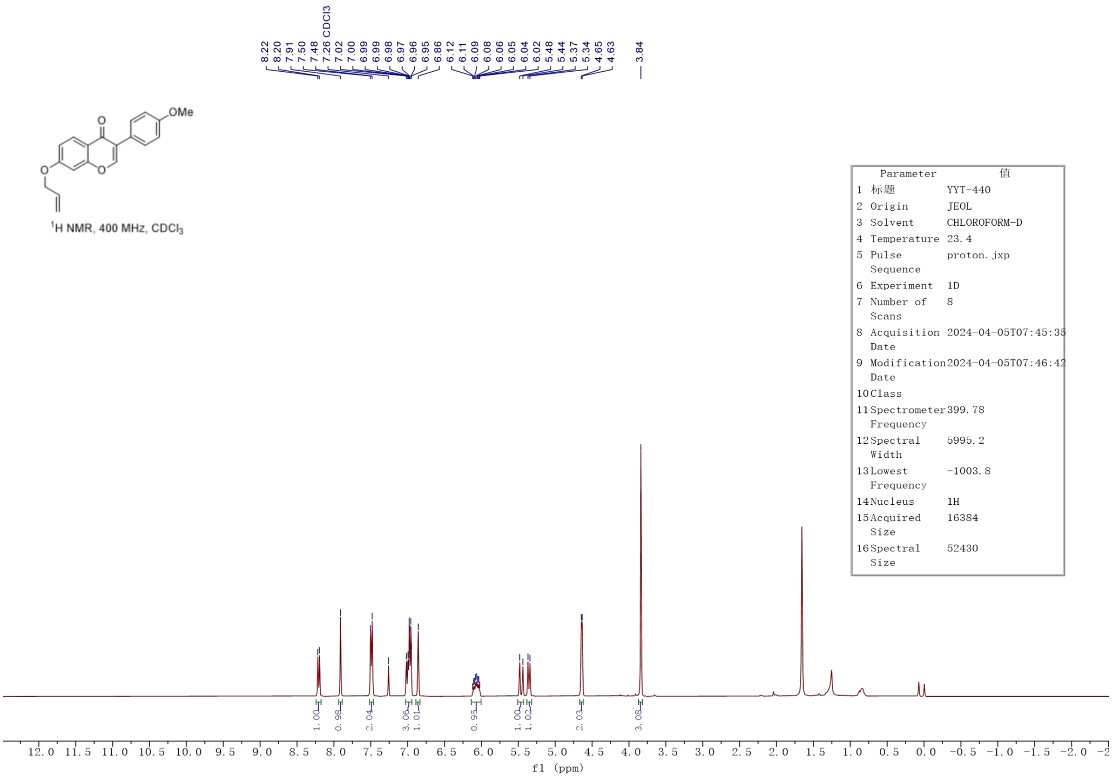

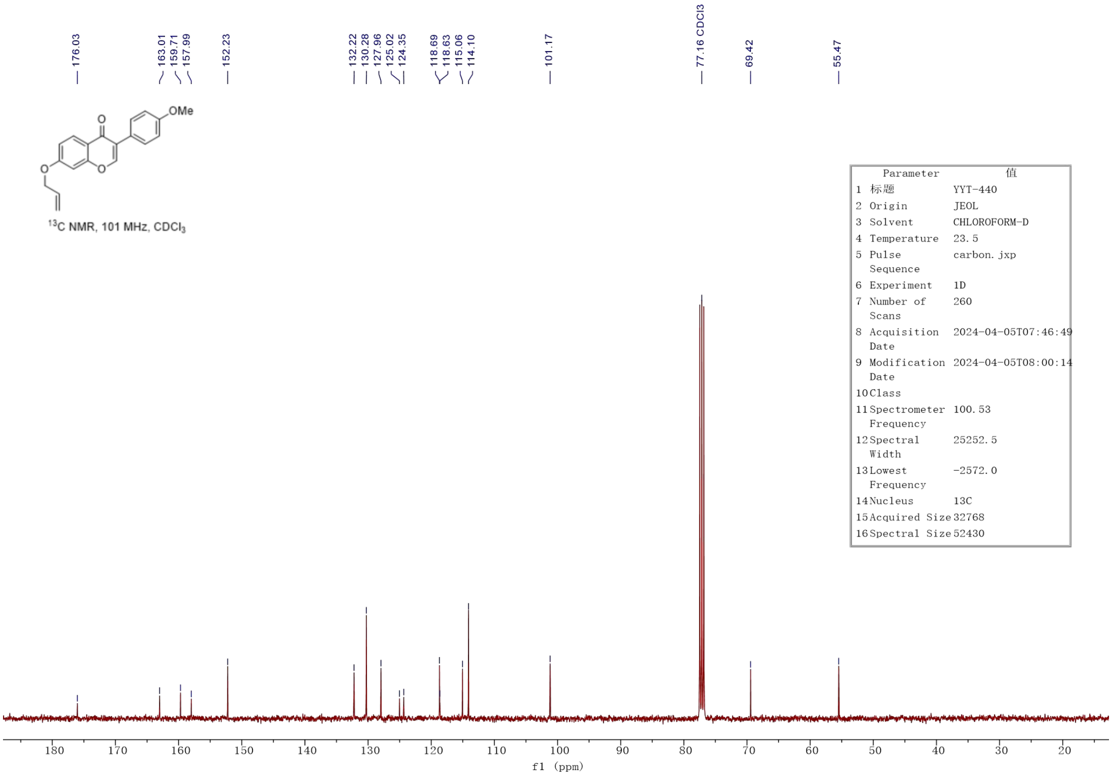

(3aR,5aS,11aS,11bS)-8-(allyloxy)-3a-methyl-1,2,3a,4,5,5a,6,11,11a,11b-decahydro-3H-cyclopenta[a]anthracen-3-one (S89)

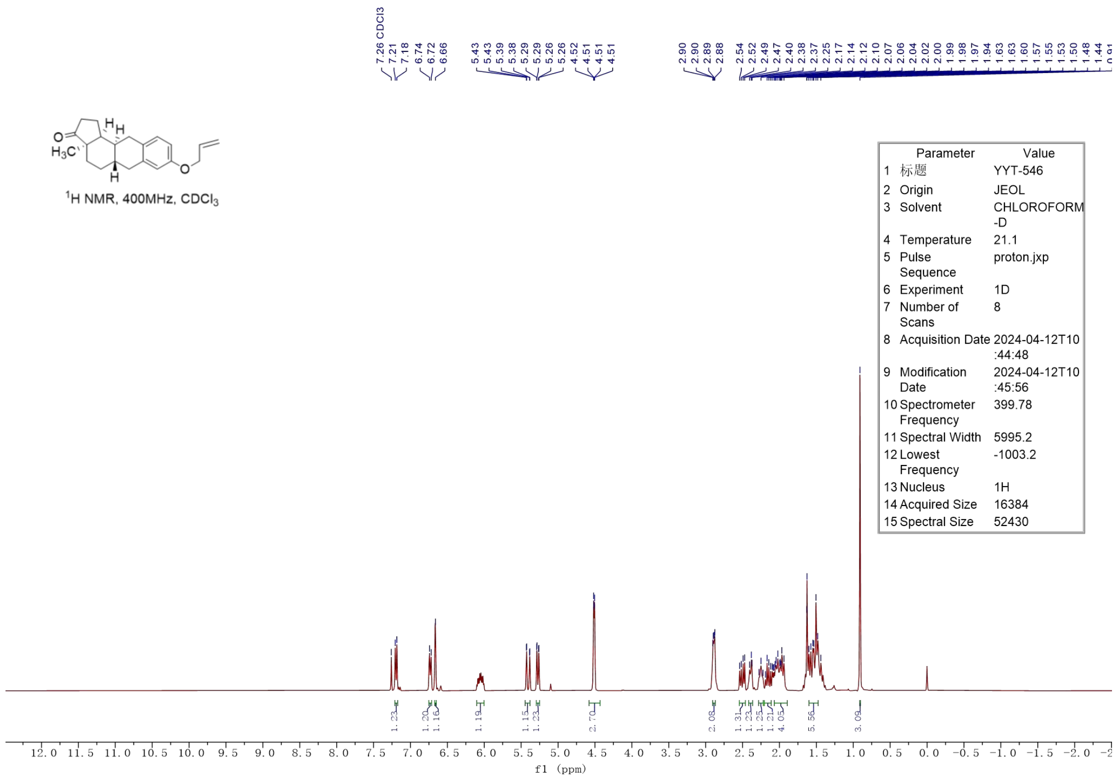

	Parameter	Value
1	标题	YYT-546
2	Origin	JEOL
3	Solvent	CHLOROFORM-D
4	Temperature	21.1
5	Pulse Sequence	proton.jxp
6	Experiment	1D
7	Number of Scans	8
8	Acquisition Date	2024-04-12T10:44:48
9	Modification Date	2024-04-12T10:45:56
10	Spectrometer Frequency	399.78
11	Spectral Width	5995.2
12	Lowest Frequency	-1003.2
13	Nucleus	1H
14	Acquired Size	16384
15	Spectral Size	52430

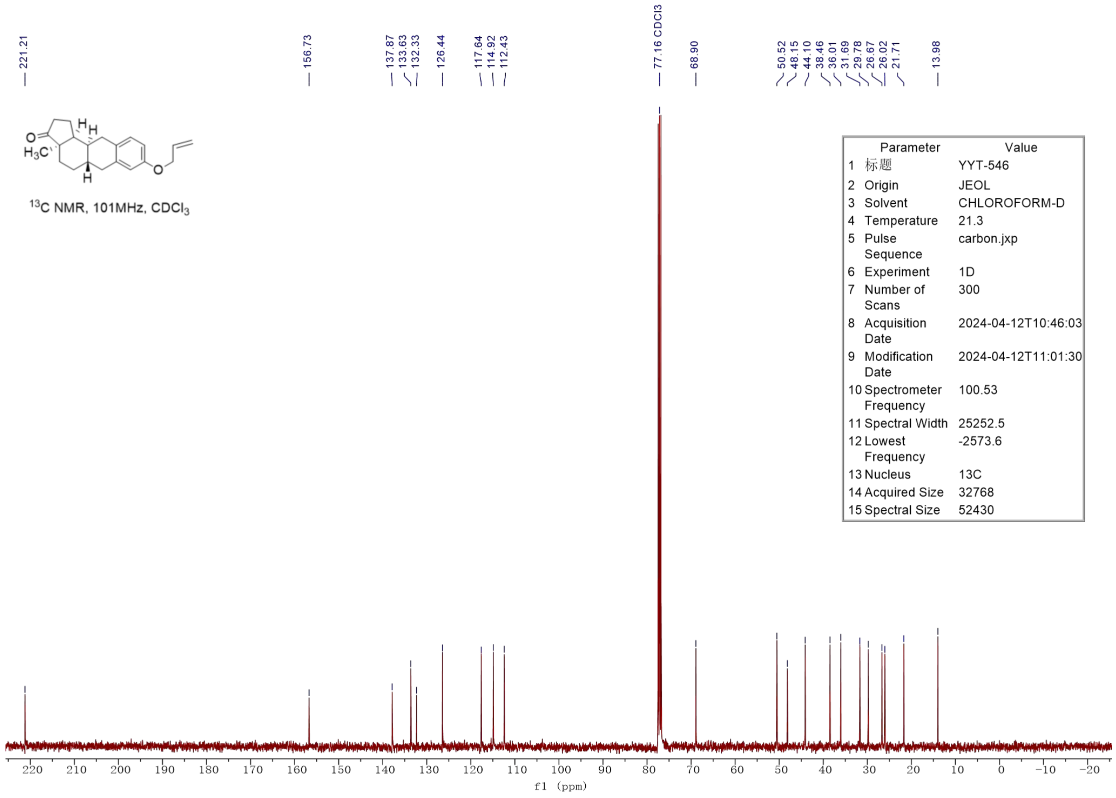

	Parameter	Value
1	标题	YYT-546
2	Origin	JEOL
3	Solvent	CHLOROFORM-D
4	Temperature	21.3
5	Pulse Sequence	carbon.jxp
6	Experiment	1D
7	Number of Scans	300
8	Acquisition Date	2024-04-12T10:46:03
9	Modification Date	2024-04-12T11:01:30
10	Spectrometer Frequency	100.53
11	Spectral Width	25252.5
12	Lowest Frequency	-2573.6
13	Nucleus	13C
14	Acquired Size	32768
15	Spectral Size	52430

2-(allyloxy)-13-methyl-7,8,9,11,12,13,14,15,16,17-decahydro-6H-cyclopenta[a]phenanthren-17-yl pentanoate (S90)

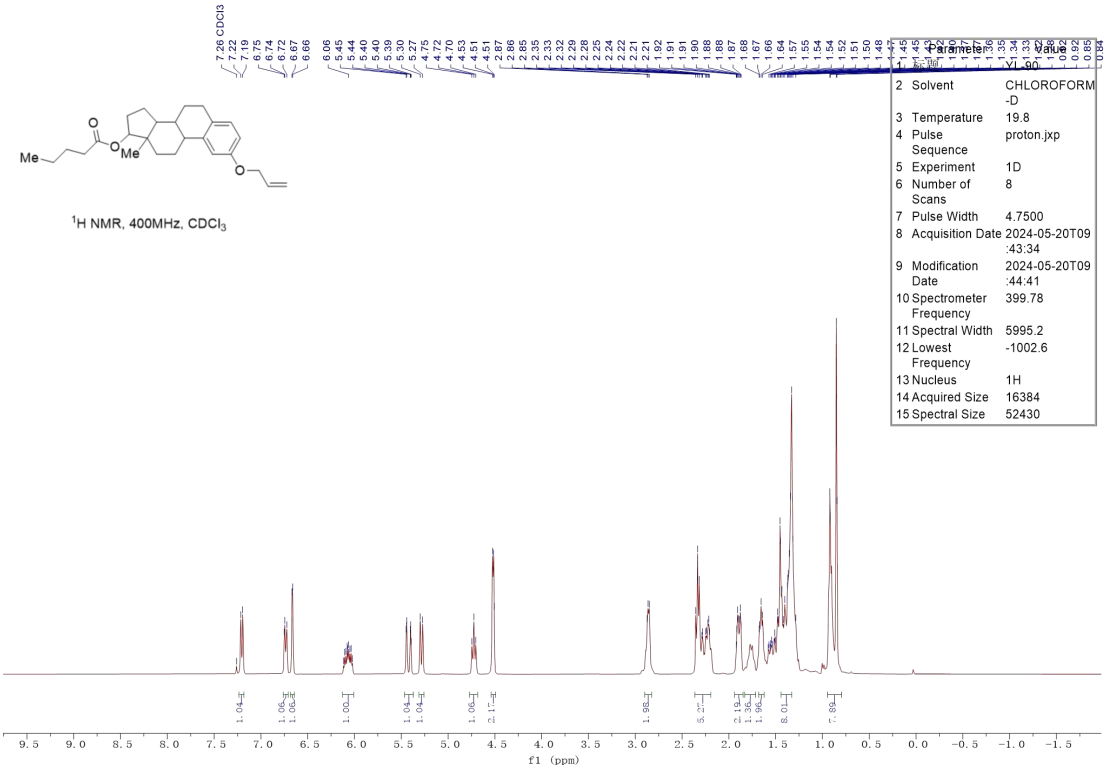

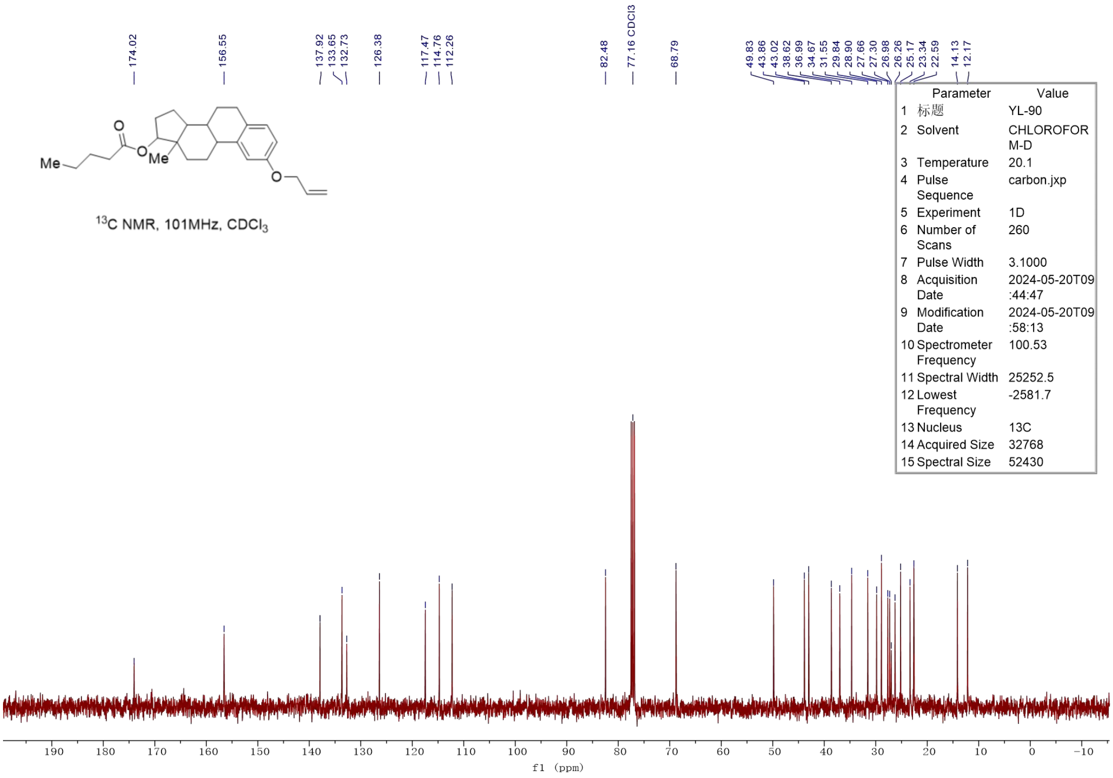

4-(but-3-en-1-yl)-2H-benzo[b][1,4]oxazin-3(4H)-one (S92)

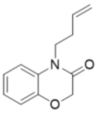

^{1}H NMR, 400MHz, $CDCl_3$

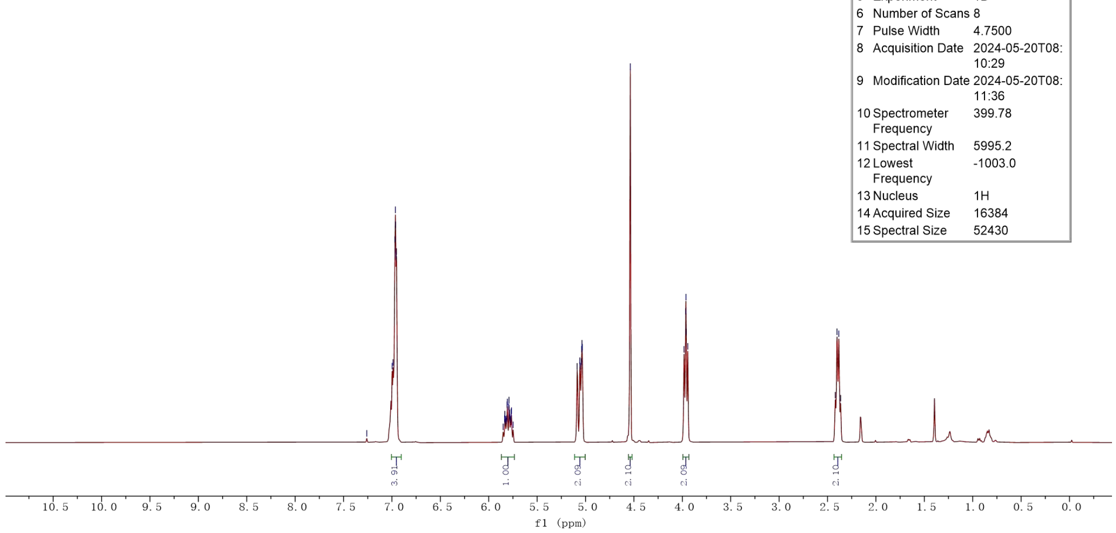

	Parameter	Value
1	标题	YL-4C
2	Solvent	CHLOROFORM-D
3	Temperature	20.0
4	Pulse Sequence	proton.jxp
5	Experiment	1D
6	Number of Scans	8
7	Pulse Width	4.7500
8	Acquisition Date	2024-05-20T08:10:29
9	Modification Date	2024-05-20T08:11:36
10	Spectrometer Frequency	399.78
11	Spectral Width	5995.2
12	Lowest Frequency	-1003.0
13	Nucleus	1H
14	Acquired Size	16384
15	Spectral Size	52430

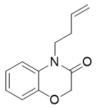

^{13}C NMR, 101MHz, $CDCl_3$

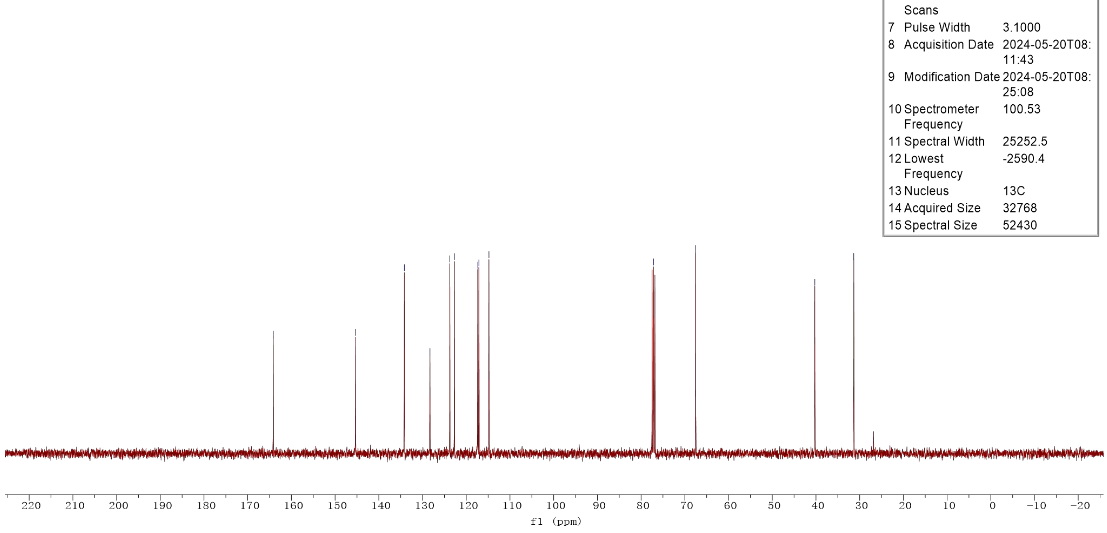

	Parameter	Value
1	标题	YL-4C
2	Solvent	CHLOROFORM-D
3	Temperature	20.1
4	Pulse Sequence	carbon.jxp
5	Experiment	1D
6	Number of Scans	260
7	Pulse Width	3.1000
8	Acquisition Date	2024-05-20T08:11:43
9	Modification Date	2024-05-20T08:25:08
10	Spectrometer Frequency	100.53
11	Spectral Width	25252.5
12	Lowest Frequency	-2590.4
13	Nucleus	13C
14	Acquired Size	32768
15	Spectral Size	52430

4-(hex-5-en-1-yl)-2H-benzo[b][1,4]oxazin-3(4H)-one(S93)

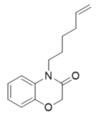

^{1}H NMR, 400MHz, $CDCl_3$

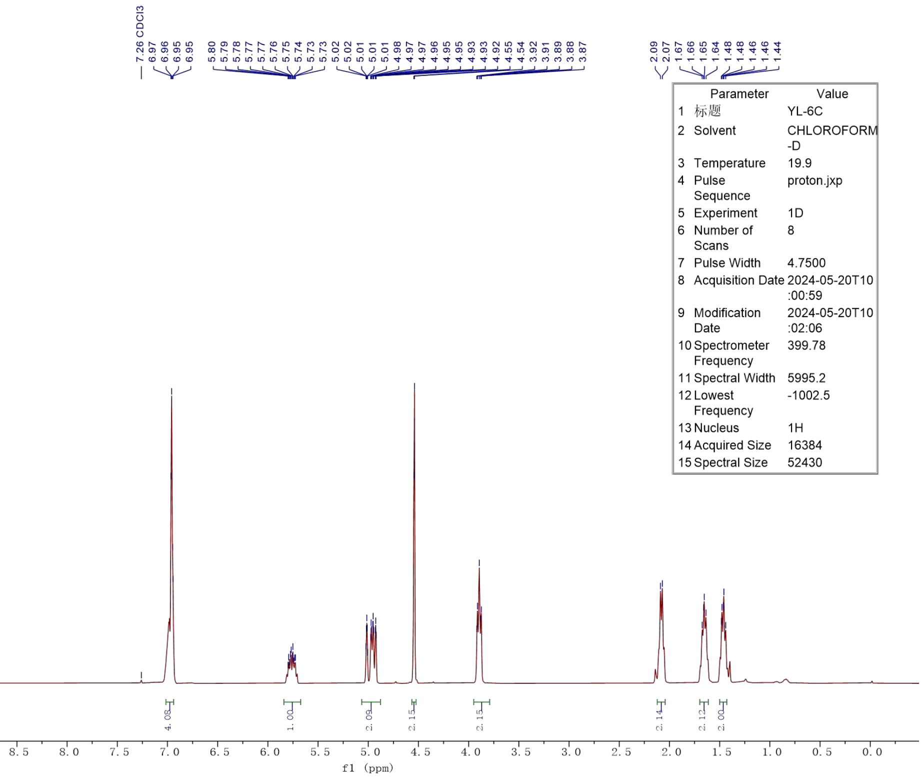

	Parameter	Value
1	标题	YL-6C
2	Solvent	CHLOROFORM-D
3	Temperature	19.9
4	Pulse Sequence	proton.jxp
5	Experiment	1D
6	Number of Scans	8
7	Pulse Width	4.7500
8	Acquisition Date	2024-05-20T10:00:59
9	Modification Date	2024-05-20T10:02:06
10	Spectrometer Frequency	399.78
11	Spectral Width	5995.2
12	Lowest Frequency	-1002.5
13	Nucleus	1H
14	Acquired Size	16384
15	Spectral Size	52430

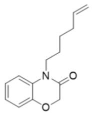

^{13}C NMR, 101MHz, $CDCl_3$

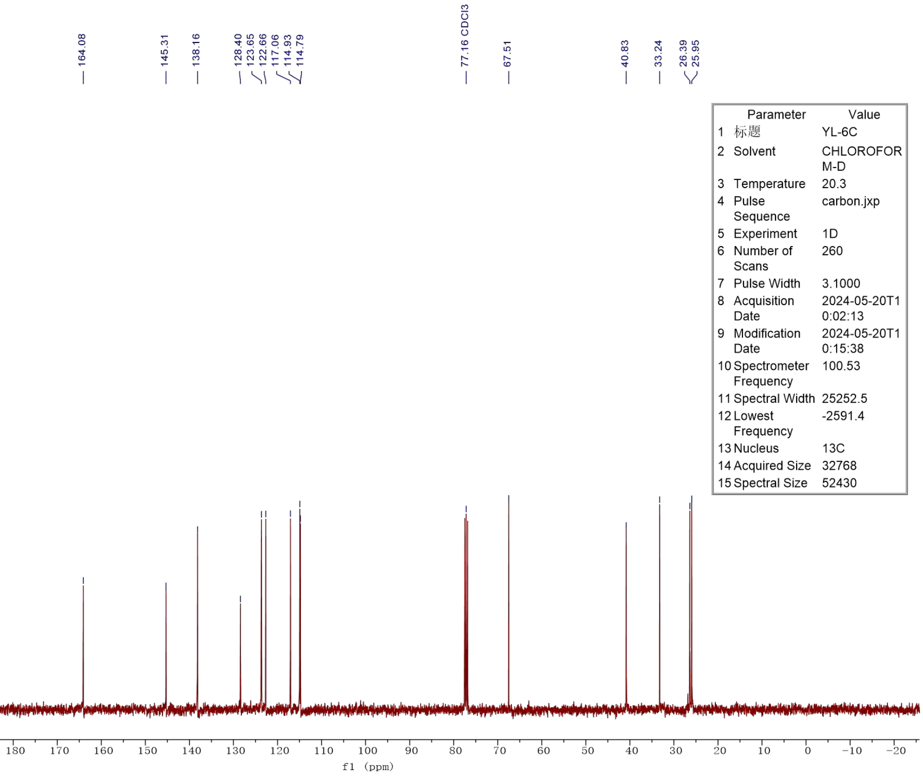

	Parameter	Value
1	标题	YL-6C
2	Solvent	CHLOROFORM-D
3	Temperature	20.3
4	Pulse Sequence	carbon.jxp
5	Experiment	1D
6	Number of Scans	260
7	Pulse Width	3.1000
8	Acquisition Date	2024-05-20T10:02:13
9	Modification Date	2024-05-20T10:15:38
10	Spectrometer Frequency	100.53
11	Spectral Width	25252.5
12	Lowest Frequency	-2591.4
13	Nucleus	13C
14	Acquired Size	32768
15	Spectral Size	52430

7-(p-tolyl)-6,7-dihydro-5*H*-[1,4]oxazino[2,3,4-ij]quinolin-3(2*H*)-one (3)

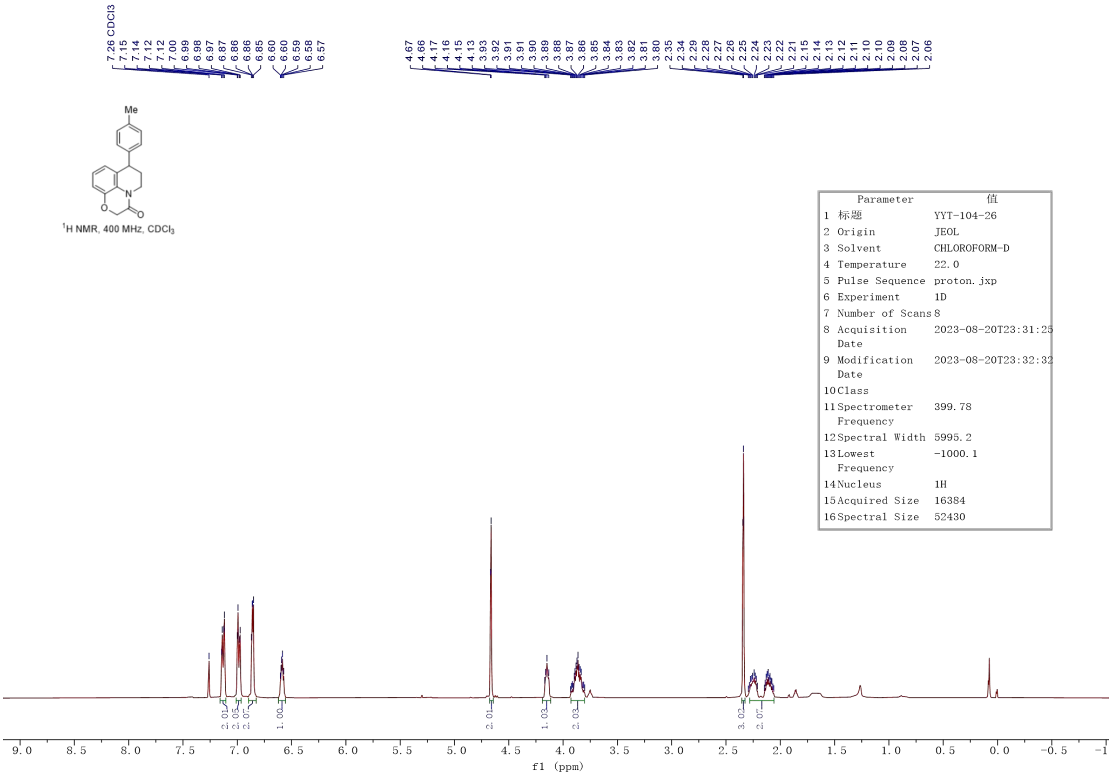

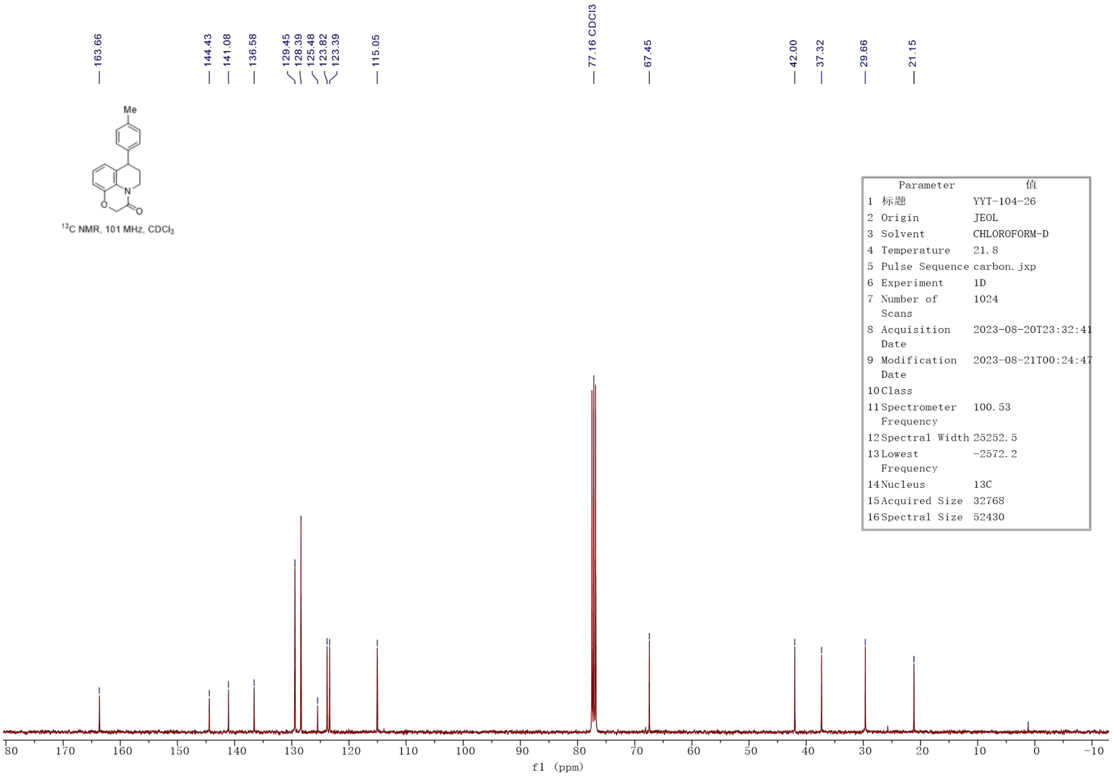

(E)-4-(3-(p-tolyl)allyl)-2H-benzo[b][1,4]oxazin-3(4H)-one (4)

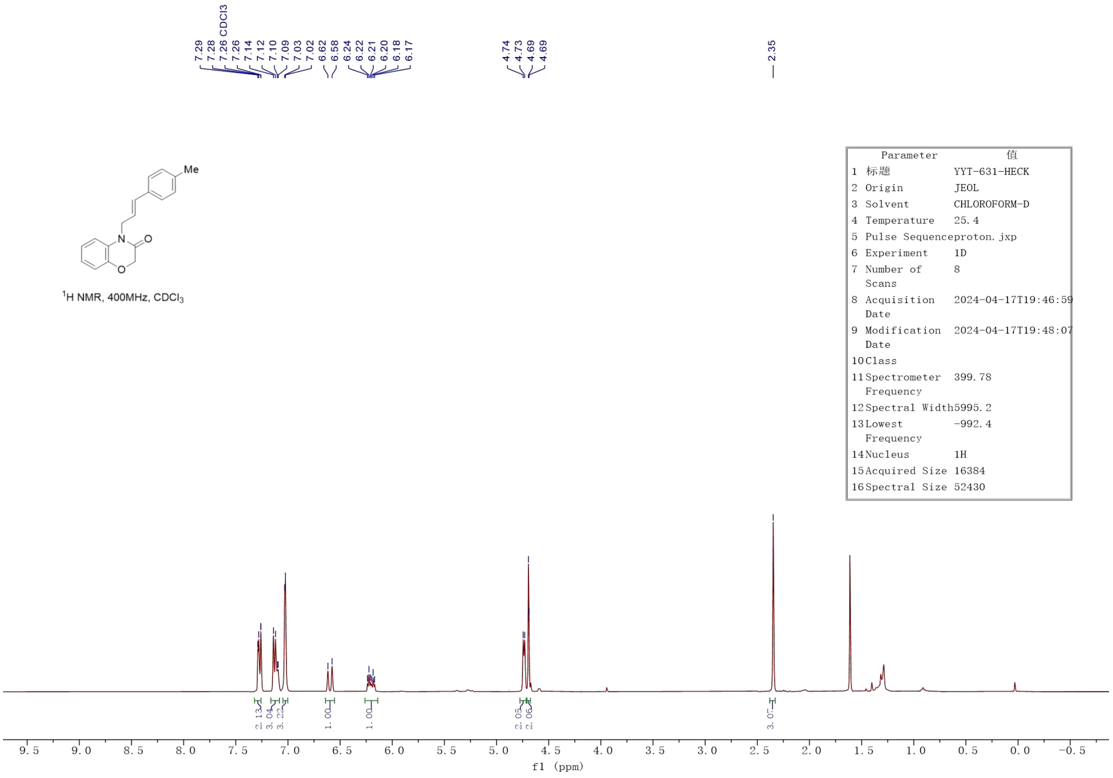

Parameter	値
1 标题	YYT-631-HECK
2 Origin	JEOL
3 Solvent	CHLOROFORM-D
4 Temperature	25.4
5 Pulse Sequence	proton.jxp
6 Experiment	1D
7 Number of Scans	8
8 Acquisition Date	2024-04-17T19:46:59
9 Modification Date	2024-04-17T19:48:07
10 Class	
11 Spectrometer Frequency	399.78
12 Spectral Width	5995.2
13 Lowest Frequency	-992.4
14 Nucleus	1H
15 Acquired Size	16384
16 Spectral Size	52430

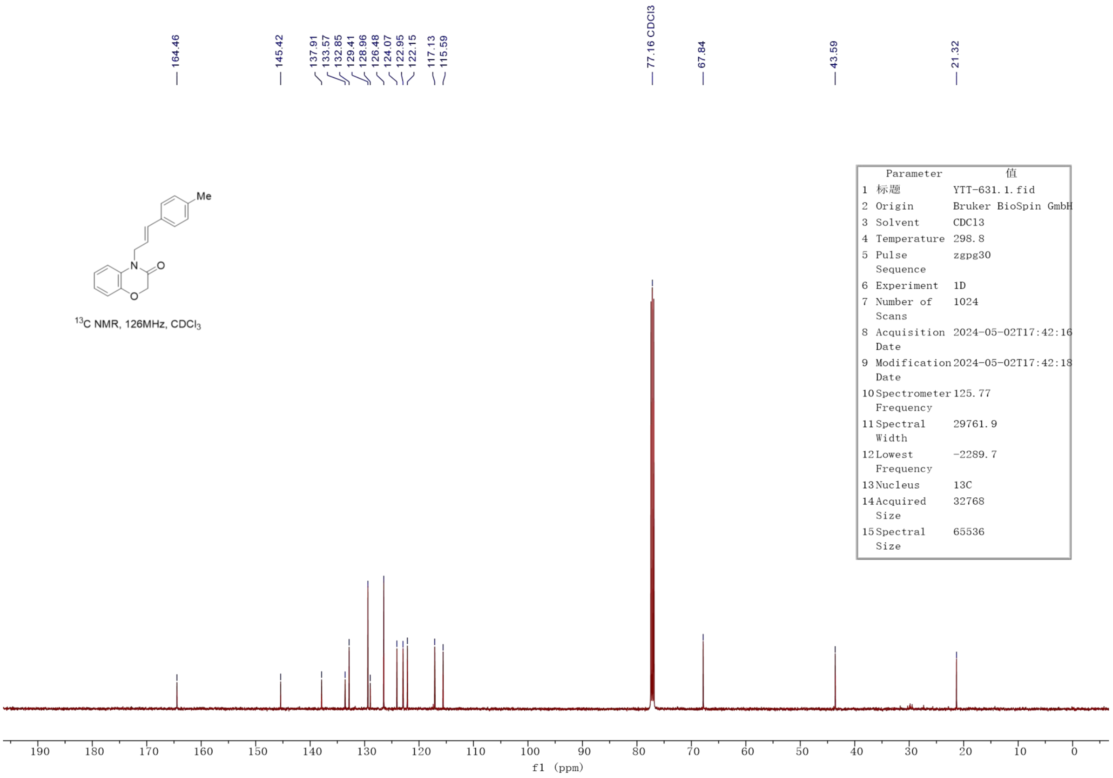

Parameter	値
1 标题	YTT-631.1.fid
2 Origin	Bruker BioSpin GmbH
3 Solvent	CDCl3
4 Temperature	298.8
5 Pulse Sequence	zgpg30
6 Experiment	1D
7 Number of Scans	1024
8 Acquisition Date	2024-05-02T17:42:16
9 Modification Date	2024-05-02T17:42:18
10 Spectrometer Frequency	125.77
11 Spectral Width	29761.9
12 Lowest Frequency	-2289.7
13 Nucleus	13C
14 Acquired Size	32768
15 Spectral Size	65536

4-(3,3-di-p-tolylallyl)-2*H*-benzo[b][1,4]oxazin-3(4*H*)-one (5)

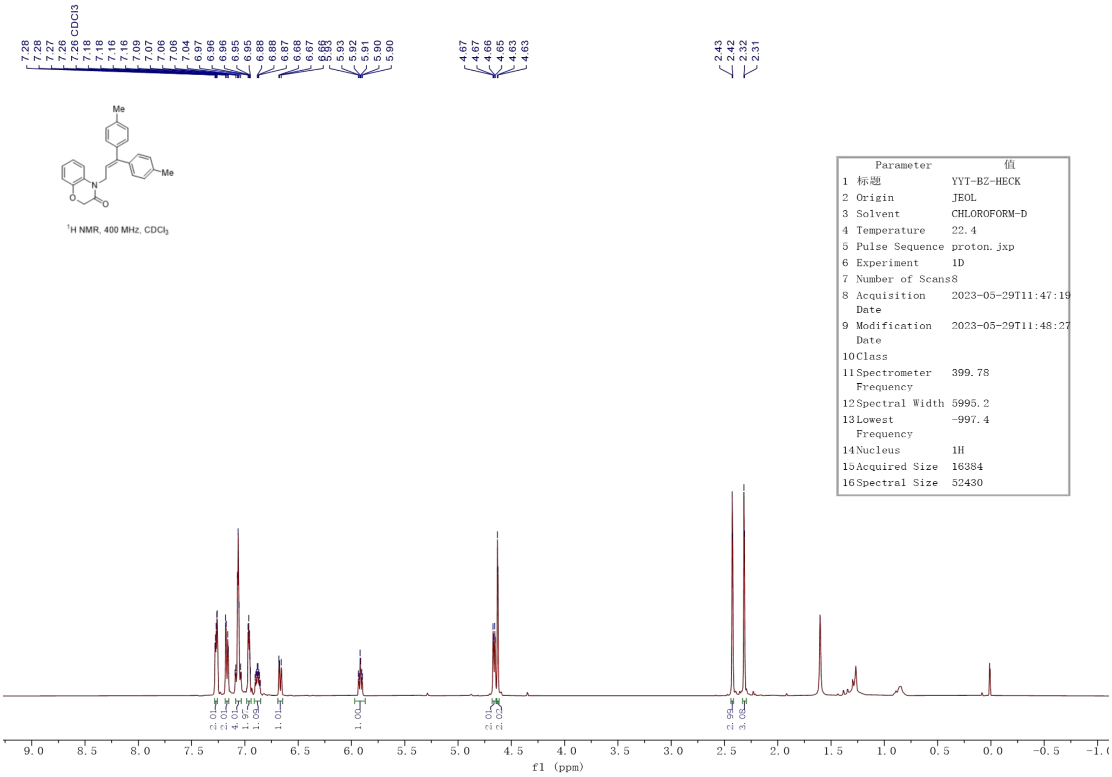

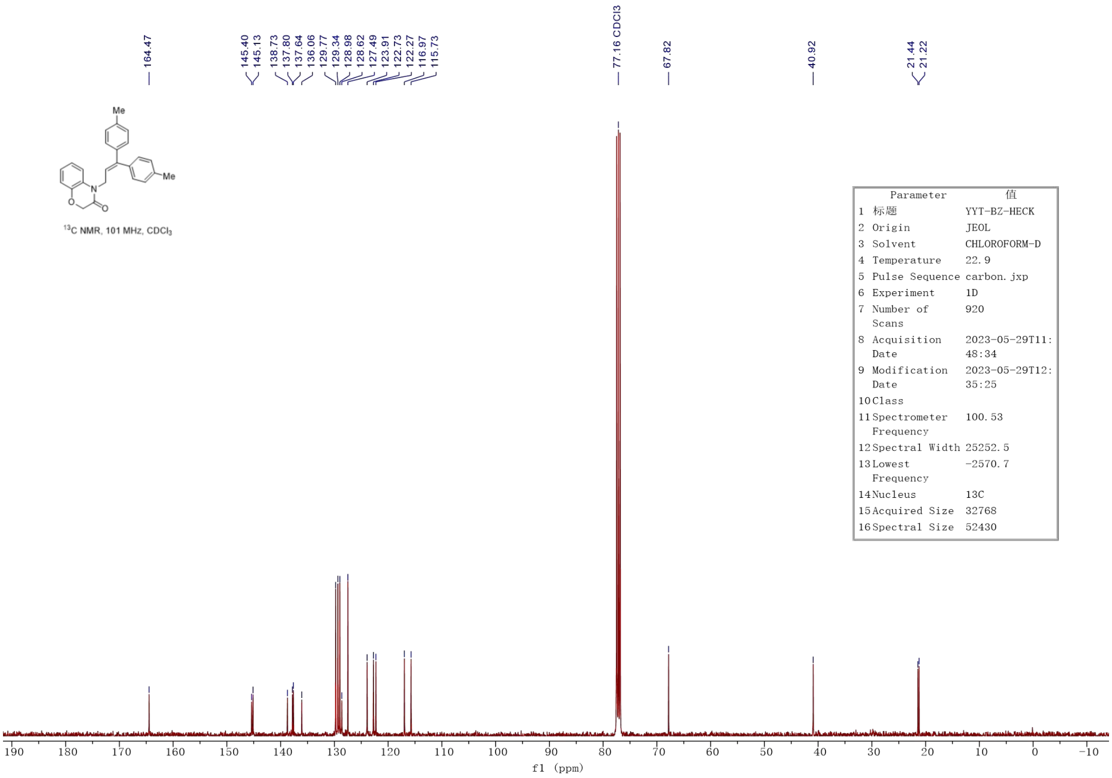

(R)-7-phenyl-6,7-dihydro-5H-[1,4]oxazino[2,3,4-i*J*]quinolin-3(2H)-one (6)

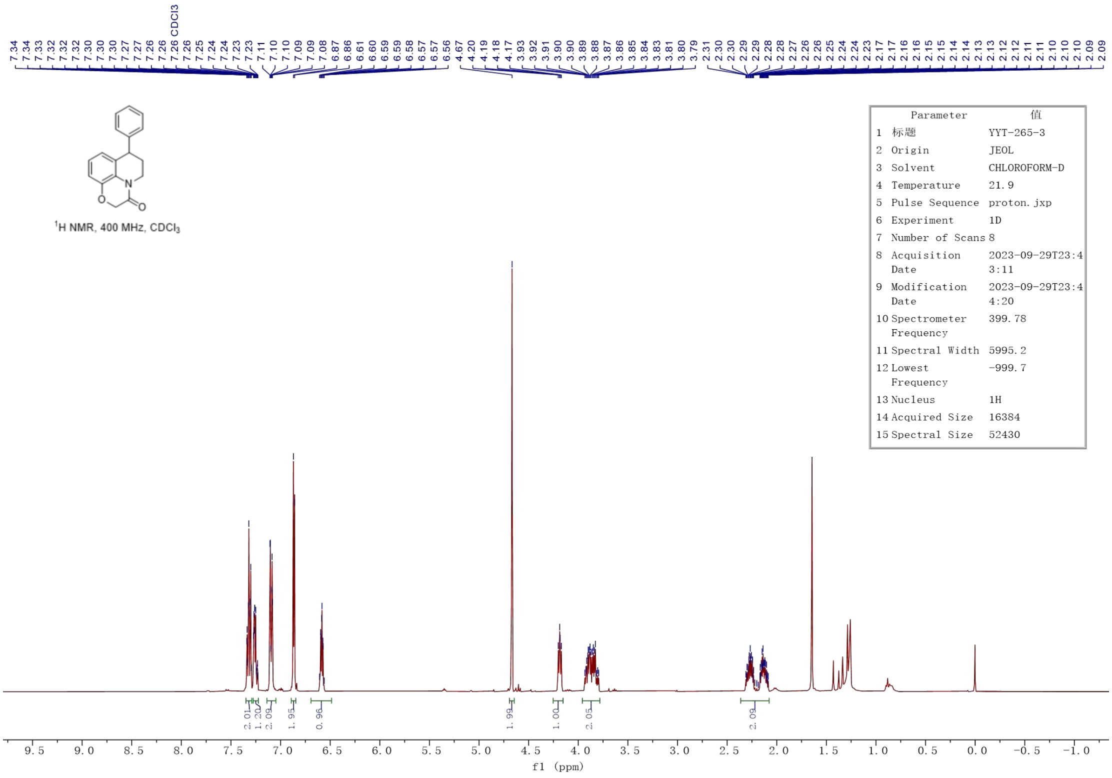

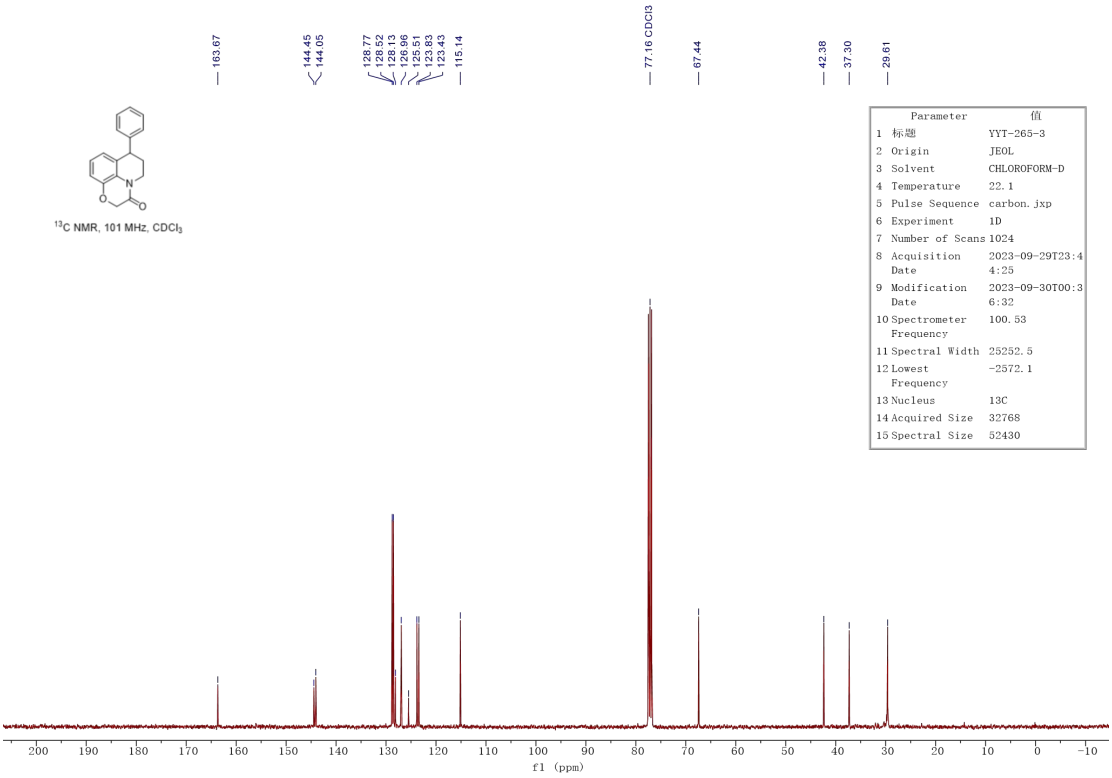

(R)-7-(naphthalen-2-yl)-6,7-dihydro-5H-[1,4]oxazino[2,3,4-i*J*]quinolin-3(2H)-one (7)

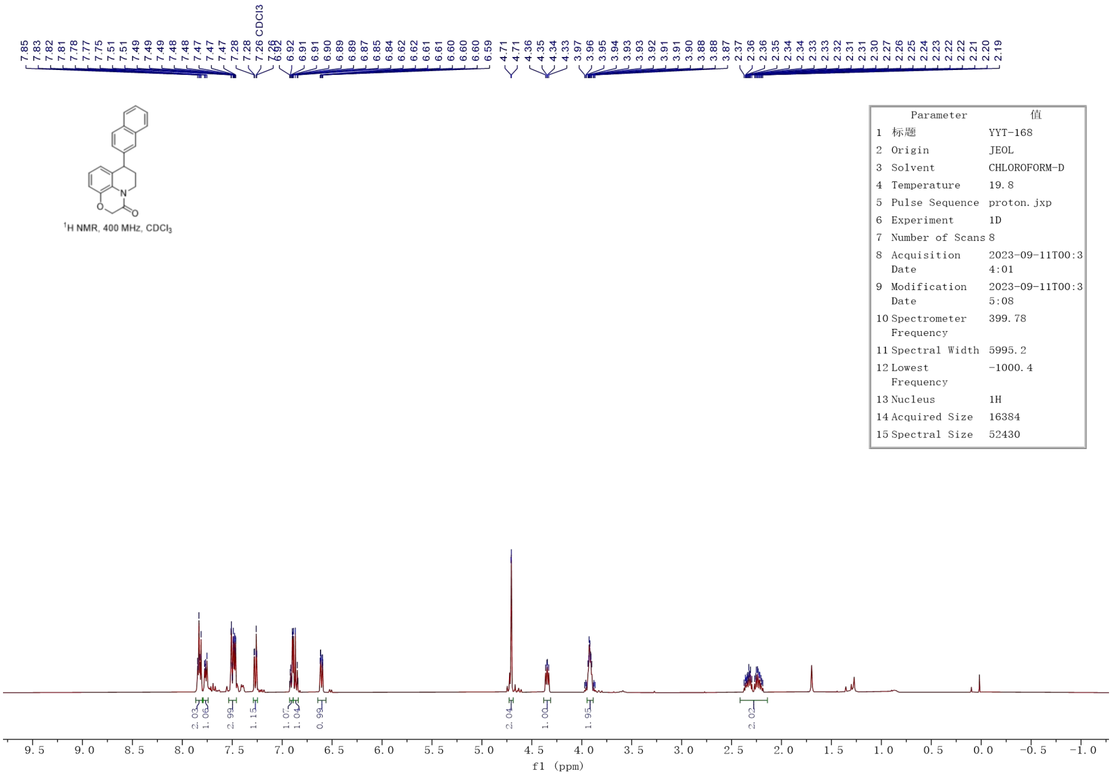

Parameter	值
1 标题	YYT-168
2 Origin	JEOL
3 Solvent	CHLOROFORM-D
4 Temperature	19.8
5 Pulse Sequence	proton.jxp
6 Experiment	1D
7 Number of Scans	8
8 Acquisition Date	2023-09-11T00:34:01
9 Modification Date	2023-09-11T00:35:08
10 Spectrometer Frequency	399.78
11 Spectral Width	5995.2
12 Lowest Frequency	-1000.4
13 Nucleus	1H
14 Acquired Size	16384
15 Spectral Size	52430

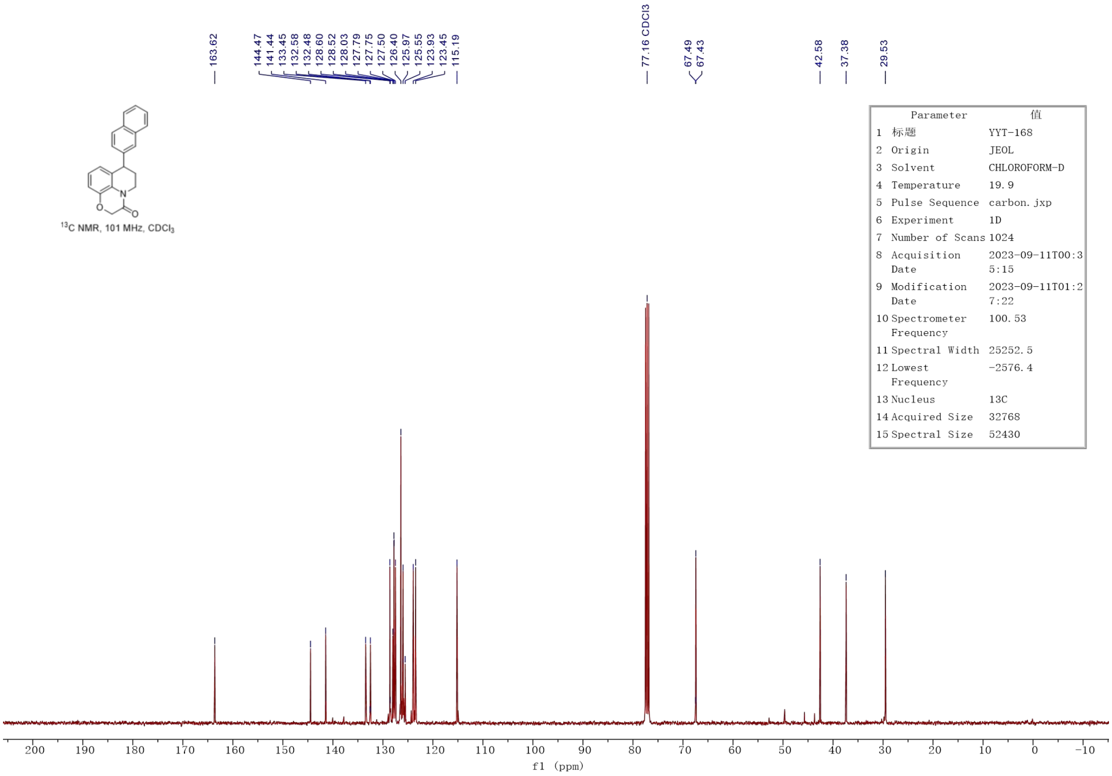

Parameter	值
1 标题	YYT-168
2 Origin	JEOL
3 Solvent	CHLOROFORM-D
4 Temperature	19.9
5 Pulse Sequence	carbon.jxp
6 Experiment	1D
7 Number of Scans	1024
8 Acquisition Date	2023-09-11T00:35:15
9 Modification Date	2023-09-11T01:27:22
10 Spectrometer Frequency	100.53
11 Spectral Width	25252.5
12 Lowest Frequency	-2576.4
13 Nucleus	13C
14 Acquired Size	32768
15 Spectral Size	52430

(R)-7-(naphthalen-1-yl)-6,7-dihydro-5H-[1,4]oxazino[2,3,4-*ij*]quinolin-3(2H)-one (8)

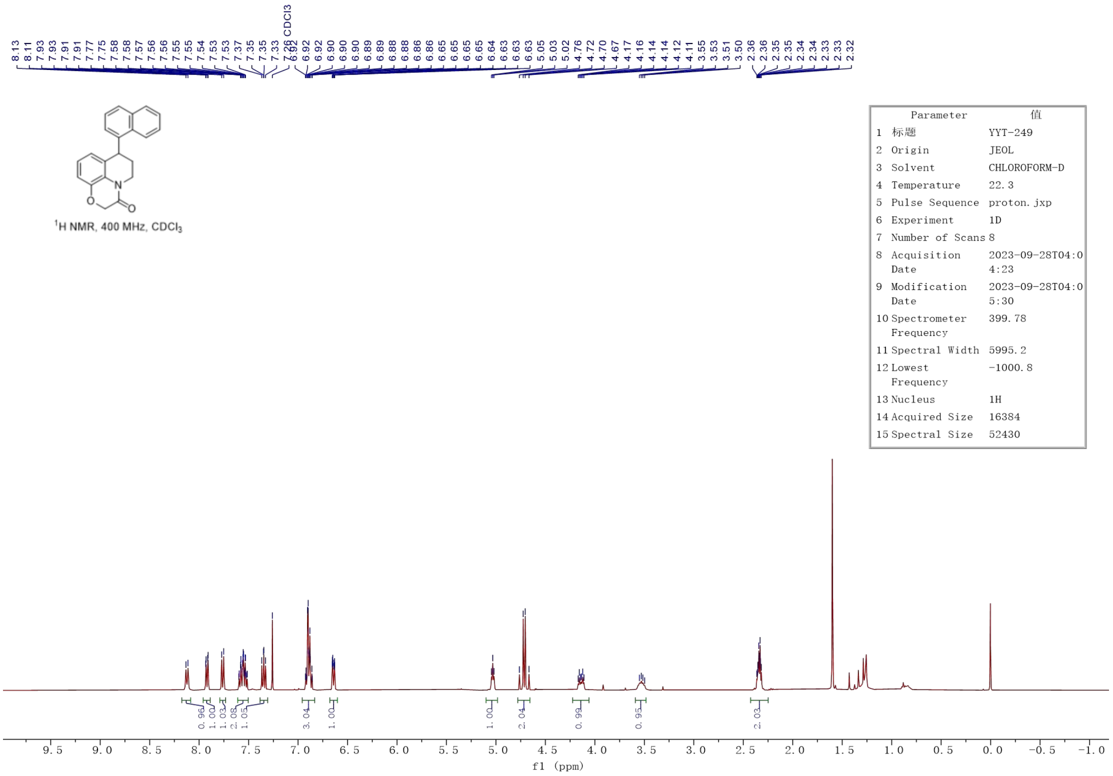

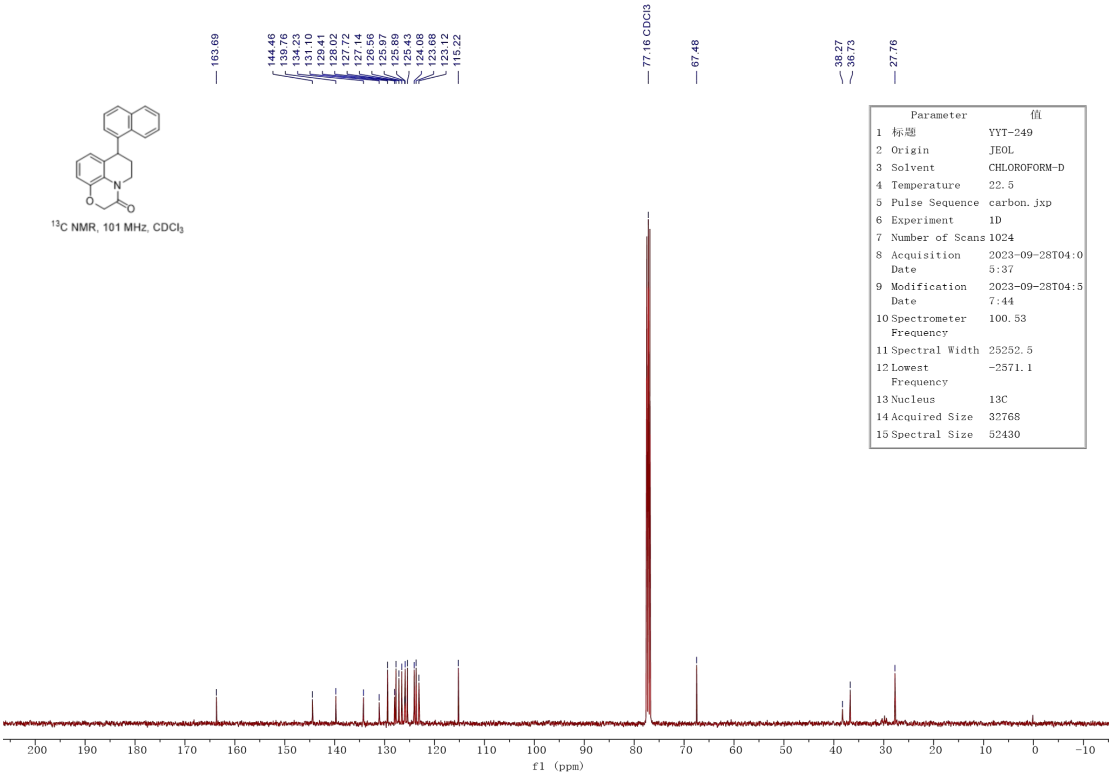

(R)-7-(4-methoxyphenyl)-6,7-dihydro-5H-[1,4]oxazino[2,3,4-i*J*]quinolin-3(2H)-one (9)

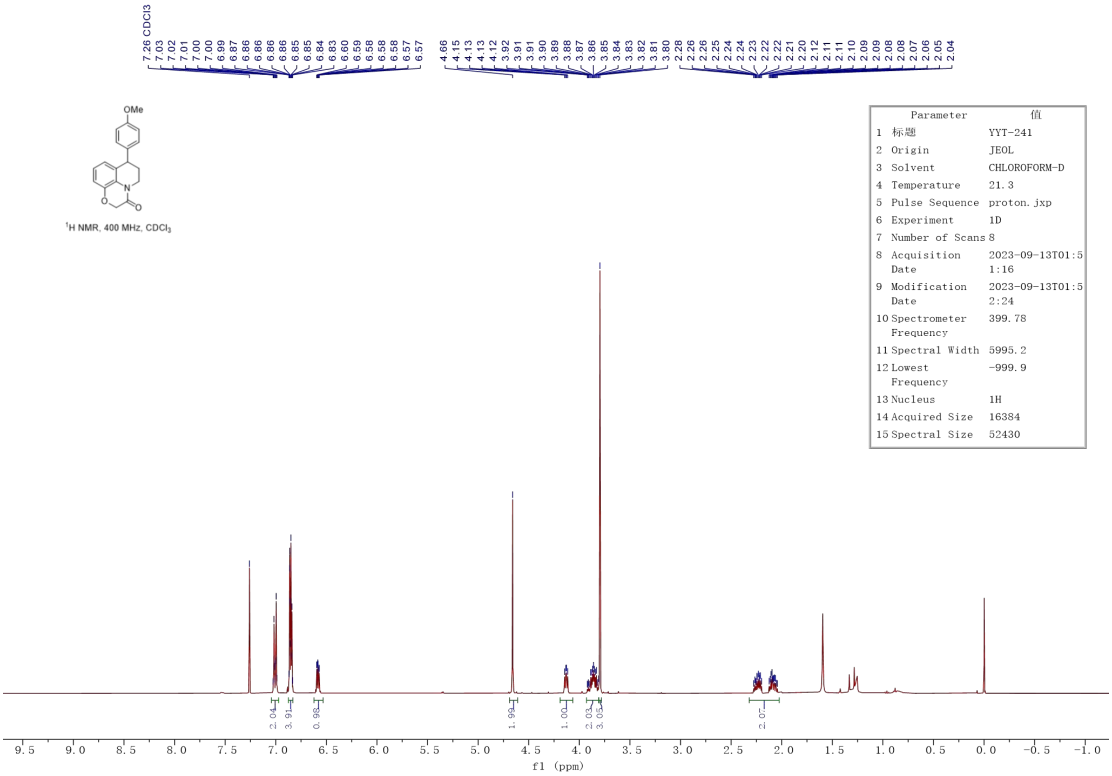

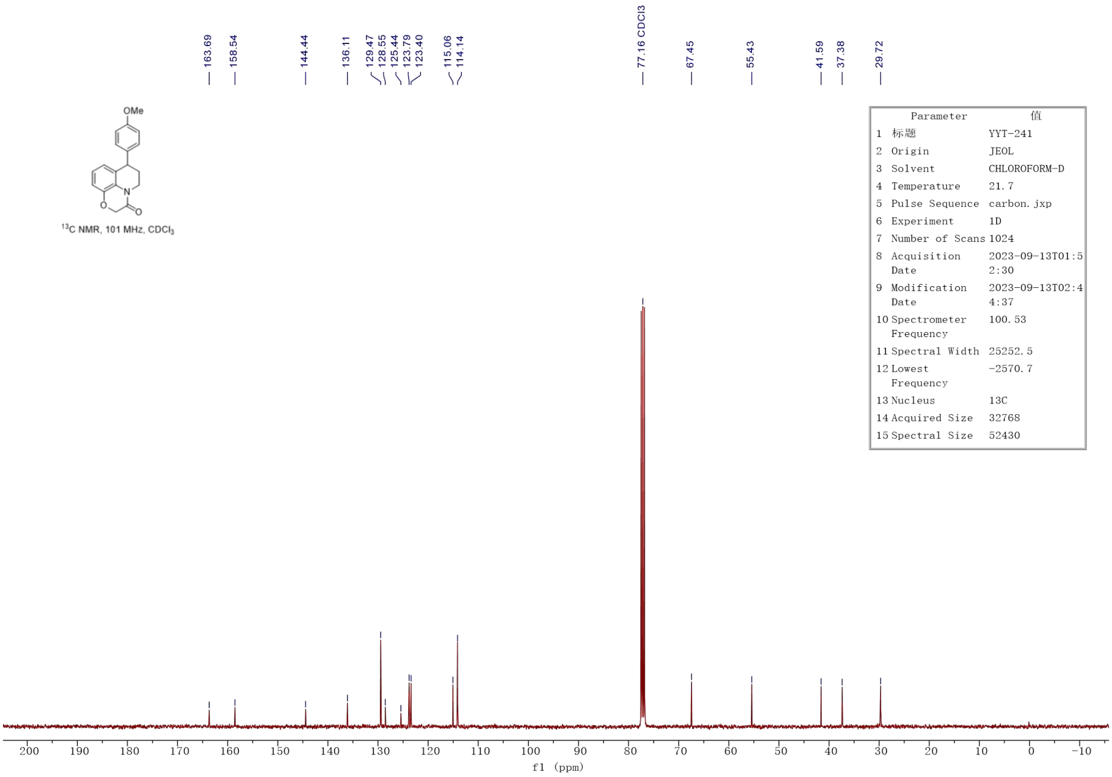

(R)-7-(4-(tert-butyl)phenyl)-6,7-dihydro-5H-[1,4]oxazino[2,3,4-i*J*]quinolin-3(2H)-one (10)

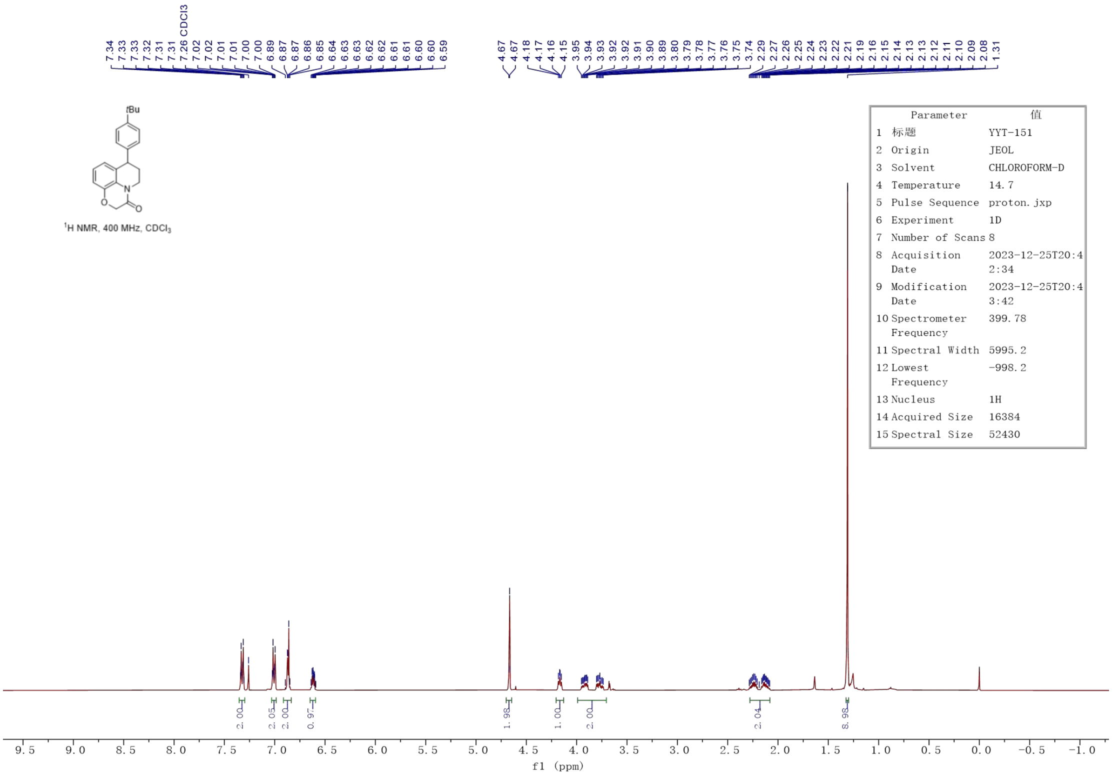

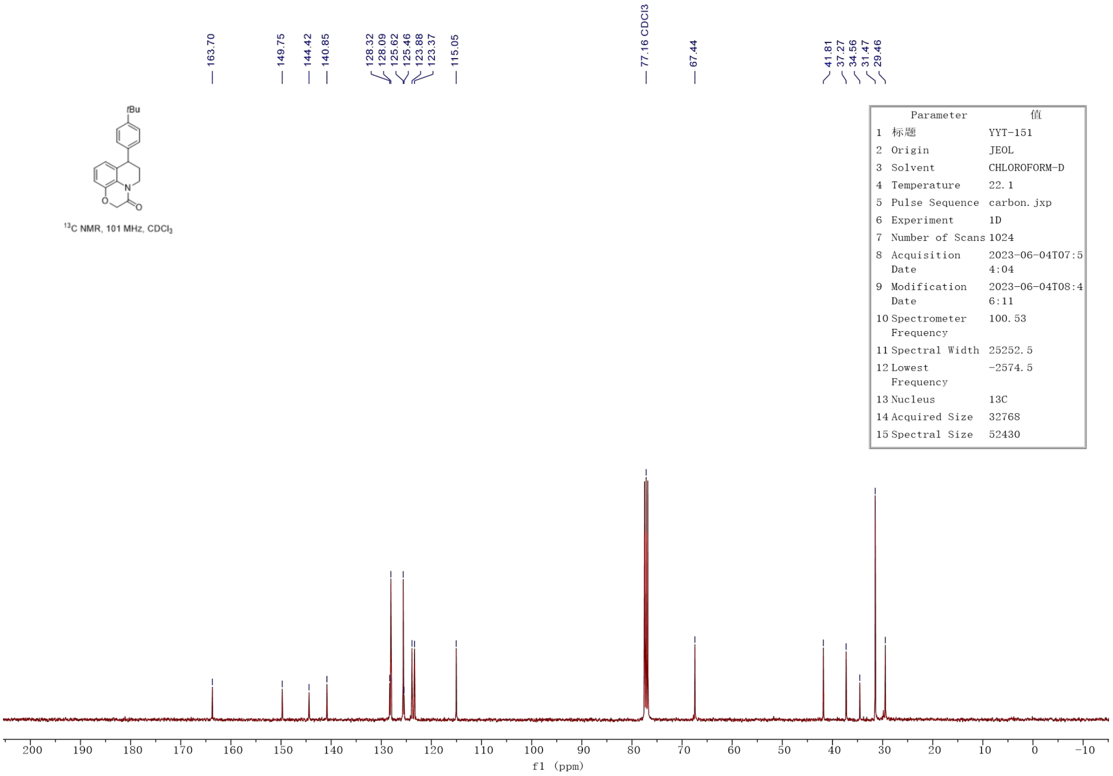

(R)-7-(4-(benzyloxy)phenyl)-6,7-dihydro-5H-[1,4]oxazino[2,3,4-i*J*]quinolin-3(2H)-one (11)

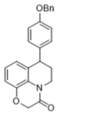

^{1}H NMR, 400 MHz, $CDCl_3$

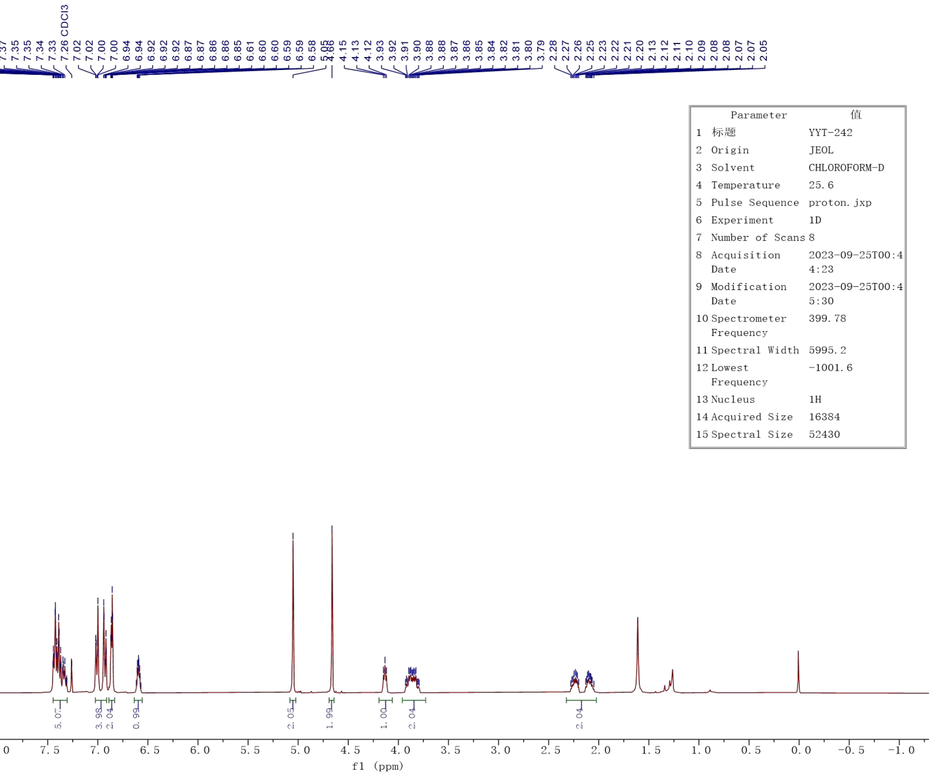

	Parameter	值
1	标题	YYT-242
2	Origin	JEOL
3	Solvent	CHLOROFORM-D
4	Temperature	25.6
5	Pulse Sequence	proton.jxp
6	Experiment	1D
7	Number of Scans	8
8	Acquisition Date	2023-09-25T00:44:23
9	Modification Date	2023-09-25T00:45:30
10	Spectrometer Frequency	399.78
11	Spectral Width	5995.2
12	Lowest Frequency	-1001.6
13	Nucleus	1H
14	Acquired Size	16384
15	Spectral Size	52430

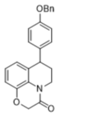

^{13}C NMR, 101 MHz, $CDCl_3$

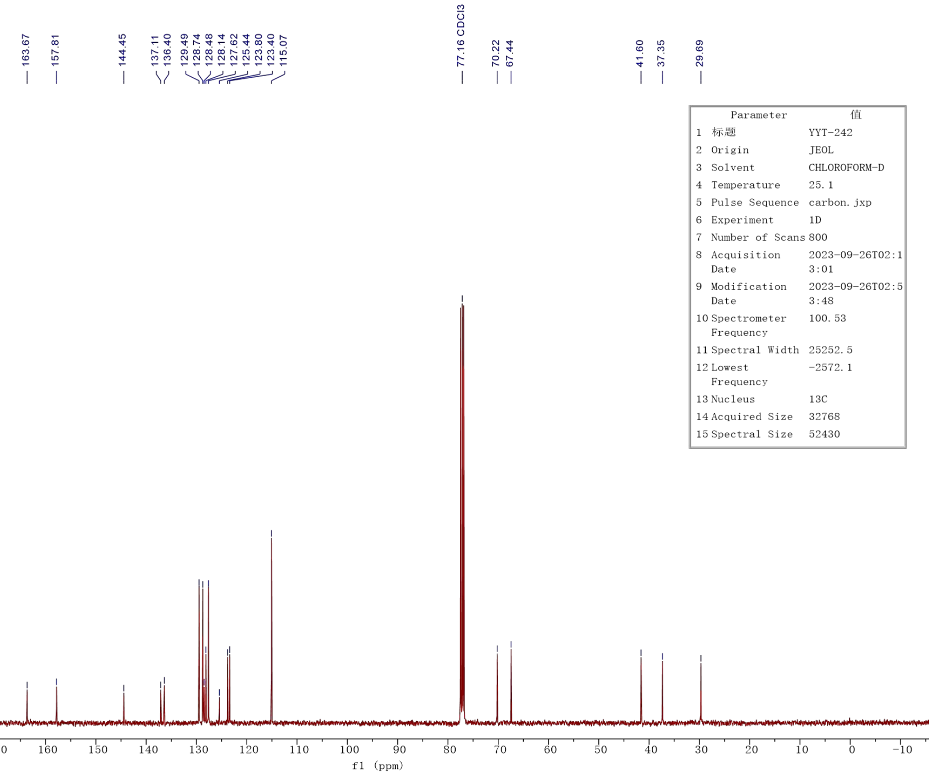

	Parameter	值
1	标题	YYT-242
2	Origin	JEOL
3	Solvent	CHLOROFORM-D
4	Temperature	25.1
5	Pulse Sequence	carbon.jxp
6	Experiment	1D
7	Number of Scans	800
8	Acquisition Date	2023-09-26T02:13:01
9	Modification Date	2023-09-26T02:53:48
10	Spectrometer Frequency	100.53
11	Spectral Width	25252.5
12	Lowest Frequency	-2572.1
13	Nucleus	13C
14	Acquired Size	32768
15	Spectral Size	52430

(R)-7-(4-isopropylphenyl)-6,7-dihydro-5H-[1,4]oxazino[2,3,4-i*J*]quinolin-3(2H)-one (12)

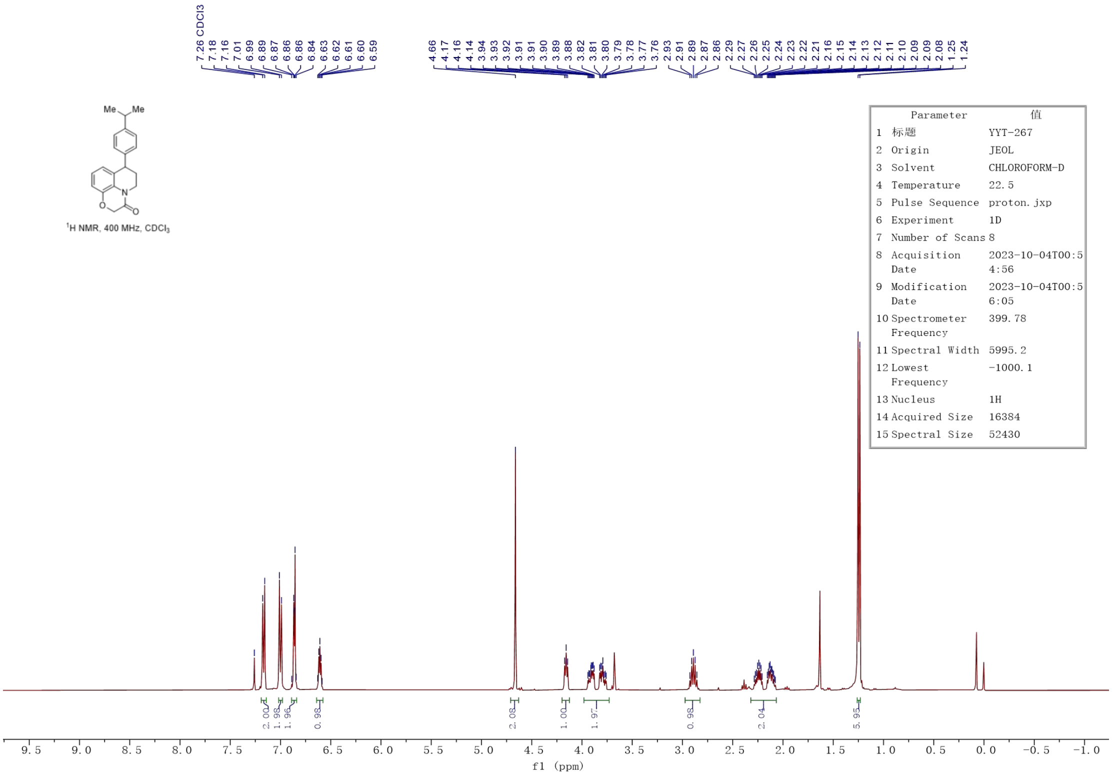

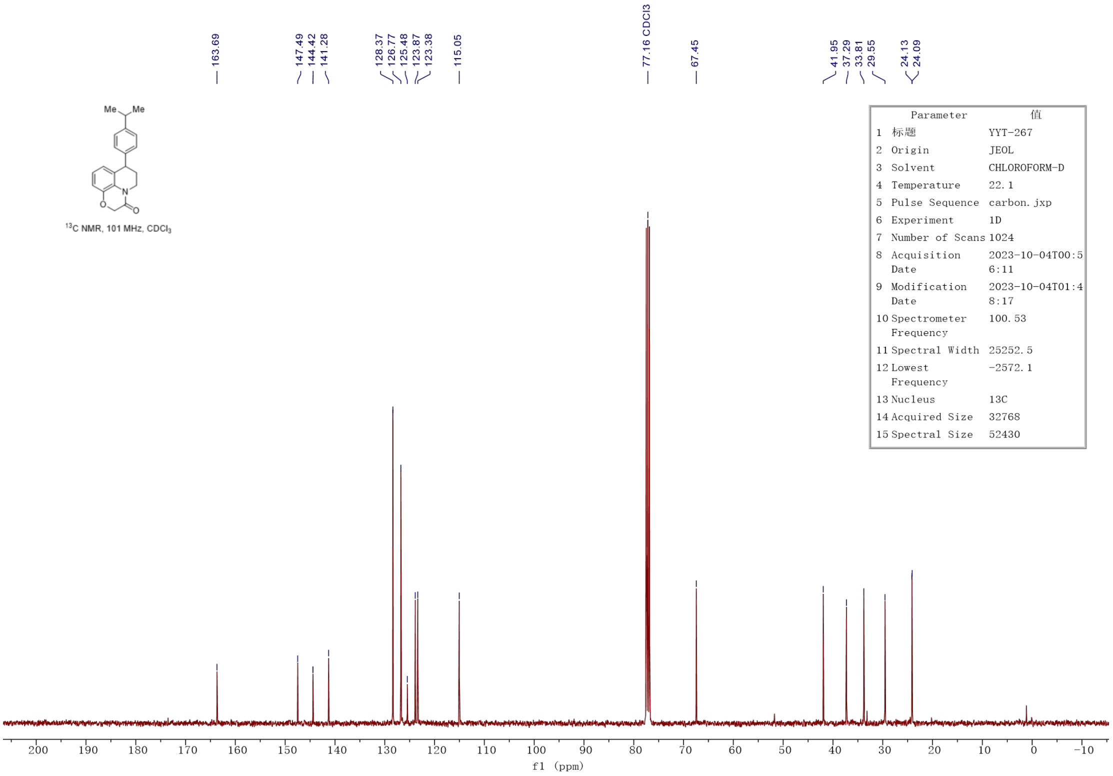

(R)-7-(4-cyclohexylphenyl)-6,7-dihydro-5H-[1,4]oxazino[2,3,4-i*J*]quinolin-3(2H)-one (13)

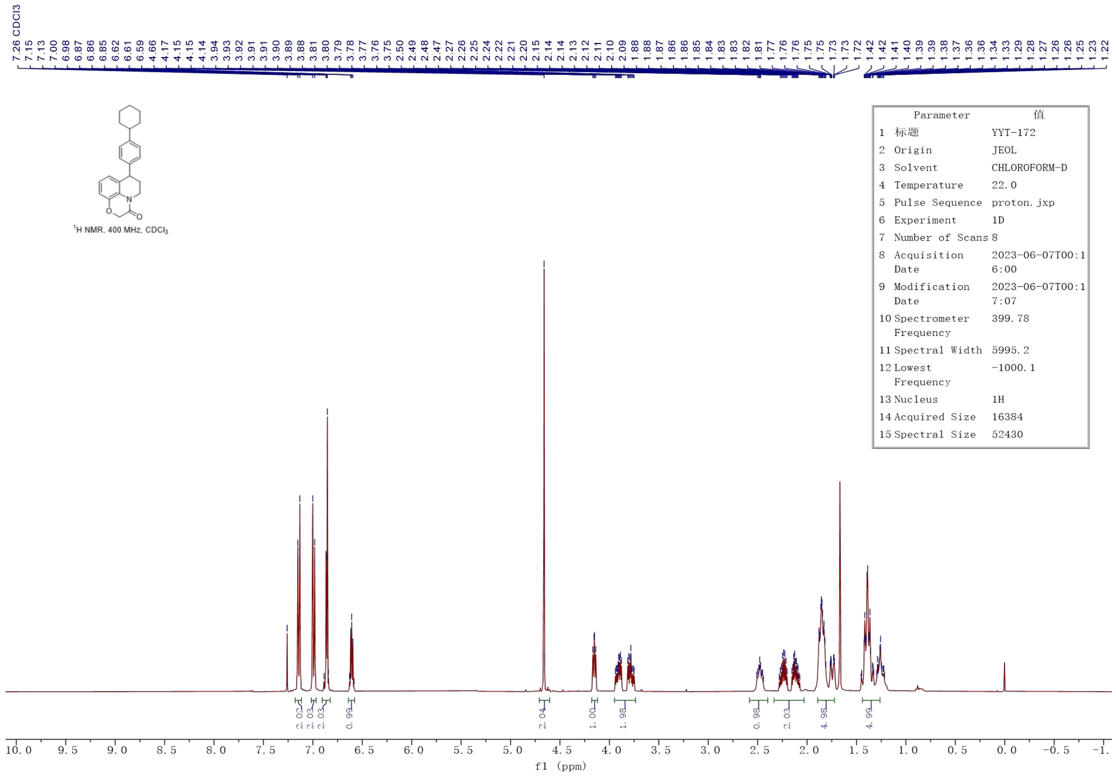

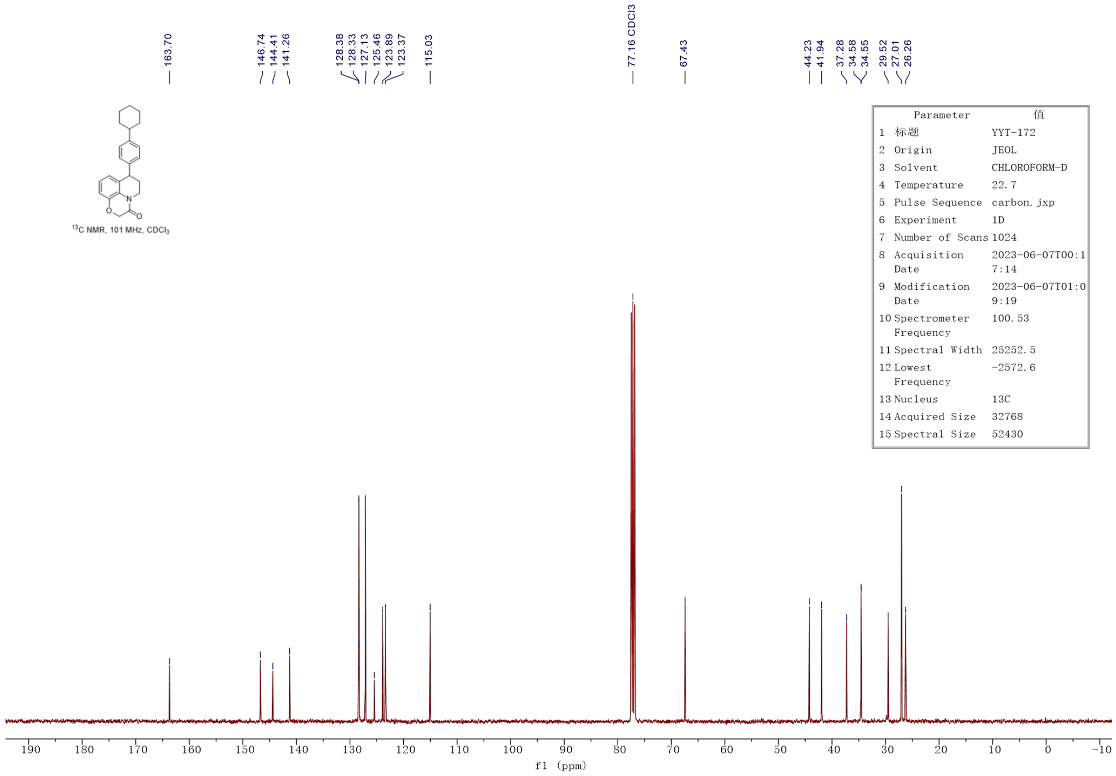

Methyl(R)-3-(4-(3-oxo-2,3,6,7-tetrahydro-5H-[1,4]oxazino[2,3,4-i*J*]quinolin-7-yl) phenyl)propanoate (14)

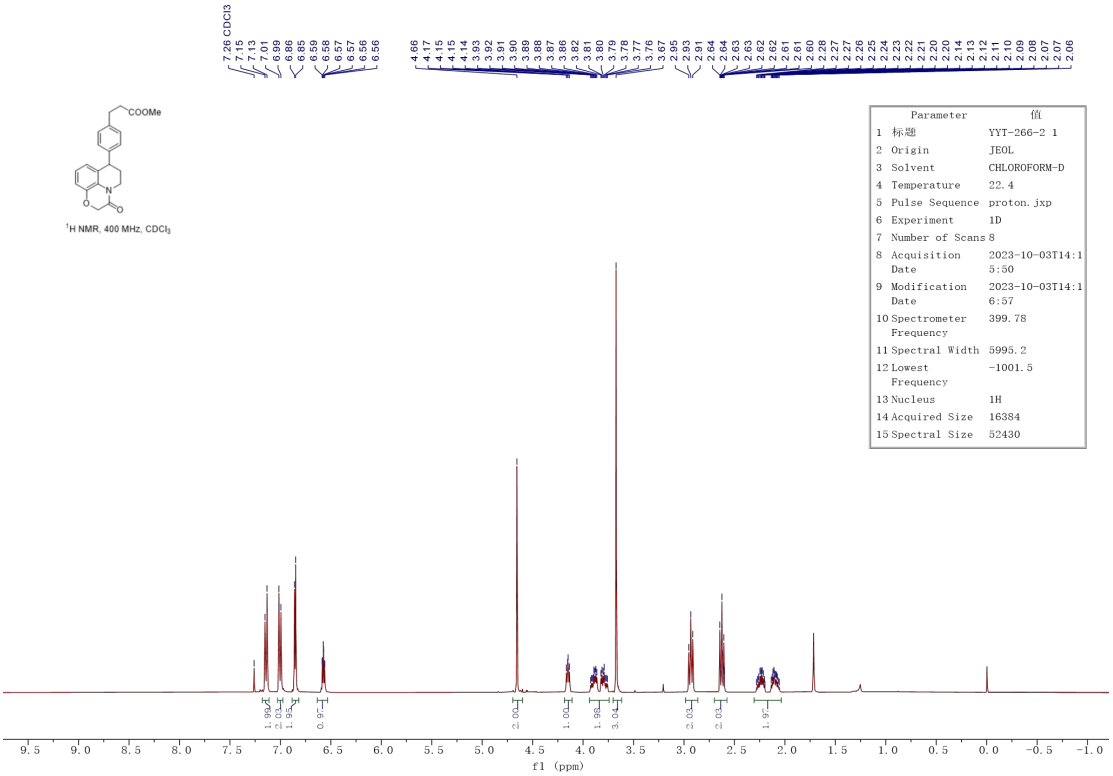

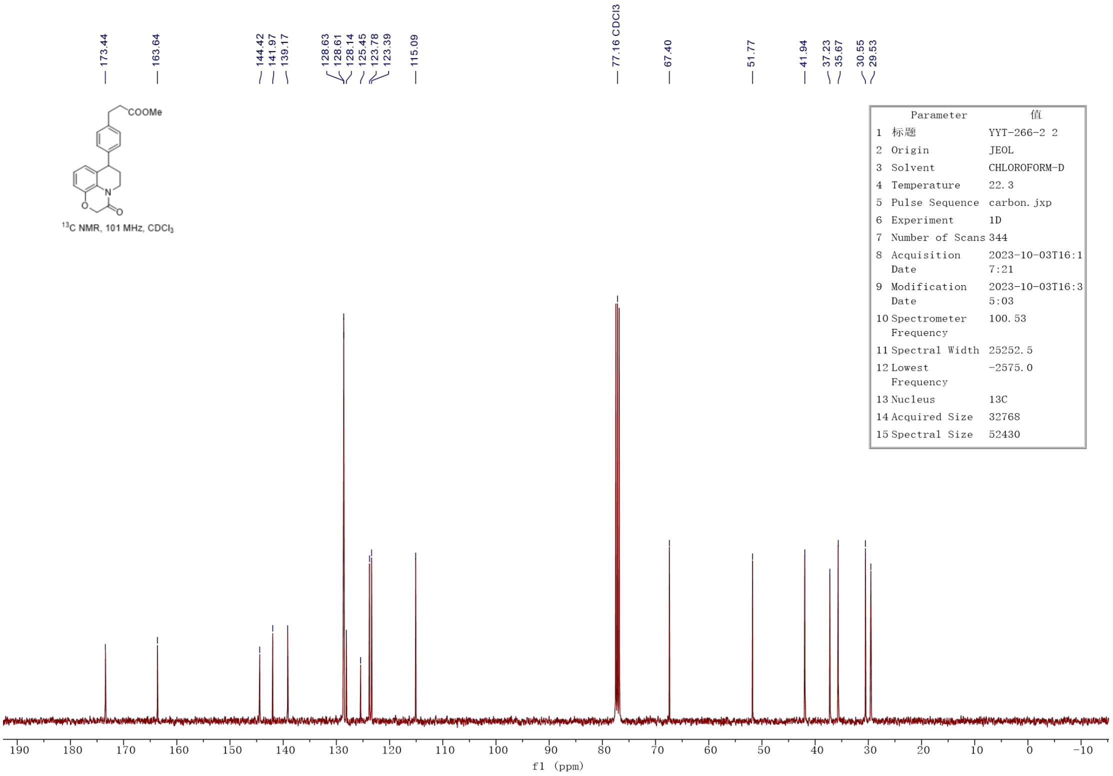

(R)-7-(4-(difluoromethoxy)phenyl)-6,7-dihydro-5H-[1,4]oxazino[2,3,4-i*J*]quinolin-3(2H)-one (15)

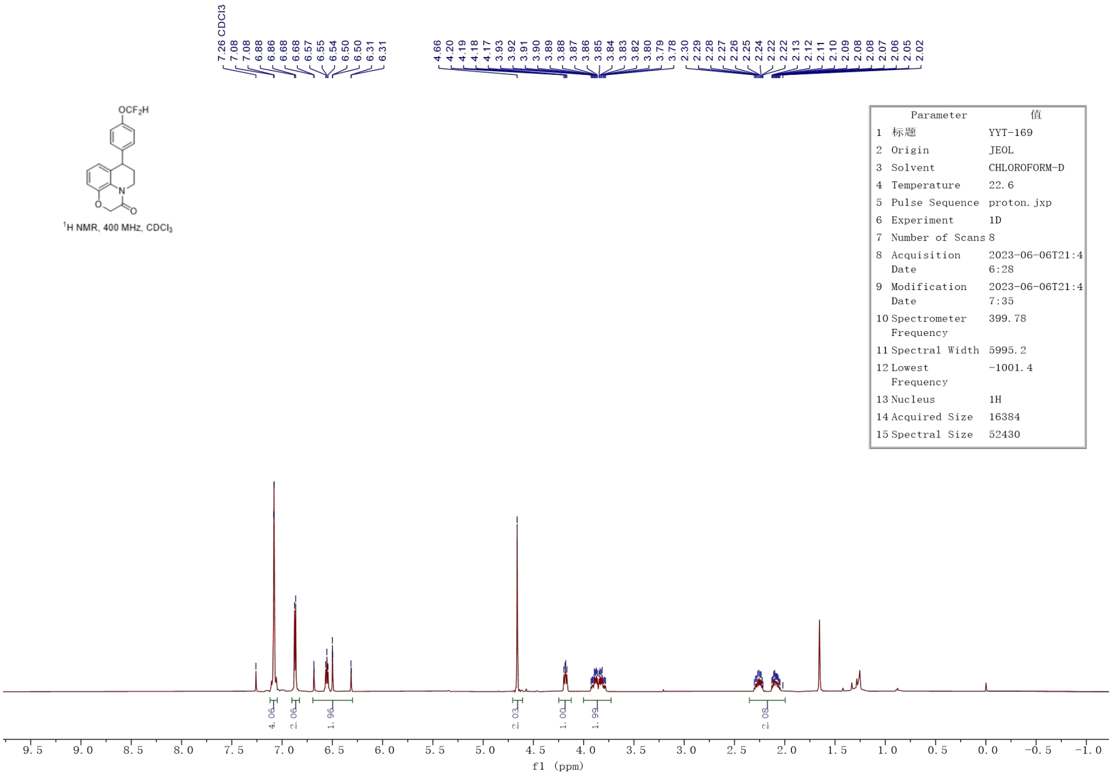

	Parameter	値
1	标题	YYT-169
2	Origin	JEOL
3	Solvent	CHLOROFORM-D
4	Temperature	22.6
5	Pulse Sequence	proton.jxp
6	Experiment	1D
7	Number of Scans	8
8	Acquisition Date	2023-06-06T21:46:28
9	Modification Date	2023-06-06T21:47:35
10	Spectrometer Frequency	399.78
11	Spectral Width	5995.2
12	Lowest Frequency	-1001.4
13	Nucleus	1H
14	Acquired Size	16384
15	Spectral Size	52430

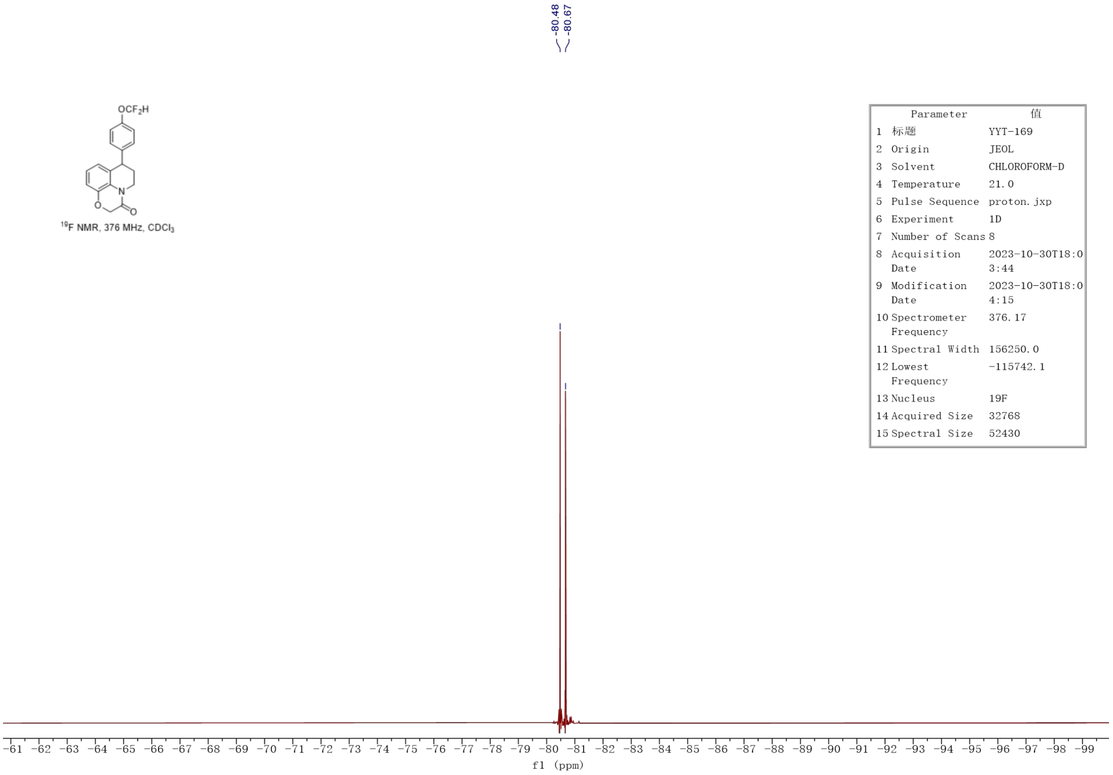

	Parameter	値
1	标题	YYT-169
2	Origin	JEOL
3	Solvent	CHLOROFORM-D
4	Temperature	21.0
5	Pulse Sequence	proton.jxp
6	Experiment	1D
7	Number of Scans	8
8	Acquisition Date	2023-10-30T18:03:44
9	Modification Date	2023-10-30T18:04:15
10	Spectrometer Frequency	376.17
11	Spectral Width	156250.0
12	Lowest Frequency	-115742.1
13	Nucleus	19F
14	Acquired Size	32768
15	Spectral Size	52430

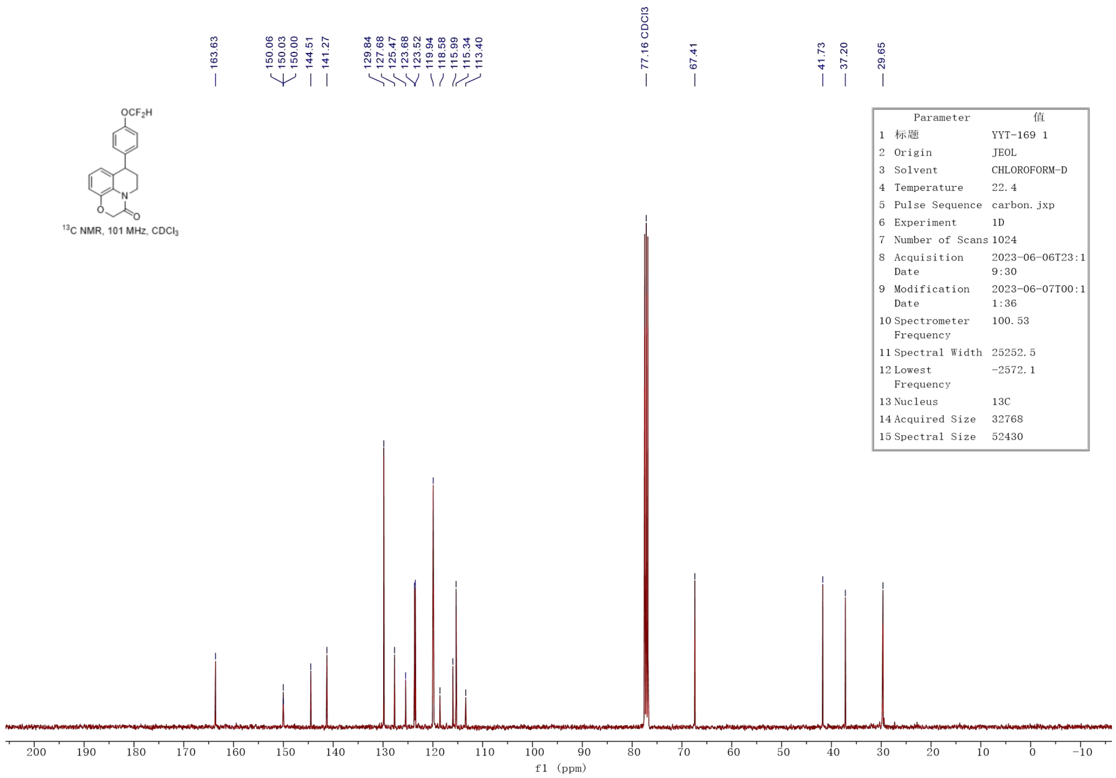

163.63
150.06
150.03
150.00
144.51
141.27
129.84
127.68
125.47
123.68
123.52
119.94
118.58
115.99
115.34
113.40
77.16 CDCl3
67.41
41.73
37.20
29.65
OCF2H
N
O
O
13C NMR, 101 MHz, CDCl3
Parameter 值
1 标题 YYT-169 1
2 Origin JEOL
3 Solvent CHLOROFORM-D
4 Temperature 22.4
5 Pulse Sequence carbon.jxp
6 Experiment 1D
7 Number of Scans 1024
8 Acquisition Date 2023-06-06T23:19:30
9 Modification Date 2023-06-07T00:11:36
10 Spectrometer Frequency 100.53
11 Spectral Width 25252.5
12 Lowest Frequency -2572.1
13 Nucleus 13C
14 Acquired Size 32768
15 Spectral Size 52430
200 190 180 170 160 150 140 130 120 110 100 90 80 70 60 50 40 30 20 10 0 -10
f1 (ppm)

(R)-7-(4-(trifluoromethoxy)phenyl)-6,7-dihydro-5H-[1,4]oxazino[2,3,4-iJ]quinoli n-3(2H)-one (16)

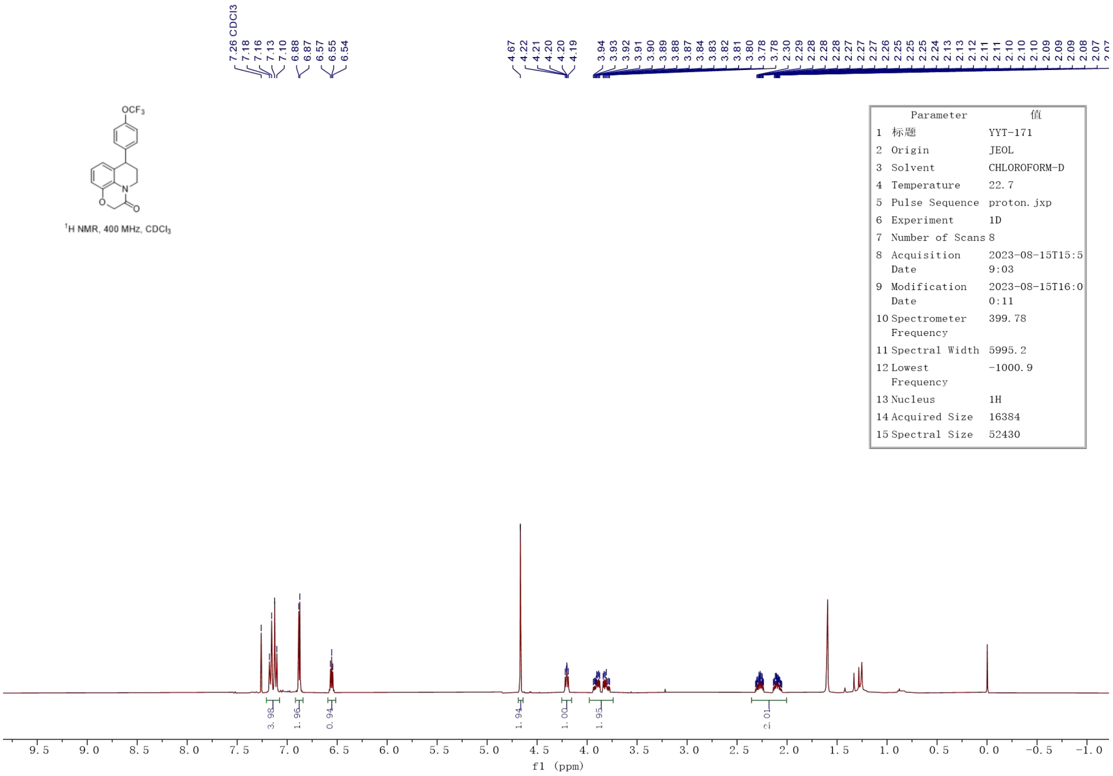

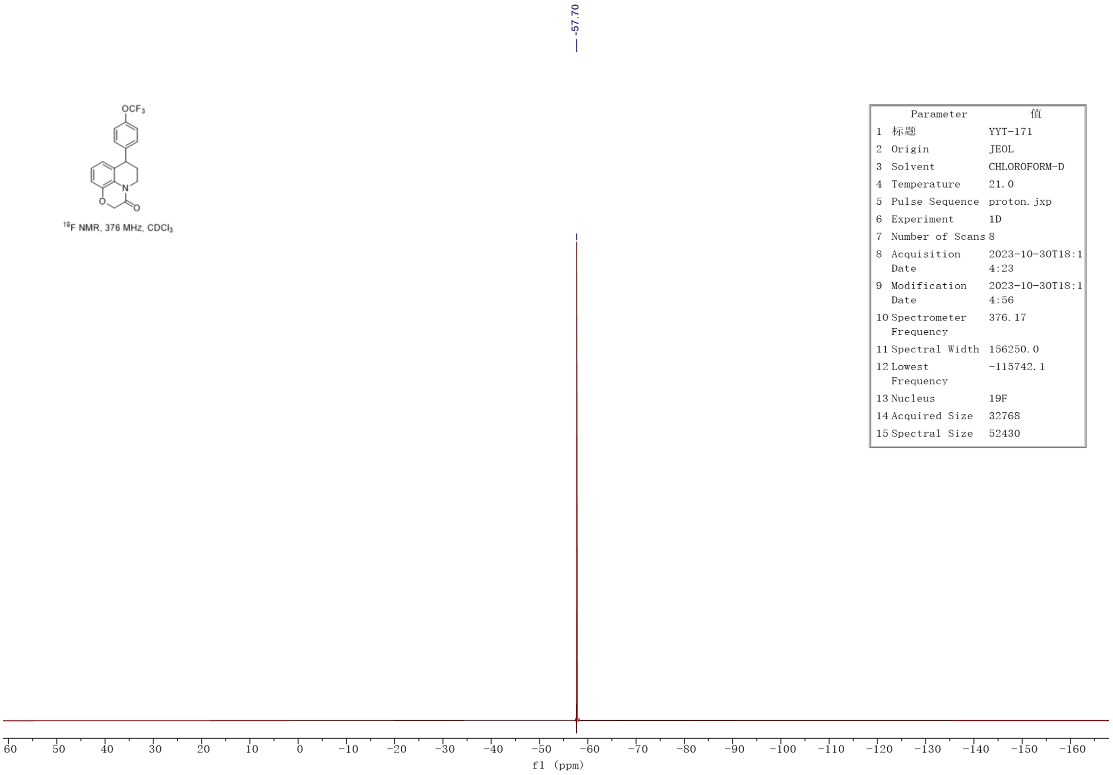

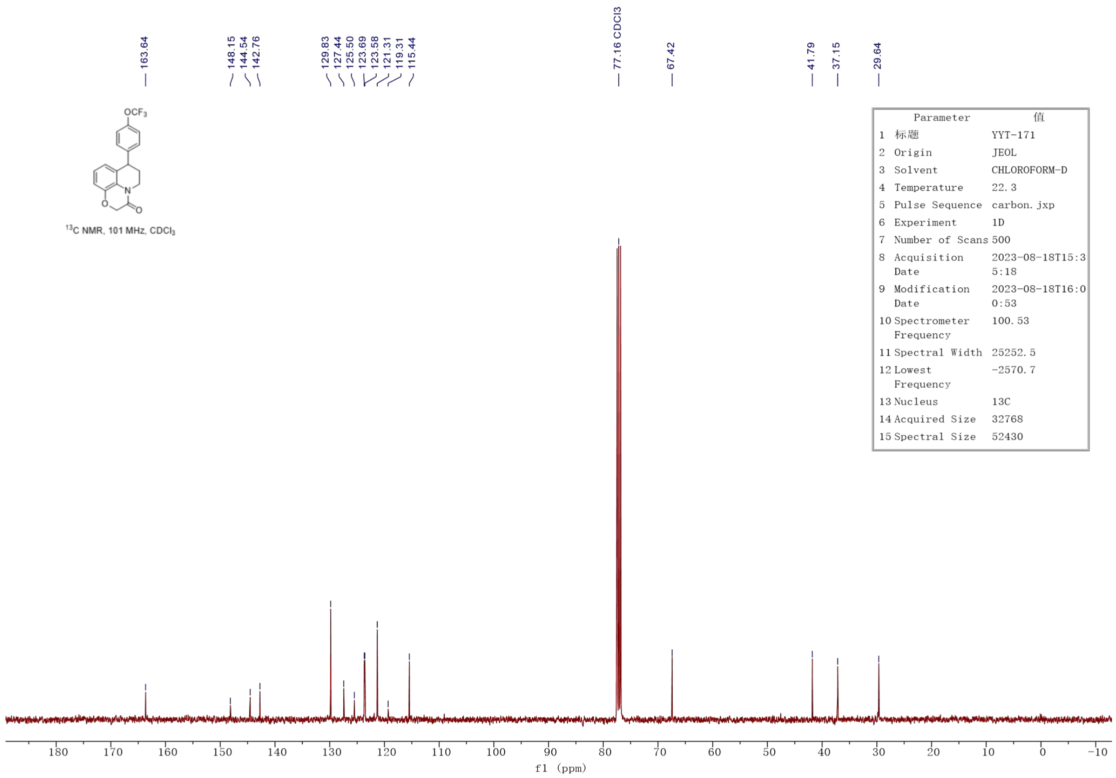

	Parameter	值
1	标题	YYT-171
2	Origin	JEOL
3	Solvent	CHLOROFORM-D
4	Temperature	22.3
5	Pulse Sequence	carbon.jxp
6	Experiment	1D
7	Number of Scans	500
8	Acquisition Date	2023-08-18T15:35:18
9	Modification Date	2023-08-18T16:00:53
10	Spectrometer Frequency	100.53
11	Spectral Width	25252.5
12	Lowest Frequency	-2570.7
13	Nucleus	13C
14	Acquired Size	32768
15	Spectral Size	52430

(R)-7-(4-(trifluoromethyl)phenyl)-6,7-dihydro-5H-[1,4]oxazino[2,3,4-i*J*]quinolin-3(2H)-one (17)

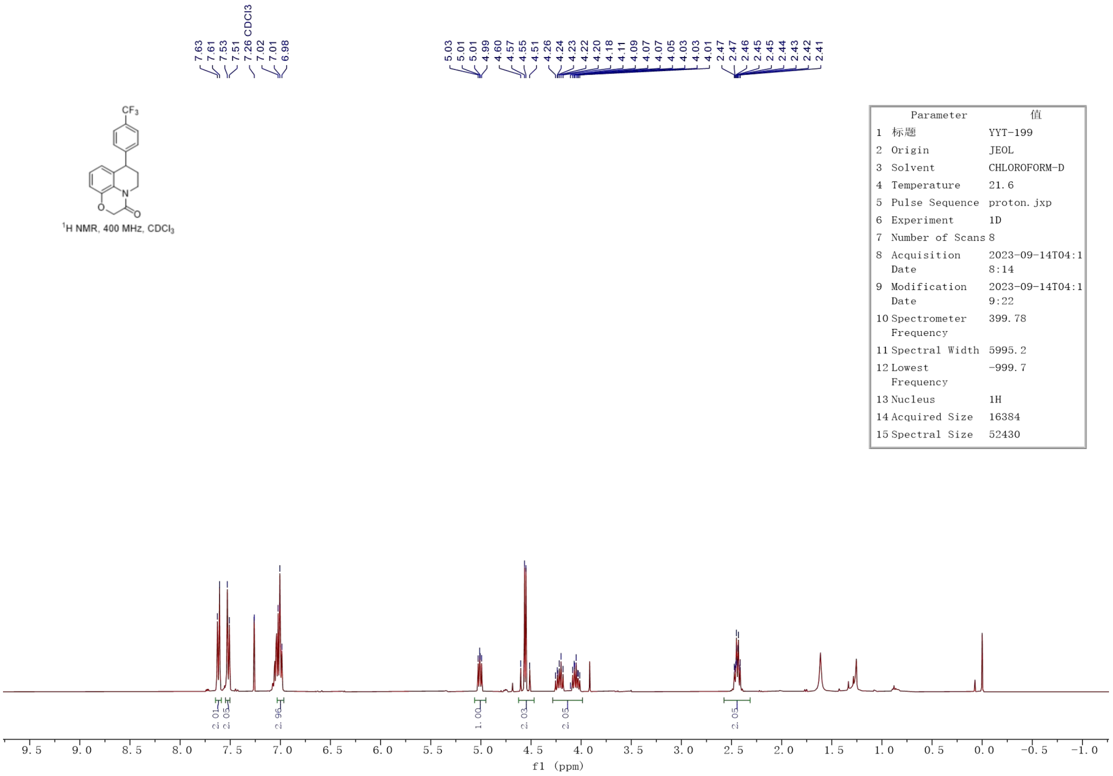

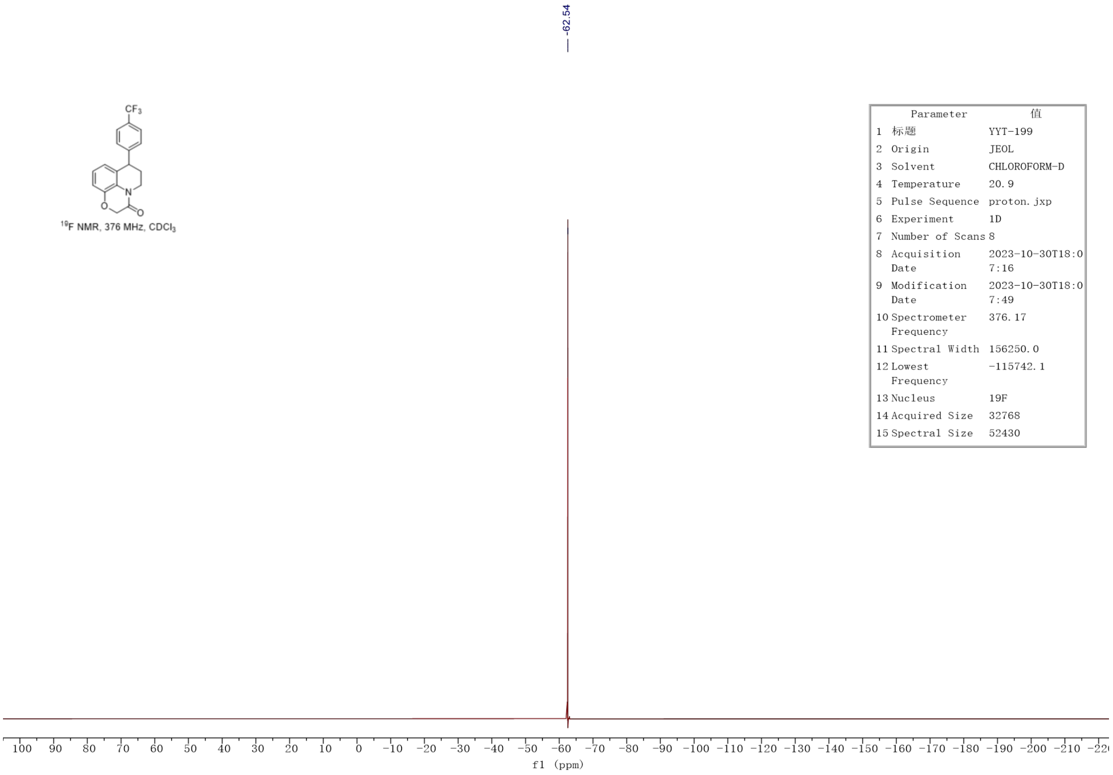

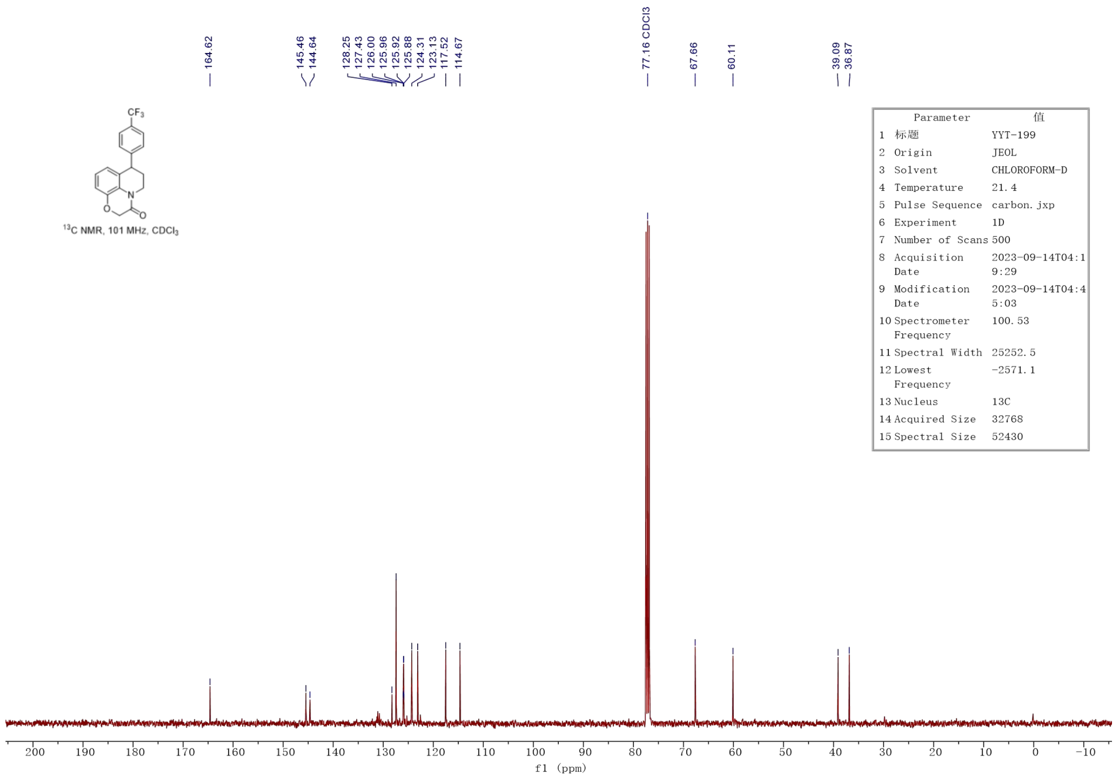

164.62
145.46
144.64
128.25
127.43
126.00
125.96
125.92
125.88
124.31
123.13
117.52
114.67
77.16 CDCl3
67.66
60.11
39.09
36.87
CF3
O
N
O
13C NMR, 101 MHz, CDCl3
Parameter 值
1 标题 YYT-199
2 Origin JEOL
3 Solvent CHLOROFORM-D
4 Temperature 21.4
5 Pulse Sequence carbon.jxp
6 Experiment 1D
7 Number of Scans 500
8 Acquisition Date 2023-09-14T04:19:29
9 Modification Date 2023-09-14T04:45:03
10 Spectrometer Frequency 100.53
11 Spectral Width 25252.5
12 Lowest Frequency -2571.1
13 Nucleus 13C
14 Acquired Size 32768
15 Spectral Size 52430
200 190 180 170 160 150 140 130 120 110 100 90 80 70 60 50 40 30 20 10 0 -10
f1 (ppm)

(R)-7-(4-acetylphenyl)-6,7-dihydro-5H-[1,4]oxazino[2,3,4-iJ]quinolin-3(2H)-one (18)

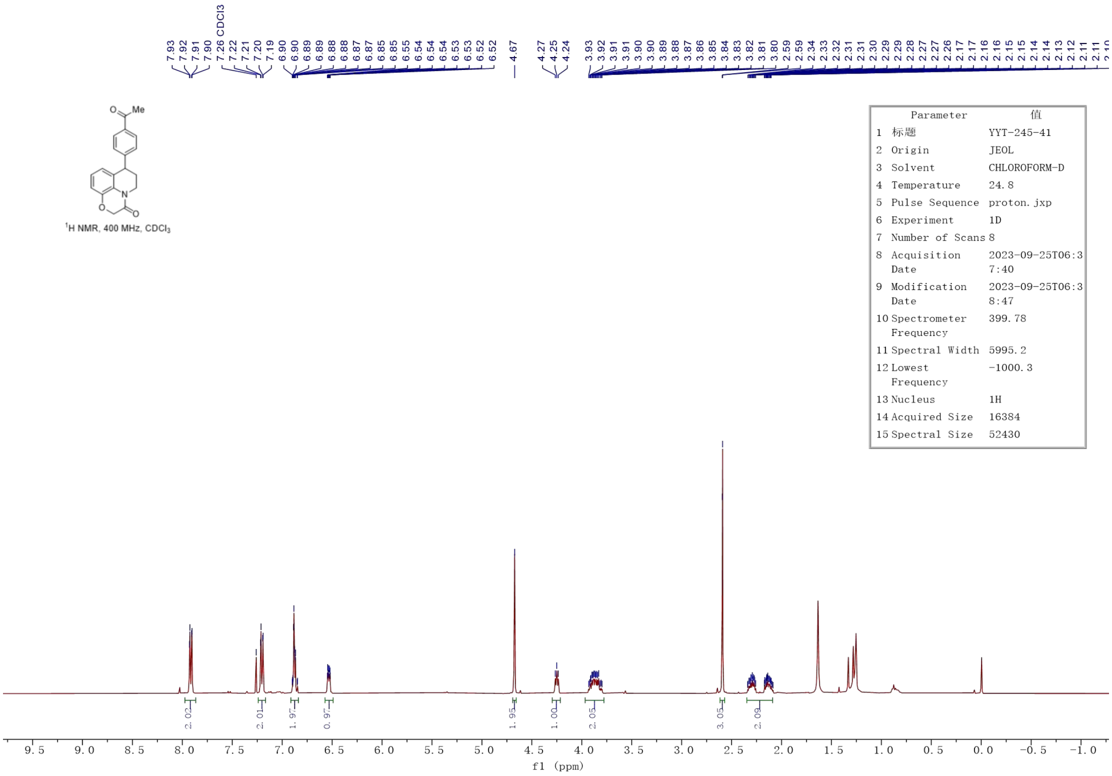

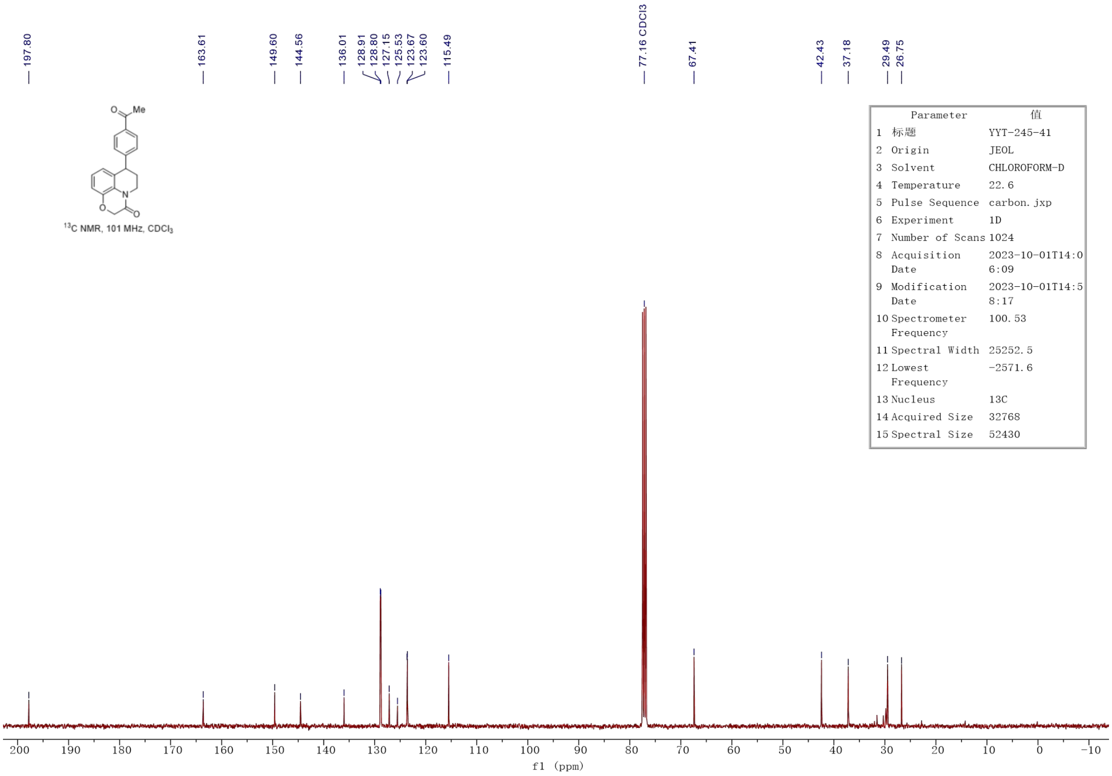

(R)-7-(4-nitrophenyl)-6,7-dihydro-5H-[1,4]oxazino[2,3,4-i*J*]quinolin-3(2H)-one (19)

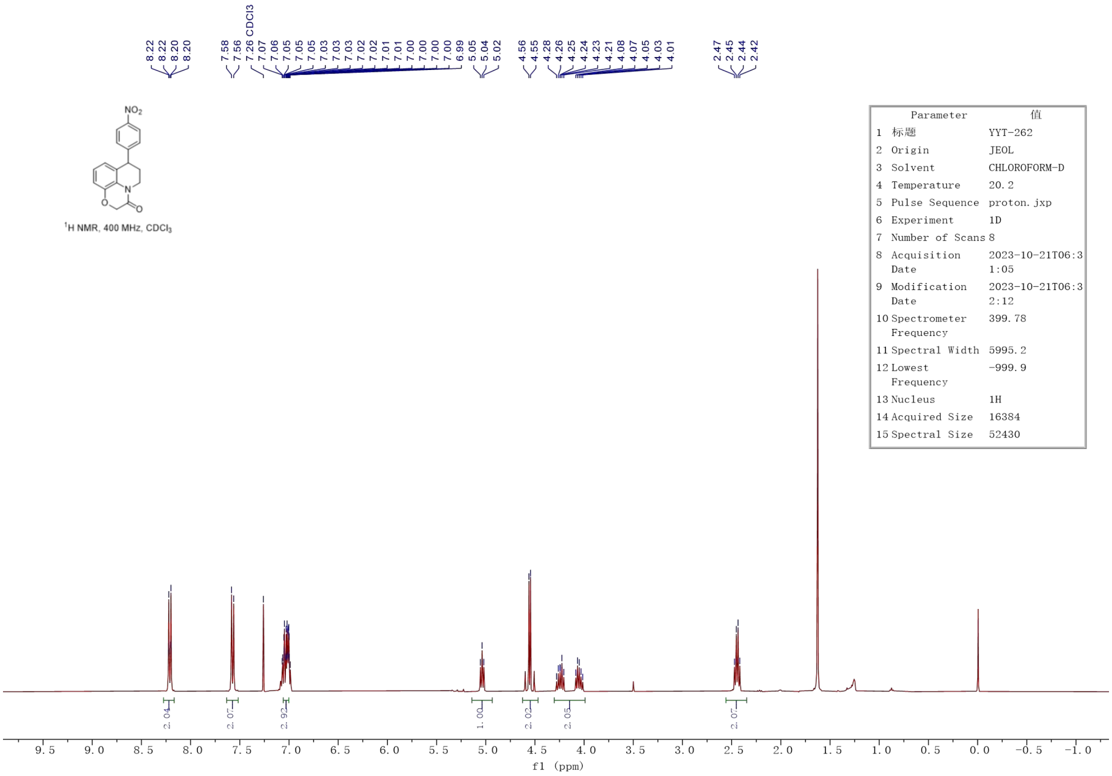

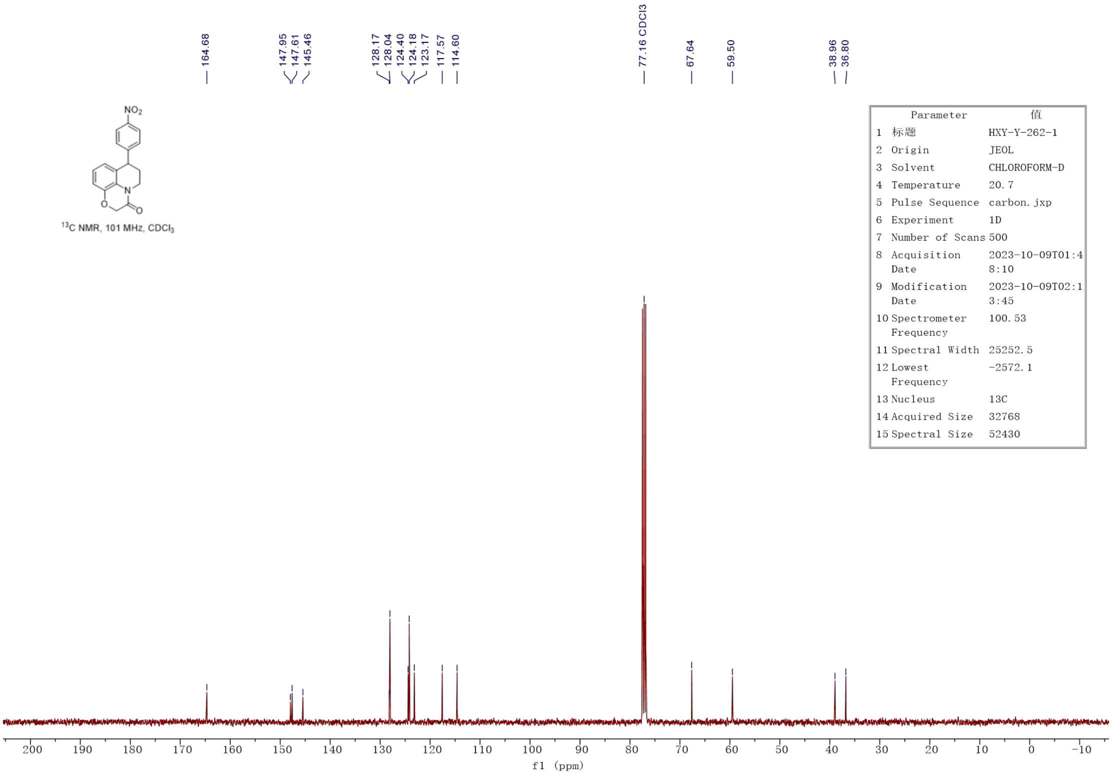

(R)-4-(3-oxo-2,3,6,7-tetrahydro-5H-[1,4]oxazino[2,3,4-i*J*]quinolin-7-yl)benzonitrile (20)

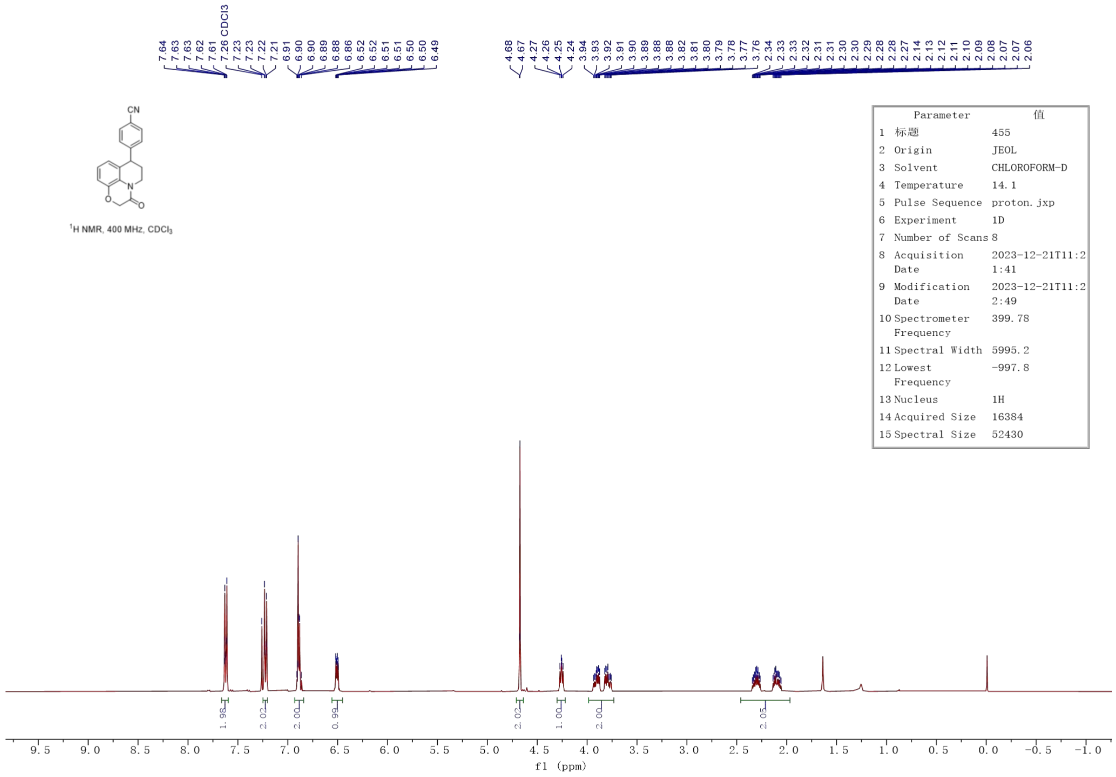

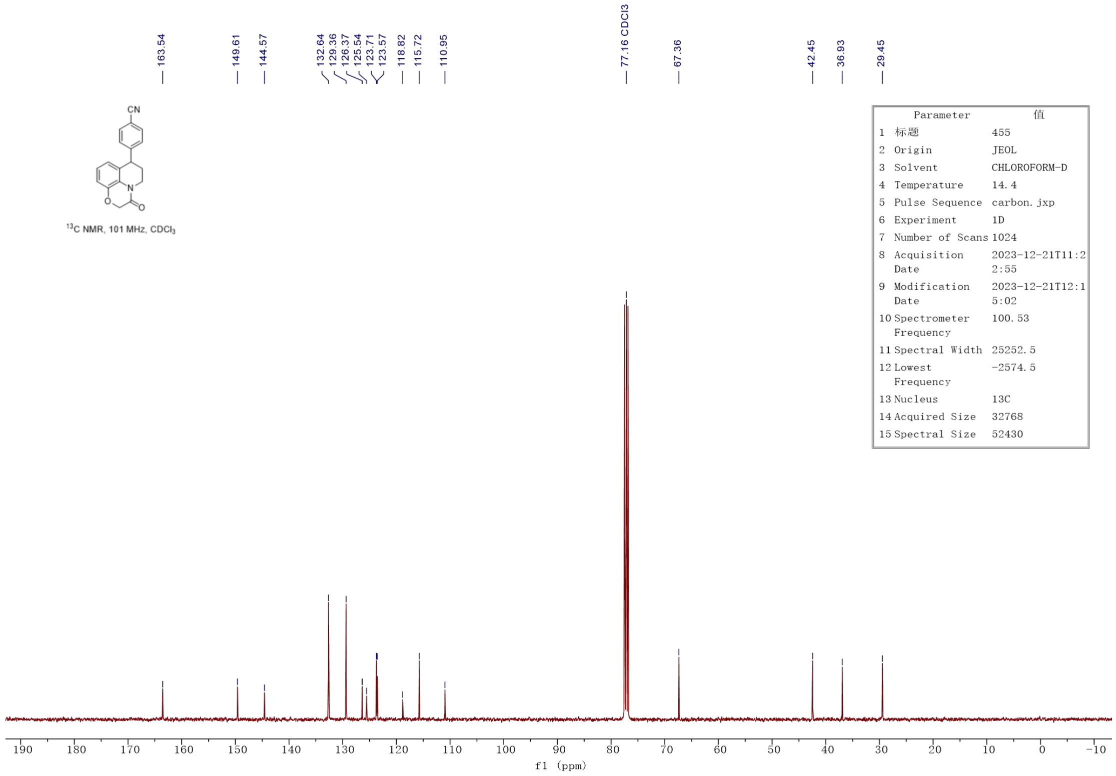

(R)-7-([1,1'-biphenyl]-4-yl)-6,7-dihydro-5H-[1,4]oxazino[2,3,4-i*J*]quinolin-3(2H)-one (21)

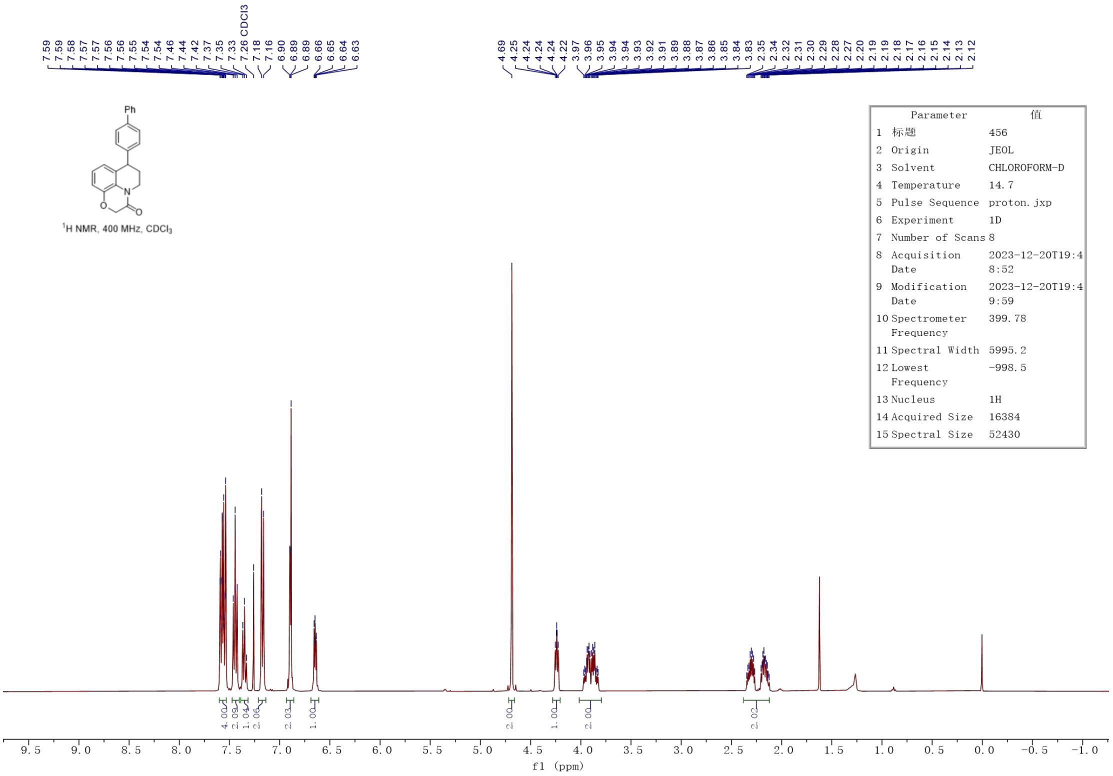

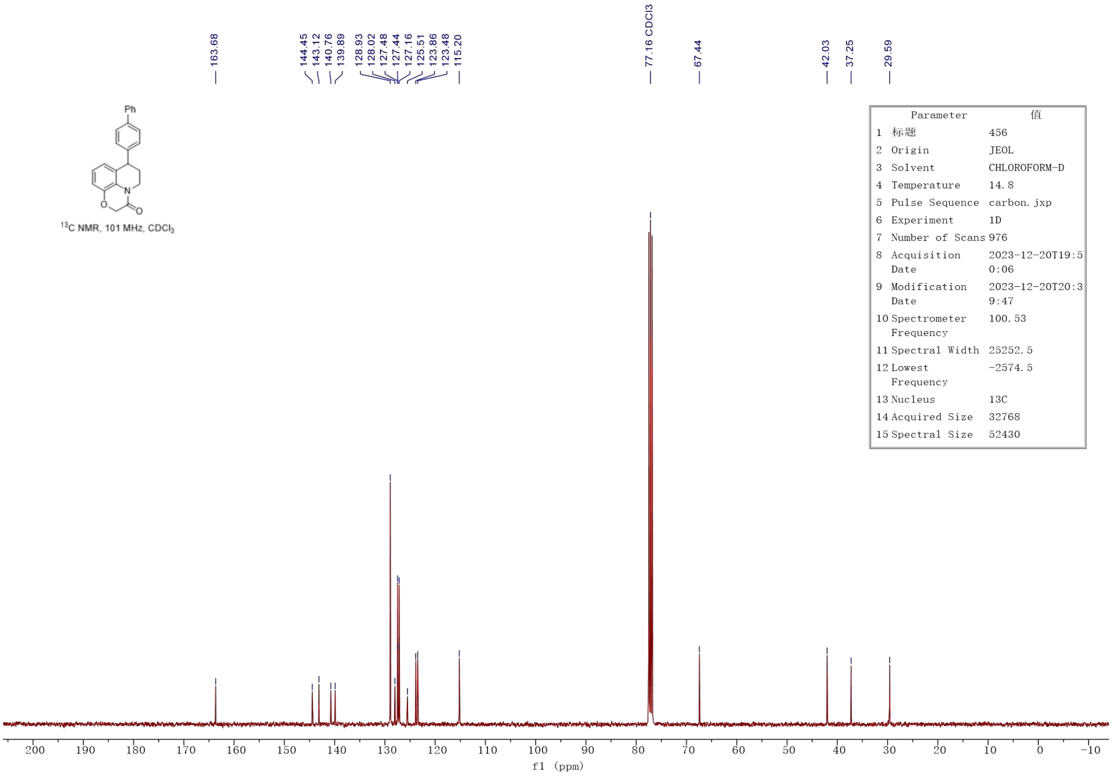

(R)-7-(4-fluorophenyl)-6,7-dihydro-5H-[1,4]oxazino[2,3,4-i*J*]quinolin-3(2H)-one (22)

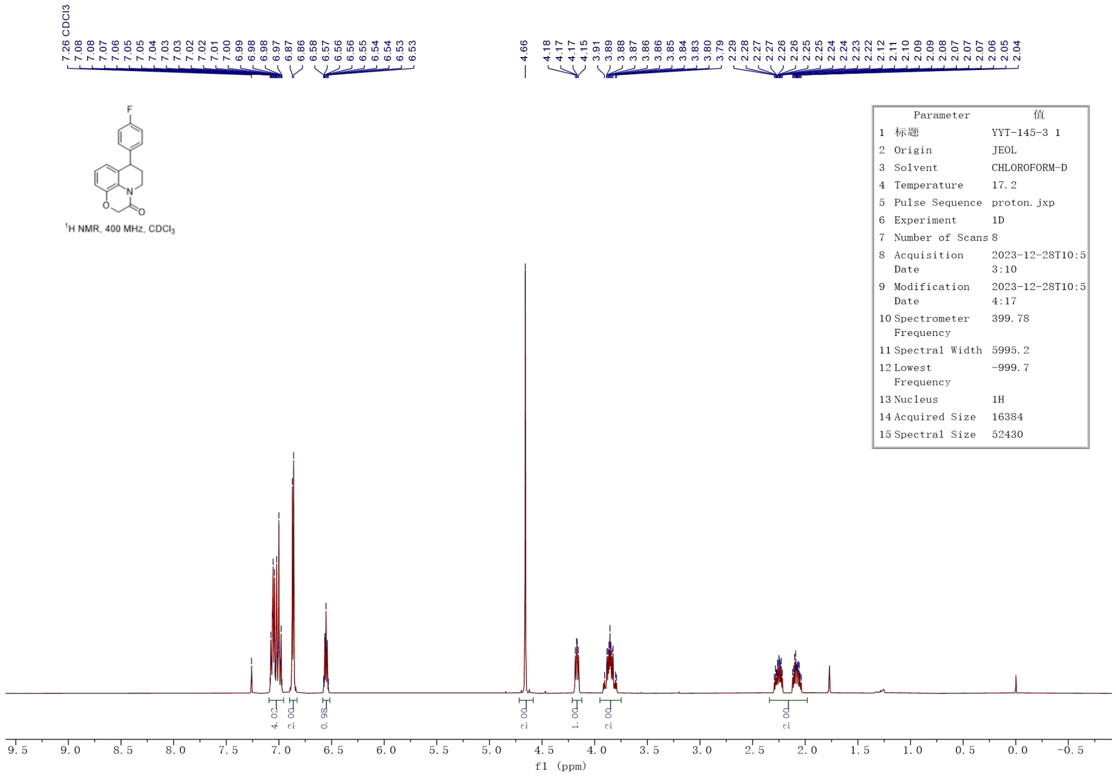

Parameter	値
1 标题	YYT-145-3 1
2 Origin	JEOL
3 Solvent	CHLOROFORM-D
4 Temperature	17.2
5 Pulse Sequence	proton.jxp
6 Experiment	1D
7 Number of Scans	8
8 Acquisition Date	2023-12-28T10:53:10
9 Modification Date	2023-12-28T10:54:17
10 Spectrometer Frequency	399.78
11 Spectral Width	5995.2
12 Lowest Frequency	-999.7
13 Nucleus	1H
14 Acquired Size	16384
15 Spectral Size	52430

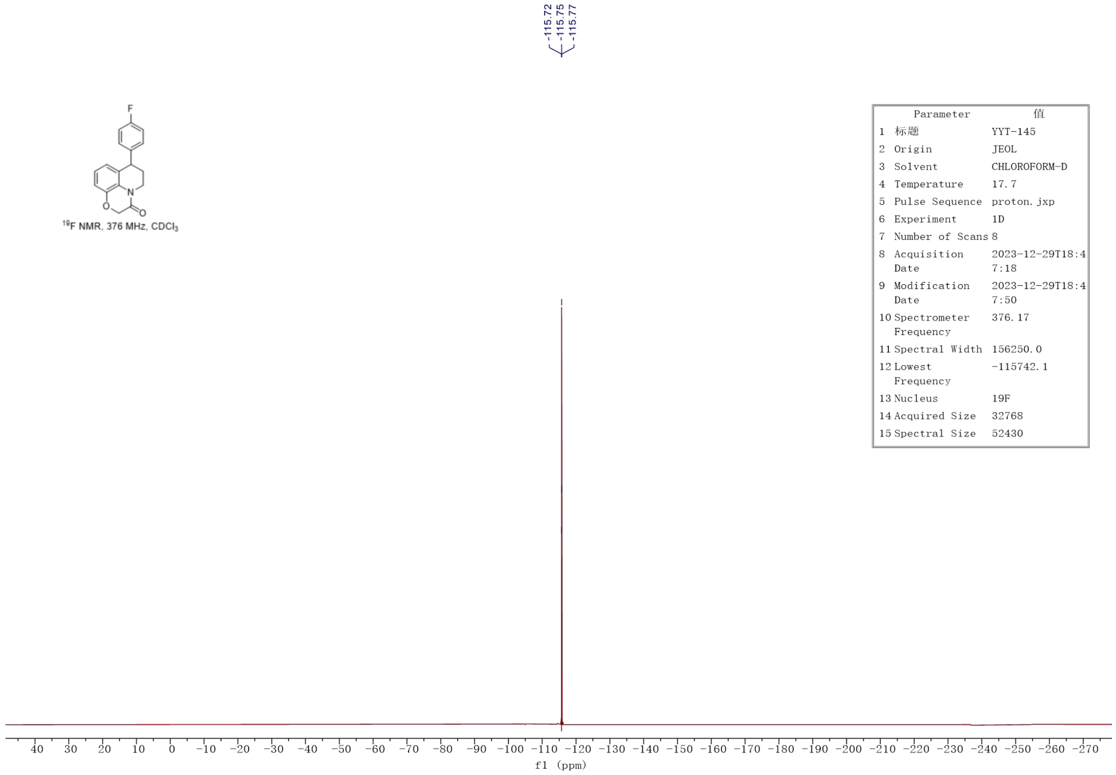

Parameter	値
1 标题	YYT-145
2 Origin	JEOL
3 Solvent	CHLOROFORM-D
4 Temperature	17.7
5 Pulse Sequence	proton.jxp
6 Experiment	1D
7 Number of Scans	8
8 Acquisition Date	2023-12-29T18:47:18
9 Modification Date	2023-12-29T18:47:50
10 Spectrometer Frequency	376.17
11 Spectral Width	156250.0
12 Lowest Frequency	-115742.1
13 Nucleus	19F
14 Acquired Size	32768
15 Spectral Size	52430

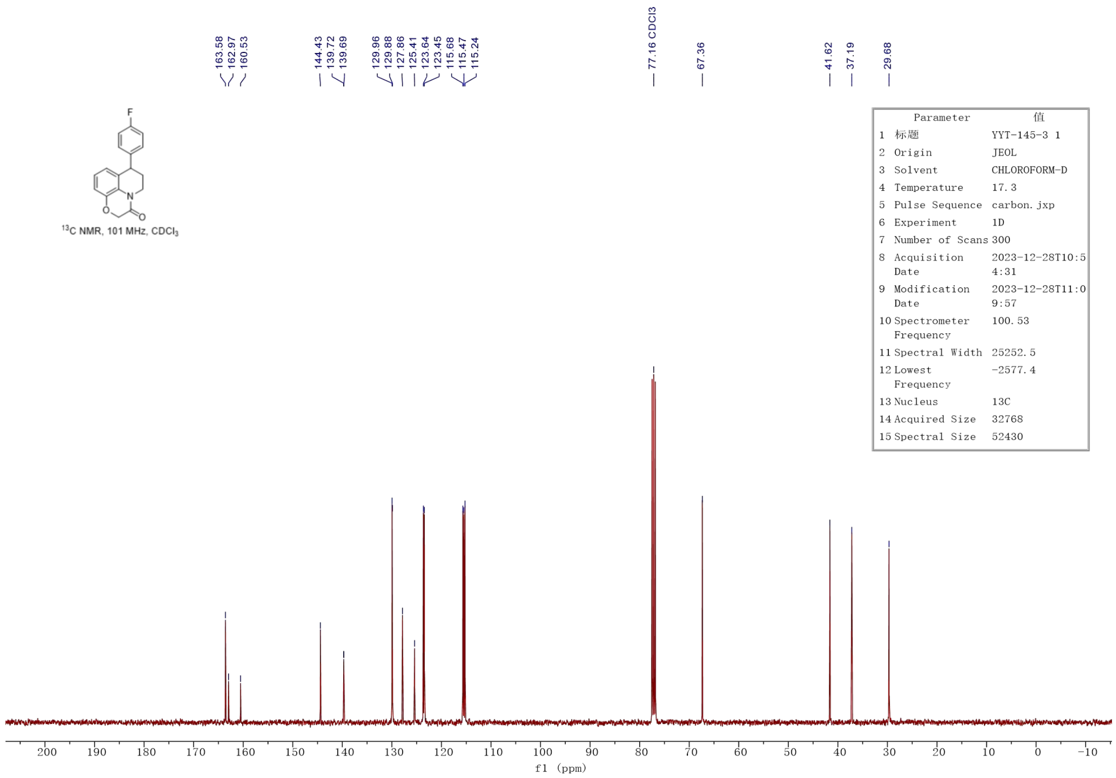

^{13}C NMR, 101 MHz, $CDCl_3$

	Parameter	值
1	标题	YYT-145-3 1
2	Origin	JEOL
3	Solvent	CHLOROFORM-D
4	Temperature	17.3
5	Pulse Sequence	carbon.jxp
6	Experiment	1D
7	Number of Scans	300
8	Acquisition Date	2023-12-28T10:54:31
9	Modification Date	2023-12-28T11:09:57
10	Spectrometer Frequency	100.53
11	Spectral Width	25252.5
12	Lowest Frequency	-2577.4
13	Nucleus	13C
14	Acquired Size	32768
15	Spectral Size	52430

(R)-7-(4-chlorophenyl)-6,7-dihydro-5H-[1,4]oxazino[2,3,4-i*J*]quinolin-3(2H)-one (23)

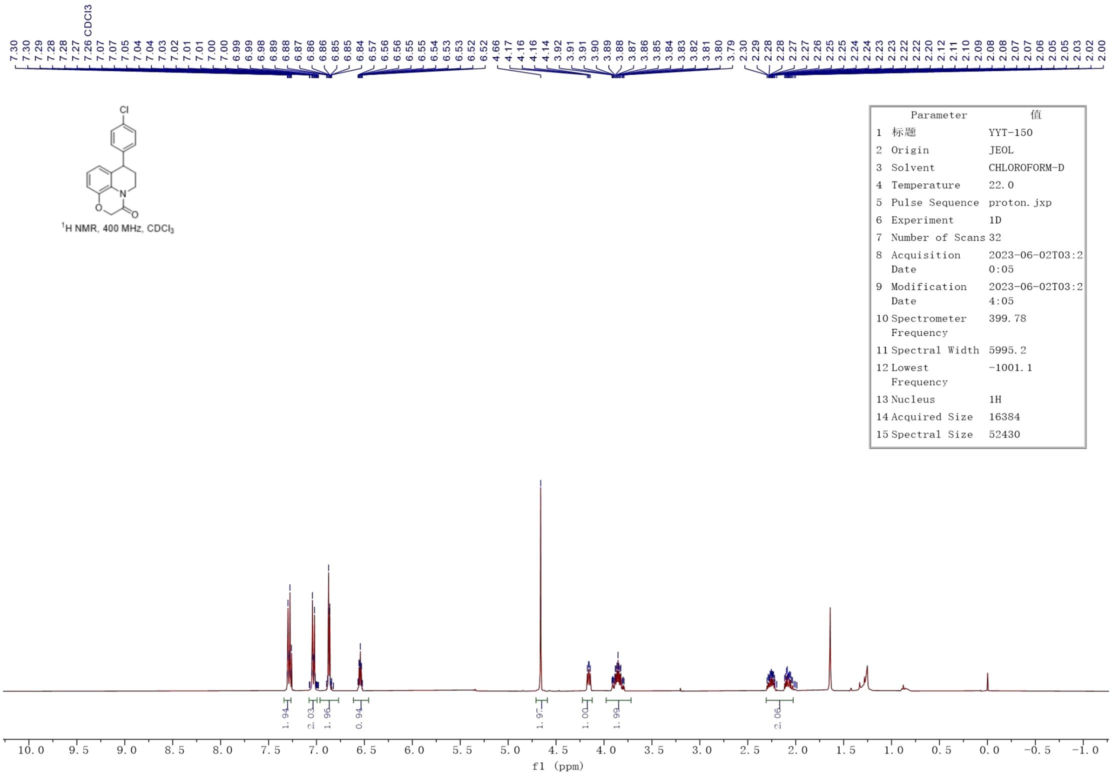

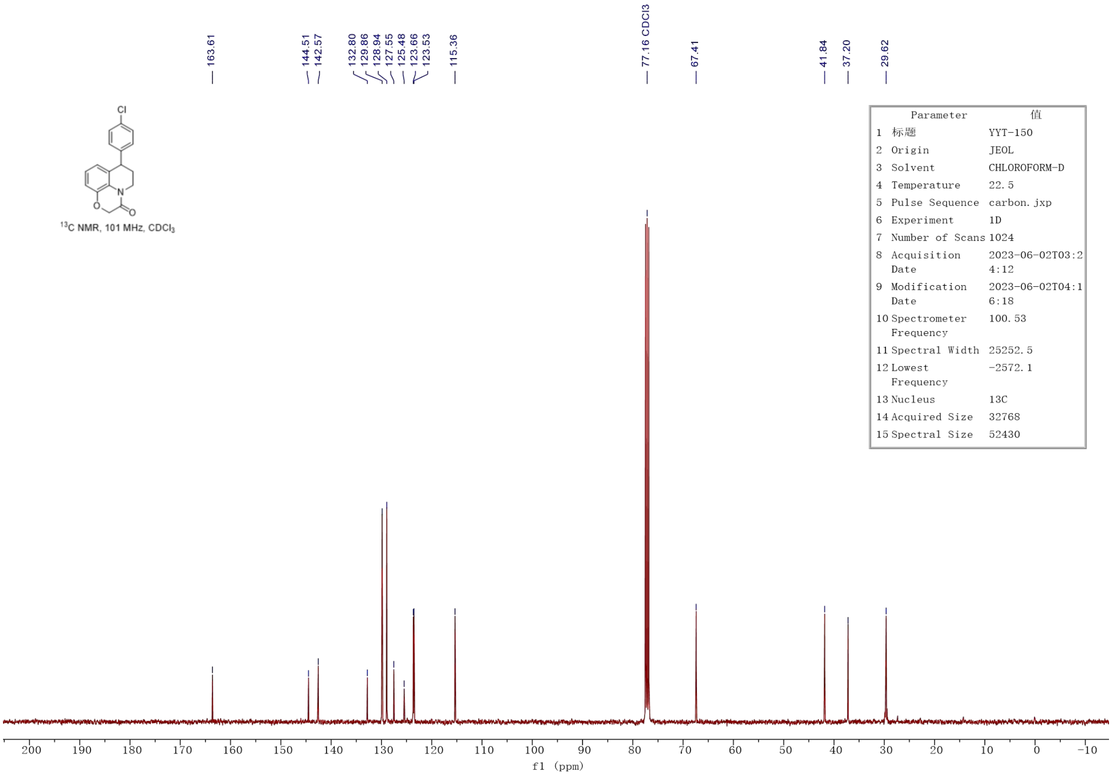

(R)-7-(4-bromophenyl)-6,7-dihydro-5H-[1,4]oxazino[2,3,4-i*J*]quinolin-3(2H)-one (24)

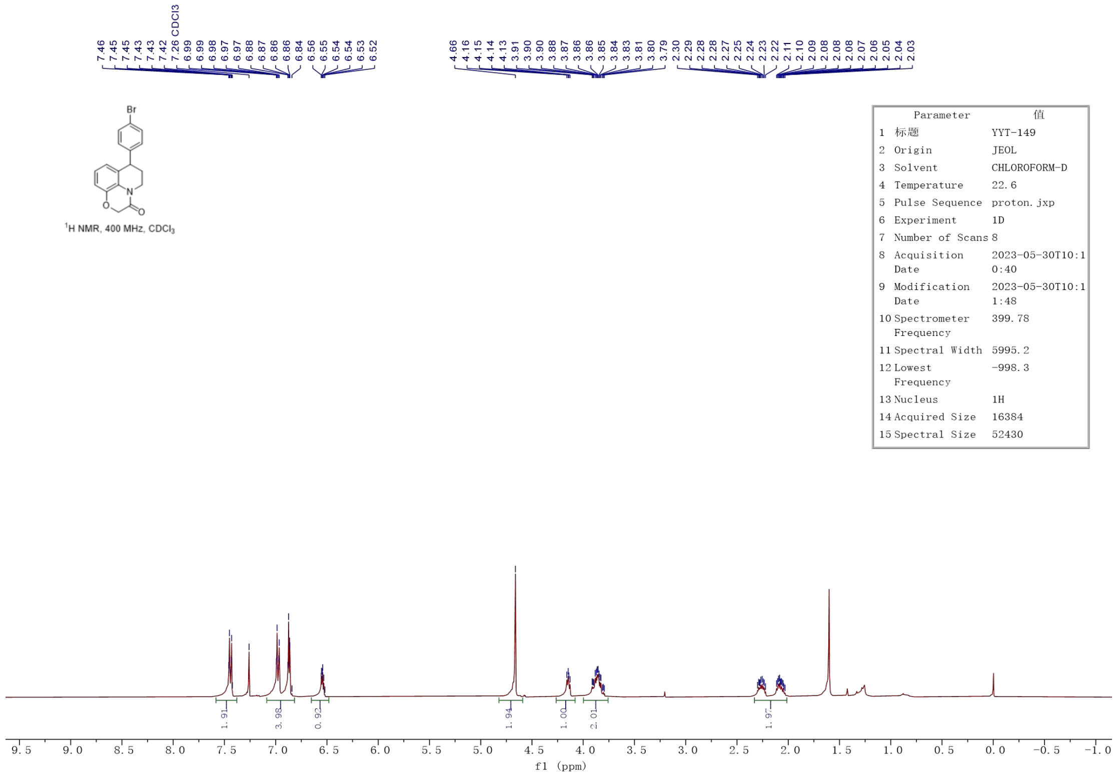

	Parameter	值
1	标题	YYT-149
2	Origin	JEOL
3	Solvent	CHLOROFORM-D
4	Temperature	22.6
5	Pulse Sequence	proton.jxp
6	Experiment	1D
7	Number of Scans	8
8	Acquisition Date	2023-05-30T10:10:40
9	Modification Date	2023-05-30T10:11:48
10	Spectrometer Frequency	399.78
11	Spectral Width	5995.2
12	Lowest Frequency	-998.3
13	Nucleus	1H
14	Acquired Size	16384
15	Spectral Size	52430

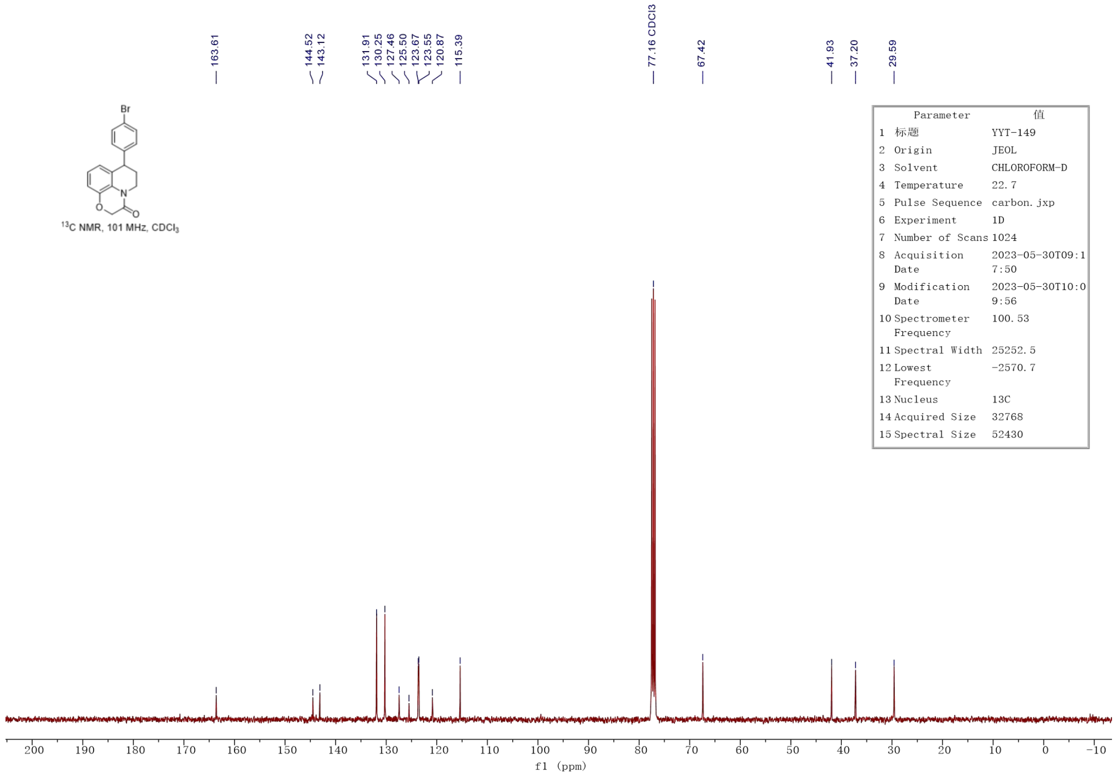

	Parameter	值
1	标题	YYT-149
2	Origin	JEOL
3	Solvent	CHLOROFORM-D
4	Temperature	22.7
5	Pulse Sequence	carbon.jxp
6	Experiment	1D
7	Number of Scans	1024
8	Acquisition Date	2023-05-30T09:17:50
9	Modification Date	2023-05-30T10:09:56
10	Spectrometer Frequency	100.53
11	Spectral Width	25252.5
12	Lowest Frequency	-2570.7
13	Nucleus	13C
14	Acquired Size	32768
15	Spectral Size	52430

(R)-7-(3-isopropylphenyl)-6,7-dihydro-5H-[1,4]oxazino[2,3,4-i*J*]quinolin-3(2H)-one (25)

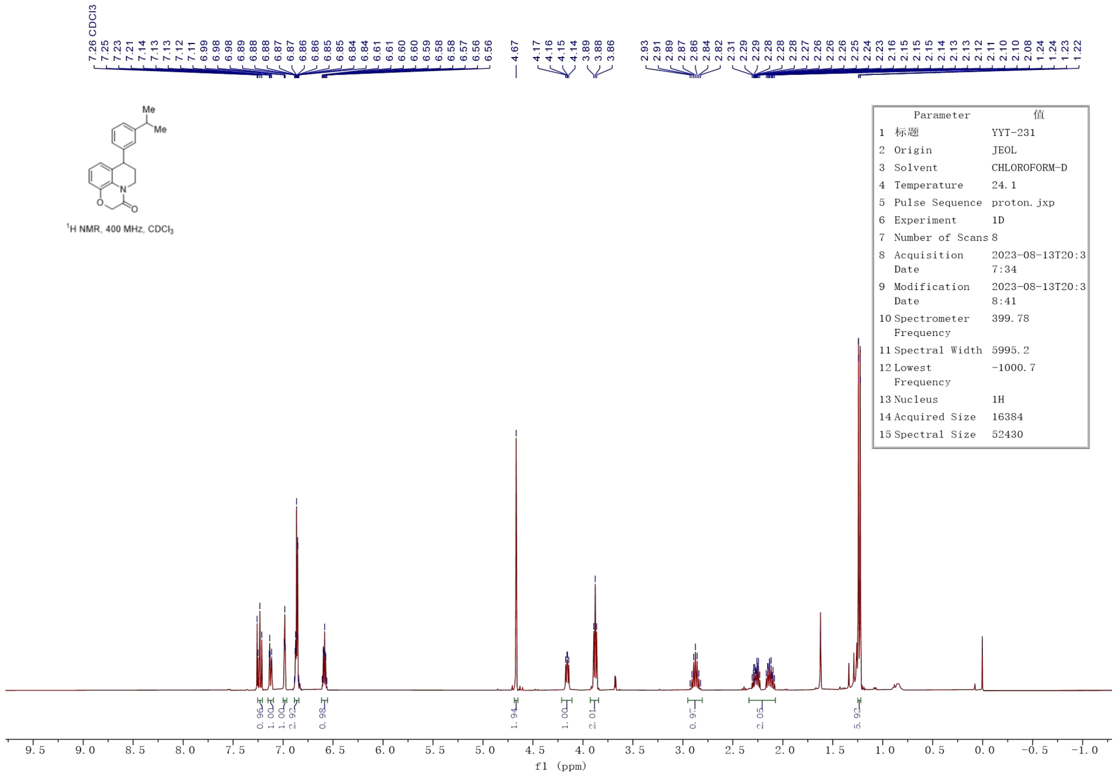

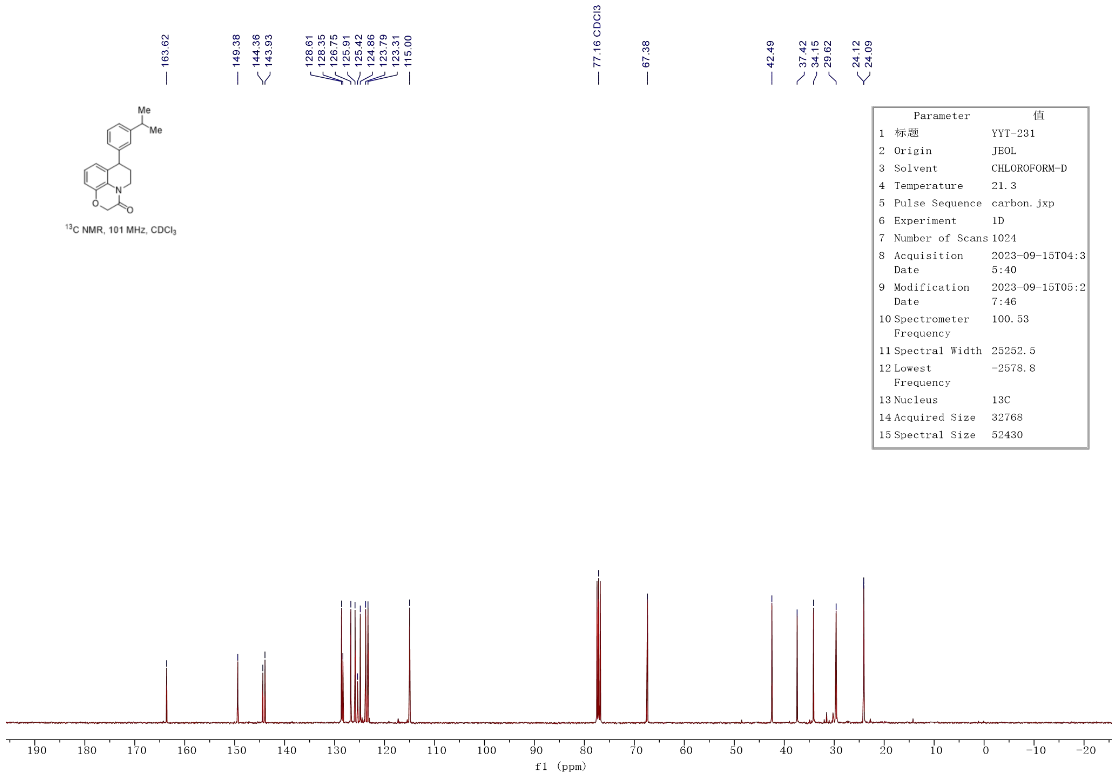

(R)-7-(m-tolyl)-6,7-dihydro-5H-[1,4]oxazino[2,3,4-i*J*]quinolin-3(2H)-one (26)

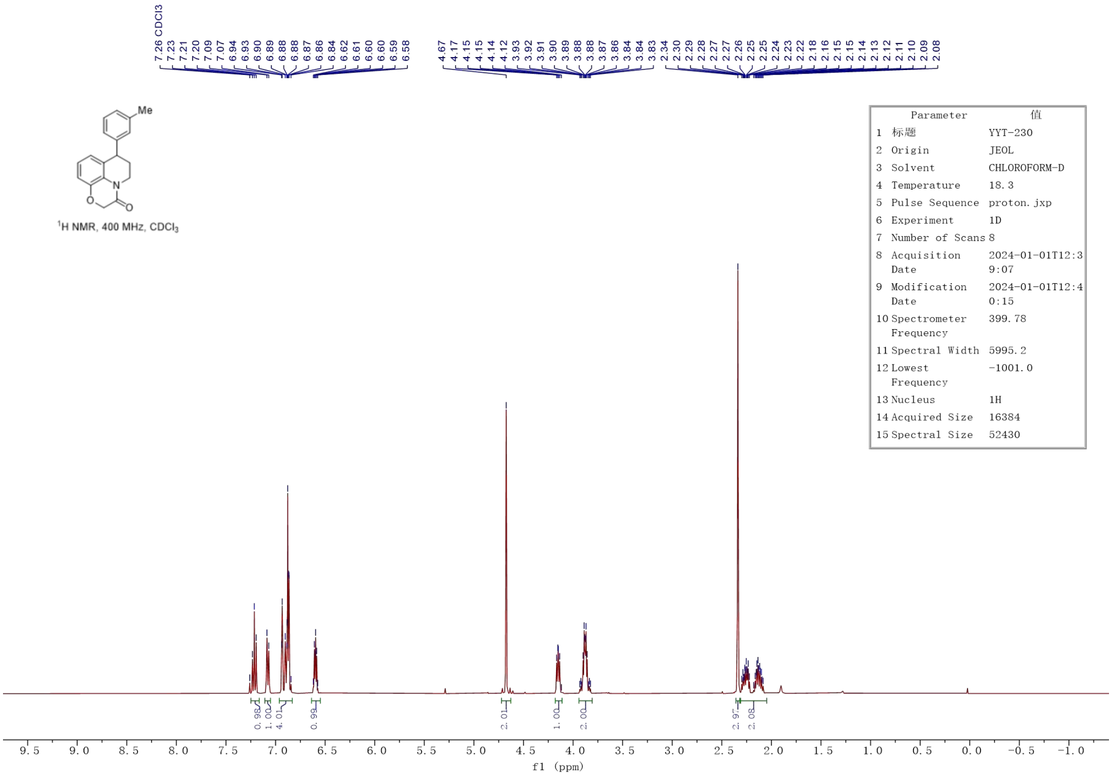

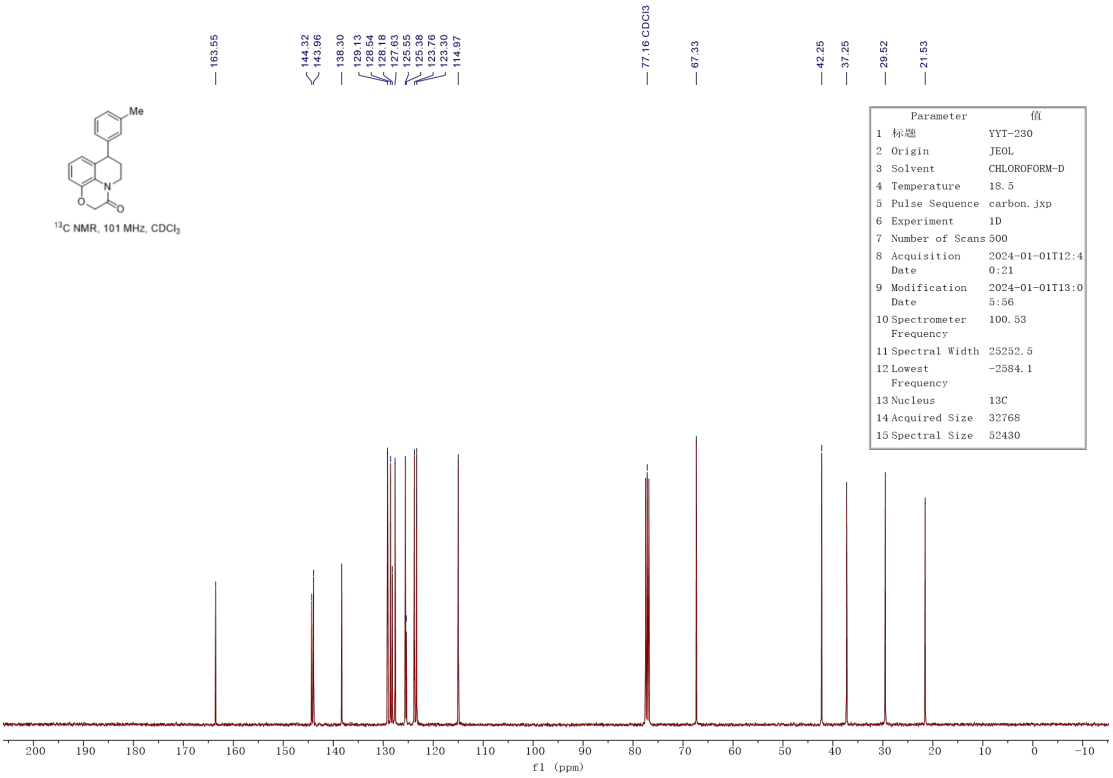

(R)-7-(3-chlorophenyl)-6,7-dihydro-5H-[1,4]oxazino[2,3,4-i*J*]quinolin-3(2H)-one (27)

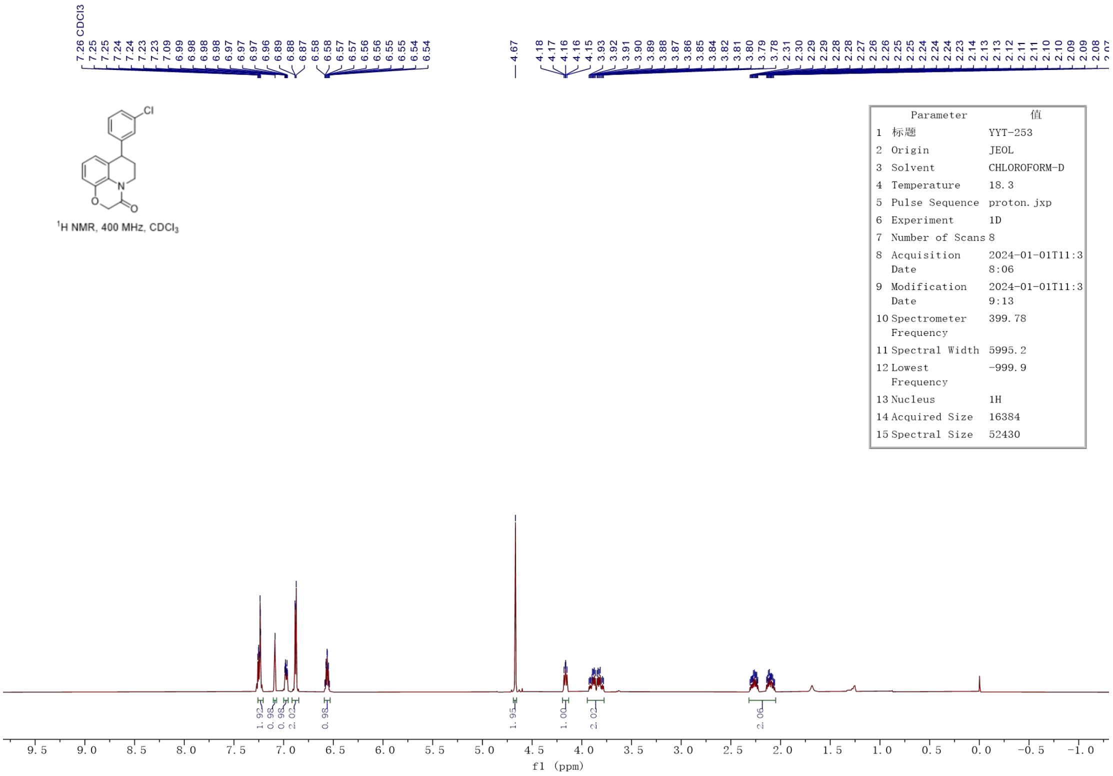

Parameter	值
1 标题	YYT-253
2 Origin	JEOL
3 Solvent	CHLOROFORM-D
4 Temperature	18.3
5 Pulse Sequence	proton.jxp
6 Experiment	1D
7 Number of Scans	8
8 Acquisition Date	2024-01-01T11:38:06
9 Modification Date	2024-01-01T11:39:13
10 Spectrometer Frequency	399.78
11 Spectral Width	5995.2
12 Lowest Frequency	-999.9
13 Nucleus	1H
14 Acquired Size	16384
15 Spectral Size	52430

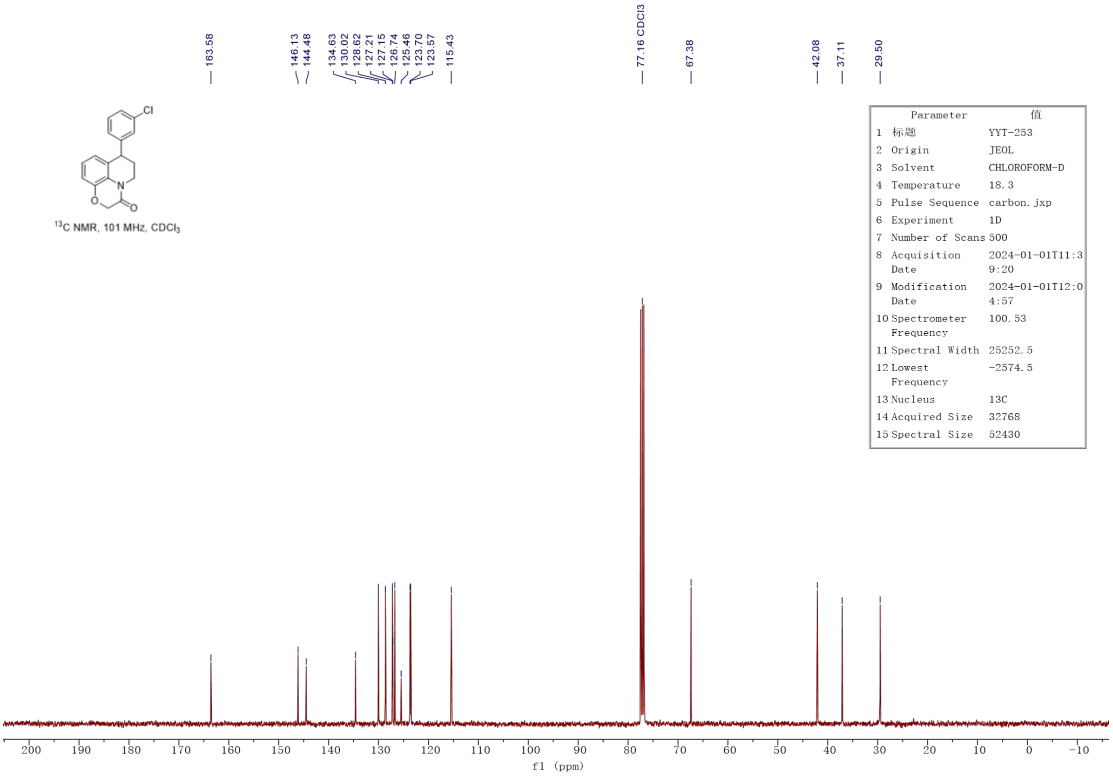

Parameter	值
1 标题	YYT-253
2 Origin	JEOL
3 Solvent	CHLOROFORM-D
4 Temperature	18.3
5 Pulse Sequence	carbon.jxp
6 Experiment	1D
7 Number of Scans	500
8 Acquisition Date	2024-01-01T11:39:20
9 Modification Date	2024-01-01T12:04:57
10 Spectrometer Frequency	100.53
11 Spectral Width	25252.5
12 Lowest Frequency	-2574.5
13 Nucleus	13C
14 Acquired Size	32768
15 Spectral Size	52430

(R)-7-(3-bromophenyl)-6,7-dihydro-5H-[1,4]oxazino[2,3,4-i*J*]quinolin-3(2H)-one (28)

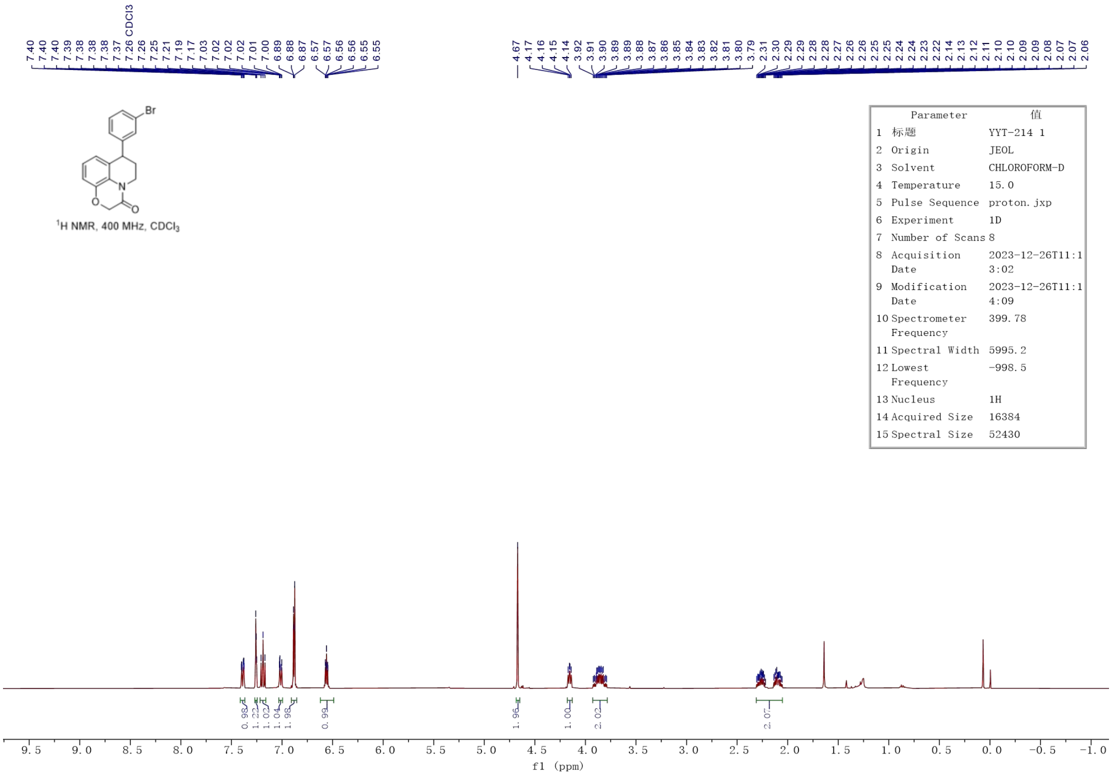

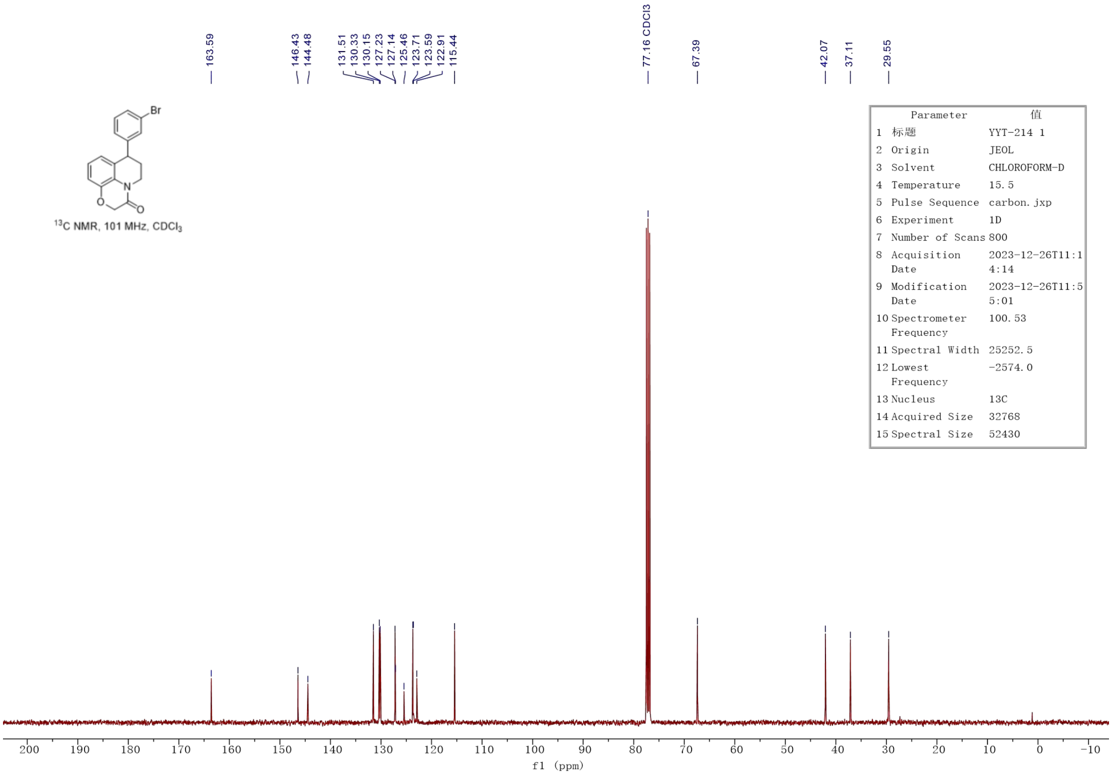

(R)-7-(3-fluorophenyl)-6,7-dihydro-5H-[1,4]oxazino[2,3,4-i*J*]quinolin-3(2H)-one (29)

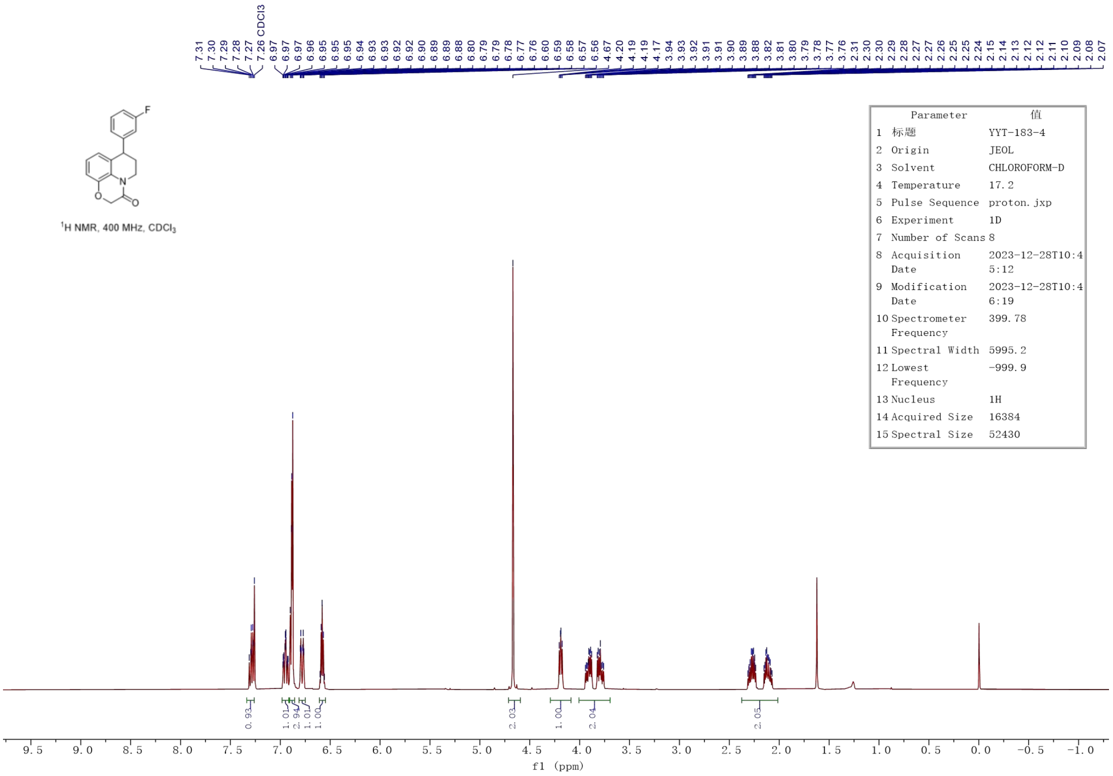

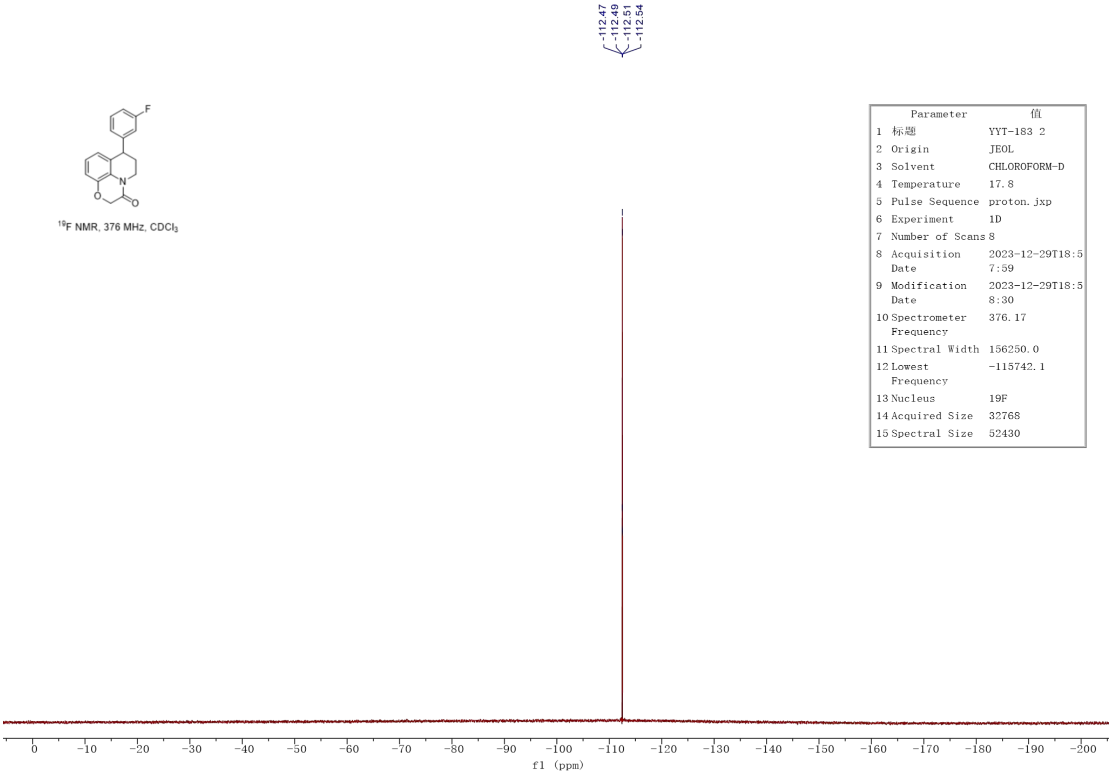

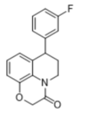

^{13}C NMR, 101 MHz, $CDCl_3$

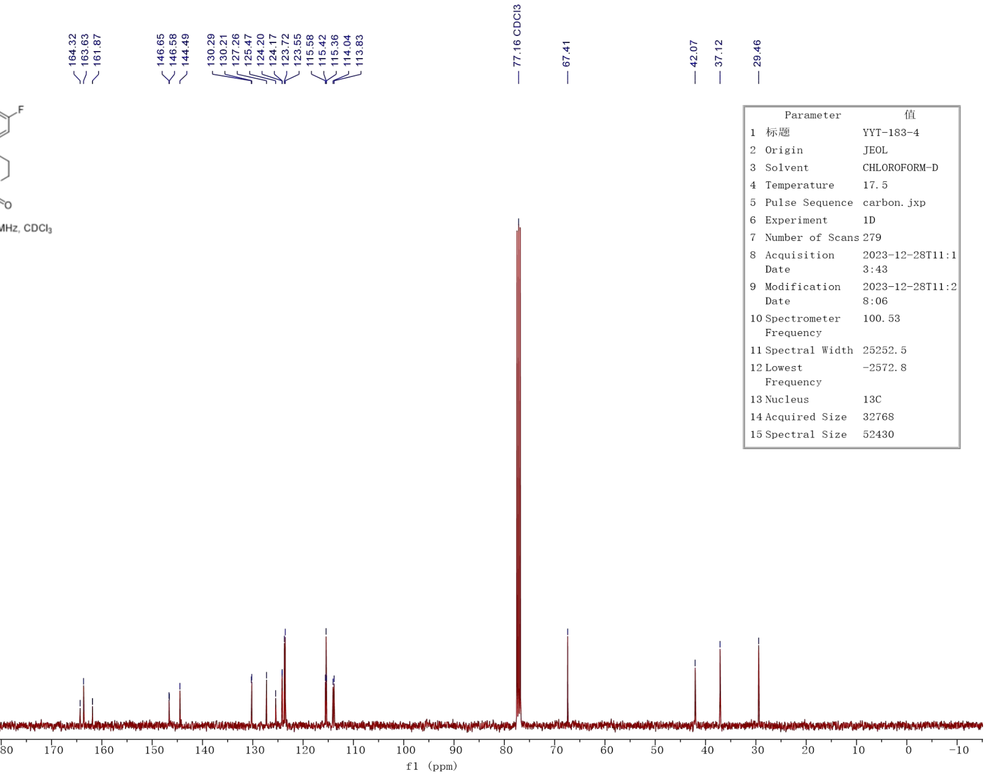

	Parameter	值
1	标题	YYT-183-4
2	Origin	JEOL
3	Solvent	CHLOROFORM-D
4	Temperature	17.5
5	Pulse Sequence	carbon.jxp
6	Experiment	1D
7	Number of Scans	279
8	Acquisition Date	2023-12-28T11:13:43
9	Modification Date	2023-12-28T11:28:06
10	Spectrometer Frequency	100.53
11	Spectral Width	25252.5
12	Lowest Frequency	-2572.8
13	Nucleus	13C
14	Acquired Size	32768
15	Spectral Size	52430

(R)-7-(3-(methylsulfonyl)phenyl)-6,7-dihydro-5H-[1,4]oxazino[2,3,4-ij]quinolin-3(2H)-one (30)

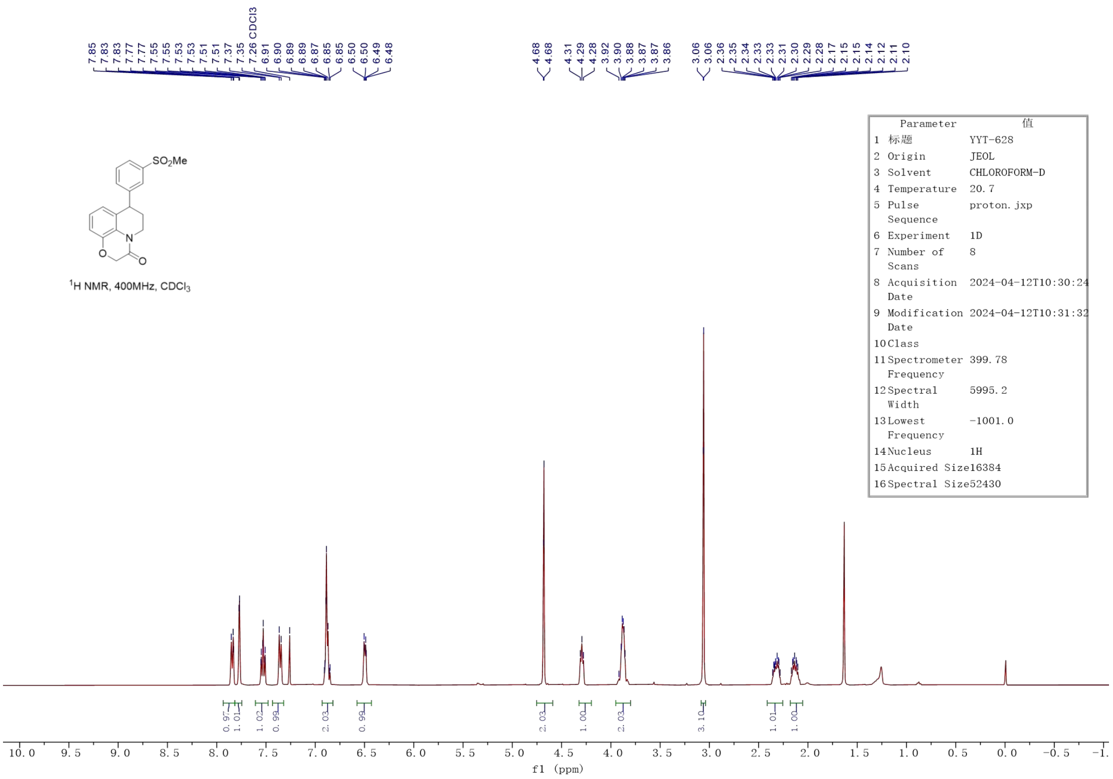

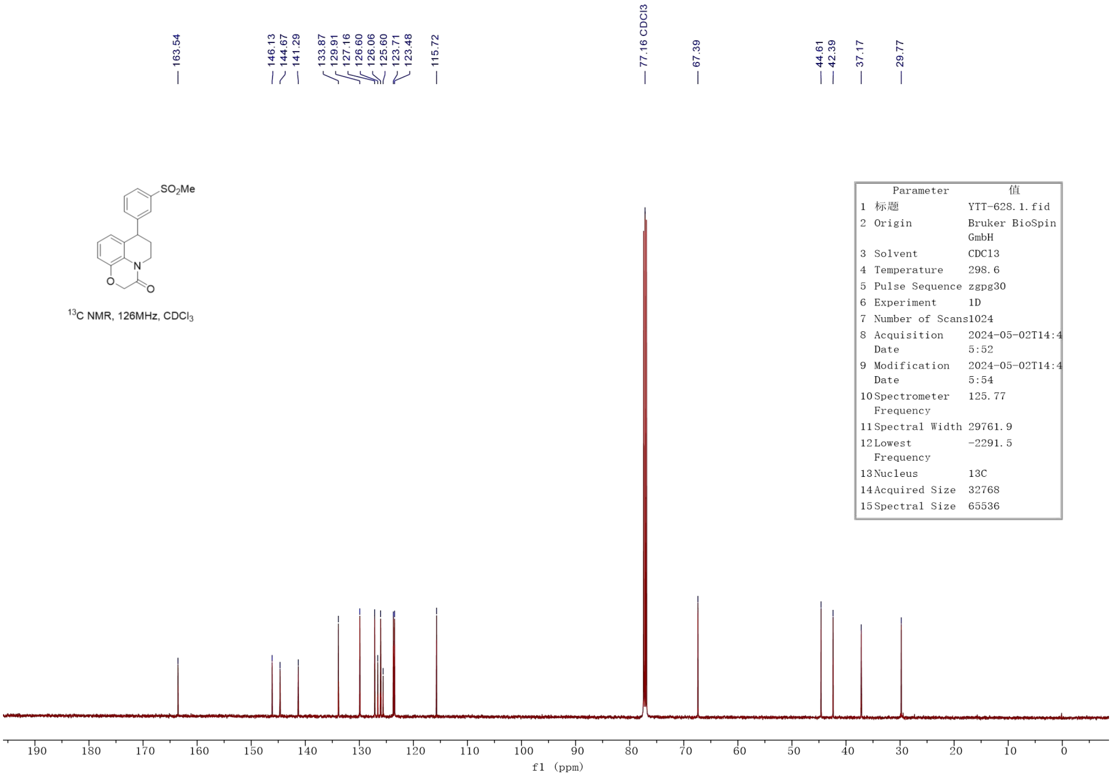

(R)-7-(2-chloro-4-fluorophenyl)-6,7-dihydro-5H-[1,4]oxazino[2,3,4-i*J*]quinolin-3(2H)-one (31)

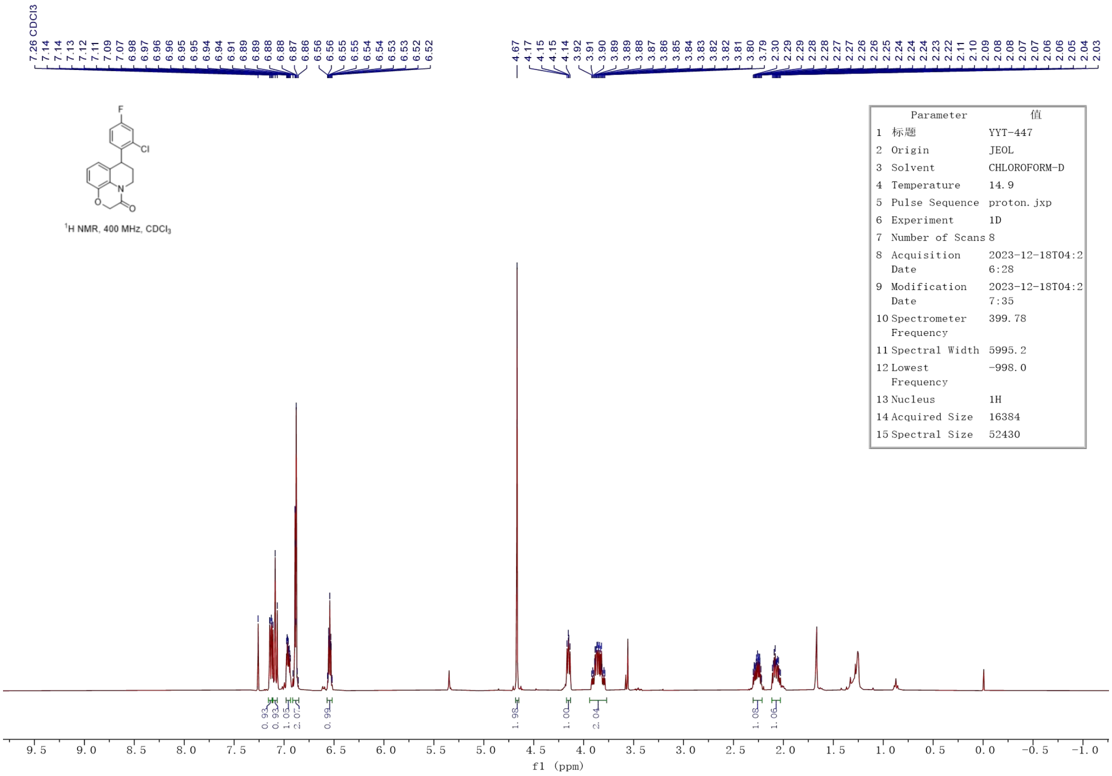

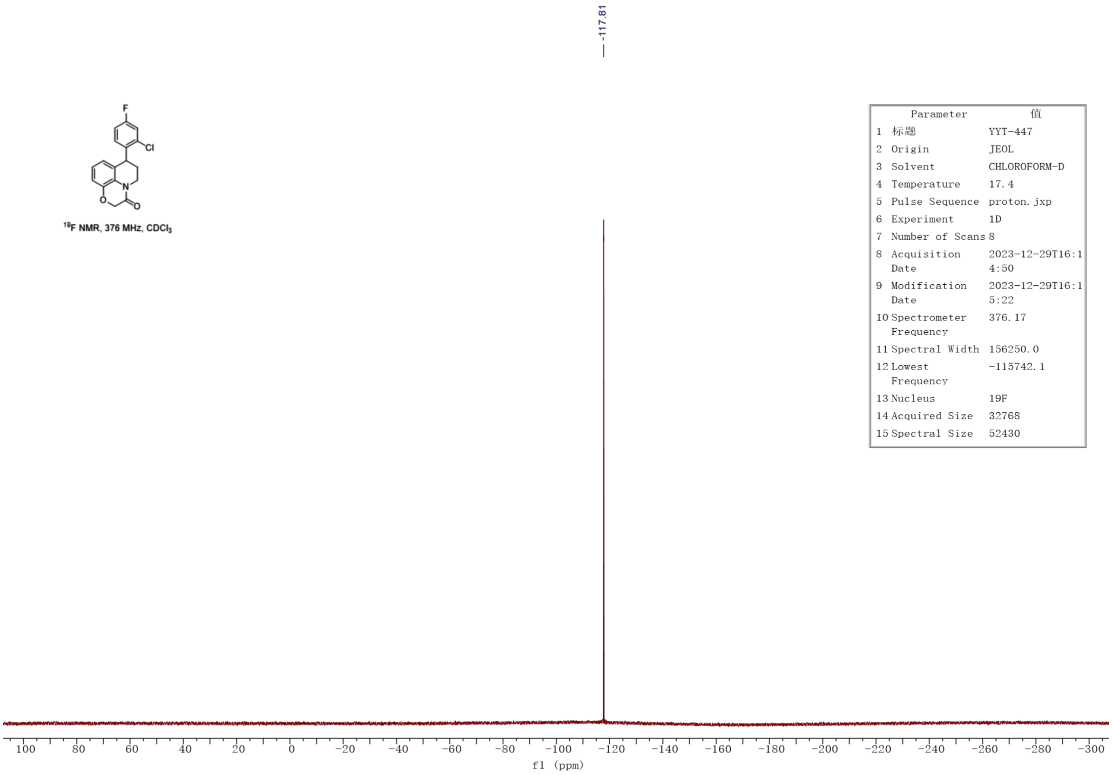

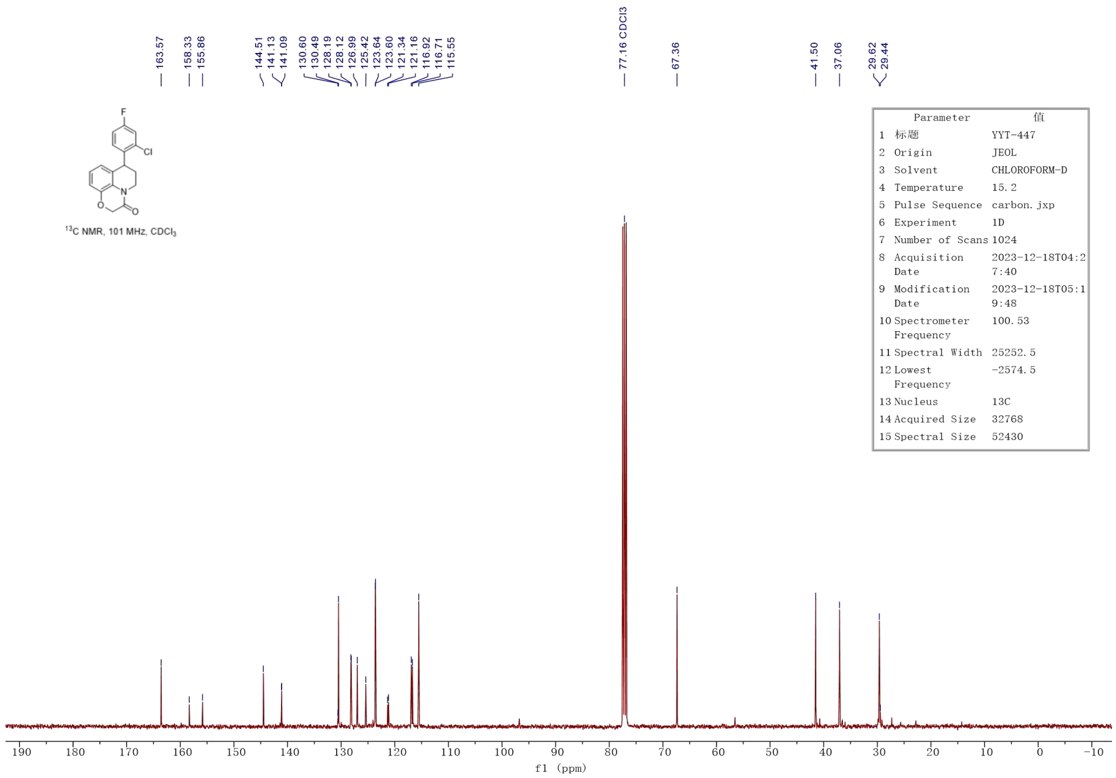

	Parameter	值
1	标题	YYT-447
2	Origin	JEOL
3	Solvent	CHLOROFORM-D
4	Temperature	15.2
5	Pulse Sequence	carbon.jxp
6	Experiment	1D
7	Number of Scans	1024
8	Acquisition Date	2023-12-18T04:27:40
9	Modification Date	2023-12-18T05:19:48
10	Spectrometer Frequency	100.53
11	Spectral Width	25252.5
12	Lowest Frequency	-2574.5
13	Nucleus	13C
14	Acquired Size	32768
15	Spectral Size	52430

(R)-7-(3-chloro-2-methylphenyl)-6,7-dihydro-5H-[1,4]oxazino[2,3,4-i*J*]quinolin-3(2H)-one (32)

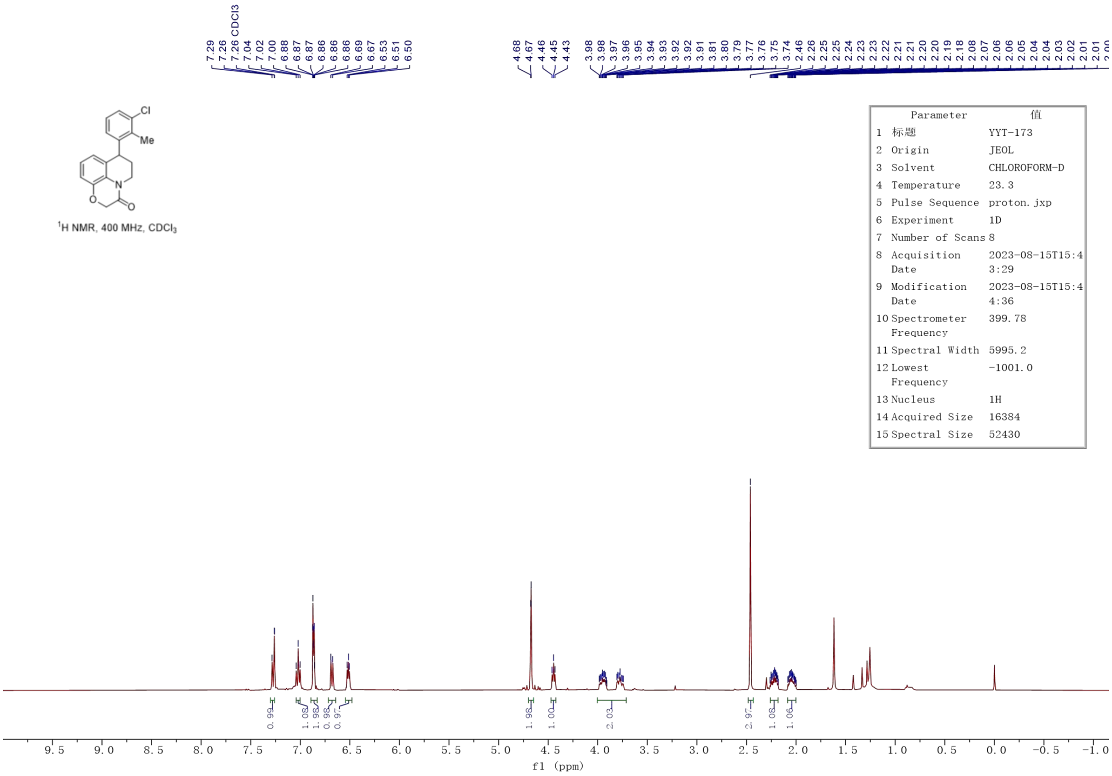

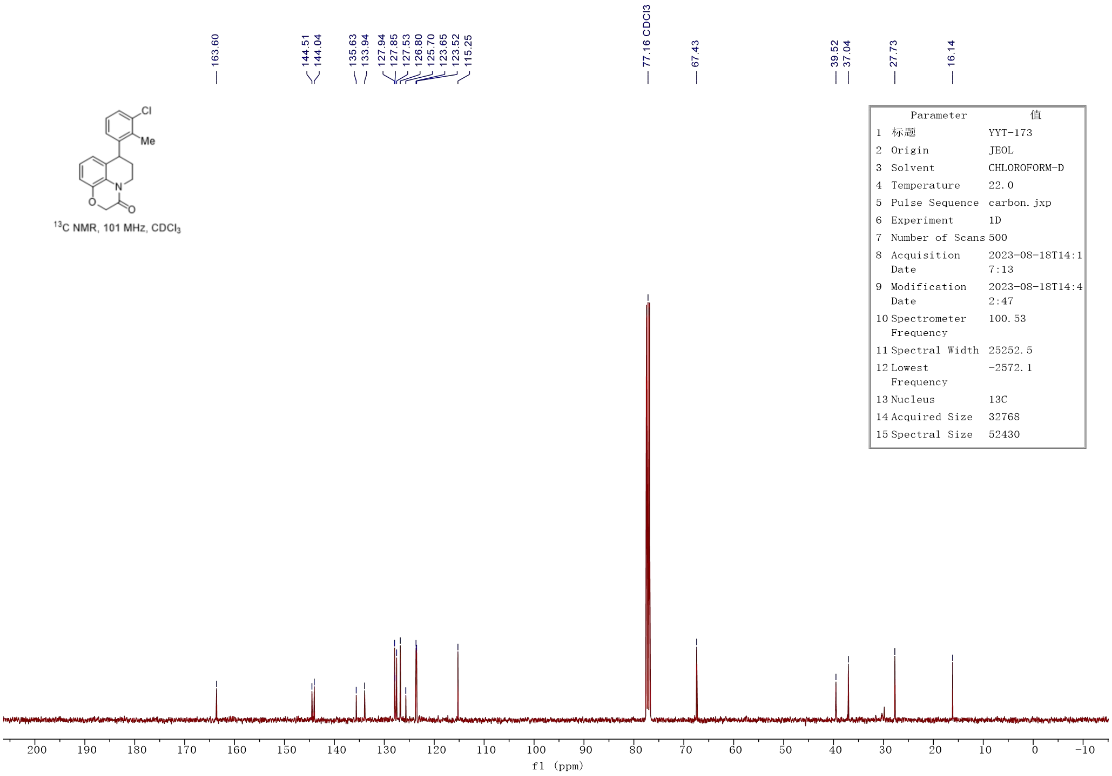

(R)-7-(4-bromo-3-methylphenyl)-6,7-dihydro-5H-[1,4]oxazino[2,3,4-i*J*]quinolin-3 (2H)-one (33)

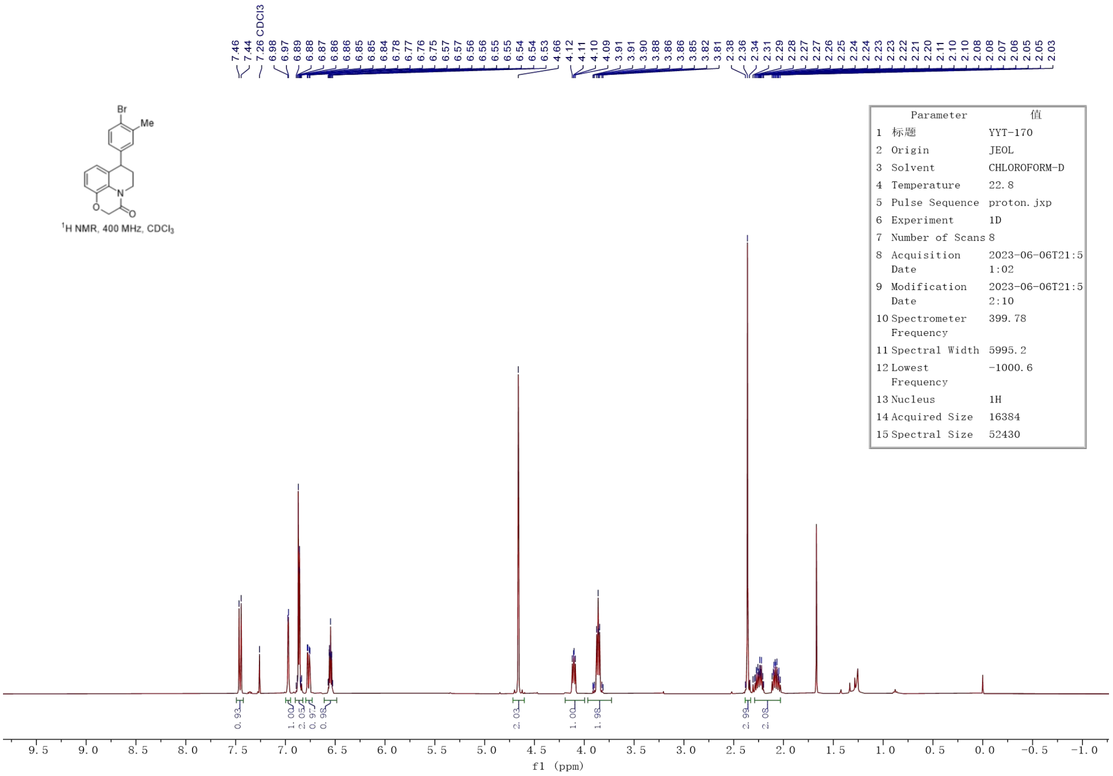

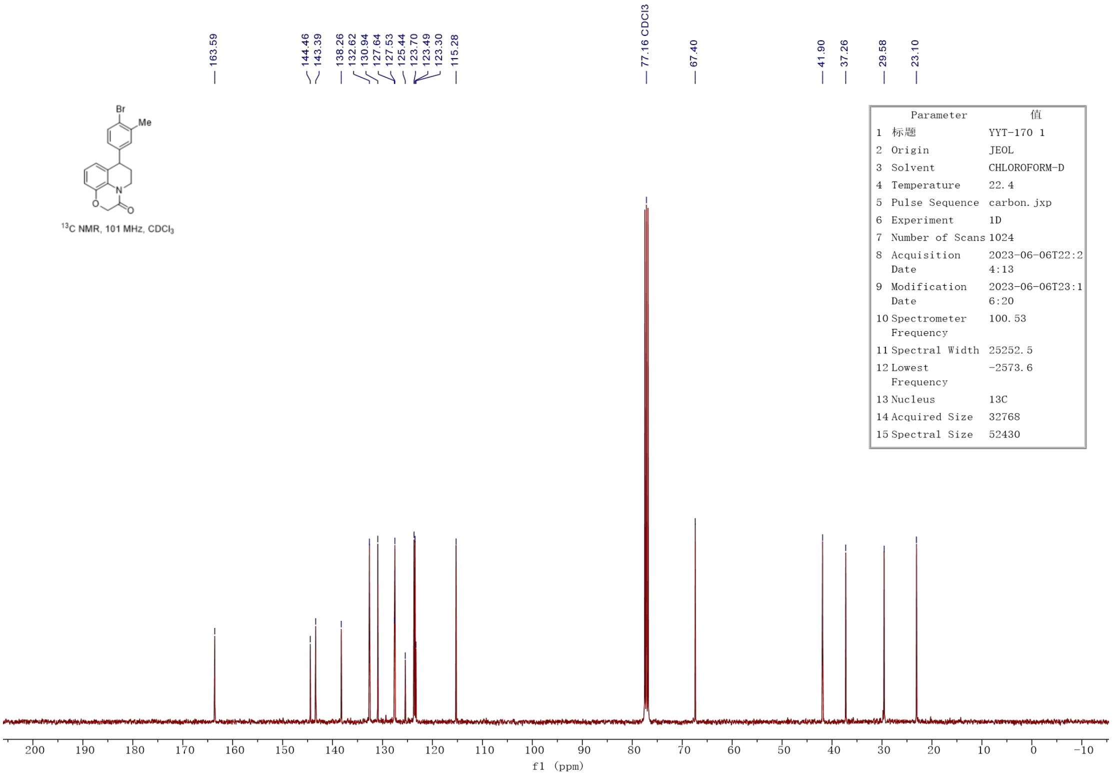

(R)-7-(4-fluoro-3-(trifluoromethyl)phenyl)-6,7-dihydro-5H-[1,4]oxazino[2,3,4-i*J*] quinolin-3(2H)-one (34)

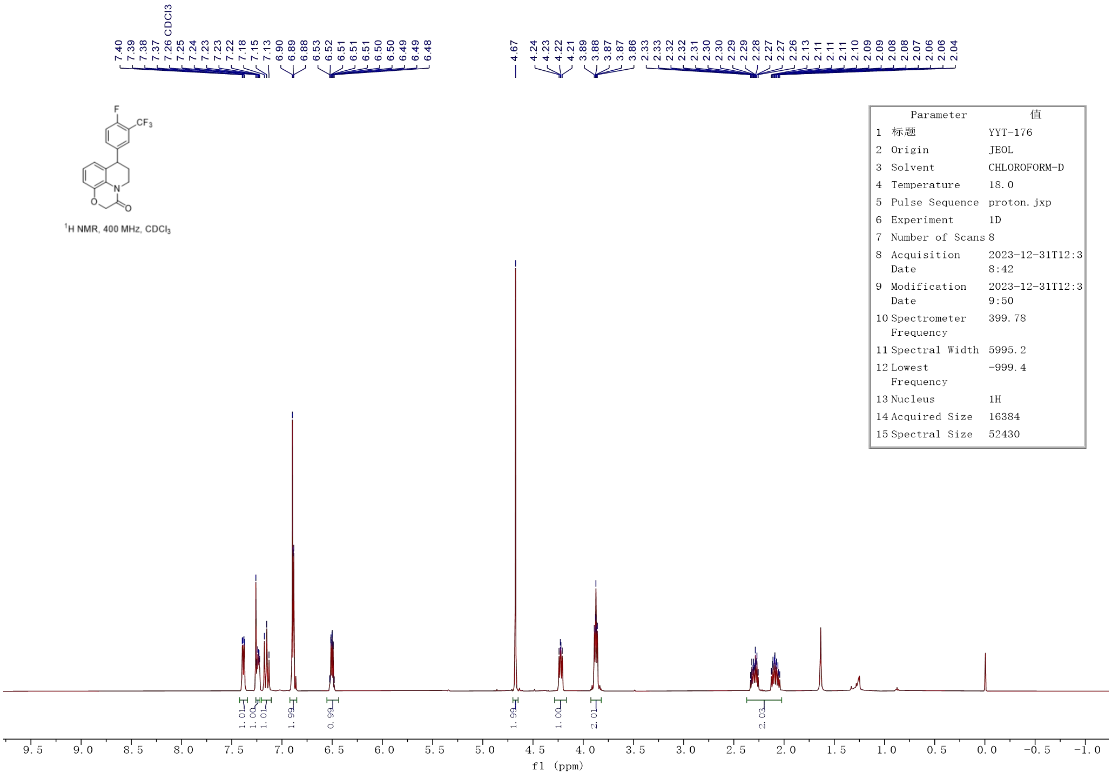

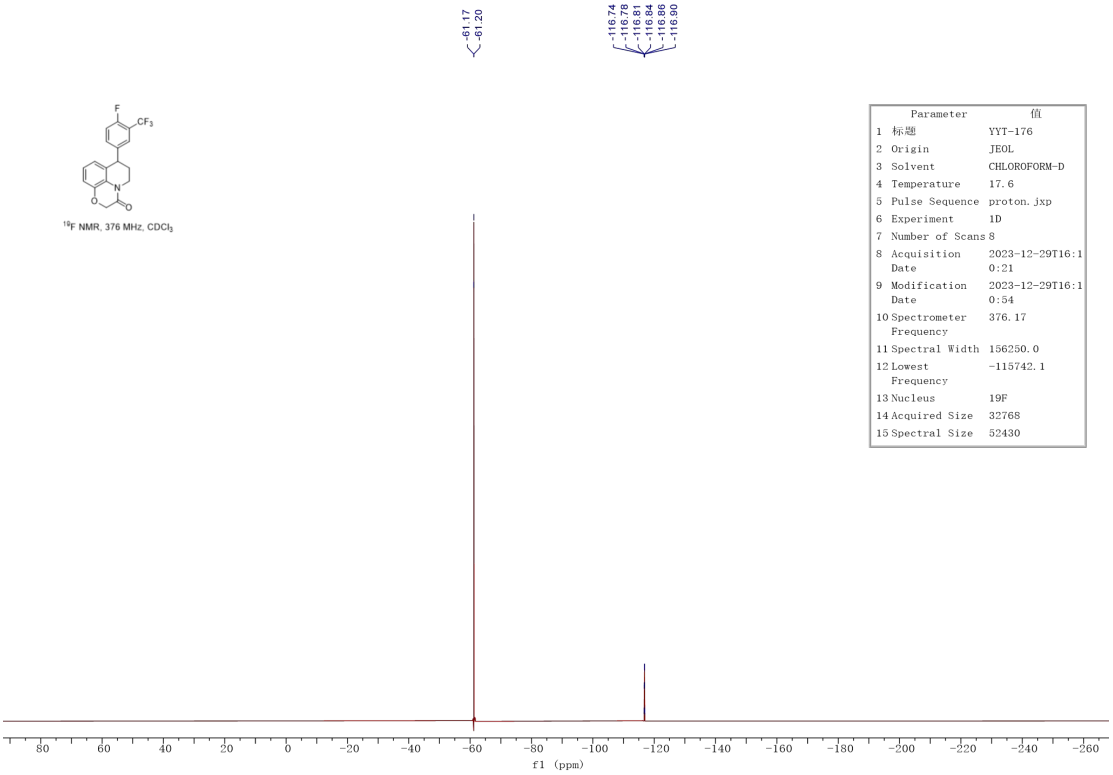

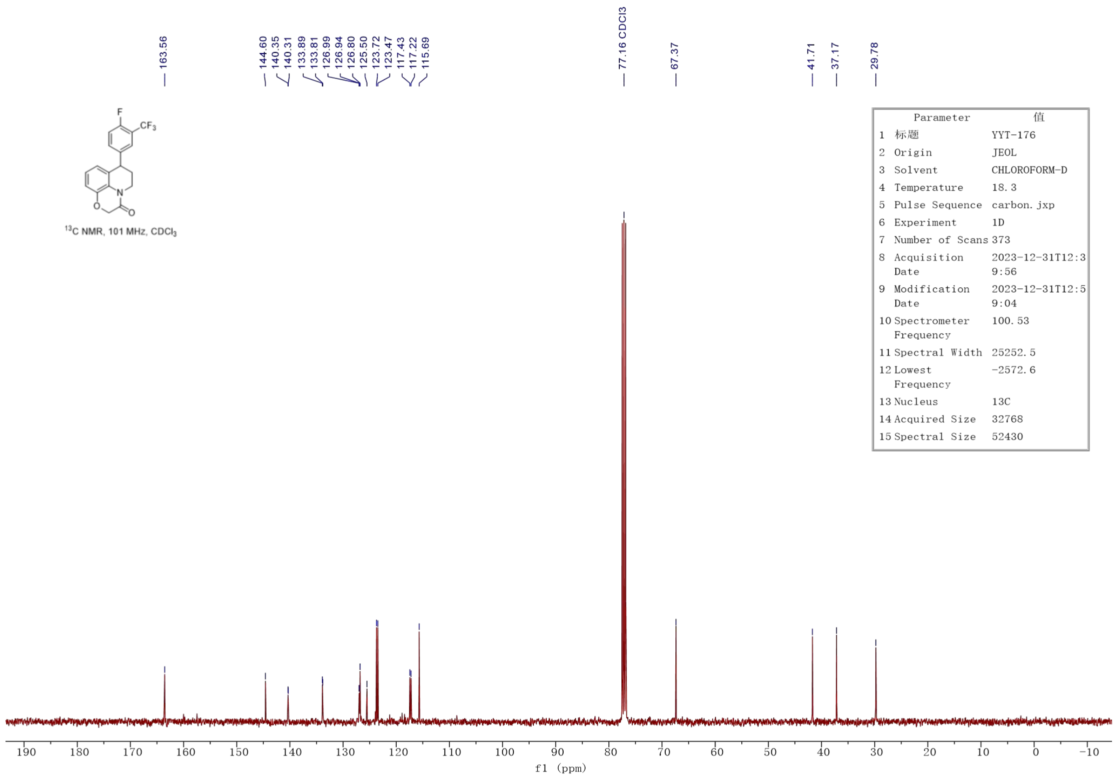

Parameter	值
1 标题	YYT-176
2 Origin	JEOL
3 Solvent	CHLOROFORM-D
4 Temperature	18.3
5 Pulse Sequence	carbon.jxp
6 Experiment	1D
7 Number of Scans	373
8 Acquisition Date	2023-12-31T12:39:56
9 Modification Date	2023-12-31T12:59:04
10 Spectrometer Frequency	100.53
11 Spectral Width	25252.5
12 Lowest Frequency	-2572.6
13 Nucleus	13C
14 Acquired Size	32768
15 Spectral Size	52430

(R)-7-(3-chloro-4-methylphenyl)-6,7-dihydro-5H-[1,4]oxazino[2,3,4-i*J*]quinolin-3 (2H)-one (35)

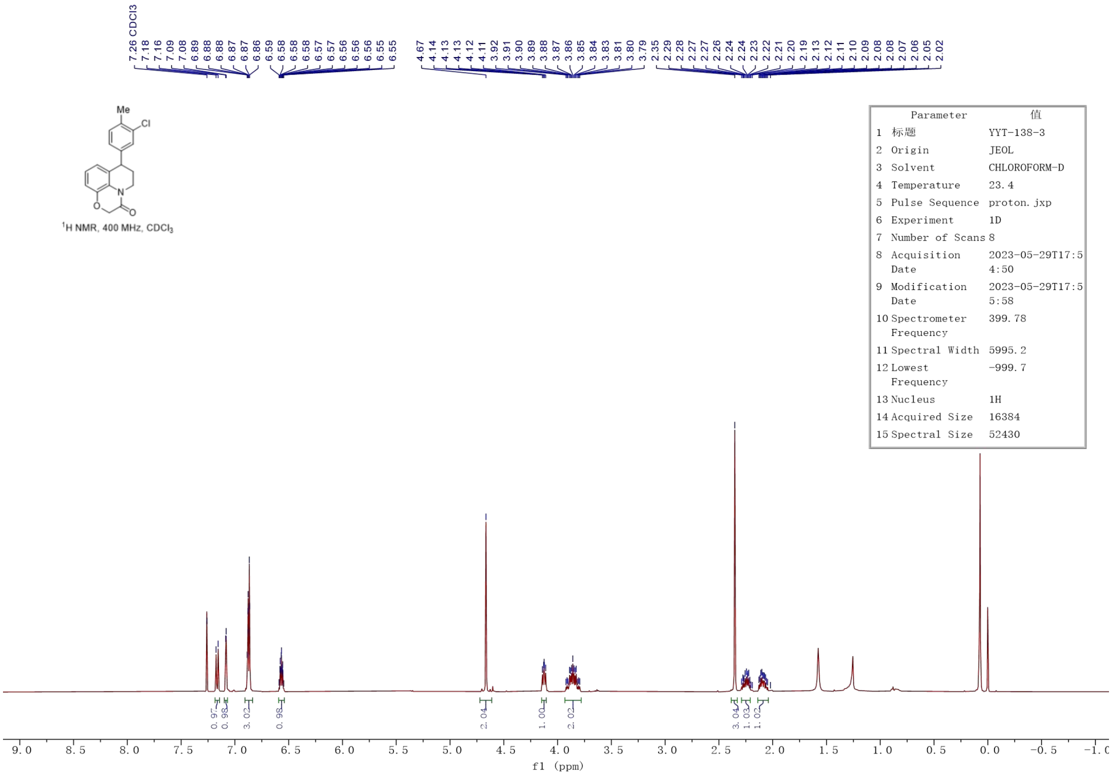

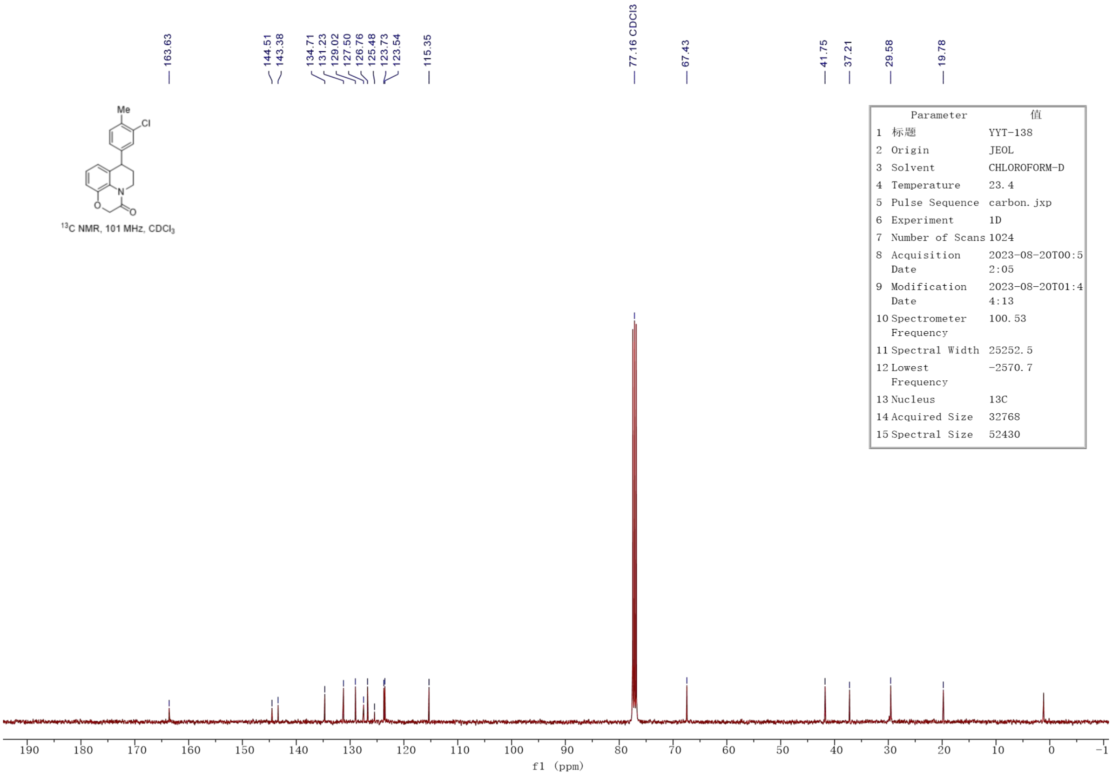

(R)-7-(2,4-dimethylphenyl)-6,7-dihydro-5H-[1,4]oxazino[2,3,4-i*J*]quinolin-3(2H)-one (36)

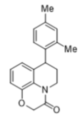

^{1}H NMR, 400 MHz, $CDCl_3$

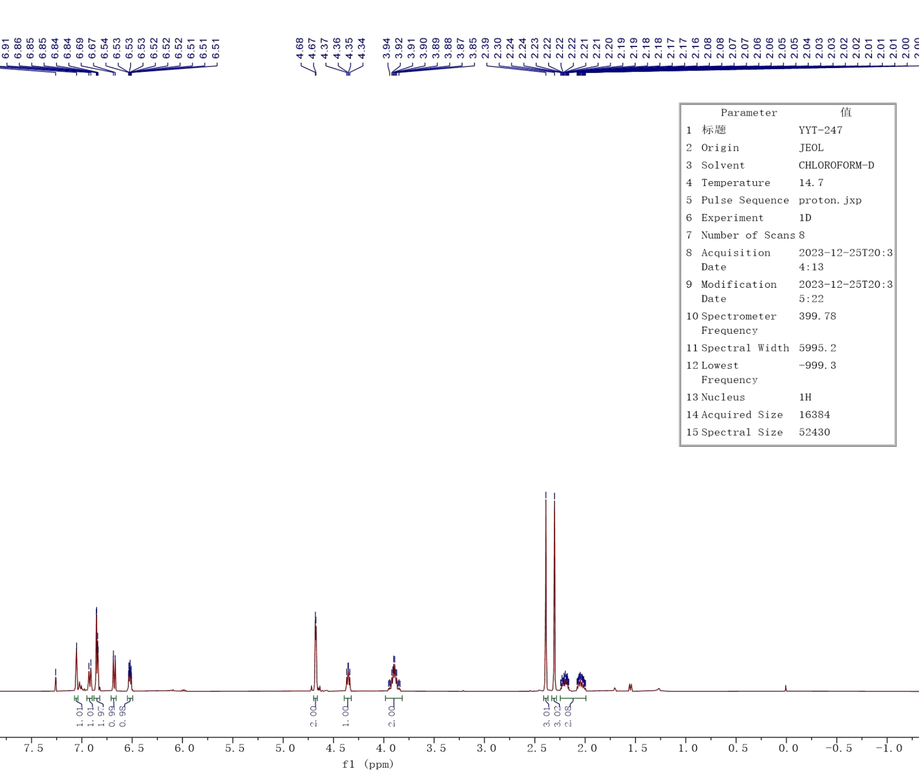

	Parameter	値
1	标题	YYT-247
2	Origin	JEOL
3	Solvent	CHLOROFORM-D
4	Temperature	14.7
5	Pulse Sequence	proton.jxp
6	Experiment	1D
7	Number of Scans	8
8	Acquisition Date	2023-12-25T20:34:13
9	Modification Date	2023-12-25T20:35:22
10	Spectrometer Frequency	399.78
11	Spectral Width	5995.2
12	Lowest Frequency	-999.3
13	Nucleus	1H
14	Acquired Size	16384
15	Spectral Size	52430

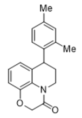

^{13}C NMR, 101 MHz, $CDCl_3$

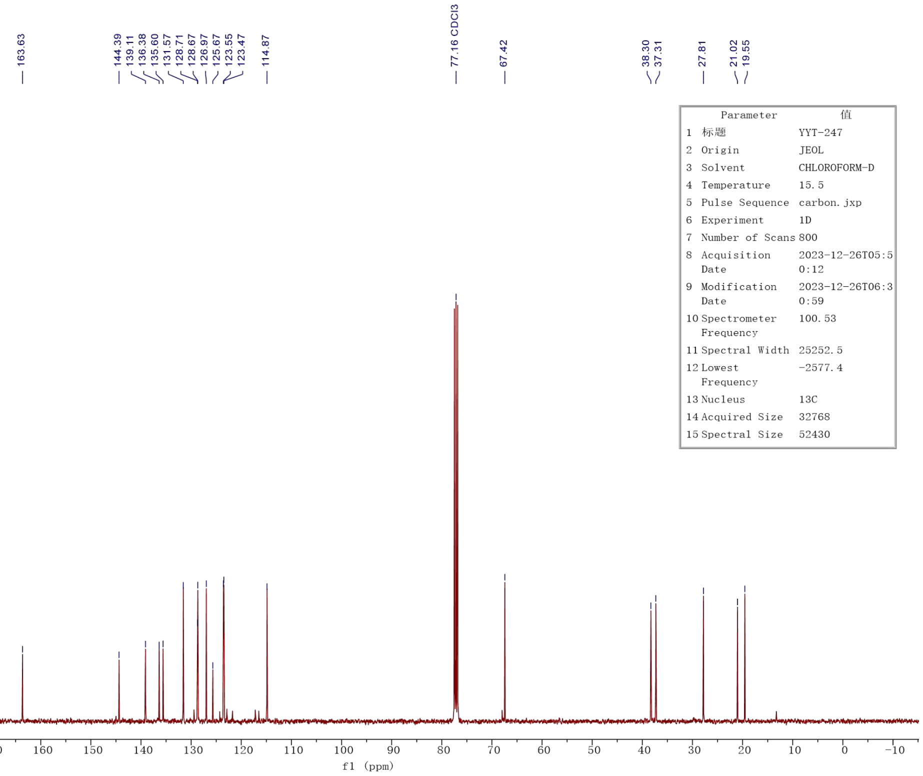

	Parameter	値
1	标题	YYT-247
2	Origin	JEOL
3	Solvent	CHLOROFORM-D
4	Temperature	15.5
5	Pulse Sequence	carbon.jxp
6	Experiment	1D
7	Number of Scans	800
8	Acquisition Date	2023-12-26T05:50:12
9	Modification Date	2023-12-26T06:30:59
10	Spectrometer Frequency	100.53
11	Spectral Width	25252.5
12	Lowest Frequency	-2577.4
13	Nucleus	13C
14	Acquired Size	32768
15	Spectral Size	52430

(R)-7-(5,6,7,8-tetrahydronaphthalen-2-yl)-6,7-dihydro-5H-[1,4]oxazino[2,3,4-i*J*]quinolin-3(2H)-one (37)

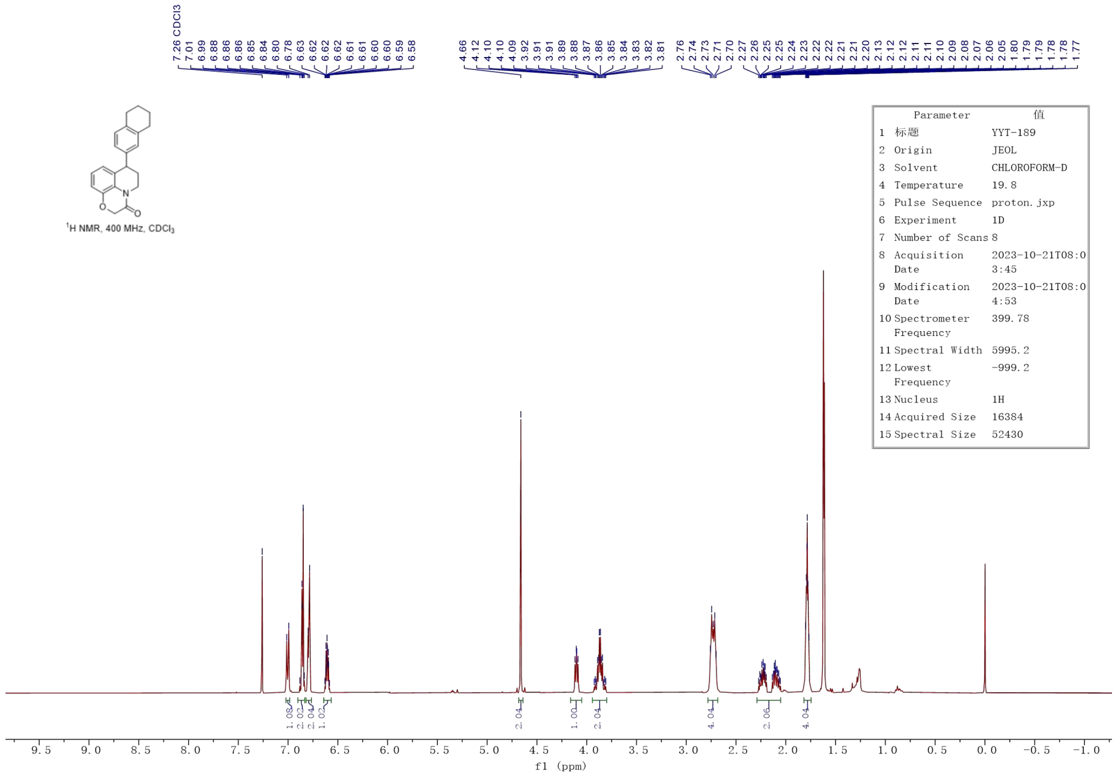

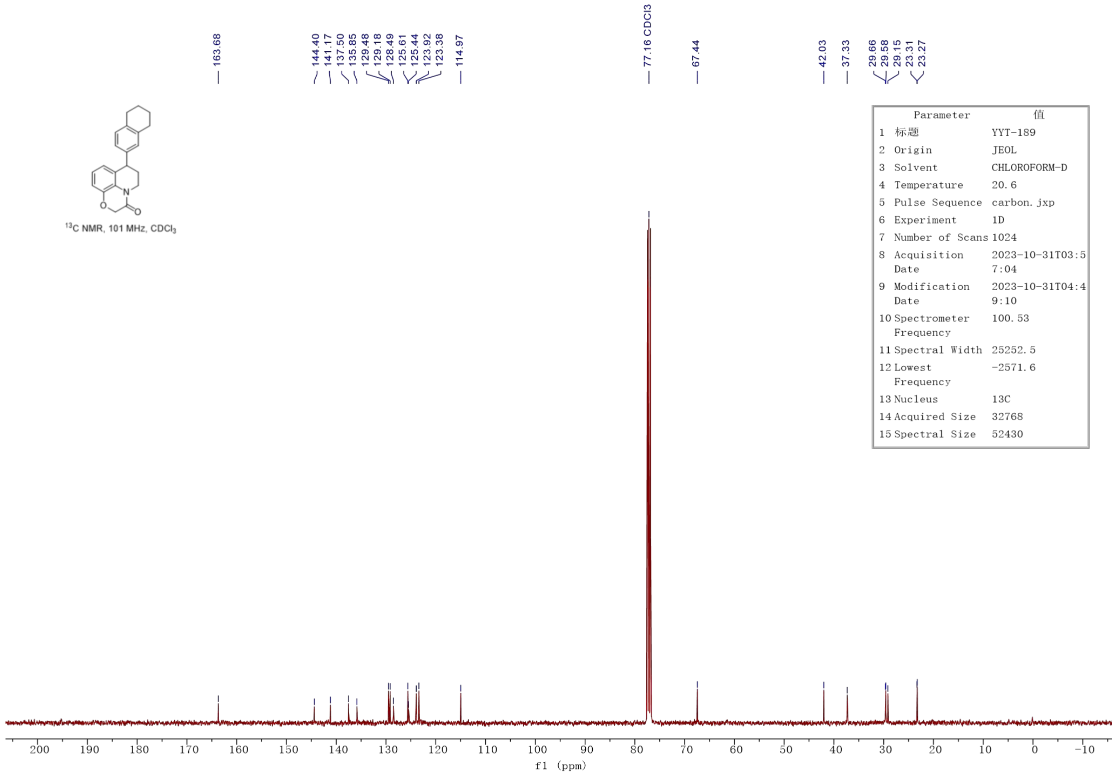

(S)-7-(6-chloropyridin-3-yl)-6,7-dihydro-5H-[1,4]oxazino[2,3,4-i*J*]quinolin-3(2H) -one (38)

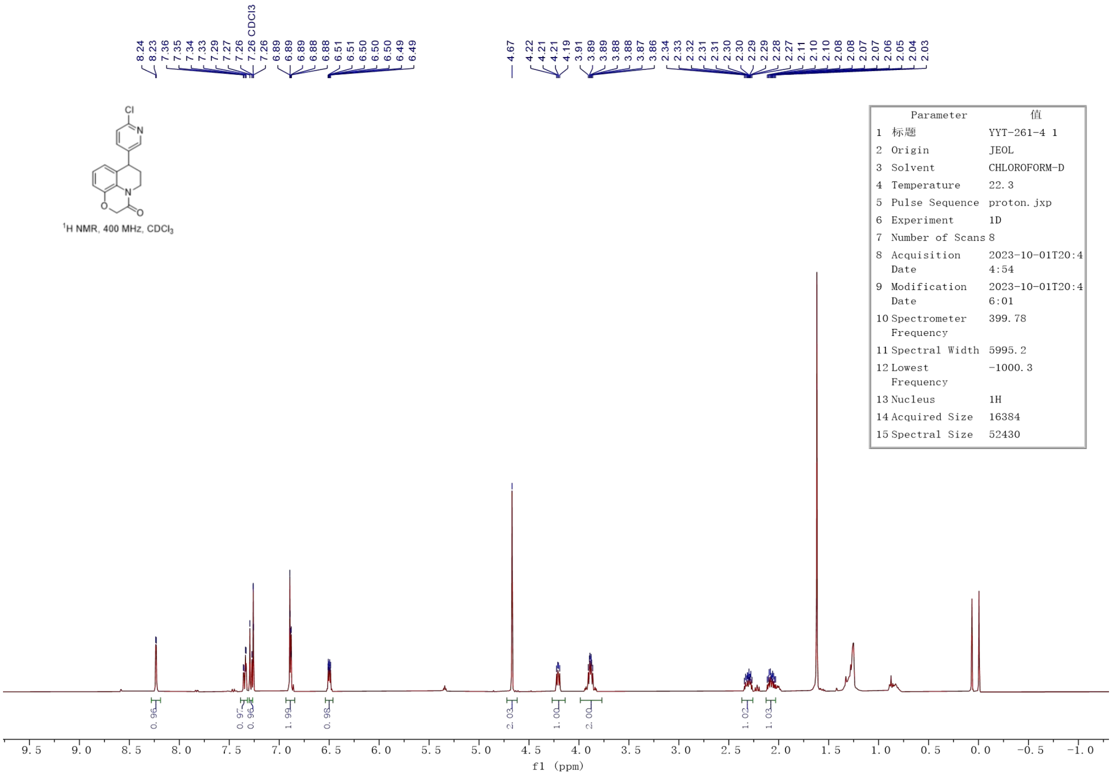

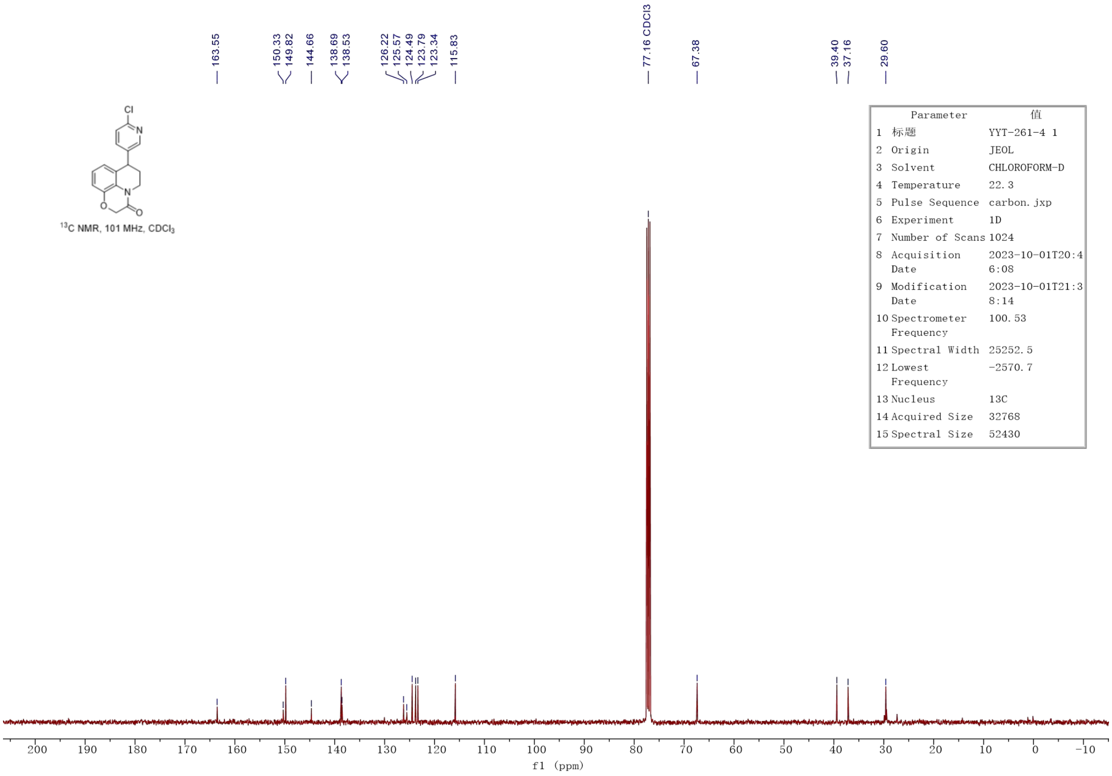

(S)-7-(3,5-dimethylisoxazol-4-yl)-6,7-dihydro-5H-[1,4]oxazino[2,3,4-i*J*]quinolin-3 (2H)-one (39)

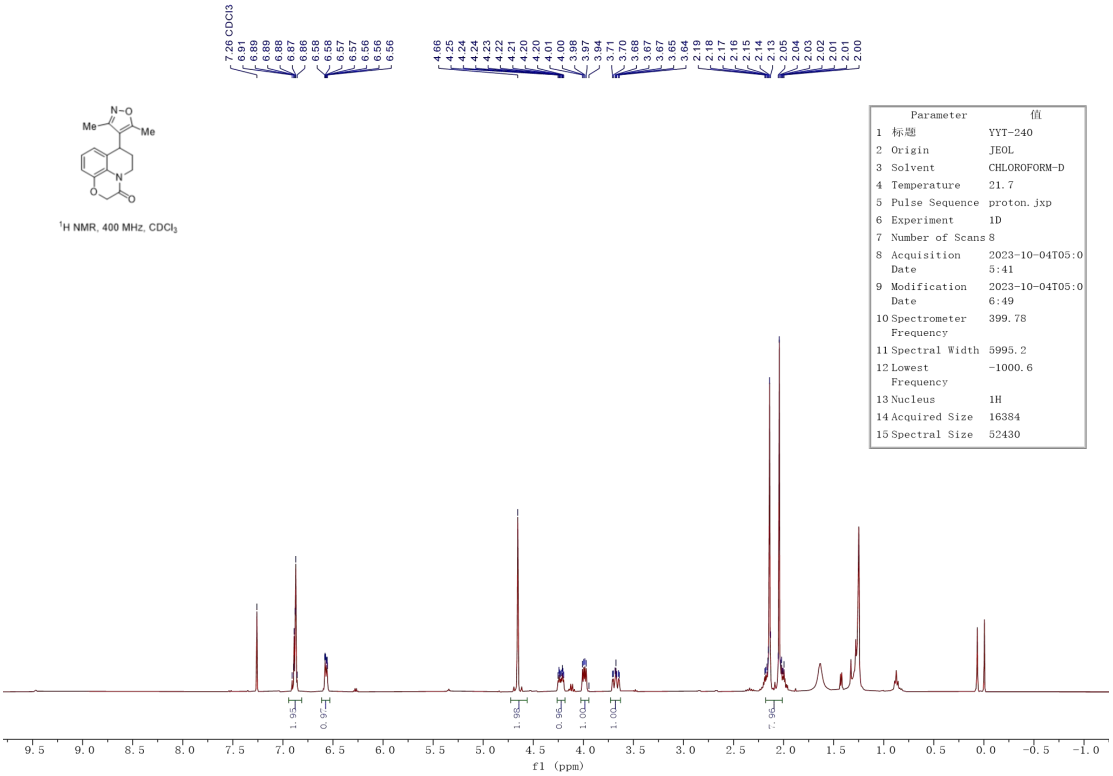

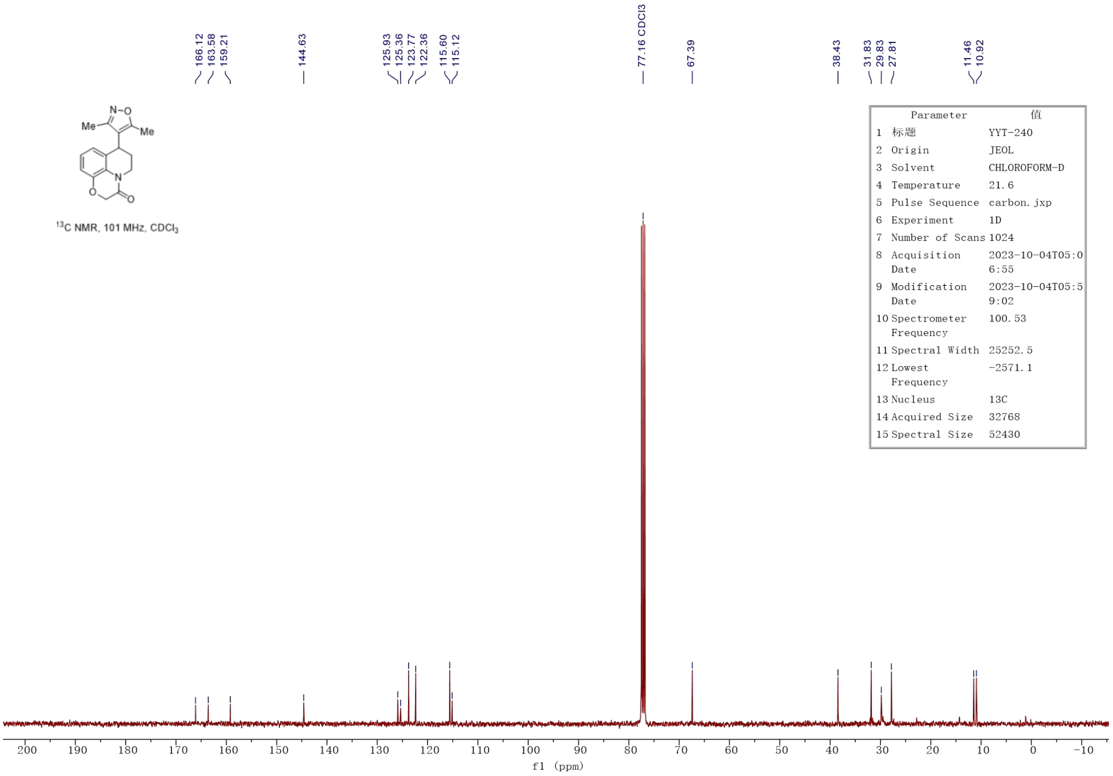

(S)-7-(3-bromo-4-chlorothiophen-2-yl)-6,7-dihydro-5H-[1,4]oxazino[2,3,4-i*J*]quinolin-3(2H)-one (40)

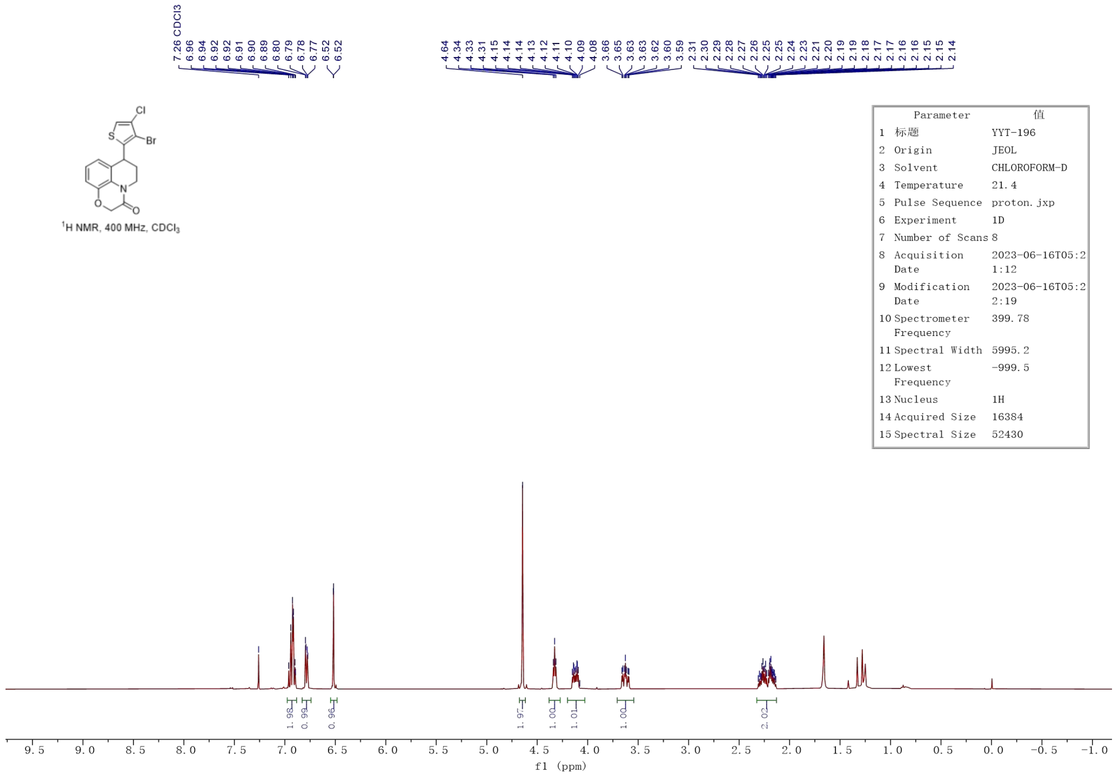

^{1}H NMR, 400 MHz, $CDCl_3$

	Parameter	值
1	标题	YYT-196
2	Origin	JEOL
3	Solvent	CHLOROFORM-D
4	Temperature	21.4
5	Pulse Sequence	proton.jxp
6	Experiment	1D
7	Number of Scans	8
8	Acquisition Date	2023-06-16T05:21:12
9	Modification Date	2023-06-16T05:22:19
10	Spectrometer Frequency	399.78
11	Spectral Width	5995.2
12	Lowest Frequency	-999.5
13	Nucleus	1H
14	Acquired Size	16384
15	Spectral Size	52430

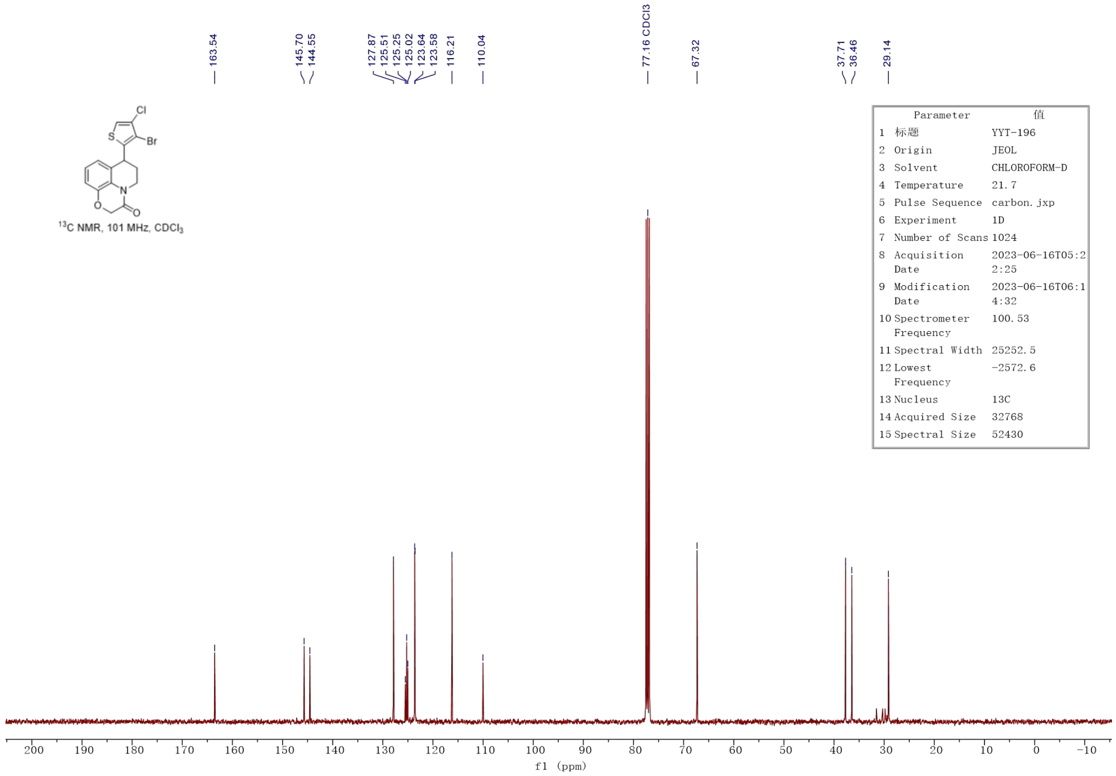

^{13}C NMR, 101 MHz, $CDCl_3$

	Parameter	值
1	标题	YYT-196
2	Origin	JEOL
3	Solvent	CHLOROFORM-D
4	Temperature	21.7
5	Pulse Sequence	carbon.jxp
6	Experiment	1D
7	Number of Scans	1024
8	Acquisition Date	2023-06-16T05:22:25
9	Modification Date	2023-06-16T06:14:32
10	Spectrometer Frequency	100.53
11	Spectral Width	25252.5
12	Lowest Frequency	-2572.6
13	Nucleus	13C
14	Acquired Size	32768
15	Spectral Size	52430

(S)-7-(5-chlorothiophen-2-yl)-6,7-dihydro-5H-[1,4]oxazino[2,3,4-i*J*]quinolin-3(2H)-one (41)

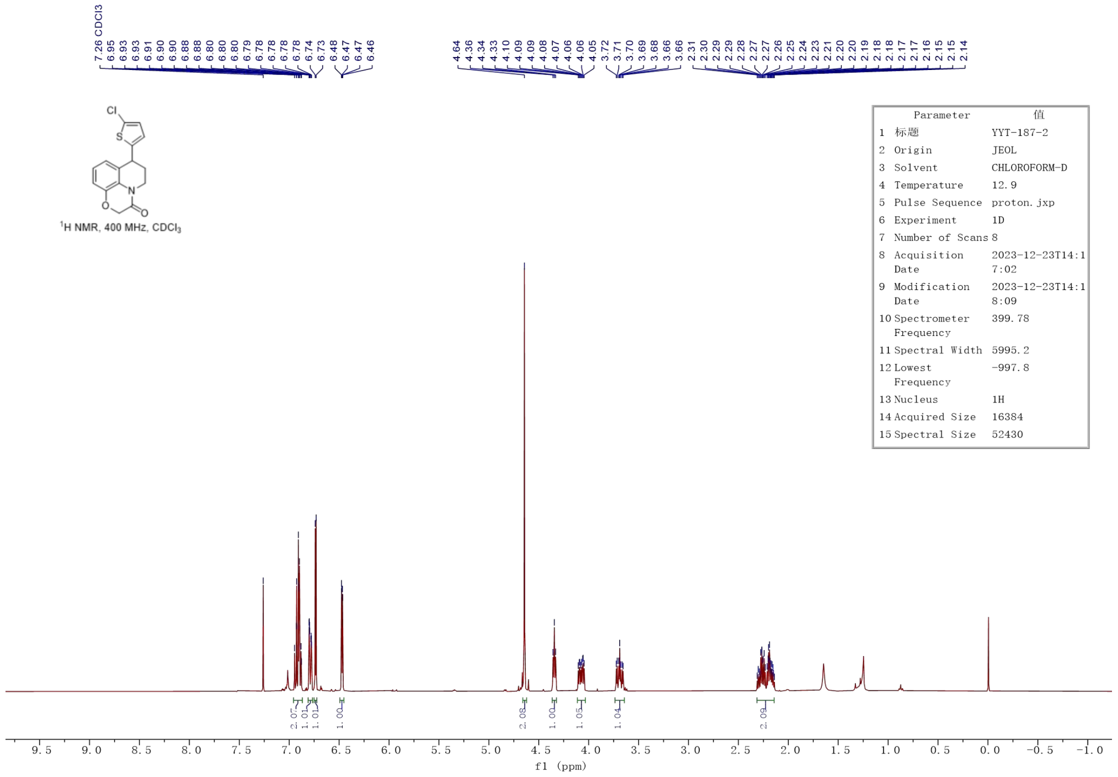

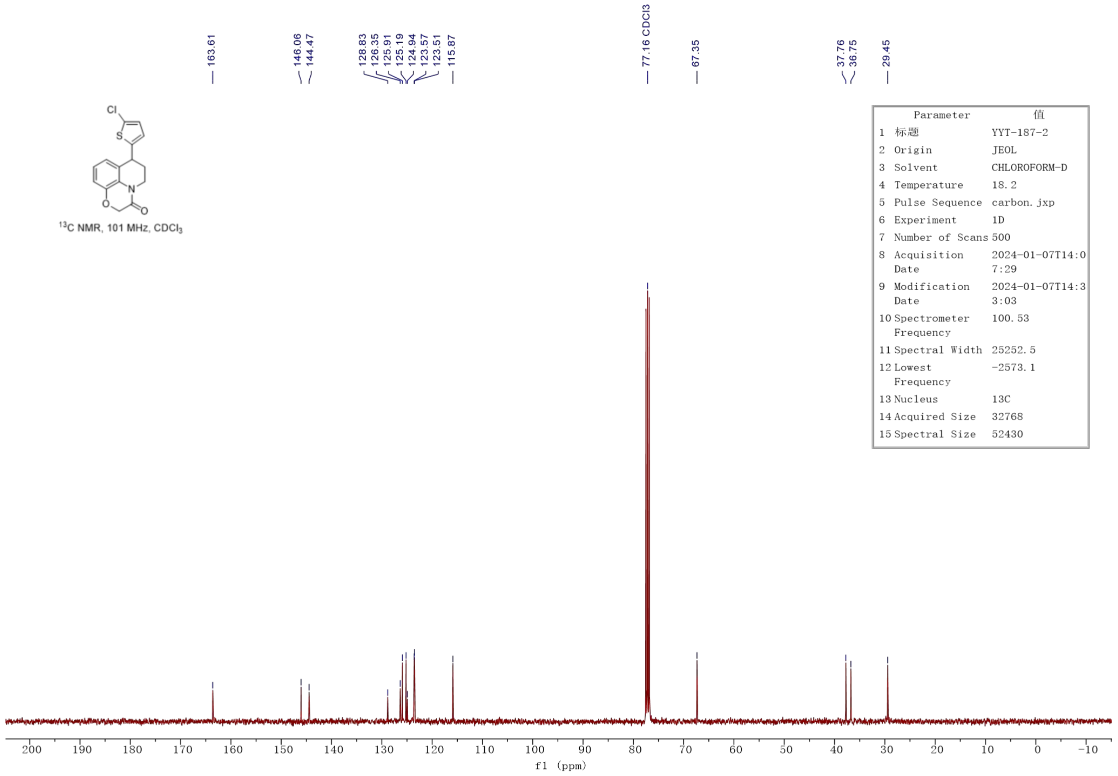

(S)-7-(benzo[b]thiophen-3-yl)-6,7-dihydro-5H-[1,4]oxazino[2,3,4-i*J*]quinolin-3(2H)-one (42)

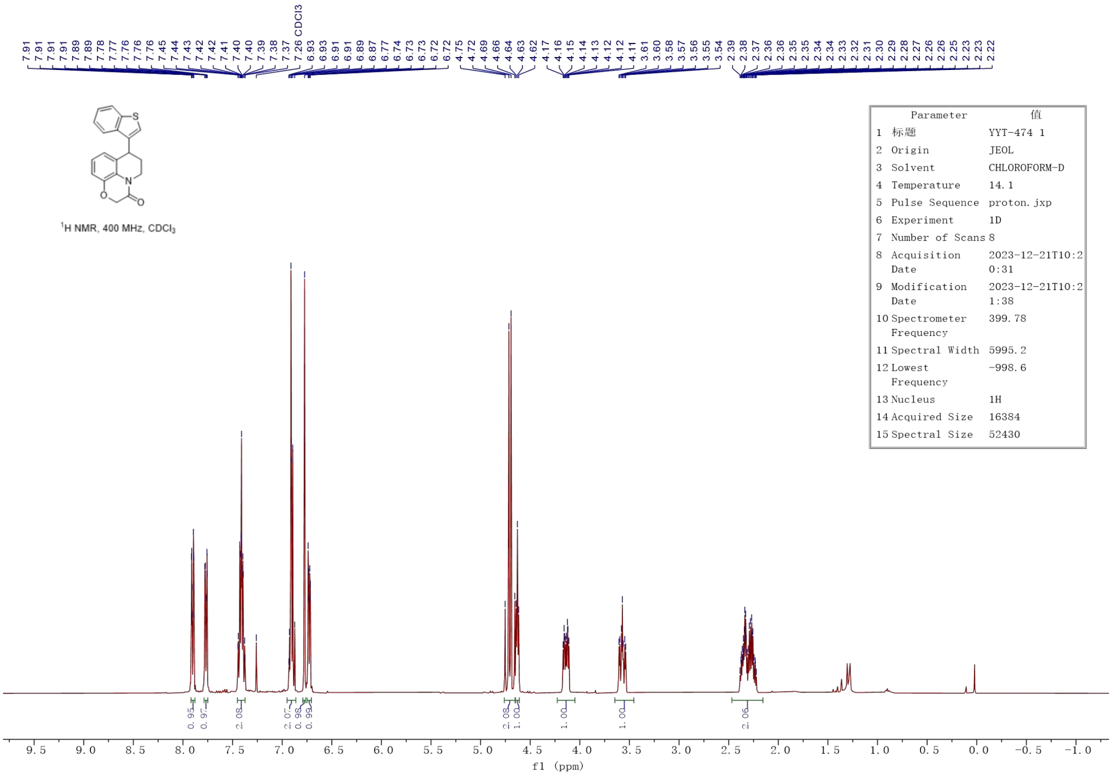

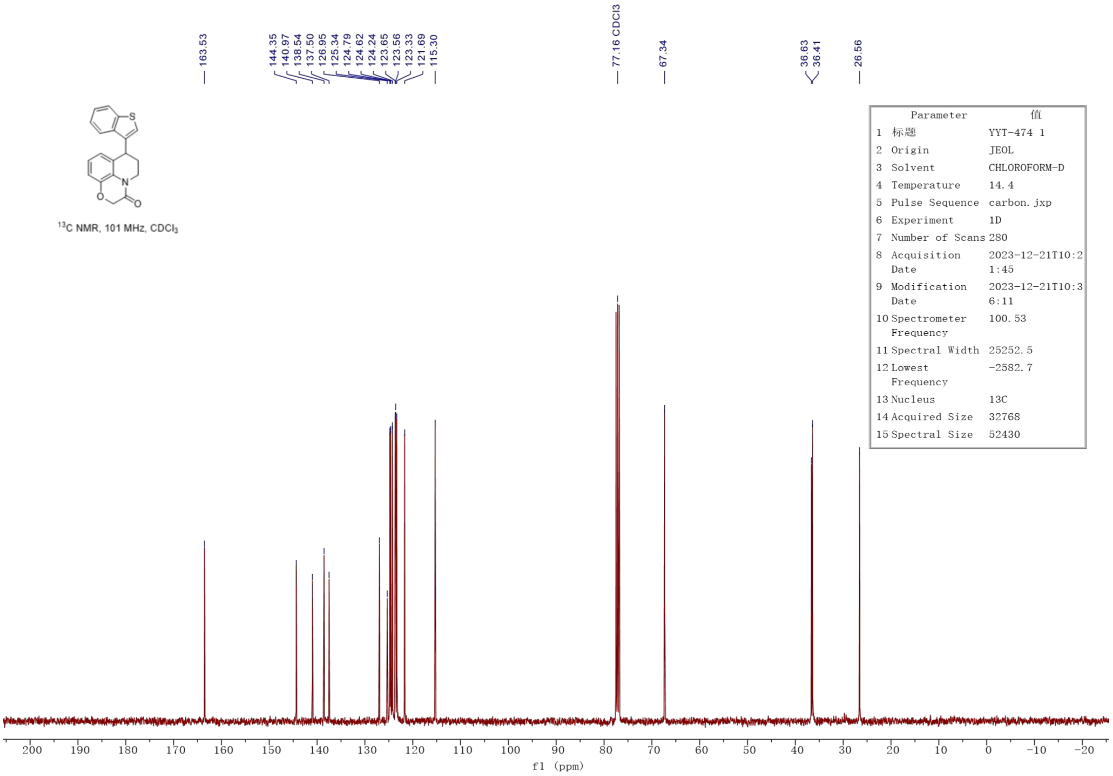

(R)-7-(2-oxo-2H-chromen-6-yl)-6,7-dihydro-5H-[1,4]oxazino[2,3,4-i*J*]quinolin-3(2H)-one (43)

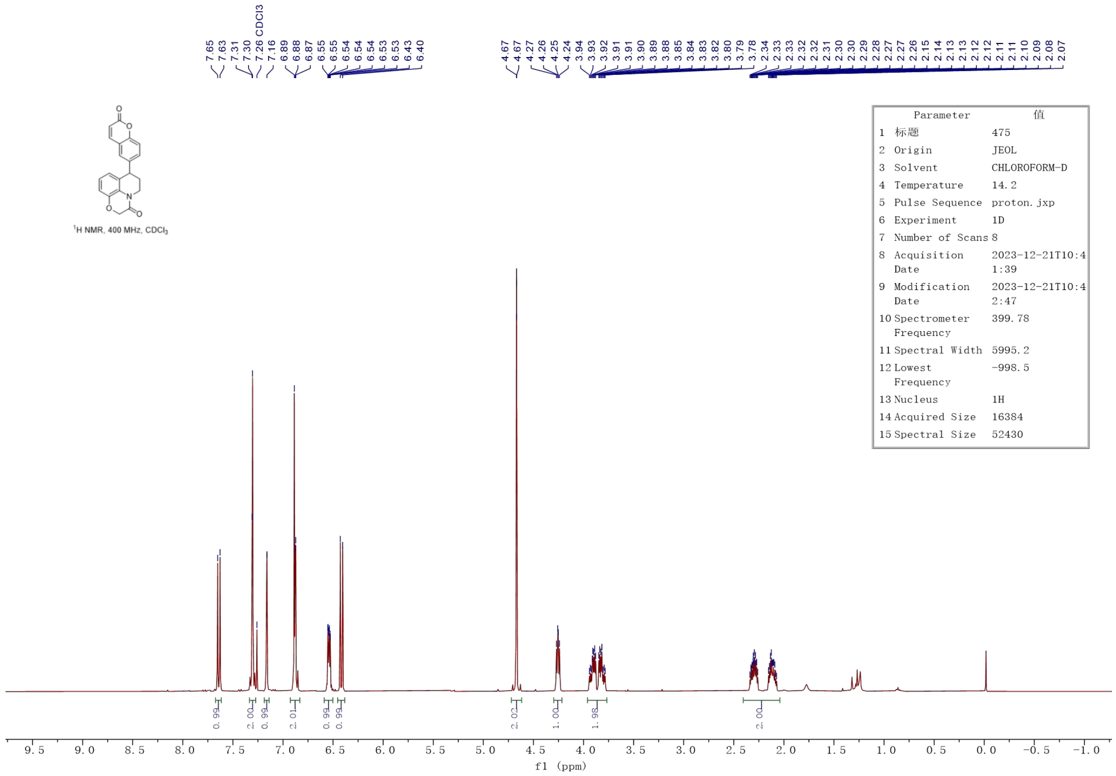

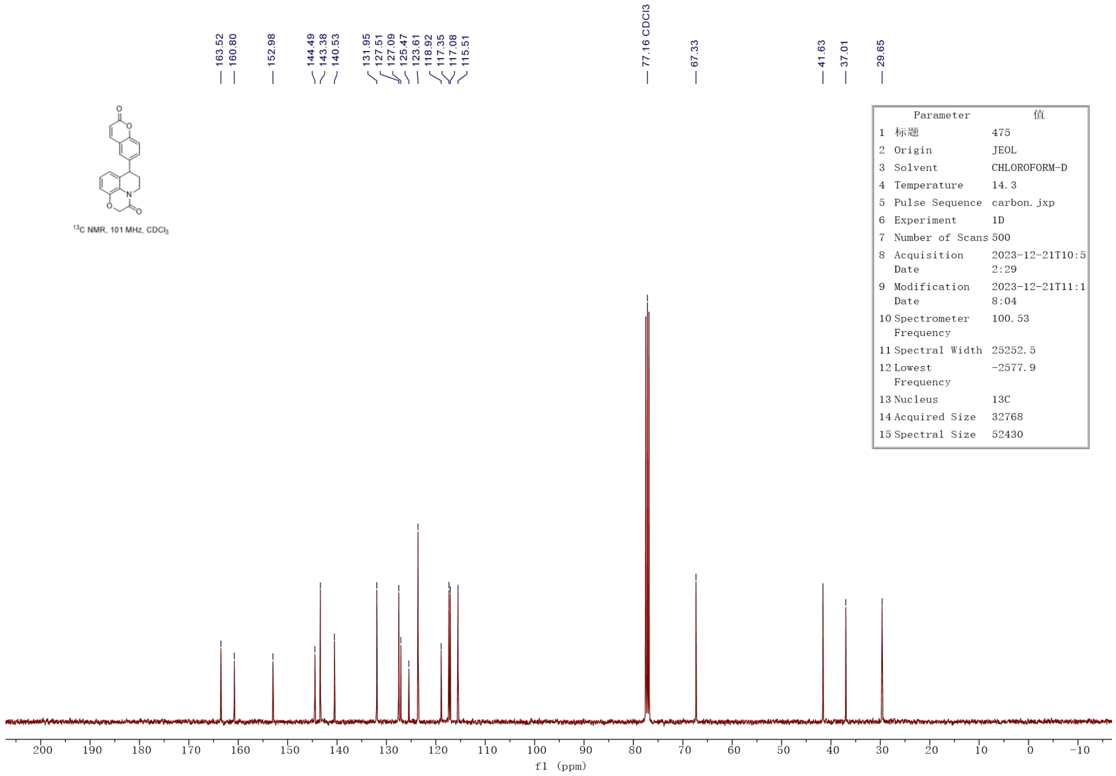

(E)-7-styryl-6,7-dihydro-5H-[1,4]oxazino[2,3,4-i*J*]quinolin-3(2H)-one (44)

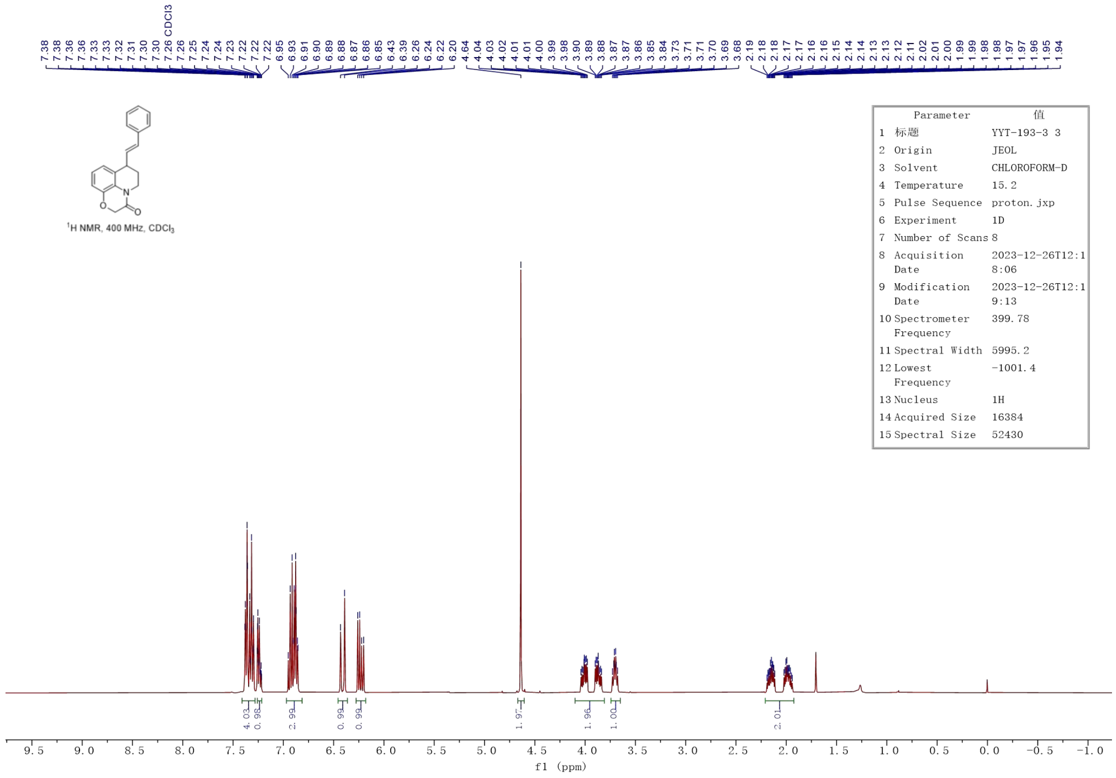

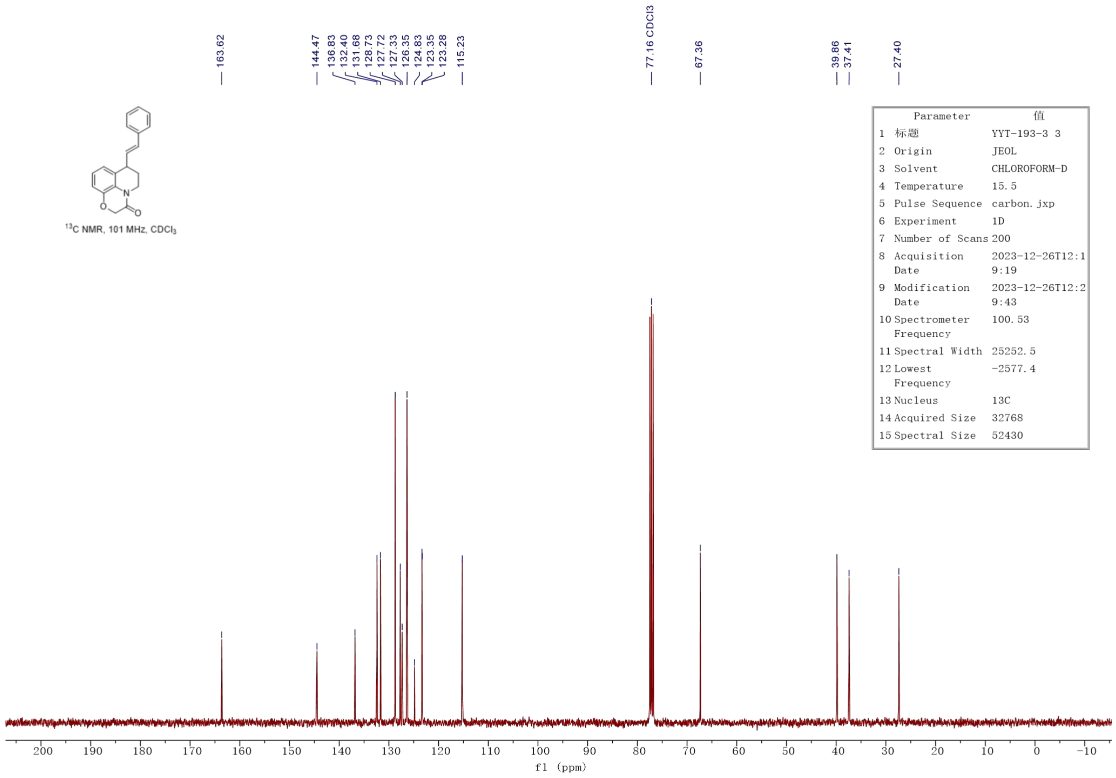

9-methoxy-7-(p-tolyl)-6,7-dihydro-5H-[1,4]oxazino[2,3,4-iJ]quinolin-3(2H)-one (45)

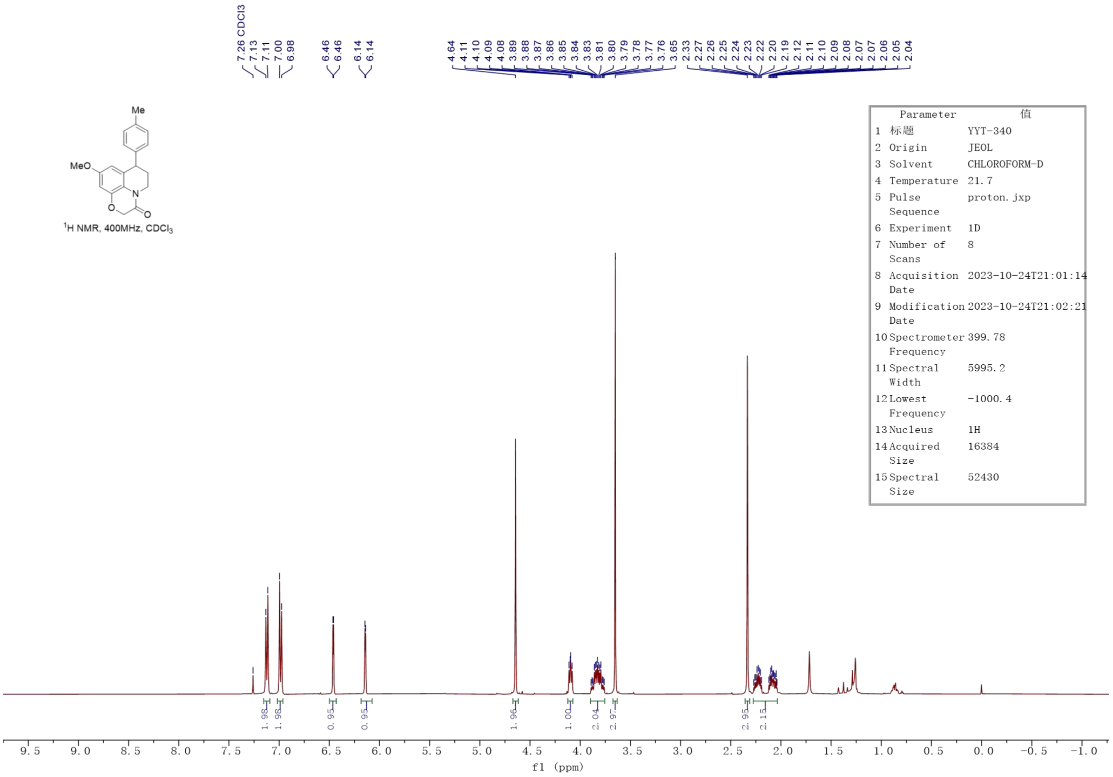

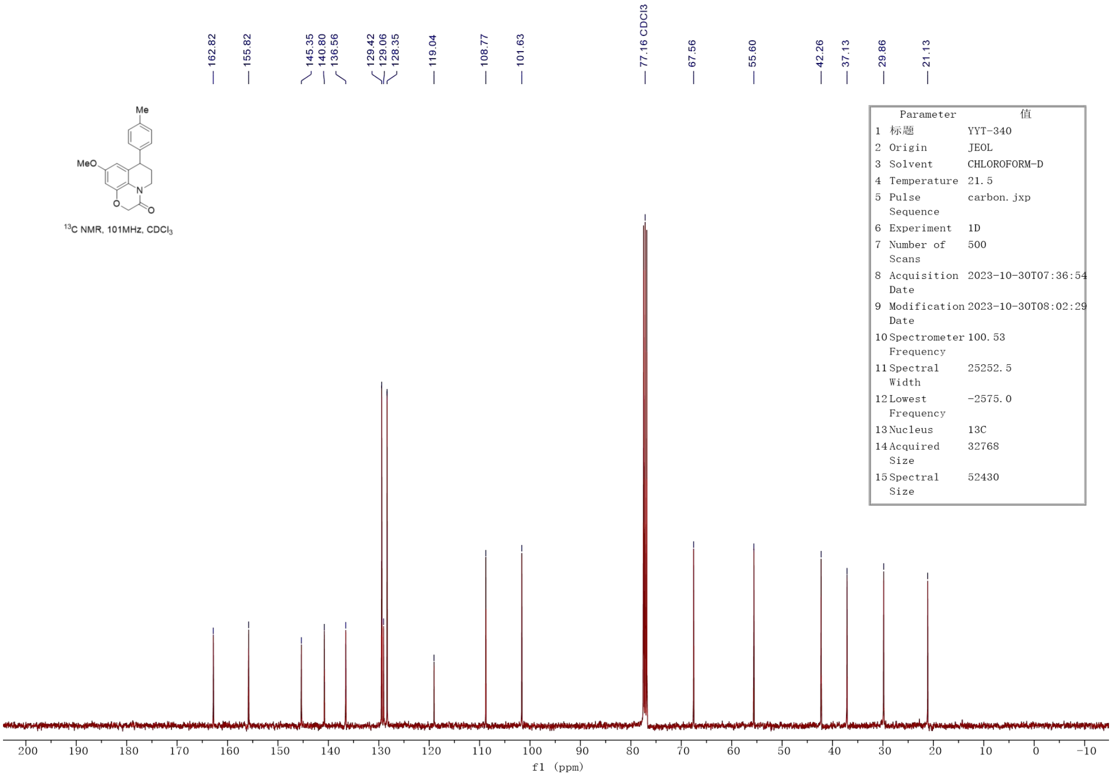

8-methyl-7-(p-tolyl)-6,7-dihydro-5H-[1,4]oxazino[2,3,4-iJ]quinolin-3(2H)-one (46)

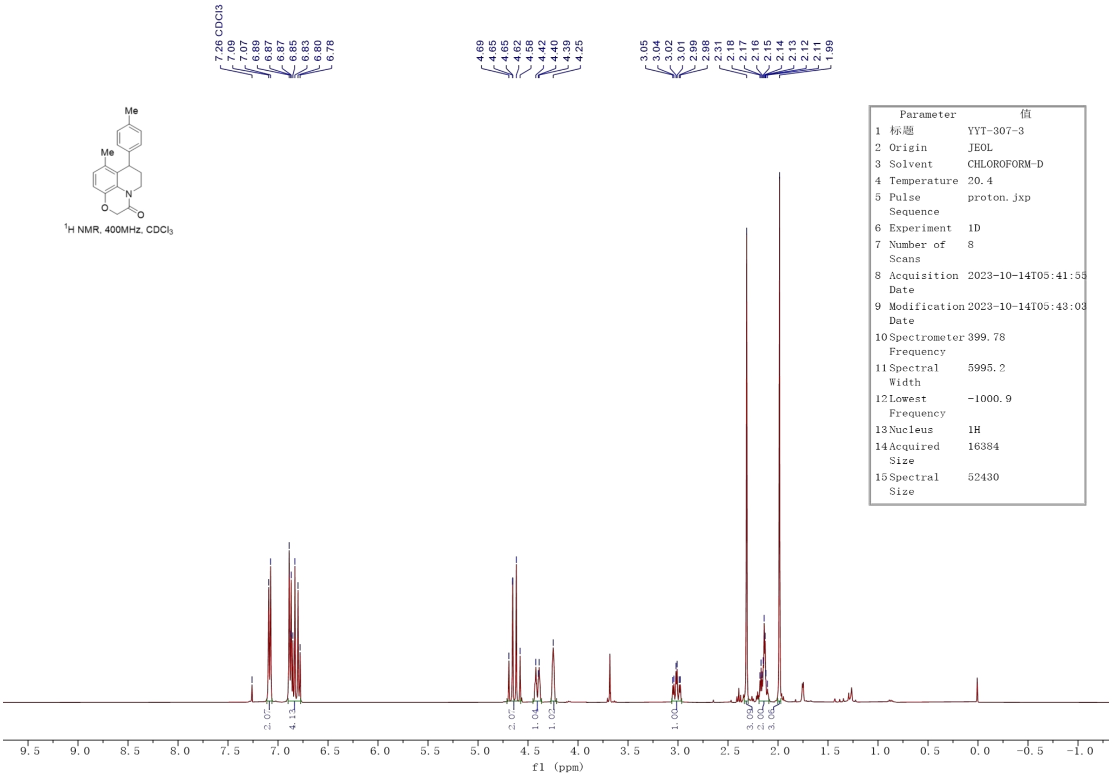

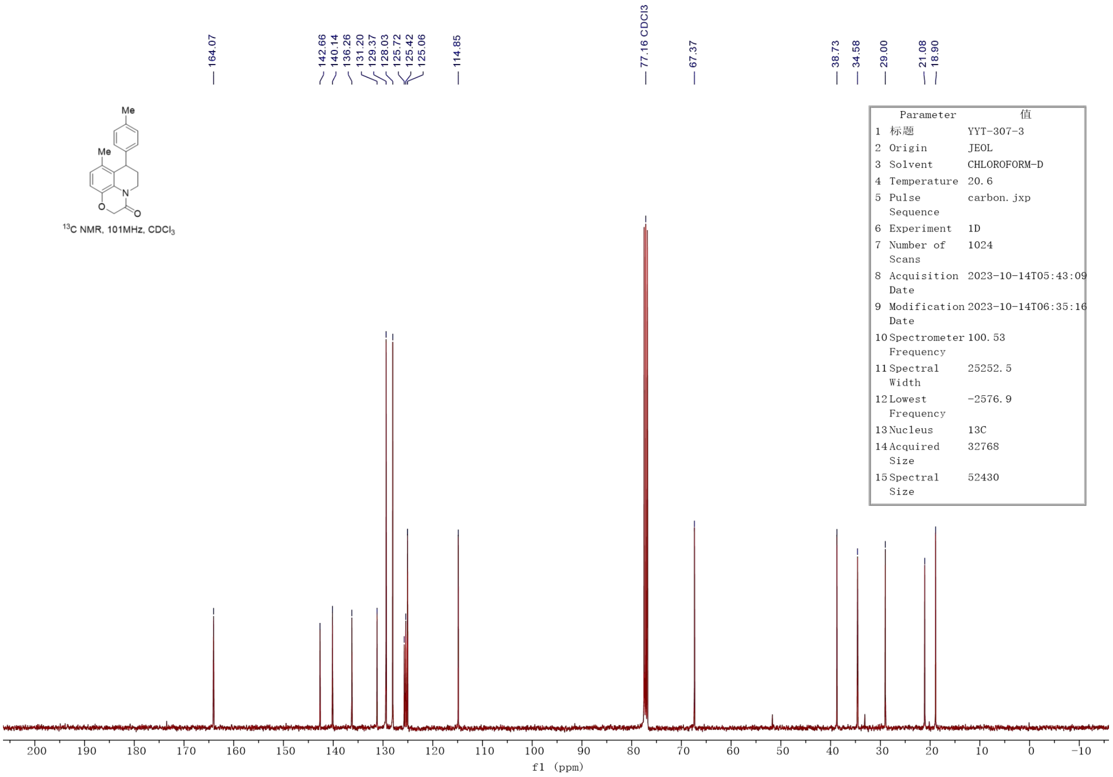

9-fluoro-7-(p-tolyl)-6,7-dihydro-5H-[1,4]oxazino[2,3,4-iJ]quinolin-3(2H)-one (47)

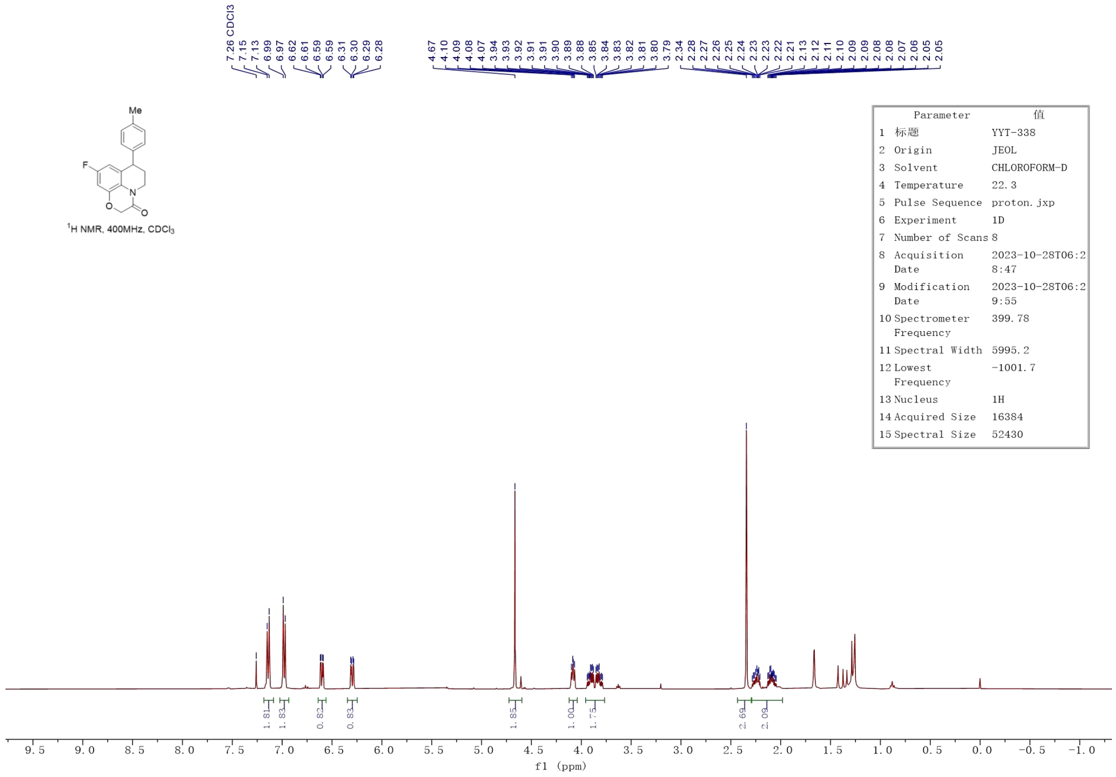

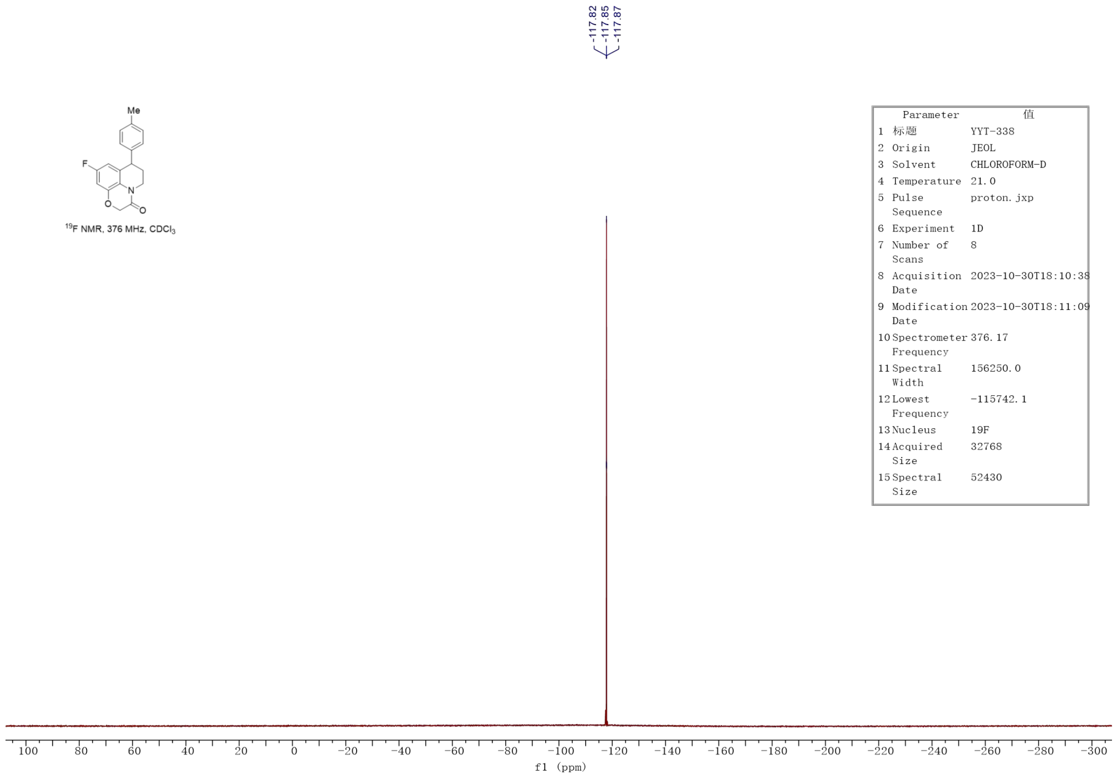

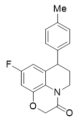

^{13}C NMR, 101MHz, $CDCl_3$

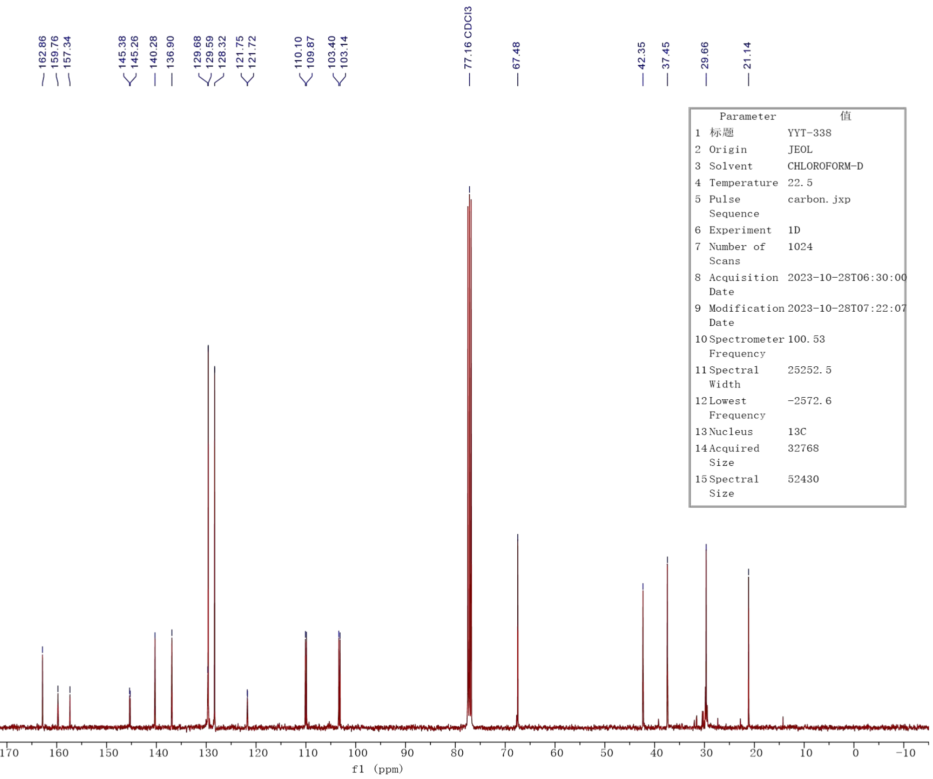

	Parameter	值
1	标题	YYT-338
2	Origin	JEOL
3	Solvent	CHLOROFORM-D
4	Temperature	22.5
5	Pulse Sequence	carbon.jxp
6	Experiment	1D
7	Number of Scans	1024
8	Acquisition Date	2023-10-28T06:30:00
9	Modification Date	2023-10-28T07:22:07
10	Spectrometer Frequency	100.53
11	Spectral Width	25252.5
12	Lowest Frequency	-2572.6
13	Nucleus	13C
14	Acquired Size	32768
15	Spectral Size	52430

1-(p-tolyl)-2,3,6,7-tetrahydro-1H,5H-pyrido[3,2,1-iJ]quinolin-5-one (48)

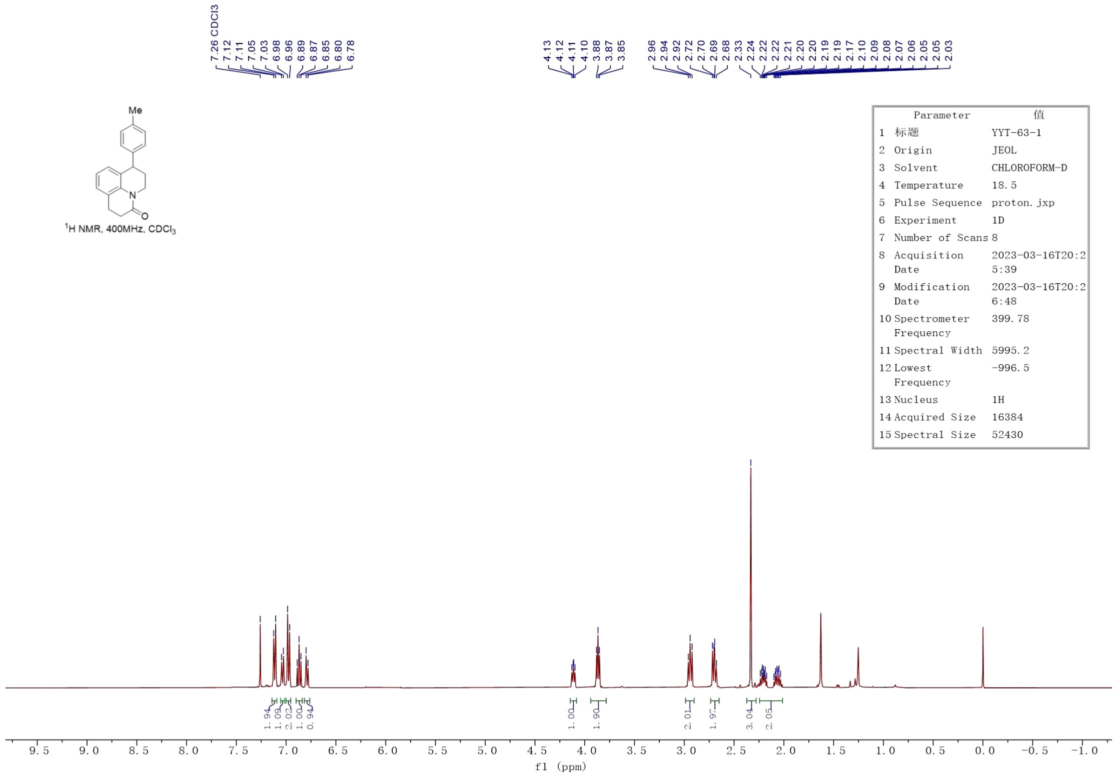

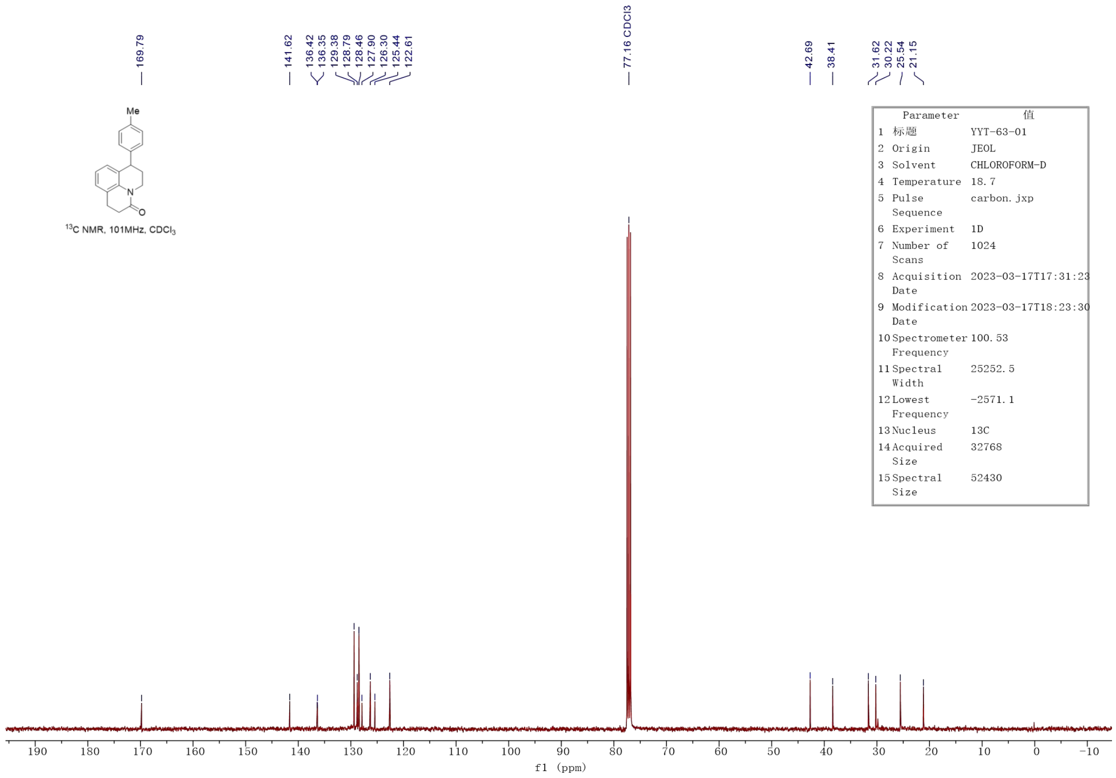

9-methoxy-1-(p-tolyl)-2,3,6,7-tetrahydro-1H,5H-pyrido[3,2,1-iJ]quinolin-5-one (49)

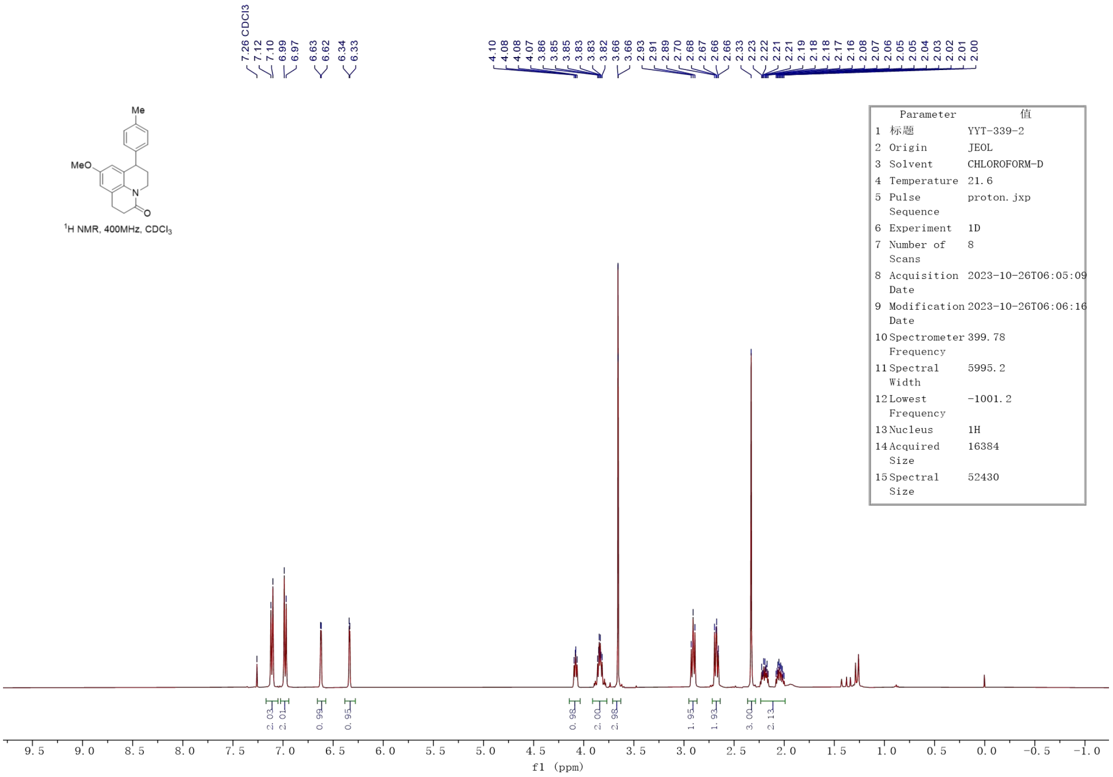

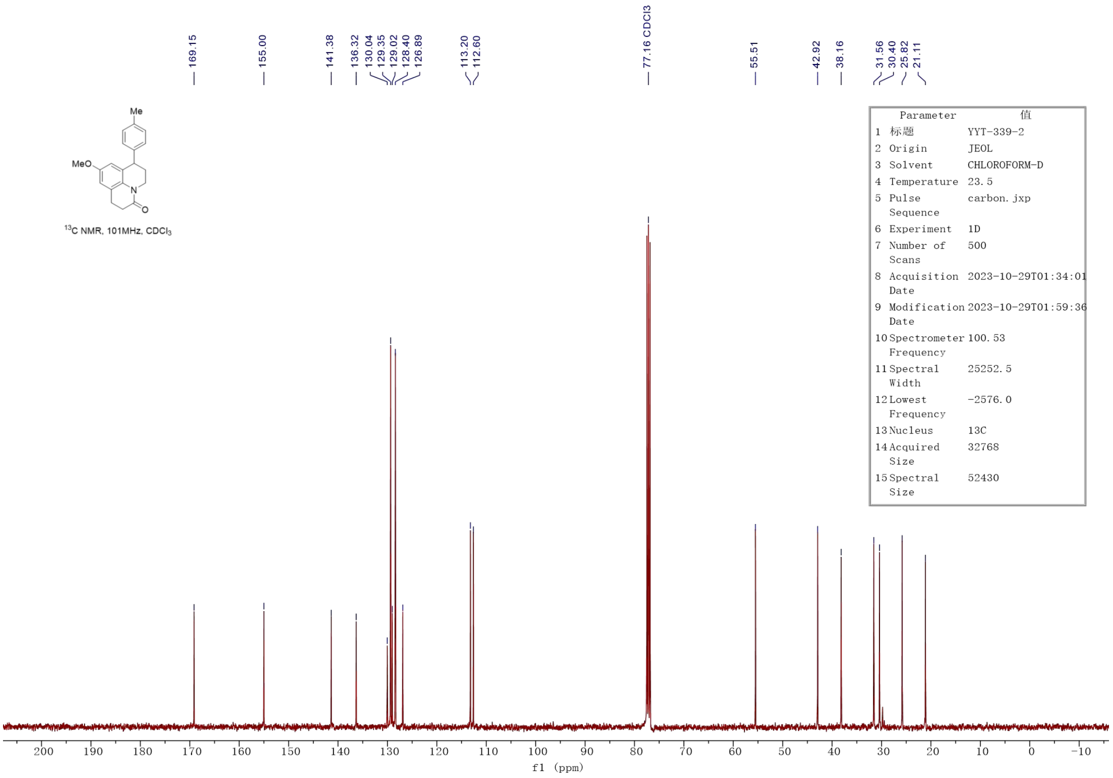

9-((tert-butyldimethylsilyl)oxy)-1-(p-tolyl)-2,3,6,7-tetrahydro-1H,5H-pyrido[3,2,1-ij]quinolin-5-one (50)

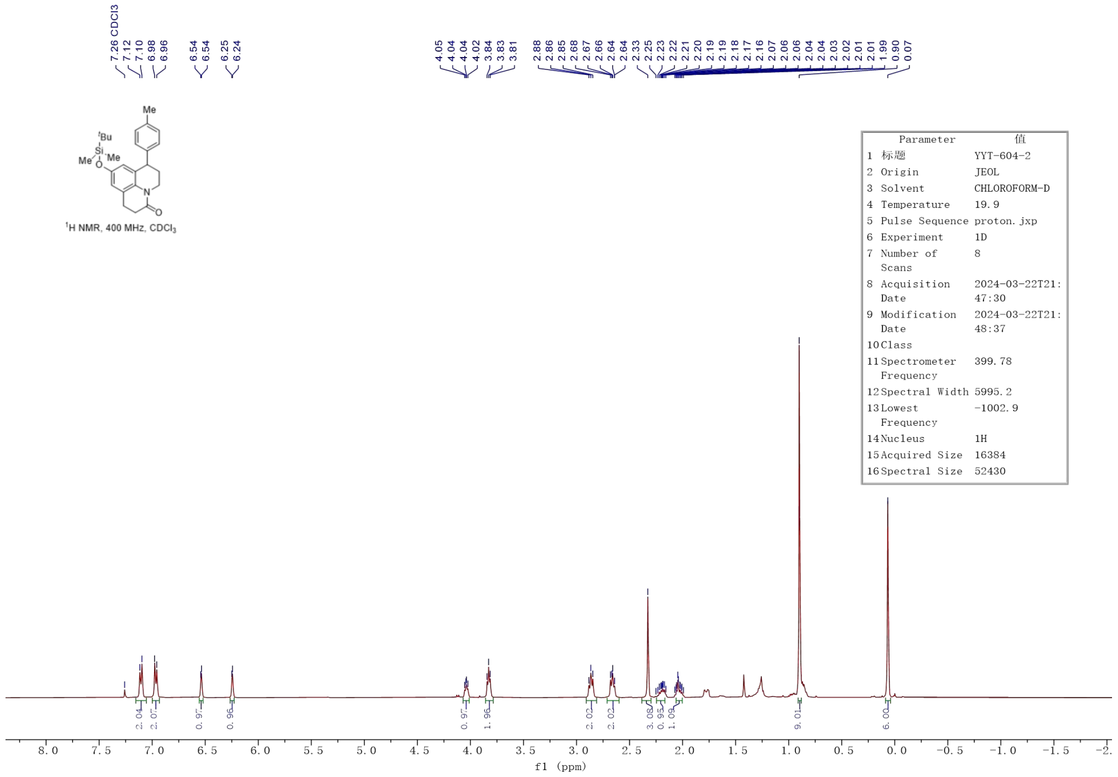

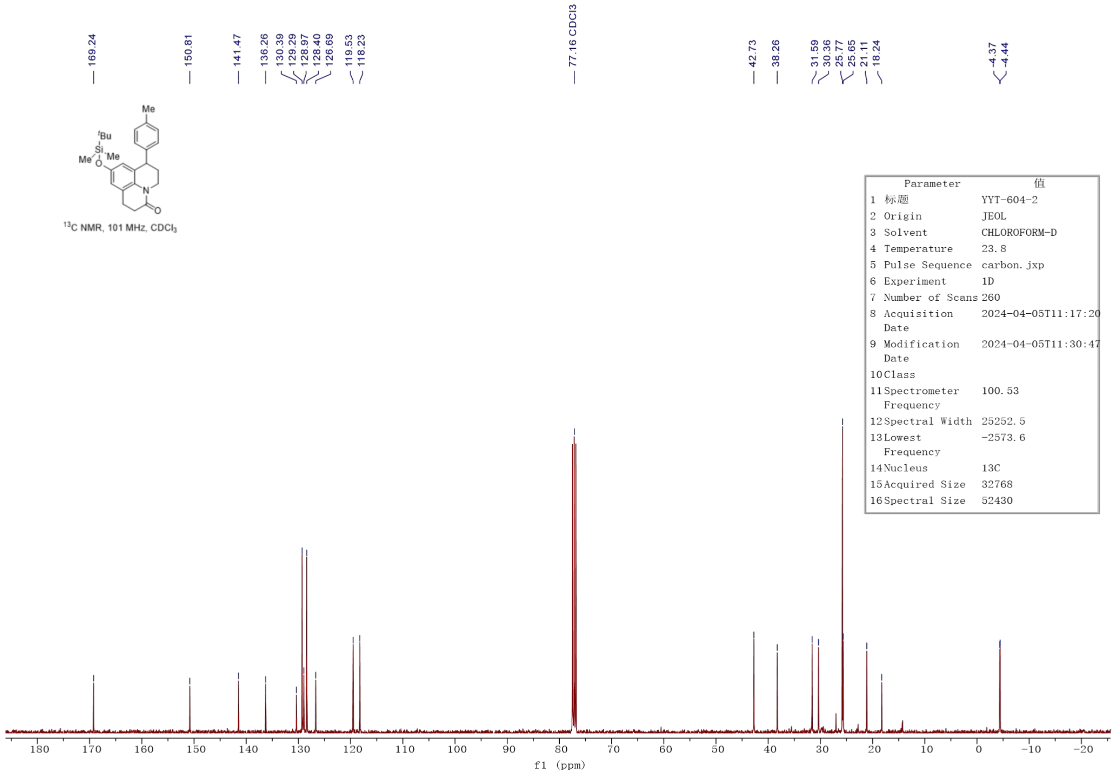

5-oxo-1-(p-tolyl)-2,3,6,7-tetrahydro-1H,5H-pyrido[3,2,1-ij]quinolin-9-yl benzoate (51)

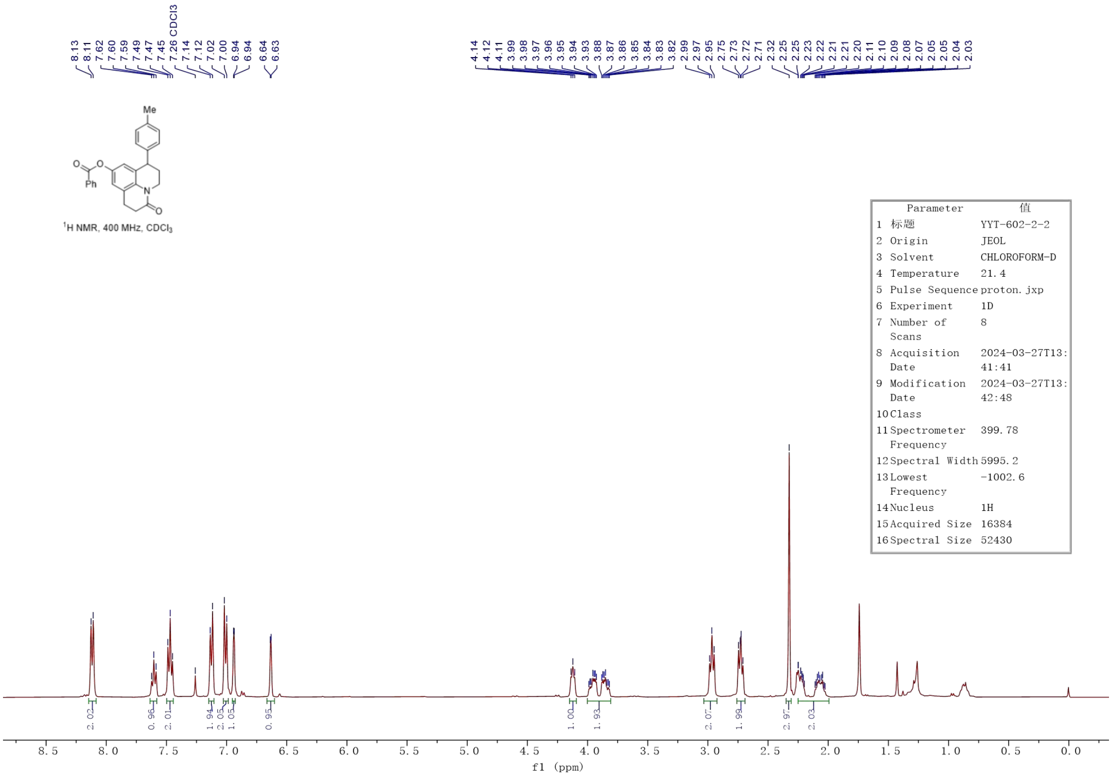

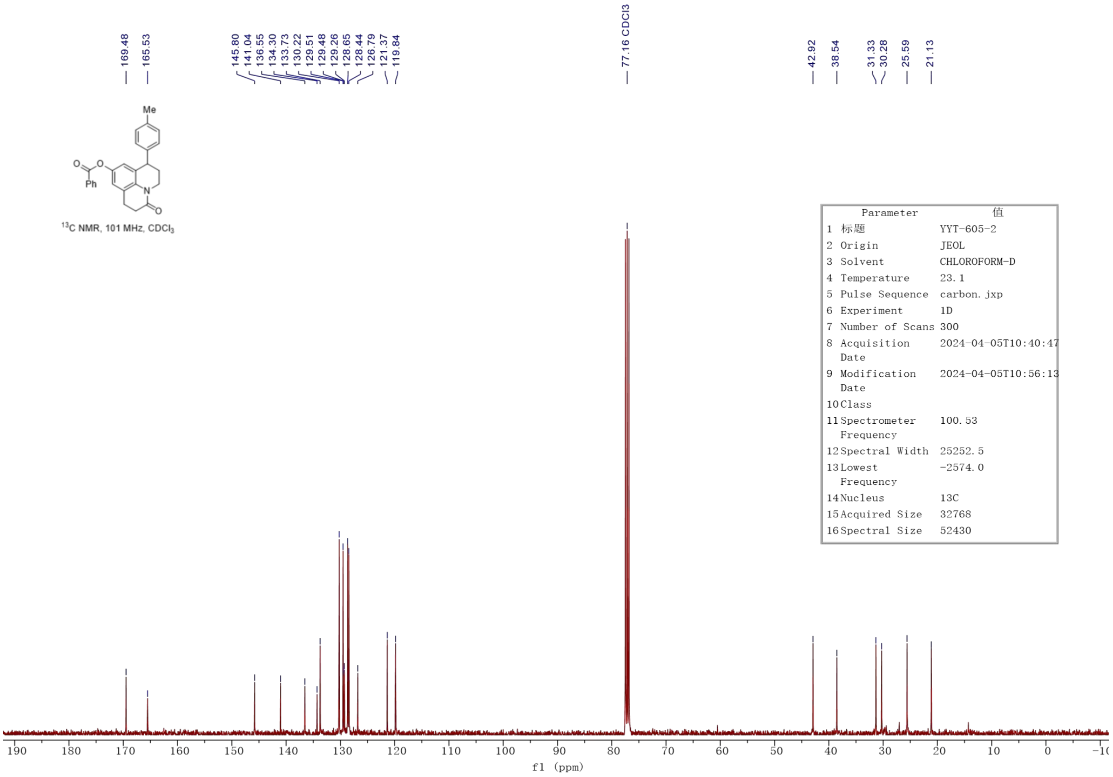

9-bromo-7,7-dimethyl-1-(p-tolyl)-2,3,6,7-tetrahydro-1H,5H-pyrido[3,2,1-iJ]quinolin-5-one (52)

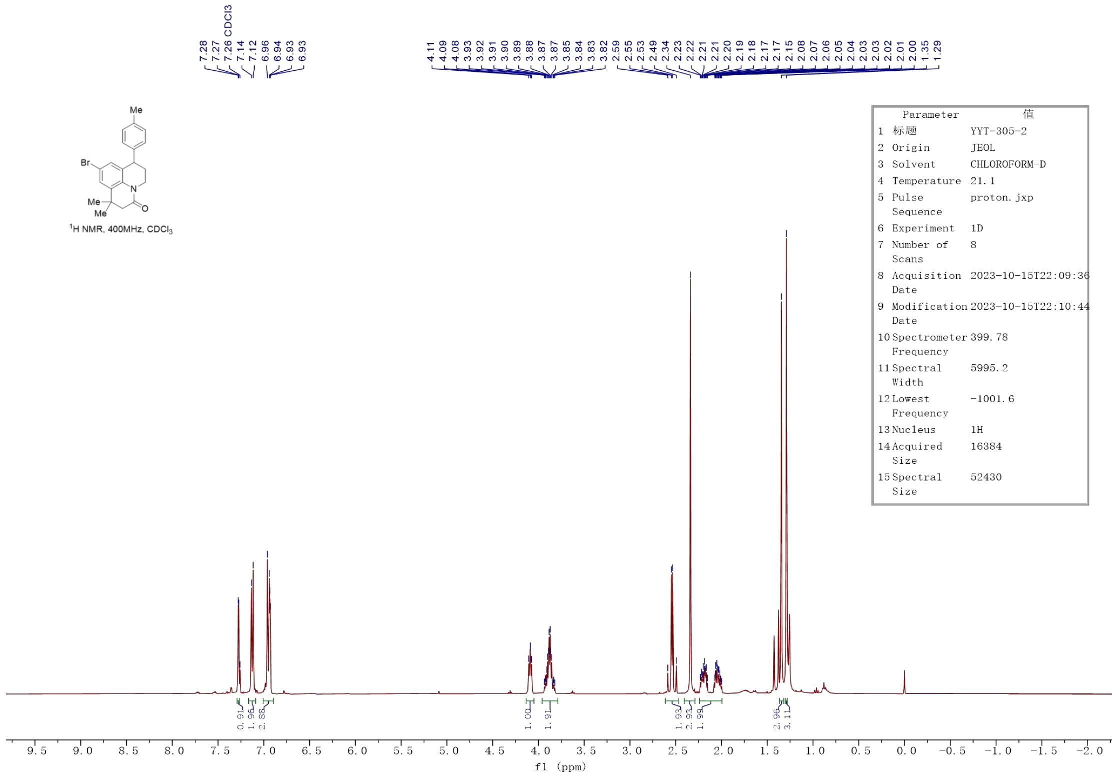

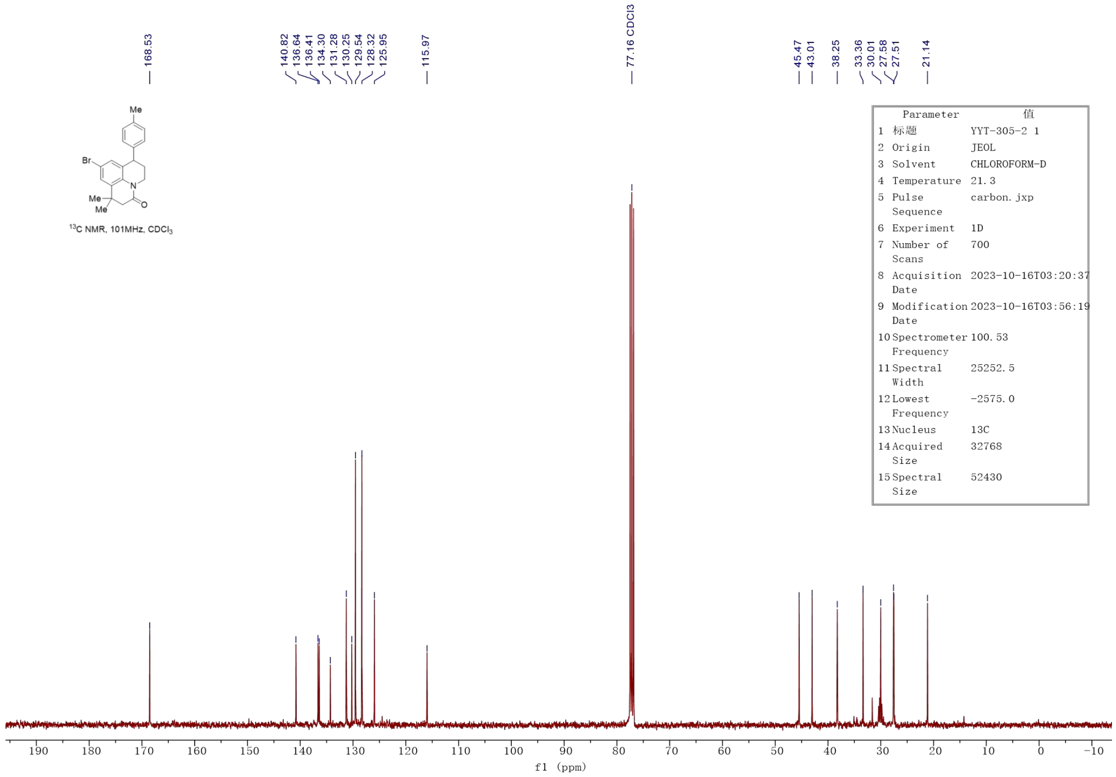

9-fluoro-1-(p-tolyl)-2,3,6,7-tetrahydro-1H,5H-pyrido[3,2,1-iJ]quinolin-5-one (53)

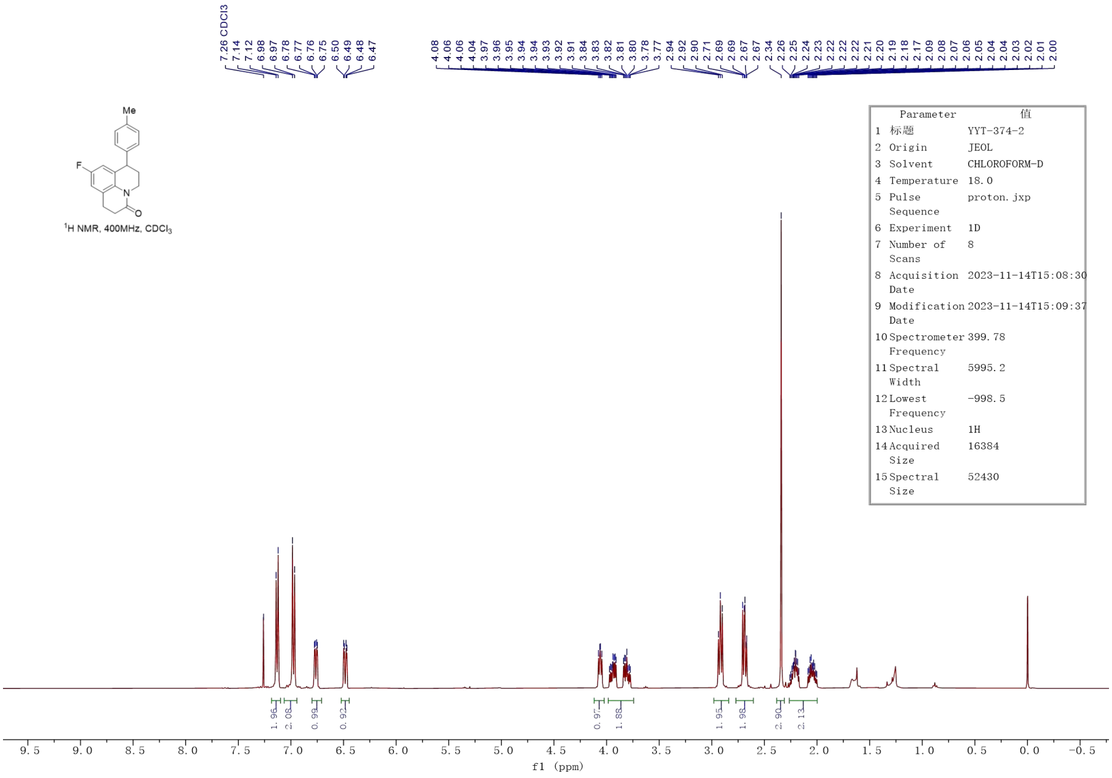

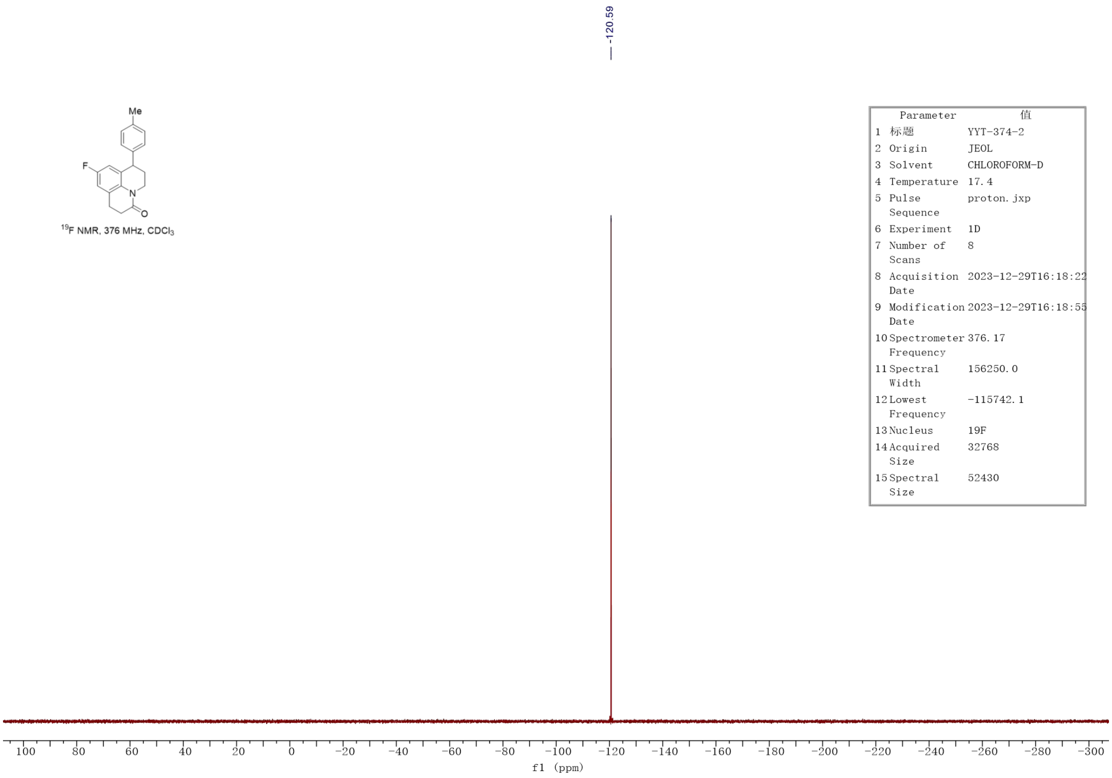

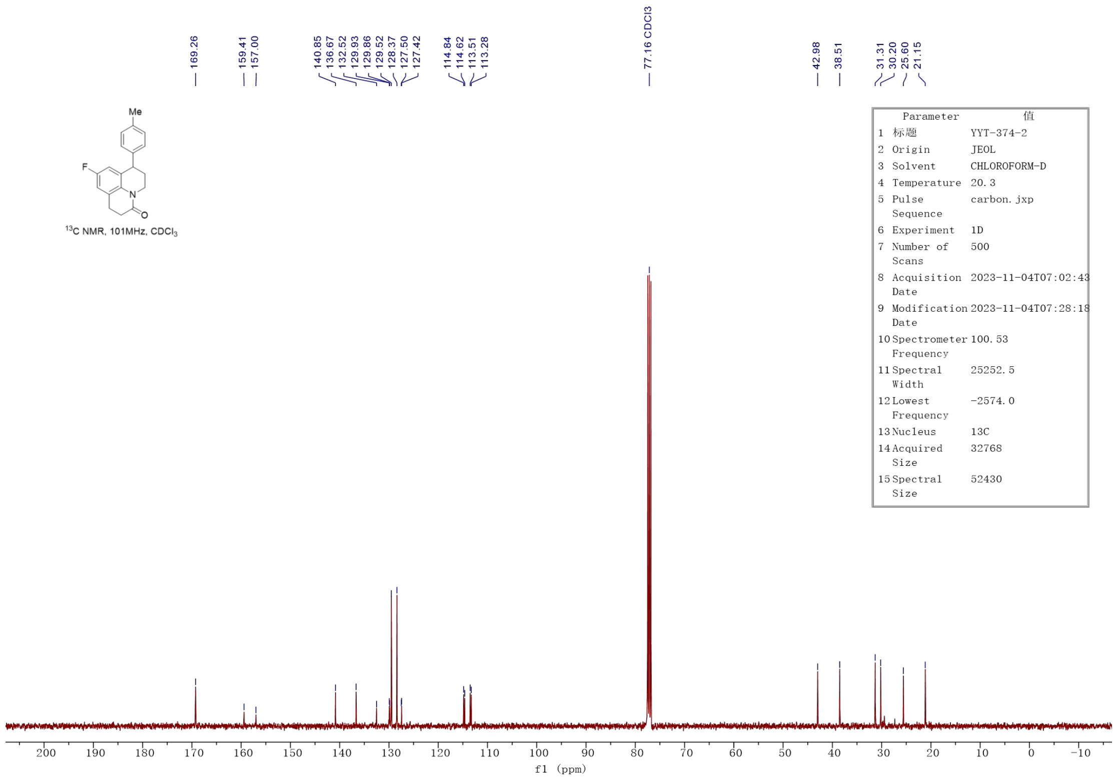
169.26
159.41
157.00
140.85
136.67
132.52
129.93
129.86
129.52
128.37
127.50
127.42
114.84
114.62
113.51
113.28
77.16 CDCl3
42.98
38.51
31.31
30.20
25.60
21.15
Me
F
N
O
^{13}C NMR, 101MHz, $CDCl_3$
Parameter 值
1 标题 YYT-374-2
2 Origin JEOL
3 Solvent CHLOROFORM-D
4 Temperature 20.3
5 Pulse Sequence carbon.jxp
6 Experiment 1D
7 Number of Scans 500
8 Acquisition Date 2023-11-04T07:02:43
9 Modification Date 2023-11-04T07:28:18
10 Spectrometer Frequency 100.53
11 Spectral Width 25252.5
12 Lowest Frequency -2574.0
13 Nucleus 13C
14 Acquired Size 32768
15 Spectral Size 52430
200 190 180 170 160 150 140 130 120 110 100 90 80 70 60 50 40 30 20 10 0 -10
f1 (ppm)

9-chloro-1-(p-tolyl)-2,3,6,7-tetrahydro-1H,5H-pyrido[3,2,1-iJ]quinolin-5-one (54)

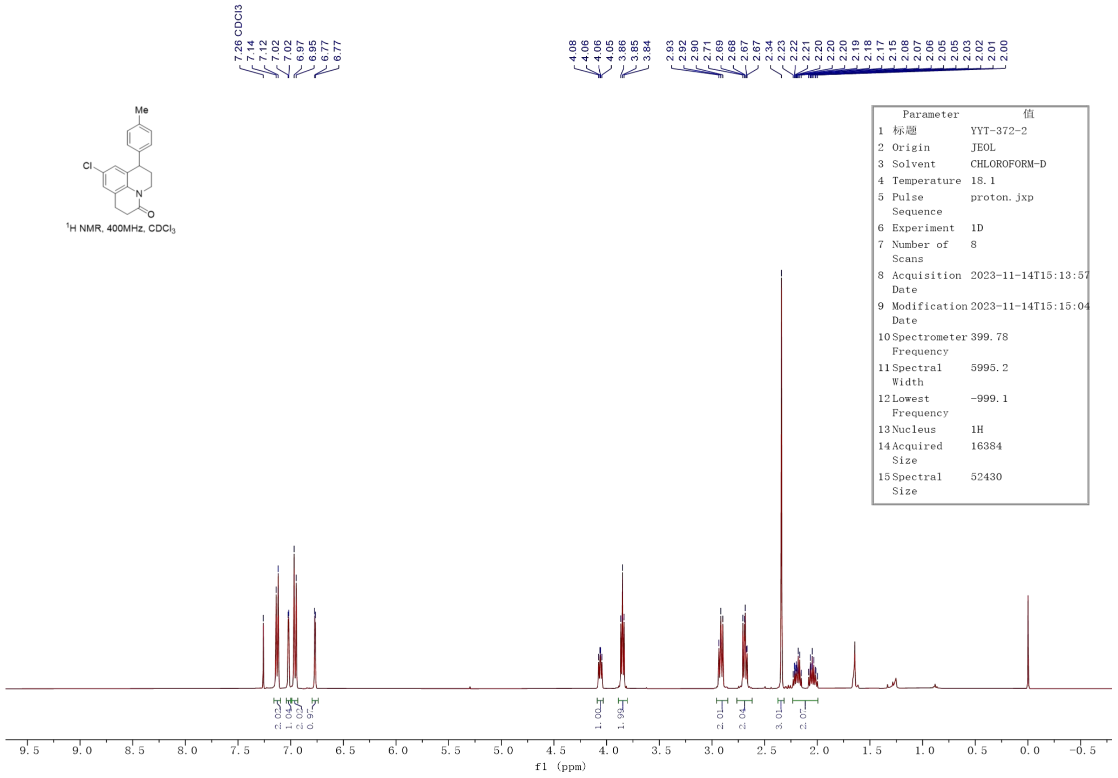

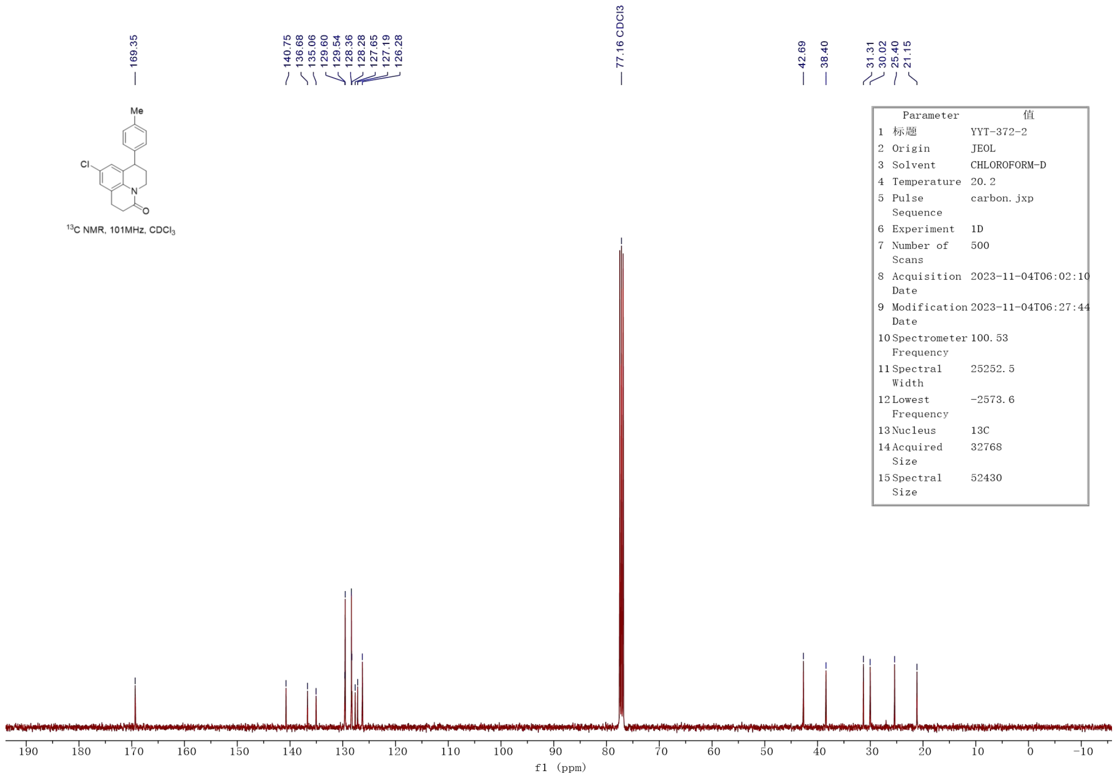

9-bromo-1-(p-tolyl)-2,3,6,7-tetrahydro-1H,5H-pyrido[3,2,1-iJ]quinolin-5-one (55)

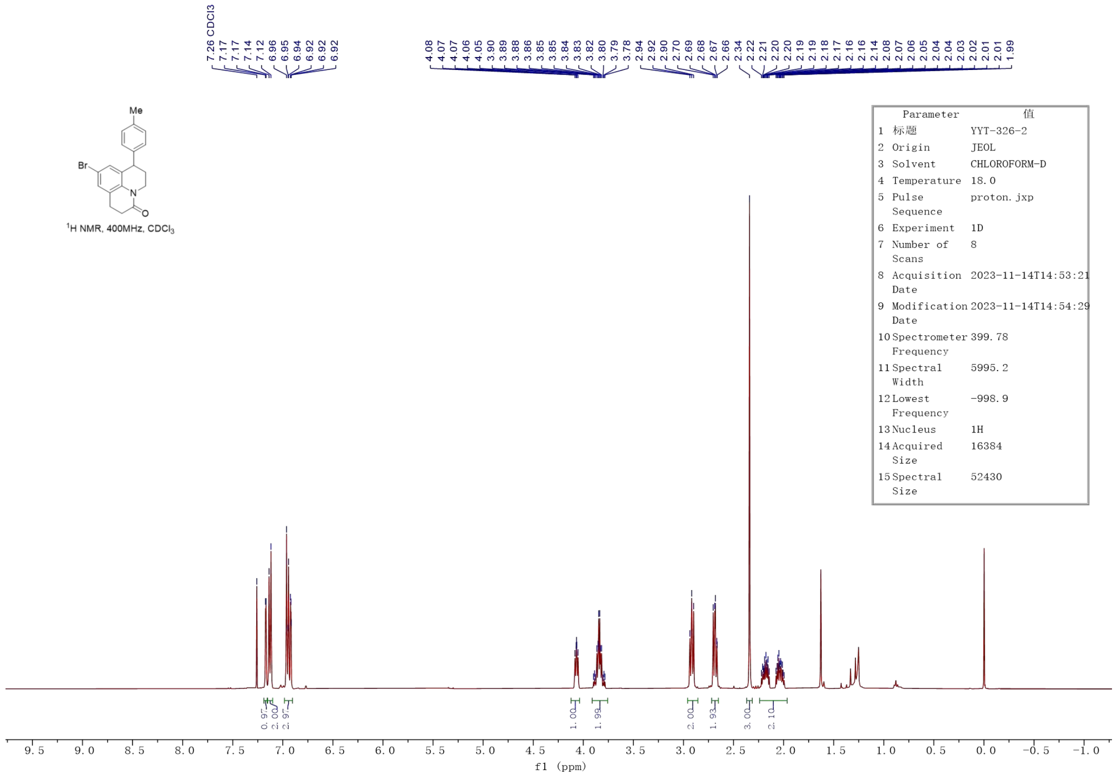

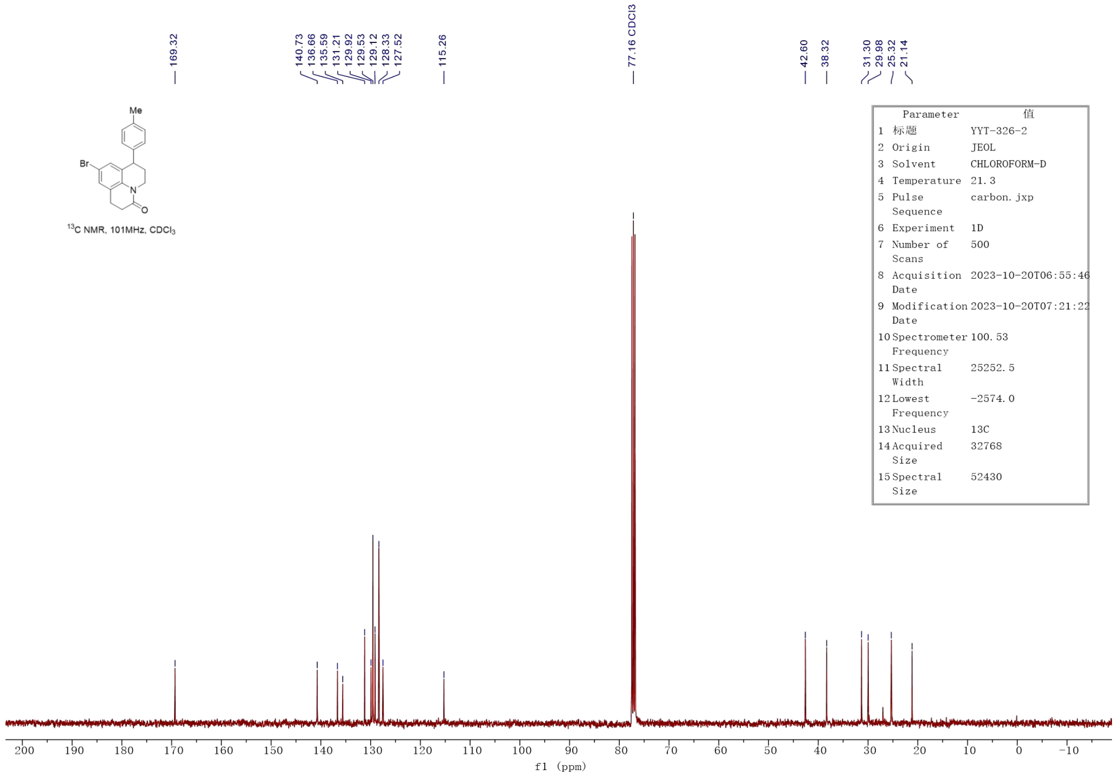

8-methyl-1-(p-tolyl)-2,3,6,7-tetrahydro-1H,5H-pyrido[3,2,1-ij]quinolin-5-one (56)

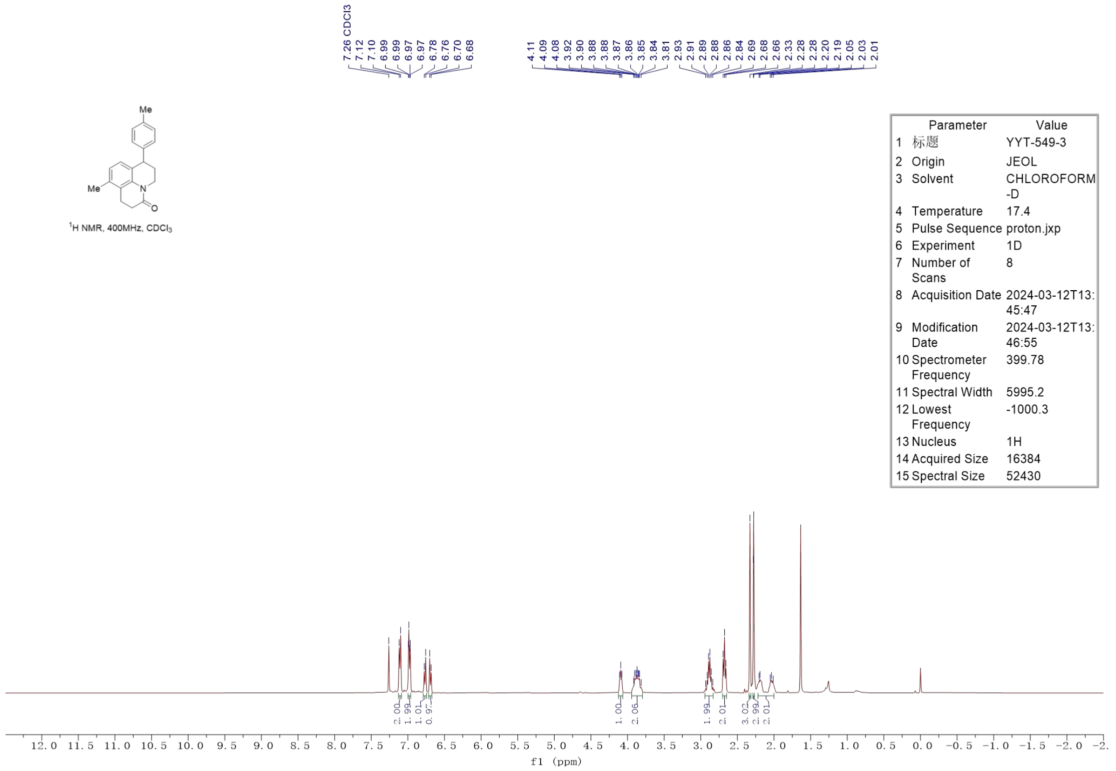

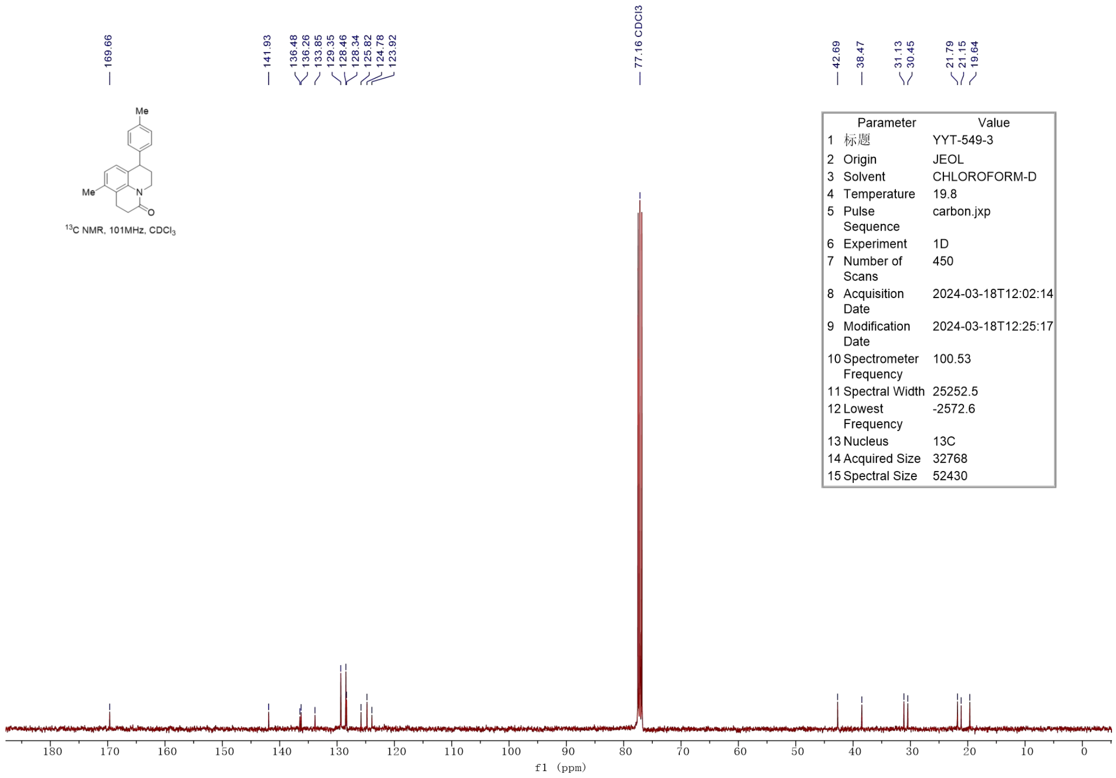

7-(p-tolyl)-6,7-dihydro-5H-[1,4]thiazino[2,3,4-iJ]quinolin-3(2H)-one (57)

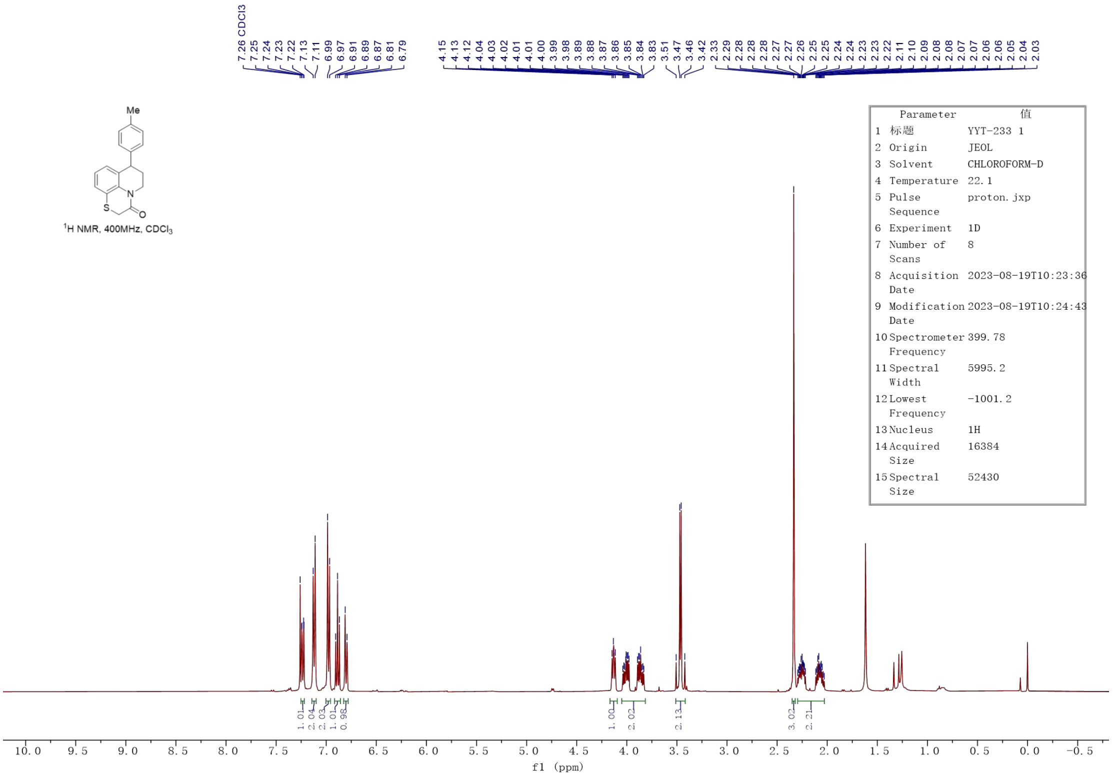

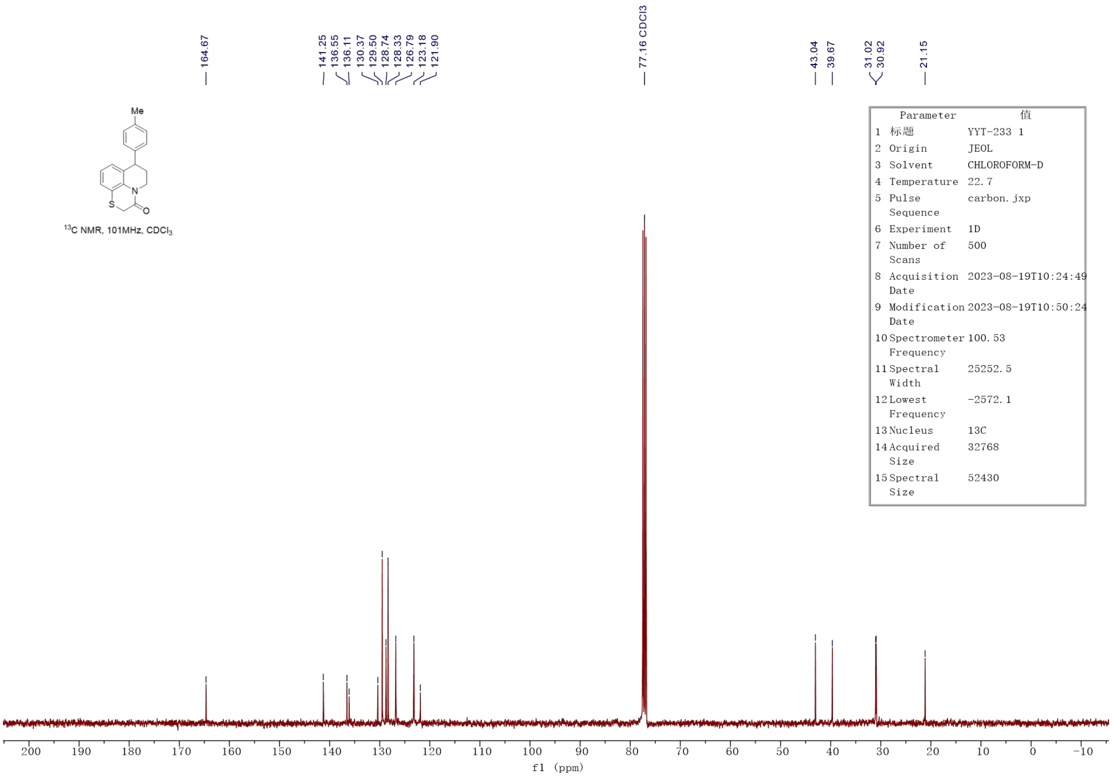

9-methyl-7-(p-tolyl)-6,7-dihydro-5H-[1,4]thiazino[2,3,4-iJ]quinolin-3(2H)-one (58)

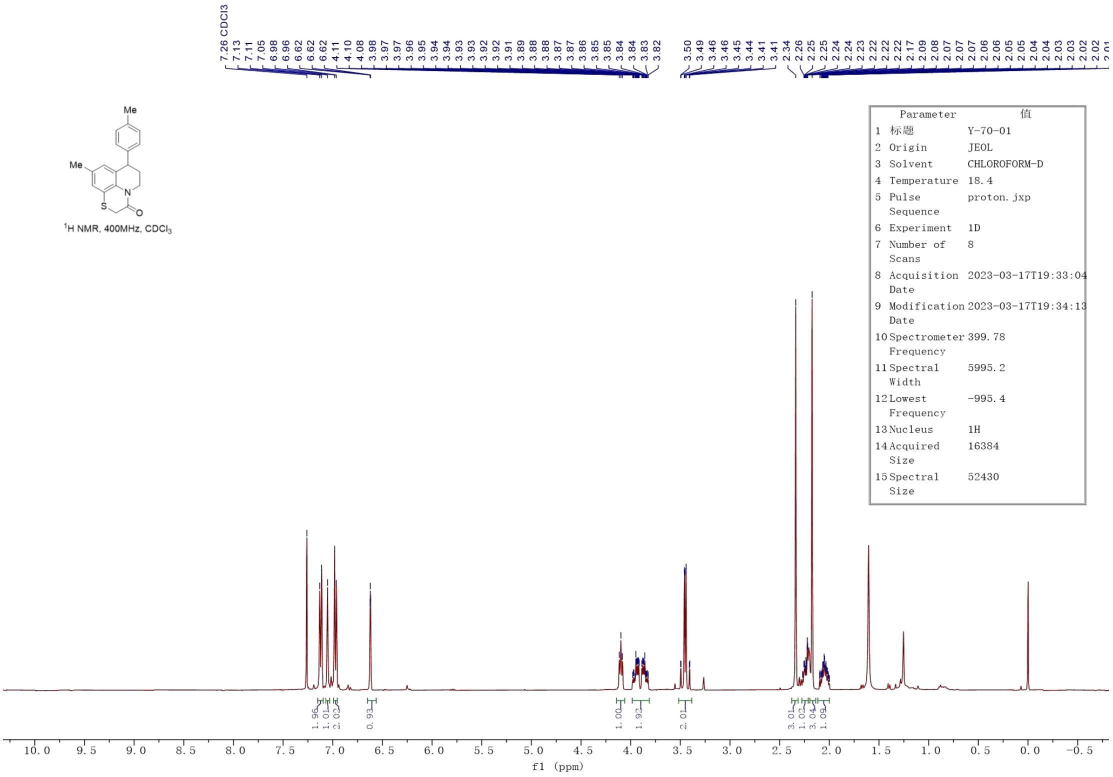

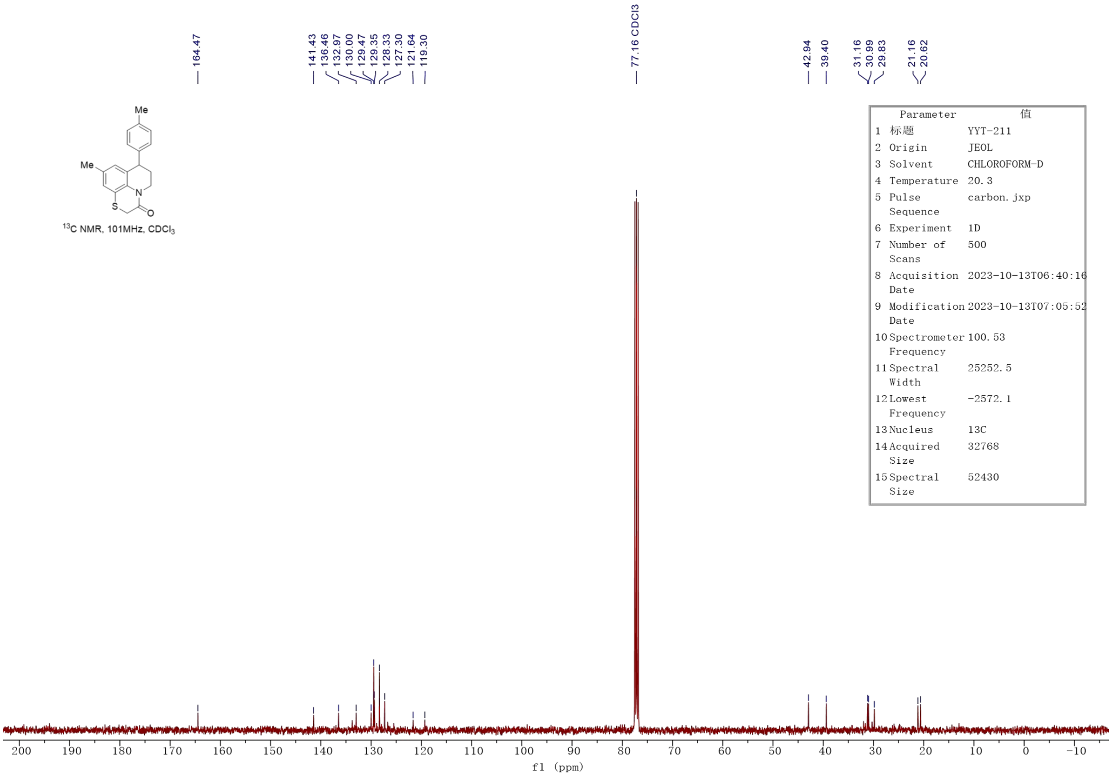

9-bromo-7-(p-tolyl)-6,7-dihydro-5H-[1,4]thiazino[2,3,4-iJ]quinolin-3(2H)-one (59)

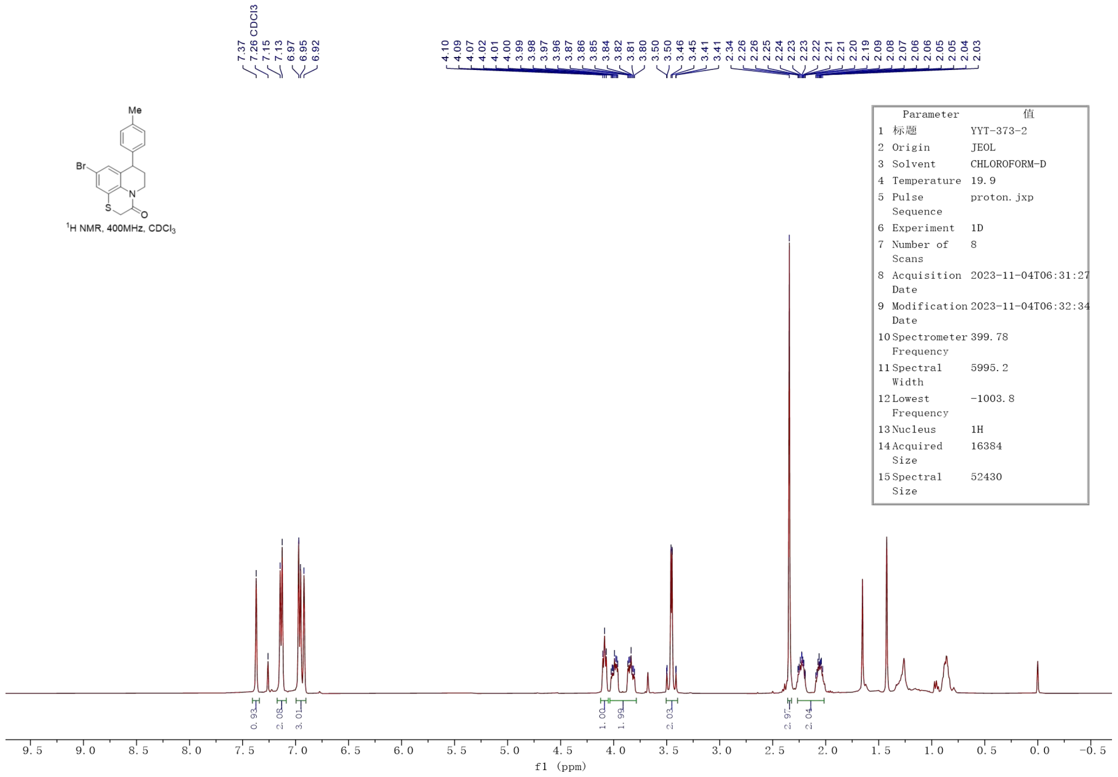

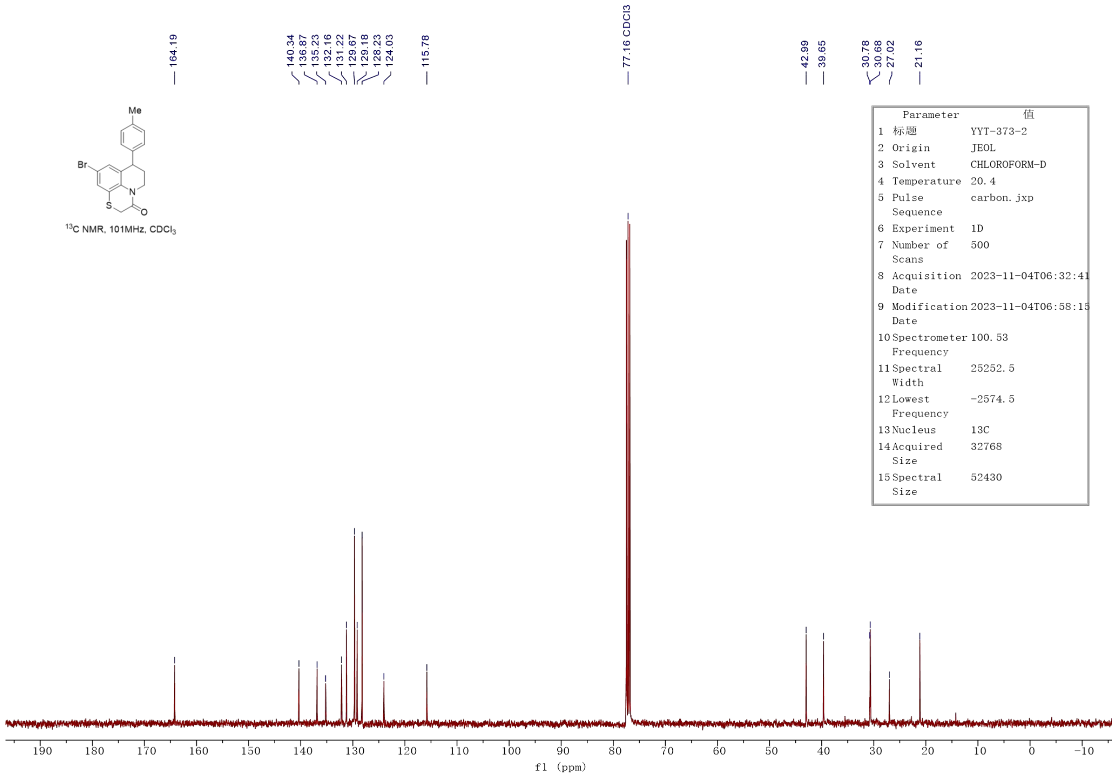

1,1-dimethyl-6-(p-tolyl)-5,6-dihydro-4H-pyrrolo[3,2,1-iJ]quinolin-2(1H)-one (60)

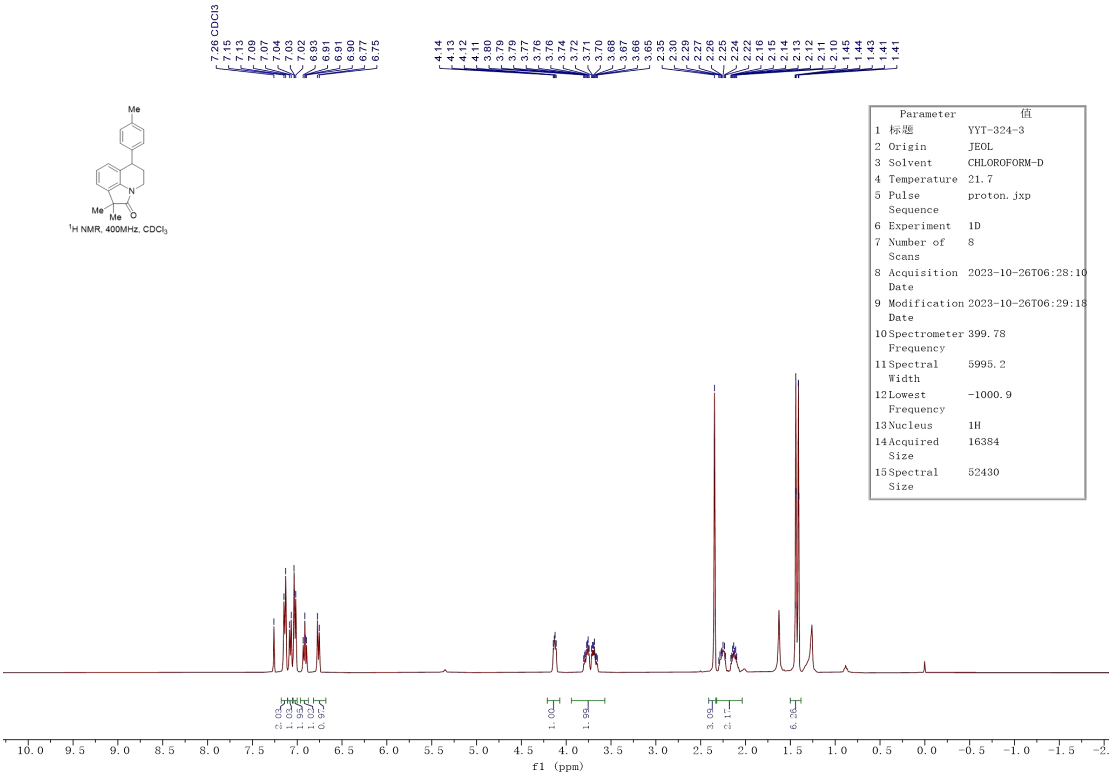

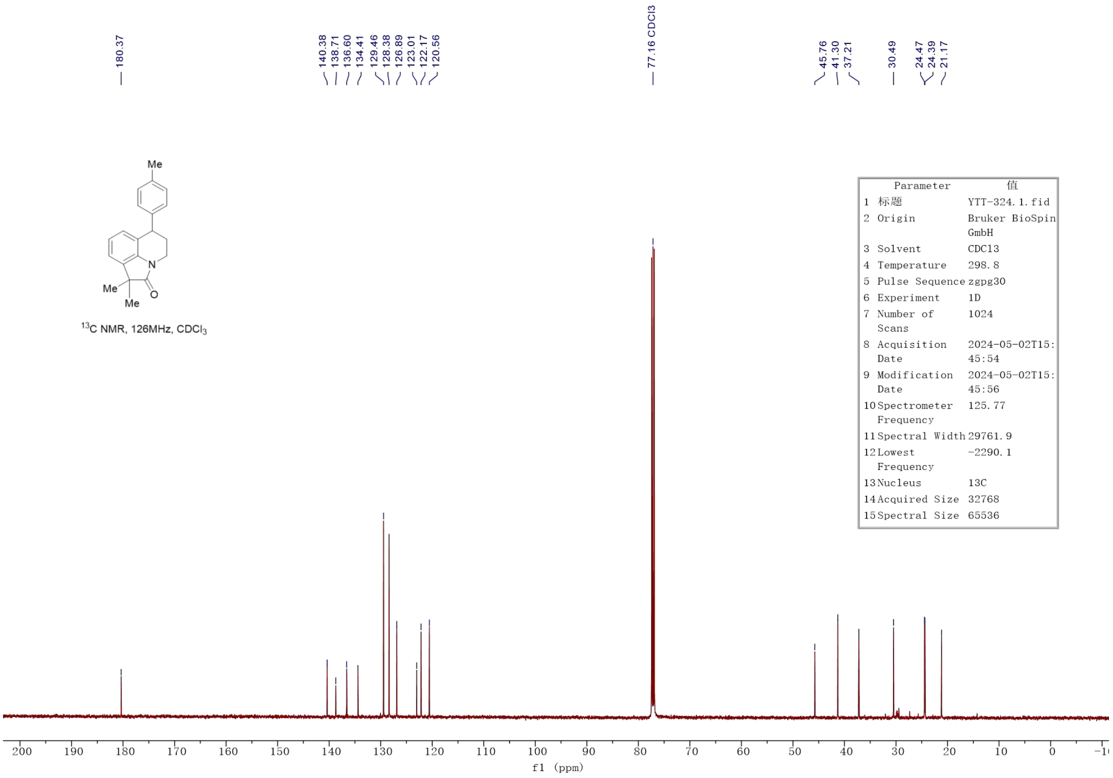

1-(p-tolyl)-2,3-dihydroisoindolo[7,1,2-hiJ]quinolin-5(1H)-one (61)

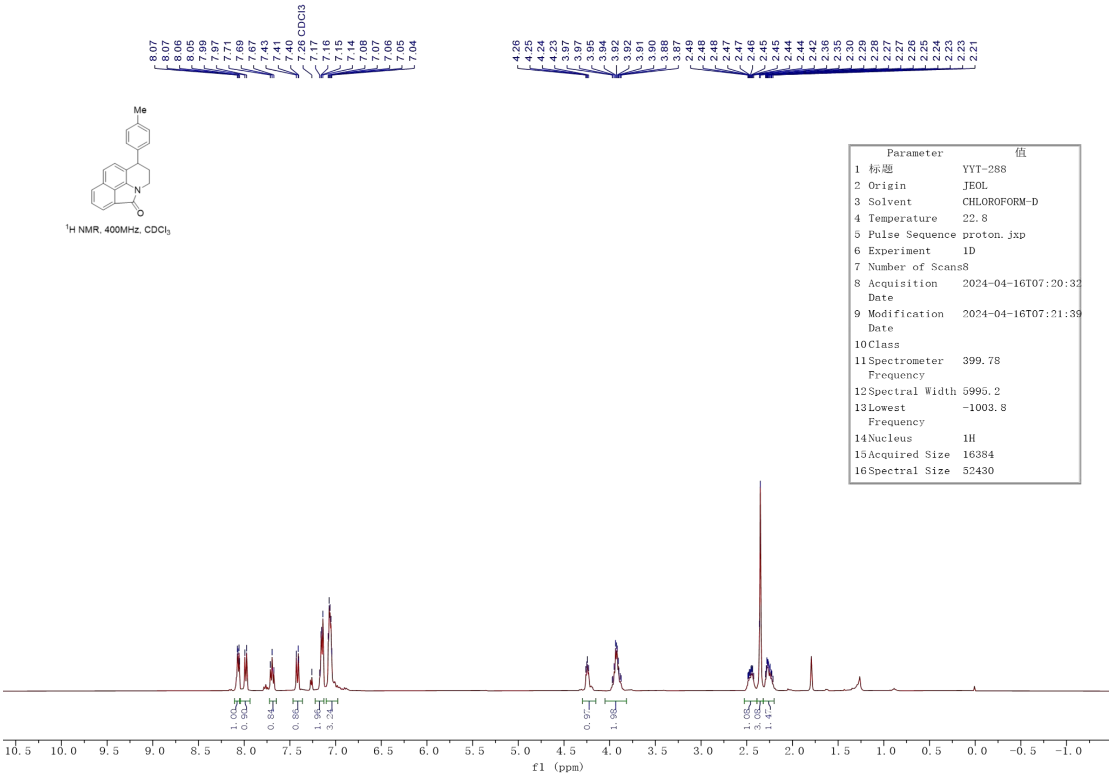

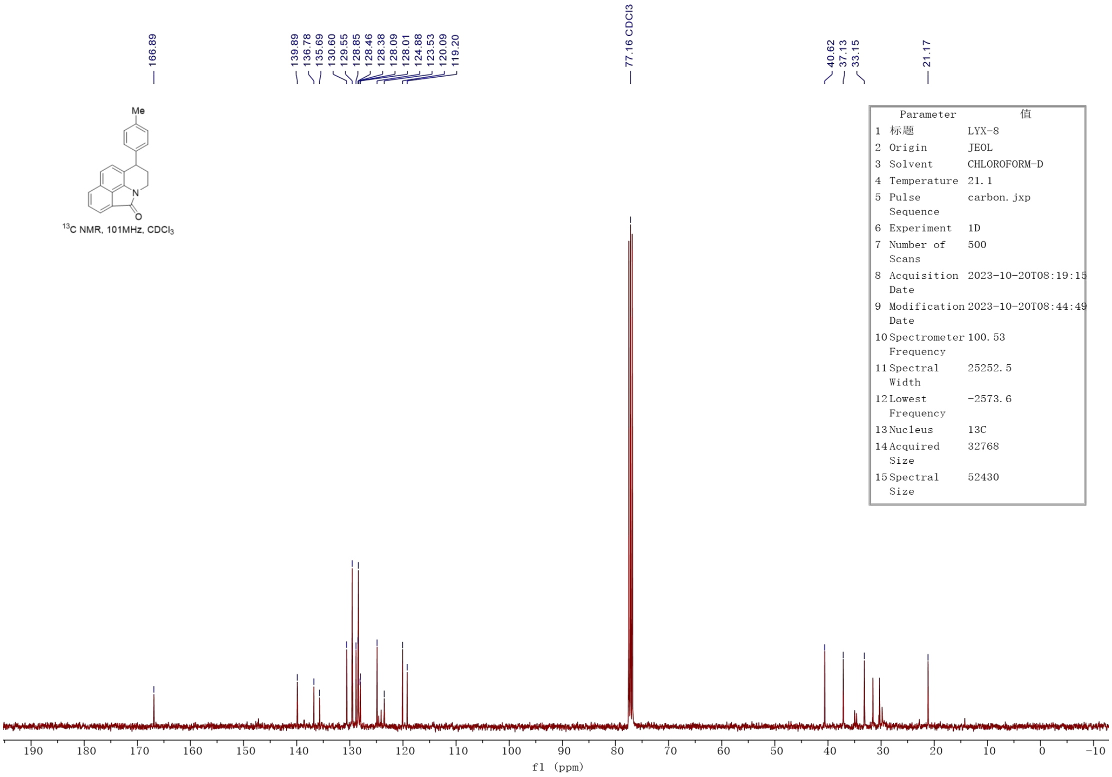

1-(p-tolyl)-2,3-dihydro-1H-4-thia-3a-azacyclopenta[def]phenanthrene 4,4-dioxide (62)

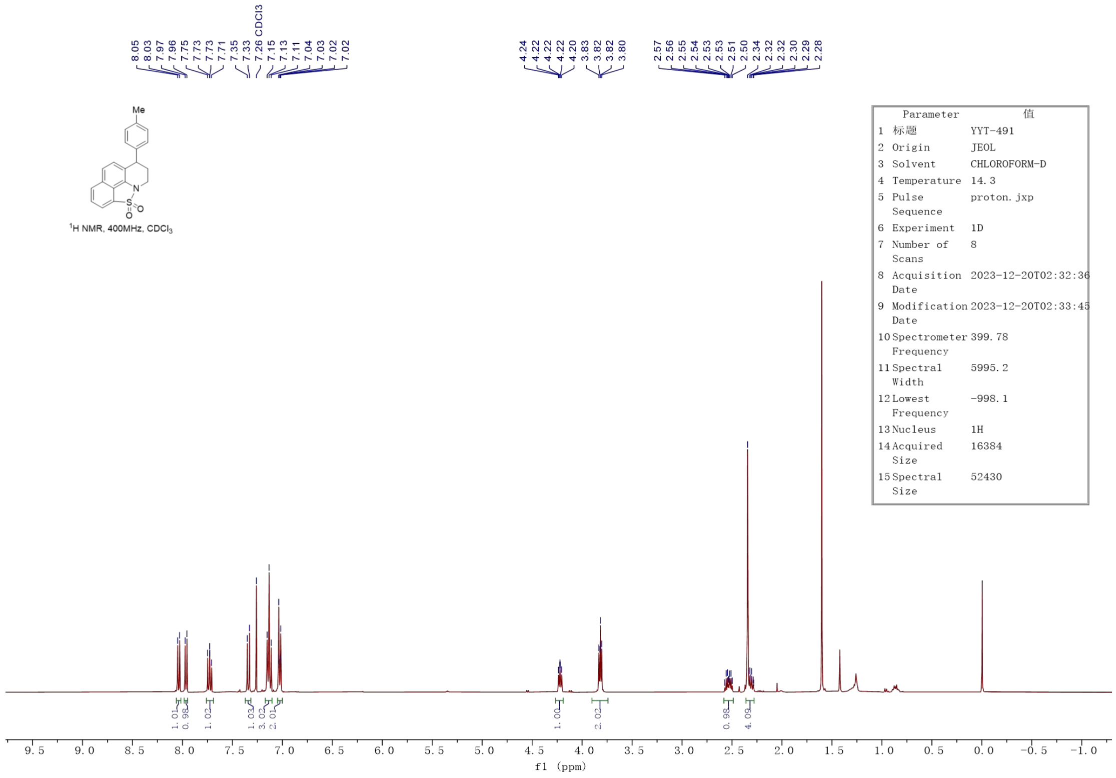

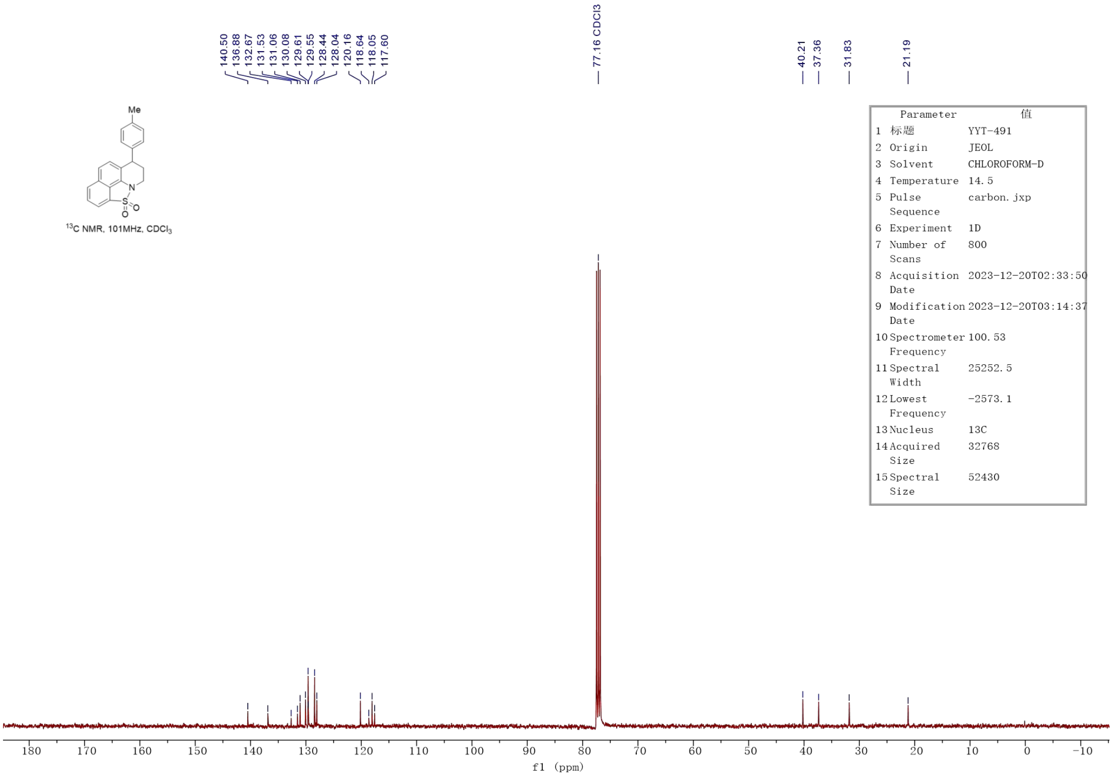

1-(p-tolyl)-2,3,7,8-tetrahydro-1H-azepino[3,2,1-iJ]quinolin-5(6H)-one (63)

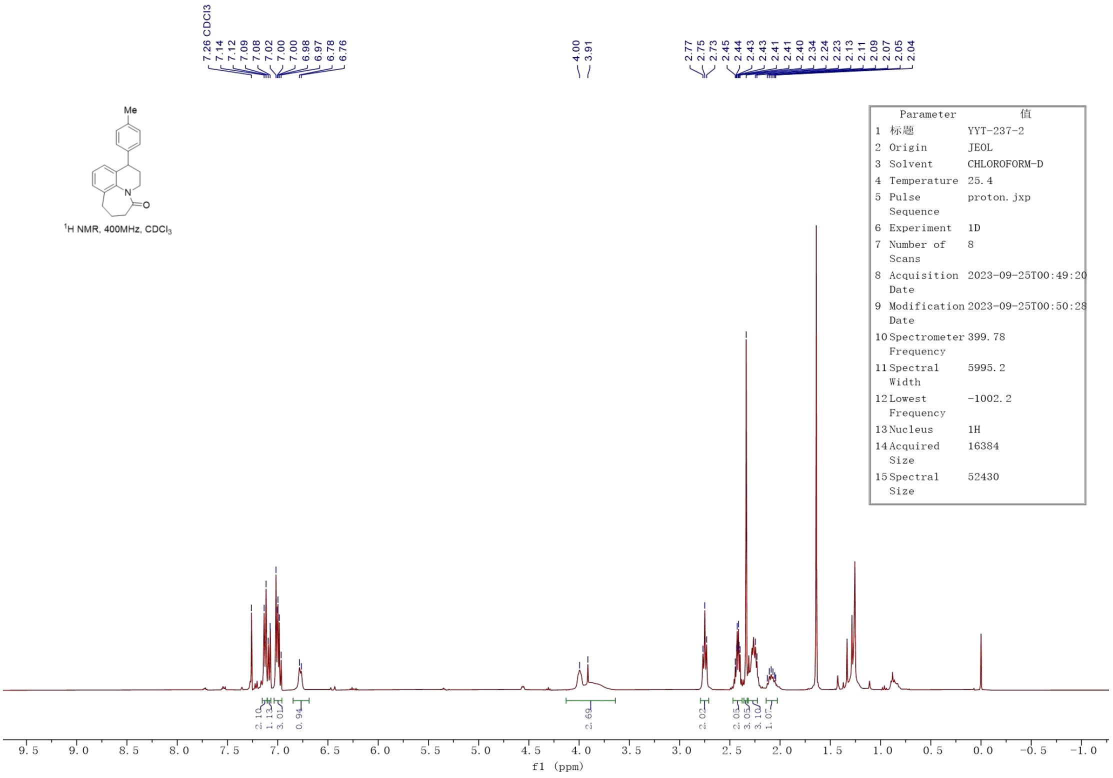

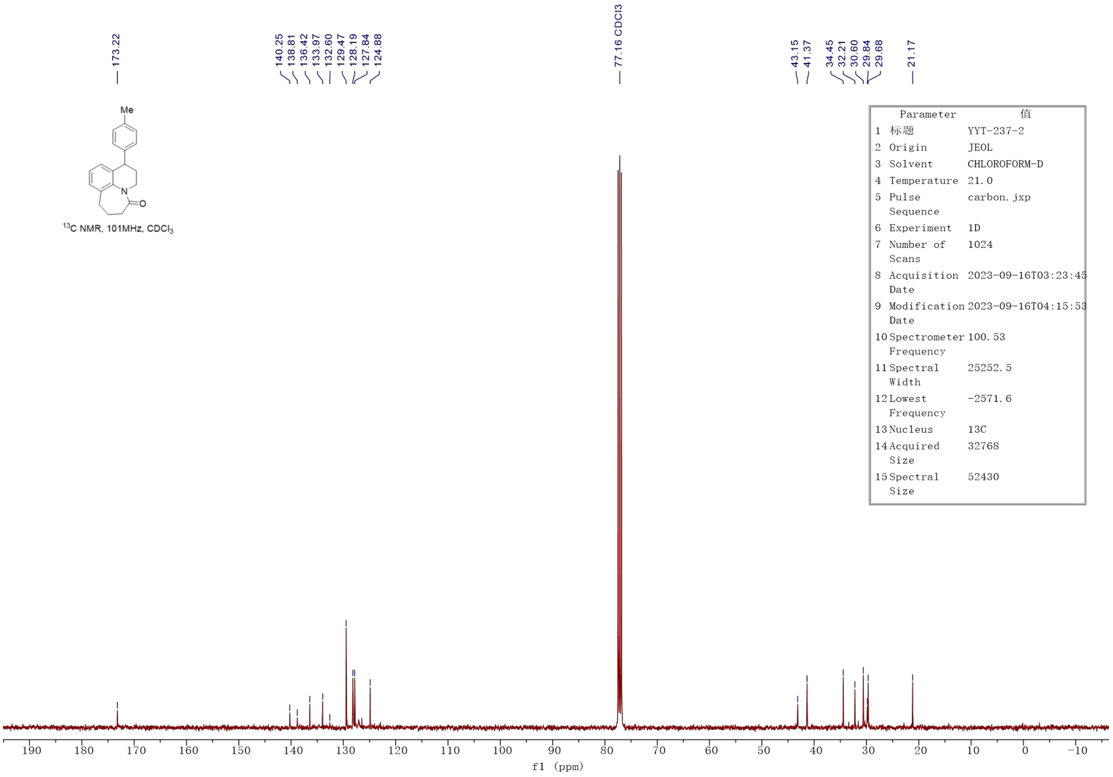

6-methyl-7-(p-tolyl)-6,7-dihydro-5H-[1,4]oxazino[2,3,4-iJ]quinolin-3(2H)-one (64)

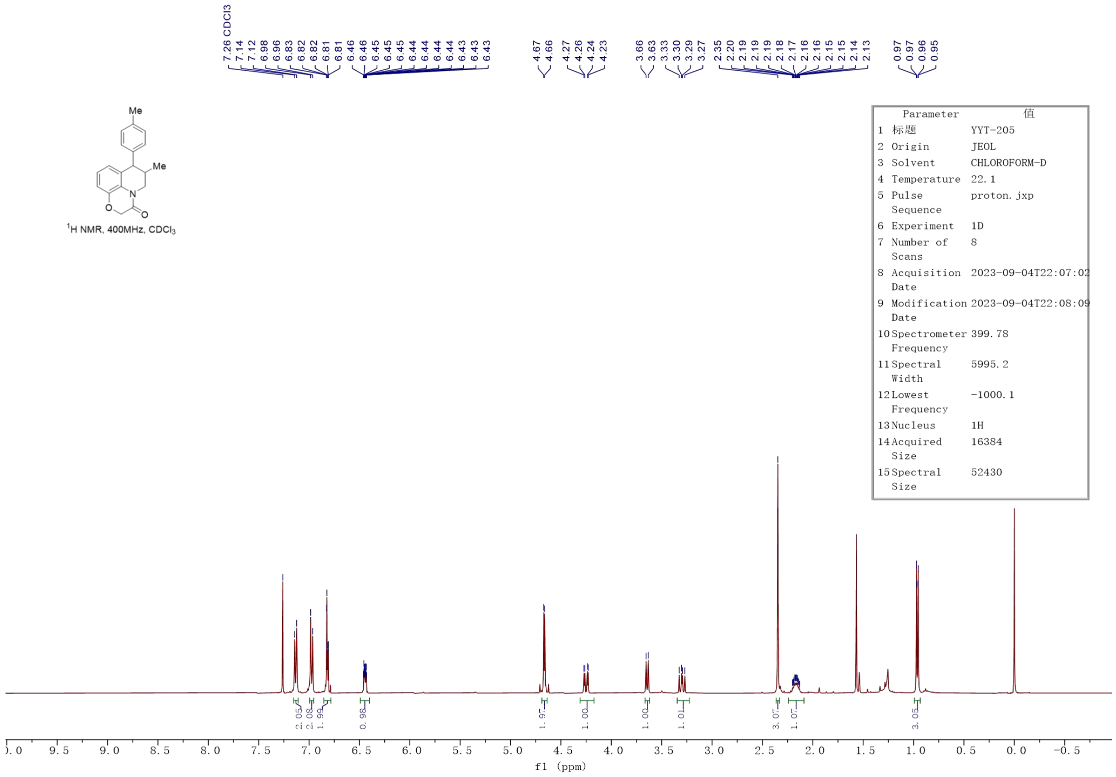

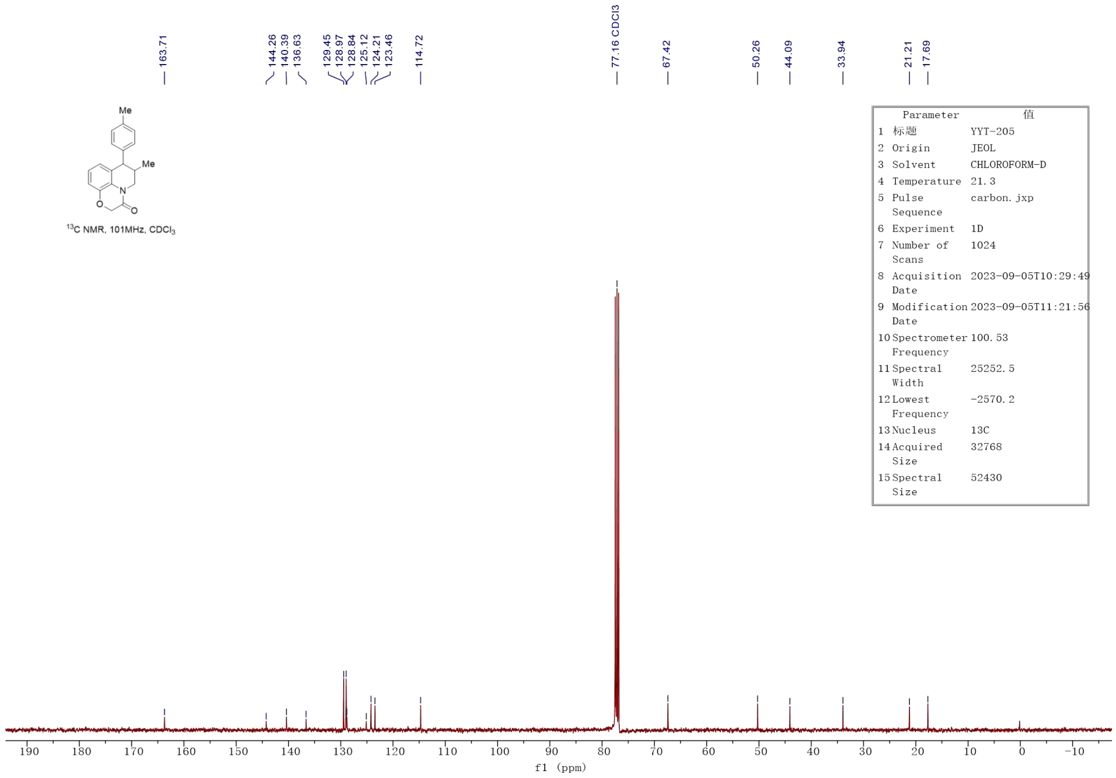

Methyl(7R)-3-oxo-7-(p-tolyl)-2,3,6,7-tetrahydro-5H-[1,4]oxazino[2,3,4-ij]quinoline-6-carboxylate (65)

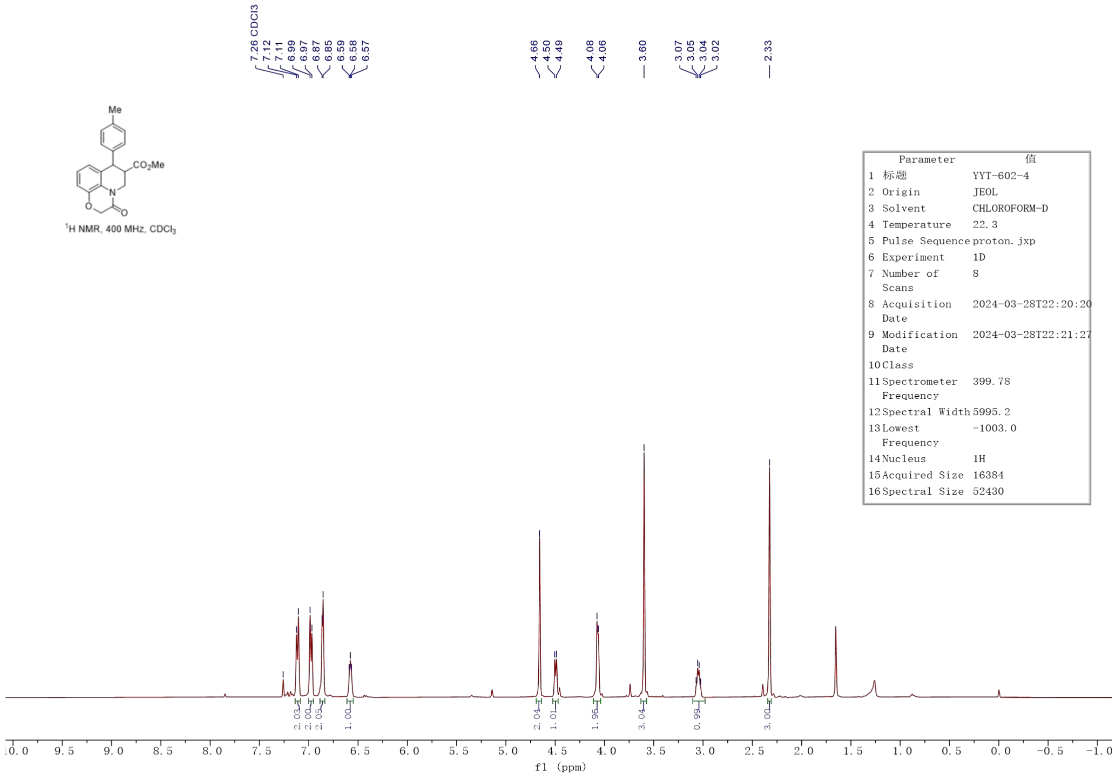

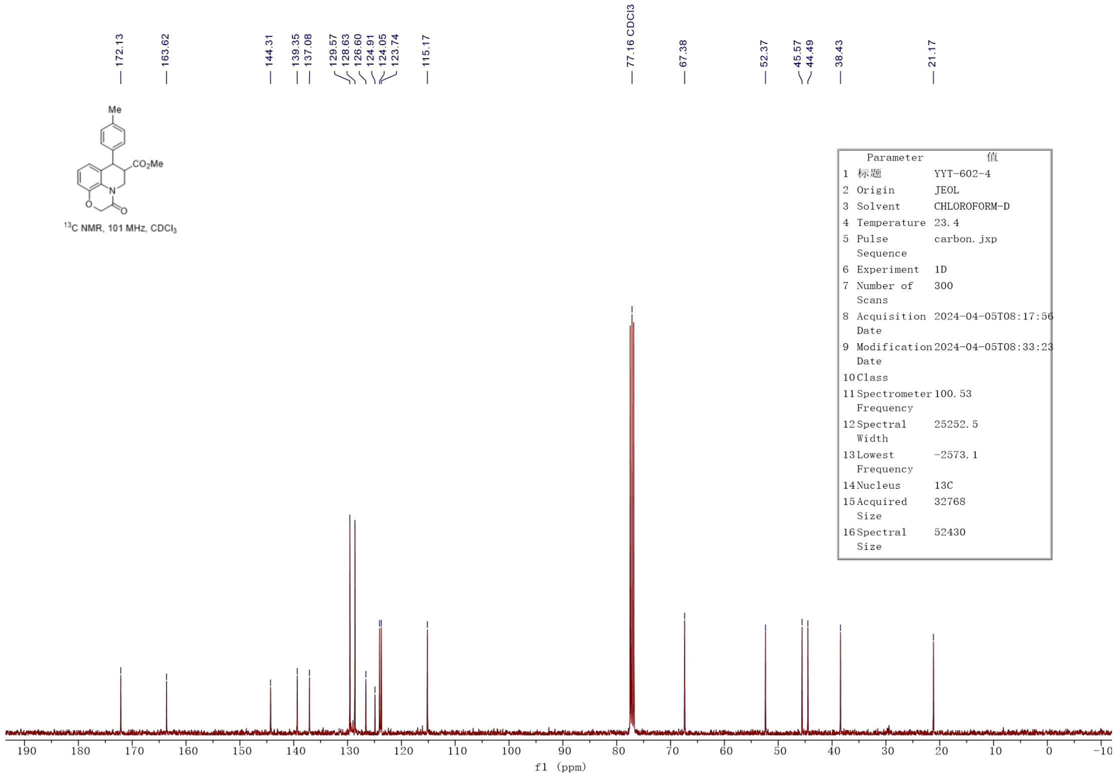

3-(p-tolyl)-2,3-dihydro-1H-pyrido[3,2,1-kl]phenoxazine (66)

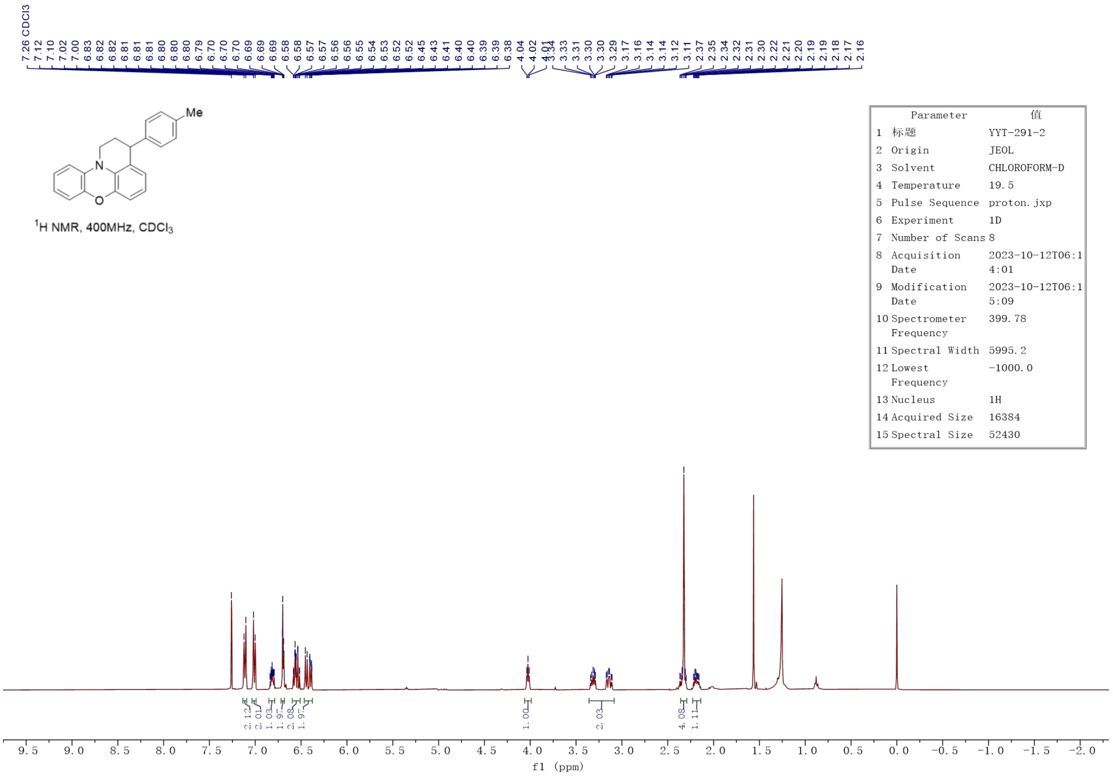

Parameter	值
1 标题	YYT-291-2
2 Origin	JEOL
3 Solvent	CHLOROFORM-D
4 Temperature	19.5
5 Pulse Sequence	proton.jxp
6 Experiment	1D
7 Number of Scans	8
8 Acquisition Date	2023-10-12T06:14:01
9 Modification Date	2023-10-12T06:15:09
10 Spectrometer Frequency	399.78
11 Spectral Width	5995.2
12 Lowest Frequency	-1000.0
13 Nucleus	1H
14 Acquired Size	16384
15 Spectral Size	52430

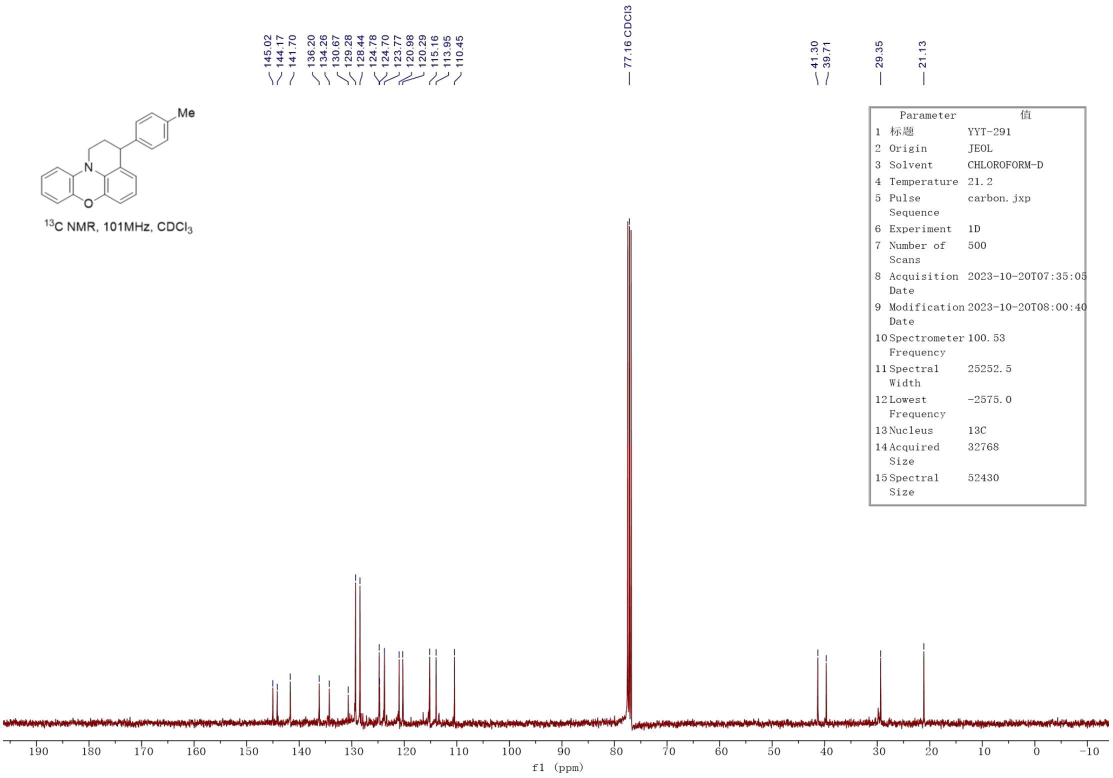

Parameter	值
1 标题	YYT-291
2 Origin	JEOL
3 Solvent	CHLOROFORM-D
4 Temperature	21.2
5 Pulse Sequence	carbon.jxp
6 Experiment	1D
7 Number of Scans	500
8 Acquisition Date	2023-10-20T07:35:05
9 Modification Date	2023-10-20T08:00:40
10 Spectrometer Frequency	100.53
11 Spectral Width	25252.5
12 Lowest Frequency	-2575.0
13 Nucleus	13C
14 Acquired Size	32768
15 Spectral Size	52430

1-(3-(p-tolyl)-2,3-dihydro-1H-pyrido[3,2,1-kl]phenothiazin-10-yl)ethan-1-one (67)

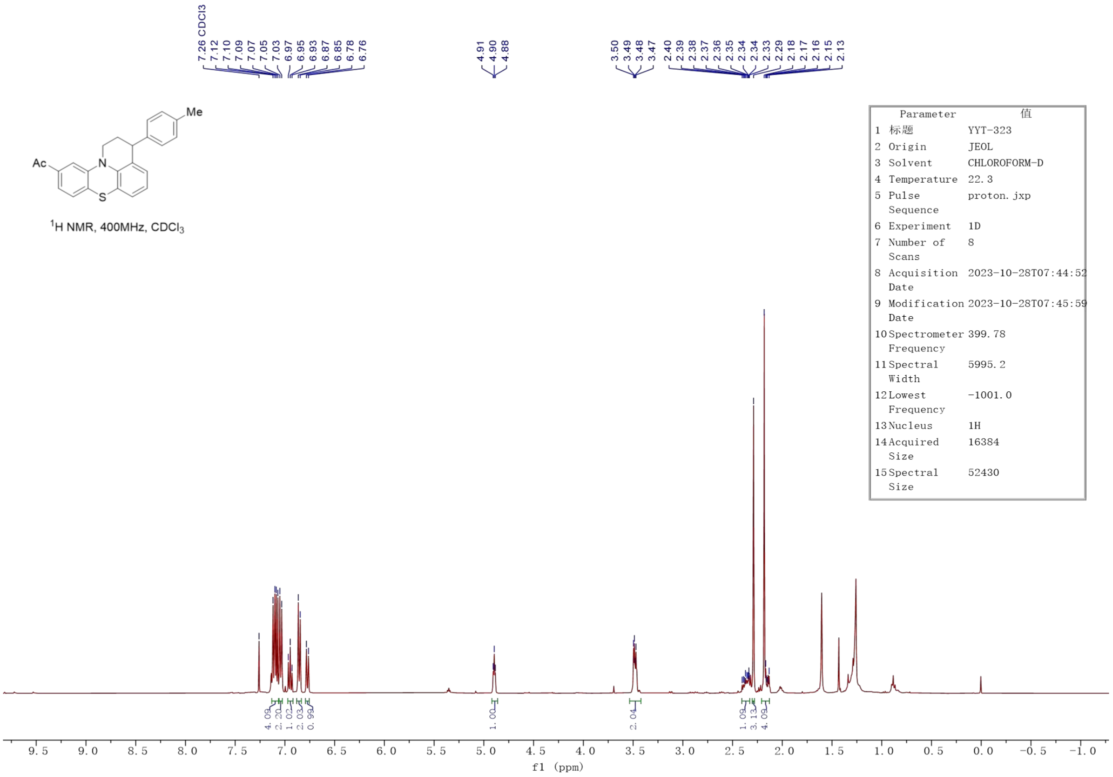

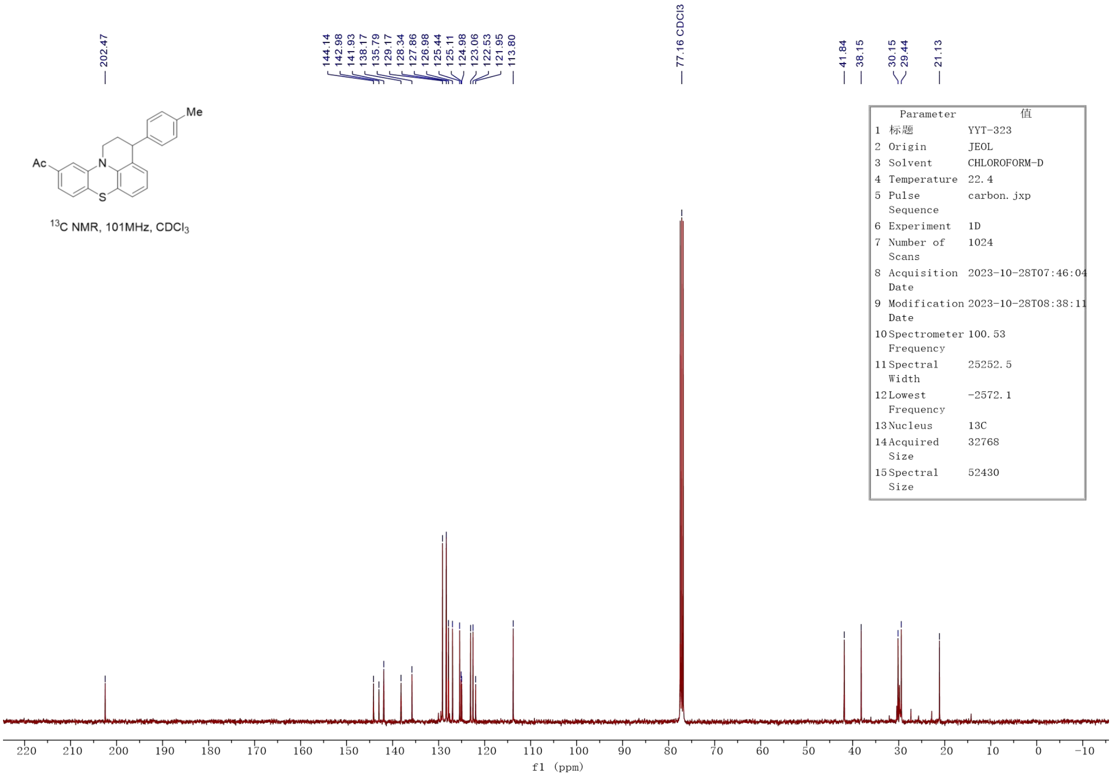

4-(p-tolyl)chromane (68)

^{1}H NMR, 400MHz, $CDCl_3$

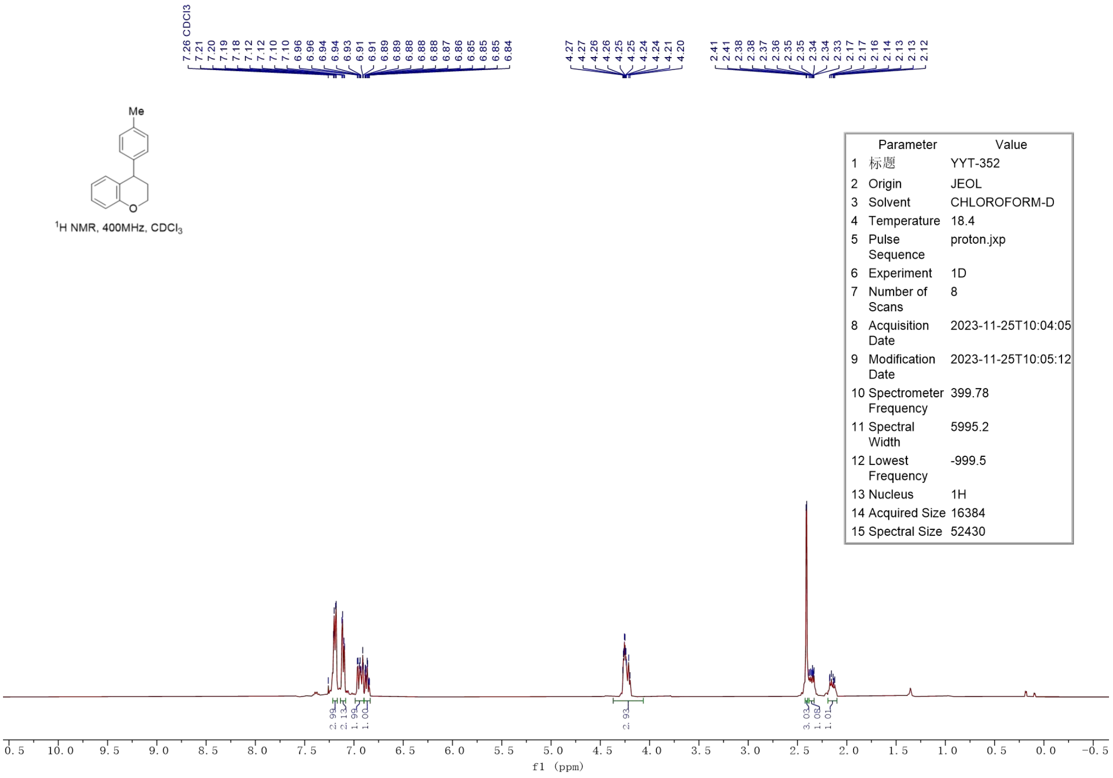

Parameter	Value
1 标题	YYT-352
2 Origin	JEOL
3 Solvent	CHLOROFORM-D
4 Temperature	18.4
5 Pulse Sequence	proton.jxp
6 Experiment	1D
7 Number of Scans	8
8 Acquisition Date	2023-11-25T10:04:05
9 Modification Date	2023-11-25T10:05:12
10 Spectrometer Frequency	399.78
11 Spectral Width	5995.2
12 Lowest Frequency	-999.5
13 Nucleus	1H
14 Acquired Size	16384
15 Spectral Size	52430

^{13}C NMR, 101MHz, $CDCl_3$

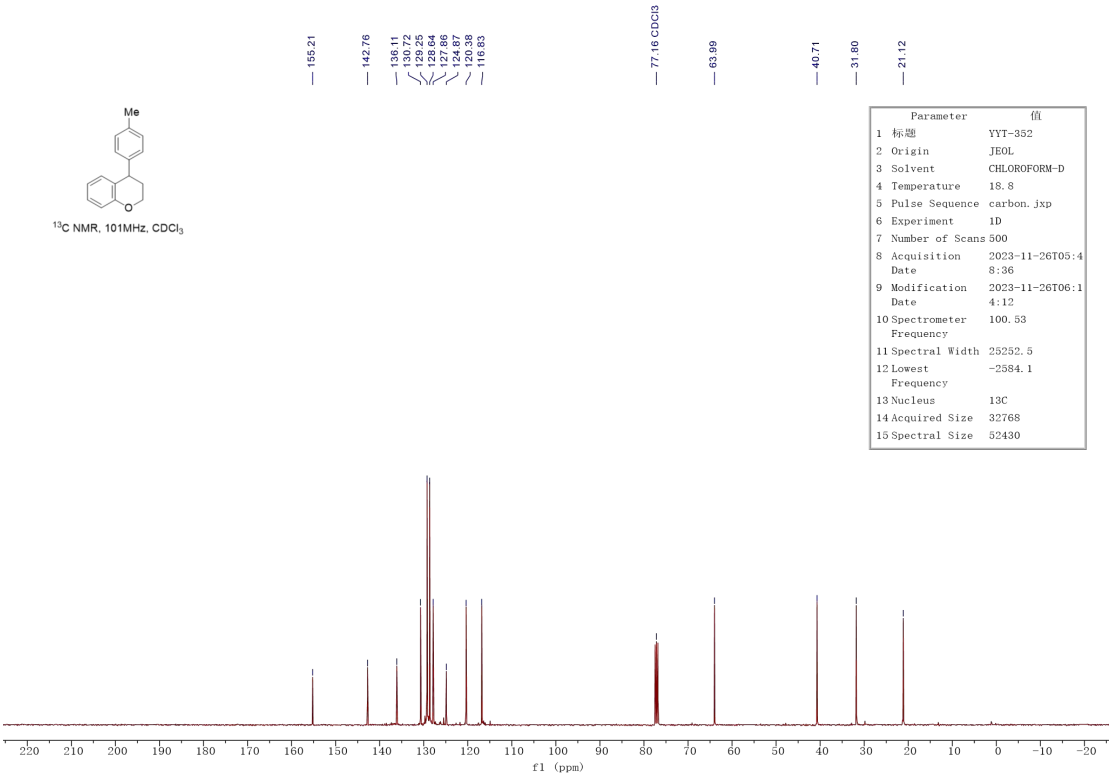

Parameter	值
1 标题	YYT-352
2 Origin	JEOL
3 Solvent	CHLOROFORM-D
4 Temperature	18.8
5 Pulse Sequence	carbon.jxp
6 Experiment	1D
7 Number of Scans	500
8 Acquisition Date	2023-11-26T05:48:36
9 Modification Date	2023-11-26T06:14:12
10 Spectrometer Frequency	100.53
11 Spectral Width	25252.5
12 Lowest Frequency	-2584.1
13 Nucleus	13C
14 Acquired Size	32768
15 Spectral Size	52430

6-methyl-4-(p-tolyl)chromane (69)

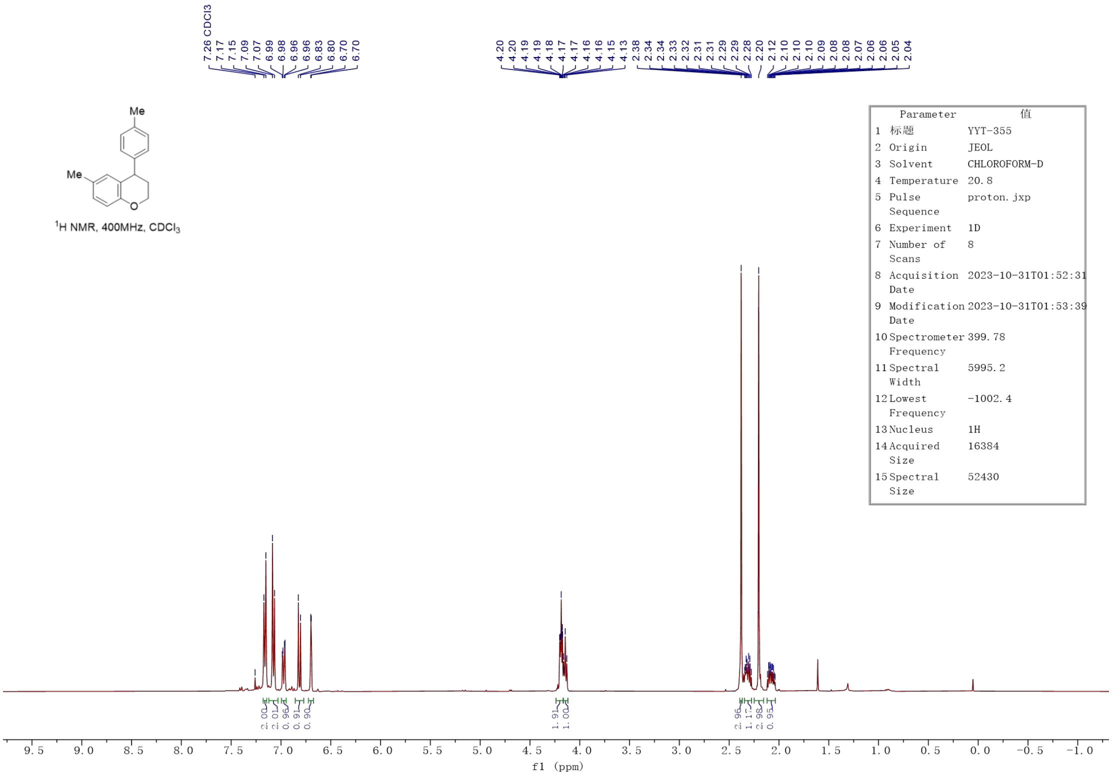

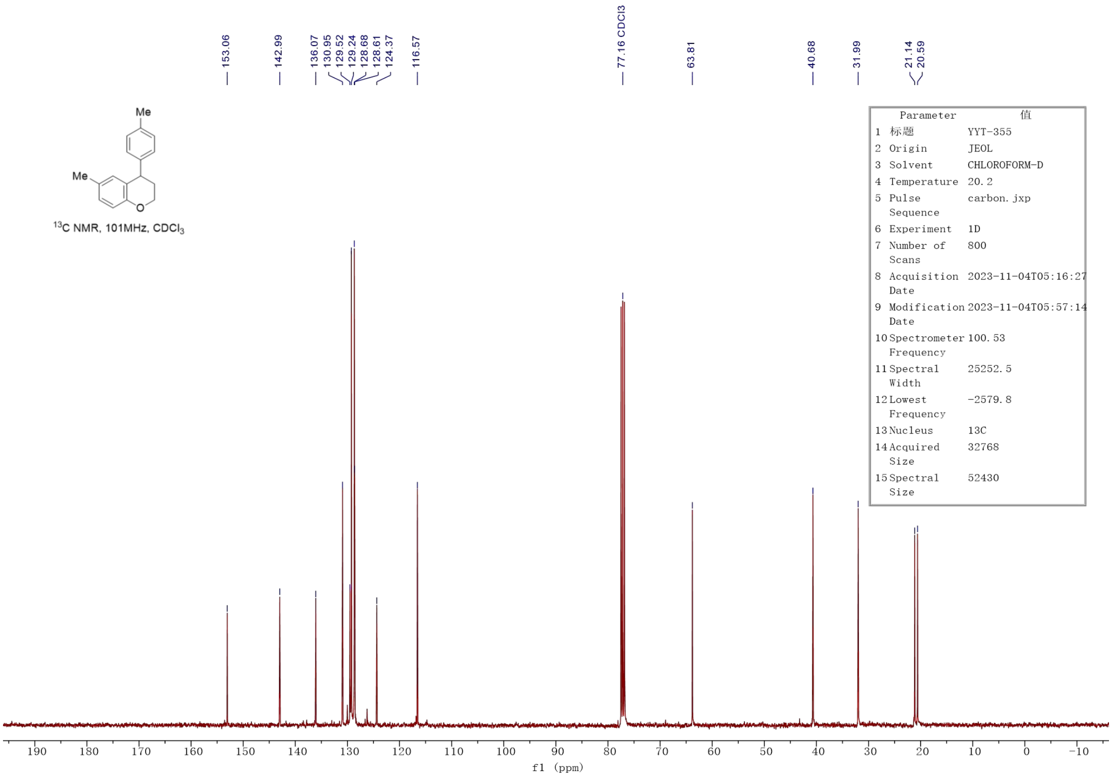

6-(sec-butyl)-4-(p-tolyl)chromane (70)

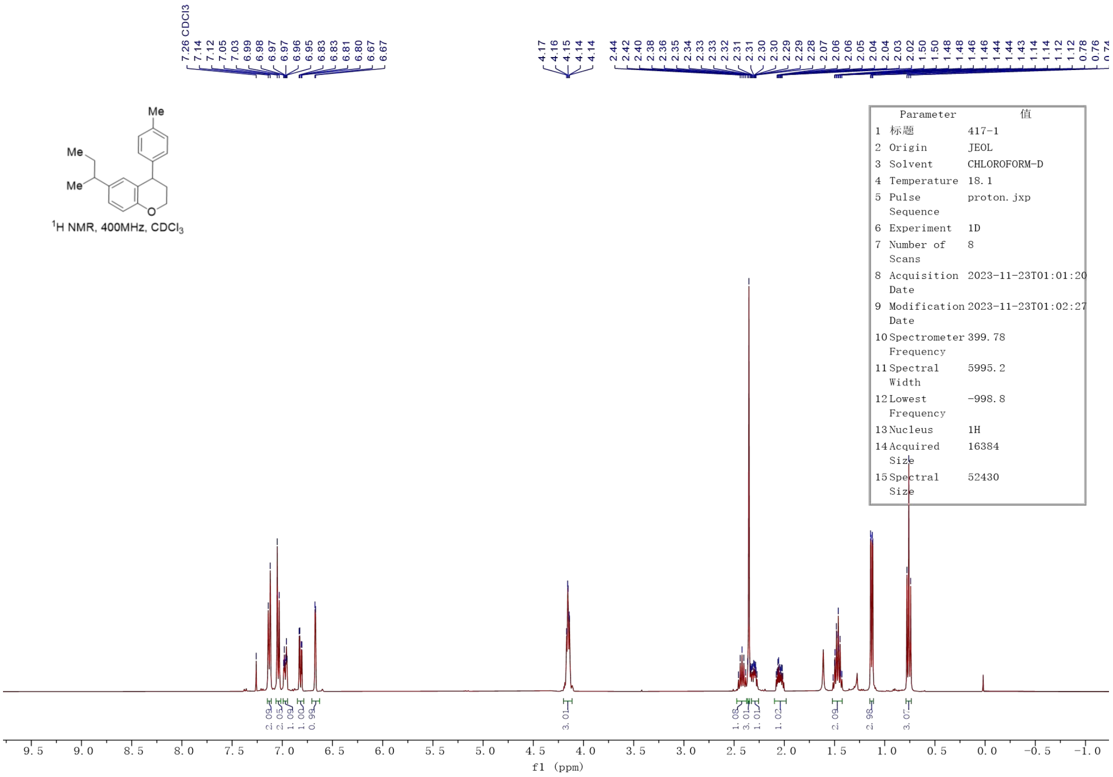

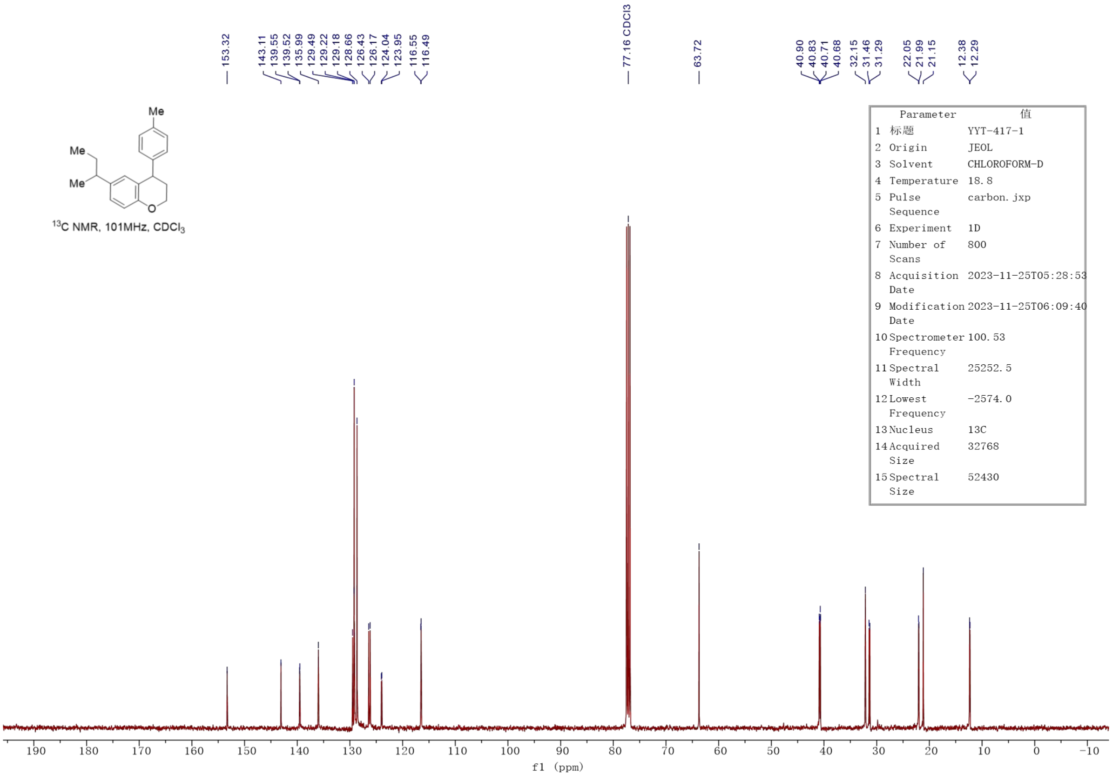

8-methyl-4-(p-tolyl)chromane (71)

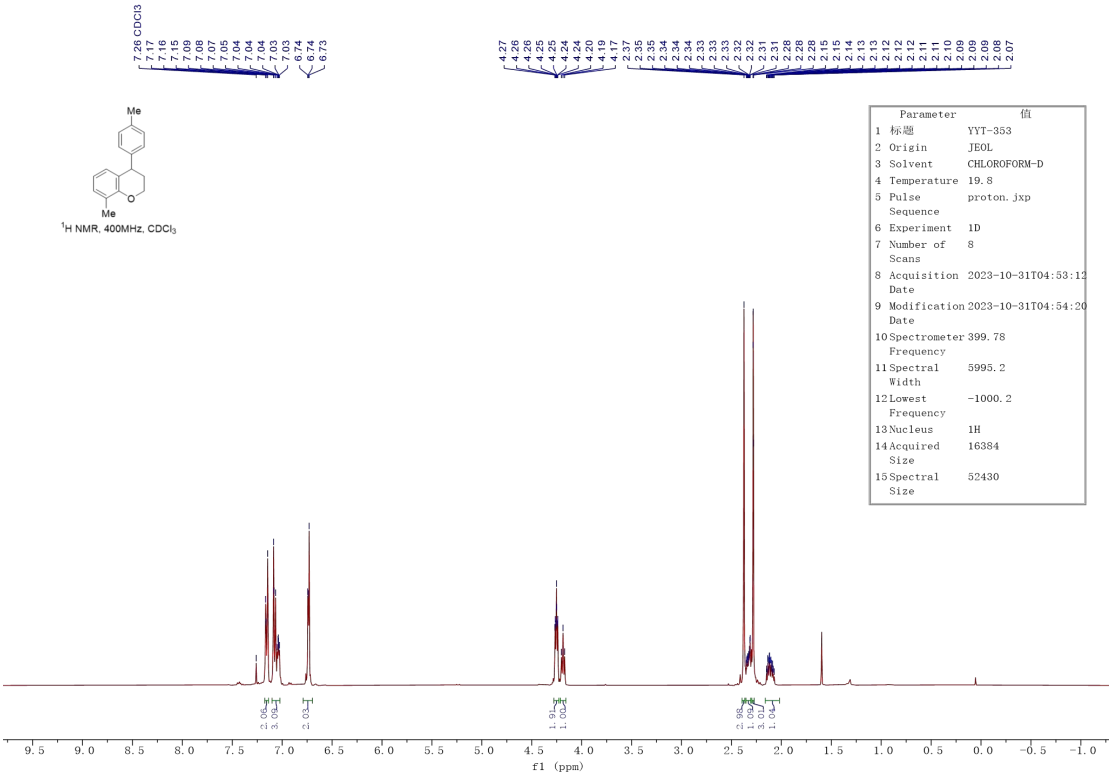

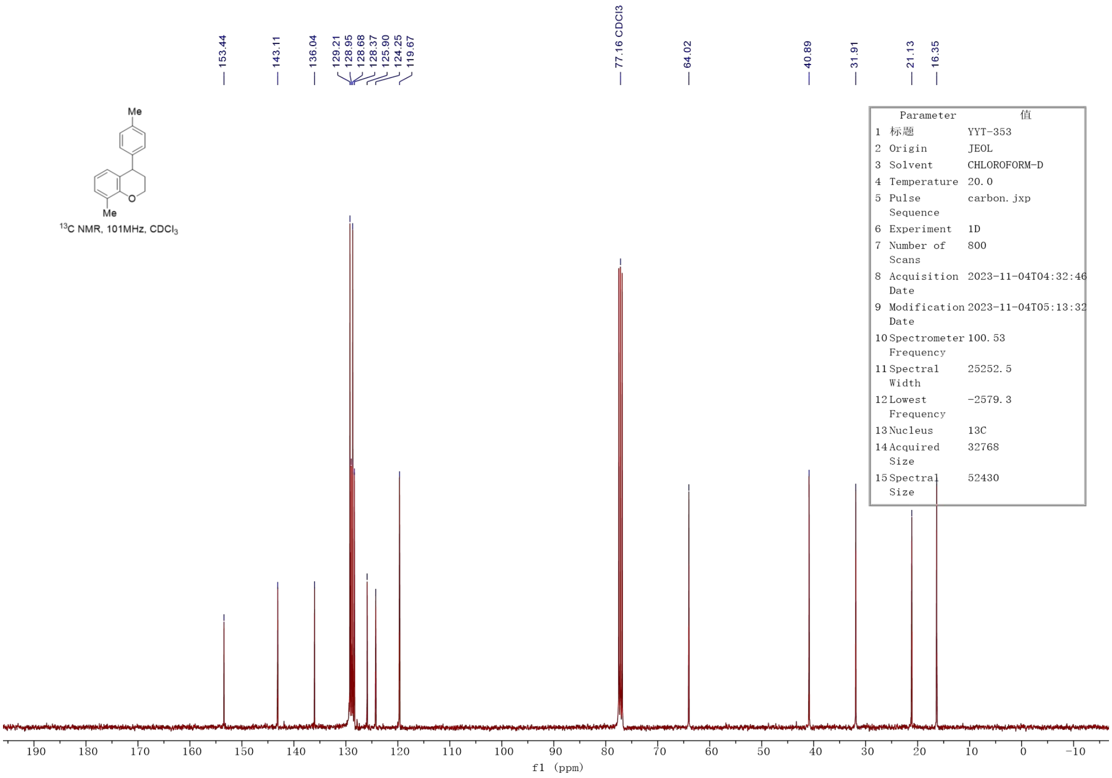

4-(4-methoxyphenyl)chromane-5,6,7,8-d$_4$ (72)

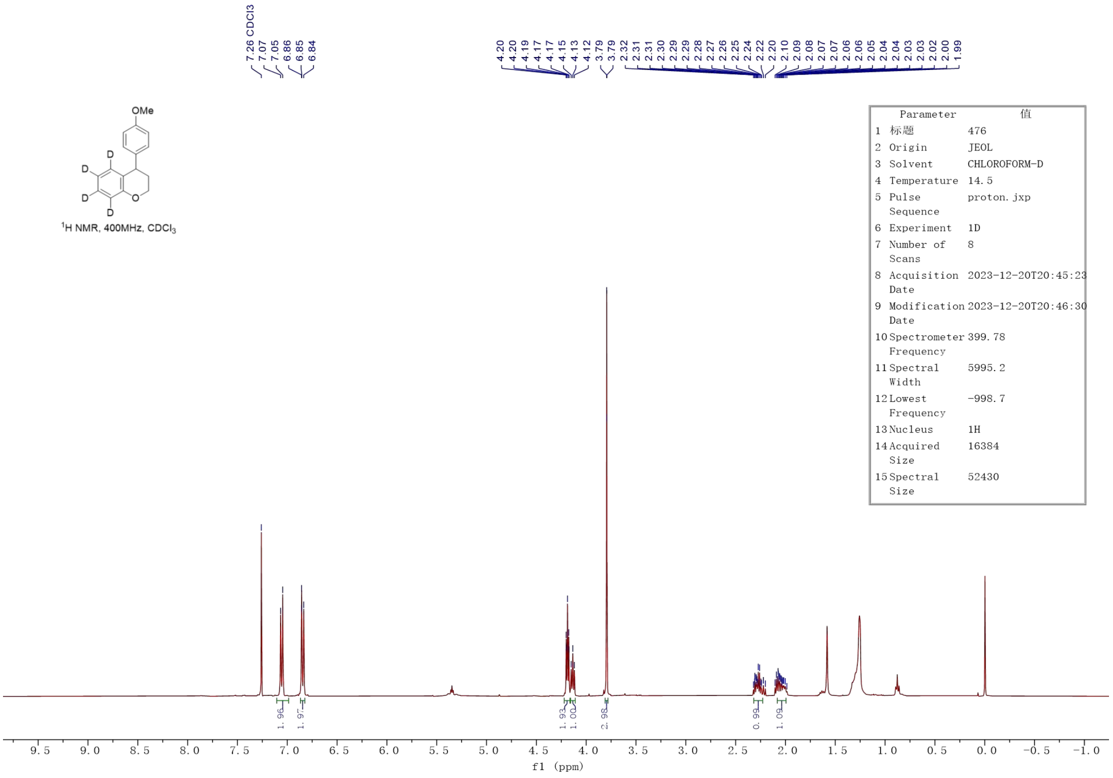

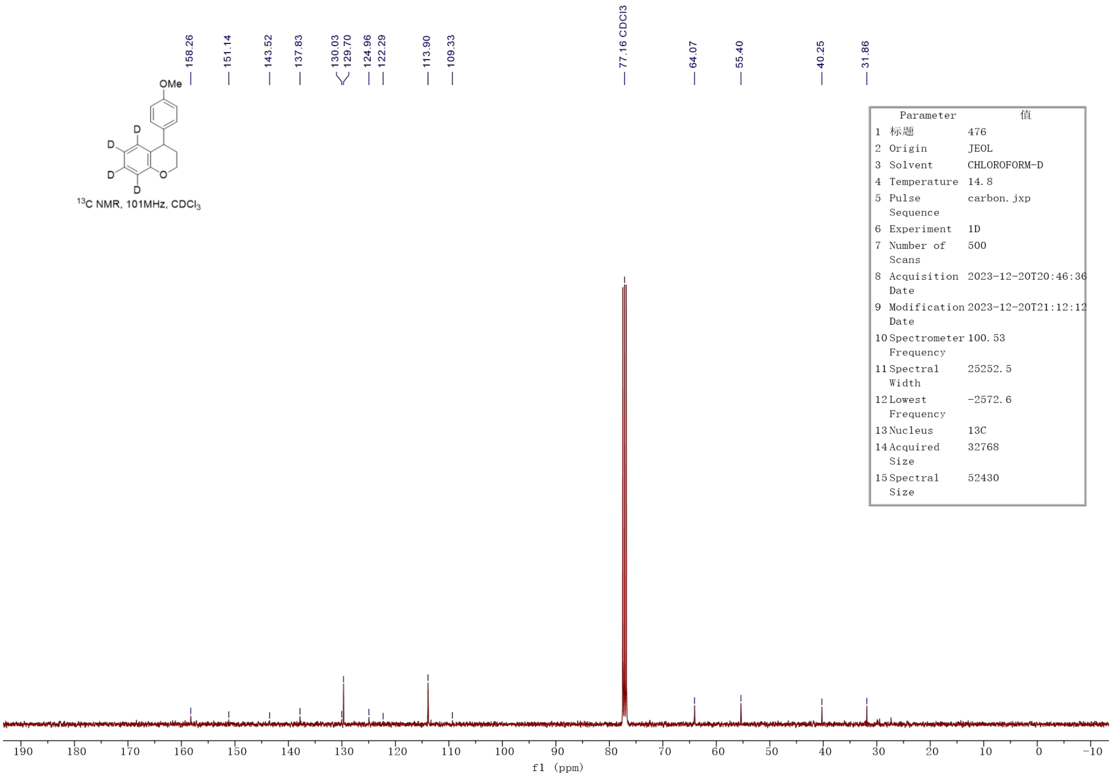

6-methoxy-4-(p-tolyl)chromane (73)

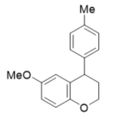

^{1}H NMR, 400MHz, $CDCl_3$

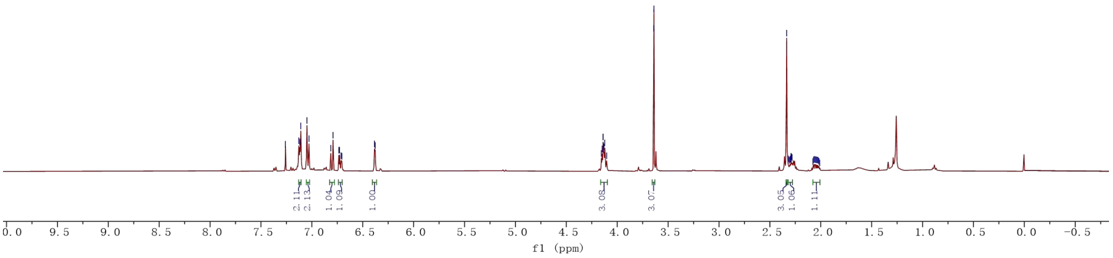

Parameter	Value
1 标题	YYT-413
2 Origin	JEOL
3 Solvent	CHLOROFORM-D
4 Temperature	18.4
5 Pulse Sequence	proton.jxp
6 Experiment	1D
7 Number of Scans	8
8 Acquisition Date	2023-11-26T06:18:19
9 Modification Date	2023-11-26T06:19:26
10 Spectrometer Frequency	399.78
11 Spectral Width	5995.2
12 Lowest Frequency	-998.9
13 Nucleus	1H
14 Acquired Size	16384
15 Spectral Size	52430

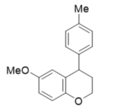

^{13}C NMR, 101MHz, $CDCl_3$

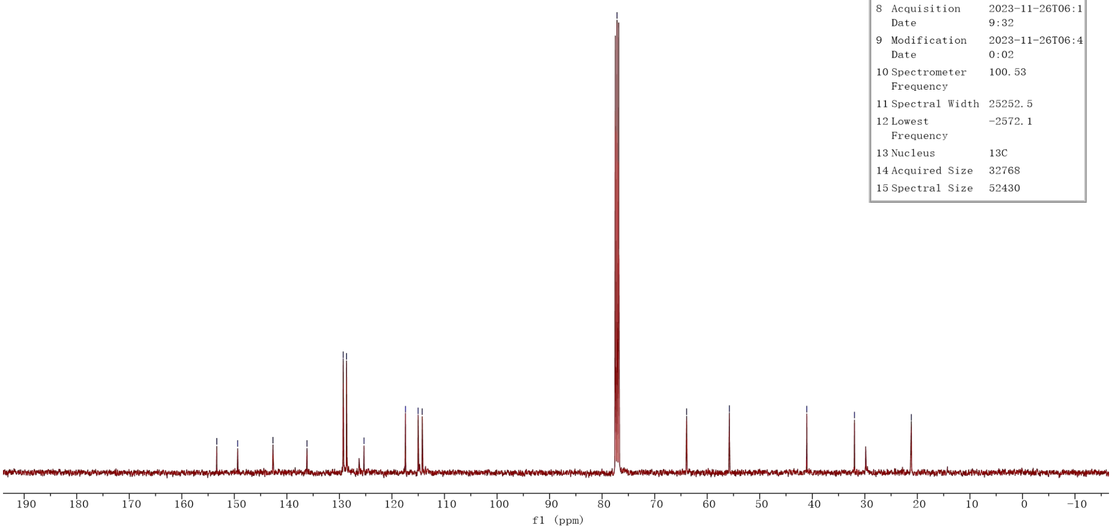

Parameter	值
1 标题	YYT-413
2 Origin	JEOL
3 Solvent	CHLOROFORM-D
4 Temperature	18.9
5 Pulse Sequence	carbon.jxp
6 Experiment	1D
7 Number of Scans	400
8 Acquisition Date	2023-11-26T06:19:32
9 Modification Date	2023-11-26T06:40:02
10 Spectrometer Frequency	100.53
11 Spectral Width	25252.5
12 Lowest Frequency	-2572.1
13 Nucleus	13C
14 Acquired Size	32768
15 Spectral Size	52430

6-ethoxy-4-(p-tolyl)chromane (74)

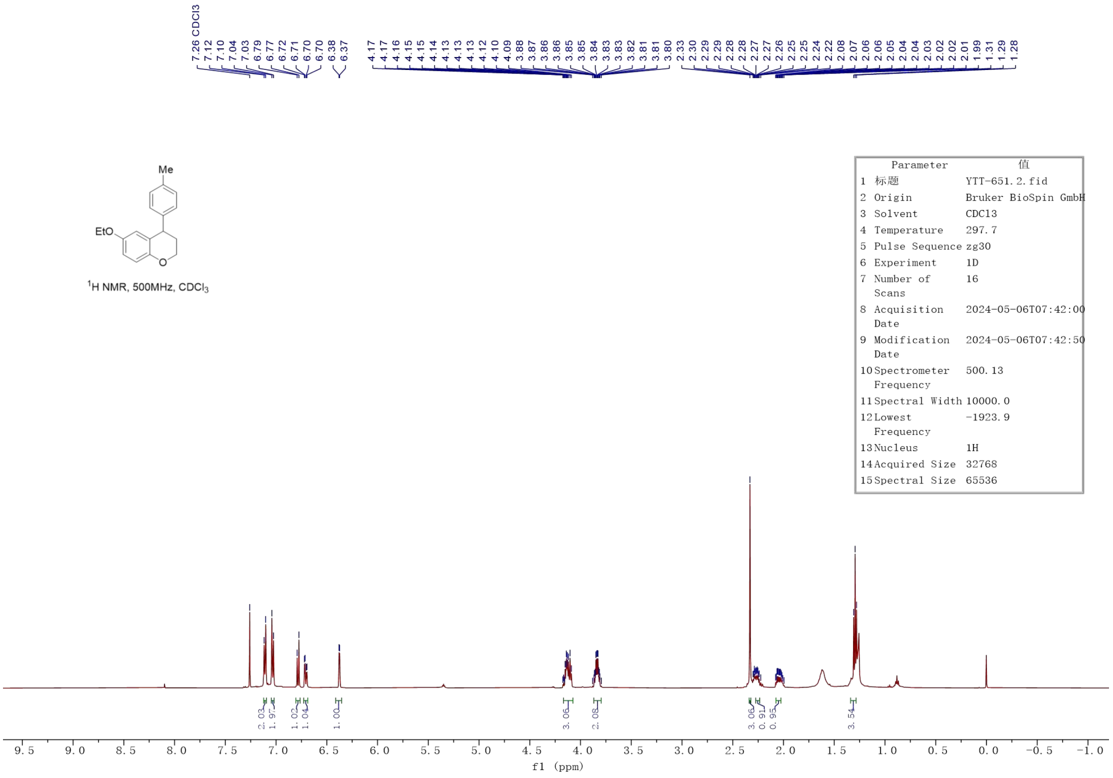

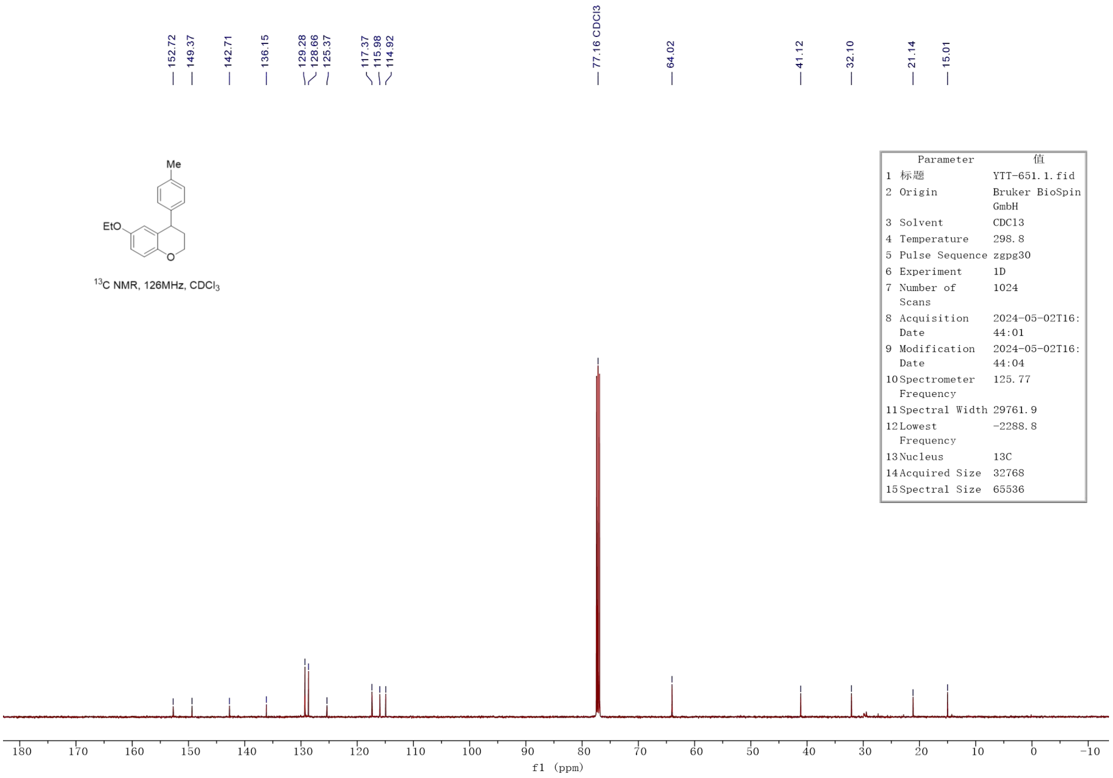

(R)-6-iodo-4-(p-tolyl)chromane (75)

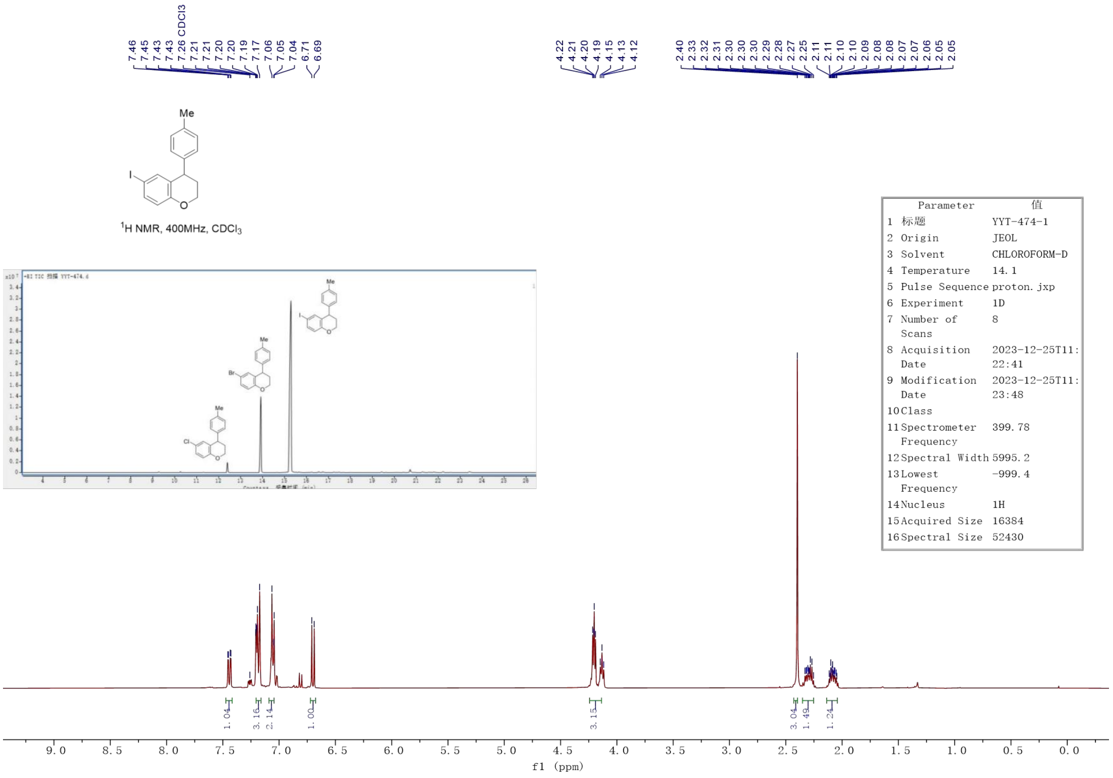

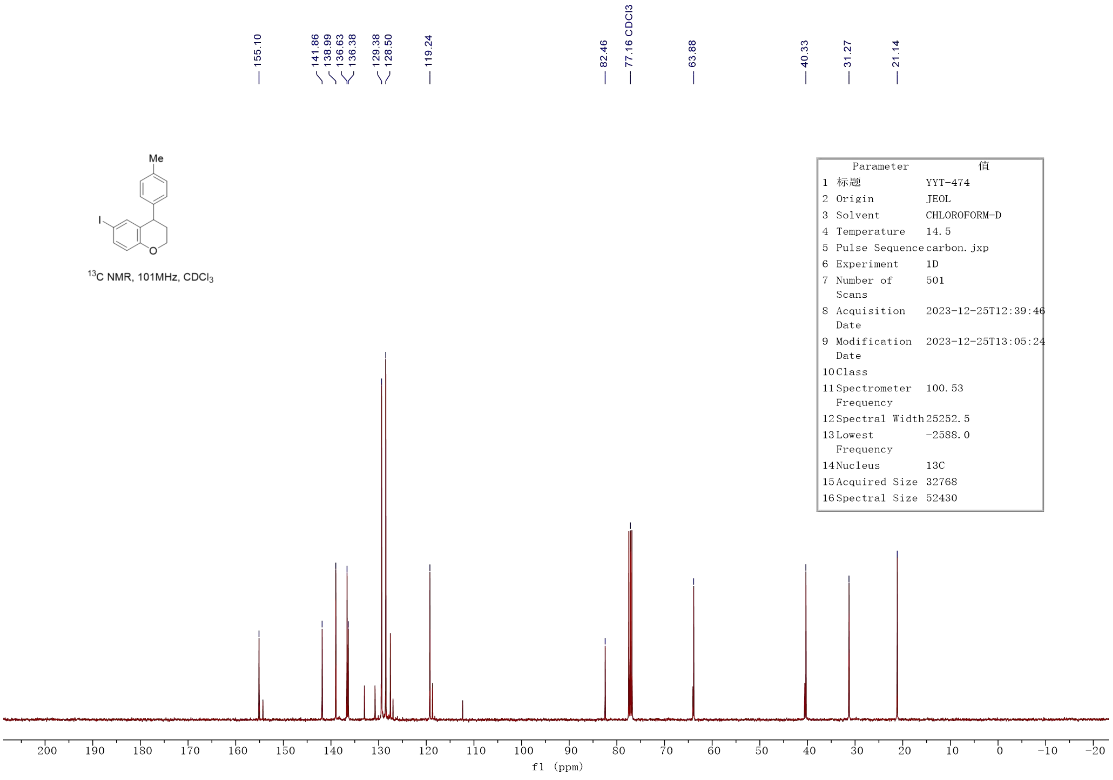

4-(p-tolyl)-6-(trifluoromethyl)chromane (76)

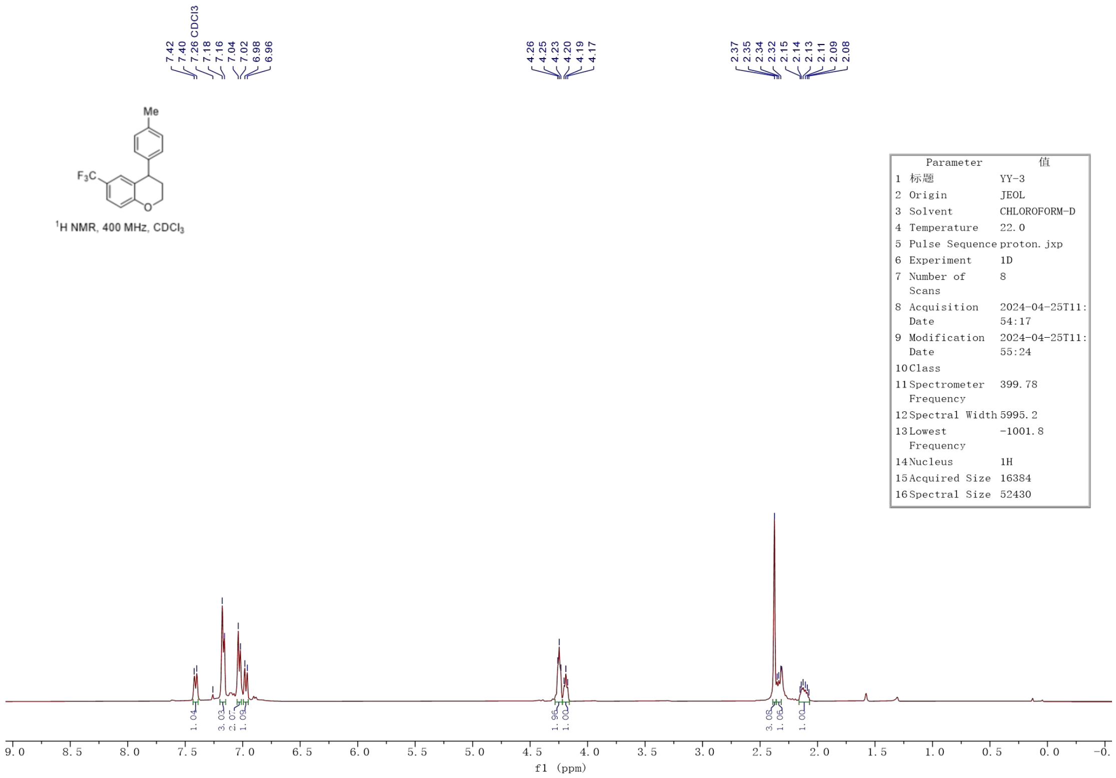

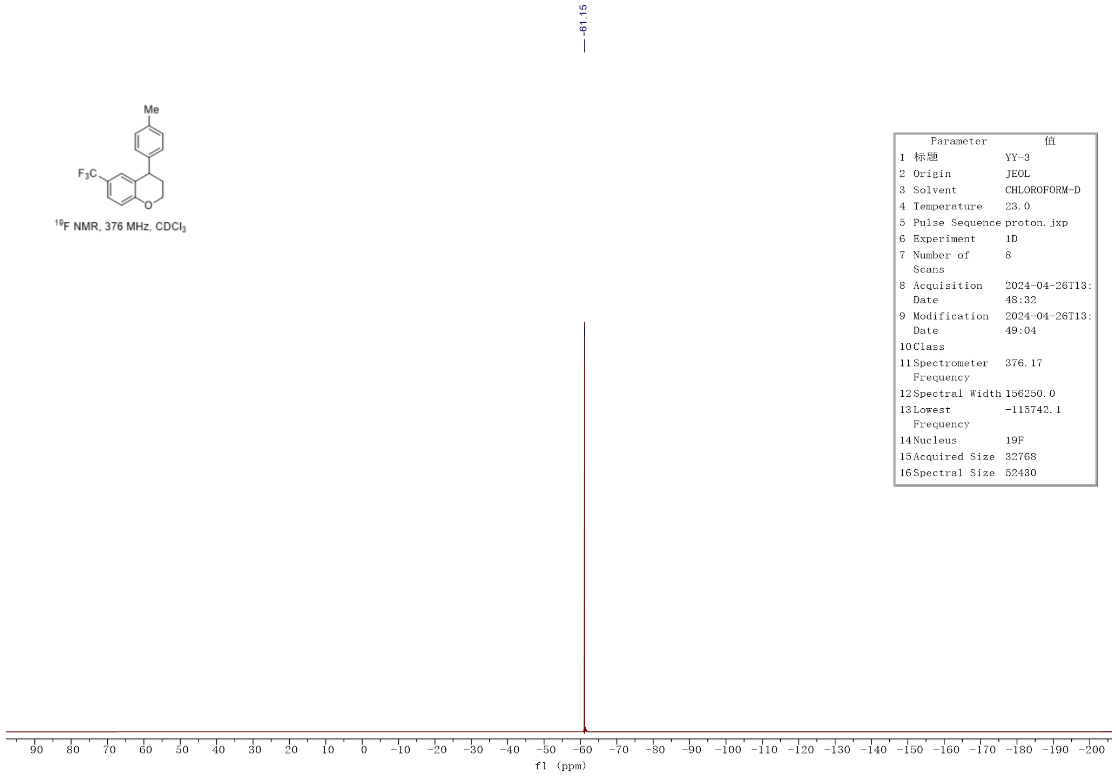

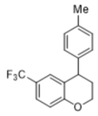

^{13}C NMR, 101 MHz, $CDCl_3$

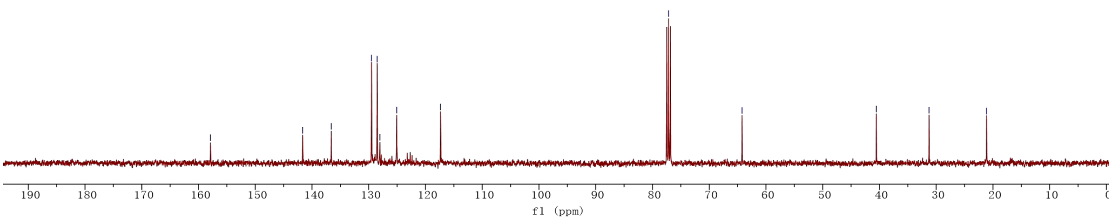

	Parameter	値
1	标题	YY-3
2	Origin	JEOL
3	Solvent	CHLOROFORM-D
4	Temperature	20.4
5	Pulse Sequence	carbon.jxp
6	Experiment	1D
7	Number of Scans	500
8	Acquisition Date	2024-05-20T07:11:16
9	Modification Date	2024-05-20T07:36:50
10	Class	
11	Spectrometer Frequency	100.53
12	Spectral Width	25252.5
13	Lowest Frequency	-2576.4
14	Nucleus	13C
15	Acquired Size	32768
16	Spectral Size	52430

3-methyl-4-(*p*-tolyl)chromane (77)

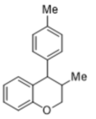

^{1}H NMR, 400 MHz, $CDCl_3$

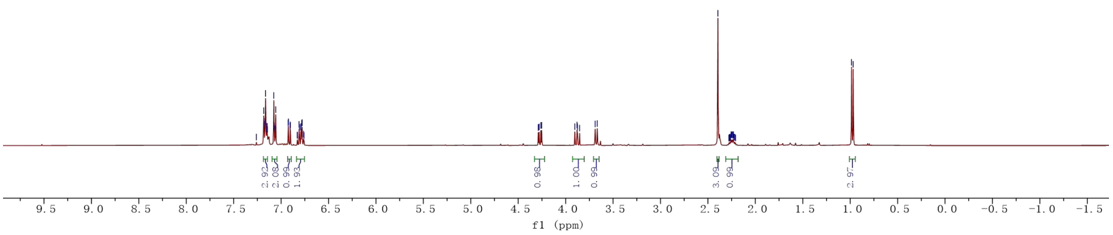

Parameter		值
1	标题	YYT-672-1-2
2	Origin	JEOL
3	Solvent	CHLOROFORM-D
4	Temperature	20.2
5	Pulse Sequence	proton.jxp
6	Experiment	1D
7	Number of Scans	8
8	Acquisition Date	2024-05-05T13:15:08
9	Modification Date	2024-05-05T13:16:15
10	Class	
11	Spectrometer Frequency	399.78
12	Spectral Width	5995.2
13	Lowest Frequency	-1002.0
14	Nucleus	1H
15	Acquired Size	16384
16	Spectral Size	52430

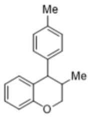

^{13}C NMR, 101 MHz, $CDCl_3$

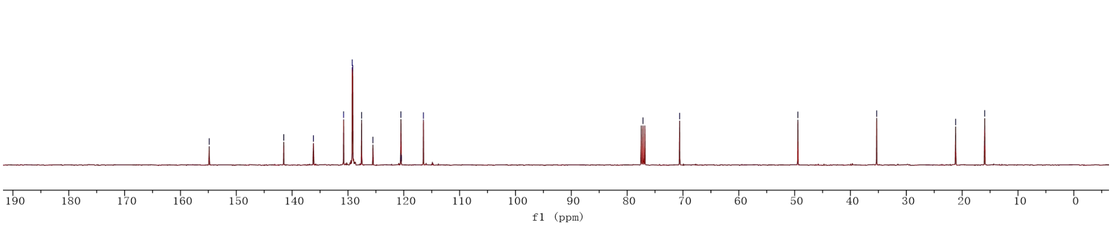

Parameter		值
1	标题	YYT-672-1-2 1
2	Origin	JEOL
3	Solvent	CHLOROFORM-D
4	Temperature	20.6
5	Pulse Sequence	carbon.jxp
6	Experiment	1D
7	Number of Scans	300
8	Acquisition Date	2024-05-05T13:24:06
9	Modification Date	2024-05-05T13:39:33
10	Class	
11	Spectrometer Frequency	100.53
12	Spectral Width	25252.5
13	Lowest Frequency	-2581.0
14	Nucleus	13C
15	Acquired Size	32768
16	Spectral Size	52430

4-(p-tolyl)-3,4,6,7,8,9-hexahydro-2H-benzo[g]chromene (78)

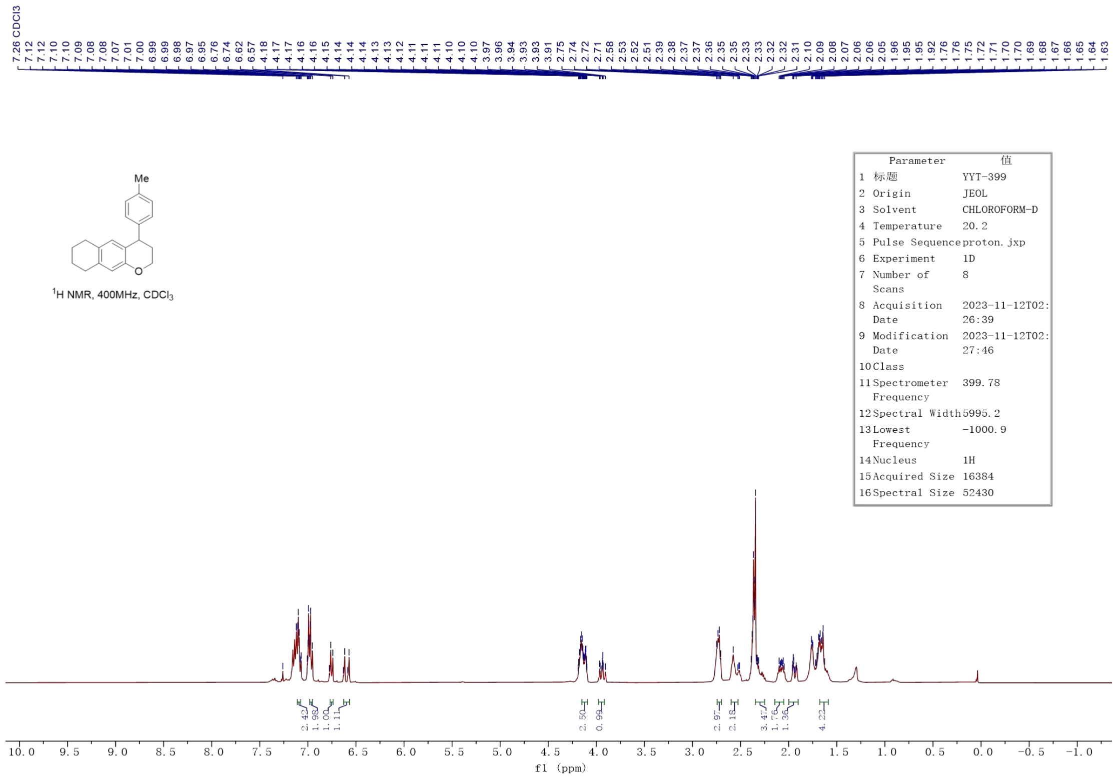

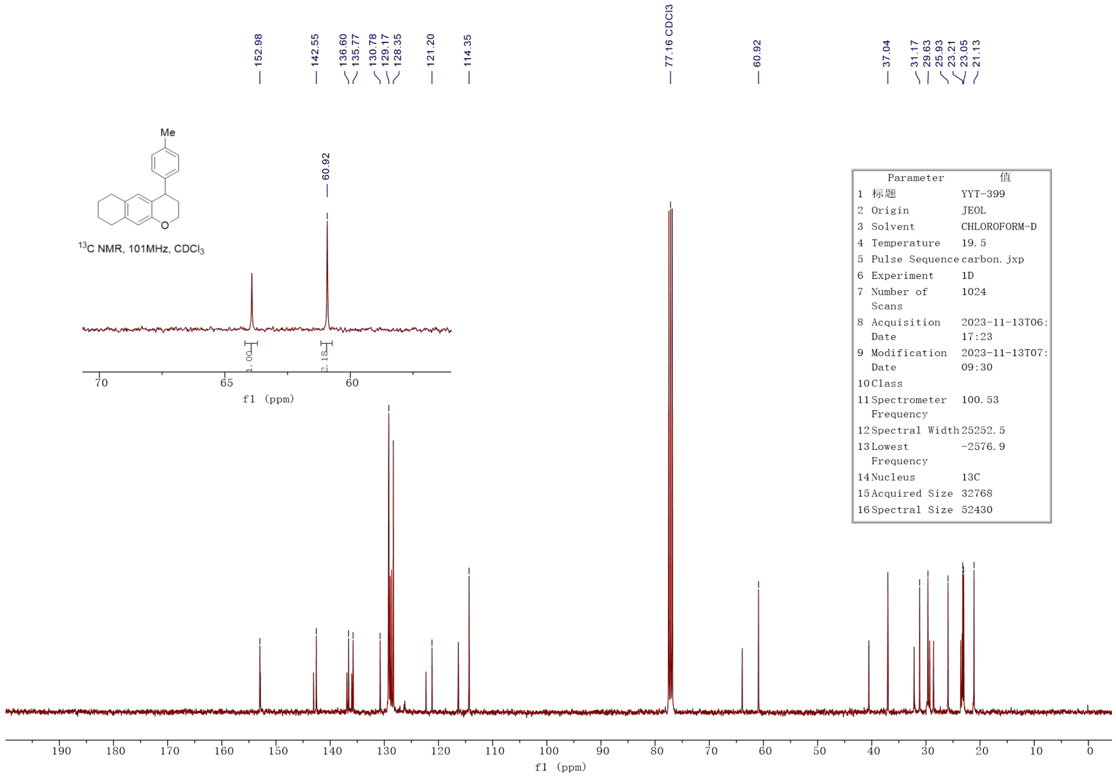

4-(p-tolyl)-3,4,7,8,9,10-hexahydro-2H-benzo[h]chromene (79)

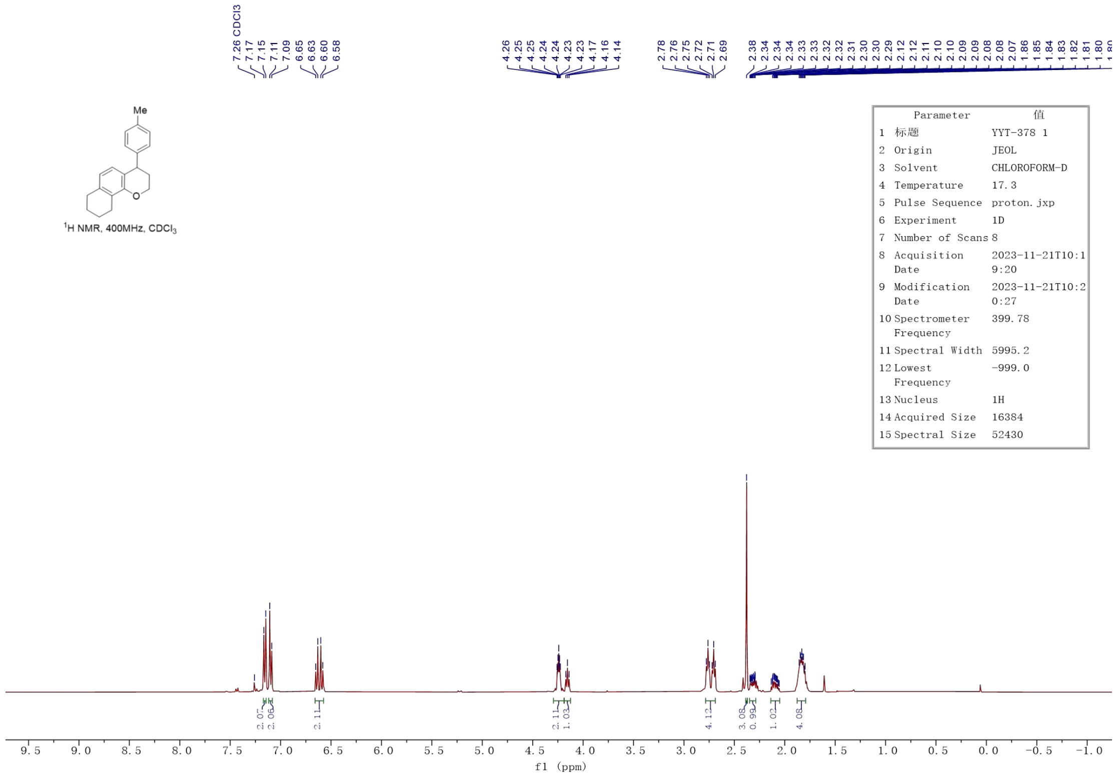

	Parameter	值
1	标题	YYT-378 1
2	Origin	JEOL
3	Solvent	CHLOROFORM-D
4	Temperature	17.3
5	Pulse Sequence	proton.jxp
6	Experiment	1D
7	Number of Scans	8
8	Acquisition Date	2023-11-21T10:19:20
9	Modification Date	2023-11-21T10:20:27
10	Spectrometer Frequency	399.78
11	Spectral Width	5995.2
12	Lowest Frequency	-999.0
13	Nucleus	1H
14	Acquired Size	16384
15	Spectral Size	52430

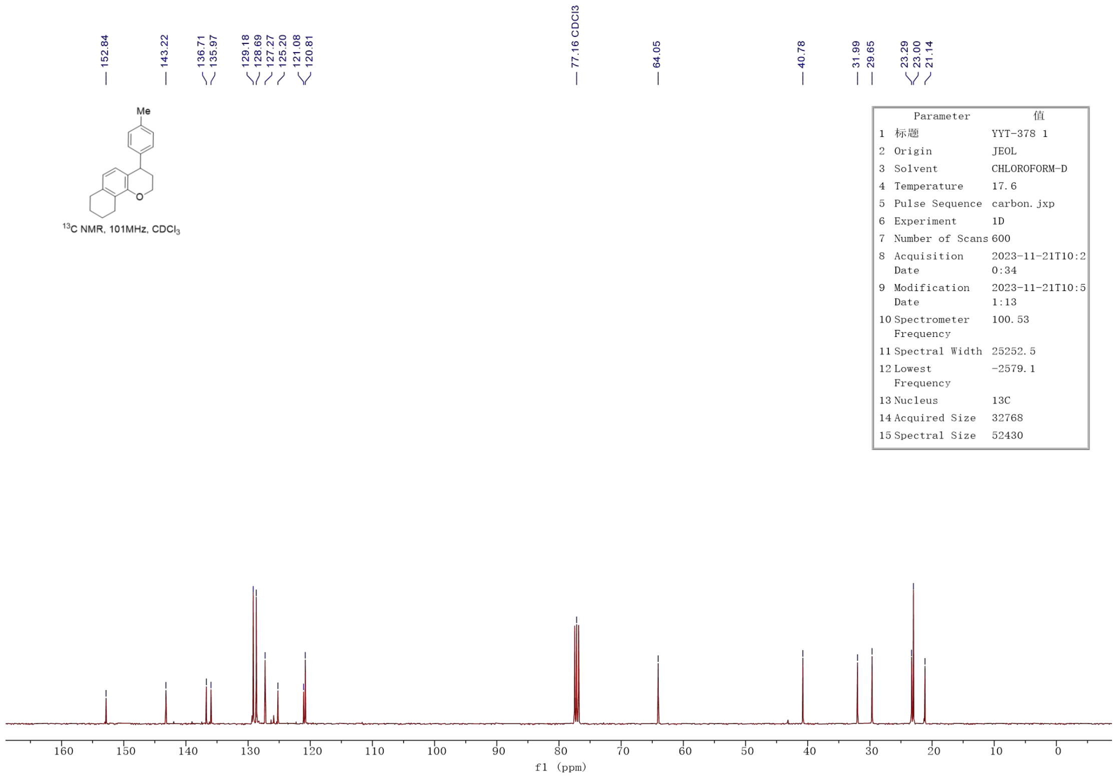

	Parameter	值
1	标题	YYT-378 1
2	Origin	JEOL
3	Solvent	CHLOROFORM-D
4	Temperature	17.6
5	Pulse Sequence	carbon.jxp
6	Experiment	1D
7	Number of Scans	600
8	Acquisition Date	2023-11-21T10:20:34
9	Modification Date	2023-11-21T10:51:13
10	Spectrometer Frequency	100.53
11	Spectral Width	25252.5
12	Lowest Frequency	-2579.1
13	Nucleus	13C
14	Acquired Size	32768
15	Spectral Size	52430

12-(p-tolyl)-11,12-dihydro-10H-phenanthro[4,5-fgh]chromene (80)

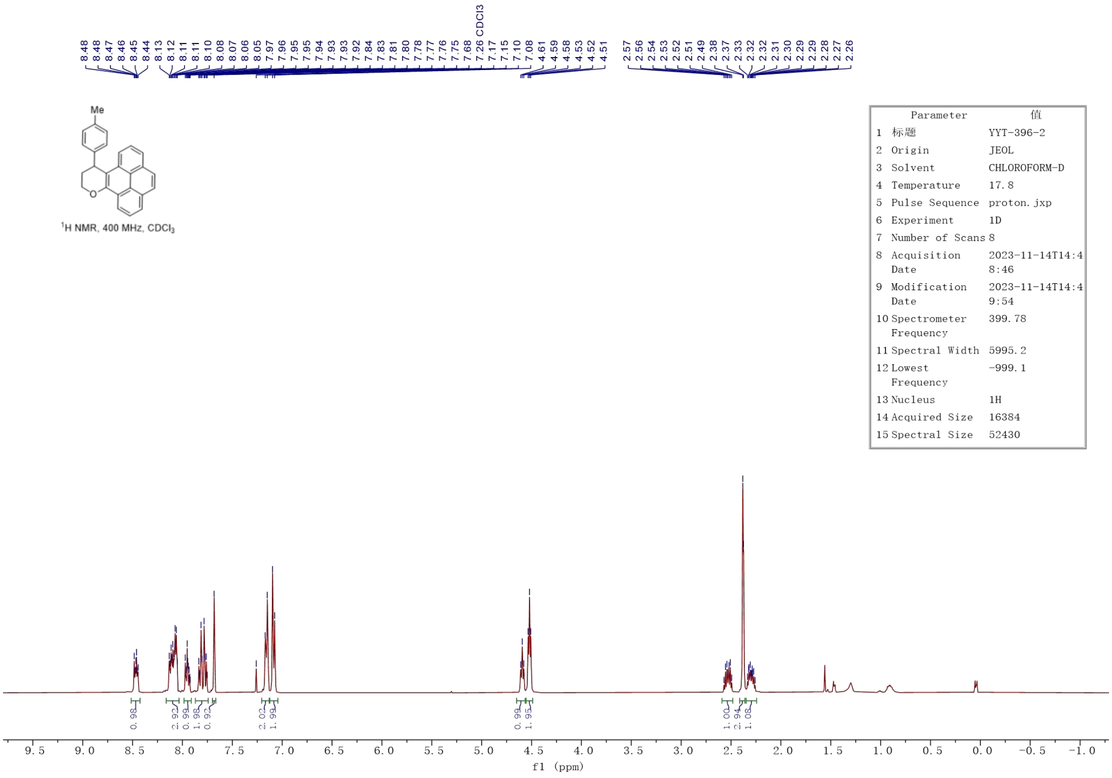

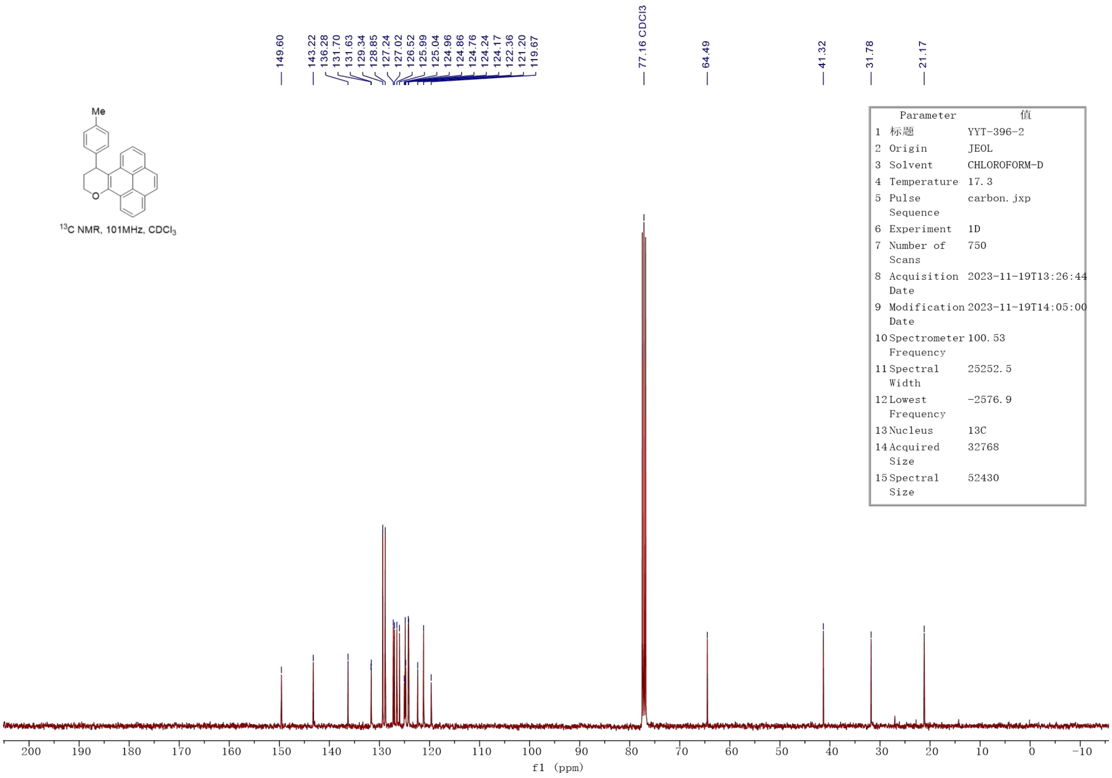

6-(p-tolyl)-7,8-dihydro-2H,6H-pyrano[3,2-g]chromen-2-one (81)

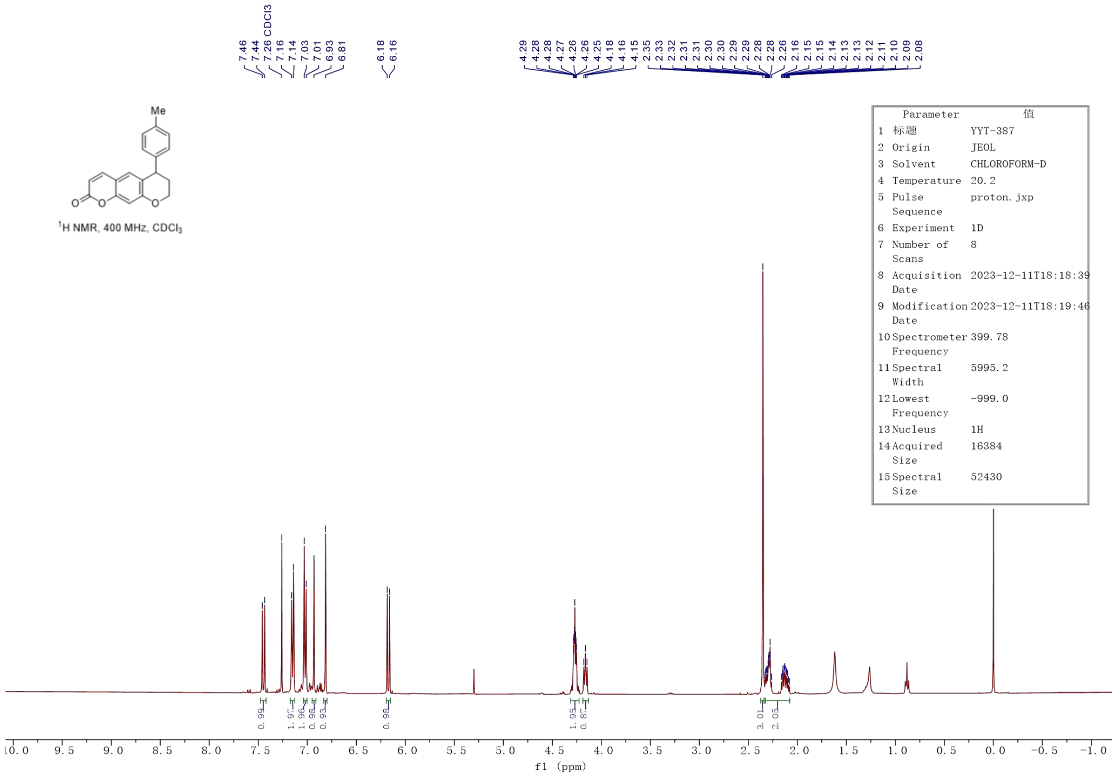

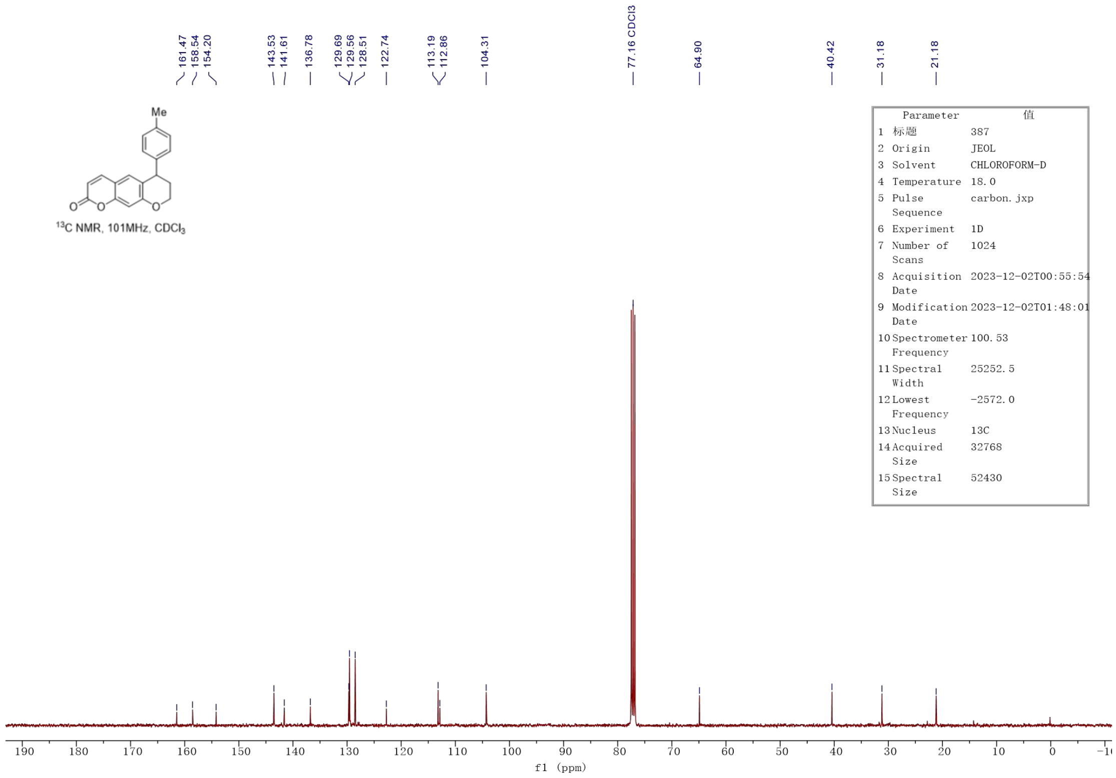

6-(p-tolyl)-4-(trifluoromethyl)-7,8-dihydro-2H,6H-pyrano[3,2-g]chromen-2-one (82)

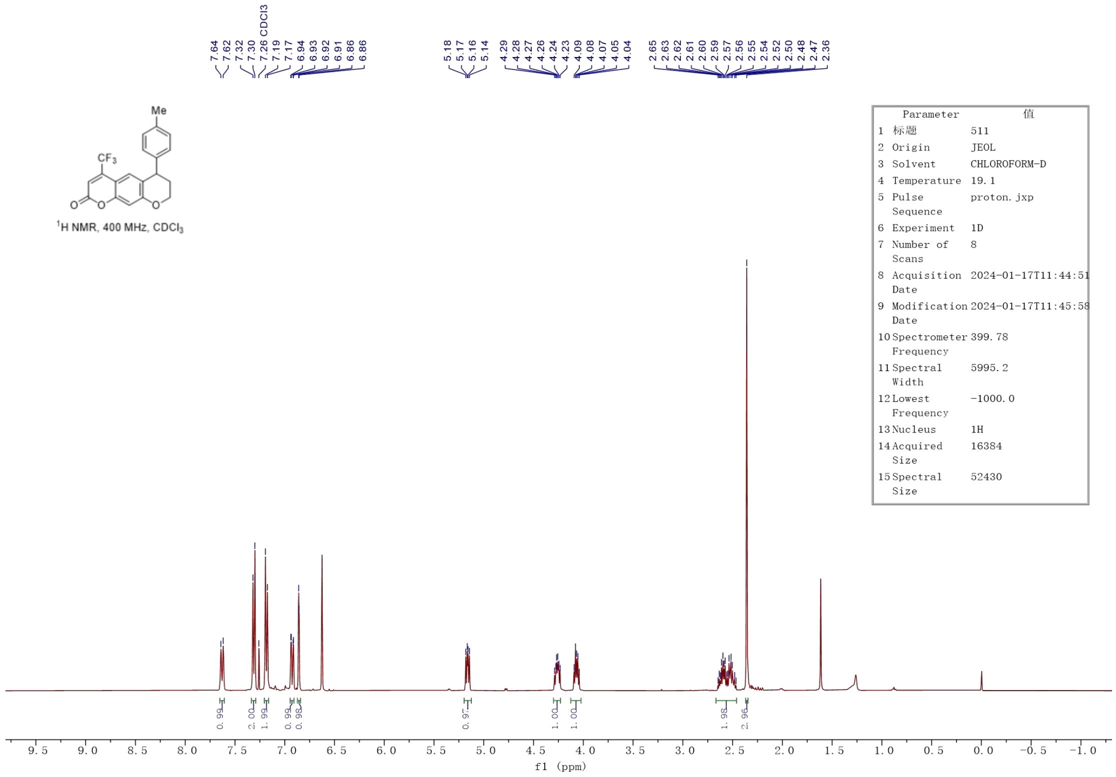

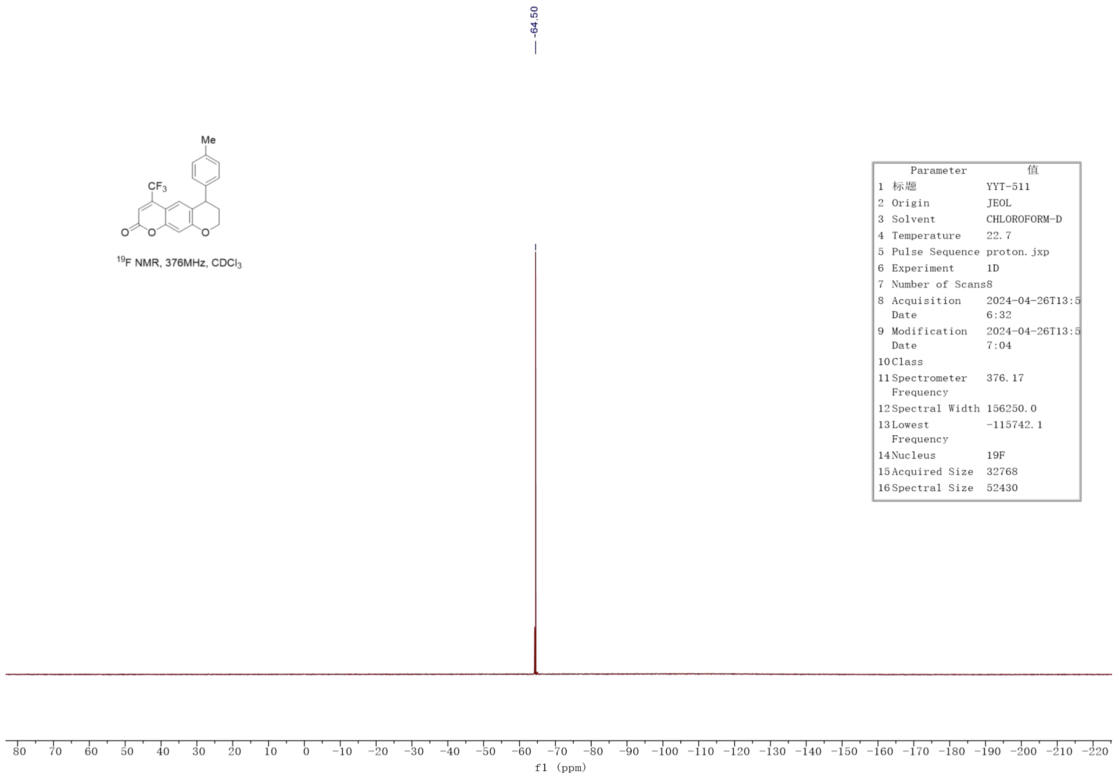

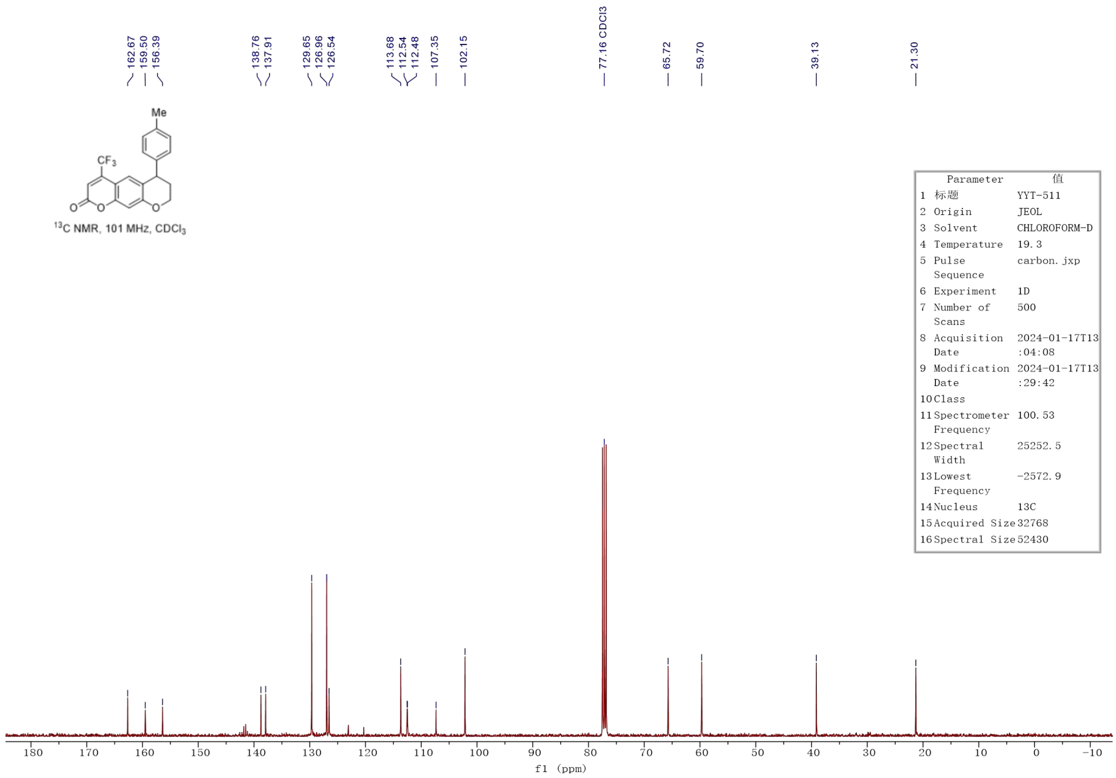

	Parameter	值
1	标题	YYT-511
2	Origin	JEOL
3	Solvent	CHLOROFORM-D
4	Temperature	19.3
5	Pulse Sequence	carbon.jxp
6	Experiment	1D
7	Number of Scans	500
8	Acquisition Date	2024-01-17T13:04:08
9	Modification Date	2024-01-17T13:29:42
10	Class	
11	Spectrometer Frequency	100.53
12	Spectral Width	25252.5
13	Lowest Frequency	-2572.9
14	Nucleus	13C
15	Acquired Size	32768
16	Spectral Size	52430

4-(*p*-tolyl)-3,4-dihydro-2*H*-benzofuro[2,3-*g*]chromene (83)

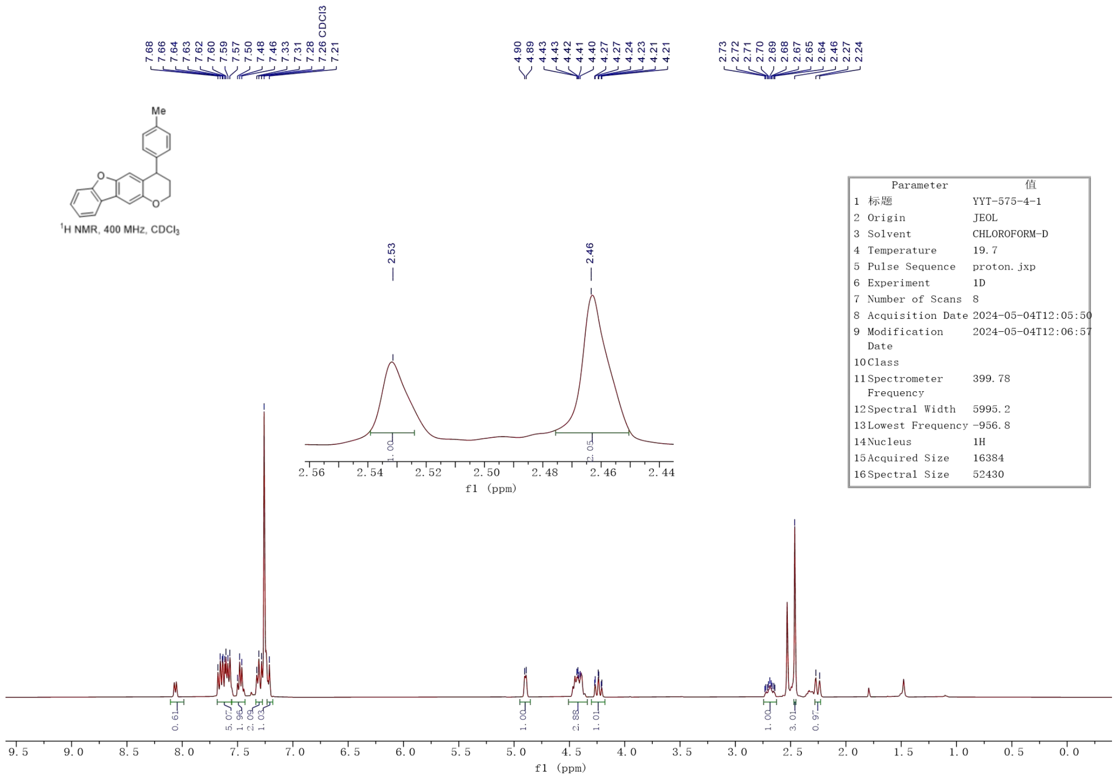

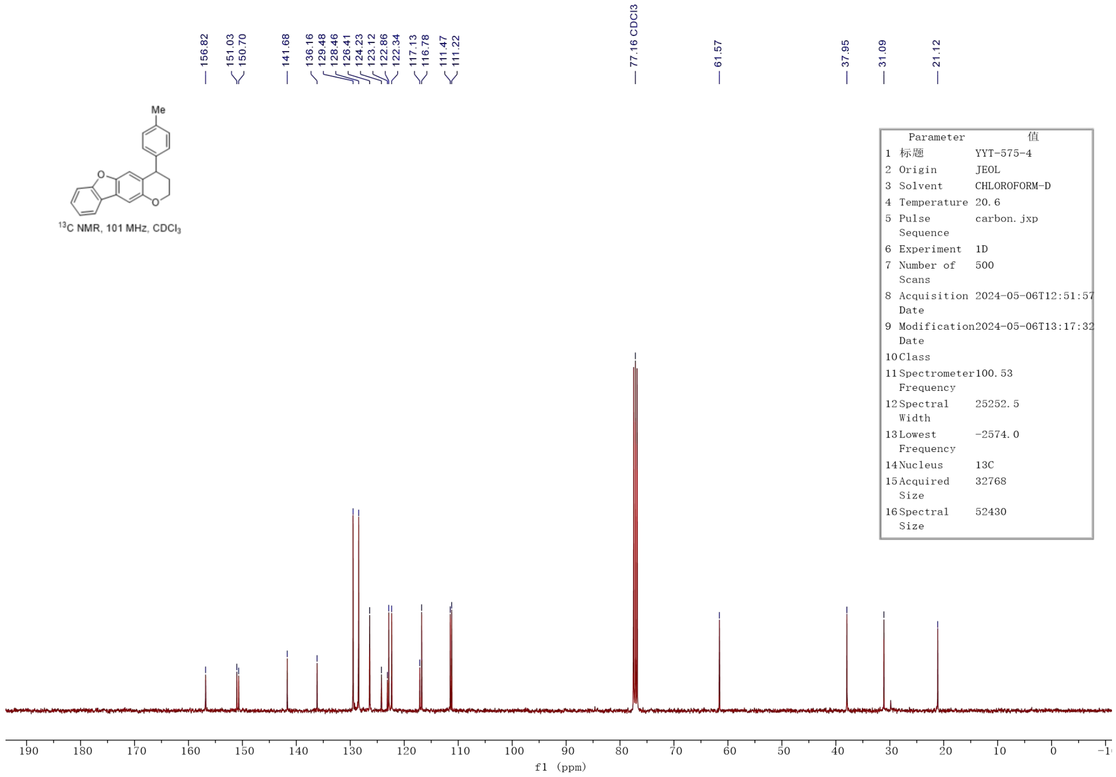

8-(p-tolyl)-7,8-dihydro-6H-[1,3]dioxolo[4,5-g]chromene (84)

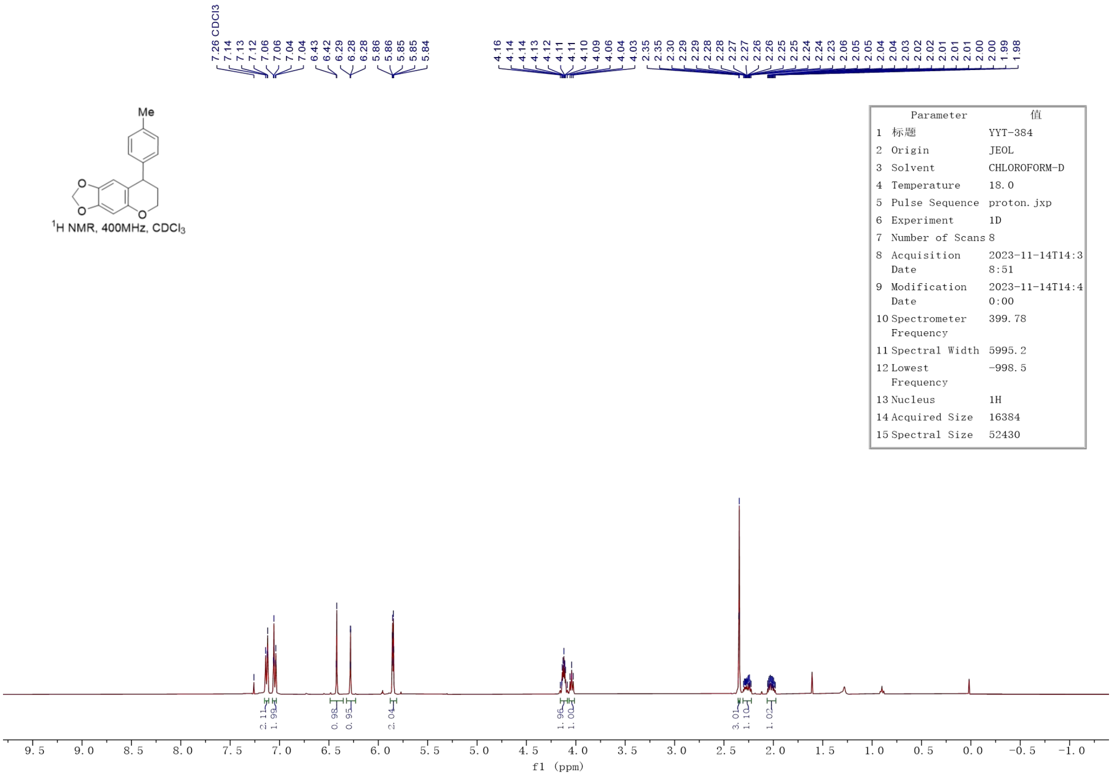

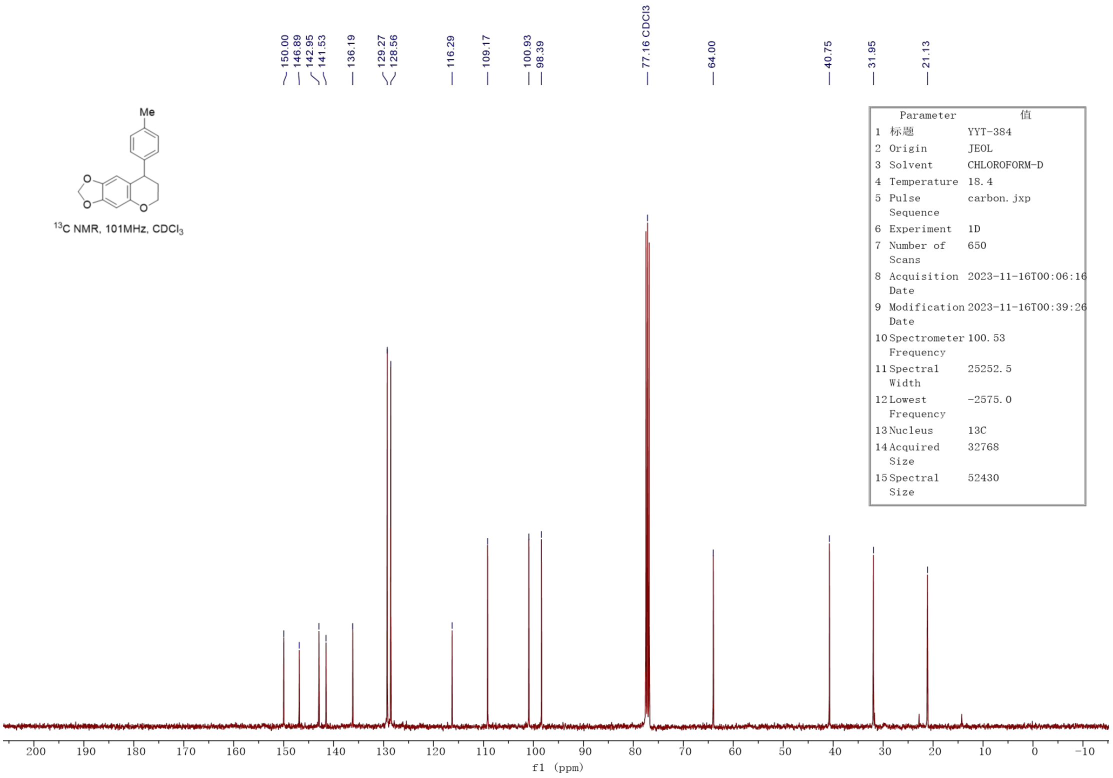

5,6,7-trimethoxy-4-(p-tolyl)chromane (85)

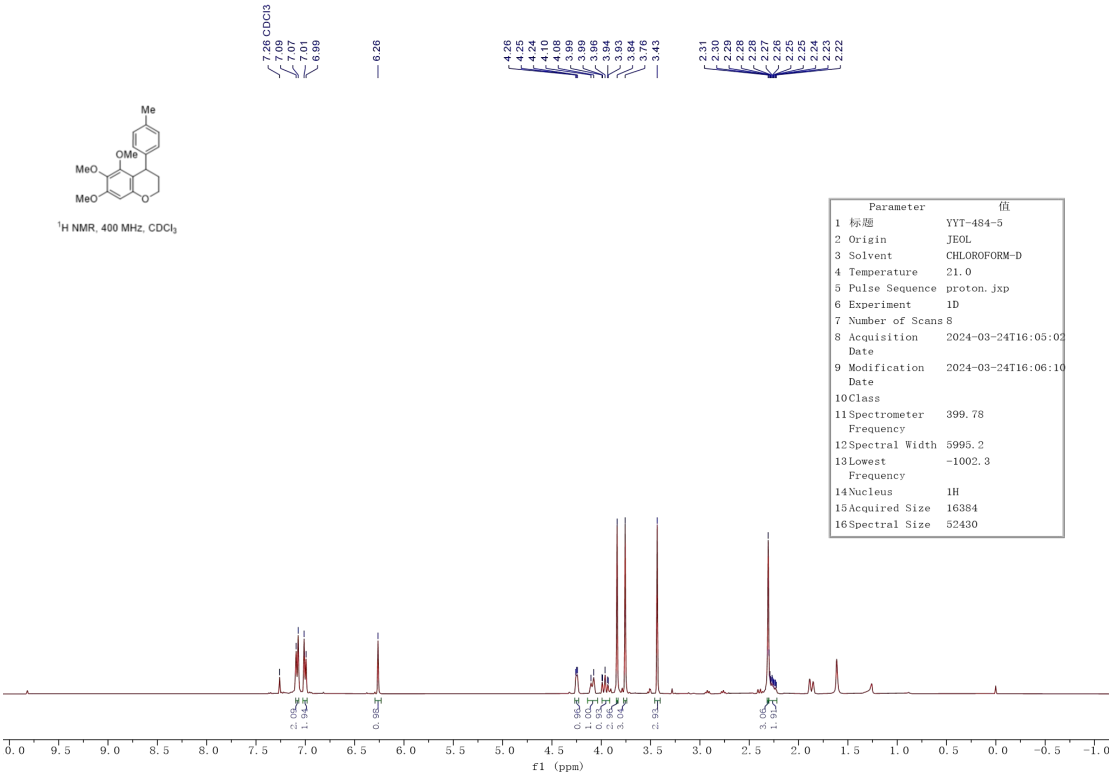

Parameter	值
1 标题	YYT-484-5
2 Origin	JEOL
3 Solvent	CHLOROFORM-D
4 Temperature	21.0
5 Pulse Sequence	proton.jxp
6 Experiment	1D
7 Number of Scans	8
8 Acquisition Date	2024-03-24T16:05:02
9 Modification Date	2024-03-24T16:06:10
10 Class	
11 Spectrometer Frequency	399.78
12 Spectral Width	5995.2
13 Lowest Frequency	-1002.3
14 Nucleus	1H
15 Acquired Size	16384
16 Spectral Size	52430

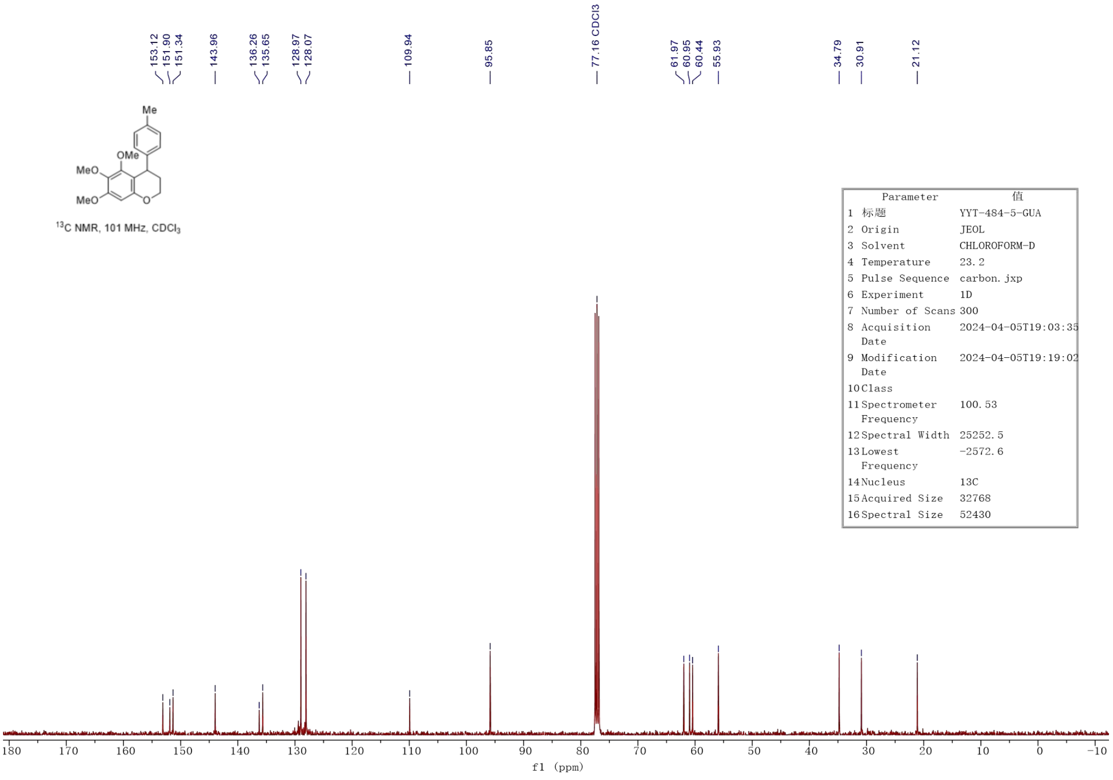

Parameter	值
1 标题	YYT-484-5-GUA
2 Origin	JEOL
3 Solvent	CHLOROFORM-D
4 Temperature	23.2
5 Pulse Sequence	carbon.jxp
6 Experiment	1D
7 Number of Scans	300
8 Acquisition Date	2024-04-05T19:03:35
9 Modification Date	2024-04-05T19:19:02
10 Class	
11 Spectrometer Frequency	100.53
12 Spectral Width	25252.5
13 Lowest Frequency	-2572.6
14 Nucleus	13C
15 Acquired Size	32768
16 Spectral Size	52430

11-(p-tolyl)-10,11-dihydro-5H,9H-benzo[c]pyrano[3,2-g]chromen-5-one (86)

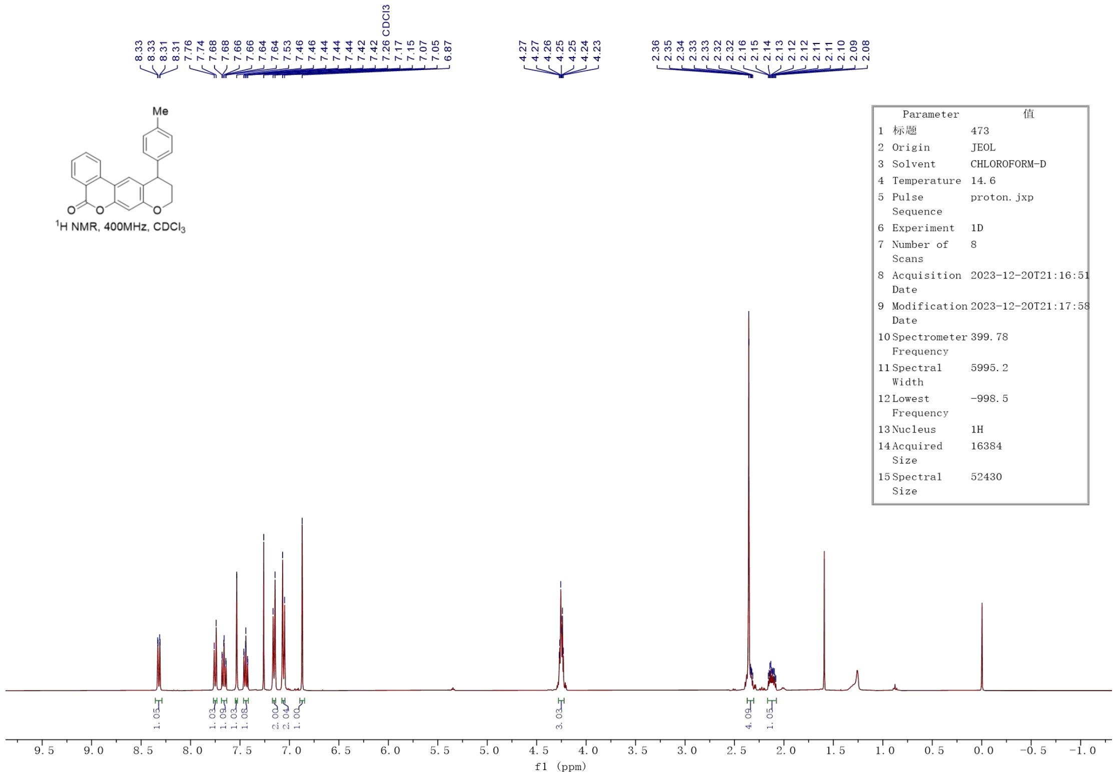

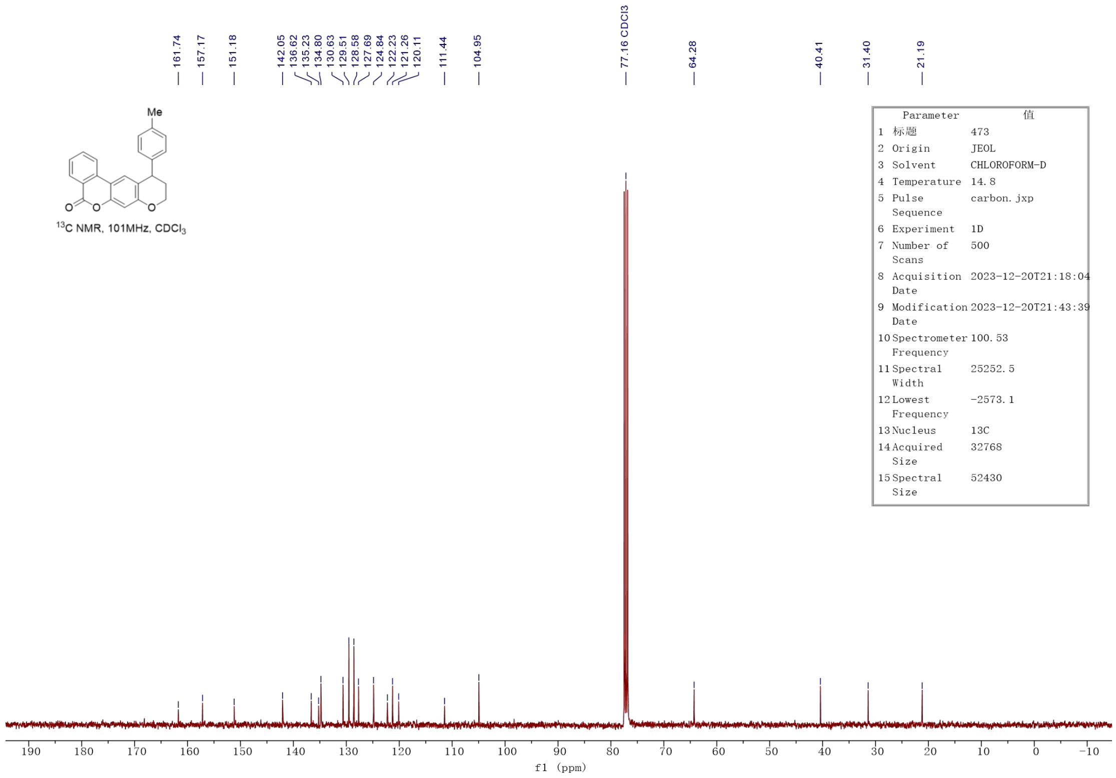

3-(4-methoxyphenyl)-10-(p-tolyl)-9,10-dihydro-4H,8H-pyrano[2,3-f]chromen-4-one (87)

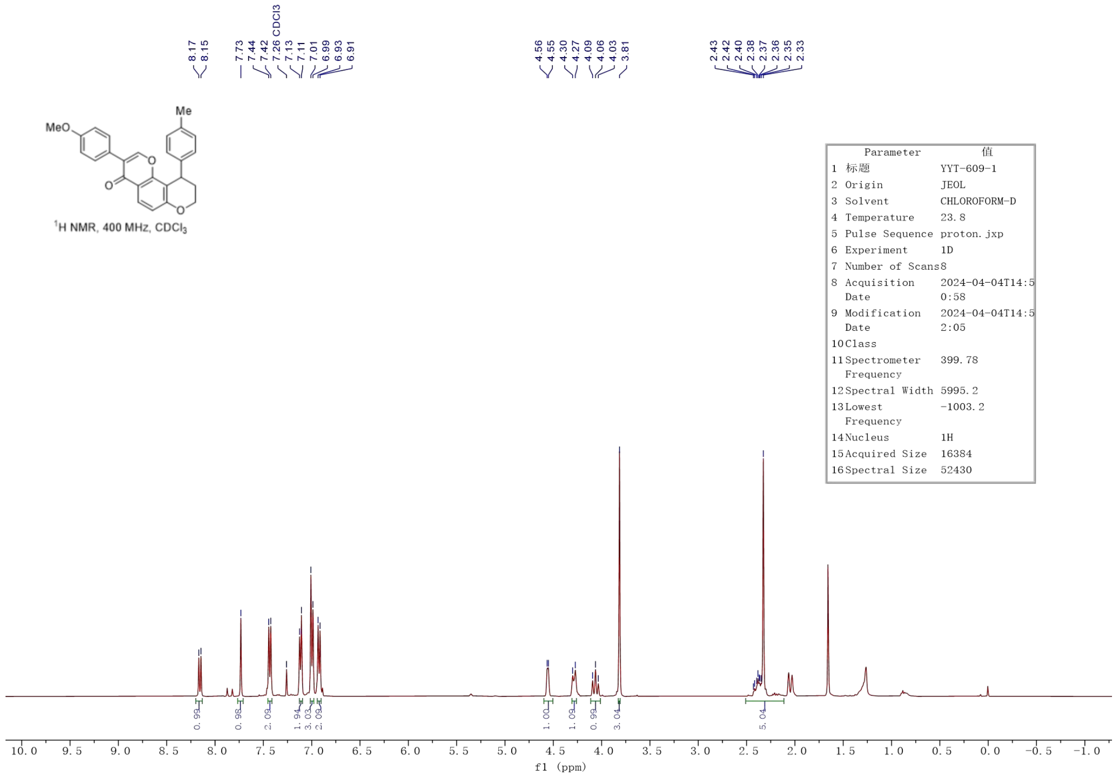

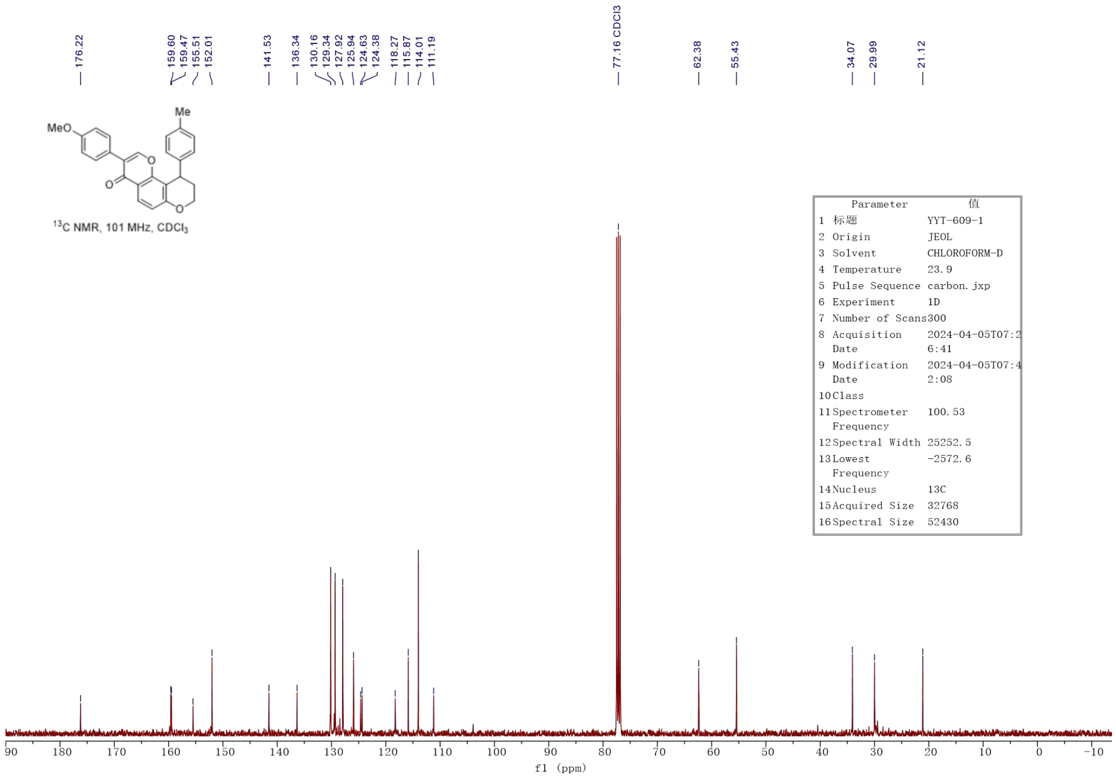

4-(9-(4-(p-tolyl)chroman-6-yl)-9H-fluoren-9-yl)phenol (88)

^{1}H NMR, 400MHz, $CDCl_3$

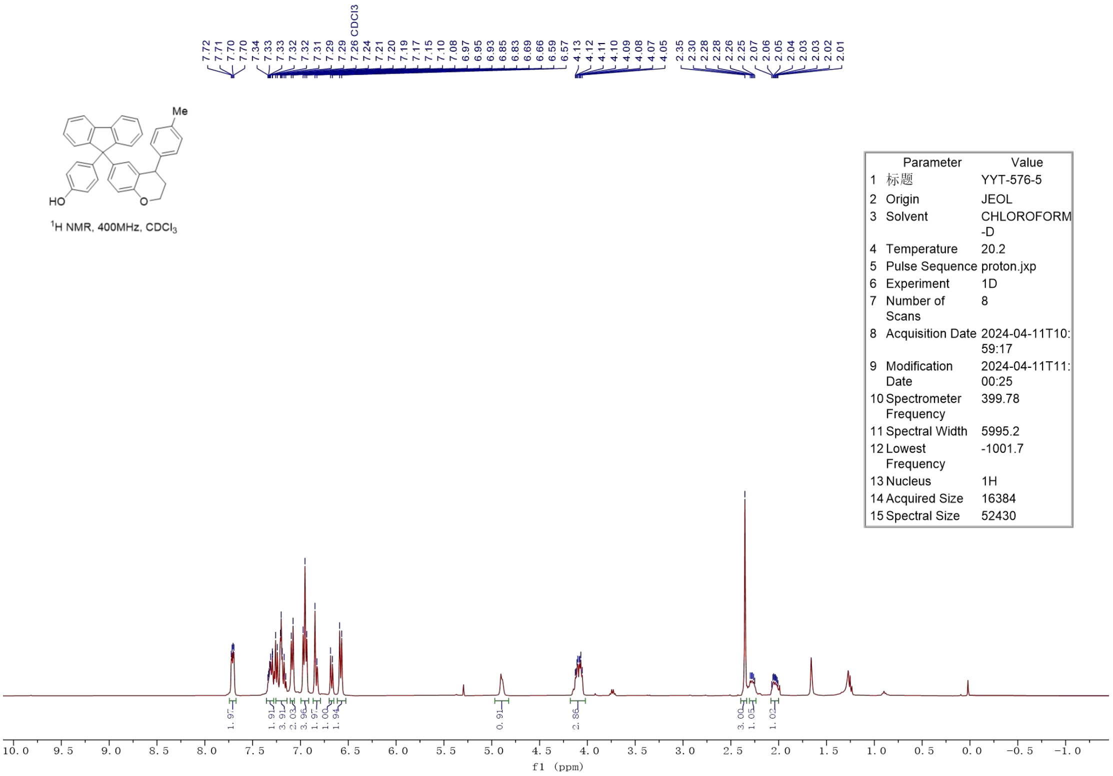

Parameter		Value
1	标题	YYT-576-5
2	Origin	JEOL
3	Solvent	CHLOROFORM-D
4	Temperature	20.2
5	Pulse Sequence	proton.jxp
6	Experiment	1D
7	Number of Scans	8
8	Acquisition Date	2024-04-11T10:59:17
9	Modification Date	2024-04-11T11:00:25
10	Spectrometer Frequency	399.78
11	Spectral Width	5995.2
12	Lowest Frequency	-1001.7
13	Nucleus	1H
14	Acquired Size	16384
15	Spectral Size	52430

^{13}C NMR, 101MHz, $CDCl_3$

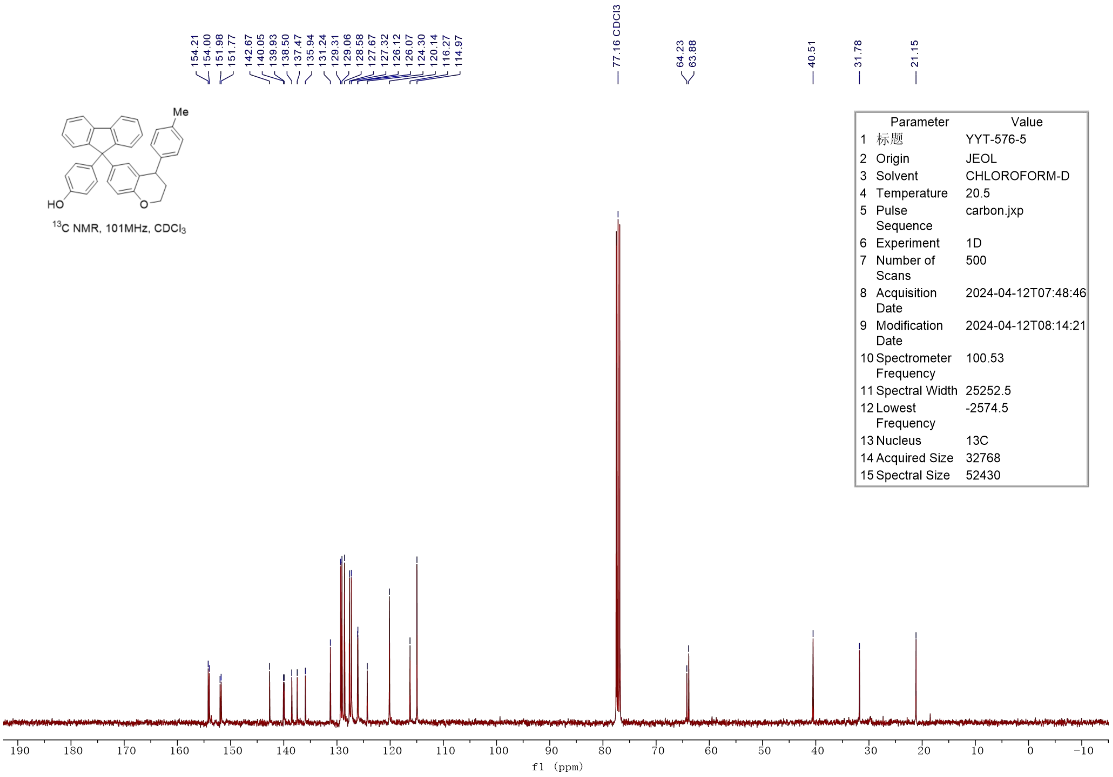

Parameter		Value
1	标题	YYT-576-5
2	Origin	JEOL
3	Solvent	CHLOROFORM-D
4	Temperature	20.5
5	Pulse Sequence	carbon.jxp
6	Experiment	1D
7	Number of Scans	500
8	Acquisition Date	2024-04-12T07:48:46
9	Modification Date	2024-04-12T08:14:21
10	Spectrometer Frequency	100.53
11	Spectral Width	25252.5
12	Lowest Frequency	-2574.5
13	Nucleus	13C
14	Acquired Size	32768
15	Spectral Size	52430

6-((4-isopropoxyphenyl)sulfonyl)-4-(p-tolyl)chromane (89)

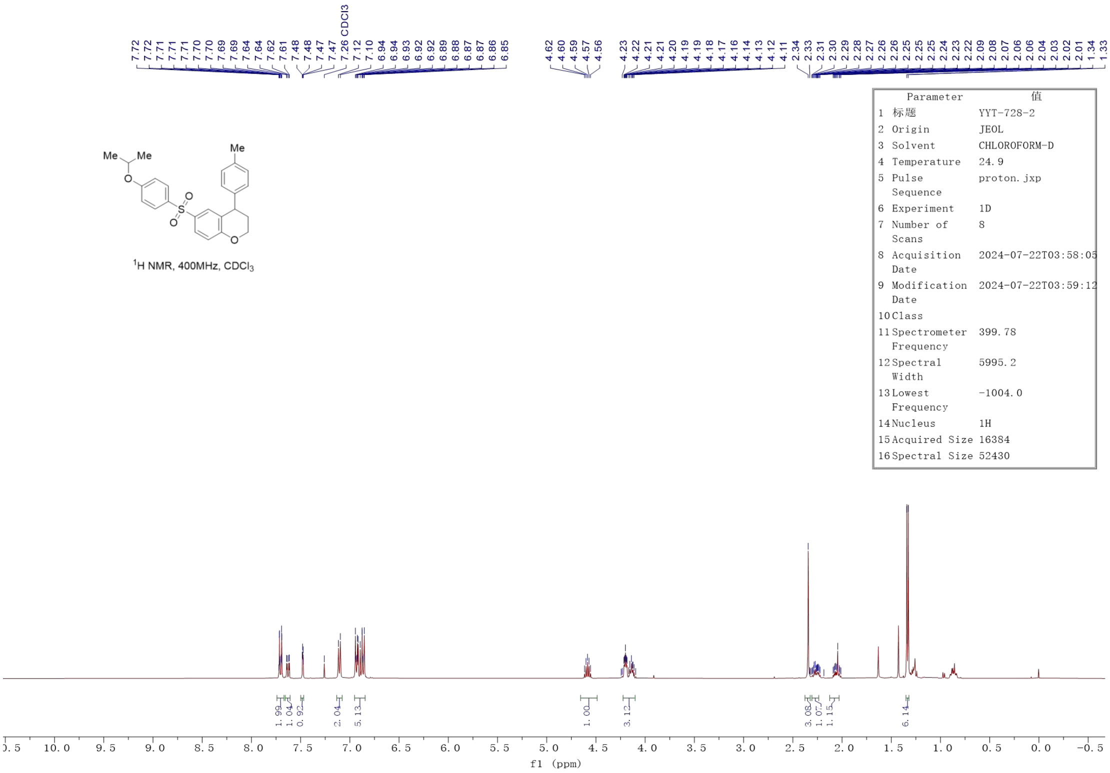

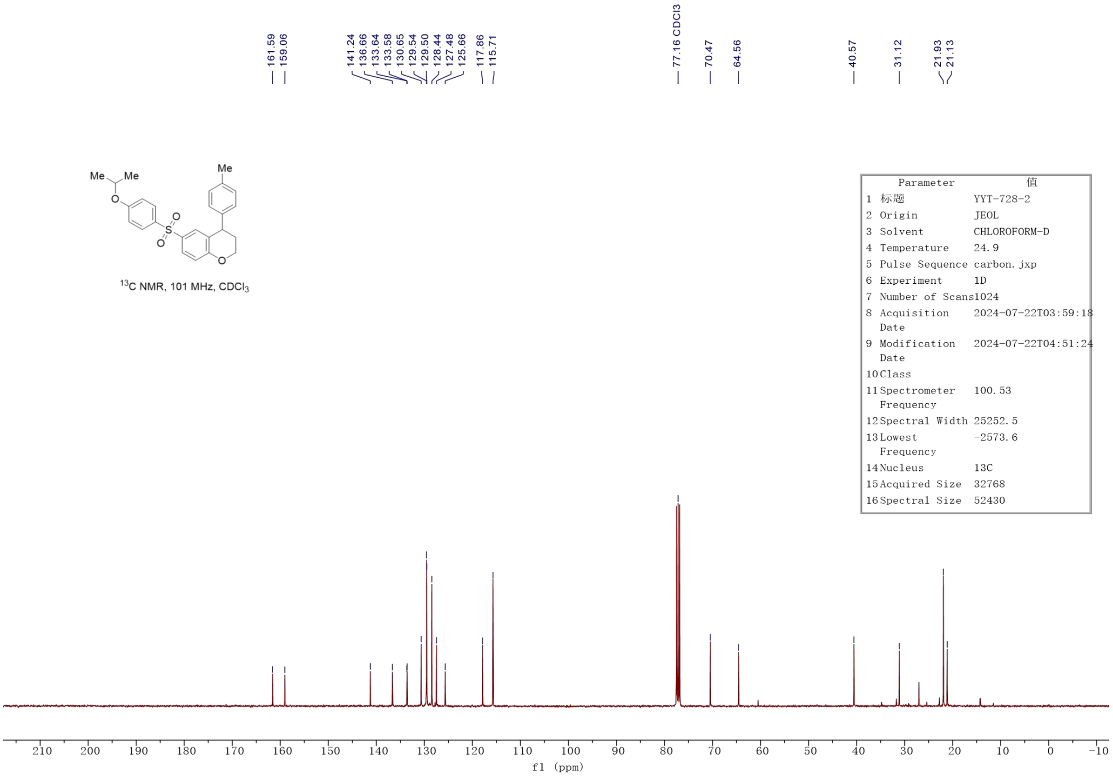

(3aR,5aS,13aS,13bS)-3a-methyl-11-(p-tolyl)-1,3a,4,5,5a,6,9,10,11,13,13a,13b-dodecahydrocyclopenta[7,8]naphtho[2,3-g]chromen-3(2H)-one (90)

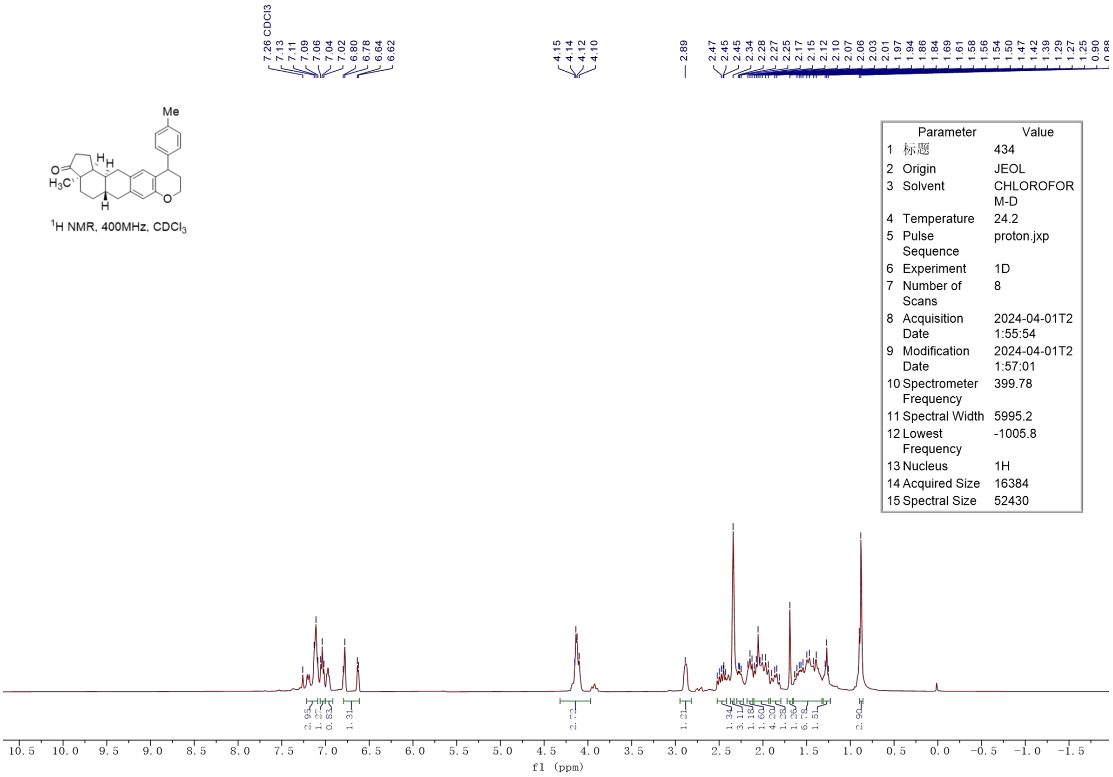

	Parameter	Value
1	标题	434
2	Origin	JEOL
3	Solvent	CHLOROFORM-D
4	Temperature	24.2
5	Pulse Sequence	proton.jxp
6	Experiment	1D
7	Number of Scans	8
8	Acquisition Date	2024-04-01T21:55:54
9	Modification Date	2024-04-01T21:57:01
10	Spectrometer Frequency	399.78
11	Spectral Width	5995.2
12	Lowest Frequency	-1005.8
13	Nucleus	1H
14	Acquired Size	16384
15	Spectral Size	52430

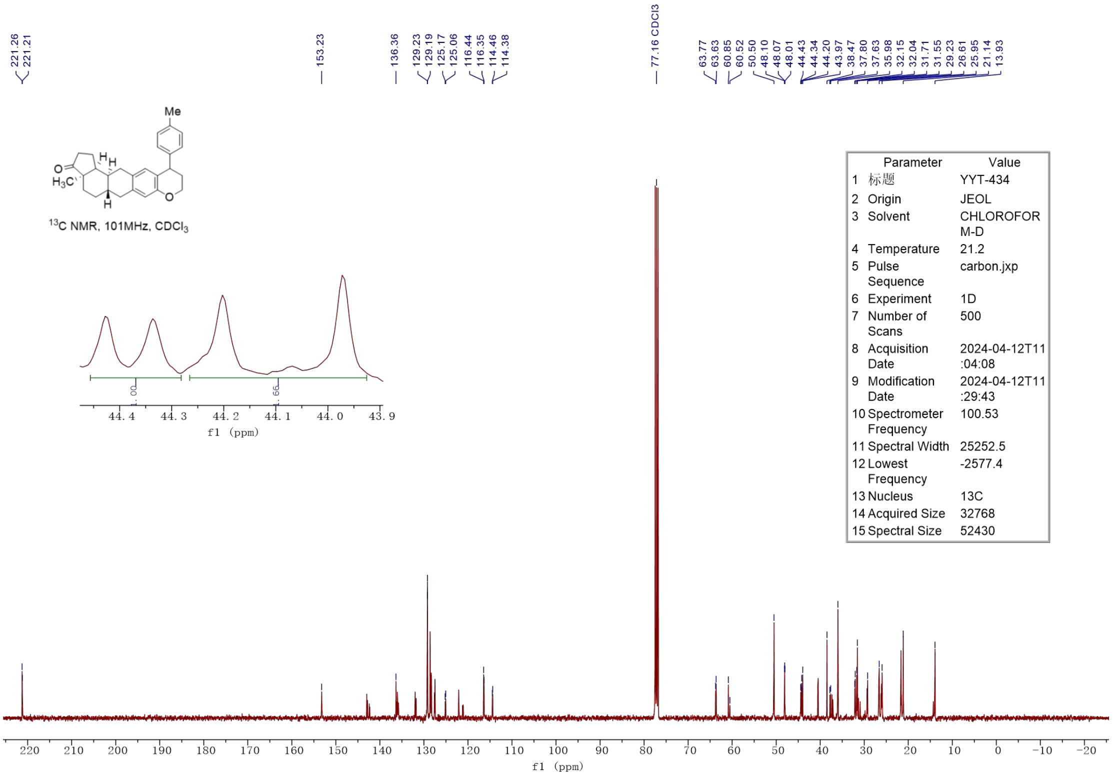

	Parameter	Value
1	标题	YYT-434
2	Origin	JEOL
3	Solvent	CHLOROFORM-D
4	Temperature	21.2
5	Pulse Sequence	carbon.jxp
6	Experiment	1D
7	Number of Scans	500
8	Acquisition Date	2024-04-12T11:04:08
9	Modification Date	2024-04-12T11:29:43
10	Spectrometer Frequency	100.53
11	Spectral Width	25252.5
12	Lowest Frequency	-2577.4
13	Nucleus	13C
14	Acquired Size	32768
15	Spectral Size	52430

7-(4-bromophenyl)-13a-methyl-1,2,3,3a,3b,4,5,7,8,9,11b,12,13,13a-tetradecahydrocyclopenta[5,6]naphtho[2,1-*g*]chromen-1-yl pentanoate (91)

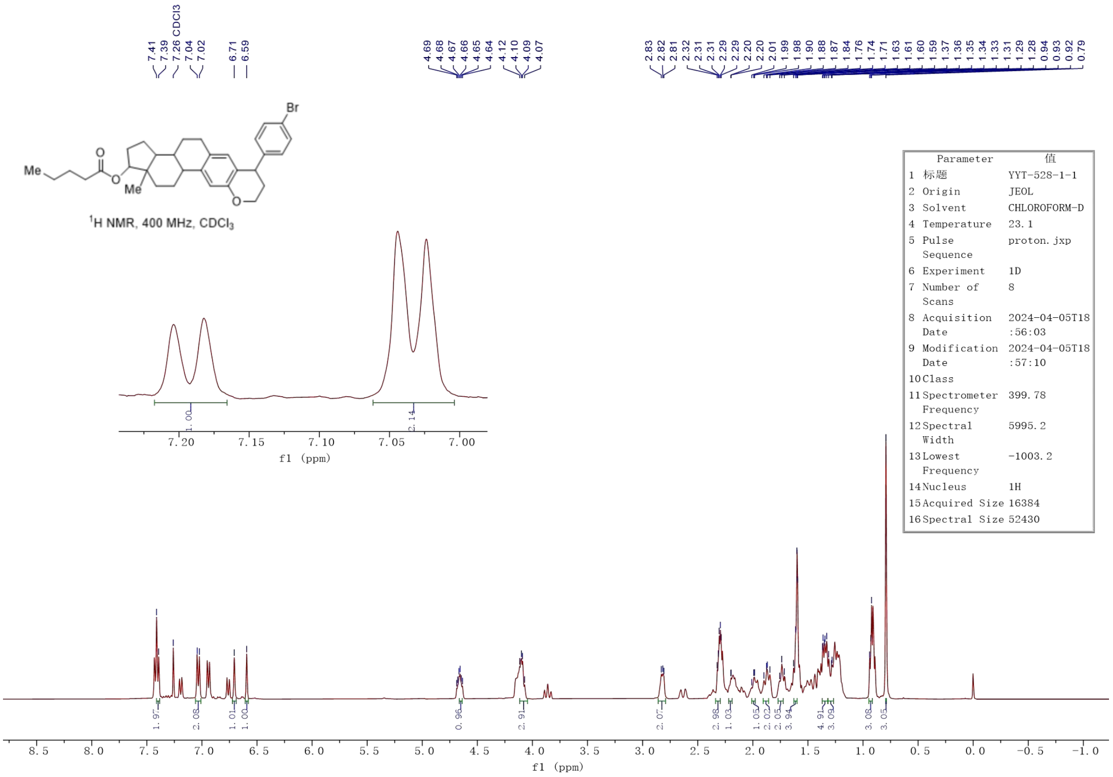

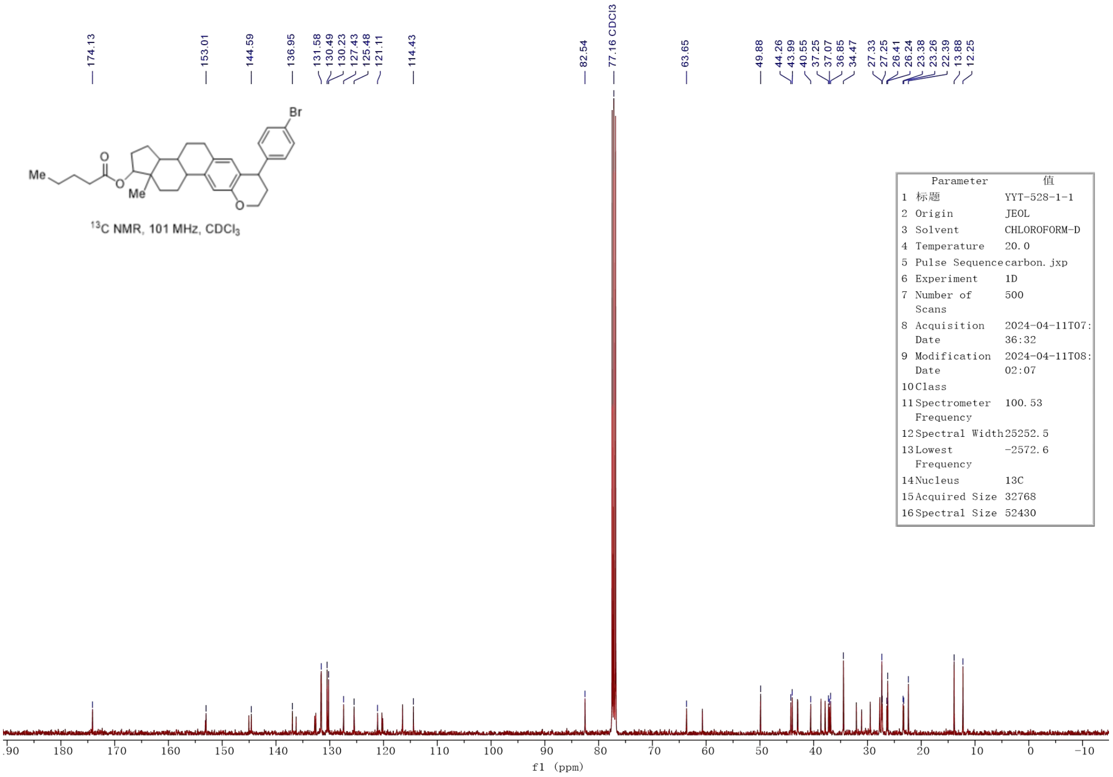

1-(p-tolyl)-9-(4-(trifluoromethyl)phenyl)-2,3,6,7-tetrahydro-1H,5H-pyrido[3,2,1-ij]quinolin-5-one (92)

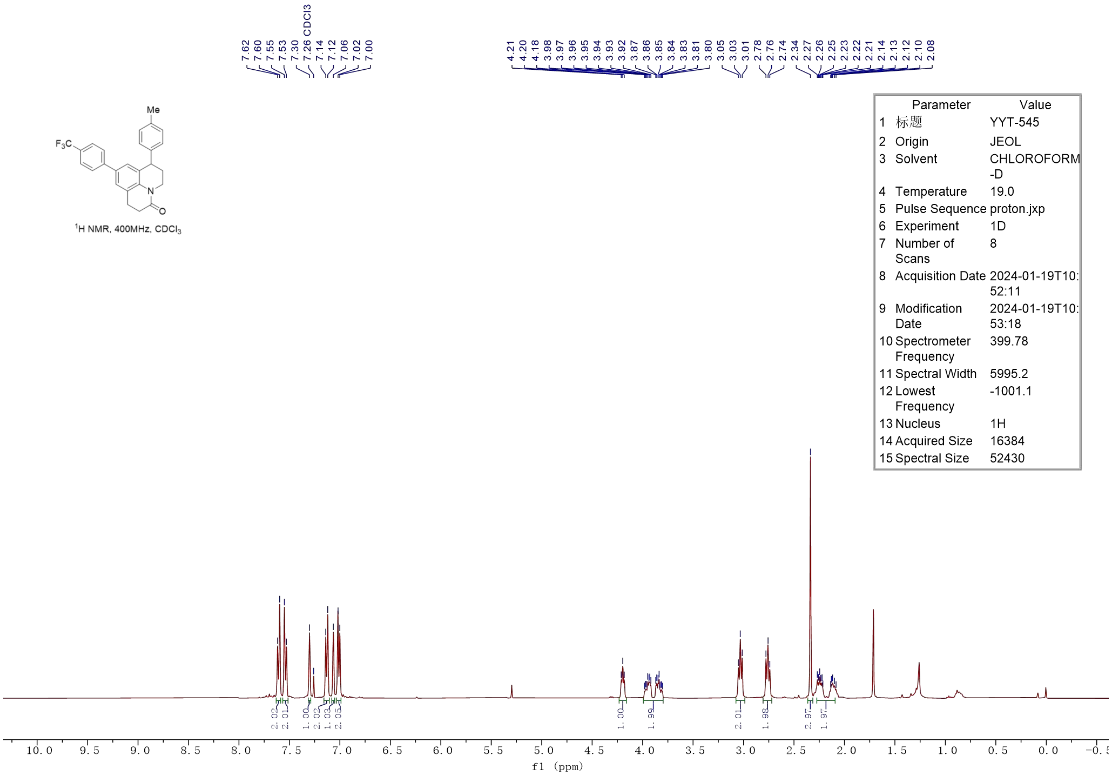

	Parameter	Value
1	标题	YYT-545
2	Origin	JEOL
3	Solvent	CHLOROFORM-D
4	Temperature	19.0
5	Pulse Sequence	proton.jxp
6	Experiment	1D
7	Number of Scans	8
8	Acquisition Date	2024-01-19T10:52:11
9	Modification Date	2024-01-19T10:53:18
10	Spectrometer Frequency	399.78
11	Spectral Width	5995.2
12	Lowest Frequency	-1001.1
13	Nucleus	1H
14	Acquired Size	16384
15	Spectral Size	52430

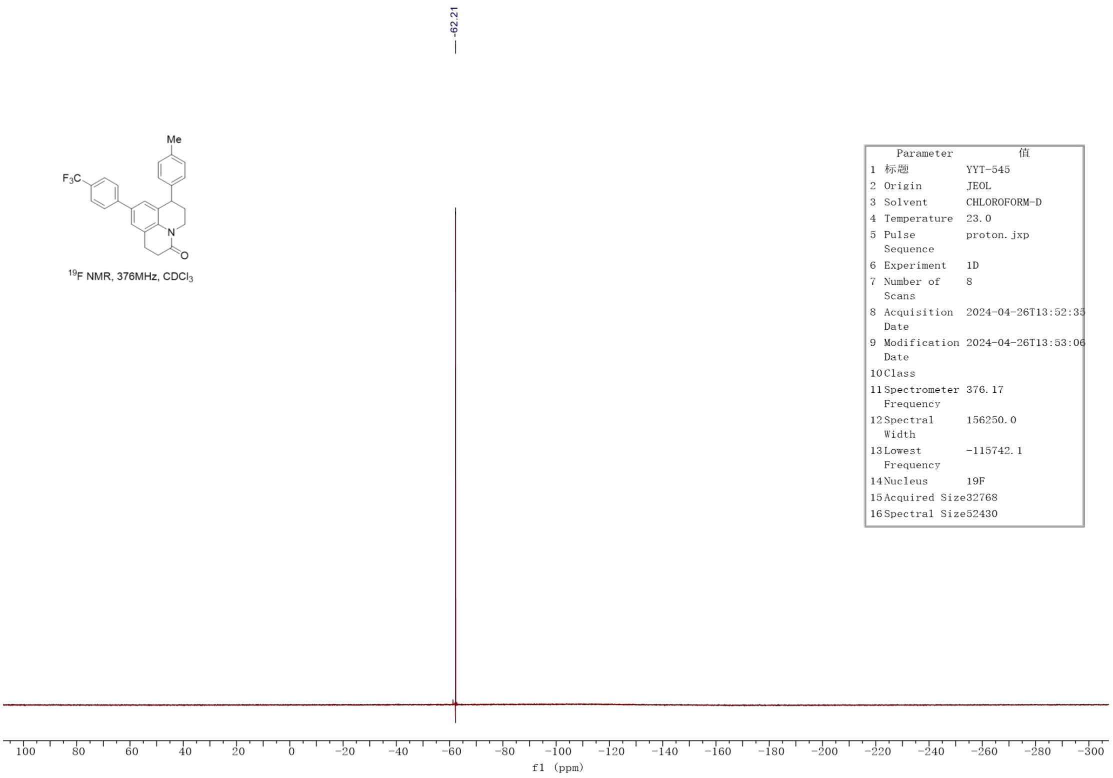

	Parameter	值
1	标题	YYT-545
2	Origin	JEOL
3	Solvent	CHLOROFORM-D
4	Temperature	23.0
5	Pulse Sequence	proton.jxp
6	Experiment	1D
7	Number of Scans	8
8	Acquisition Date	2024-04-26T13:52:35
9	Modification Date	2024-04-26T13:53:06
10	Class	
11	Spectrometer Frequency	376.17
12	Spectral Width	156250.0
13	Lowest Frequency	-115742.1
14	Nucleus	19F
15	Acquired Size	32768
16	Spectral Size	52430

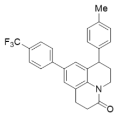

^{13}C NMR, 101MHz, $CDCl_3$

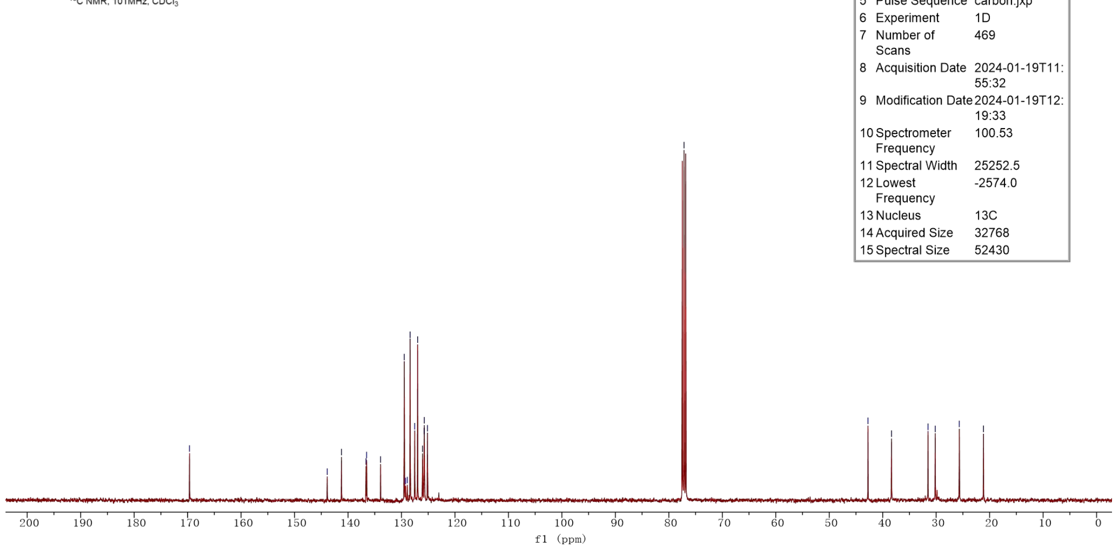

	Parameter	Value
1	标题	YYT-545
2	Origin	JEOL
3	Solvent	CHLOROFORM-D
4	Temperature	19.2
5	Pulse Sequence	carbon.jxp
6	Experiment	1D
7	Number of Scans	469
8	Acquisition Date	2024-01-19T11:55:32
9	Modification Date	2024-01-19T12:19:33
10	Spectrometer Frequency	100.53
11	Spectral Width	25252.5
12	Lowest Frequency	-2574.0
13	Nucleus	13C
14	Acquired Size	32768
15	Spectral Size	52430

9-hydroxy-1-(p-tolyl)-2,3,6,7-tetrahydro-1H,5H-pyrido[3,2,1-ij]quinolin-5-one (93)

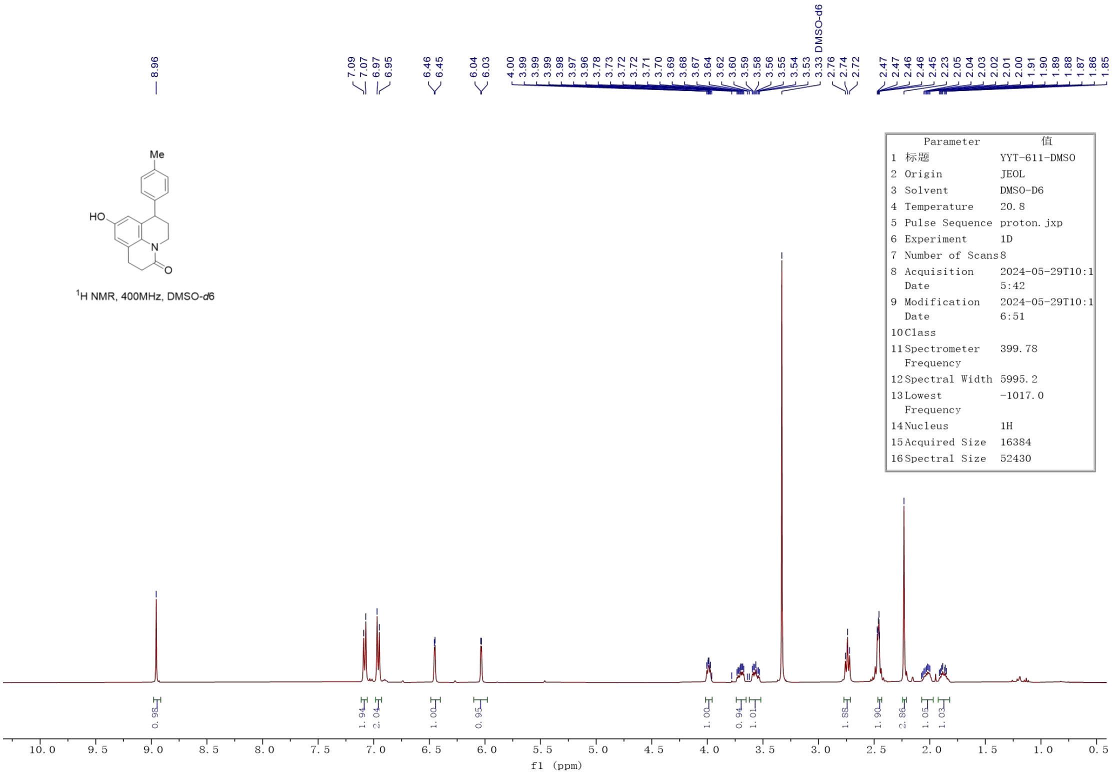

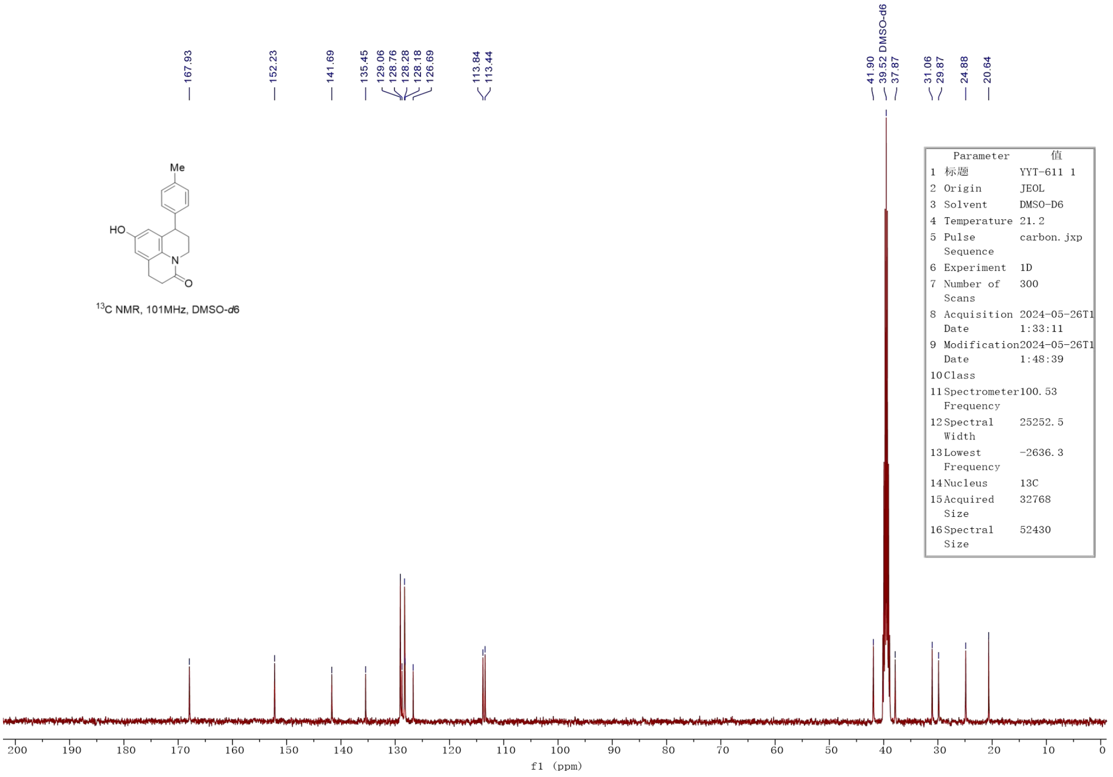

9-(4-(1-cyclohexyl-1H-tetrazol-5-yl)butoxy)-1-(p-tolyl)-2,3,6,7-tetrahydro-1H,5H-pyrido[3,2,1-ij]quinolin-5-one (94)

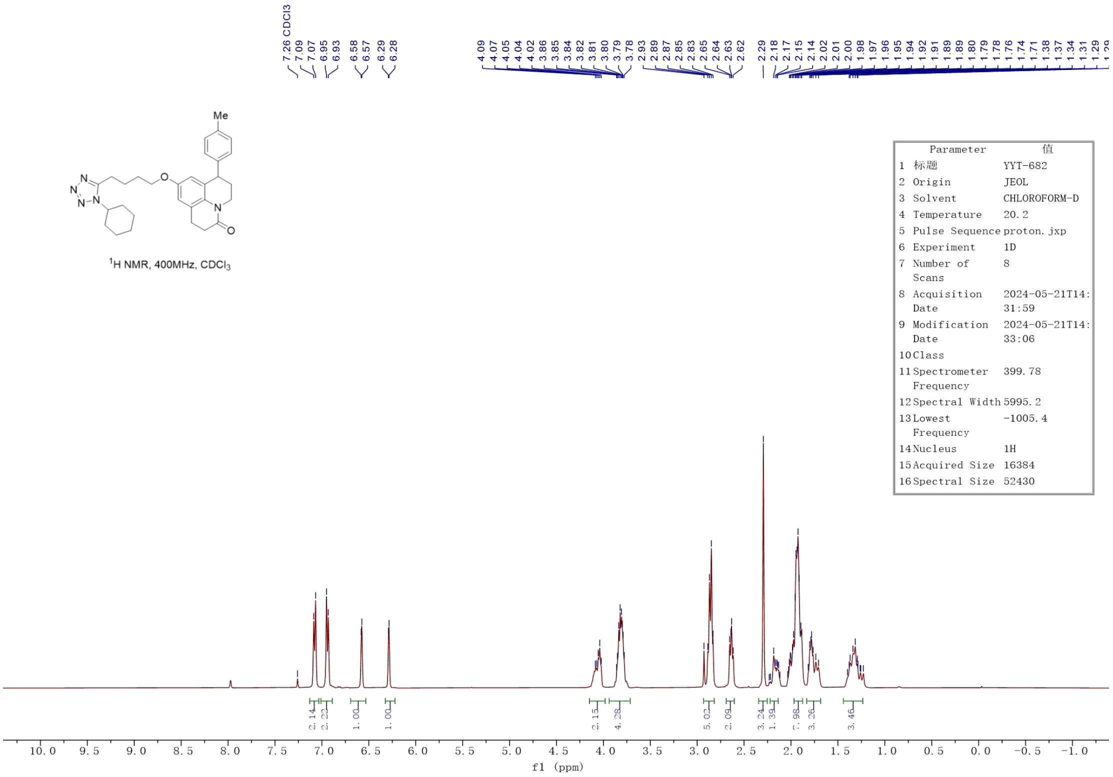

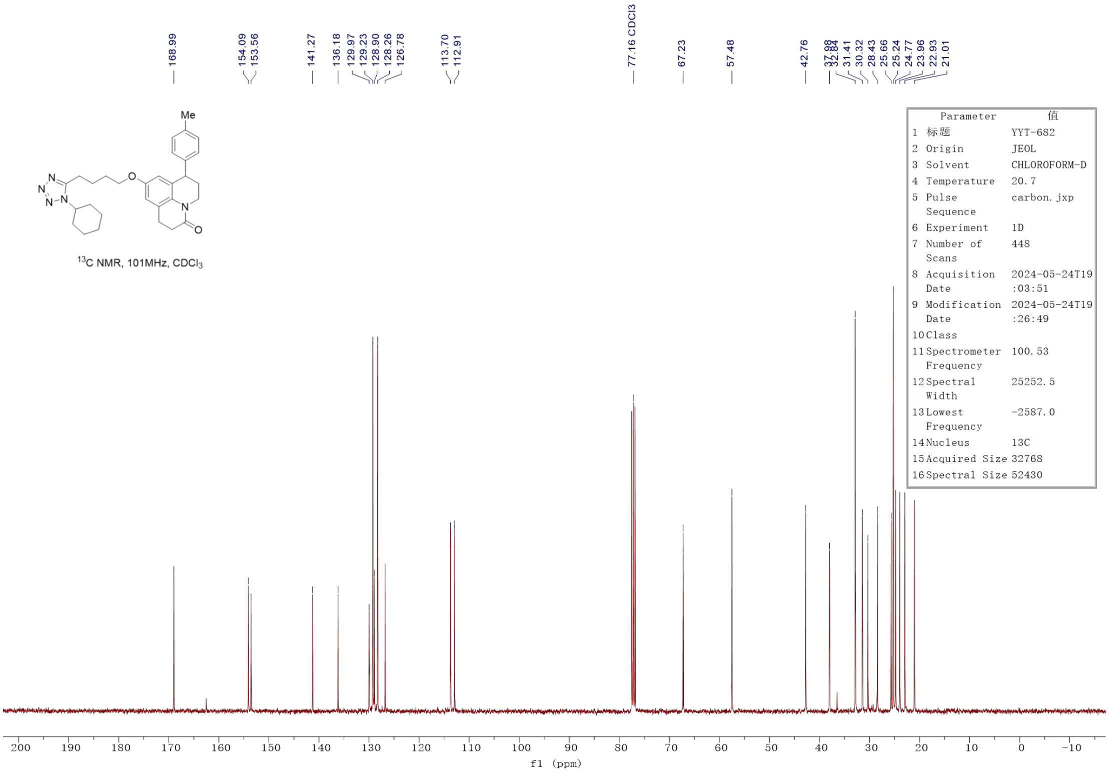

1-allyl-5-methoxyindoline-2,3-dione (95)

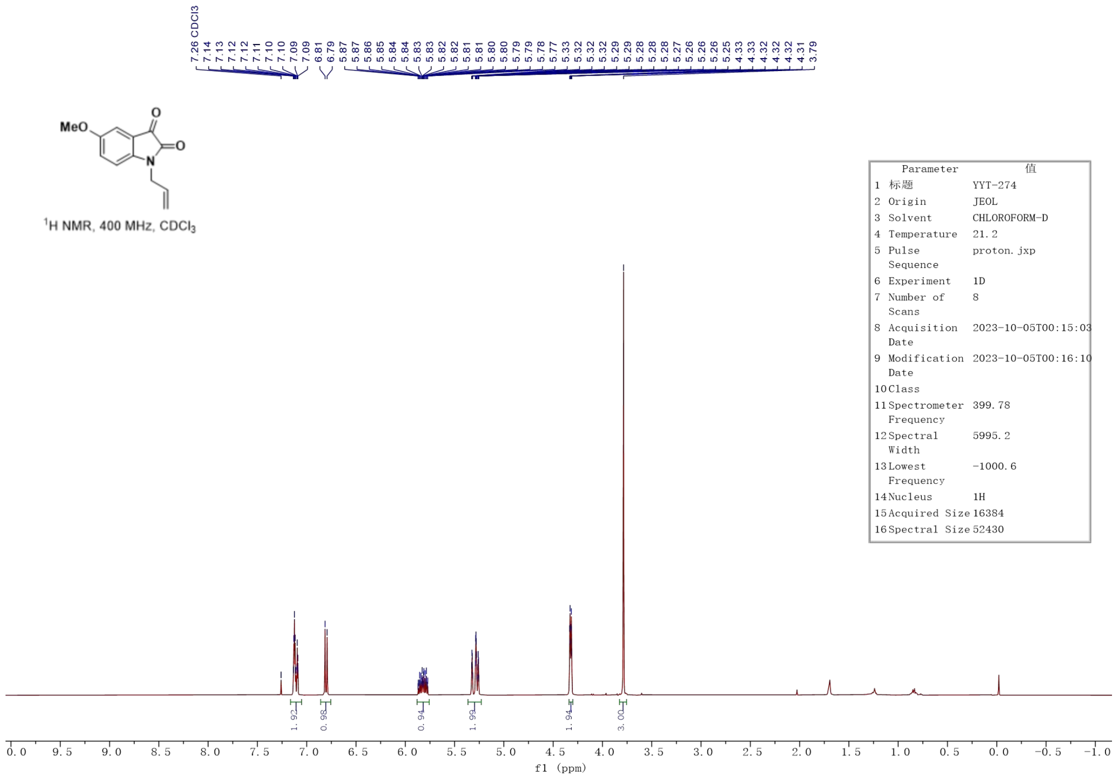

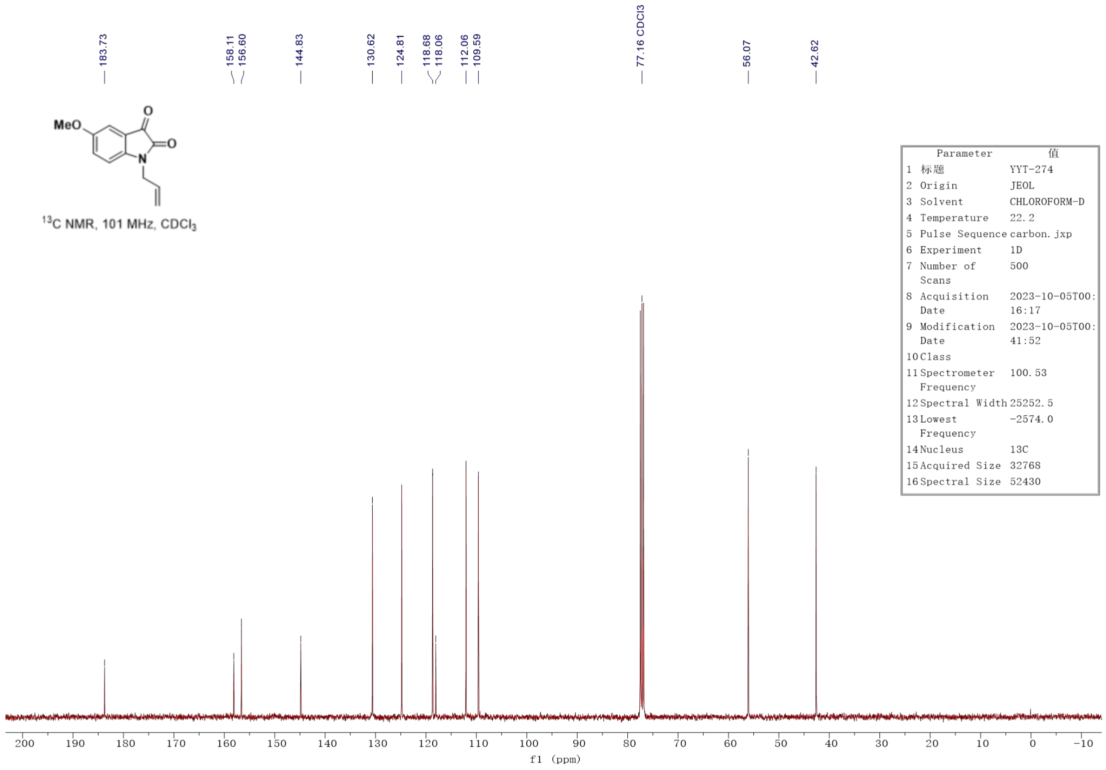

1-allyl-5-methoxyspiro[indoline-3,2'-[1,3]dioxolan]-2-one (96)

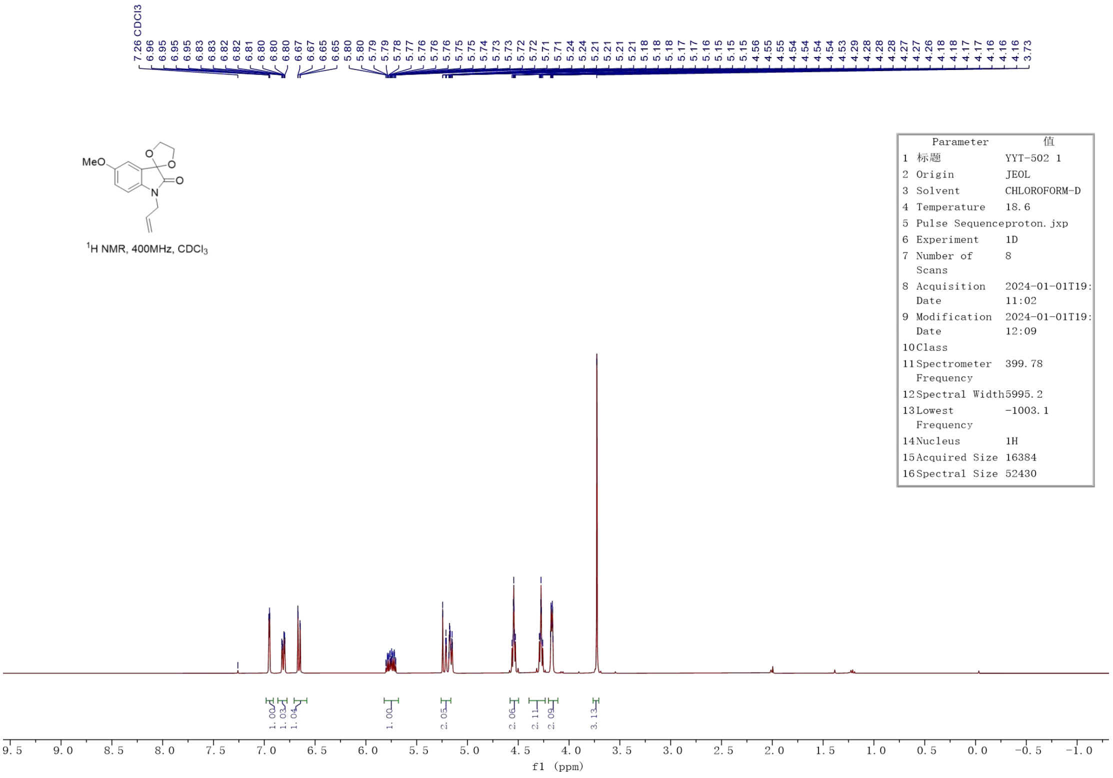

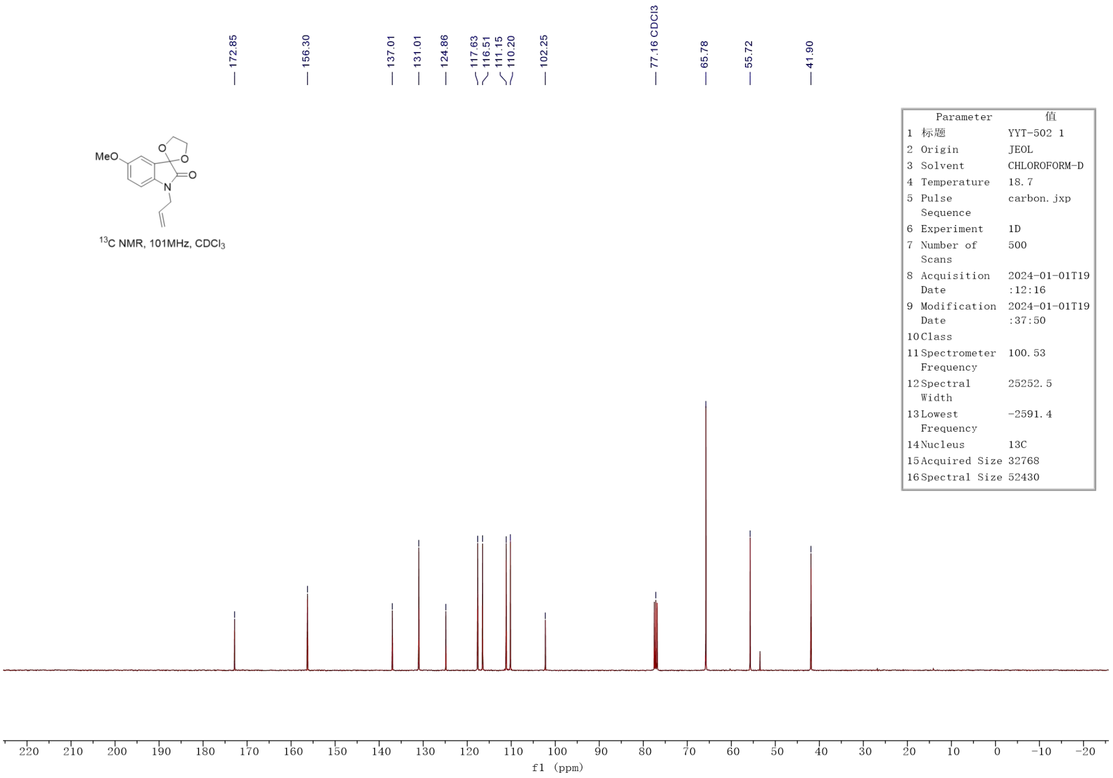

(*E*)-5-methoxy-1-(3-(p-tolyl)allyl)indoline-2,3-dione (97)

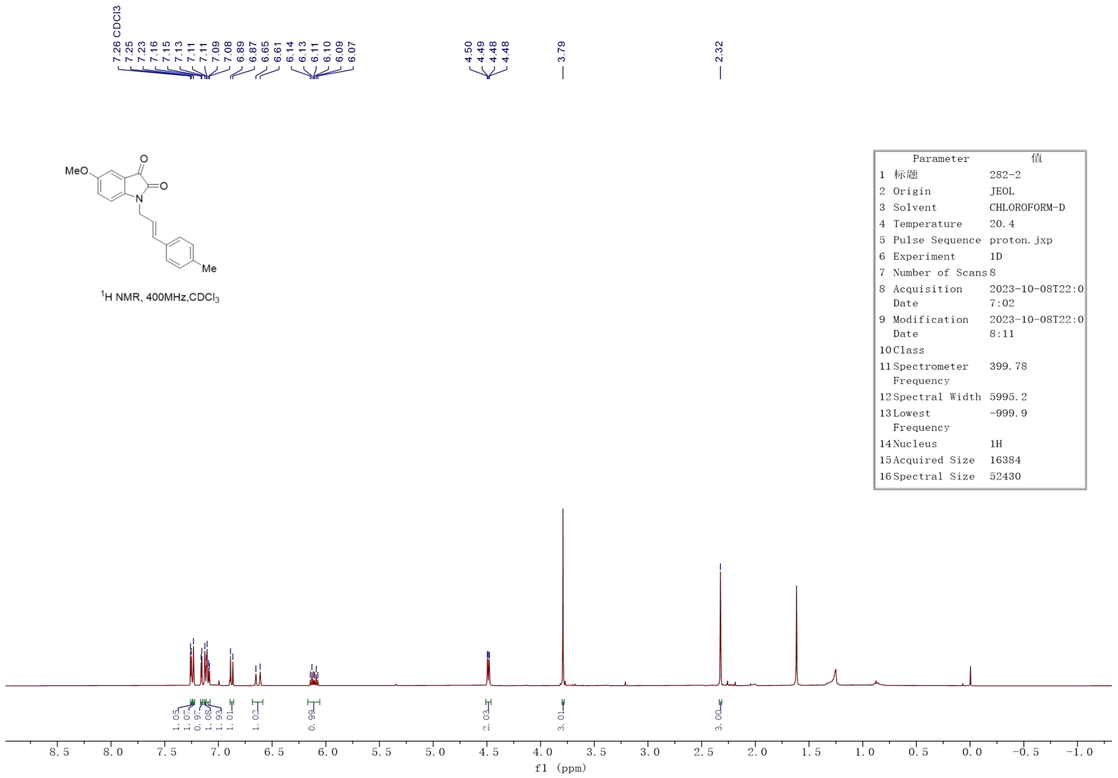

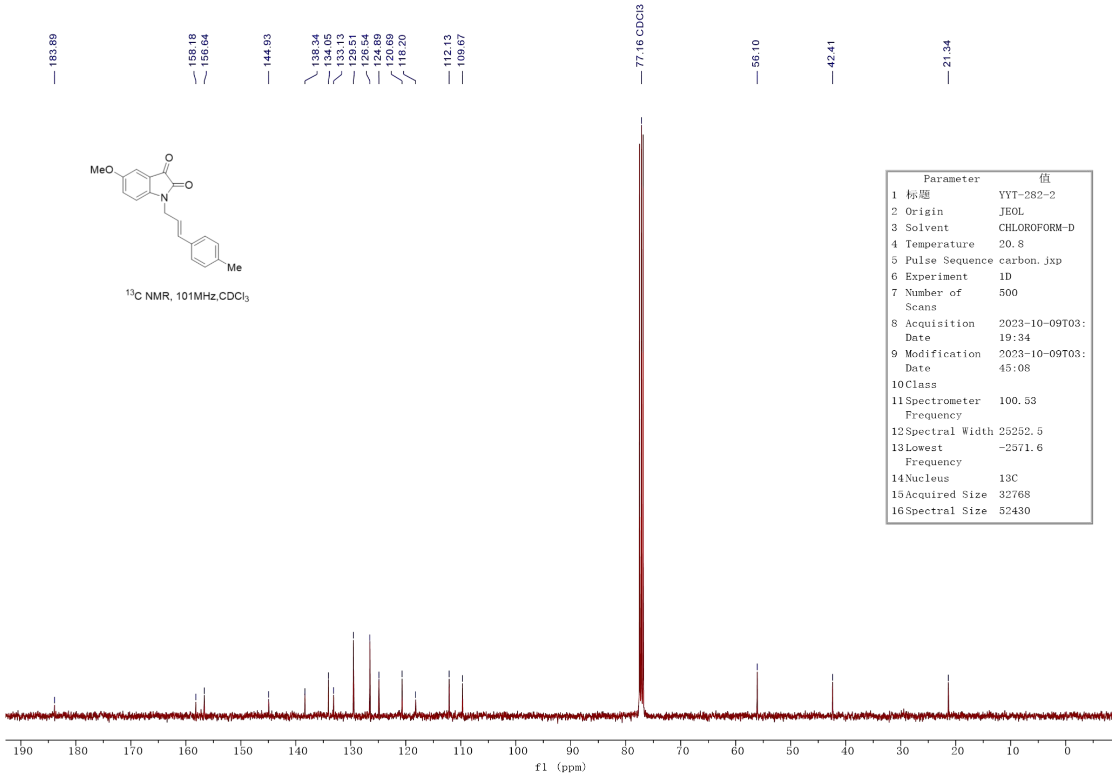

1-(3,3-di-p-tolylallyl)-5-methoxyindoline-2,3-dione (98)

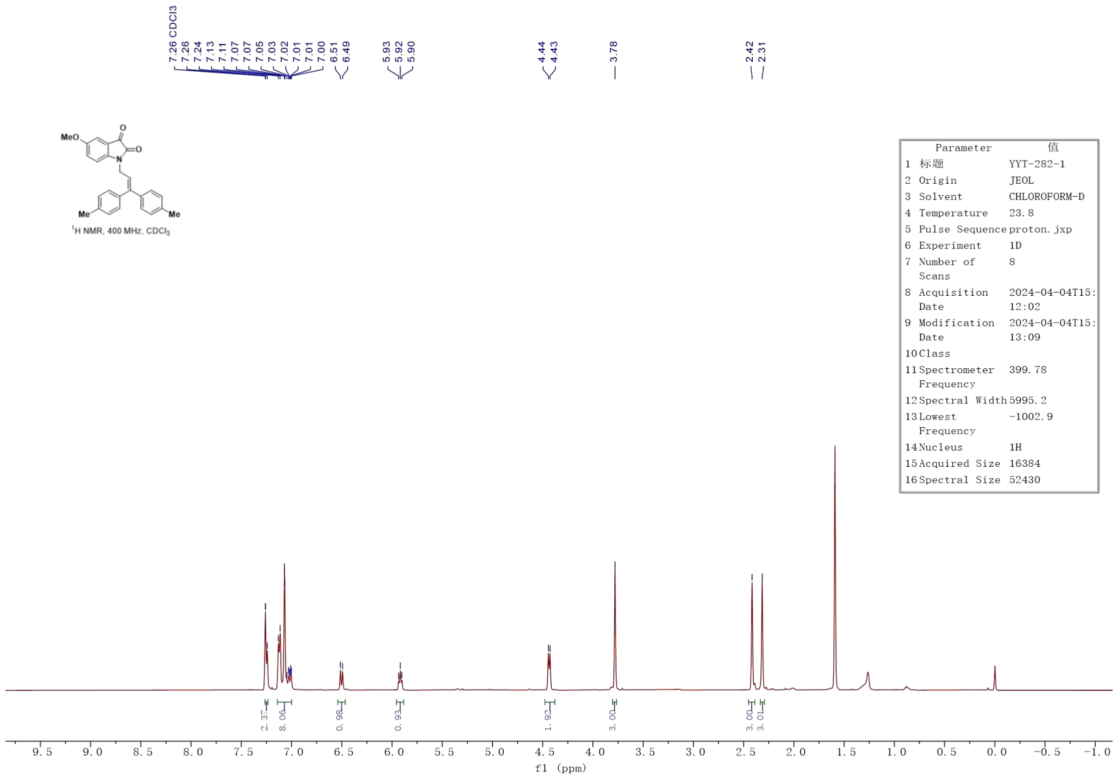

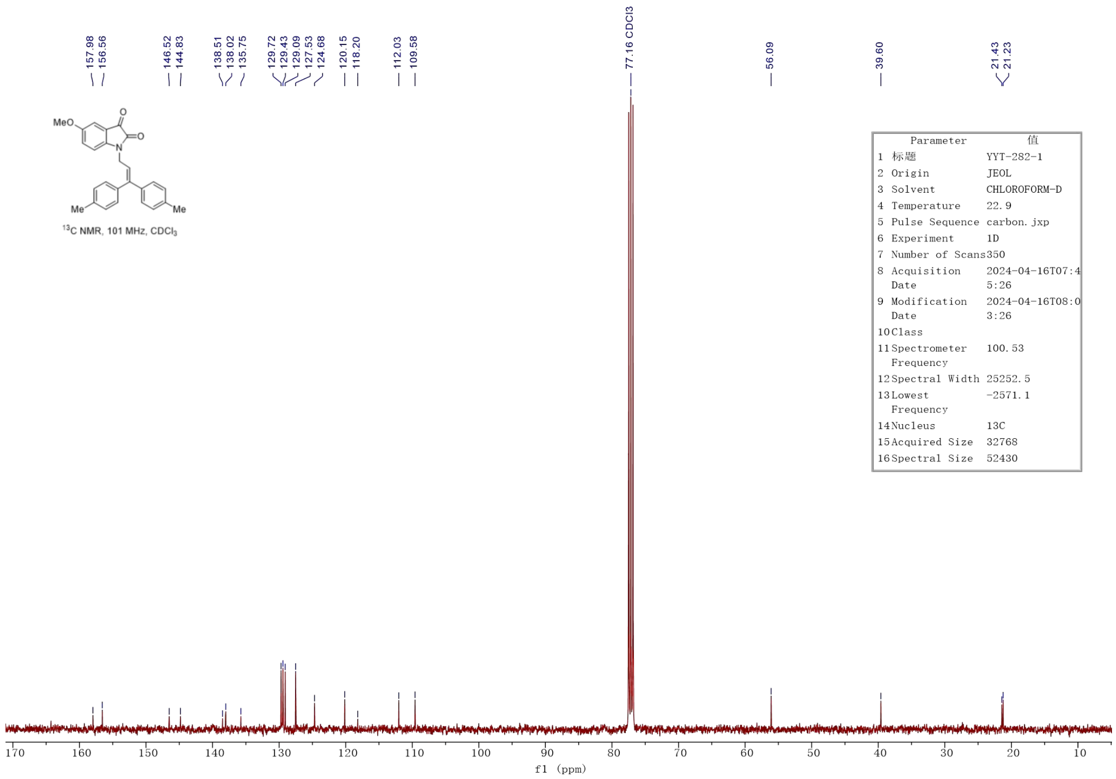

8-methoxy-6-(p-tolyl)-5,6-dihydro-2H,4H-spiro[pyrrolo[3,2,1-ij]quinoline-1,2'-[1,3]dioxolan]-2-one (99)

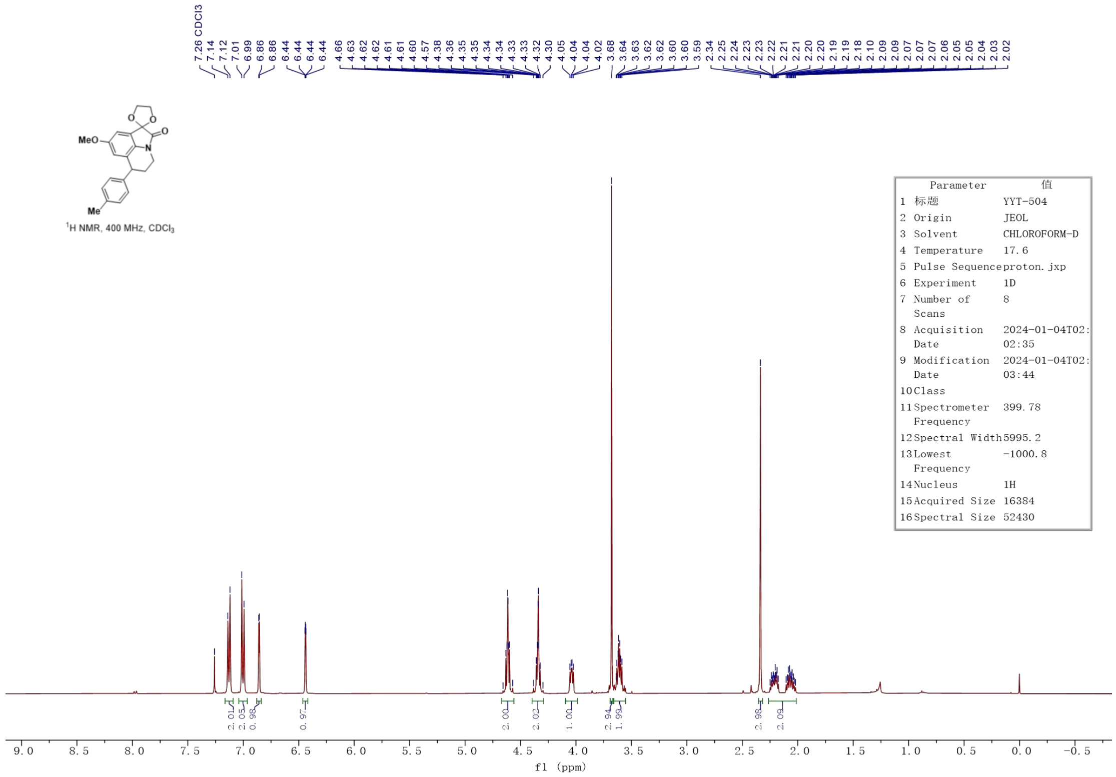

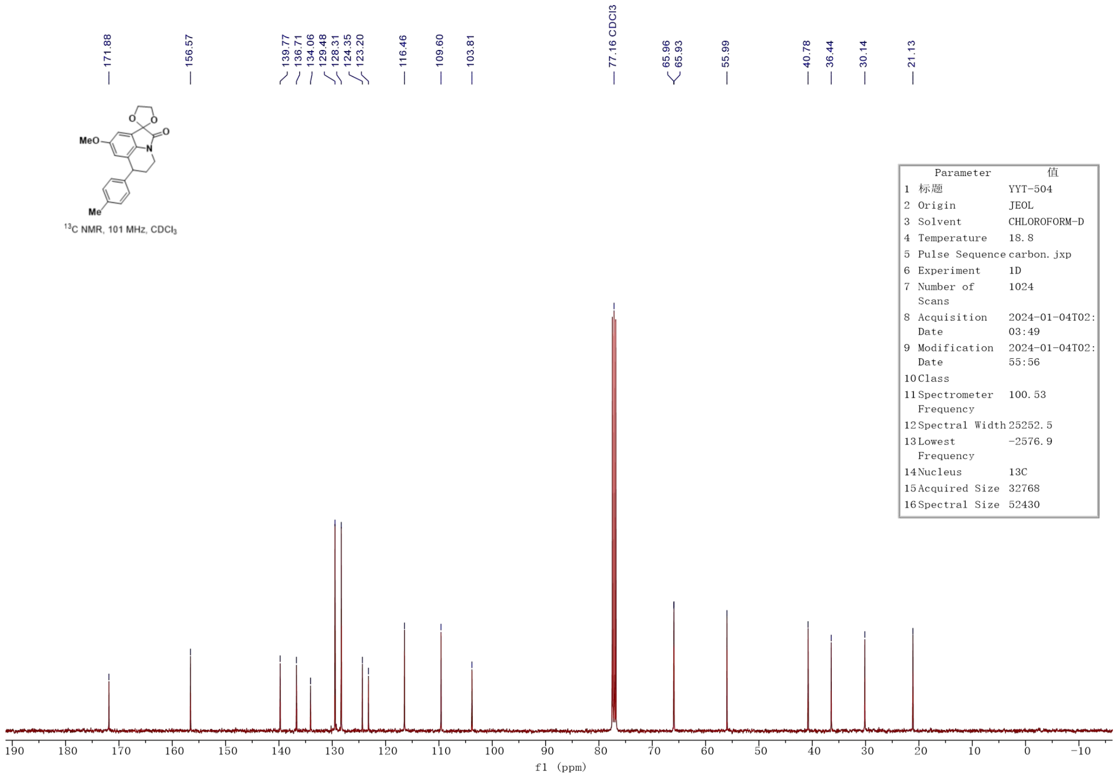

4-(1-oxo-1,2,7,8,9,10-hexahydro-3-oxa-10a-azacyclohepta[de]naphthalen-7-yl)benzonitrile (101)

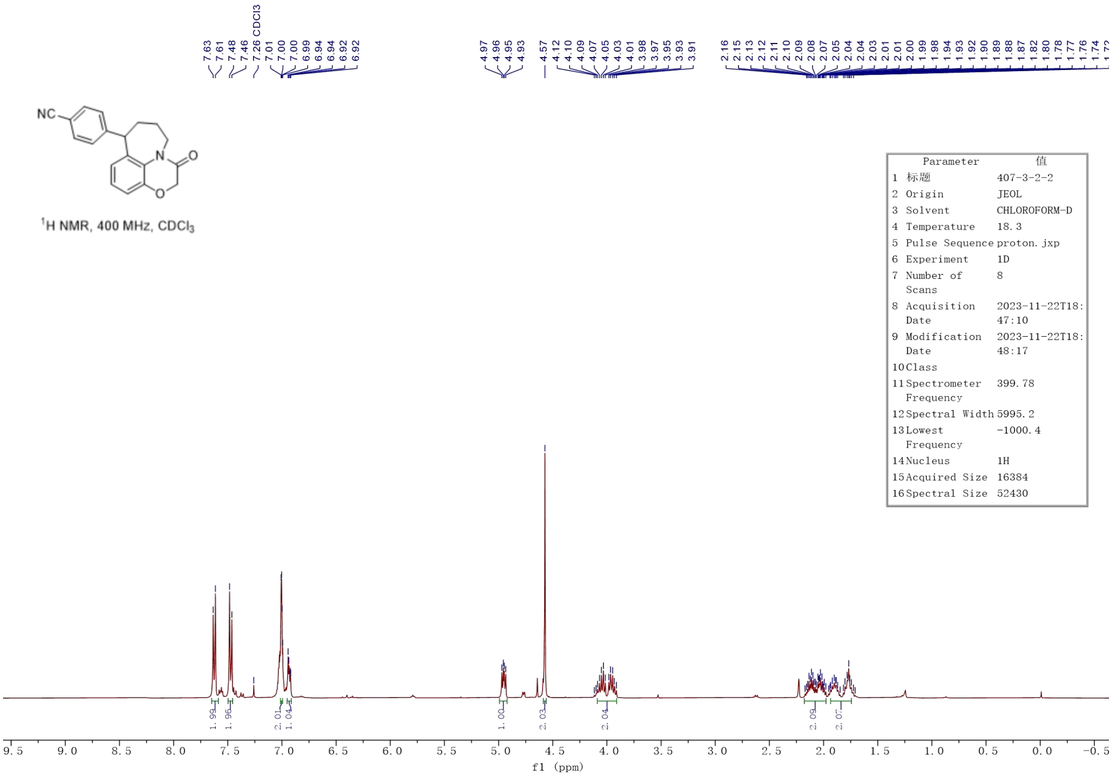

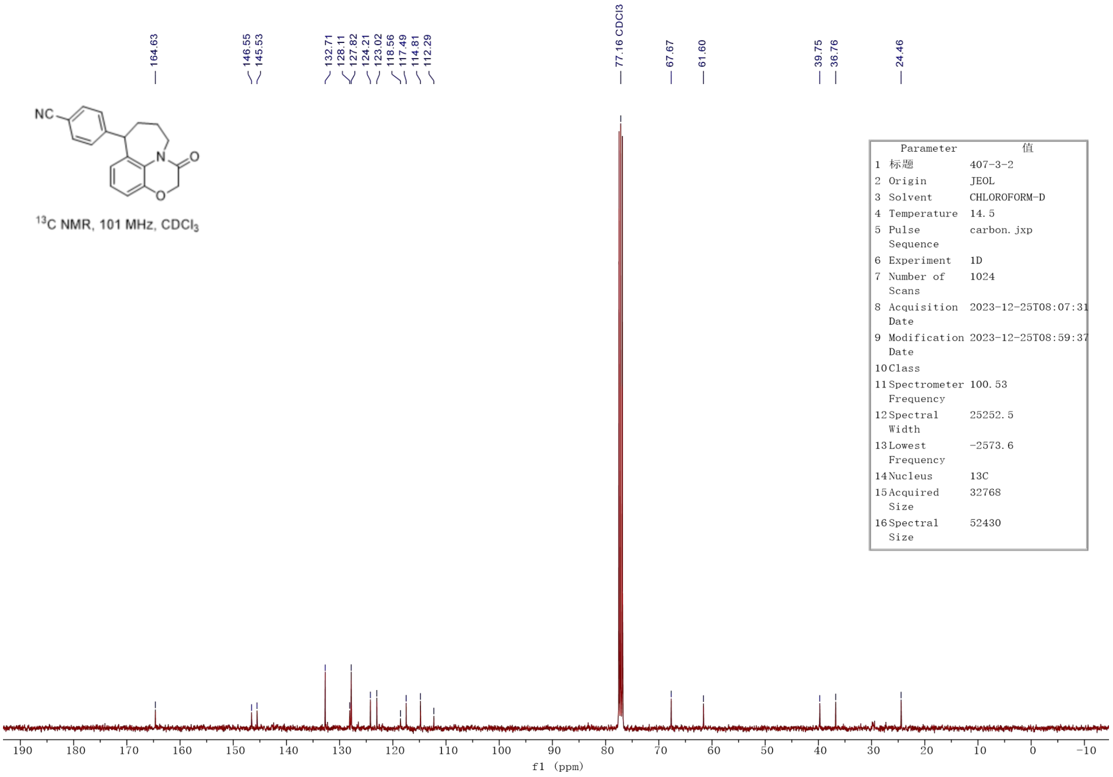

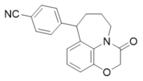

DEPT-135, 101 MHz, $CDCl_3$

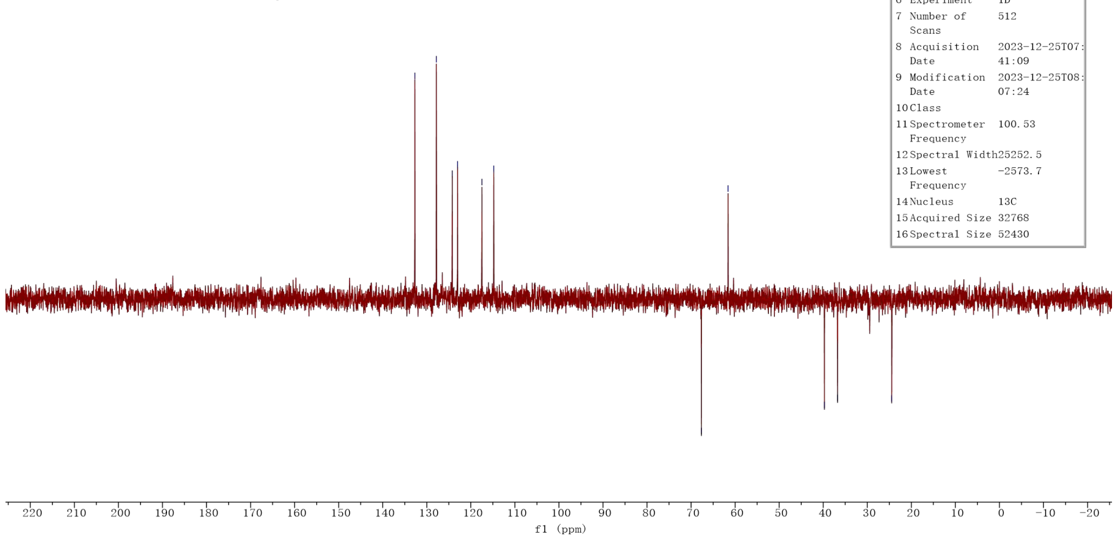

7-(*p*-tolyl)-7,8,9,10,11,12-hexahydro-3-oxa-12a-azacyclonona[*de*]naphthalen-1(2*H*)-one (102)

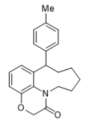

^{1}H NMR, 400 MHz, $CDCl_3$

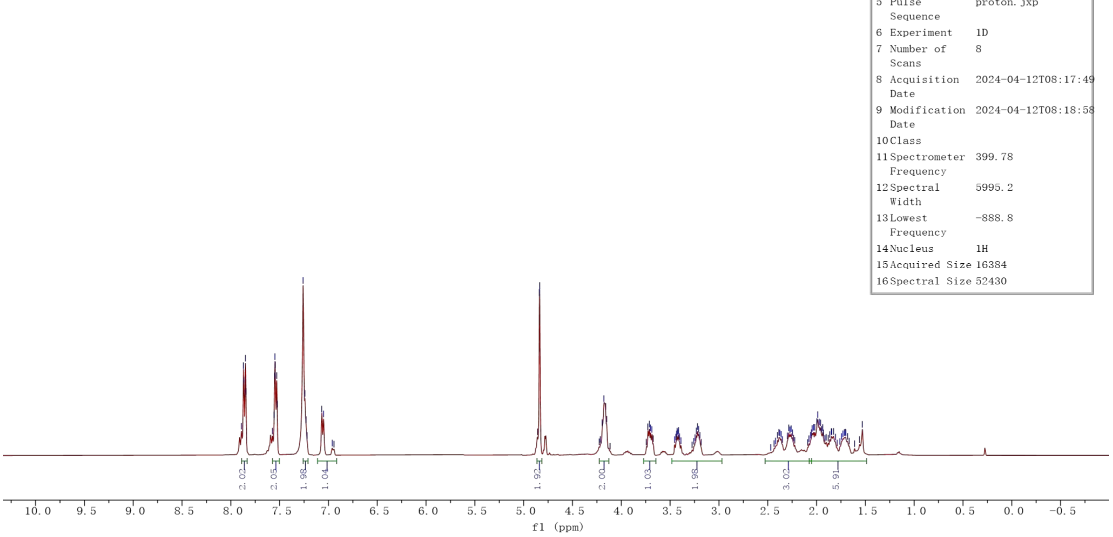

	Parameter	值
1	标题	YYT-618-2-1
2	Origin	JEOL
3	Solvent	CHLOROFORM-D
4	Temperature	20.7
5	Pulse Sequence	proton.jxp
6	Experiment	1D
7	Number of Scans	8
8	Acquisition Date	2024-04-12T08:17:49
9	Modification Date	2024-04-12T08:18:58
10	Class	
11	Spectrometer Frequency	399.78
12	Spectral Width	5995.2
13	Lowest Frequency	-888.8
14	Nucleus	1H
15	Acquired Size	16384
16	Spectral Size	52430

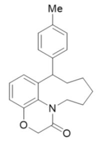

^{13}C NMR, 126MHz, $CDCl_3$

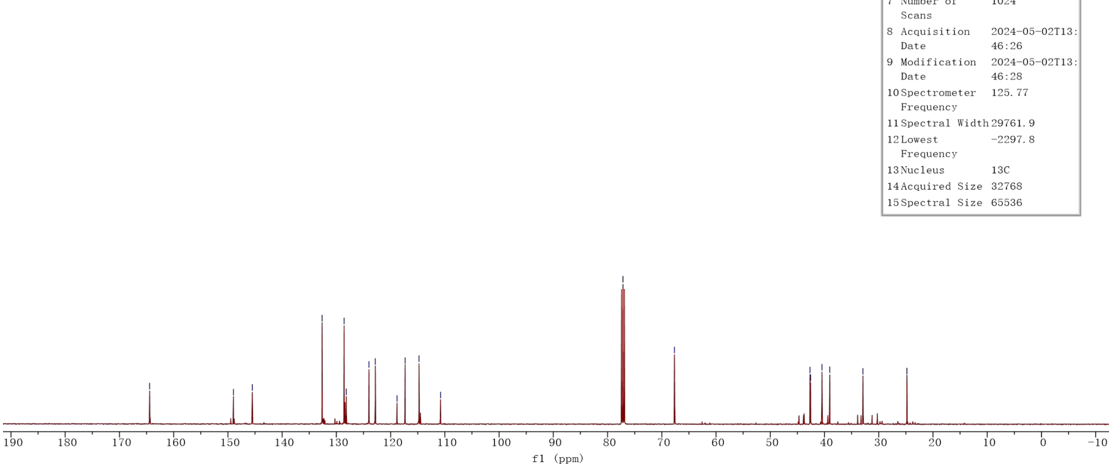

	Parameter	值
1	标题	YYT-618.1.fid
2	Origin	Bruker BioSpin GmbH
3	Solvent	CDCl3
4	Temperature	298.5
5	Pulse Sequence	zgpg30
6	Experiment	1D
7	Number of Scans	1024
8	Acquisition Date	2024-05-02T13:46:26
9	Modification Date	2024-05-02T13:46:28
10	Spectrometer Frequency	125.77
11	Spectral Width	29761.9
12	Lowest Frequency	-2297.8
13	Nucleus	13C
14	Acquired Size	32768
15	Spectral Size	65536

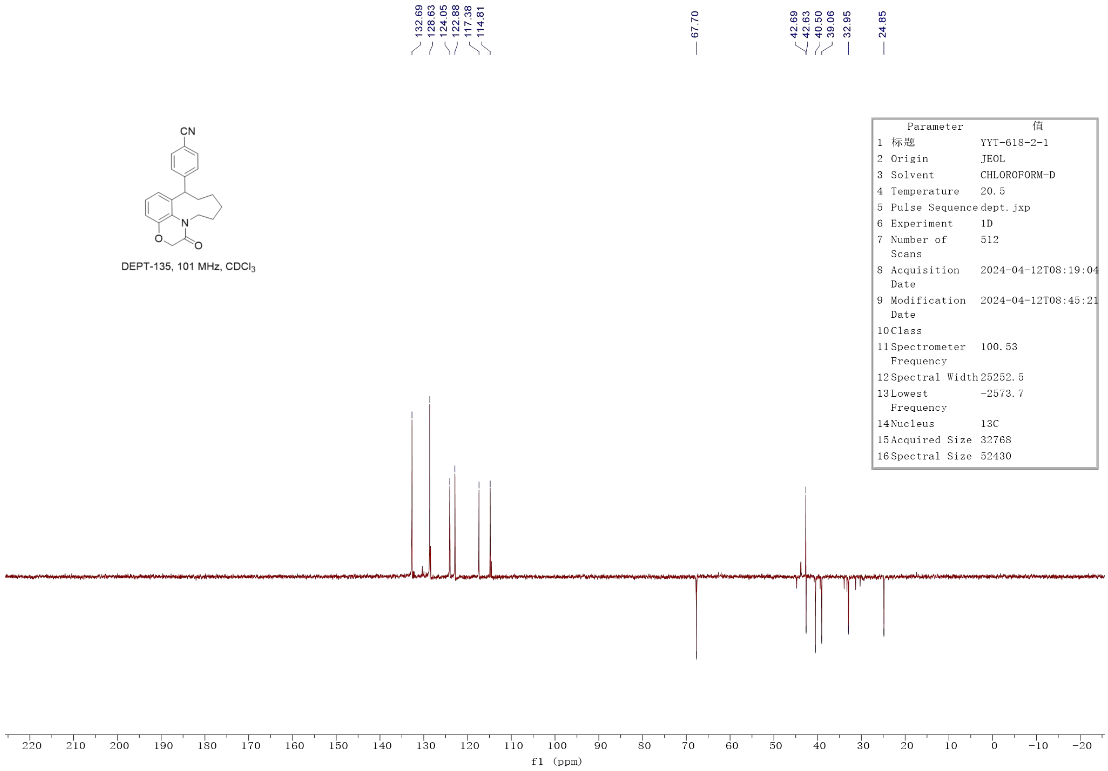
132.69
128.63
124.05
122.88
117.38
114.81
67.70
42.69
42.63
40.50
39.06
32.95
24.85
CN
N
O
O
DEPT-135, 101 MHz, $CDCl_3$
Parameter 値
1 标题 YYT-618-2-1
2 Origin JEOL
3 Solvent CHLOROFORM-D
4 Temperature 20.5
5 Pulse Sequence dept.jxp
6 Experiment 1D
7 Number of Scans 512
8 Acquisition Date 2024-04-12T08:19:04
9 Modification Date 2024-04-12T08:45:21
10 Class
11 Spectrometer Frequency 100.53
12 Spectral Width 25252.5
13 Lowest Frequency -2573.7
14 Nucleus 13C
15 Acquired Size 32768
16 Spectral Size 52430
220 210 200 190 180 170 160 150 140 130 120 110 100 90 80 70 60 50 40 30 20 10 0 −10 −20
f1 (ppm)

4-allyl-2H-benzo[b][1,4]oxazin-3(4H)-one-d1 (92% *d*) (Y4)

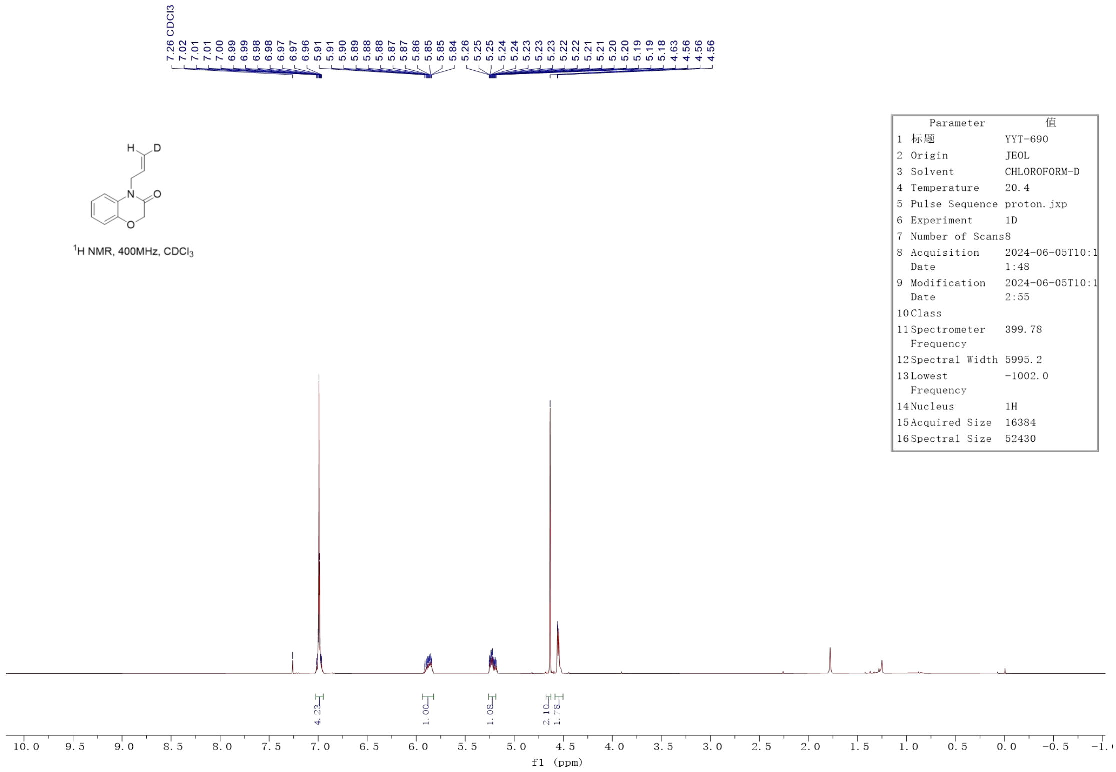

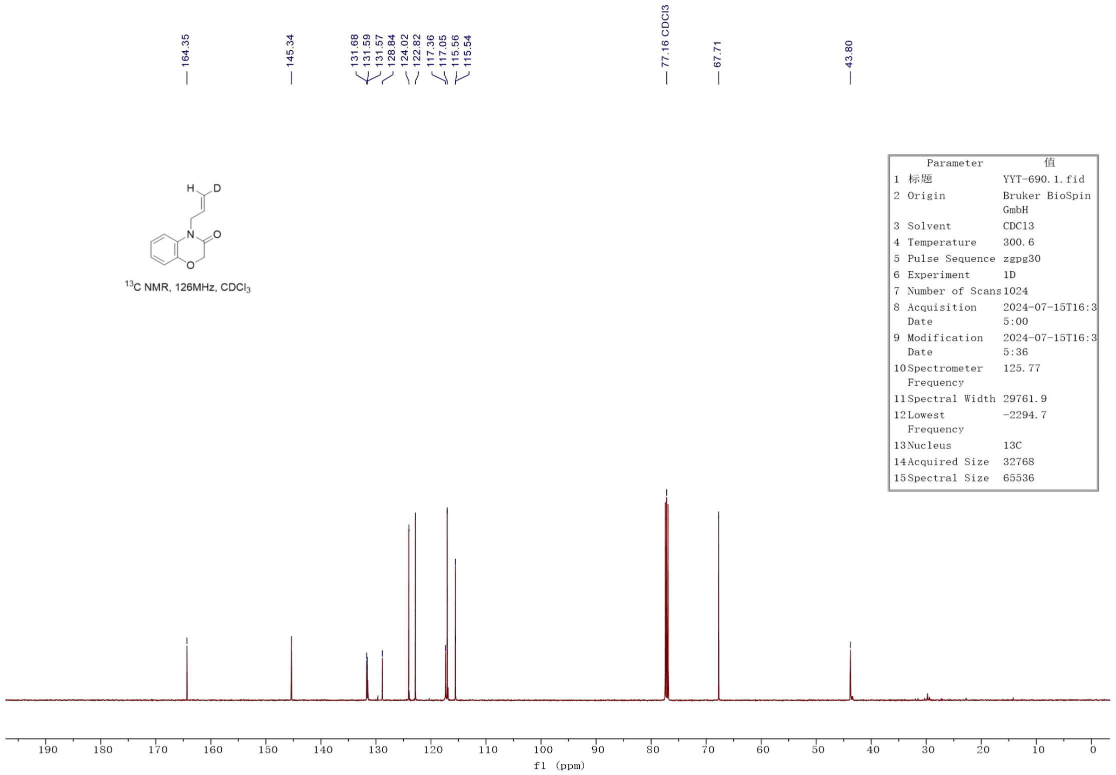

7-(p-tolyl)-6,7-dihydro-5H-[1,4]oxazino[2,3,4-ij]quinolin-3(2H)-one-7-*d* (96% *d*) (103)

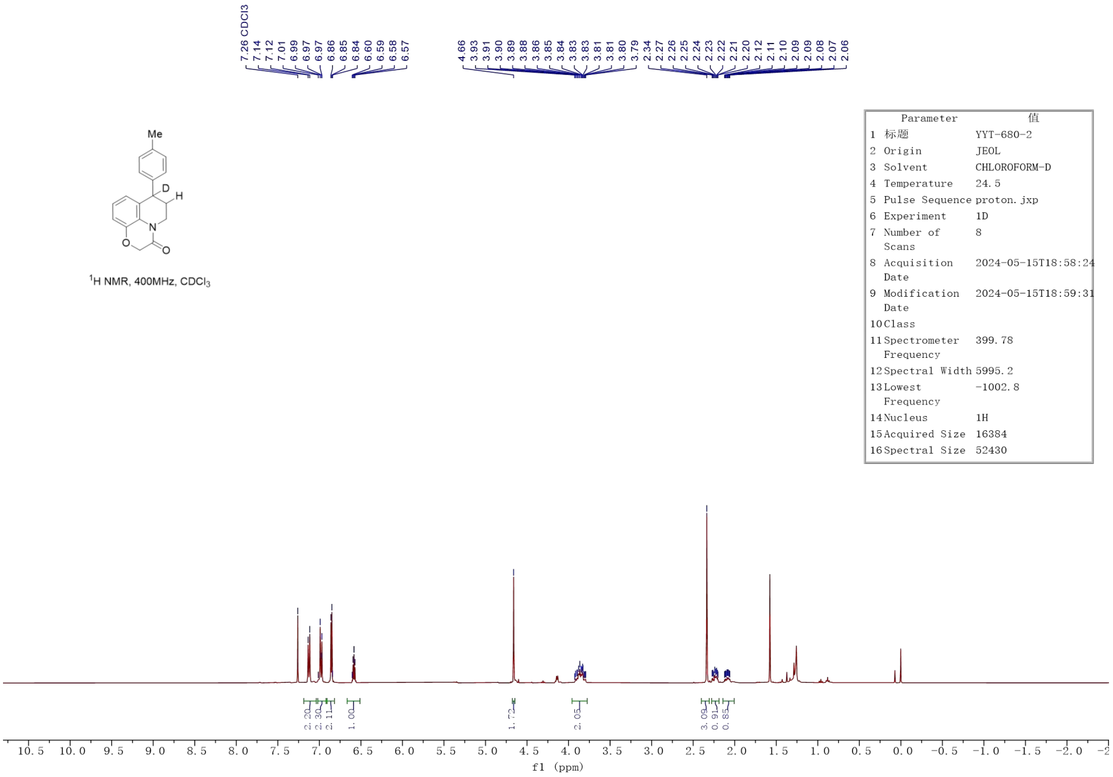

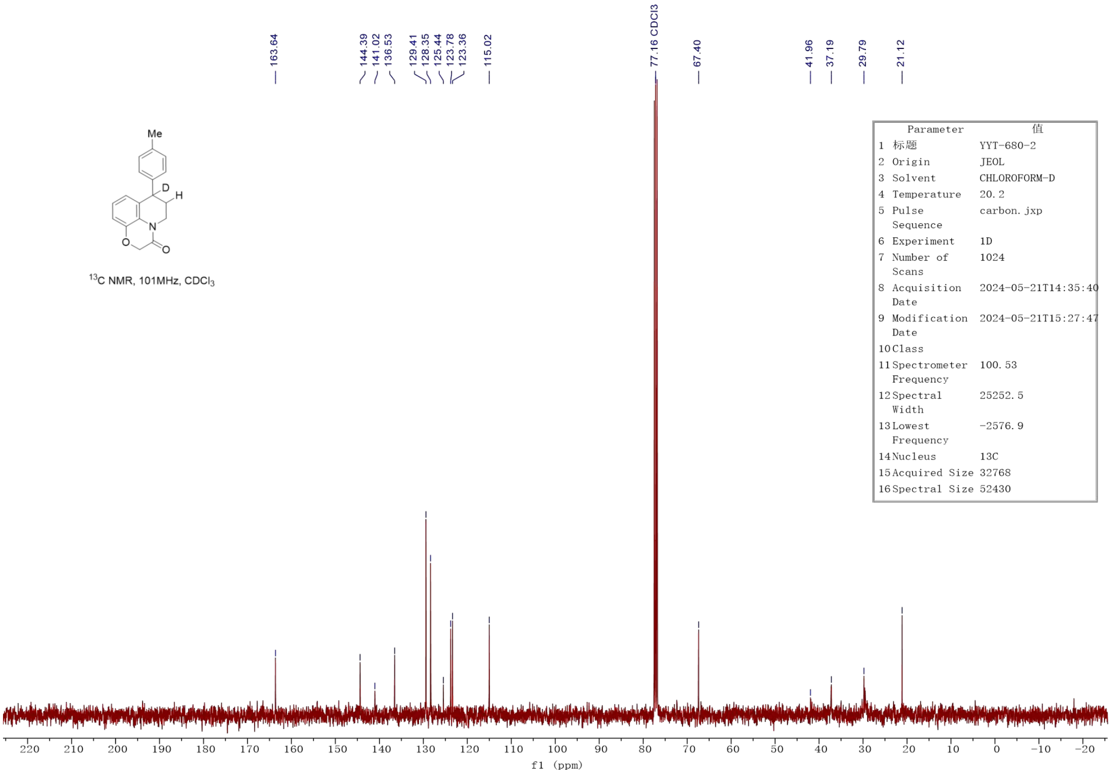

4-(2-methylallyl-3,3-d2)-2H-benzo[b][1,4]oxazin-3(4H)-one (Y6)

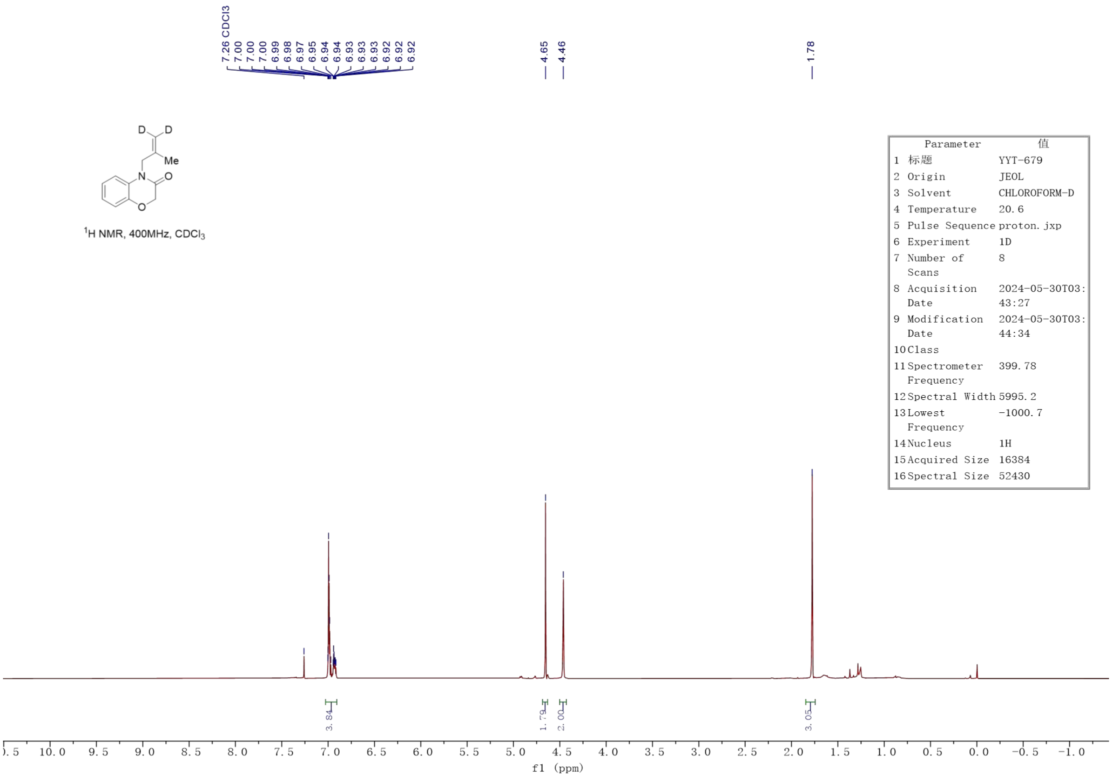

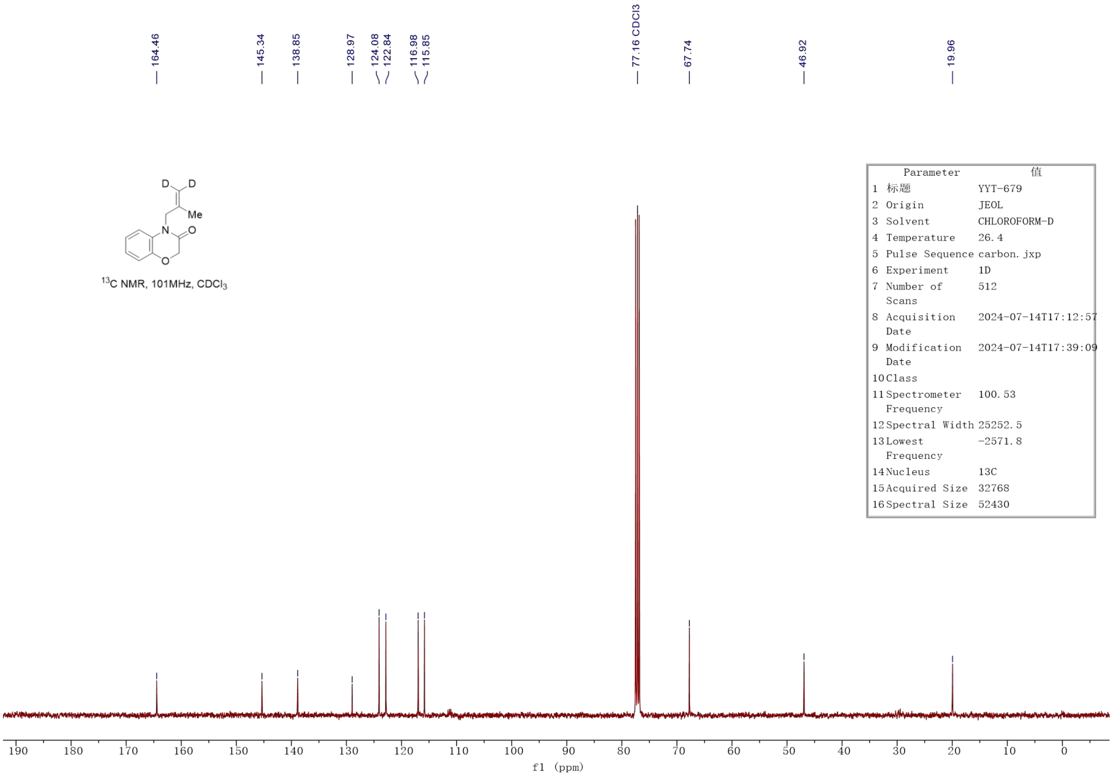

6-methyl-7-(p-tolyl)-6,7-dihydro-5H-[1,4]oxazino[2,3,4-ij]quinolin-3(2H)-one-6,7-*d*2 (104)

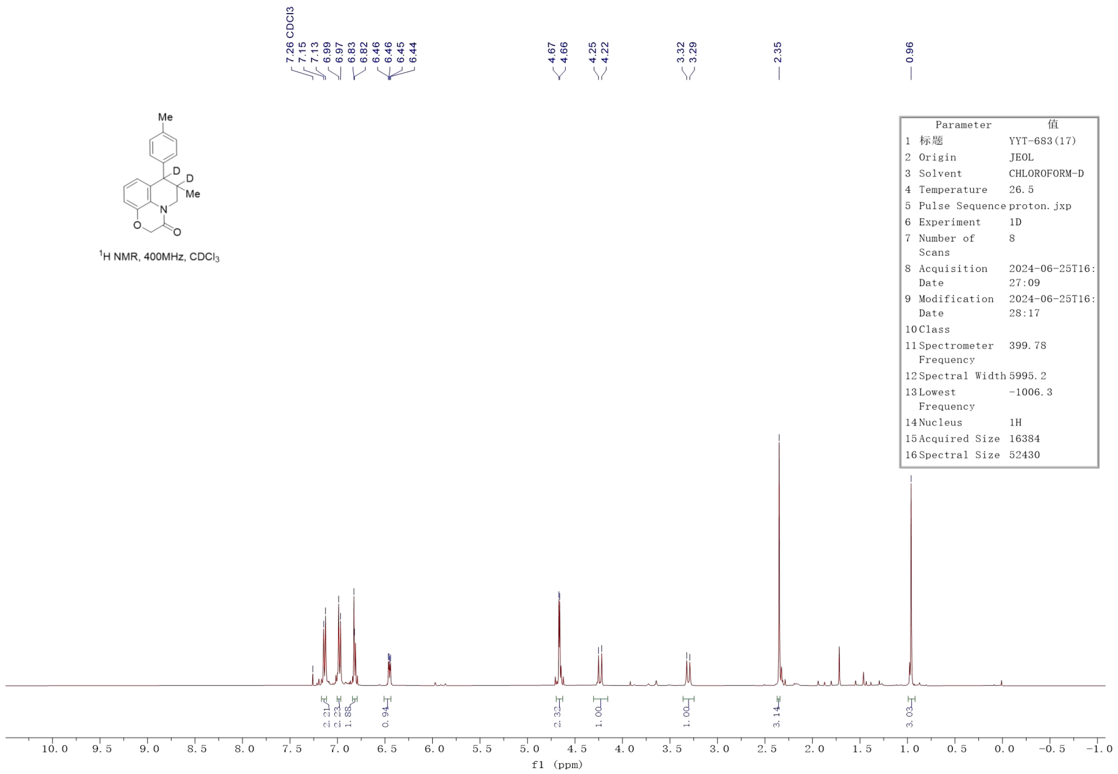

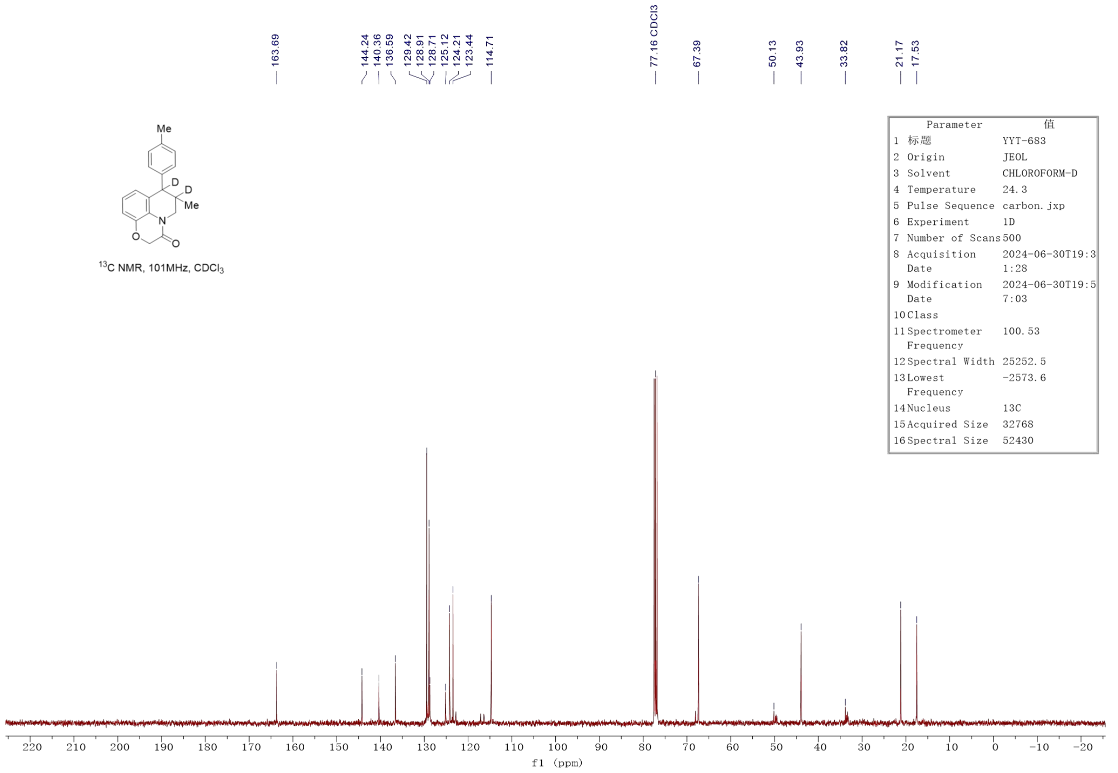

4-(p-tolyl)chromane-5,6,7,8-*d*4 (105)

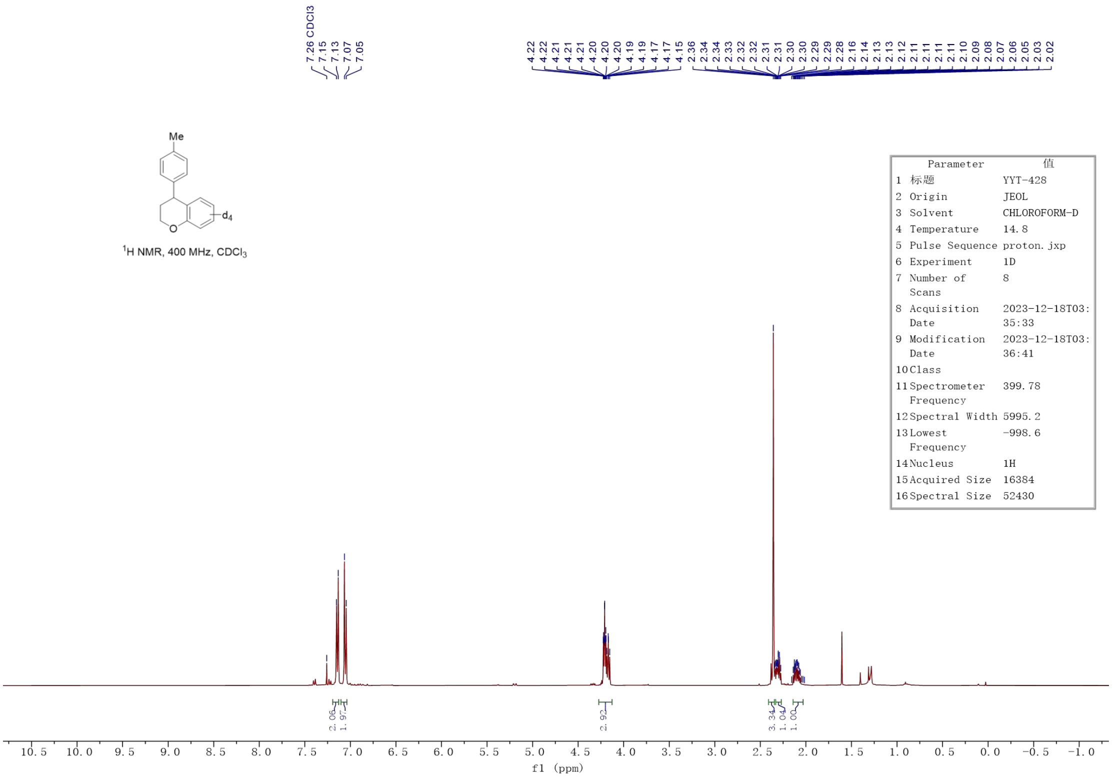

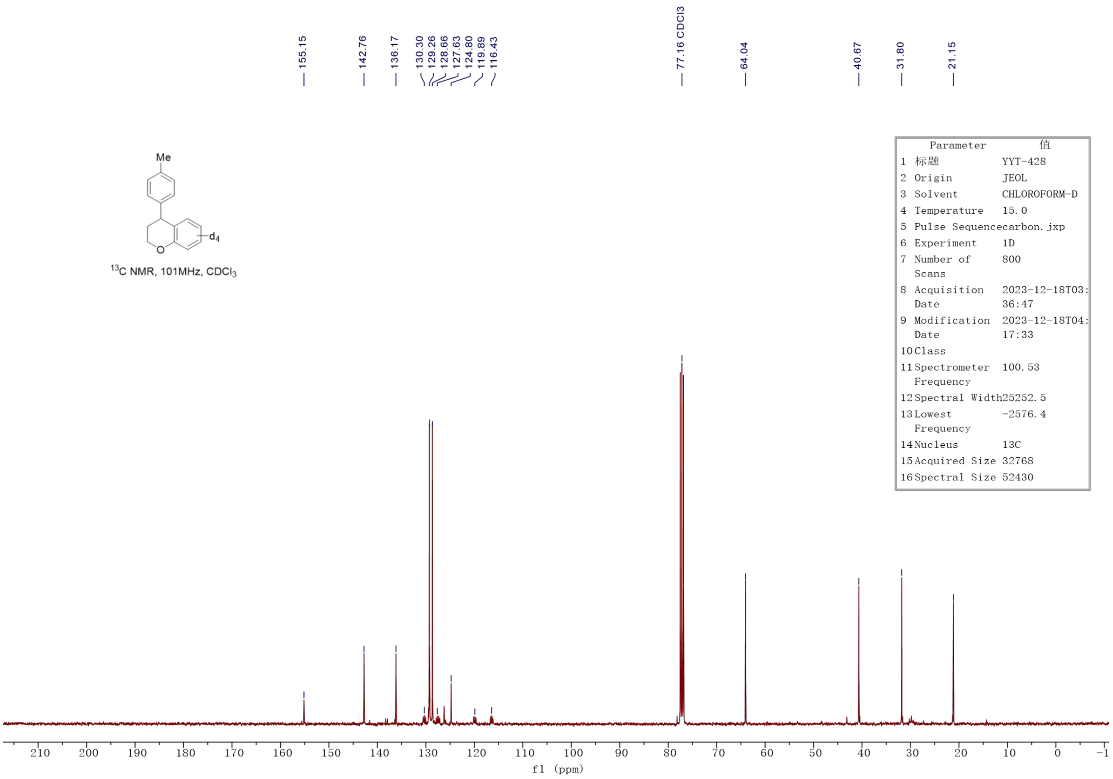

References

[1] X. Huo, M. Quan, G. Yang, X. Zhao, D. Liu, Y. Liu, W. Zhang, *Org. Lett.* **2014**, *16*, 1570-1573.

[2] X. Li, J. Xu, *Tetrahedron*, **2016**, *72*, 5515-5520.

[3] E. Taskinen, *J. Chem. Soc., Perkin Trans.* **2001**, *2*, 1824-1834.

[4] J. Niu, H. Zhou, Z. Li, J. Xu, S. Hu, *J. Org. Chem.* **2008**, *73*, 7814-7817.

[5] W. Sander, S. Roy, I. Polyak, J. M. R. Anguita, E. S. Garcia, *J. Am. Chem. Soc*. **2012**, *134*, 8222-8230.

[6] M. F. Corrêa, R. J. A. Barbosa, L. B. Teixeira, D. A. Duarte, S. C. Simões, L. T. Parreiras-e-Silva, A. M. Balbino, R. G. Landgraf, M. Bouvier, C. M. Costa-Neto, J. P. S. Fernandes, *Front. Pharmacol*. **2017**, *8*, 825/1-825/12.

[7] Y. Akhtar, Y. Yu, M. B. Isman, E. Plettner, *J. Agric. Food. Chem*. **2010**, *58*, 4983-4991.

[8] Q. Gou, B. Yuan, M. Ran, J. Ren, M. Zhang, X. Tan, T. Yuan, X. Zhang, *Org. Lett*. **2021**, *23*, 118-123.

[9] J. C. Sarie, C. Thiehoff, J. Neufeld, C. G. Daniliuc, R. Gilmour, *Angew. Chem. Int. Ed*. **2020**, *59*, 15069-15075.

[10] R. Trivedi, J. A. Tunge, *Org Lett*. **2009**, *11*, 5650-5652.

[11] W. Pan, C. Li, H. Zhu, F. Li, T. Li, W. Zhao, *Org. Biomol. Chem.* **2021**, *19*, 7633-7640.

[12] H. Yin, Z. Meng, B. Liu, *Synthesis.* **2016**, *48*, 3951-3956.

[13] J. Magolan, M. J. Coster, *J. Org. Chem*. **2009**, *74*, 5083-5086.

[14] Y. Hu, Y. Zhong, J. Li, L. Cai, H. Li, *Color. Technol.* **2011**, *127*, 335-339.

[15] D. C. Borda, E. Wimmer, B. Gouilleux, E. Barré, N. Oger, L. Goulamaly, L. Peault, B. Charrier, C. Truchet, P. Giraudeau, M. R. Zubiri, E. L. Grognec, F. X. Felpin, *J. Org. Chem.* **2018**, *83*, 14286-14299.

[16] A. Bochicchio, R. Cefola, S. Choppin, F. Colobert, M. A. D. Noia, M. Funicello, G. Hanquet, I. Pisano, S. Todisco, L. Chiummiento, *Tetrahedron Lett.* **2016**, *57*, 4053-4055.

[17] J. Dai, W. Xu, Y. Wu, W. Zhang, Y. Gong, X. He, X. Zhang, H. Xu, *J. Org. Chem*. **2015**, *80*, 911-919.

[18] P. M. Kathe, A. Berkefeld, I. Fleischer, *Synlett.* **2021**, *32*, 1629-1632.

[19] K. Yuan, T. Feoktistova, P. H. Cheong, R. A. Altman, *Chem. Sci*. **2021**, *12*, 1363-1367.
