# Supplementary material for: Palladium‐Catalyzed Dual Csp2─Csp3 Bond Formation: A Versatile Platform for the Synthesis of Benzo‐Fused Heterocycles
Source: Adv Sci (Weinh). 2025 Apr 28;12(25):2500897. doi: 10.1002/advs.202500897 (PMC12224930; doi:10.1002/advs.202500897)
Supplement: Supplementary file 2 — Supporting Information 2 [file ADVS-12-2500897-s001.pdf]

ADVANCED SCIENCE
Open Access

Supporting Information

for *Adv. Sci.*, DOI 10.1002/advs.202500897

Palladium-Catalyzed Dual Csp^2—Csp^3 Bond Formation: A Versatile Platform for the Synthesis of Benzo-Fused Heterocycles

Jiahui Huang, Yuantao You, Yijian Ma, Xingying He, Yixiao Li, Arunachalam Kesavan, Chengzhi Jin, Chengshuo Shen, Min Zhang* and Kedong Yuan**

General Information of Theoretical Calculations

Density functional theory (DFT) calculations were carried out using Gaussian 09 program[1]. Geometrical optimization calculations were carried out at the PBE0-D3(BJ)[2],[3]/def-TZVP[4] level with the SMD continuum solvent model[5] using eps of 3.12 (for dimethyl carbonate, DMC) without any symmetry assumptions unless otherwise stated. Harmonic vibration frequency calculations were performed at the same level to verify the resulting stationary points as local minima (with all the frequencies real) or saddle points (with only one imaginary frequency). The assignment of the saddle points was performed using intrinsic reaction coordinate (IRC) calculations. In order to improve the relative energy, we further performed the single point calculations on the optimized geometries at the PBE0-D3(BJ)/ma-TZVPP[6] level with the SMD continuum solvent model. Free energies at 298.15 K were converted using the equation: $G_{\text{ma-TZVPP}} = EE_{\text{ma-TZVPP}} + G_{\text{corr,def-TZVP}}$. Where $EE_{\text{ma-TZVPP}}$ refers to the single point electronic energy calculated at the PBE0-D3(BJ)/ma-TZVPP level on the optimized geometry that has been calculated at the PBE0-D3(BJ)def-TZVP level. $G_{\text{corr,def-TZVP}}$ refers to the thermal correction to free energy at the PBE0-D3(BJ)/def-TZVP level at 298.15 K.

Cartesian coordinates (Å)

A

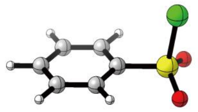

S	1.215962	0.002535	-0.515576	C	-2.550299	-1.207817	0.133458	H	-3.084971	-2.146344	0.218799
O	1.623194	-1.264313	-1.106848	C	-3.228116	-0.001385	0.254080	H	-3.086373	2.143865	0.231969
O	1.622931	1.274990	-1.094866	C	-2.551149	1.206194	0.140863	H	-0.640989	2.150273	-0.184699
C	-0.531152	0.000945	-0.208429	C	-1.185144	1.218532	-0.091680	Cl	1.982687	-0.006748	1.446089
C	-1.184338	-1.217817	-0.099228	H	-0.639614	-2.148628	-0.198198	H	-4.296932	-0.002336	0.437165

B

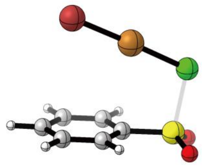

S	-2.529359	-0.268228	0.177342	C	0.426805	3.093347	-0.137905	H	-1.612811	1.206072	-2.084518
O	-2.916499	-0.521829	1.552338	C	-0.202245	2.623726	-1.284705	Cl	-1.194298	-1.988419	-0.326193
O	-3.479472	-0.267636	-0.919520	C	-1.114443	1.587194	-1.201686	Cu	0.880398	-1.154123	-0.115916
C	-1.377919	1.058560	0.056473	H	-0.978981	1.051367	2.177714	H	1.149513	3.897177	-0.216323
C	-0.755986	1.500348	1.217990	H	0.650629	2.910196	1.991679	Br	2.944562	-0.247922	0.072391
C	0.148474	2.542076	1.105577	H	0.025746	3.058397	-2.250339				

C

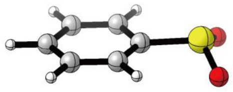

S	1.689202	-0.000009	-0.253372	C	-2.149235	1.206283	0.034537	H	-2.690014	2.144834	0.070796
O	2.206617	1.294314	0.265313	C	-2.836943	-0.000002	0.076618	H	-2.690016	-2.144830	0.070715
O	2.206590	-1.294309	0.265404	C	-2.149218	-1.206289	0.034493	H	-0.213591	-2.148144	-0.085903
C	-0.100371	0.000014	-0.079875	C	-0.765994	-1.216852	-0.058436	H	-3.919073	-0.000022	0.144799
C	-0.766000	1.216862	-0.058398	H	-0.213628	2.148174	-0.085839				

D

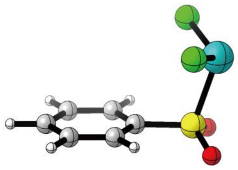

O	0.086055	1.276076	2.069112	C	-3.351463	-1.208548	-0.606113	S	-0.087281	0.001060	1.366016
O	0.087245	-1.274479	2.068111	C	-2.155276	-1.221798	0.091153	Pd	1.613177	-0.000581	-0.135680
C	-1.595801	0.000656	0.434237	H	-1.670941	2.152275	0.359952	Cl	1.205233	2.037004	-1.082564
C	-2.153599	1.222715	0.086910	H	-3.817131	2.146169	-0.886904	Cl	1.203803	-2.037772	-1.082993
C	-3.349672	1.208687	-0.610503	H	-3.820406	-2.146353	-0.878894	H	-4.877038	-0.000450	-1.504367
C	-3.942573	-0.000142	-0.954939	H	-1.673846	-2.151030	0.367427				

E

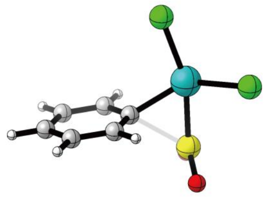

O	-0.176037	2.594132	0.975607	C	3.347255	-0.443939	-0.798099	S	-0.160080	1.805543	-0.247948
O	0.158050	2.436986	-1.524247	C	2.019496	-0.130426	-1.044730	Pd	-0.845647	-0.331452	-0.264707
C	1.182307	0.032321	0.053099	H	0.923902	0.048665	2.197445	Cl	-2.834610	0.117400	0.852198
C	1.609199	-0.091652	1.369676	H	3.303807	-0.512491	2.603270	Cl	-0.844172	-2.606839	-0.342273
C	2.940099	-0.403947	1.588256	H	4.028210	-0.573741	-1.631070	H	4.841261	-0.820758	0.690429
C	3.799786	-0.580465	0.508902	H	1.658196	-0.003411	-2.058618				

F

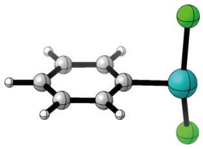

Pd	-1.077891	-0.000033	-0.250063	C	1.585364	-0.000570	-1.236248	C	3.599423	-0.000091	0.089844
Cl	-1.292704	-2.178602	0.314475	C	2.844047	0.000598	1.255744	H	3.330856	0.001047	2.224664
Cl	-1.292580	2.178693	0.313702	H	0.859194	0.001154	2.100327	H	3.561732	-0.001159	-2.061095
C	0.874321	0.000044	-0.054240	C	2.975551	-0.000652	-1.148866	H	4.681903	-0.000196	0.148584
C	1.454797	0.000706	1.196389	H	1.098126	-0.001095	-2.204329				

G

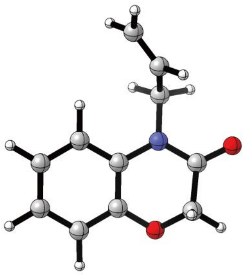

N	-0.768170	0.387643	-0.296237	C	2.871164	-0.084542	0.042540	H	-2.899407	-2.912763	-0.474490
C	-1.947985	-0.344833	-0.739268	C	2.975859	-1.468387	0.072370	H	0.311171	3.437724	0.238441
C	-2.692442	-0.997422	0.383368	C	1.829634	-2.247249	0.024010	H	0.421117	2.271177	1.576772
C	-3.107817	-2.255587	0.366182	C	0.579044	-1.651211	-0.067505	H	3.747878	0.551604	0.082874
C	-0.899146	1.712044	0.001140	O	-1.952350	2.316571	-0.075001	H	3.952752	-1.932466	0.142634
C	0.366890	2.379531	0.483927	H	-2.595569	0.390184	-1.222125	H	1.900494	-3.328127	0.060071
O	1.539080	1.866339	-0.122026	H	-1.643413	-1.076129	-1.490164	H	-0.310021	-2.266628	-0.095901
C	1.626116	0.510768	-0.046282	H	-2.915643	-0.357768	1.234379				
C	0.464389	-0.266203	-0.118658	H	-3.680250	-2.675040	1.186431				

H

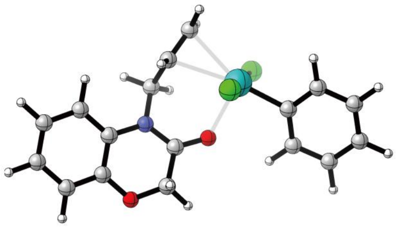

N	2.125228	-0.146309	-0.545677	O	0.054361	0.777384	-0.735820	Pd	-1.353018	-0.772699	0.117421
C	1.621347	-1.476298	-0.868637	H	0.983075	-1.400329	-1.751505	C	-2.875431	0.516473	0.110713
C	0.873602	-2.076049	0.281258	H	2.478269	-2.093648	-1.140148	C	-3.995962	0.118104	0.810011
C	-0.095657	-2.993246	0.157207	H	1.192014	-1.765773	1.272357	C	-2.807738	1.703944	-0.583627
C	1.270971	0.884803	-0.538193	H	-0.536671	-3.445599	1.039426	C	-5.106870	0.957485	0.807998
C	1.858888	2.237235	-0.251347	H	-0.411769	-3.367758	-0.809683	H	-4.021698	-0.816109	1.360607
O	3.196876	2.358992	-0.691506	H	1.269589	2.989432	-0.771774	C	-3.926129	2.533579	-0.569521
C	3.991553	1.345940	-0.251027	H	1.779886	2.413883	0.831275	H	-1.917136	1.981653	-1.131310
C	3.471266	0.053180	-0.155746	H	5.695235	2.604113	-0.051387	C	-5.070871	2.162847	0.121612
C	5.318467	1.592919	0.047718	H	7.173087	0.745033	0.702380	H	-5.995948	0.661260	1.354298
C	6.133326	0.550643	0.466759	H	6.242389	-1.541714	0.942310	H	-3.895192	3.471978	-1.113168
C	5.614620	-0.729074	0.596874	H	3.887611	-1.980875	0.404045	H	-5.937608	2.814496	0.125510
C	4.284196	-0.979691	0.290820	Cl	-0.874456	-0.160616	2.331022	Cl	-2.066148	-1.498768	-1.993579

I

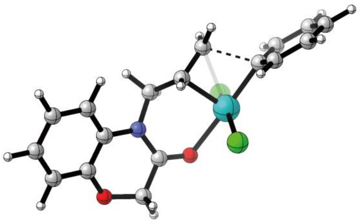

N	-2.038298	-0.196985	-0.431347	O	-0.676136	-1.668659	0.643942	Pd	1.189538	-0.555771	0.245030
C	-1.012054	0.136627	-1.410326	H	-0.641722	-0.781381	-1.872492	C	2.899410	0.533379	-0.171498
C	0.116842	0.927204	-0.816441	H	-1.489248	0.726480	-2.194823	C	3.207189	1.598294	0.678553
C	1.340984	1.125200	-1.482000	H	-0.177738	1.674643	-0.083472	C	3.900853	-0.095912	-0.909182
C	-1.782130	-1.151697	0.483065	H	1.802990	2.101108	-1.438873	C	4.527737	1.981056	0.837336
C	-2.934397	-1.558377	1.355386	H	1.570472	0.519895	-2.352208	H	2.423688	2.103636	1.227241
O	-4.185112	-1.436282	0.705570	H	-2.805756	-2.602365	1.633784	C	5.220189	0.300905	-0.737460
C	-4.372724	-0.205530	0.157124	H	-2.899744	-0.942379	2.265699	H	3.650831	-0.899229	-1.589132
C	-3.291249	0.461892	-0.422457	H	-6.457858	-0.198380	0.579202	C	5.537795	1.332994	0.133747
C	-5.637687	0.352848	0.134424	H	-6.821152	2.033956	-0.466306	H	4.766770	2.793766	1.514450
C	-5.829673	1.596854	-0.449481	H	-4.894058	3.263631	-1.430972	H	6.000345	-0.204584	-1.296091
C	-4.753530	2.282503	-0.993269	H	-2.649450	2.269313	-1.392400	H	6.570106	1.640086	0.259855
C	-3.484609	1.720461	-0.974950	Cl	0.690059	0.514162	2.372495	Cl	1.578952	-2.263956	-1.483489

J

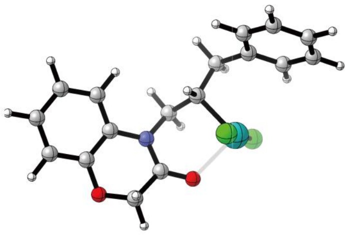

N	-1.963152	-0.258788	-0.462628	C	-4.264701	0.036232	0.175319	H	-0.659149	-0.858301	-1.974270
C	-0.879413	0.036810	-1.390621	C	-3.093083	0.587376	-0.348152	H	-1.238052	0.794510	-2.091588
C	0.352792	0.554400	-0.702997	C	-5.424634	0.785426	0.246004	H	0.110864	1.190819	0.148380
C	1.421361	1.159346	-1.570898	C	-5.417036	2.104410	-0.184752	H	1.022317	2.111457	-1.949135
C	-1.861091	-1.339688	0.325162	C	-4.247440	2.670224	-0.670150	H	1.632470	0.519776	-2.428828
C	-3.056925	-1.660336	1.175062	C	-3.084569	1.916581	-0.748082	H	-3.096718	-2.736886	1.328313
O	-4.273496	-1.265466	0.572831	O	-0.845921	-2.042390	0.393117	H	-2.919228	-1.169002	2.149067

H	-6.320919	0.322175	0.641410	C	2.669538	1.415354	-0.766421	H	3.684382	-0.339967	-1.471894
H	-6.325765	2.692099	-0.128564	C	2.746557	2.513679	0.086606	C	4.903563	1.787986	0.866943
H	-4.231453	3.705749	-0.988309	C	3.730008	0.510698	-0.800600	H	3.899449	3.557040	1.560034
H	-2.176769	2.373781	-1.121055	C	3.854518	2.700085	0.896903	H	5.657830	-0.015553	-0.021230
Cl	0.935455	-0.037835	2.312139	H	1.926021	3.223685	0.123704	H	5.766694	1.931331	1.507487
Pd	1.175718	-1.068093	0.235752	C	4.841161	0.697199	0.014480	Cl	1.696237	-2.241621	-1.738028

K

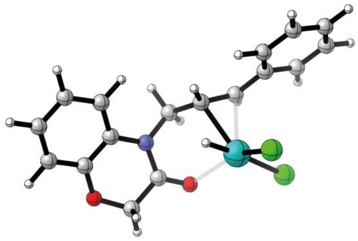

N	-2.163795	0.022523	-0.681015	O	-1.080723	-1.963868	-0.640019	C	3.852705	0.857854	-1.324848
C	-1.035903	0.629336	-1.365368	H	-0.746074	-0.041196	-2.176012	C	2.781797	2.263235	0.317999
C	0.169522	0.916771	-0.506762	H	-1.380762	1.561506	-1.815920	C	5.077691	1.414403	-1.004511
C	1.434219	0.653793	-1.046357	H	0.065807	1.702492	0.235166	H	3.786552	0.075203	-2.072701
C	-2.100785	-1.301342	-0.438033	H	1.458051	0.084141	-1.973128	C	4.006544	2.818031	0.634002
C	-3.341144	-1.926056	0.136804	H	-3.402677	-2.955694	-0.210284	H	1.896437	2.594479	0.846988
O	-4.526358	-1.266140	-0.265463	H	-3.246673	-1.929697	1.232363	C	5.157046	2.394320	-0.023772
C	-4.484929	0.079332	-0.064518	H	-6.553836	0.199438	0.418626	H	5.973553	1.077742	-1.513305
C	-3.288047	0.770136	-0.266575	H	-6.499460	2.671080	0.723006	H	4.070635	3.580514	1.401878
C	-5.636792	0.760722	0.283027	H	-4.366056	3.899143	0.423857	H	6.117123	2.828183	0.232947
C	-5.596582	2.138182	0.448798	H	-2.318554	2.686244	-0.201279	Cl	2.297892	-2.679571	-0.647316
C	-4.404181	2.826235	0.278177	Pd	0.863378	-1.049211	0.321368	H	-0.100232	-0.094435	1.030040
C	-3.248004	2.143945	-0.074592	C	2.689795	1.273551	-0.667053	Cl	2.016006	-0.630295	2.338331

L

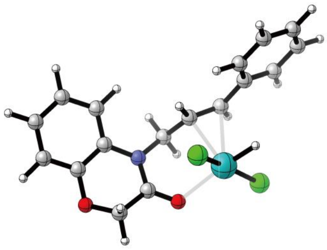

N	-1.954986	-0.061717	-0.690536	C	-2.831637	2.231865	-0.551217	H	-1.856977	2.650585	-0.770037
C	-0.769014	0.314906	-1.442292	O	-1.076167	-2.095163	-0.228699	Cl	0.157557	-0.541803	2.385056
C	0.394820	0.705206	-0.581107	H	-0.500702	-0.510928	-2.104306	Pd	1.001160	-1.373231	0.365030
C	1.674242	0.679834	-1.067984	H	-1.045596	1.160157	-2.076627	C	2.839975	1.323963	-0.493728
C	-2.019289	-1.304131	-0.178873	H	0.159294	1.273634	0.312227	C	4.048620	1.238144	-1.193836
C	-3.306283	-1.674938	0.498369	H	1.837585	0.181878	-2.020522	C	2.809358	2.014570	0.725337
O	-4.430274	-1.005795	-0.042382	H	2.349922	-1.037385	0.904847	C	5.194013	1.839966	-0.700743
C	-4.257969	0.341107	-0.128180	H	-3.467931	-2.744152	0.375456	H	4.080756	0.686166	-2.127325
C	-3.001443	0.857618	-0.453450	H	-3.193612	-1.455064	1.569854	C	3.955891	2.608123	1.216625
C	-5.338347	1.184994	0.052029	H	-6.302982	0.751918	0.289426	H	1.892663	2.071363	1.300759
C	-5.167919	2.556686	-0.073039	H	-6.014851	3.217343	0.070655	C	5.149702	2.526588	0.504805
C	-3.916389	3.078033	-0.365272	H	-3.774681	4.149198	-0.445377	H	6.123770	1.767282	-1.253348

H	3.924532	3.135576	2.163233	H	6.045930	2.994696	0.896595	Cl	2.117500	-2.576970	-1.295594

M

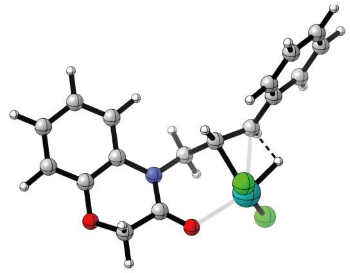

N	-1.935553	0.005237	-0.430993	O	-1.220644	-2.029142	0.322404	Pd	0.964178	-1.539655	0.175899
C	-0.734452	0.237646	-1.224999	H	-0.592107	-0.593843	-1.919418	C	2.896734	1.262699	-0.390905
C	0.478697	0.443628	-0.367626	H	-0.909823	1.130884	-1.827341	C	3.958432	1.517940	-1.261620
C	1.795924	0.419793	-0.882773	H	0.302987	1.005282	0.545434	C	2.915324	1.813869	0.892943
C	-2.092026	-1.165764	0.222404	H	1.907095	0.063548	-1.904799	C	5.003668	2.339123	-0.870747
C	-3.426536	-1.376762	0.881350	H	2.343949	-0.906200	-0.171025	H	3.955848	1.074921	-2.252081
O	-4.484912	-0.731946	0.201350	H	-3.638781	-2.443767	0.888492	C	3.966874	2.624466	1.281223
C	-4.236684	0.587784	-0.014749	H	-3.352575	-1.024236	1.920109	H	2.127800	1.579475	1.599890
C	-2.939277	1.000920	-0.325548	H	-6.272293	1.141375	0.258294	C	5.009226	2.895251	0.400907
C	-5.275249	1.499704	0.030624	H	-5.835860	3.556739	-0.174605	H	5.817645	2.539218	-1.558255
C	-5.021997	2.842105	-0.211014	H	-3.521784	4.316815	-0.648180	H	3.978464	3.043133	2.281152
C	-3.729445	3.266729	-0.480634	H	-1.682526	2.699247	-0.733927	H	5.829692	3.532451	0.711604
C	-2.687893	2.350851	-0.532154	Cl	1.060536	-0.924892	2.487952	Cl	1.239199	-2.792681	-1.850297

N

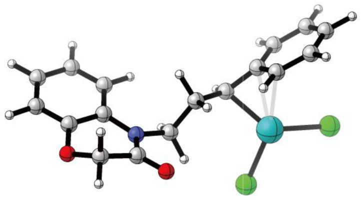

N	2.707963	-0.509051	-0.167757	O	2.047178	-2.030455	-1.719084	C	-2.772059	2.319299	0.501223
C	1.380426	-0.474820	0.419499	H	0.977731	-1.486079	0.392133	C	-2.298997	1.090425	-1.553544
C	0.455181	0.473328	-0.332251	H	1.474342	-0.188740	1.467652	C	-3.922749	2.801164	-0.063475
C	-0.811251	0.790811	0.395185	H	0.963237	1.440105	-0.465938	H	-2.498811	2.578182	1.517859
C	2.919582	-1.338081	-1.230504	H	-0.685826	1.007785	1.454986	C	-3.488364	1.570312	-2.098110
C	4.316678	-1.299365	-1.801861	H	4.541432	-2.277479	-2.222135	H	-1.631218	0.496665	-2.167423
O	5.316685	-1.012886	-0.841584	H	4.337399	-0.555214	-2.611227	C	-4.289830	2.415710	-1.360410
C	5.040314	0.087921	-0.092646	H	7.091998	0.618257	0.074871	H	-4.568278	3.462448	0.502432
C	3.716762	0.371416	0.262586	H	6.630020	2.595989	1.520932	H	-3.775176	1.275323	-3.100131
C	6.079576	0.879890	0.360037	H	4.285406	3.139312	2.131935	H	-5.215915	2.786705	-1.784563
C	5.813410	1.977570	1.167124	H	2.444048	1.729174	1.337902	Cl	-1.368043	-2.517508	1.385377
C	4.504373	2.279865	1.509076	Pd	-2.206984	-0.743446	0.193555	Cl	-4.519353	-0.773972	0.652233
C	3.460197	1.483845	1.057199	C	-1.920625	1.463204	-0.238328	H	0.274221	0.086382	-1.337361

O

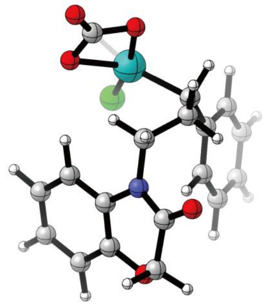

N	-1.571741	-1.394069	-0.826967	H	0.425903	-1.856552	-0.478726	H	1.900544	3.272376	-1.172096
C	-0.209547	-1.587142	-1.315477	H	-0.239905	-2.442474	-1.990983	C	-1.885440	3.026976	-0.500262
C	0.396284	-0.416374	-2.096068	H	1.188621	-0.867785	-2.693582	H	-1.671212	0.935891	-0.869985
C	1.023729	0.772304	-1.387546	H	1.917570	1.090312	-1.933504	C	-1.251004	4.259917	-0.427310
C	-2.578520	-1.521587	-1.736079	H	-4.620361	-0.999440	-1.967258	H	0.618572	5.303534	-0.629926
C	-3.972289	-1.369812	-1.175651	H	-4.332171	-2.357937	-0.853911	H	-2.950332	2.948948	-0.308849
O	-4.037706	-0.445064	-0.107504	H	-4.470035	-0.079880	2.403587	H	-1.815808	5.151063	-0.175581
C	-3.139045	-0.730040	0.879983	H	-2.826854	-0.508337	4.235542	H	-0.355197	-0.040837	-2.799237
C	-1.872417	-1.222850	0.543686	H	-0.578953	-1.403007	3.662784	H	0.032876	-1.836060	1.352058
C	-3.482027	-0.473710	2.193838	Pd	2.136564	0.145539	0.255808	O	2.333144	-1.894143	0.891310
C	-2.563041	-0.715866	3.204886	C	0.211146	1.950248	-1.050444	C	2.985036	-2.189028	-0.216913
C	-1.312470	-1.220769	2.886463	C	0.832338	3.205317	-0.990825	O	3.084551	-1.142816	-1.010937
C	-0.964562	-1.473739	1.566811	C	-1.164021	1.887424	-0.814745	O	3.434339	-3.291916	-0.473324
O	-2.392333	-1.765035	-2.915547	C	0.114165	4.344504	-0.679890	Cl	1.418303	1.524178	1.991914

P

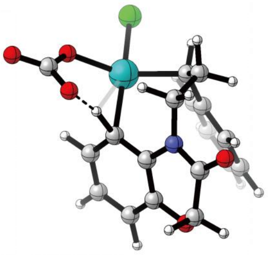

N	-1.414070	-1.535820	-0.884277	H	0.544696	-1.805035	-1.520890	H	0.616641	3.234504	0.069123
C	-0.390565	-1.406240	-1.910901	H	-0.704525	-2.041644	-2.737062	C	-3.117699	2.289649	-0.027303
C	-0.200220	0.014105	-2.437034	H	0.584093	-0.058313	-3.194872	H	-2.546649	0.716244	-1.346732
C	0.192235	1.112605	-1.476569	H	0.861410	1.817342	-1.966126	C	-2.703392	3.369031	0.741246
C	-2.641316	-2.020022	-1.236081	H	-4.615858	-2.169219	-0.471308	H	-1.018185	4.555325	1.356378
C	-3.599913	-2.210993	-0.084880	H	-3.424675	-3.205136	0.351495	H	-4.168290	2.023146	-0.070324
O	-3.483472	-1.207099	0.904298	H	-2.842042	-0.468623	3.273495	H	-3.425997	3.948317	1.305787
C	-2.200654	-1.032831	1.330408	H	-0.520542	-0.102013	4.115401	H	-1.110971	0.318206	-2.968588
C	-1.131650	-1.198222	0.444790	H	1.391961	-0.507342	2.598050	H	1.062970	-1.715408	0.382434
C	-1.982390	-0.618192	2.629795	Pd	1.569404	0.442013	-0.065357	Cl	3.006975	2.206687	-0.684408
C	-0.687344	-0.409387	3.089448	C	-0.832165	1.853961	-0.710807	O	3.189251	-0.770209	0.323301
C	0.376004	-0.619558	2.232800	C	-0.434857	2.966974	0.050089	C	3.128139	-2.104213	0.408664
C	0.183351	-0.981823	0.886837	C	-2.195915	1.541069	-0.741100	O	2.026674	-2.672449	0.079775
O	-2.947912	-2.322983	-2.374688	C	-1.355149	3.707028	0.770169	O	4.148499	-2.671455	0.804517

Q

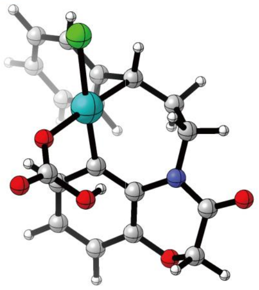

N	-2.123582	-0.469343	1.171977	H	-0.776823	0.740526	2.239879	H	3.773527	-0.072129	1.085184
C	-1.158268	-0.286270	2.249089	H	-1.731320	-0.406608	3.166323	C	2.533731	-3.090256	-0.984179
C	-0.007665	-1.289312	2.180969	H	0.206785	-1.637704	3.197572	H	0.662587	-2.843646	-0.001581
C	1.249578	-0.690551	1.622250	H	1.791732	-0.081449	2.342868	C	3.840376	-2.625505	-1.144383
C	-3.446194	-0.455877	1.502147	H	-5.307803	-1.147504	0.762289	H	5.299871	-1.177119	-0.511878
C	-4.376765	-0.759657	0.354877	H	-4.584898	0.165030	-0.202001	H	2.185122	-3.934028	-1.569614
O	-3.842510	-1.748323	-0.506032	H	-3.039619	-2.237549	-2.899835	H	4.504174	-3.107180	-1.853877
C	-2.625524	-1.377738	-1.000429	H	-0.818631	-1.559757	-3.841138	H	-0.335284	-2.165031	1.619171
C	-1.720500	-0.692421	-0.170455	H	0.748784	-0.278706	-2.444425	H	-1.584203	1.643687	-0.588722
C	-2.308419	-1.705624	-2.302408	Pd	0.914162	0.829720	0.200519	Cl	2.517530	2.262559	1.363157
C	-1.076569	-1.323027	-2.814548	C	2.103571	-1.372280	0.679483	O	0.466105	2.411486	-1.123564
C	-0.195438	-0.606240	-2.022915	C	3.440465	-0.926218	0.507005	C	-0.608360	3.019230	-1.458082
C	-0.491679	-0.282260	-0.697608	C	1.677137	-2.479785	-0.096618	O	-1.787953	2.500979	-0.984218
O	-3.868154	-0.221493	2.622053	C	4.286198	-1.542994	-0.392247	O	-0.676544	4.016685	-2.154899

R

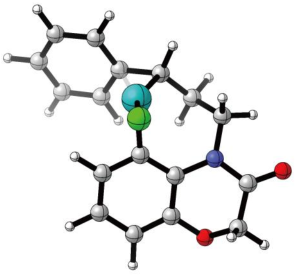

N	-2.174785	0.344966	0.860714	O	-4.051597	1.268650	1.762161	C	2.126527	-1.583619	1.131916
C	-1.340711	1.049662	1.828268	H	-1.018421	2.010730	1.414620	C	4.517612	-0.321386	0.420350
C	-0.132146	0.234724	2.289137	H	-2.001584	1.271108	2.664482	H	3.546544	1.516717	0.942809
C	1.111403	0.675572	1.586783	H	0.005134	0.398909	3.364133	C	3.206142	-2.327418	0.717473
C	-3.526677	0.493667	0.983703	H	1.408751	1.691335	1.863742	H	1.198720	-2.076715	1.391969
C	-4.331446	-0.404888	0.079384	H	-5.315273	-0.546074	0.520995	C	4.403561	-1.706154	0.352219
O	-3.730942	-1.683969	-0.042101	H	-4.441404	0.063353	-0.908334	H	5.448356	0.162849	0.147857
C	-2.460523	-1.600327	-0.522218	H	-2.663351	-3.400083	-1.636575	H	3.124044	-3.407386	0.667927
C	-1.647822	-0.525571	-0.118498	H	-0.333677	-3.264380	-2.544245	H	5.244426	-2.304707	0.021076
C	-1.998060	-2.587511	-1.369996	H	1.079059	-1.329366	-1.976672	Cl	0.184544	2.290031	-2.291680
C	-0.708071	-2.502926	-1.869427	Pd	0.909342	1.046297	-0.456243	H	-0.327495	-0.830071	2.158062
C	0.089516	-1.416447	-1.542032	C	2.213553	-0.169229	1.206710				
C	-0.377919	-0.432053	-0.679906	C	3.448895	0.439134	0.850873				

S

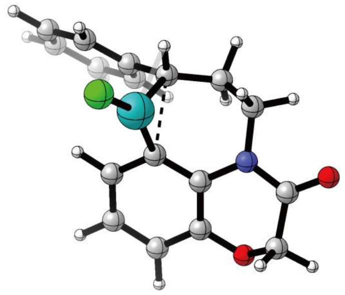

N	2.228736	-0.109476	-0.867134
C	1.444209	0.309895	-2.017554
C	0.179059	-0.518325	-2.136700
C	-0.908142	-0.024385	-1.220038
C	3.590209	-0.093840	-0.943462
C	4.282770	-0.628368	0.288195
O	3.586888	-1.714916	0.879306
C	2.284402	-1.431422	1.143805
C	1.580071	-0.580597	0.282249
C	1.660002	-2.029591	2.222378
C	0.314977	-1.782249	2.460854
C	-0.393035	-0.924177	1.633403
C	0.247541	-0.288474	0.573259
O	4.203154	0.329279	-1.904295
H	1.200669	1.377391	-1.937201
H	2.082434	0.186852	-2.890976
H	-0.194281	-0.442142	-3.163397
H	-1.279451	0.943537	-1.623038
H	5.265809	-0.993671	0.000362
H	4.402418	0.188342	1.013584
H	2.236162	-2.693414	2.856002
H	-0.181585	-2.255981	3.299736
H	-1.447783	-0.744655	1.811981
Pd	-0.550710	1.541014	0.239301
C	-2.062197	-0.877981	-0.897570
C	-3.266727	-0.264365	-0.537594
C	-1.994239	-2.273607	-0.896490
C	-4.371241	-1.020483	-0.181348
H	-3.330060	0.820281	-0.542269
C	-3.101584	-3.028600	-0.549770
H	-1.067610	-2.774171	-1.150048
C	-4.291435	-2.406648	-0.188148
H	-5.296197	-0.527258	0.095564
H	-3.035394	-4.110903	-0.553019
H	-5.154877	-3.002780	0.085606
Cl	-1.080963	3.749462	0.806274
H	0.419466	-1.568203	-1.960336

T

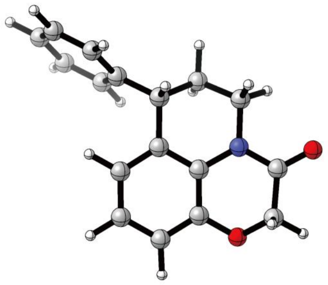

N	1.907694	-0.973789	0.069242
C	1.057820	-2.161288	0.099118
C	-0.362450	-1.810964	0.469221
C	-0.905566	-0.712618	-0.445963
C	3.252063	-1.164157	-0.010890
C	4.071216	0.092579	-0.178643
O	3.496444	1.233324	0.430317
C	2.186222	1.417426	0.108638
C	1.350244	0.309762	-0.062976
C	1.689266	2.703783	0.014379
C	0.341295	2.894644	-0.249827
C	-0.496139	1.802269	-0.412529
C	-0.011305	0.501305	-0.317456
O	3.768120	-2.267101	-0.001492
H	1.089069	-2.641136	-0.885116
H	1.491428	-2.861929	0.812983
H	-0.976794	-2.709660	0.385215
H	-0.830780	-1.079763	-1.477409
H	5.044164	-0.065059	0.282351
H	4.216234	0.263142	-1.255506
H	2.367718	3.536932	0.155900
H	-0.055782	3.900075	-0.329028
H	-1.551460	1.954647	-0.603745
C	-2.360498	-0.427386	-0.176705
C	-3.309141	-0.600466	-1.178929
C	-2.788406	-0.001629	1.080645
C	-4.656001	-0.358989	-0.935952
H	-2.988254	-0.926929	-2.163608
C	-4.130750	0.240089	1.327325
H	-2.061560	0.154044	1.871134
C	-5.070683	0.061572	0.318769
H	-5.380830	-0.498949	-1.730769
H	-4.446289	0.571656	2.310849
H	-6.120813	0.251453	0.512131
H	-0.409546	-1.477374	1.509952

U

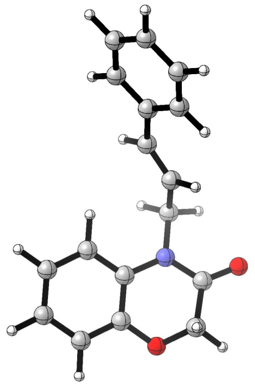

N	1.567718	-0.843168	0.540818	C	1.322081	1.591702	0.356069	C	-3.219699	-0.014253	0.309451
C	0.382788	-0.978359	1.380486	O	1.724912	-3.095042	0.263324	C	-3.605842	-0.826378	-0.762597
C	-0.894277	-0.896298	0.609639	H	0.462897	-1.959781	1.853610	C	-4.120961	0.953396	0.761659
C	-1.920326	-0.127454	0.972061	H	0.422967	-0.219202	2.164263	C	-4.842333	-0.664419	-1.362805
C	2.147550	-1.974467	0.047755	H	-0.951306	-1.534743	-0.268158	H	-2.937244	-1.597978	-1.127308
C	3.346783	-1.735539	-0.839211	H	-1.801516	0.503148	1.852821	C	-5.360697	1.115324	0.161176
O	4.113849	-0.608209	-0.458064	H	3.997034	-2.605551	-0.778450	H	-3.839197	1.587307	1.596891
C	3.365767	0.518344	-0.309023	H	2.994897	-1.628180	-1.875352	C	-5.726206	0.307545	-0.905791
C	2.059936	0.425437	0.185214	H	4.947505	1.780118	-0.962336	H	-5.123310	-1.304655	-2.192121
C	3.928525	1.749031	-0.594015	H	3.633588	3.871913	-0.624852	H	-6.043014	1.873892	0.529219
C	3.190435	2.908655	-0.400282	H	1.298895	3.725360	0.208215	H	-6.694915	0.429218	-1.377719
C	1.887580	2.826362	0.067292	H	0.301981	1.535551	0.711006				

CuBr

Cu	0.000000	0.000000	-1.237930	Br	0.000000	0.000000	1.025713

CuBrCl

Cu	0.000000	0.814794	0.000000	Cl	2.109012	1.091472	0.000000	Br	-1.024377	-1.205259	0.000000

SO_2

S	0.000000	0.000000	0.380758	O	0.000000	1.247633	-0.380758	O	0.000000	-1.247633	-0.380758

$PdCl_2H$

Pd	0.000000	0.000000	-0.113812	Cl	0.000000	-2.220493	0.113186
Cl	0.000000	2.220493	0.113186	H	0.000000	0.000000	1.387026

CO_3^{2-}

C	0.000000	0.000000	0.000140	O	0.000000	1.121785	-0.648447
O	0.000000	0.000000	1.296788	O	0.000000	-1.121785	-0.648447

HCO_3^-

C	0.000000	0.161152	0.000000	O	1.217320	0.426777	0.000000	H	0.575821	-1.643346	0.000000
O	-0.293551	-1.230566	0.000000	O	-0.995746	0.888343	0.000000				

PdCl

Pd	0.000000	0.000000	0.623082
Cl	0.000000	0.000000	-1.685986

References

[1] M. J. Frisch, G. W. Trucks, H.B. Schlegel, G. E. Scuseria, M. A. Robb, J. R. Cheeseman, G. Scalmani, V. Barone, B. Mennucci, G. A. Petersson, H. Nakatsuji, M. Caricato, X. Li, H. P. Hratchian, A. F. Izmaylov, J. Bloino, G. Zheng, J. L. Sonnenberg, M. Hada, M. Ehara, K. Toyota, R. Fukuda, J. Hasegawa, M. Ishida, T. Nakajima, Y. Honda, O. Kitao, H. Nakai, T. Vreven, J. A. Jr. Montgomery, J. E. Peralta, F. Ogliaro, M. Bearpark, J. J. Heyd, E. Brothers, K. N. Kudin, V. N. Staroverov, R. Kobayashi, J. Normand, K. Raghavachari, A. Rendell, J. C. Burant, S. S. Iyengar, J. Tomasi, M. Cossi, N. Rega, N. J. Millam, M. Klene, J. E. Knox, J. B. Cross, V. Bakken, C. Adamo, J. Jaramillo, R. Gomperts, R. E. Stratmann, O. Yazyev, A. J. Austin, R. Cammi, C. Pomelli, J. W. Ochterski, R. L. Martin, K. Morokuma, V. G. Zakrzewski, G. A. Voth, P. Salvador, J. J. Dannenberg, S. Dapprich, A. D. Daniels, Ö. Farkas, J. B. Foresman, J. V. Ortiz, J. Cioslowski, D. J. Fox, Gaussian, Inc., Gaussian 09, Revision E.01. *Wallingford CT,* **2013**.

[2] C. Adamo, V. Barone, *J. Chem. Phys.* **1999**, *110*, 6158-6170.

[3] S. Grimme, J. Antony, S. Ehrlich, H. Krieg, *J. Chem. Phys.* **2010**, *132*, 154104.

[4] A. Schäfer, C. Huber, R. Ahlrichs, R. *J. Chem. Phys.* **1994**, *100*, 5829-5839.

[5] A. V. Marenich, C. J. Cramer, D. G. Truhlar, *J. Phys. Chem. B* **2009**, 113, 6378-6396.

[6] J. Zheng, X. Xu, D. G. Truhlar, *Theor. Chem. Acc.* **2011**, 128, 295-305.
