# Supplementary material for: Palladium‐Catalyzed Dual Csp2─Csp3 Bond Formation: A Versatile Platform for the Synthesis of Benzo‐Fused Heterocycles
Source: Adv Sci (Weinh). 2025 Apr 28;12(25):2500897. doi: 10.1002/advs.202500897 (PMC12224930; doi:10.1002/advs.202500897)
Supplement: Supplementary file 3 — Supporting Information 3 [file ADVS-12-2500897-s002.pdf]

ADVANCED SCIENCE
Open Access

Supporting Information

for *Adv. Sci.*, DOI 10.1002/advs.202500897

Palladium-Catalyzed Dual Csp^2—Csp^3 Bond Formation: A Versatile Platform for the Synthesis of Benzo-Fused Heterocycles

Jiahui Huang, Yuantao You, Yijian Ma, Xingying He, Yixiao Li, Arunachalam Kesavan, Chengzhi Jin, Chengshuo Shen, Min Zhang* and Kedong Yuan**

(R)-7-(4-fluoro-3-(trifluoromethyl)phenyl)-6,7-dihydro-5H-[1,4]oxazino[2,3,4-i*J*] quinolin-3(2H)-one (34)

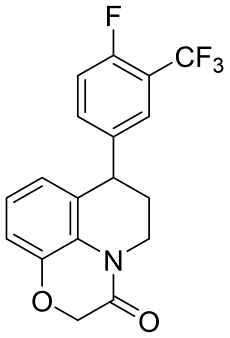

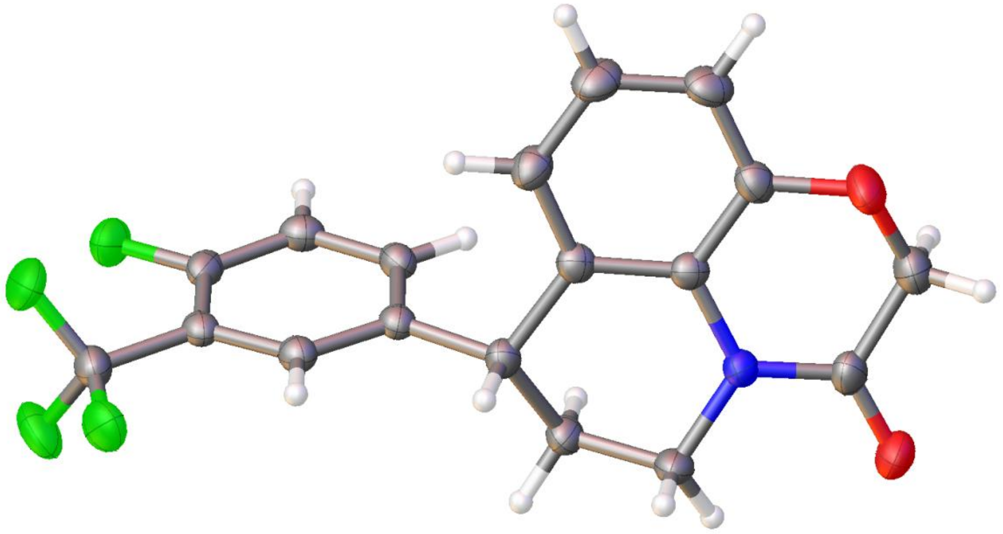

Table 1 Crystal data and structure refinement for YYT-176.

Identification code	YYT-176
Empirical formula	$C_{18}H_{13}F_4NO_2$
Formula weight	351.29
Temperature/K	150.00(10)
Crystal system	triclinic
Space group	P-1
a/Å	7.5840(8)
b/Å	9.1837(7)
c/Å	11.7093(8)
α/°	79.685(6)
β/°	77.854(7)
γ/°	68.533(9)
Volume/Å^3	737.30(12)
Z	2
ρ_{calc}g/cm^3	1.582

μ/mm^{-1}	0.137
F(000)	360.0
Crystal size/mm^3	0.16 × 0.14 × 0.12
Radiation	MoKα (λ = 0.71073)
2Θ range for data collection/°	4.796 to 59.104
Index ranges	-10 ≤ h ≤ 7, -12 ≤ k ≤ 9, -15 ≤ l ≤ 13
Reflections collected	5695
Independent reflections	3400 [R_{int} = 0.0277, R_{sigma} = 0.0489]
Data/restraints/parameters	3400/0/226
Goodness-of-fit on F^2	1.069
Final R indexes [I>=2σ (I)]	R_1 = 0.0481, wR_2 = 0.1136
Final R indexes [all data]	R_1 = 0.0675, wR_2 = 0.1252
Largest diff. peak/hole / e Å^{-3}	0.35/-0.31

Table 2 Fractional Atomic Coordinates (×10^4) and Equivalent Isotropic Displacement Parameters (Å^2×10^3) for YYT-176. U_{eq} is defined as 1/3 of of the trace of the orthogonalised U_{IJ} tensor.

Atom	*x*	*y*	*z*	U(eq)
F1	6574.3(17)	2153.0(11)	9470.5(9)	38.7(3)
F3	855.5(18)	2849.6(12)	9612.4(10)	43.3(3)
F2	3608.1(19)	1076.2(11)	9183.8(10)	46.2(3)
F4	2930(2)	2151.3(13)	10775.9(9)	48.9(4)
O2	511(2)	13646.1(13)	6547.3(11)	42.9(4)
O1	2402(2)	11686.7(14)	3942.6(11)	46.2(4)
N1	1250(2)	11066.8(15)	6348.3(11)	28.1(4)
C13	2342(3)	5093.9(17)	8456.6(13)	25.2(4)
C12	3060(3)	6275.7(17)	7904.0(13)	23.8(4)
C7	2127(3)	9813.8(18)	5638.7(13)	24.1(4)
C15	5417(3)	3509.1(18)	8952.7(13)	26.7(4)
C17	4988(3)	6026.4(18)	7880.2(13)	25.9(4)
C14	3519(3)	3704.7(17)	9000.6(13)	25.4(4)
C5	1726(3)	7841.8(17)	7404.0(14)	25.8(4)
C6	2345(3)	8253.9(18)	6097.2(14)	25.3(4)
C16	6193(3)	4642.1(19)	8393.3(14)	28.4(4)
C8	2727(3)	10162.9(19)	4435.7(14)	30.3(4)
C3	385(3)	10739.6(19)	7570.6(14)	31.1(4)
C2	1213(3)	12551.4(18)	5943.3(15)	30.7(4)
C4	1585(3)	9151.2(18)	8096.9(14)	30.0(4)

C18	2740(3)	2447.4(19)	9635.4(15)	33.0(5)
C11	3094(3)	7099(2)	5329.9(15)	32.9(4)
C9	3547(3)	8988(2)	3704.5(15)	36.2(5)
C1	2117(3)	12815(2)	4692.0(16)	37.8(5)
C10	3693(3)	7457(2)	4146.9(15)	39.0(5)

Table 3 Anisotropic Displacement Parameters (Å^2×10^3) for YYT-176. The Anisotropic displacement factor exponent takes the form: $-2\pi^2[h^2a^{*2}U_{11}+2hka^*b^*U_{12}+\ldots]$.

Atom	U_{11}	U_{22}	U_{33}	U_{23}	U_{13}	U_{12}
F1	41.4(8)	28.8(5)	35.0(6)	3.8(4)	-8.9(5)	-1.0(5)
F3	44.8(8)	38.3(6)	48.1(6)	0.3(5)	2.1(6)	-23.3(6)
F2	62.5(9)	22.6(5)	51.7(7)	-7.1(5)	-0.8(6)	-15.5(5)
F4	76.4(10)	49.6(7)	25.9(5)	8.4(5)	-5.7(6)	-34.6(7)
O2	57.4(11)	23.7(6)	39.8(7)	-2.8(5)	-2.3(7)	-8.1(6)
O1	73.0(12)	34.1(7)	25.6(6)	4.5(5)	-0.6(7)	-19.1(7)
N1	34.8(10)	22.6(6)	21.8(7)	0.0(5)	-1.2(6)	-6.7(6)
C13	28.1(11)	25.1(8)	22.8(8)	-4.5(6)	-2.1(7)	-9.5(7)
C12	28.7(11)	23.1(7)	18.8(7)	-3.4(6)	-2.0(7)	-8.3(7)
C7	22.3(10)	26.1(8)	22.5(8)	-2.8(6)	-4.9(7)	-5.9(7)
C15	34.1(12)	22.7(7)	18.4(7)	-2.3(6)	-4.9(7)	-3.5(7)
C17	31.4(11)	25.2(8)	21.5(8)	-2.9(6)	0.1(7)	-12.1(7)
C14	33.1(11)	23.3(7)	19.3(7)	-4.6(6)	-0.4(7)	-10.1(7)
C5	26.4(11)	22.9(7)	25.3(8)	0.9(6)	-3.8(7)	-7.0(7)
C6	24.5(10)	27.2(8)	23.5(8)	-1.3(6)	-7.1(7)	-7.2(7)
C16	27.3(11)	32.8(8)	24.2(8)	-6.9(7)	-1.7(7)	-8.8(8)
C8	32.4(12)	31.9(9)	24.6(8)	1.8(7)	-5.1(7)	-10.7(8)
C3	38.8(12)	26.8(8)	21.4(8)	-3.4(6)	1.5(8)	-7.0(8)
C2	30.4(11)	23.8(8)	32.7(9)	2.5(7)	-5.5(8)	-5.4(7)
C4	40.1(12)	24.9(8)	21.4(8)	-1.3(6)	-2.9(7)	-8.3(8)
C18	43.7(13)	26.1(8)	27.3(8)	-0.5(7)	-1.0(8)	-13.1(8)
C11	41.1(13)	26.9(8)	29.3(9)	-4.6(7)	-9.4(8)	-7.4(8)
C9	40.1(13)	44.1(10)	20.2(8)	-3.8(7)	-4.1(8)	-9.6(9)
C1	46.0(14)	31.0(9)	33.4(9)	3.7(7)	-3.1(9)	-14.6(9)
C10	48.9(14)	37.5(10)	26.9(9)	-11.5(7)	-9.3(9)	-4.9(9)

Table 4 Bond Lengths for YYT-176.

Atom	Atom	Length/Å	Atom	Atom	Length/Å

F1	C15	1.3573(18)	C7	C6	1.399(2)
F3	C18	1.344(2)	C7	C8	1.403(2)
F2	C18	1.3358(19)	C15	C14	1.374(3)
F4	C18	1.342(2)	C15	C16	1.378(2)
O2	C2	1.224(2)	C17	C16	1.386(2)
O1	C8	1.368(2)	C14	C18	1.497(2)
O1	C1	1.405(2)	C5	C6	1.525(2)
N1	C7	1.417(2)	C5	C4	1.528(2)
N1	C3	1.471(2)	C6	C11	1.393(2)
N1	C2	1.353(2)	C8	C9	1.378(2)
C13	C12	1.388(2)	C3	C4	1.510(2)
C13	C14	1.395(2)	C2	C1	1.499(2)
C12	C17	1.390(3)	C11	C10	1.384(2)
C12	C5	1.522(2)	C9	C10	1.381(3)

Table 5 Bond Angles for YYT-176.

Atom	Atom	Atom	Angle/°	Atom	Atom	Atom	Angle/°
C8	O1	C1	117.49(13)	C11	C6	C7	118.54(14)
C7	N1	C3	119.04(12)	C11	C6	C5	121.01(13)
C2	N1	C7	122.03(13)	C15	C16	C17	117.93(17)
C2	N1	C3	118.91(13)	O1	C8	C7	120.90(15)
C12	C13	C14	120.68(17)	O1	C8	C9	118.08(14)
C13	C12	C17	118.48(15)	C9	C8	C7	120.89(15)
C13	C12	C5	120.25(16)	N1	C3	C4	109.88(14)
C17	C12	C5	121.17(13)	O2	C2	N1	123.34(15)
C6	C7	N1	121.76(14)	O2	C2	C1	120.07(14)
C6	C7	C8	119.44(15)	N1	C2	C1	116.58(15)
C8	C7	N1	118.76(14)	C3	C4	C5	111.12(14)
F1	C15	C14	119.05(14)	F3	C18	C14	112.09(15)
F1	C15	C16	118.66(17)	F2	C18	F3	106.78(15)
C14	C15	C16	122.29(15)	F2	C18	F4	106.44(14)
C16	C17	C12	121.82(14)	F2	C18	C14	112.83(15)
C13	C14	C18	121.03(17)	F4	C18	F3	105.90(15)
C15	C14	C13	118.78(14)	F4	C18	C14	112.32(15)
C15	C14	C18	120.19(15)	C10	C11	C6	121.40(15)
C12	C5	C6	112.98(13)	C8	C9	C10	119.67(16)
C12	C5	C4	109.70(14)	O1	C1	C2	117.81(14)
C6	C5	C4	110.20(12)	C9	C10	C11	119.94(16)
C7	C6	C5	120.44(14)				

Table 6 Torsion Angles for YYT-176.

A	B	C	D	Angle/°	A	B	C	D	Angle/°
F1	C15	C14	C13	-179.35(13)	C15	C14	C18	F4	59.7(2)
F1	C15	C14	C18	1.1(2)	C17	C12	C5	C6	60.5(2)
F1	C15	C16	C17	-179.32(13)	C17	C12	C5	C4	-62.86(19)
O2	C2	C1	O1	-162.17(18)	C14	C13	C12	C17	1.6(2)
O1	C8	C9	C10	-172.56(19)	C14	C13	C12	C5	-174.79(14)
N1	C7	C6	C5	-3.0(3)	C14	C15	C16	C17	0.9(2)
N1	C7	C6	C11	175.41(17)	C5	C12	C17	C16	176.12(14)
N1	C7	C8	O1	-2.7(3)	C5	C6	C11	C10	-178.77(19)
N1	C7	C8	C9	-178.51(17)	C6	C7	C8	O1	175.02(17)
N1	C3	C4	C5	-59.9(2)	C6	C7	C8	C9	-0.8(3)
N1	C2	C1	O1	18.7(3)	C6	C5	C4	C3	51.0(2)
C13	C12	C17	C16	-0.2(2)	C6	C11	C10	C9	-0.3(3)
C13	C12	C5	C6	-123.21(16)	C16	C15	C14	C13	0.5(2)
C13	C12	C5	C4	113.41(17)	C16	C15	C14	C18	-179.06(15)
C13	C14	C18	F3	-0.7(2)	C8	O1	C1	C2	-30.4(3)
C13	C14	C18	F2	119.85(18)	C8	C7	C6	C5	179.28(17)
C13	C14	C18	F4	-119.84(18)	C8	C7	C6	C11	-2.3(3)
C12	C13	C14	C15	-1.7(2)	C8	C9	C10	C11	-2.8(3)
C12	C13	C14	C18	177.82(14)	C3	N1	C7	C6	-5.9(3)
C12	C17	C16	C15	-1.0(2)	C3	N1	C7	C8	171.83(17)
C12	C5	C6	C7	-143.00(17)	C3	N1	C2	O2	0.9(3)
C12	C5	C6	C11	38.6(2)	C3	N1	C2	C1	179.95(17)
C12	C5	C4	C3	175.96(14)	C2	N1	C7	C6	172.96(18)
C7	N1	C3	C4	37.0(2)	C2	N1	C7	C8	-9.3(3)
C7	N1	C2	O2	-177.98(18)	C2	N1	C3	C4	-141.83(17)
C7	N1	C2	C1	1.1(3)	C4	C5	C6	C7	-19.9(2)
C7	C6	C11	C10	2.8(3)	C4	C5	C6	C11	161.70(17)
C7	C8	C9	C10	3.3(3)	C1	O1	C8	C7	22.6(3)
C15	C14	C18	F3	178.78(15)	C1	O1	C8	C9	-161.49(18)
C15	C14	C18	F2	-60.6(2)					

Table 7 Hydrogen Atom Coordinates (Å×10^4) and Isotropic Displacement Parameters (Å^2×10^3) for YYT-176.

Atom	*x*	*y*	*z*	U(eq)
H13	1035	5233	8464	30

H17	5493	6827	7502	31
H5	419	7764	7508	31
H16	7513	4479	8361	34
H3A	-932	10751	7591	37
H3B	303	11567	8038	37
H4A	1005	8946	8923	36
H4B	2889	9155	8096	36
H11	3195	6043	5625	39
H9	4009	9230	2900	43
H1A	1310	13846	4343	45
H1B	3380	12899	4698	45
H10	4204	6650	3640	47

Experimental

Single crystals of $C_{18}H_{13}F_4NO_2$ [YYT-176] were []. A suitable crystal was selected and [] on a SuperNova, Dual, Cu at zero, AtlasS2 diffractometer. The crystal was kept at 150.00(10) K during data collection. Using Olex2 [1], the structure was solved with the ShelXT [2] structure solution program using Intrinsic Phasing and refined with the ShelXL [3] refinement package using Least Squares minimisation.

1. Dolomanov, O.V., Bourhis, L.J., Gildea, R.J, Howard, J.A.K. & Puschmann, H. (2009), J. Appl. Cryst. 42, 339-341.
2. Sheldrick, G.M. (2008). Acta Cryst. A64, 112-122.
3. Sheldrick, G.M. (2008). Acta Cryst. A64, 112-122.

Crystal structure determination of [YYT-176]

Crystal Data for $C_{18}H_{13}F_4NO_2$ (M =351.29 g/mol): triclinic, space group P-1 (no. 2), a = 7.5840(8) Å, b = 9.1837(7) Å, c = 11.7093(8) Å, α = 79.685(6)°, β = 77.854(7)°, γ = 68.533(9)°, V = 737.30(12) Å^3, Z = 2, T = 150.00(10) K, μ(MoKα) = 0.137 mm^{-1}, $Dcalc$ = 1.582 g/cm^3, 5695 reflections measured (4.796° ≤ 2Θ ≤ 59.104°), 3400 unique (R_{int} = 0.0277, R_{sigma} = 0.0489) which were used in all calculations. The final R_1 was 0.0481 (I > 2σ(I)) and wR_2 was 0.1252 (all data).

Refinement model description

Number of restraints - 0, number of constraints - unknown.

Details:

1. Fixed Uiso
 At 1.2 times of:
  All C(H) groups, All C(H,H) groups
2.a Ternary CH refined with riding coordinates:
 C5(H5)
2.b Secondary CH2 refined with riding coordinates:
 C3(H3A,H3B), C4(H4A,H4B), C1(H1A,H1B)

2.c Aromatic/amide H refined with riding coordinates:
C13(H13), C17(H17), C16(H16), C11(H11), C9(H9), C10(H10)

This report has been created with Olex2, compiled on 2014.11.28 svn.r3106 for OlexSys. Please let us know if there are any errors or if you would like to have additional features.

9-chloro-1-(p-tolyl)-2,3,6,7-tetrahydro-1H,5H-pyrido[3,2,1-iJ]quinolin-5-one (54)

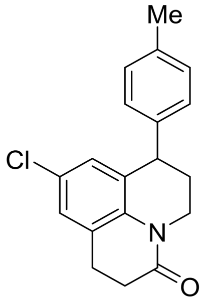

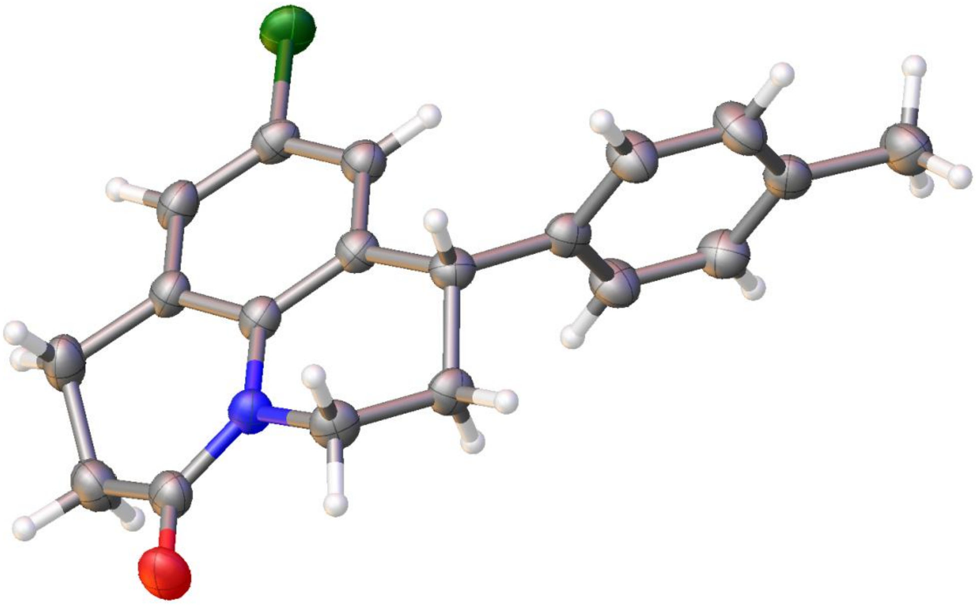

Table 1 Crystal data and structure refinement for yyt-372.

Identification code	yyt-372
Empirical formula	$C_{19}H_{18}ClNO$
Formula weight	311.79
Temperature/K	169.99(10)
Crystal system	monoclinic
Space group	$P2_1/c$
a/Å	5.4508(2)
b/Å	26.4137(10)
c/Å	10.8299(4)
α/°	90

β/°	98.247(3)
γ/°	90
Volume/Å^3	1543.13(10)
Z	4
ρ_{calc}g/cm^3	1.342
μ/mm^{-1}	2.187
F(000)	656.0
Crystal size/mm^3	0.15 × 0.13 × 0.11
Radiation	CuKα (λ = 1.54184)
2Θ range for data collection/°	6.692 to 147.772
Index ranges	$-6 \leq h \leq 6$, $-30 \leq k \leq 32$, $-13 \leq l \leq 13$
Reflections collected	5851
Independent reflections	3040 [R_{int} = 0.0456, R_{sigma} = 0.0586]
Data/restraints/parameters	3040/0/200
Goodness-of-fit on F^2	1.043
Final R indexes [I>=2σ (I)]	R_1 = 0.0602, wR_2 = 0.1613
Final R indexes [all data]	R_1 = 0.0703, wR_2 = 0.1744
Largest diff. peak/hole / e Å^{-3}	0.36/-0.42

Table 2 Fractional Atomic Coordinates (×10^4) and Equivalent Isotropic Displacement Parameters (Å^2×10^3) for yyt-372. U_{eq} is defined as 1/3 of of the trace of the orthogonalised U_{IJ} tensor.

Atom	*x*	*y*	*z*	U(eq)
Cl1	710.9(12)	6631.2(2)	810.6(6)	42.2(2)
O1	10552(4)	6869.4(8)	6960.8(18)	45.7(5)
N1	8852(4)	6575.2(7)	5067.3(19)	29.8(4)
C17	6931(4)	6587.4(8)	4029(2)	27.4(5)
C9	6660(4)	6189.2(8)	3162(2)	28.0(5)
C8	8483(4)	5749.9(9)	3245(2)	29.4(5)
C5	7370(4)	5256.1(9)	2717(2)	30.4(5)
C2	5498(4)	4312.6(10)	1789(2)	33.1(5)
C13	5347(4)	7007.8(8)	3868(2)	30.2(5)
C16	8871(5)	6888.3(9)	6083(2)	33.9(5)
C12	3418(5)	7021.1(9)	2880(2)	33.2(5)
C11	3136(4)	6621.2(9)	2051(2)	31.2(5)
C10	4719(4)	6212.4(9)	2173(2)	30.8(5)
C19	9703(4)	5690.1(9)	4596(2)	31.1(5)
C6	8378(5)	5006.7(10)	1778(3)	38.5(6)
C18	10784(4)	6186.9(10)	5112(2)	34.1(5)

C3	4476(5)	4563.1(10)	2712(2)	36.9(6)
C4	5405(5)	5029.1(10)	3174(2)	36.9(6)
C15	6701(5)	7238.3(10)	6068(3)	40.6(6)
C14	5804(5)	7438.7(10)	4764(3)	40.4(6)
C1	4531(6)	3801.5(11)	1328(3)	45.0(7)
C7	7454(5)	4543.9(11)	1328(3)	43.1(6)

Table 3 Anisotropic Displacement Parameters (Å^2×10^3) for yyt-372. The Anisotropic displacement factor exponent takes the form: $-2\pi^2[h^2a^{*2}U_{11}+2hka^*b^*U_{12}+\ldots]$.

Atom	U_{11}	U_{22}	U_{33}	U_{23}	U_{13}	U_{12}
Cl1	35.3(4)	46.4(4)	40.0(4)	4.9(3)	-11.5(3)	4.0(2)
O1	46.5(11)	44.6(11)	40.2(10)	-7.5(8)	-13.4(8)	2.2(8)
N1	26.2(10)	29.7(10)	31.1(10)	0.6(8)	-3.7(8)	0.5(8)
C17	22.2(11)	27.2(11)	32.0(12)	3.2(9)	1.2(9)	-1.1(8)
C9	24.5(11)	28.9(11)	30.2(11)	2.2(9)	2.6(9)	-0.5(9)
C8	25.8(11)	33.1(11)	29.6(11)	0.5(9)	5.4(9)	1.3(9)
C5	26.4(11)	33.2(12)	31.0(11)	1.9(9)	1.4(9)	5.4(9)
C2	30.4(12)	36.2(12)	30.5(12)	-0.2(9)	-3.9(9)	-0.2(10)
C13	28.3(11)	25.6(10)	35.6(12)	4.4(9)	0.6(9)	-1.2(9)
C16	34.3(12)	30.1(11)	35.9(12)	-1(1)	0.3(10)	-3(1)
C12	31.2(12)	26.6(11)	40.4(13)	6.8(10)	1(1)	2.4(9)
C11	24.7(11)	34.5(12)	32.8(12)	7.7(9)	-2.0(9)	-1.5(9)
C10	28.9(11)	31.2(11)	31.6(12)	1.2(9)	1.9(9)	0.3(9)
C19	24.4(10)	32.4(11)	35.2(12)	3.0(9)	0.1(9)	5.9(9)
C6	33.2(13)	43.5(13)	41.7(14)	-6.4(12)	14.7(11)	-5.3(11)
C18	22.9(11)	39.4(13)	38.1(13)	0.4(10)	-2.7(9)	3.5(10)
C3	31.0(12)	43.3(14)	37.3(13)	0.4(11)	7.4(10)	-4.7(10)
C4	34.9(13)	41.7(13)	36.6(13)	-4.9(11)	13.1(11)	0.5(11)
C15	41.0(14)	37.8(13)	41.5(14)	-7.5(11)	0.3(11)	1.3(11)
C14	40.1(14)	28.6(11)	49.5(15)	-3.7(11)	-4.0(11)	5.1(10)
C1	48.3(16)	44.2(14)	40.6(14)	-4.7(11)	-0.4(12)	-9.9(12)
C7	42.7(14)	48.0(15)	41.3(15)	-13.8(12)	14.7(12)	-6.9(12)

Table 4 Bond Lengths for yyt-372.

Atom	Atom	Length/Å	Atom	Atom	Length/Å
Cl1	C11	1.745(2)	C2	C3	1.381(4)
O1	C16	1.223(3)	C2	C1	1.508(4)

N1	C17	1.423(3)	C2	C7	1.382(4)
N1	C16	1.375(3)	C13	C12	1.388(3)
N1	C18	1.466(3)	C13	C14	1.494(3)
C17	C9	1.404(3)	C16	C15	1.500(4)
C17	C13	1.402(3)	C12	C11	1.381(3)
C9	C8	1.522(3)	C11	C10	1.377(3)
C9	C10	1.394(3)	C19	C18	1.512(3)
C8	C5	1.516(3)	C6	C7	1.384(4)
C8	C19	1.526(3)	C3	C4	1.397(4)
C5	C6	1.389(3)	C15	C14	1.521(4)
C5	C4	1.380(3)			

Table 5 Bond Angles for yyt-372.

Atom	Atom	Atom	Angle/°	Atom	Atom	Atom	Angle/°
C17	N1	C18	119.06(19)	C12	C13	C17	120.1(2)
C16	N1	C17	122.6(2)	C12	C13	C14	121.3(2)
C16	N1	C18	118.1(2)	O1	C16	N1	121.1(2)
C9	C17	N1	120.5(2)	O1	C16	C15	122.4(2)
C13	C17	N1	119.1(2)	N1	C16	C15	116.5(2)
C13	C17	C9	120.4(2)	C11	C12	C13	118.9(2)
C17	C9	C8	121.8(2)	C12	C11	Cl1	119.37(18)
C10	C9	C17	118.5(2)	C10	C11	Cl1	118.75(19)
C10	C9	C8	119.7(2)	C10	C11	C12	121.9(2)
C9	C8	C19	109.08(19)	C11	C10	C9	120.3(2)
C5	C8	C9	114.29(19)	C18	C19	C8	111.0(2)
C5	C8	C19	111.63(19)	C7	C6	C5	121.0(2)
C6	C5	C8	120.4(2)	N1	C18	C19	110.80(19)
C4	C5	C8	121.8(2)	C2	C3	C4	121.2(2)
C4	C5	C6	117.8(2)	C5	C4	C3	121.0(2)
C3	C2	C1	120.7(2)	C16	C15	C14	111.9(2)
C3	C2	C7	117.5(2)	C13	C14	C15	109.9(2)
C7	C2	C1	121.8(2)	C2	C7	C6	121.6(2)
C17	C13	C14	118.7(2)				

Table 6 Torsion Angles for yyt-372.

A	B	C	D	Angle/°	A	B	C	D	Angle/°
Cl1	C11	C10	C9	-179.24(17)	C2	C3	C4	C5	0.4(4)

O1	C16	C15	C14	144.9(3)	C13	C17	C9	C8	174.6(2)
N1	C17	C9	C8	-4.0(3)	C13	C17	C9	C10	-2.7(3)
N1	C17	C9	C10	178.7(2)	C13	C12	C11	Cl1	179.65(18)
N1	C17	C13	C12	-178.2(2)	C13	C12	C11	C10	-0.4(4)
N1	C17	C13	C14	2.6(3)	C16	N1	C17	C9	-164.8(2)
N1	C16	C15	C14	-36.9(3)	C16	N1	C17	C13	16.6(3)
C17	N1	C16	O1	-179.9(2)	C16	N1	C18	C19	137.5(2)
C17	N1	C16	C15	1.9(3)	C16	C15	C14	C13	52.5(3)
C17	N1	C18	C19	-37.4(3)	C12	C13	C14	C15	144.5(2)
C17	C9	C8	C5	151.2(2)	C12	C11	C10	C9	0.8(4)
C17	C9	C8	C19	25.4(3)	C10	C9	C8	C5	-31.6(3)
C17	C9	C10	C11	0.8(3)	C10	C9	C8	C19	-157.3(2)
C17	C13	C12	C11	-1.6(4)	C19	C8	C5	C6	-112.1(3)
C17	C13	C14	C15	-36.4(3)	C19	C8	C5	C4	65.3(3)
C9	C17	C13	C12	3.2(4)	C6	C5	C4	C3	0.3(4)
C9	C17	C13	C14	-176.0(2)	C18	N1	C17	C9	9.9(3)
C9	C8	C5	C6	123.5(2)	C18	N1	C17	C13	-168.6(2)
C9	C8	C5	C4	-59.0(3)	C18	N1	C16	O1	5.3(3)
C9	C8	C19	C18	-52.3(2)	C18	N1	C16	C15	-172.9(2)
C8	C9	C10	C11	-176.6(2)	C3	C2	C7	C6	0.6(4)
C8	C5	C6	C7	177.0(2)	C4	C5	C6	C7	-0.6(4)
C8	C5	C4	C3	-177.2(2)	C14	C13	C12	C11	177.5(2)
C8	C19	C18	N1	59.5(3)	C1	C2	C3	C4	178.0(2)
C5	C8	C19	C18	-179.50(19)	C1	C2	C7	C6	-178.2(3)
C5	C6	C7	C2	0.1(4)	C7	C2	C3	C4	-0.9(4)

Table 7 Hydrogen Atom Coordinates (Å×10[4]) and Isotropic Displacement Parameters (Å[2]×10[3]) for yyt-372.

Atom	*x*	*y*	*z*	U(eq)
H8	9817	5845	2744	35
H12	2312	7301	2777	40
H10	4489	5945	1581	37
H19A	8458	5570	5110	37
H19B	11035	5433	4639	37
H6	9721	5156	1440	46
H18A	11572	6138	5986	41
H18B	12078	6301	4619	41
H3	3115	4416	3038	44
H4	4674	5192	3813	44

H15A	7181	7527	6635	49
H15B	5328	7056	6381	49
H14A	4254	7634	4769	49
H14B	7067	7668	4500	49
H1A	5804	3544	1567	68
H1B	4117	3809	417	68
H1C	3044	3719	1699	68
H7	8181	4381	687	52

Experimental

Single crystals of $C_{19}H_{18}ClNO$ [yyt-372] were []. A suitable crystal was selected and [] on a SuperNova, Dual, Cu at zero, AtlasS2 diffractometer. The crystal was kept at 169.99(10) K during data collection. Using Olex2 [1], the structure was solved with the ShelXT [2] structure solution program using Direct Methods and refined with the ShelXL [3] refinement package using Least Squares minimisation.

1. Dolomanov, O.V., Bourhis, L.J., Gildea, R.J, Howard, J.A.K. & Puschmann, H. (2009), J. Appl. Cryst. 42, 339-341.
2. Sheldrick, G.M. (2008). Acta Cryst. A64, 112-122.
3. Sheldrick, G.M. (2008). Acta Cryst. A64, 112-122.

Crystal structure determination of [yyt-372]

Crystal Data for $C_{19}H_{18}ClNO$ (M =311.79 g/mol): monoclinic, space group P2$_1$/c (no. 14), a = 5.4508(2) Å, b = 26.4137(10) Å, c = 10.8299(4) Å, β = 98.247(3)°, V = 1543.13(10) Å^3, Z = 4, T = 169.99(10) K, μ(CuKα) = 2.187 mm^{-1}, $Dcalc$ = 1.342 g/cm^3, 5851 reflections measured (6.692° ≤ 2Θ ≤ 147.772°), 3040 unique (R_{int} = 0.0456, R_{sigma} = 0.0586) which were used in all calculations. The final R_1 was 0.0602 (I > 2σ(I)) and wR_2 was 0.1744 (all data).

Refinement model description

Number of restraints - 0, number of constraints - unknown.

Details:

1. Fixed Uiso

At 1.2 times of:

All C(H) groups, All C(H,H) groups

At 1.5 times of:

All C(H,H,H) groups

2.a Ternary CH refined with riding coordinates:

C8(H8)

2.b Secondary CH2 refined with riding coordinates:

C19(H19A,H19B), C18(H18A,H18B), C15(H15A,H15B), C14(H14A,H14B)

2.c Aromatic/amide H refined with riding coordinates:

C12(H12), C10(H10), C6(H6), C3(H3), C4(H4), C7(H7)

2.d Idealised Me refined as rotating group:
C1(H1A,H1B,H1C)

This report has been created with Olex2, compiled on 2014.11.28 svn.r3106 for OlexSys. Please let us know if there are any errors or if you would like to have additional features.

8-(p-tolyl)-7,8-dihydro-6H-[1,3]dioxolo[4,5-g]chromene (84)

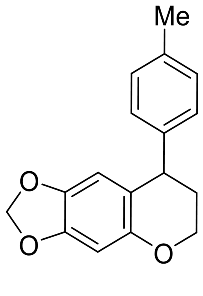

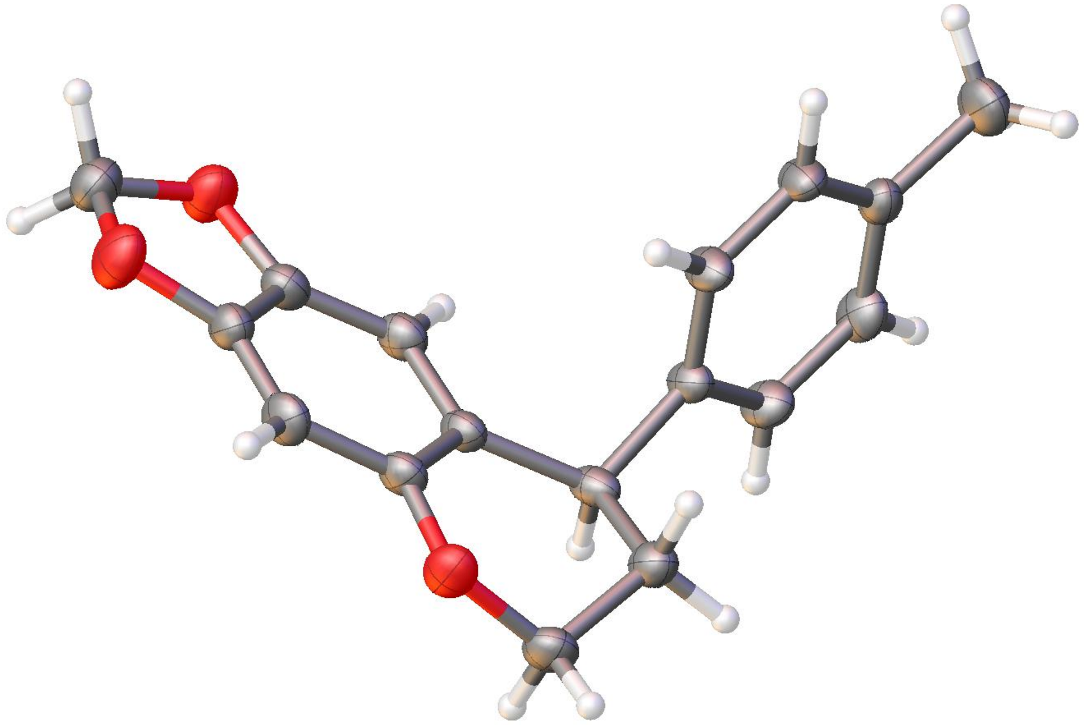

Table 1 Crystal data and structure refinement for YYT-384.

Identification code	YYT-384
Empirical formula	$C_{17}H_{16}O_3$
Formula weight	268.30
Temperature/K	185(20)
Crystal system	monoclinic
Space group	$P2_1/c$
a/Å	6.4369(3)
b/Å	10.7530(5)
c/Å	19.1181(10)

α/°	90
β/°	94.173(5)
γ/°	90
Volume/Å^3	1319.77(12)
Z	4
ρ_{calc}g/cm^3	1.350
μ/mm^{-1}	0.743
F(000)	568.0
Crystal size/mm^3	0.15 × 0.13 × 0.12
Radiation	CuKα (λ = 1.54184)
2Θ range for data collection/°	9.276 to 147.074
Index ranges	-6 ≤ h ≤ 7, -13 ≤ k ≤ 9, -23 ≤ l ≤ 22
Reflections collected	4514
Independent reflections	2595 [R_{int} = 0.0539, R_{sigma} = 0.0545]
Data/restraints/parameters	2595/0/182
Goodness-of-fit on F^2	1.063
Final R indexes [I>=2σ (I)]	R_1 = 0.0719, wR_2 = 0.1956
Final R indexes [all data]	R_1 = 0.0841, wR_2 = 0.2180
Largest diff. peak/hole / e Å^{-3}	0.28/-0.41

Table 2 Fractional Atomic Coordinates (×10^4) and Equivalent Isotropic Displacement Parameters (Å^2×10^3) for YYT-384. U_{eq} is defined as 1/3 of of the trace of the orthogonalised U_{IJ} tensor.

**Atom**	***x***	***y***	***z***	**U(eq)**
O1	10283(3)	4768.1(18)	7103.4(10)	36.0(5)
O3	5764(3)	1209.6(19)	5632.5(11)	42.3(6)
O2	9108(3)	1640(2)	5328.4(11)	45.7(6)
C17	7069(4)	3557(2)	7016.8(13)	25.7(5)
C15	6647(4)	2106(2)	6069.3(13)	29.9(6)
C16	5855(4)	2673(2)	6637.6(14)	29.1(6)
C5	5282(4)	3265(2)	8150.2(13)	25.7(5)
C11	9031(4)	3863(2)	6798.2(13)	28.0(5)
C8	6260(4)	4182(2)	7660.0(13)	27.2(6)
C12	9863(4)	3248(3)	6231.6(14)	32.8(6)
C13	8643(4)	2384(2)	5885.9(13)	30.5(6)
C6	6236(4)	2154(2)	8345.5(14)	30.8(6)
C9	8029(4)	4889(2)	8064.1(14)	31.2(6)
C4	3448(4)	3587(2)	8445.4(14)	30.9(6)

C2	3553(4)	1693(2)	9128.4(13)	30.9(6)
C7	5377(5)	1371(2)	8827.0(14)	34.0(6)
C3	2600(4)	2803(3)	8933.1(15)	33.8(6)
C10	9273(4)	5608(3)	7558.2(15)	35.3(6)
C14	7204(5)	1012(3)	5107.1(15)	39.5(7)
C1	2633(5)	866(3)	9663.7(17)	47.1(8)

Table 3 Anisotropic Displacement Parameters (Å^2×10^3) for YYT-384. The Anisotropic displacement factor exponent takes the form: $-2\pi^2[h^2a^{*2}U_{11}+2hka^*b^*U_{12}+\ldots]$.

Atom	U_{11}	U_{22}	U_{33}	U_{23}	U_{13}	U_{12}
O1	34.7(10)	35.4(10)	39.5(11)	-4.3(8)	14.3(8)	-13.1(8)
O3	48.1(12)	40.5(11)	39.9(11)	-9.8(9)	13.7(9)	-16.0(9)
O2	48.0(12)	49.5(13)	42.2(12)	-13.2(10)	21.4(9)	-9.1(10)
C17	28.1(12)	21.5(11)	28.7(12)	3.7(9)	10.6(9)	-1.4(9)
C15	34.0(13)	26.6(12)	29.7(13)	3.2(10)	6.4(10)	-5.2(10)
C16	27.4(12)	28.6(13)	32.3(13)	3.7(10)	9.7(9)	-3.6(10)
C5	27.8(12)	20.9(11)	30.0(12)	0.3(9)	12.3(9)	0.7(9)
C11	29.3(12)	24.9(12)	30.9(12)	3.3(10)	9.2(9)	-5.9(10)
C8	27.6(12)	20.3(11)	35.2(13)	5(1)	12.7(10)	2.4(9)
C12	31.7(13)	35.4(14)	33.3(13)	1.7(11)	16.2(10)	-5.1(11)
C13	37.6(14)	27.1(13)	28.3(13)	-0.3(10)	12.2(10)	-2.8(10)
C6	35.7(13)	24.2(12)	34.9(14)	1.9(10)	18.2(11)	7.1(10)
C9	36.6(13)	25.3(12)	33.1(13)	-1.6(10)	11.9(10)	-1.8(10)
C4	28.6(12)	26.6(12)	39.1(14)	-2.2(11)	13.4(10)	4.3(10)
C2	40.4(14)	26.7(12)	26.9(12)	-3.9(10)	12.5(10)	-7.1(10)
C7	48.4(16)	21.8(12)	33.7(14)	0.1(10)	17.3(12)	6.5(11)
C3	29.9(13)	36.8(14)	36.9(14)	-5.1(11)	16.9(10)	-1.8(11)
C10	40.9(14)	26.2(13)	40.0(15)	0.2(11)	10.9(12)	-9.8(11)
C14	49.2(16)	34.9(14)	35.2(14)	-7.7(12)	9.0(12)	-5.8(12)
C1	60.9(19)	43.1(17)	40.3(16)	3.0(13)	25.1(14)	-10.5(15)

Table 4 Bond Lengths for YYT-384.

Atom	Atom	Length/Å	Atom	Atom	Length/Å
O1	C11	1.367(3)	C5	C6	1.382(3)
O1	C10	1.441(3)	C5	C4	1.389(3)
O3	C15	1.371(3)	C11	C12	1.408(4)
O3	C14	1.431(3)	C8	C9	1.531(4)

O2	C13	1.382(3)	C12	C13	1.357(4)
O2	C14	1.435(4)	C6	C7	1.391(3)
C17	C16	1.399(4)	C9	C10	1.512(3)
C17	C11	1.398(3)	C4	C3	1.397(4)
C17	C8	1.526(3)	C2	C7	1.389(4)
C15	C16	1.376(4)	C2	C3	1.381(4)
C15	C13	1.389(4)	C2	C1	1.509(4)
C5	C8	1.528(3)			

Table 5 Bond Angles for YYT-384.

Atom	Atom	Atom	Angle/°	Atom	Atom	Atom	Angle/°
C11	O1	C10	115.18(19)	C17	C8	C9	109.90(19)
C15	O3	C14	105.7(2)	C5	C8	C9	109.6(2)
C13	O2	C14	105.8(2)	C13	C12	C11	117.2(2)
C16	C17	C8	120.4(2)	O2	C13	C15	109.0(2)
C11	C17	C16	119.2(2)	C12	C13	O2	128.4(2)
C11	C17	C8	120.4(2)	C12	C13	C15	122.5(2)
O3	C15	C16	129.1(2)	C5	C6	C7	120.8(2)
O3	C15	C13	110.4(2)	C10	C9	C8	109.9(2)
C16	C15	C13	120.4(2)	C5	C4	C3	120.4(2)
C15	C16	C17	119.0(2)	C7	C2	C1	121.3(3)
C6	C5	C8	122.0(2)	C3	C2	C7	118.4(2)
C6	C5	C4	118.7(2)	C3	C2	C1	120.3(2)
C4	C5	C8	119.2(2)	C2	C7	C6	120.8(2)
O1	C11	C17	123.9(2)	C2	C3	C4	121.0(2)
O1	C11	C12	114.7(2)	O1	C10	C9	110.4(2)
C17	C11	C12	121.5(2)	O3	C14	O2	107.8(2)
C17	C8	C5	112.93(19)				

Table 6 Torsion Angles for YYT-384.

A	B	C	D	Angle/°	A	B	C	D	Angle/°
O1	C11	C12	C13	-176.8(2)	C8	C17	C11	O1	-4.1(4)
O3	C15	C16	C17	178.9(3)	C8	C17	C11	C12	176.3(2)
O3	C15	C13	O2	-1.3(3)	C8	C5	C6	C7	175.9(3)
O3	C15	C13	C12	179.6(3)	C8	C5	C4	C3	-175.5(2)
C17	C11	C12	C13	2.9(4)	C8	C9	C10	O1	-64.3(3)
C17	C8	C9	C10	44.2(3)	C13	O2	C14	O3	-11.7(3)

C15	O3	C14	O2	10.9(3)	C13	C15	C16	C17	1.6(4)
C16	C17	C11	O1	175.7(2)	C6	C5	C8	C17	46.2(3)
C16	C17	C11	C12	-3.9(4)	C6	C5	C8	C9	-76.7(3)
C16	C17	C8	C5	45.4(3)	C6	C5	C4	C3	0.4(4)
C16	C17	C8	C9	168.1(2)	C4	C5	C8	C17	-138.0(2)
C16	C15	C13	O2	176.4(2)	C4	C5	C8	C9	99.1(3)
C16	C15	C13	C12	-2.7(4)	C4	C5	C6	C7	0.1(4)
C5	C8	C9	C10	168.9(2)	C7	C2	C3	C4	-0.1(4)
C5	C6	C7	C2	-0.7(4)	C3	C2	C7	C6	0.6(4)
C5	C4	C3	C2	-0.4(4)	C10	O1	C11	C17	-14.6(4)
C11	O1	C10	C9	48.5(3)	C10	O1	C11	C12	165.1(2)
C11	C17	C16	C15	1.6(4)	C14	O3	C15	C16	176.5(3)
C11	C17	C8	C5	-134.8(2)	C14	O3	C15	C13	-6.0(3)
C11	C17	C8	C9	-12.1(3)	C14	O2	C13	C15	8.1(3)
C11	C12	C13	O2	-178.5(3)	C14	O2	C13	C12	-172.9(3)
C11	C12	C13	C15	0.4(4)	C1	C2	C7	C6	-178.6(3)
C8	C17	C16	C15	-178.6(2)	C1	C2	C3	C4	179.1(3)

Table 7 Hydrogen Atom Coordinates (Å×10^{4}) and Isotropic Displacement Parameters (Å^{2}×10^{3}) for YYT-384.

**Atom**	***x***	***y***	***z***	**U(eq)**
H16	4503	2467	6771	35
H8	5169	4798	7495	33
H12	11222	3430	6097	39
H6	7492	1922	8149	37
H9A	7445	5470	8400	37
H9B	8951	4294	8333	37
H4	2766	4344	8315	37
H7	6047	607	8951	41
H3	1350	3038	9133	41
H10A	8335	6171	7273	42
H10B	10334	6122	7824	42
H14A	6629	1348	4651	47
H14B	7464	112	5051	47
H1A	2878	-7	9548	71
H1B	1131	1019	9661	71
H1C	3292	1050	10130	71

Experimental

Single crystals of $C_{17}H_{16}O_3$ [YYT-384] were []. A suitable crystal was selected and [] on a SuperNova, Dual, Cu at zero, AtlasS2 diffractometer. The crystal was kept at 185(20) K during data collection. Using Olex2 [1], the structure was solved with the ShelXT [2] structure solution program using Direct Methods and refined with the ShelXL [3] refinement package using Least Squares minimisation.

1. Dolomanov, O.V., Bourhis, L.J., Gildea, R.J, Howard, J.A.K. & Puschmann, H. (2009), J. Appl. Cryst. 42, 339-341.
2. Sheldrick, G.M. (2008). Acta Cryst. A64, 112-122.
3. Sheldrick, G.M. (2008). Acta Cryst. A64, 112-122.

Crystal structure determination of [YYT-384]

Crystal Data for $C_{17}H_{16}O_3$ (M =268.30 g/mol): monoclinic, space group P2$_1$/c (no. 14), a = 6.4369(3) Å, b = 10.7530(5) Å, c = 19.1181(10) Å, β = 94.173(5)°, V = 1319.77(12) Å^3, Z = 4, T = 185(20) K, μ(CuKα) = 0.743 mm^{-1}, $Dcalc$ = 1.350 g/cm^3, 4514 reflections measured (9.276° ≤ 2Θ ≤ 147.074°), 2595 unique (R_{int} = 0.0539, R_{sigma} = 0.0545) which were used in all calculations. The final R_1 was 0.0719 (I > 2σ(I)) and wR_2 was 0.2180 (all data).

Refinement model description

Number of restraints - 0, number of constraints - unknown.

Details:

1. Fixed Uiso

At 1.2 times of:

All C(H) groups, All C(H,H) groups

At 1.5 times of:

All C(H,H,H) groups

2.a Ternary CH refined with riding coordinates:

C8(H8)

2.b Secondary CH2 refined with riding coordinates:

C9(H9A,H9B), C10(H10A,H10B), C14(H14A,H14B)

2.c Aromatic/amide H refined with riding coordinates:

C16(H16), C12(H12), C6(H6), C4(H4), C7(H7), C3(H3)

2.d Idealised Me refined as rotating group:

C1(H1A,H1B,H1C)

This report has been created with Olex2, compiled on 2014.11.28 svn.r3106 for OlexSys. Please let us know if there are any errors or if you would like to have additional features.
